# Sctensor detects many-to-many cell–cell interactions from single cell RNA-sequencing data

**DOI:** 10.1186/s12859-023-05490-y

**Published:** 2023-11-07

**Authors:** Koki Tsuyuzaki, Manabu Ishii, Itoshi Nikaido

**Affiliations:** 1 Laboratory for Bioinformatics Research RIKEN Center for Biosystems Dynamics Research, 2-1 Hirosawa, Wako, Saitama, 351-0198 Japan; 2 Japan Science and Technology Agency, PRESTO, 7 Gobancho, Chiyoda-ku, Tokyo, 102-0076 Japan; 3 Department of Functional Genome Informatics, Division of Biological Data Science, Medical Research Institute, Tokyo Medical and Dental University (TMDU), 1-5-45 Yushima, Bunkyo-ku, Tokyo, 113-8510 Japan

**Keywords:** Single-cell RNA-sequencing, Cell–cell interaction, Hypergraph, Dimension reduction, Tensor decomposition, Non-negative Tucker2 decomposition, R/Bioconductor

## Abstract

**Background:**

Complex biological systems are described as a multitude of cell–cell interactions (CCIs). Recent single-cell RNA-sequencing studies focus on CCIs based on ligand–receptor (L–R) gene co-expression but the analytical methods are not appropriate to detect many-to-many CCIs.

**Results:**

In this work, we propose scTensor, a novel method for extracting representative triadic relationships (or hypergraphs), which include ligand-expression, receptor-expression, and related L–R pairs.

**Conclusions:**

Through extensive studies with simulated and empirical datasets, we have shown that scTensor can detect some hypergraphs that cannot be detected using conventional CCI detection methods, especially when they include many-to-many relationships. scTensor is implemented as a freely available R/Bioconductor package.

**Supplementary Information:**

The online version contains supplementary material available at 10.1186/s12859-023-05490-y.

## Background

Complex biological systems and processes such as tissue homeostasis [1, 2], neurotransmission [3, 4], immune response [5], ontogenesis [6], and stem cell niches niche [7, 8] are composed of cell–cell interactions (CCIs). Many molecular biology studies have decomposed such systems into constituent parts (e.g., genes, proteins, and metabolites) to clarify their functions. Nevertheless, more sophisticated methodologies are required because CCIs essentially differentiate whole systems from functioning merely as the sum of their parts. Accordingly, micro-level measurements of such parts cannot always explain macro-level biological functions.

Previous studies have investigated CCIs using technologies such as fluorescence microscopy [9–13], microdevice-based methods such as microwells, micropatterns, single-cell traps, droplet microfluidics, and micropillars [14–22], and transcriptome-based methods [23–52]. In particular, the recent single-cell RNA-sequencing (scRNA-seq) studies have focused on CCIs based on ligand–receptor (L–R) gene co-expression. By investigating the detected cell types through scRNA-seq and the L–R pairs specifically expressed in the cell types, CCIs can potentially be understood at high resolution.

Despite their wide usage, the analytical methods based on L–R pairs are still not mature; such methods implicitly assume that CCIs consist of one-to-one relationships between two cell types and that the corresponding L–R co-expression is observed in a cell-type-specific manner. One study even removed ligand and receptor genes expressed in multiple cell types from their data matrix, assuming one-to-one CCIs [53]. In real empirical data, however, each ligand and receptor gene can be expressed across multiple cell types, and some studies have actually focused on many-to-many CCIs [25, 33, 36, 48, 54]. Such a difference between actual CCI patterns composed of real data and the hypothesis assumed by a model will cause severe bias in the detection of CCIs.

For the above reason, we propose scTensor, which is a novel CCI prediction method based on a tensor decomposition algorithm. Our method regards CCIs as hypergraphs and extracts some representative triadic relationships consisting of ligand-expression, receptor-expression, and related L–R pairs. The main contributions of this article are summarized as follows.

- We developed a novel simulator to model the CCIs as hypergraphs and quantitatively evaluate the performance of scTensor and other L–R detection methods.
- We re-implement some L–R detection methods from scratch in order to analyze the same L–R database with all of these methods and focus on only the performance of L–R detection methods, not the slight differences in data pre-processing and the L–R database used.
- We show that scTensor’s performance with respect to its accuracy of many-to-many CCI detection, computation time, and memory usage are superior to the other L–R detection methods.
- We describe the implementation of scTensor as an R/Bioconductor package to enable the reproducibility of data analyses as well as continuous maintenance and improvements. We provide some original visualization functions and a function to generate an HTML report in scTensor to enable detailed interpretation of the results. We have extended our framework to work with 125 species.

## Results

### CCI as a hypergraph

**Fig. 1:**
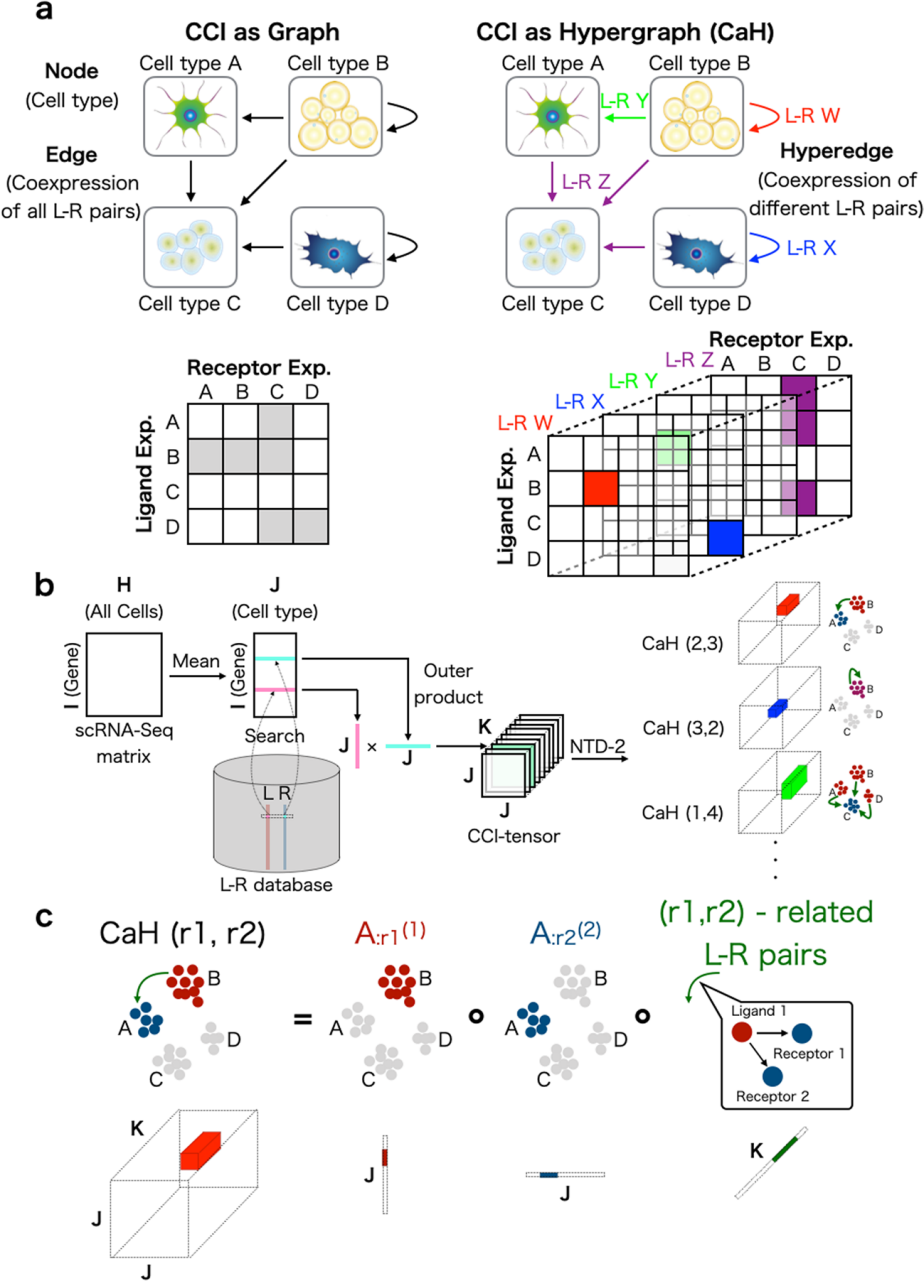
Cell–cell interaction (CCI) as a hypergraph (CaH). **a** Previous scRNA-seq studies have regarded CCIs as graphs, and the corresponding data structure can be expressed as an adjacency matrix (left). In this work, CCIs are regarded as context-aware edges (hypergraphs), and the corresponding data structure is a tensor (right). **b** The CCI-tensor is generated by users’ scRNA-Seq matrices, cell-type labels, and ligand–receptor (L–R) databases. NTD-2 is used to extract CaHs from the CCI-tensor. **c** Each CaH(r1,r2) is equal to the outer product of three vectors. $A_{:r1}^{(1)}$ represents the ligand expression pattern, $A_{:r2}^{(2)}$ represents the receptor expression pattern, and $G_{r1,r2,:}$ represents the patterns of related L–R pairs

One of the simplest CCI representations is a directed graph, where each node represents a cell type and each edge represents the co-expression of all L–R pairs (Fig. 1a, left). The direction of each edge is set as the ligand expressing cell type $\rightarrow$ the receptor-expressing cell type. Such a data structure corresponds to an asymmetric adjacency matrix, in which each row and column represents a ligand-expressing cell type and receptor-expressing cell type, respectively. If some combinations of cell types are regarded as interacting, the corresponding elements of the matrix are filled with 1 and otherwise 0. If the degree of CCI is not a binary relationship, weighted graphs and corresponding weighted adjacent matrices may also be used. The previous analytical methods are categorized within this approach [23, 24, 26–34, 36, 40, 43–46, 48, 49, 51, 52, 55].

The drawback of using an adjacency matrix to describe CCIs is that multiple L–R co-expression scores are collapsed into a single value by summation or averaging. Because the average is simply a constant multiple of the sum, here we discuss only the sum. The summed value has no meaning in which L–R pairs are related to the CCI, and therefore CCIs and the related L–R pair lists cannot be detected simultaneously.

In contrast to an adjacency matrix (i.e., graph), the triadic relationship of CCIs also can be described as directed hypergraphs (i.e., CCI as hypergraph; CaH), where each node is a cell type but the edges are distinguished from each other by the different related L–R pair sets (Fig. 1a, right). Such a context-aware edge is called a “hyperedge” and is described as multiple different adjacency matrices. The set of matrices corresponds to a “tensor”, which is a generalization of a matrix to expand its order.

### Overview of **scTensor**

Here we introduce the procedure of scTensor. Firstly, a tensor data is constructed through the following steps (Fig. 1b). A scRNA-seq matrix and the cellular labels specifying cell types are supposed to be provided by users. Firstly, the gene expression values of each cell are normalized by count per median of library size (CPMED [56–58]) and logarithm transformation, for variance-stabilization, is performed to the data matrix [i.e., $\log _{10}{(\textrm{CPMED} + 1)}$].

Next, the data matrix is converted to a cell-type-level average matrix according to the cell type labels. Combined with an L–R database, two corresponding row-vectors of an L–R pair are extracted from the matrix. The outer product (direct product) of the two vectors is calculated, and a matrix is generated. The matrix can be considered as the similarity matrix of all possible cell-type combinations for each L–R pair. Finally, for each L–R pair, the matrix is calculated, and the tensor $\mathcal {\chi } \in \mathbb {R}^{J \times J \times K}$, where *J* is the number of cell types and *K* is the number of L–R pairs, is generated as the merged matrices. In this work, this tensor is called the “CCI-tensor”.

After the construction of the CCI-tensor, we use the non-negative Tucker2 decomposition (NTD-2) algorithm [59, 60]. NTD-2 decomposes the CCI-tensor as a core tensor $\mathcal {G} \in \mathbb {R}^{R1 \times R2 \times K}$, and two factor matrices $\varvec{A}^{\left( 1\right) } \in \mathbb {R}^{J \times R1}$ and $\varvec{A}^{\left( 2\right) } \in \mathbb {R}^{J \times R2}$, where *R*1 and *R*2 are the NTD-2 rank parameters. The factor matrix $\varvec{A}^{\left( 1\right) }$ describes the *R*1 of ligand gene expression patterns in each cell type and the factor matrix $\varvec{A}^{\left( 2\right) }$ describes *R*2 of receptor gene expression patterns in each cell type, and core tensor $\mathcal {G}$ describes the degree of association of all the combination ($R1 \times R2$) of the ligand and receptor expression patterns of each L–R pair.

The result of NTD-2 is considered the sum of some representative triadic relationships. In this work, each triadic relationship is termed $\texttt {CaH}{}\left( r1,r2\right)$, which refers to the outer product of three vectors, $\varvec{A}_{:r1}^{\left( 1\right) }$, $\varvec{A}_{:r2}^{\left( 2\right) }$, and $\mathcal {G}_{r1,r2,:}$, where *r*1 ($1 \le r1 \le R1$) and *r*2 ($1 \le r2 \le R2$) are the indices of the columns of the two factor matrices (Fig. 1c). The CaHs are extracted in a data-driven way without the assumption of one-to-one CCIs. Therefore, this approach can also detect many-to-many CCIs according to the data complexity.

### Evaluation of many-to-many CCIs detection

**Fig. 2:**
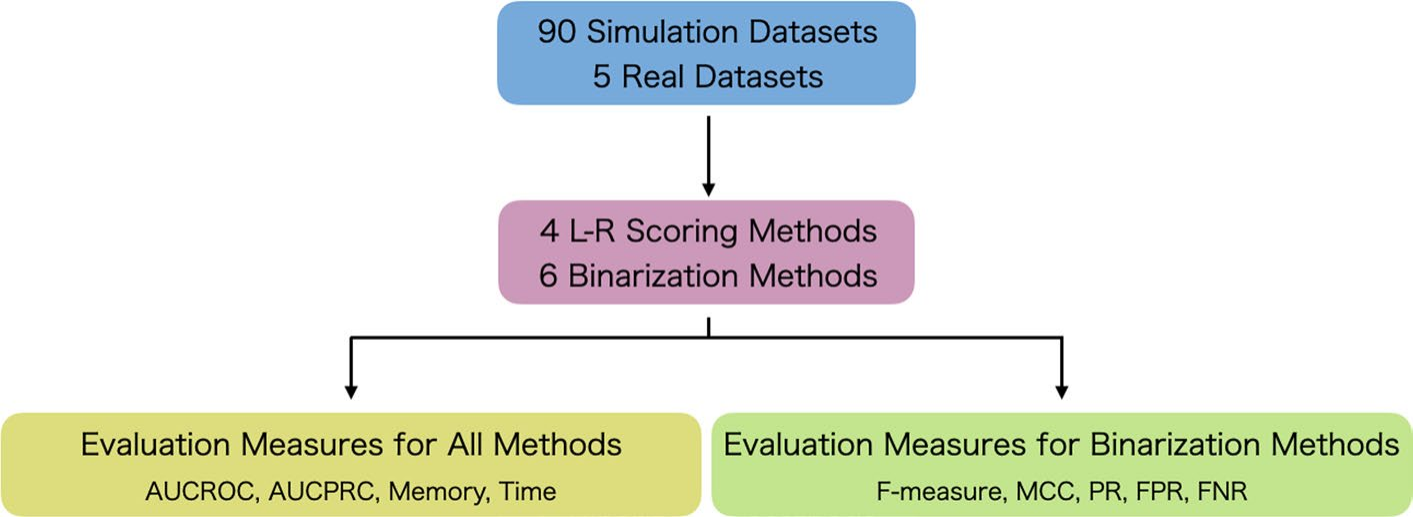
Evaluation scheme. To evaluate CCI methods, 90 simulated datasets and six real empirical datasets were prepared. Four ligand–receptor (L–R) scoring methods and six binarization methods were then evaluated. For the evaluation of these methods, area under the receiver operating characteristic curve (AUCROC), area under the precision–recall curve (AUCPRC), memory usage, and computational time were determined. For the evaluation of binarization methods, F-measure, Matthews correlation coefficient (MCC), positive ratio (PR), false positive ratio (FPR), and false negative ratio (FNR) were determined

To examine the performance of the CCI methods in terms of detecting CaH, we validated the CCI methods using both simulated and empirical datasets (Fig. 2).

We first prepared 90 simulated datasets considering five numbers of cell types (3, 5, 10, 20, or 30), two CCI styles (one-to-one or many-to-many including one-to-many and many-to-one), three numbers of CCI types (1, 3, or 5), and three threshold values (E2, E5, or E10) for recognition of differentially expressed genes (DEGs). According to these conditions, ground truth CCIs were determined (Additional file 1).

Next, we prepared five real empirical datasets (FetalKidney [36], GermlineFemale [25], HeadandNeckCancer [54], Uterus [33], and VisualCortex [48]), each of which focused on many-to-many CCIs in their respective original papers.

There are many L–R scoring methods to quantify the degree of co-expression of ligand and receptor genes. We re-implemented four scores used in many CCI prediction methods to evaluate performance independent of software implementation (Table 1 and Additional file 1). Here, we selected as the four methods sum score (CellPhoneDB [37], Giotto [61], CrossTalkR [62], and Squidpy [63]), product score (NATME [64], FunRes [65], ICELLNET [66], and TraSig [67]) Halpern’s score [68], and Cabello $\mathrm {-}$ Aguilar’s score (SingleCellSignalR [69] and CellTalkDB [70]), each of which is widely used in many studies.

To differentiate significant CCIs from non-significant CCIs, many CCI methods introduce a label permutation test with a random permutation of cell-type labels to simulate the null distribution of CCIs. This process is considered a kind of binarization (1 for significant CCIs, 0 for non-significant CCIs). For scTensor, binarization was realized by median absolute deviation (MAD) thresholding against each column vector in factor matrices calculated by tensor decomposition.

To quantitatively evaluate how selectively each CCI method was able to detect the ground truth CCIs before and after binarization, nine evaluation measures were introduced. Four of them were applied both before and after binarization, and the remaining five were applied to the results only after binarization.

#### **scTensor** could selectively detect many-to-many CCIs in simulated datasets

**Fig. 3:**
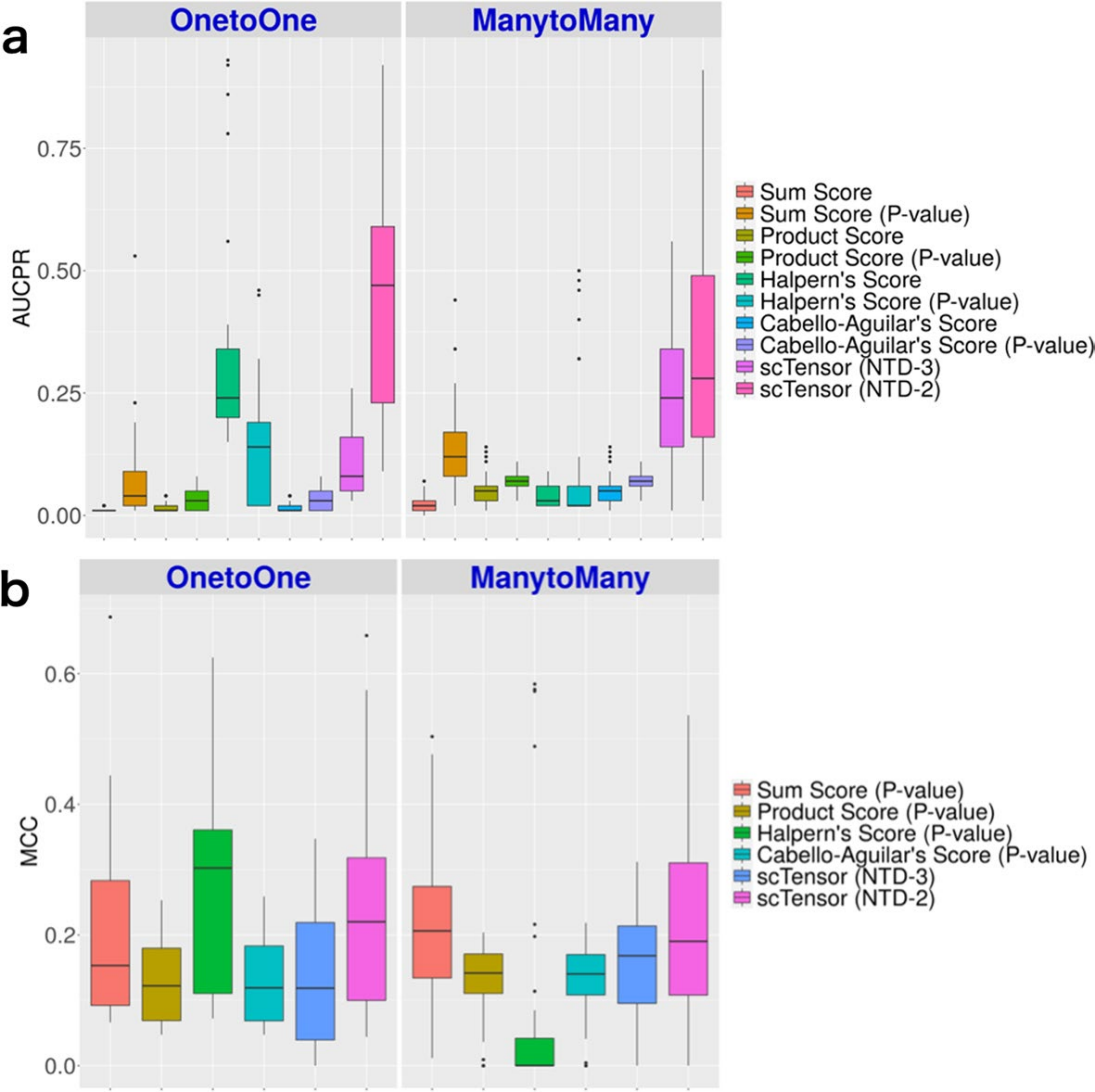
Results of simulated datasets. **a** Area under the curve of precision–recall (AUCPR) of all the methods. **b** Matthews correlation coefficient (MCC) of the binarization methods

The area under the curve of precision–recall (AUCPR) and Matthews correlation coefficient (MCC) values of 30 datasets with an E10 threshold value are shown in Fig. 3. For the details of all the evaluation results for all the conditions, see Additional files 3–12. Figure 3 shows that the AUCPR values can vary among the CCI methods. When the CCI-style was one-to-one (Fig. 3a, left), scTensor (NTD-2) achieved the highest AUCPR scores, and Halpern’s score obtained the second-highest AUCPR values on average. For Halpern’s score, however, binarization has significantly reduced the significant CCIs. This may be explained by Halpern’s score having the lowest FPR (Additional file 10) and the highest FNR (Additional file 11), and it suggests that Halpern’s score is a quite conservative method to detect one-to-one CCIs. When the CCI-style was set as many-to-many, both the previous and current versions of scTensor (NTD-3 and NTD-2, respectively) achieved higher AUCPR values on average (Additional file 4), compared with the other methods (Fig. 3a, right).

Figure 3b shows that the MCC values also varied among the CCI methods. When the CCI-style was one-to-one (Fig. 3b, left), Halpern’s score achieved the highest MCC values and scTensor (NTD-2) obtained the second-highest values on average (Additional file 8). When the CCI-style was many-to-many, scTensor (NTD-2) and sum score obtained the highest MCC values, compared with the other methods (Fig. 3b, right).

#### Characteristics of **scTensor** (NTD-2), **Halpern’s score**, and **sum score**

**Fig. 4:**
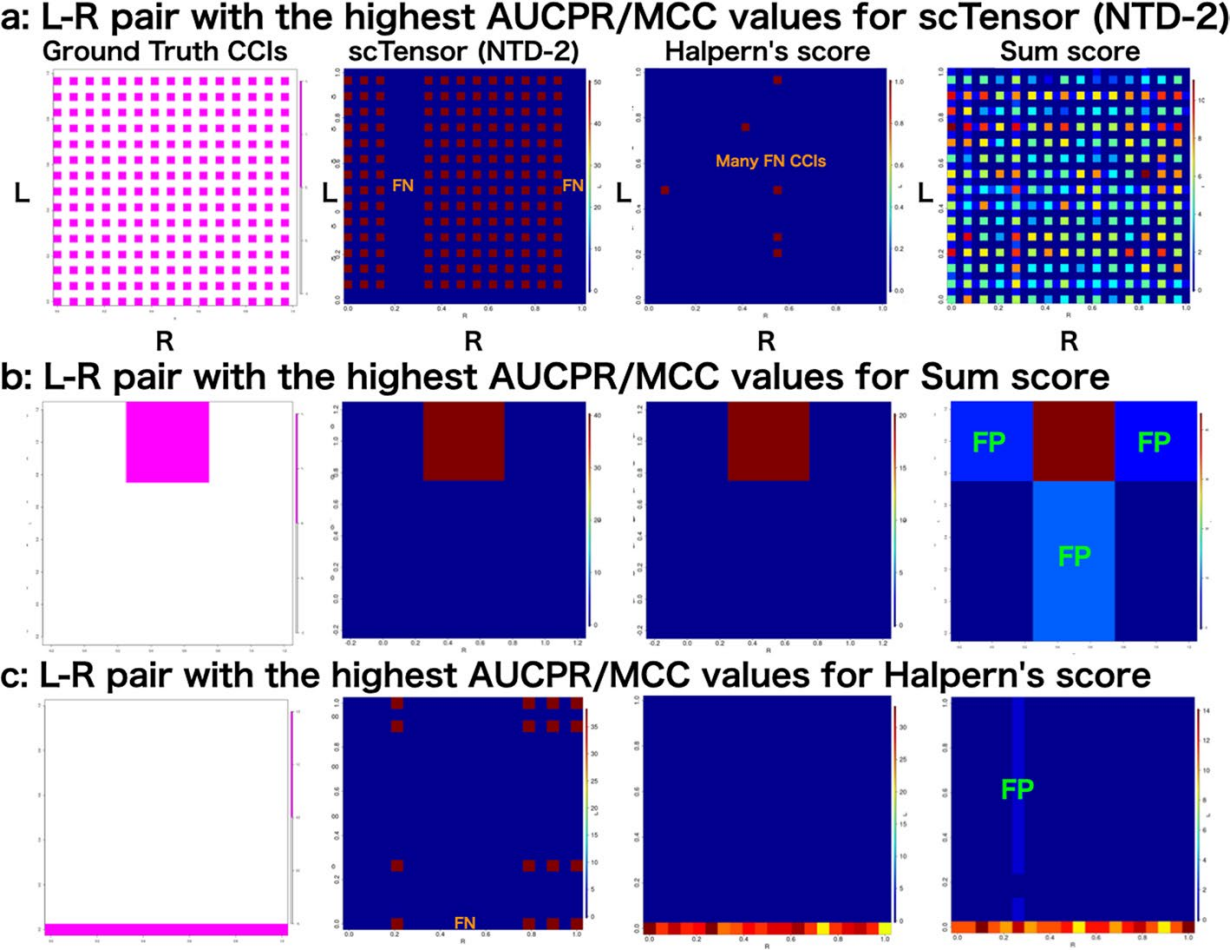
Analyses of three datasets in which each of the three methods excelled. Summary of the number of significant cell–cell interactions (CCIs) with **a** three cell types, one CCI types, one-to-one CCI style, and 1st-CCI type; **b** 20 cell types, five CCI types, many-to-many CCI style, 2nd-CCI type; and **c** 30 cell types, five CCI types, many-to-many CCI style, 5th-CCI type. The *y*-axis (L) and *x*-axis (R) indicate the ligand-expressing cell types and the receptor-expressing cell types, respectively. FN and FP indicate false negative and false positive CCIs, respectively

The comprehensive validation described that the three methods (scTensor (NTD-2), Halpern’s score, and sum score) performed better than the others under certain conditions. To further examine the characteristics and trends of each method, we aggregated the number of CCIs detected in three datasets in which each of the three methods excelled (Fig. 4).

scTensor (NTD-2): this method performed well when the CCI style was many-to-many. For example, in Fig. 4a, most many-to-many CCIs could be detected. Although there were some false negative (FN) CCIs that were not detected, there were fewer false positive (FP) CCIs. In contrast, Halpern’s score was too conservative against this dataset and failed to detect most of the CCIs by the label permutation test. At a first glance of Fig. 4a, the sum score appears to work well with these data, but under scrutiny at the level of individual L–R pairs, sum score results contain many FP and FN CCIs (Additional file 13).

Sum score: This method performed well when the number of cell types was small and the style of CCI was restricted to one-to-one (Fig. 4b). Even though Halpern’s score and scTensor (NTD-2) were able to detect similar CCIs, Halpern’s score was quite conservative and contained many FN CCIs because it considered many CCIs to not be significant. For sum score, there seemed to be a bias toward FP CCIs. If the degree of co-expression of an L–R pair is high between two cell types, this method seems to detect FP pairs in which only one of the L–R is highly expressed. In such cases, cross-shaped patterns were observed in the heatmap in Fig. 4. In our simulated datasets, this cross-shaped pattern of FP CCIs were observed more frequently in the sum score.

Halpern’s score: In most data sets, Halpern’s score was found to be too conservative, with many FN CCIs, but in a very specific situation, that is, when the CCI-style was one-to-all (or all-to-one), it outperformed the other methods (Fig. 4c). In contrast, scTensor (NTD-2) inferred many FN CCIs among these data, while the sum score identified many FP CCIs (Additional file 13).

#### **scTensor** could selectively detect many-to-many CCIs in real datasets

**Fig. 5:**
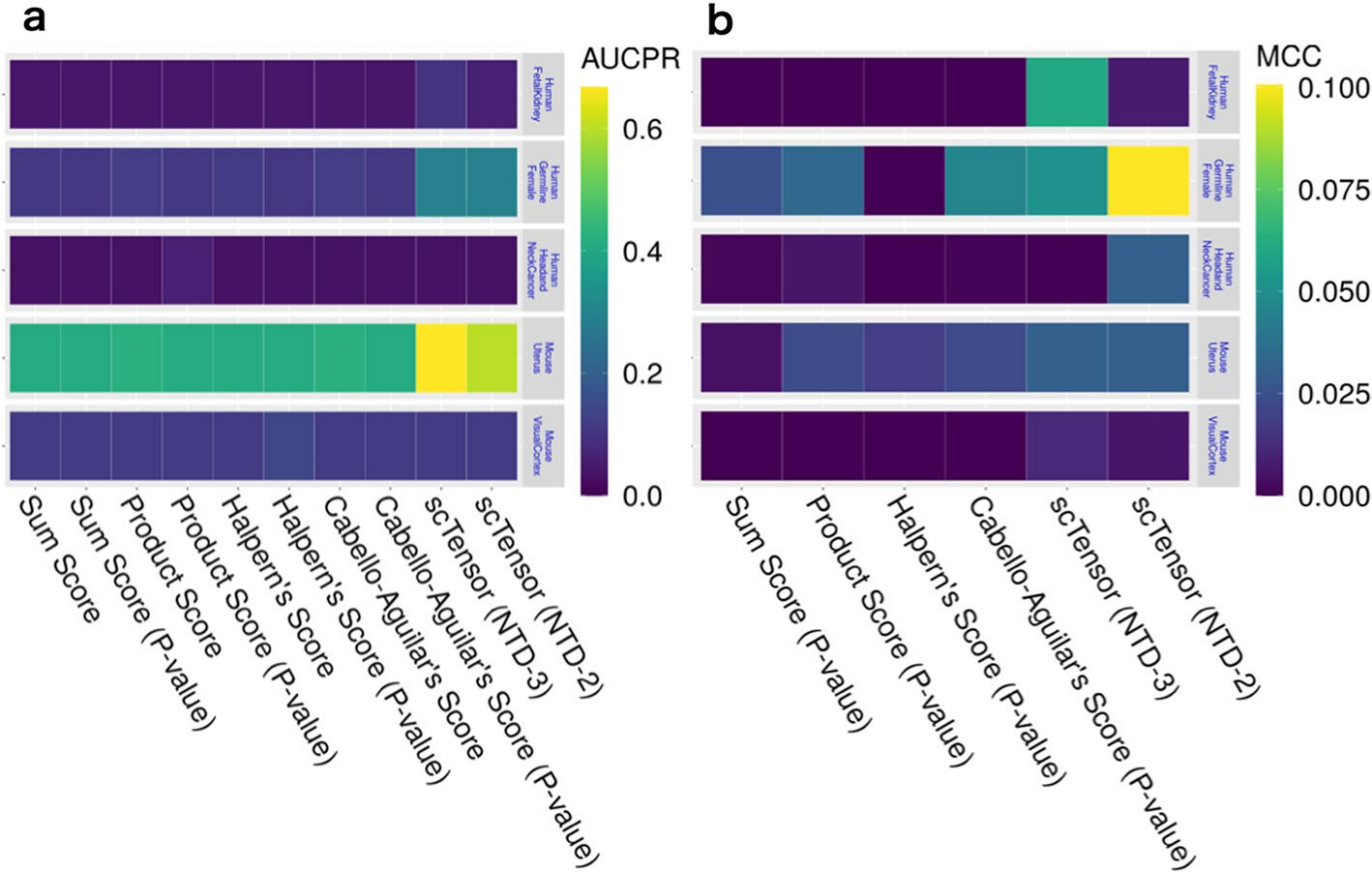
Cell–cell interaction (CCI) identification results from real empirical datasets. **a** Area under the curve of precision–recall (AUCPR) of all the methods. **b** Matthews correlation coefficient (MCC) of the binarization methods

Next, we applied these CCI methods to real empirical datasets (Table 2 and Additional files 3–12). As expected from the results of simulated datasets, scTensor (NTD-2) outperformed the other methods on these real datasets, which contain many-to-many CCIs. Regarding AUCPR (Fig. 5a) and MCC (Fig. 5b) values, scTensor (NTD-2) achieved higher values compared with the other methods, although the difficulty of detecting the CCIs was highly dependent on the dataset (Additional files 4, 8). We further investigated the real empirical datasets and found that the known CCIs reported by the original papers were reproduced by scTensor (Table 3). Additionally, some predicted many-to-many CCIs can be considered biologically plausible because the CCIs are related to the same signaling pathways of known CCIs, although the original papers did not refer to the CCIs. These results can be interactively investigated using the HTML report generated by scTensor (Additional files 13–17).

#### Computational complexity and memory usage

We also assessed the orders of computational complexity and memory usage of all the CCI methods (Table 4). All the L–R score methods require $\mathcal {O}(N^{2}L)$ order both in the computation and in memory usage, where *N* is the number of cell types and *L* is the number of L–R pairs. The label permutation tests combining any L–R scores require $\mathcal {O}(N^{2}LP)$ in computation, where *P* is the number of random shuffles of cell-type labels. In many cases, *P* is typically set as a large value greater than 1000 [37, 69], making this a very time-consuming calculation.

In contrast, scTensor (NTD-2) does not perform the label permutation; instead, it simply utilizes the factor matrices after the decomposition of the CCI-tensor. Hence, the order of computational complexity is reduced to $\mathcal {O}(N^{2}L(R1+R2))$, where *R*1 and *R*2 are the number of columns or “rank” parameters for the first- and second-factor matrices, respectively. The rank parameters are typically set as small numbers (e.g., 10), this leads to a substantial computational advantage compared with the label permutation test. The computation time and memory usage when analyzing the simulated and real empirical datasets show that scTensor has an advantage in computational complexity compared with the label permutation test (Additional files 5, 6).

### Method comparisons

The first method similar to scTensor is CellChat. This method uses the communication probabilities (3rd-order tensor), which is a CCI tensor constructed with the authors’ original score and is normalized so that the sum in the second mode is 1. In addition to the label permutation test on each L–R pair in the third-order tensor, NMF on the matrix data summarized by the second mode of the 3rd-order tensor is performed to detect global CCI patterns. However, this summarization reduces the order of tensor (i.e., 3rd to 2nd) and loses information on which L–R pairs contributed to the CCI. In particular, as this study has shown, the label permutation test tends to detect one-to-one CCI, whereas NMF may also detect many-vs-many CCI, making it difficult to consider many-vs-many CCI and the L–R pairs that contribute to it simultaneously, even when the two methods are combined. Therefore, models like scTensor that can handle higher-order data as is are preferable.

Inspired by our method, another method Tensor-cell2cell [71] extended our approach to higher-order CCI tensors (e.g., 4th-order tensors) to consider CCIs and the CCI contexts (e.g., disease state, organismal life stage, and tissue microenvironment) simultaneously. Other than its effectiveness for such a higher-order CCI tensor, the main differences between Tensor-cell2cell and scTensor may include the following. First, Tensor-cell2cell is implemented by Python but scTensor is implemented by R. Python offers a wide range of machine learning/deep learning packages, while R offers data preprocessing and visualization with TidyVerse and bioinformatics-related packages with Bioconductor.

Second, there are differences in tensor decomposition models; Tensor-cell2cell performs CANDECOMP/PARAFAC-type non-negative tensor decomposition but scTensor’s model is NTD-2. The difference between these models is the number of rank parameters. Tensor-cell2cell has only one rank parameter, while scTensor has rank parameters for the number of tensor orders (i.e. 3). This difference can be an advantage or a disadvantage; a small number of ranks reduces the computational time required to estimate the optimal ranks but it might make the model too simple.

**Fig. 6:**
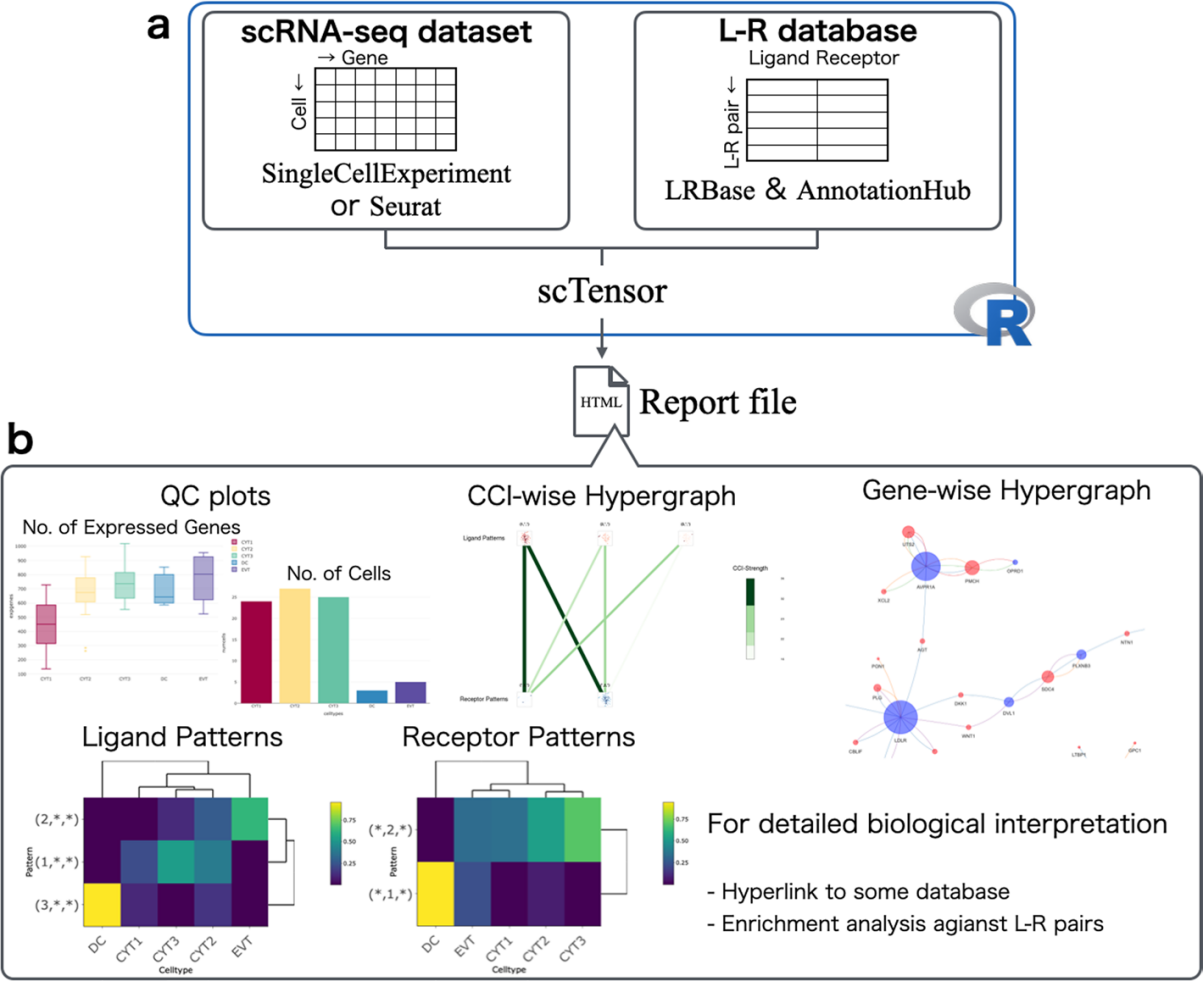
Implementation of the scTensor package. **a** scTensor is an R package that requires the input of both an scRNA-Seq expression matrix (SingleCellExperiment or Seurat) and a ligand–receptor (L–R) database (LRBase). The LRBase is retrieved from the AnnotationHub remote server, after which a LRBase object is created. **b** Using these objects, scTensor generates an HTML report file, and the results of cell–cell interaction (CCI) analysis can be visualized with a wide variety of plots

Third, there are differences in the related tools. Tensor-cell2cell assumes their text file for input, it only supports major species such as mouse and human, and it seems to assume to be used with LIANA [72, 73], another CCI tool of the authors. On the other hand, scTensor supports 124 species (September 5, 2024) in the Bioconductor package LRBase, and can be combined with various other single-cell packages via the SingleCellExperiment object and Seurat (see Implementations and Fig. 6). With an understanding of these differences, users should choose the tool they want to use according to what they want to do.

### Implementations

scTensor is implemented as an R/Bioconductor package that is freely available. Both a scRNA-seq dataset and L–R database are required for scTensor execution. The default format for a scRNA-seq dataset is SingleCellExperiment, in which the gene IDs correspond to NCBI’s Gene database to allow links with other databases (Fig. 6a). A scRNA-seq dataset can also be converted from a Seurat object. We provided instructions for this data conversion (https://bioconductor.org/packages/release/bioc/vignettes/scTensor/inst/doc/scTensor_1_Data_format_ID_Conversion.html#case-iii-umi-count).

LRBase, which is the L–R database for scTensor, is stored on a remote server called AnnotationHub and is downloaded to the user’s machine on demand, only when called by the user (Fig. 6a). To extend out method to a wide range of organisms, in this work, we originally constructed and are providing the L–R lists for 125 organisms (https://github.com/rikenbit/lrbase-workflow/blob/master/sample_sheet/sample_sheet.csv). The details of the data processing pipeline are summarized in the README.md of lrbase-workflow (https://github.com/rikenbit/lrbase-workflow), which is a workflow for constructing the LRBase for each of the species. For data sustainability, we offer the data files, including older versions, on the AnnotationHub server. The data files are bi-annually updated in conjunction with Bioconductor updates and are provided using lrbase-workflow. Users can specify which version of the data is used for analysis, thus ensuring data reproducibility.

NTD-2 was implemented as within the function of nnTensor [74] R/CRAN package and internally imported into scTensor. scTensor constructs the CCI-tensor, decomposes the tensor by the NTD-2 algorithm, and generates an HTML report.

To enhance the biological interpretation of CaH results, we implemented some visualization functions (Fig. 6a) and these plots can be interactively investigated via web browser. A wide variety of gene-wise information is included in the report and can be linked to the L–R lists through the use of other R/Bioconductor packages; the gene annotation is assigned by biomaRt [75] (Gene Name, Description, Gene Ontology [GO], STRING, and UniProtKB), reactome.db [76] (Reactome) and MeSH.XXX.eg.db [77] (Medical Subject Headings [MeSH]), while the enrichment analysis (also known as over-representative analysis [ORA]) is performed by GOstats [78] (GO-ORA), meshr [77] (MeSH-ORA), ReactomePA [79] (Reactome-ORA), and DOSE [80] (Disease Ontology (DO)-ORA, Network of Cancer Genes (NCG)-ORA, DisGeNET-ORA).

To validate that the detected the co-expression of L–R gene pairs is also consistently detected in the other data including tissue- or cell-type-level transcriptome data, the hyperlinks to RefEx [81], Expression Atlas [82], SingleCell Expression Atlas [83], scRNASeqDB [84], and PanglaoDB [85] are embedded in the HTML report, facilitating comparisons of the L–R expression results with the data from large-scale genomics projects such as GTEx [86], FANTOM5 [87], the NIH Epigenomics Roadmap [88], ENCODE [89], and the Human Protein Atlas [90]. Additionally, in consideration of users who might want to experimentally investigate detected CCIs, we embedded hyperlinks to Connectivity Map (CMap [91]), which provides relationships between perturbations by the addition of particular chemical compounds/genetic reagents and the resulting gene expression change.

## Discussion

In this work, we regarded CCIs as CaHs, which represent the triadic relationships of ligand-expressing cell types, receptor-expressing cell types, and the related L–R pairs. We implemented a novel algorithm scTensor based on a tensor decomposition algorithm for detecting such CaHs. Our evaluations using both simulated and real empirical datasets suggest that scTensor can detect many-to-many CCIs more accurately than the other conventional CCI methods. Additionally, the calculation time and memory usage performances of scTensor are also superior to those of the other CCI methods.

To extend the use of scTensor to a wide range of organisms, we also created multiple L–R datasets for 125 organisms. scTensor has been published as an R/Bioconductor package, facilitating the reproducibility of data analysis and the maintainability of datasets. We also implemented an HTML report function that simplifies checking the analysis results of scTensor. Like many CCI tools, scTensor can import an external L–R database.

In the development of many CCI tools, the authors also develop their own L–R databases and investigate the differences among various L–R databases, particulaly when comparing their method with other conventional methods [72]. This makes it difficult to distinguish whether the performance of a method is caused by differences in algorithms or databases. Although the primary CCI resources used for existing L–R tools are highly duplicated, even slight differences can influence the detection of CCIs [72]. Therefore, to separate these two comparisons and to focus only on the algorithmic differences, in this work, we compared several existing CCI algorithms, by re-implementing them and anchoring them to a common L–R database.

We were also able to examine several strengths and weaknesses of the methods other than scTensor. For example, Halpern’s score was found to be too conservative with many FN CCIs, but it was superior to the other methods with respect to the detection of one-to-all (or all-to-one) CCIs. A possible reason for this is that since the formula for this score includes the square root of the chi-square distribution with two degree of freedom (or an exponential distribution with an expected value of 2), and these distributions are known to be heavy-tailed to some extent, thus potentially inflating the number of significant L–R pairs.

The permutation test implicitly assumes that the interactions occur between very few cell types because the larger the observed L–R score is than the empirical distribution computed by label permutation, the more significant the test result is. However, if the expression levels of ligand and receptor genes are high in any cell of any cell type, the L–R scores calculated by the label permutation are will also be high, and thus, the observed value of the L–R score will be regarded as not a particularly high value in the empirical distribution; consequently, such a test result will be not significant. Therefore, detection of many-to-many CCIs by label permutation test is difficult in principle. In the extreme case of all-to-all CCIs, the current approaches (although it also includes scTensor) cannot avoid FN CCIs.

There are still some plans to improve scTensor to build on the advantages of this current framework. For example, the algorithm can be improved by utilizing acceleration techniques such as randomized algorithm/sketching methods [92], incremental algorithm/stochastic optimization [93, 94], or distributed computing on large-scale memory machines [95] for tensor decomposition, as is now available.

To reduce the memory usage of scTensor, we are developing DelayedTensor [96], which is an R/Bioconductor package to perform various tensor arithmetic and tensor decomposition algorithms based on DelayedArray [97], another R/Bioconductor package for handling out-of-core multidimensional arrays in R. We intend to reduce the memory usage of scTensor by supporting this data format.

Tensor data formats are very flexible ways to represent heterogeneous biological data structures [98], because they easily integrate supplemental information about genes or cell types in a semi-supervised manner. Such information could extend the scope of the data and thus improve the accuracy of inferences. For example, there are some attempts to use the following additional information for CCI detection as well (for more details, see Additional file 1).

- CCI inference via receptor-receptor and extracellular matrix data [51, 99].
- Consideration of multi-subunit complexes [37].
- Comparison of CCIs across multiple conditions [62, 71, 100–106].
- Consideration of downstream transcriptional factors, target genes, and signaling pathways [69, 107–109].
- Integration with bulk RNA-Seq or other type of omics datasets [37, 64, 65, 107–110].
- Integration with pseudo-time [67, 111, 112].
- Integration with spatial transcriptome data [113].

In particular, in a recent benchmark study [113], the proximity of spatial coordinates on tissue sections measured by spatial transcriptome technology and the CCI detected by L–R data were correlated, and some studies have attempted to integrate these two kinds of datasets a single model ([113] and Additional file 1). Auxiliary Information such as the proximity in spatial coordinates can be incorporated as a regularization term to extend the tensor decomposition model [114–116].

Although it is beyond the scope of the present paper to cover all of the above-mentioned topics, considering these in the framework of tensor decomposition is a promising research direction, so we aim to continuously work on these through the development of updates and releases of scTensor for Bioconductor.

## Conclusion

In this work, we present and evaluate scTensor, a new method for detecting CCIs based on L–R co-expression in scRNA-seq datasets. We also revealed that the widely used label permutation test has a bias that impedes the detection of many-to-many CCIs and demonstrated that the proposed method is a viable alternative.

## Materials and methods

### Simulated datasets

The simulated single-cell gene expression data were sampled from the negative binomial distribution $NB\left( f_{gc}m_{g},\phi _{g}\right)$, where $f_{gc}$ is the fold-change (FC) for gene *g* and cell type *c*, and $m_{g}$ and $\phi _{g}$ are the average gene expression and the dispersion parameter of the expression of gene *g*, respectively.

The $m_{g}$ value and gene-wise variance $v_{g}$ were calculated from a real scRNA-seq dataset of mouse embryonic stem cells (mESCs) measured by Quartz-Seq [117], and the gene-wise dispersion parameter $\phi _{g}$ was estimated as $\phi _{g} = \left( v_{g} - m_{g}\right) /m_{g}^2$.

For the determination of differentially expressed genes (DEGs) and non-DEGs, $f_{gc}$ values were calculated based on the non-linear relationship of FC and the gene expression level $\log _{10}{f_{gc}} = a \exp (-b \log _{10}{\left( m_{g}+1\right) })$. To estimate the parameters *a* and *b*, we detected the DEGs using edgeR. By setting the threshold values (i.e., false discovery rate) of edgeR as $10^{-2}$ (E2), $10^{-5}$ (E5), and $10^{-10}$ (E10) and using the resulting DEGs, *a* and *b* values for each threshold were estimated as (0.701, 0.363), (1.907, 0.666), and (4.429, 0.814), respectively.

For genes identified as DEGs based on a threshold according to the non-linear relationship above, the estimated $f_{gc}$ value was used, otherwise, 1 is specified as $f_{gc}$. If a ligand gene of a cell type and a receptor gene of a cell type were both DEGs, we defined the relationship between these cell types as the ground truth CCIs and used them for quantitative evaluation.

To simulate the “dropout” phenomenon of scRNA-seq experiments, we also introduced the dropout probability $p_{dropout}^{gc} = \exp (-c f_{gc}m_{g}^2)$, which is used in ZIFA [118] (default, *c*=1), and the expression values were randomly converted to 0 according to the dropout probability.

To simulate various situations, we set many different CCI tensors, considering the number of cell types (3, 5, 10, 20, or 30), the style of CCIs (one-to-one or many-to-many including one-to-many and many-to-one), the number of types of CCIs (1, 3, or 5), and the DEG threshold value (E2, E5, or E10); in total, 90 synthetic CCI tensors were generated.

### scRNA-seq real datasets

The gene expression matrix of human FetalKidney data was retrieved from the GEO database (GSE109205), and only highly variable genes (HVGs: http://pklab.med.harvard.edu/scw2014/subpop_tutorial.html) with low $P$ values ($\le$ 1E−1) were extracted. The cell-type label data were provided by the authors upon our request.

The gene expression matrix of human GermlineFemale data was retrieved from the GEO database (GSE86146), and only HVGs with low $P$ values ($\le$ 1E−7) were extracted.

The gene expression matrix and the cell-type labels of human HeadandNeckCancer data were retrieved from the GEO database (GSE103322), and only HVGs with low $P$ values ($\le$ 1E−1) were extracted.

The gene expression matrix of mouse Uterus data was retrieved from the GEO database (GSE118180), and only HVGs with low $P$ values ($\le$ 1E−1) were extracted. The cell-type labels were provided by the authors upon our request.

The gene expression matrix and the cell-type labels of mouse VisualCortex data were retrieved from the GEO database (GSE102827), and only HVGs with low $P$ values ($\le$ 1E−1) were extracted.

The gene expression values of each cell are normalized by CPMED [56–58] and logarithm transformation, for variance-stabilization, is performed to the data matrix. For analyzing these real datasets, known L–R pairs in DLRP [119], IUPHAR [120], and HPMR [121] were searched in the data matrix. We defined the ground truth CCIs between two cell types if the CCIs were reported by the original studies. The L–R pairs associated with the CCIs were used for quantitative evaluation.

### **scTensor** algorithm

#### CCI-tensor construction

Here, data matrix $\varvec{Y} \in \mathbb {R}^{I \times H}$ is the gene expression matrix of scRNA-seq data, where *I* is the number of genes and *H* is the number of cells. Matrix $\varvec{Y}$ is converted into cell-type-wise average matrix $\varvec{X} \in \mathbb {R}^{I \times J}$, where *J* is the number of cell types. The cell-type labels are assumed to be specified by the user’s prior analysis, such as clustering or confirmation of marker gene expression. The relationship between the $\varvec{X}$ and $\varvec{Y}$ is described below:

1 $$\begin{aligned} \varvec{X} = \varvec{Y} \varvec{A}, \end{aligned}$$

where the matrix $\varvec{A} \in \mathbb {R}^{H \times J}$ converts cellular-level matrix $\varvec{Y}$ to cell-type-level matrix *X* and each element of ${\varvec{A}}$ is

2 $$\begin{aligned} {\varvec{A}}_{hj} = {\left\{ \begin{array}{ll} 1/n_{j} &{} \left( h\mathrm{th\,\, cell\,\, belongs \,\,to \,\,}j \mathrm{th \,\,cell \,\,type}\right) \\ 0 &{} \left( \text {otherwise}\right) , \end{array}\right. } \end{aligned}$$

where $n_{j}$ is the number of cells belonging to the *j*’s cell type.

Next, we search to determine whether any L–R pairs stored in the L–R database are both in the row names of matrix *X*, and if both IDs are found, corresponding *J*-length row-vectors of the ligand and receptor genes ($\varvec{x}_{L}$ and $\varvec{x}_{R}$) are extracted.

Finally, a ${J \times J}$ matrix is calculated as the outer product of $\varvec{x}_{L}$ and $\varvec{x}_{R}$ and incrementally stored. The stacked ${J \times J}$ matrices can be considered as a three-dimensional array, which is also known as a three-order tensor. The following outer product in the *k*th L–R pair ($L\left( k\right)$ and $R\left( k\right)$) found is stored as the frontal slice (sub-tensor) of the CCI-tensor $\mathcal {\chi } \in \mathbb {R}^{J \times J \times K}$:

3 $$\begin{aligned} \mathcal {\chi }_{::k} = \varvec{x}_{L\left( k\right) } \circ \varvec{x}_{R\left( k\right) } \end{aligned}$$

#### Non-negative Tucker3 decomposition (NTD-3)

To extract the CaHs from the CCI-tensor $\mathcal {\chi } \in \mathbb {R}^{J \times J \times K}$, we utilize NTD-3 and NTD-2, which are generalizations of non-negative matrix factorization (NMF) to tensor data [59, 60]. The NMF approximates a non-negative matrix data as the product of two lower rank non-negative matrices (also known as factor matrices). Similar to NMF, NTD-3 and NTD-2 approximate a non-negative tensor data as the product of some factor matrices and a core tensor.

To extend NMF to NTD-3, we consider iterative updating $\varvec{A}^{(n)} \mathcal {G}_{(n)} \varvec{A}^{(-n)T} = \varvec{A}^{(n)} \mathcal {G}_{A}^{(n)} \left( n = 1,2,3 \right)$, which is the matricized expression of tensor decomposition. Here $\varvec{A}^{(-n)}$ is Kronecker product of the factor matrices without $\varvec{A}^{(n)}$ and $\mathcal {G}_{(n)}$ is the mode-*n* matricization of the core tensor $\mathcal {G}$. For example, if $n=1$, these become $\varvec{A}^{(2)} \otimes \varvec{A}^{(3)}$ and $\mathcal {G}_{(n)}$, respectively. By replacing *X* in the multiplicative update rule [60] with $\varvec{A}^{(n)} \mathcal {G}_{(n)} \varvec{A}^{(-n)T} \left( n = 1,2,3 \right)$, we can obtain the update rule for $\varvec{A}^{(n)}$ as follows;

4 $$\begin{aligned} \begin{aligned} \varvec{A}^{(1)}&\leftarrow \varvec{A}^{(1)}\ *\frac{ \varvec{X}_{(1)} \mathcal {G}_{A}^{(1)T}}{\varvec{A}^{(1)} \mathcal {G}_{A}^{(1)} \mathcal {G}_{A}^{(1)T}} \\ \varvec{A}^{(2)}&\leftarrow \varvec{A}^{(2)}\ *\frac{ \varvec{X}_{(2)} \mathcal {G}_{A}^{(2)T}}{\varvec{A}^{(2)} \mathcal {G}_{A}^{(2)} \mathcal {G}_{A}^{(2)T}} \\ \varvec{A}^{(3)}&\leftarrow \varvec{A}^{(3)}\ *\frac{ \varvec{X}_{(3)} \mathcal {G}_{A}^{(3)T}}{\varvec{A}^{(3)} \mathcal {G}_{A}^{(3)} \mathcal {G}_{A}^{(3)T}}. \end{aligned} \end{aligned}$$

Similarly, the updating rule for core tensor $\mathcal {G}$ is:

5 $$\begin{aligned} \begin{aligned} \mathcal {G}&\leftarrow \max \{\mathcal {\chi } \times _{1} \varvec{A}^{(1)T} \times _{2} \varvec{A}^{(2)T} \times _{3} \varvec{A}^{(3)T}, \epsilon \} \\ \mathcal {G}&\leftarrow \frac{\mathcal {\chi } \times _{1} \varvec{A}^{(1)T} \times _{2} \varvec{A}^{(2)T} \times _{3} \varvec{A}^{(3)T}}{\mathcal {G} \times _{1} \varvec{A}^{(1)T} \varvec{A}^{(1)} \times _{2} \varvec{A}^{(2)T} \varvec{A}^{(2)} \times _{3} \varvec{A}^{(3)T} \varvec{A}^{(3)}}, \end{aligned} \end{aligned}$$

where $\epsilon$ is a small value included to avoid generating negative values (default value 1E−10).

#### Non-negative Tucker2 decomposition (NTD-2)

The NTD-3 has three rank parameters to be estimated, and it requires huge search space ($R1 \times R2 \times R3$). Additionally, the fewer the factor matrices, the more interpretable the results are. For these reasons, we further expanded the NTD-3 into a model called the NTD-2 [60] since v1.4.0 of scTensor.

In NTD-2, the third factor matrix $\varvec{A}^{(3)}$, which is related to L–R pairs, is replaced by an identity matrix $I_{K}$, where the *K* diagonal elements are all 1 and the iteration step of $\varvec{A}^{(3)}$ is skipped as follows:

6 $$\begin{aligned} \begin{aligned} \varvec{A}^{(1)}&\leftarrow \varvec{A}^{(1)}\ *\frac{ \varvec{X}_{(1)} \mathcal {G}_{A}^{(1)T}}{\varvec{A}^{(1)} \mathcal {G}_{A}^{(1)} \mathcal {G}_{A}^{(1)T}} \\ \varvec{A}^{(2)}&\leftarrow \varvec{A}^{(2)}\ *\frac{ \varvec{X}_{(2)} \mathcal {G}_{A}^{(2)T}}{\varvec{A}^{(2)} \mathcal {G}_{A}^{(2)} \mathcal {G}_{A}^{(2)T}}. \end{aligned} \end{aligned}$$

Here, $$\mathcal {G}_{A}^{(1)} = \mathcal {G}_{1} [\varvec{A}^{(2)} I_{K}]$$ and $$\mathcal {G}_{A}^{(2)} = \mathcal {G}_{2} [\varvec{A}^{(1)} I_{K}]$$.

The updating rule for core tensor $\mathcal {G}$ is

7 $$\begin{aligned} \begin{aligned} \mathcal {G}&\leftarrow \max \{\mathcal {\chi } \times _{1} \varvec{A}^{(1)T} \times _{2} \varvec{A}^{(2)T}, \epsilon \} \\ \mathcal {G}&\leftarrow \frac{\mathcal {\chi } \times _{1} \varvec{A}^{(1)T} \times _{2} \varvec{A}^{(2)T}}{\mathcal {G} \times _{1} \varvec{A}^{(1)T} \varvec{A}^{(1)} \times _{2} \varvec{A}^{(2)T} \varvec{A}^{(2)}}. \end{aligned} \end{aligned}$$

#### Rank estimation of NTD-2

To extract the CaHs, scTensor estimates the NTD-2 ranks for each matricized CCI-tensor ($X^{(n)}$, $n=1$ or 2). To be able to focus only on the dimensions that are informative and are not noisy, we used an ad hoc approach for NTD-2 rank estimation.

Because NMF is performed in each matricized CCI-tensor in scTensor, we estimated each rank of NMF based on the residual sum of squares (RSS) [122] as

8 $$\begin{aligned} \frac{\textrm{RSS}_{\textrm{max}} - \textrm{RSS}_{k}}{\textrm{RSS}_{\textrm{max}} - \textrm{RSS}_{\textrm{min}}} > \textrm{thr}_{\textrm{rank}}, \end{aligned}$$

where $\textrm{RSS}_{\textrm{max}}$ is the RSS by full rank NMF, $\textrm{RSS}_{\textrm{min}}$ is the RSS by rank-1 NMF, $\textrm{RSS}_{\textrm{k}}$ is the RSS by rank-*k* NMF, and $\textrm{thr}_{\textrm{rank}}$ is the threshold value, ranging 0 to 1 (the default value is 0.8). RSS by rank-*k* NMF is calculated between a data matrix *X* and the reconstructed matrix from *W* and *H* calculated by multiplicative updating rule [60] as follows:

9 $$\begin{aligned} \textrm{RSS}_{k} = \Vert X^{(n)} - W_{k} H_{k}\Vert _{F}^{2}. \end{aligned}$$

RSS by full-rank and rank-1 NMF is calculated by setting *k* as *J* and 1, respectively. With the estimated ranks $(\hat{R1}, \hat{R2})$, NTD-2 was performed, and only the pairs (r1,r2) with large core tensor values are selected as CaHs. In its default mode, scTensor selects CaHs that explain the top 20 pairs sorted by the core tensor values.

#### Binarization

To binarize each column vector of the factor matrices obtained by NTD-2, median absolute deviation (MAD), which is the median version of standard deviation (SD), was applied. Because we are only interested in the outliers of the elements of each vector in the positive direction, not the negative one, we focused only on the elements that deviate from the median in the positive direction as follows:

10 $$\begin{aligned} x_{i} = {\left\{ \begin{array}{ll} 1 &{} \left( x_{i} \ge \textrm{median}\left( x \right) + \textrm{thr}_{\textrm{bin}} \times \textrm{MAD}\left( x \right) \right) \\ 0 &{} \left( otherwise \right) . \end{array}\right. } \end{aligned}$$

Here, $\textrm{MAD}\left( x\right)$ is $\textrm{median}\left( \Vert x - \textrm{median}\left( x\right) \Vert \right)$ and $\textrm{thr}_{\textrm{bin}}$ is the threshold value (the default value is 1.0).

### L–R scoring

Several methods have been proposed to score the degree of co-expression of a given L–R pair between two cell types, as described below.

#### Sum score

The gene expression of a ligand gene *l* can be averaged over cells belonging to the *s*th cell type within *J* cell types as follows:

11 $$\begin{aligned} x_{l}^{C_{s}} = \frac{1}{N_{C_{s}}}\sum _{c \in C_{s}}{x_{lc}}. \end{aligned}$$

Here, $C_{s} \in \left( C_{1}, C_{2}, \ldots , C_{J} \right)$ and $N_{C_{s}}$ is the number of cells belonging to cell type $C_{s}$.

Likewise, the gene expression of a receptor gene *r* is averaged over cells belonging to the *t*th cell type within *J* cell types as follows:

12 $$\begin{aligned} x_{r}^{C_{t}} = \frac{1}{N_{C_{t}}}\sum _{c \in C_{t}}{x_{rc}}. \end{aligned}$$

Here, $C_{t} \in \left( C_{1}, C_{2}, \ldots , C_{J} \right)$ and $N_{C_{t}}$ is the number of cells belonging to cell type $C_{t}$.

Using these values, the sum score is calculated as follows:

13 $$\begin{aligned} Score_{sum}^{l,C_{s},r,C_{t}} = x_{l}^{C_{s}} + x_{r}^{C_{t}}. \end{aligned}$$

For example, some methods such as CellPhoneDB [37], Giotto [61], CrossTalkR [62], and Squidpy [63], essentially use this type of scoring (Table 1 and Additional file 1).

#### Product score

In some studies, the degree of co-expression is expressed as a product instead of a summation.

14 $$\begin{aligned} Score_{prod}^{l,C_{s},r,C_{t}} = x_{l}^{C_{s}} \times x_{r}^{C_{t}} \end{aligned}$$

For example, some methods such as NATME [64], FunRes [65], ICELLNET [66], and TraSig [67], essentially use this type of scoring (Table 1 and Additional file 1).

#### **Halpern**’s score

Derived from the sum score, Halpern et al. proposed a score described below (Table 1 and Additional files 1).

In this score, *Z*-scaling is firstly applied to both $x_{l}^{C_{s}}$ and $x_{r}^{C_{t}}$ over *J* cell types as follows:

15 $$\begin{aligned} Z_{l}^{C_{s}}= & {} \frac{x_{l}^{C_{s}} - \textrm{mean}\left( x_{L} \right) }{\textrm{std}\left( x_{L} \right) } \end{aligned}$$

16 $$\begin{aligned} Z_{r}^{C_{t}}= & {} \frac{x_{r}^{C_{t}} - \textrm{mean}\left( x_{R} \right) }{\textrm{std}\left( x_{R} \right) }. \end{aligned}$$

Here, $x_{L} = \left( x_{l}^{C_{1}}, x_{l}^{C_{2}}, \ldots , x_{l}^{C_{J}} \right)$ and $x_{R} = \left( x_{r}^{C_{1}}, x_{r}^{C_{2}}, \ldots , x_{r}^{C_{J}} \right)$. Then, the square root of the sum of squares of these values is used as the degree of co-expression as follows:

17 $$\begin{aligned} Score_{\texttt {Halpern}{}}^{l,C_{s},r,C_{t}} = \sqrt{\left( Z_{l}^{C_{s}} \right) ^2 + \left( Z_{r}^{C_{t}} \right) ^2}. \end{aligned}$$

#### **Cabello**$\mathrm {-}$**Aguilar**’s score

Derived from the product score, Cabello $\mathrm {-}$ Aguilar et al. proposed a score described as follows (Table 1 and Additional file 1):

18 $$\begin{aligned} Score_{{\texttt {Cabello}\mathrm {-}{} \texttt {Aguilar}}{}}^{l,C_{s},r,C_{t}} = \frac{\sqrt{Score_{prod}}}{\mu + \sqrt{Score_{prod}}}. \end{aligned}$$

Here, $\mu$ is the averaged value of the normalized read count matrix and is added as a scaling factor to avoid division by zero. This score is used in SingleCellSignalR [69] and CellTalkDB [70].

### Label permutation method

To quantify the deviation of the observed scores obtained from real data, many studies employ $P$ values in a statistical hypothesis testing framework. Typically, the label permutation method is widely used to calculate $P$ values. In principle, this method can be used in combination with any L–R score as described above.

Here, we consider assigning a $P$ value to any type of $Score^{l,C_{s},r,C_{t}}$ above. In this method, the cluster labels of all the cells are randomly shuffled, and a synthetic score value is calculated. Performing this process 1000 times generates 1000 of the values. These values are used to generate the null distribution; for a combination of cell types, the proportion of the means which are “as or more extreme” than the observed mean is calculated as the $P$ value. The label permutation test is performed as a one-tailed test; there is a focus on L–R scores with significantly higher values compared to the null distribution, and not on L–R scores with significantly lower values. Because separating significant CCIs from non-significant CCIs by hypothesis testing can be regarded as a binarization process, label permutation results were compared with the results of scTensor binarization.

### Quantitative evaluation of CCIs

The CCIs detected by the various methods tested in this paper were compared with ground truth CCIs to quantitatively evaluate the performance of each method. To evaluate the results, we used the metrics below.

#### Evaluation of the scoring before and after binarization

Each CCI method uses each corresponding L–R score to quantify the degree of co-expression of a given L–R pair between two cell types. To quantitatively evaluate the performance of each score, area under the curve of receiver operating characteristic (AUCROC) and area under the curve of precision–recall (AUCPR) were used.

A receiver operating characteristic (ROC) curve is a plot of the true positive rate (TPR, or the sensitivity = $\frac{TP}{TP + FN}$) versus the false positive rate (FPR, or 1 - specificity, where specificity = $\frac{TN}{TN + FP}$) (where TP is the number of true positive CCIs, FP is the number of false positive CCIs, TN is the number of true negative CCIs, and FN is the number of false negative CCIs). The AUCROC value is the area under the ROC curve. AUCROC values range from 0 to 1, and the closer the value is to 1, the more the score indicates enrichment of the ground truch CCIs among the inferred CCIs.

A precision–recall curve is a plot of recall (i.e., sensitivity) versus precision (i.e., positive predictive value = $\frac{TP}{TP + FP}$). The AUCPR value is the value of the area under the precision–recall curve. AUCPR ranges from 0 to 1, and the closer the value is to 1, the more the score indicates enrichment of the ground truch CCIs among the inferred CCIs. AUCPR is known for its robustness against class imbalance, compared with AUCROC [123–125]. Hence, it seems that AUCPR is more appropriate than AUCROC because the number of significant CCIs are assumed to be less than that of non-significant CCIs in both simulated and real empirical data.

To evaluate whether the binarization was properly performed, we also applied these metrics to assess label permutation. As the label permutation test outputs $P$ values, we utilized 1 − $P$ value to quantify the degree of co-expression of elements of L–R pairs in the test. Because tensor decomposition is an unsupervised learning methods, we cannot distinguish which CaHs are enriched within the ground truth CCIs in advance. Additionally, we expected that scTensor could separate different styles of CCIs as multiple CaHs. Hence, we used the combination of CaHs from scTensor and ground truth CCIs with the maximum metrics values.

The calculation time and memory usage were evaluated by using the benchmark rules of Snakemake (https://snakemake.readthedocs.io/en/latest/snakefiles/rules.html?highlight=benchmark#benchmark-rules).

#### Evaluation of the scoring after binarization

Each CCI method uses a threshold value (e.g., $P$ value, or MAD for scTensor) to differentiate significant CCIs from non-significant CCIs. This process is considered a kind of binarization (1 for significant CCIs, 0 for non-significant CCIs), so we evaluated how well each thresholding strategy could selectively detect the ground truch CCI by comparing the metrics below.

**Table 1:** Correspondence between L–R scoring used in this study and previous tools

L–R scoring used in this study	Previous tools
Sum score	CellPhoneDB [37], Giotto [61], CrossTalkR [62], and Squidpy [63]
Product score	NATME [64], FunRes [65], ICELLNET [66], and TraSig [67]
Halpern’s score	Halpern et al.
Cabello $\mathrm {-}$ Aguilar’s score	SingleCellSignalR [69] and CellTalkDB [70]

**Table 2:** Empirical datasets subjected to cell–cell interaction (CCI) identification

Name	Organisms	GEO ID	# Genes	# Cells	# Cell types	CCI-tensor size
FetalKidney [36]	*Homo sapiens*	GSE109205	3204	4131	11	11 $\times$ 11 $\times$ 1072
GermlineFemale [25]	*Homo sapiens*	GSE86146	2717	992	8	8 $\times$ 8 $\times$ 1622
HeadandNeckCancer [54]	*Homo sapiens*	GSE103322	5244	5577	26	26 $\times$ 26 $\times$ 61
Uterus [33]	*Mus musculus*	GSE118180	2566	6443	12	12 $\times$ 12 $\times$ 2282
VisualCortex [48]	*Mus musculus*	GSE102827	5365	47,209	30	30 $\times$ 30 $\times$ 274

**Table 3:** Many-to-many cell–cell interactions (CCIs) detected by only scTensor

Dataset	Signal pathway	Ligand and receptor	Reported
FetalKidney [36]	Eph/ephrin	EFNB2–EPHB3	Yes
FetalKidney [36]	Eph/ephrin	EFNB2–EPHB4	No
FetalKidney [36]	Eph/ephrin	EFNB2–EPHB6	No
FetalKidney [36]	Eph/ephrin	EFNA1–EPHA4/7	No
FetalKidney [36]	Eph/ephrin	EFNA5–EPHA4/7	No
GermlineFemale [25]	Bone morphogenetic protein (BMP)	BMP2–BMPR2	Yes
GermlineFemale [25]	Bone morphogenetic protein (BMP)	GDF9–BMPR2	No
GermlineFemale [25]	NOTCH	JAG1–NOTCH2	Yes
GermlineFemale [25]	NOTCH	JAG1–NOTCH3	No
HeadandNeckCancer [54]	Fibroblast growth factor (FGF)	FGF7–FGFR2	Yes
Uterus [33]	Insulin-like growth factor (IGF)	Igf1–Igf1r	Yes
Uterus [33]	Insulin-like growth factor (IGF)	Igf2–Igf1r	No
Uterus [33]	Pleiotrophin (PTN)	Ptn–Ptprb	Yes
Uterus [33]	Wingless-related integration site (Wnt)	Rspo3–Lgr5	Yes
Uterus [33]	Vascular endothelial growth factor (VEGF)	Vegfa–Kdr	Yes
Uterus [33]	Vascular endothelial growth factor (VEGF)	Vegfa–Flt1/Nrp1	No
VisualCortex [48]	Activin receptor (ACVR)	Inhba–Acvr1b	Yes
VisualCortex [48]	Activin receptor (ACVR)	Nodal–Acvr1b	No
VisualCortex [48]	Activin receptor (ACVR)	Nodal/Bmp5/Bmp7–Acvr2a	No

**Table 4:** Order of calculation time and memory space for cell–cell interaction (CCI) identification

Method	Calculation time	Memory space
Sum/product score	$\mathcal {O}(N^{2}L)$	$\mathcal {O}(N^{2}L)$
Permutation test of sum/product score	$\mathcal {O}(N^{2}LP)$	$\mathcal {O}(N^{2}L)$
Halpern’s score	$\mathcal {O}(N^{2}L)$	$\mathcal {O}(N^{2}L)$
Permutation test of Halpern’s score	$\mathcal {O}(N^{2}LP)$	$\mathcal {O}(N^{2}L)$
Cabello $\mathrm {-}$ Aguilar’s score	$\mathcal {O}(N^{2}L)$	$\mathcal {O}(N^{2}L)$
Permutation test of Cabello $\mathrm {-}$ Aguilar’s score	$\mathcal {O}(N^{2}LP)$	$\mathcal {O}(N^{2}L)$
Previous scTensor (NTD-3)	$\mathcal {O}(N^{2}L(R1+R2+R3))$	$\mathcal {O}(N^{2}L)$
scTensor (NTD-2)	$\mathcal {O}(N^{2}L(R1+R2))$	$\mathcal {O}(N^{2}L)$

F-measure is the harmonic mean of precision and recall and is defined as follows:

19 $$\begin{aligned} F\mathrm {-}measure = \frac{2\ \textrm{precision} \times \textrm{recall}}{\textrm{precision} + \textrm{recall}}. \end{aligned}$$

Matthews Correlation Coefficient (MCC) is a special case of Pearson correlation coefficient when two variables are both binary vectors. MCC is defined as follows:

20 $$\begin{aligned} MCC = \frac{\textrm{TP} \times \textrm{TN} - \textrm{FP} \times \textrm{FN}}{\sqrt{ \left( \textrm{TP} + \textrm{FP} \right) \left( \textrm{TP} + \textrm{FN} \right) \left( \textrm{TN} + \textrm{FP} \right) \left( \textrm{TN} + \textrm{FN} \right) }}. \end{aligned}$$

MCC is widely used for binary classification evaluation and especially known for its robustness against the class imbalance, compared with the other metrics such as accuracy, balanced accuracy, bookmaker informedness, markedness, and F-measure [126–128]. Hence, it seems that MCC is more appropriate to use than F-measure because the number of significant CCIs are assumed to be less than that of non-significant CCIs in both simulated and real empirical data.

To distinguish whether the F-measure and MCC values correspond to the number of detected CCIs or their selectivity in focusing only the ground truth CCIs, we also compared the positive rate (PR), false positive rate (FPR), and false negative rate (FNR) values of all the methods.

### Availability and requirements

#### R packages

- scTensor: https://bioconductor.org/packages/devel/bioc/html/scTensor.html
- nnTensor: https://cran.r-project.org/web/packages/nnTensor/index.html
- AnnotationHub: https://bioconductor.org/packages/release/bioc/html/AnnotationHub.html
- LRBaseDbi: https://bioconductor.org/packages/release/bioc/html/LRBaseDbi.html
- Operating system: Linux, Mac OS X, Windows
- Programming language: R (v$-$4.1.0 or higher), Bioconductor version (v$-$3.14.0 or higher)
- License: Artistic$$-$$2.0
- Any restrictions to use by non-academics: For non-profit use only

#### Snakemake workflows

- scTensor-experiments (for the analyses conducted in this study): https://github.com/rikenbit/scTensor-experiments
- lrbase-workflow (for the bi-annual updates of LRBase): https://github.com/rikenbit/lrbase-workflow
- Operating system: Linux, Mac OS X, Windows
- Programming language: Python (v$-$3.7.8 or higher), Snakemake (v$-$6.0.5 or higher), Singularity (v$-$3.8.0 or higher)
- License: MIT
- Any restrictions to use by non-academics: For non-profit use only

### Supplementary Information

**Additional file 1**. List of existing L–R scoring and CCI detection methods.

**Additional file 2**. Ground truth CCIs in simulated datasets.

**Additional file 3**. AUCROC values of all methods.

**Additional file 4**. AUCPR values of all methods.

**Additional file 5**. Memory values of all methods.

**Additional file 6**. Computational time values of all methods.

**Additional file 7**. F-measure values of all binarization methods.

**Additional file 8**. MCC values of all binarization methods.

**Additional file 9**. PR values of all binarization methods.

**Additional file 10**. FPR values of all binarization methods.

**Additional file 11**. FNR values of all binarization methods.

**Additional file 12**. TR of all datasets.

**Additional file 13**. Three L-R pairs in which each of the three methods excelled.

**Additional file 14**. HTML report of FetalKidney.

**Additional file 15**. HTML report of GermlineFemale.

**Additional file 16**. HTML report of HeadandNeckCancer.

**Additional file 17**. HTML report of Uterus.

**Additional file 18**. HTML report of VisualCortex.

## Abbreviations

CCIs: Cell–cell interactions
scRNA-seq: Single-cell RNA sequencing
L–R: Ligand and receptor
CaH: CCI as hypergraph
CMPED: Count per median of library size
NTD-3: Non-negative Tucker3 decomposition
NTD-2: Non-negative Tucker2 decomposition
AUCPR: Area under the curve of precision–recall
FPR: False positive rate
FNR: False negative rate
MCC: Matthews correlation coefficient
FP: False positive
FN: False negative
GO: Gene ontology
MeSH: Medical subject headings
ORA: Over-representative analysis
DO: Disease ontology
NCG: Network of cancer genes
CMap: Connectivity map
FC: Fold-change
mESCs: Mouse embryonic stem cells
DEGs: Differentially expressed genes
HVGs: Highly variable genes
NMF: Non-negative matrix factorization
RSS: Residual sum of squares
MAD: Median absolute deviation
SD: Standard deviation
AUCROC: Area under the curve
TPR: True positive rate
TP: True positive
TN: True negative
PR: Positive rate

## References

[1] Yu Y, Elble RC (2016). Homeostatic signaling by cell-cell junctions and its dysregulation during cancer progression. J Clin Med.

[2] Livshits G, Kobielak A, Fuchs E (2012). Governing epidermal homeostasis by coupling cell–cell adhesion to integrin and growth factor signaling, proliferation, and apoptosis. PNAS.

[3] Chao DL, Ma L, Shen K (2009). Transient cell–cell interactions in neural circuit formation. Nat Rev Neurosci.

[4] Kasukawa T, Masumoto K, Nikaido I, Nagano M, Uno KD, Tsujino K, Hanashima C, Shigeyoshi Y, Ueda HR (2011). Quantitative expression profile of distinct functional regions in the adult mouse brain. PLoS ONE.

[5] Miller JFAP, Mitchell GF (1970). Cell to cell interaction in the immune response v. target cells for tolerance induction. J Exp Med.

[6] Pieters T, Roy VF (2014). Role of cell–cell adhesion complexes in embryonic stem cell biology. J Cell Sci.

[7] Tweedell KS (2017). The adaptability of somatic stem cells: a review. J Stem Cells Regen Med.

[8] Plaks V, Kong N, Werb Z (2015). The cancer stem cell niche: how essential is the niche in regulating stemness of tumor cells?. Cell Stem Cell.

[9] Hegerfeldt Y, Tusch M, Brocker EB, Friedl P (2002). Collective cell movement in primary melanoma explants: plasticity of cell-cell interaction, beta1-integrin function, and migration strategies. Cancer Res.

[10] Hofschroer V, Koch KA, Ludwig FT, Friedl P, Oberleithner H, Stock C, Schwab A (2017). Extracellular protonation modulates cell–cell interaction mechanics and tissue invasion in human melanoma cells. Sci Rep.

[11] Stein JV, Gonzalez SF (2016). Dynamic intravital imaging of cell–cell interactions in the lymph node. Mech Allerg Dis.

[12] Reinhar-King CA, Dembo M, Hammer DA (2008). Cell–cell mechanical communication through compliant substrates. Biophys J.

[13] Dewji NN, Mukhopadhyay D, Singer SJ (2006). An early specific cell–cell interaction occurs in the production of beta-amyloid in cell cultures. PNAS.

[14] Konry T, Sarkar S, Sabhachandani P, Cohen N (2016). Innovative tools and technology for analysis of single cells and cell–cell interaction. Annu Rev Biomed Eng.

[15] Rothbauer M, Zirath H, Ertl P (2018). Recent advances in microfluidic technologies for cell-to-cell interaction studies. Lab Chip.

[16] Li R, Lv X, Zhang X, Saeed O, Deng Y (2016). Microfluidics for cell–cell interactions: a review. Front Chem Sci Eng.

[17] Wiklund M, Christakou AE, Ohlin M, Iranmanesh I, Frisk T, Vanherberghen V, Onfelt B (2014). Ultrasound-induced cell–cell interaction studies in a multi-well microplate. Micromachines.

[18] Tauriainen J, Gustafsson K, Gothlin M, Gertow J, Buggert M, Frisk TW, Karlsson AC, Uhlin M, Onfelt B (2015). Single-cell characterization of in vitro migration and interaction dynamics of t cells expanded with il-2 and il-7. Front Immunol.

[19] Merouane A, Rey-Villamizar N, Lu Y, Liadi I, Romain G, Lu J, Singh H, Cooper LJN, Varadarajan N, Roysam B (2015). Automated profiling of individual cell–cell interactions from high-throughput time-lapse imaging microscopy in nanowell grids (timing). Bioinformatics.

[20] Espulgar W, Yamaguchi Y, Aoki W, Mita D, Saito M, Lee JK, Tamiya E (2015). Single cell trapping and cell–cell interaction monitoring of cardiomyocytes in a designed microfluidic chip. Sens Actuators B Chem.

[21] Sarkar S, Sabhachandani P, Stroopinsky D, Palmer K, Cohen N, Rosenblatt J, Avigan D, Konry T (2016). Dynamic analysis of immune and cancer cell interactions at single cell level in microfluidic droplets. Biomicrofluidics.

[22] Dura B, Dougan SK, Barisa M, Hoehl MM, Lo CT, Ploegh HL, Voldman J (2015). Profiling lymphocyte interactions at the single-cell level by microfluidic cell pairing. Nat Commun.

[23] Ramilowski JA, Goldberg T, Harshbarger J, Kloppmann E, Lizio M, Satagopam VP, Itoh M, Kawaji H, Carninci P, Rost B, Forrest ARR (2015). A draft network of ligand-receptor-mediated multicellular signalling in human. Nat Commun.

[24] Camp JG, Sekin K, Gerber T, Loeffler-Wirth H, Binder H, Gac M, Kanton S, Kageyama J, Damm G, Seehofer D, Belicova L, Bickle M, Barsacchi R, Okuda R, Yoshizawa E, Kimura M, Ayabe H, Taniguchi H, Takebe T, Treutlein B (2017). Multilineage communication regulates human liver bud development from pluripotency. Nature.

[25] Li L, Dong J, Yan L, Yong J, Liu X, Hu Y, Fan X, Wu X, Guo H, Wang X, Zhu X, Li R, Yan J, Wei Y, Zhao Y, Wang W, Ren Y, Yuan P, Yan Z, Hu B, Guo F, Wen L, Tang F, Qiao J (2017). Single-cell RNA-seq analysis maps development of human germline cells and gonadal niche interactions. Cell Stem Cell.

[26] Zhou JX, Taramelli R, Pedrini E, Knijnenburg T, Hunag S (2017). Extracting intercellular signaling network of cancer tissues using ligand-receptor expression patterns from whole-tumor and single-cell transcriptomes. Sci Rep.

[27] Skelly DA, Squiers GT, McLellan MA, Bolisetty MT, Robson P, Rosenthal NA, Pinto AR (2018). Single-cell transcriptional profiling reveals cellular diversity and intercommunication in the mouse heart. Cell Rep.

[28] Pavlicev M, Wagner GP, Chavan AR, Owens K, Maziarz J, Dunn-Fletcher C, Lallapur SG, Muglia L, Jones H (2017). Single-cell transcriptomics of the human placenta: inferring the cell communication network of the maternal-fetal interface. Genome Res.

[29] Joost S, Jacob T, Sun X, Annusver K, La Manno G, Sur I, Kasper M (2018). Single-cell transcriptomics of traced epidermal and hair follicle stem cells reveals rapid adaptations during wound healing. Cell Rep.

[30] Kramann R, Machado F, Wu H, Kusaba T, Hoeft K, Schneider RK, Humphreys BD (2018). Parabiosis and single-cell RNA sequencing reveal a limited contribution of monocytes to myofibroblasts in kidney fibrosis. JCI Insight.

[31] Cohen M, Giladi A, Gorki AD, Solodkin DG, Zada M, Hladik A, Miklosi A, Salame TM, Halpern KB, David E, Itzkovitz S, Harkany T, Knapp S, Amit I (2018). Lung single-cell signaling interaction map reveals basophil role in macrophage imprinting. Cell.

[32] Davidson S, Efremova M, Riedel A, Mahata B, Pramanik J, Huuhtanen J, Kar G, Vento-Tormo R, Hagai T, Chen X, Haniffa MA, Shields JD, Teichmann SA (2018). Single-cell RNA sequencing reveals a dynamic stromal niche within the evolving tumour microenvironment. bioRxiv.

[33] Mucenski ML, Mahoney R, Adam M, Potter AS, Potter SS (2019). Single cell RNA-seq study of wild type and hox9,10,11 mutant developing uterus. Sci Rep.

[34] Wu H, Uchimura K, Donnelly EL, Kirita Y, Morris SA, Humphreys BD (2018). Comparative analysis and refinement of human PSC-derived kidney organoid differentiation with single-cell transcriptomics. Cell Stem Cell.

[35] Chen L, Lee JW, Chou CL, Nair AV, Battistone MA, Paunescu TG, Merkulova M, Breton S, Verlander JW, Wall SM, Brown D, Burg MB, Knepper MA (2017). Transcriptomes of major renal collecting duct cell types in mouse identified by single-cell RNA-seq. PNAS.

[36] Menon R, Otto EA, Kokoruda A, Zhou J, Zhang Z, Yoon E, Chen YC, Troyanskaya O, Spence JR, Kretzler M, Cebrian C (2018). Single-cell analysis of progenitor cell dynamics and lineage specification in the human fetal kidney. Development.

[37] Vento-Tormo R, Efremova M, Botting RA, Turco MY, Vento-Tormo M, Meyer KB, Park JE, Stephenson E, Polanski K, Goncalves A, Gardner L, Holmqvist S, Henriksson J, Zou A, Sharkey AM, Millar B, Innes B, Wood L, Wilbrey-Clark A, Payne RP, Ivarsson MA, Lisgo S, Filby A, Rowitch DH, Bulmer JN, Wright GJ, Stubbington MJT, Haniffa M, Moffett A, Teichmann SA (2018). Single-cell reconstruction of the early maternal-fetal interface in humans. Nature.

[38] Biton M, Haber AL, Rogel N, Burgin G, Beyaz S, Schnell A, Ashenberg O, Su CW, Smillie C, Shekhar K, Chen Z, Wu C, Ordovas-Montanes J, Alvarez D, Herbst RH, Zhang M, Tirosh I, Dionne D, Nguyen LT, Xifaras ME, Shalek AK, von Andrian UH, Graham DB, Rozenblatt-Rosen O, Shi HN, Kuchroo V, Yilmaz OH, Regev A, Xavier RJ (2018). T helper cell cytokines modulate intestinal stem cell renewal and differentiation. Cell.

[39] Kumar MP, Du J, Lagoudas G, Jiao Y, Sawyer A, Drummond DC, Lauffenburger DA, Raue A (2018). Analysis of single-cell RNA-seq identifies cell–cell communication associated with tumor characteristics. Cell Rep.

[40] Verma M, Asakura Y, Murakonda BSR, Pengo T, Latroche C, Chazaud B, McLoon LK, Asakura A (2018). Muscle satellite cell cross-talk with a vascular niche maintains quiescence via VEGF and notch signaling. Cell Stem Cell.

[41] Jerby-Arnon L, Shah P, Cuoco MS, Rodman C, Su MJ, Melms JC, Leeson R, Kanodia A, Mei S, Lin JR, Wang S, Rabasha B, Liu D, Zhang G, Margolais C, Ashenberg O, Ott PA, Buchbinder EI, Haq R, Hodi FS, Boland GM, Sullivan RJ, Frederick DT, Miao B, Moll T, Flaherty KT, Herlyn M, Jenkins RW, Thummalapalli R, Kowalczyk MS, Canadas I, Schilling B, Cartwright ANR, Luoma AM, Malu S, Hwu P, Bernatchez C, Forget MA, Barbie DA, Shalek AK, Tirosh I, Sorger PK, Wucherpfennig K, Van Allen EM, Schadendorf D, Johnson BE, Rotem A, Rozenblatt-Rosen O, Garraway LA, Yoon CH, Izar B, Regev A (2018). A cancer cell program promotes T cell exclusion and resistance to checkpoint blockade. Cell.

[42] Kelleher AM, Milano-Foster J, Behura SK, Spencer TE (2018). Uterine glands coordinate on-time embryo implantation and impact endometrial decidualization for pregnancy success. Nat Commun.

[43] Yin J, Li Z, Yan C, Fang E, Wang T, Zhou H, Luo W, Zhou Q, Zhang J, Hu J, Jin H, Wang L, Zhao X, Li J, Qi X, Zhou W, Huang C, He C, Yang H, Kristiansen K, Hou Y, Zhu S, Zhou D, Wang L, Dean M, Wu K, Hu H, Li G (2018). Comprehensive analysis of immune evasion in breast cancer by single-cell RNA-seq. bioRxiv.

[44] Biase FH, Kimble KM (2018). Functional signaling and gene regulatory networks between the oocyte and the surrounding cumulus cells. BMC Genom.

[45] Thorsson V, Gibbs DL, Brown SD, Wolf D, Bortone DS, Ou Yang TH, Porta-Pardo E, Gao GF, Plaisier CL, Eddy JA, Ziv E, Culhane AC, Paull EO, Sivakumar IKA, Gentles AJ, Malhotra R, Farshidfar F, Colaprico A, Parker JS, Mose LE, Vo NS, Liu J, Liu Y, Rader J, Dhankani V, Reynolds SM, Bowlby R, Califano A, Cherniack AD, Anastassiou D, Bedognetti D, Rao A, Chen K, Krasnitz A, Hu H, Malta TM, Noushmehr H, Pedamallu CS, Bullman S, Ojesina AI, Lamb A, Zhou W, Shen H, Choueiri TK, Weinstein JN, Guinney J, Saltz J, Holt RA, Rabkin CE, Network CGAR, Lazar AJ, Serody JS, Demicco EG, Disis ML, Vincent BG, Shmulevich L (2018). The immune landscape of cancer. Immunity.

[46] Han X, Chen H, Huang D, Chen H, Fei L, Cheng C, Huang H, Yuan GC, Guo G (2018). Mapping human pluripotent stem cell differentiation pathways using high throughput single-cell RNA-sequencing open access. BMC Genome Biol.

[47] Costa A, Kieffer Y, Scholer-Dahirel A, Pelon F, Bourachot B, Cardon M, Sirven P, Magagna I, Fuhrmann L, Bernard C, Bonneau C, Kondratova M, Kuperstein I, Zinovyev A, Givel AM, Parrini MC, Soumelis V, Vincent-Salomon A, Mechta-Grigoriou F (2018). Fibroblast heterogeneity and immunosuppressive environment in human breast cancer. Cancer Cells.

[48] Hrvatin S, Hochbaum DR, Nagy MA, Cicconet M, Robertson K, Cheadle L, Zilionis R, Ratner A, Borges-Monroy R, Klein AM, Sabatini BL, Greenberg ME (2018). Single-cell analysis of experience-dependent transcriptomic states in the mouse visual cortex. Nat Neurosci.

[49] Suryawanshi H, Morozov P, Straus A, Sahasrabudhe N, Max KEA, Garzia A, Kustagi M, Tuschl T, Williams Z (2018). A single-cell survey of the human first-trimester placenta and decidua. Sci Adv.

[50] Puram SV, Tirosh I, Parikh AS, Patel AP, Yizhak K, Gillespie S, Rodman C, Luo CL, Mroz EA, Emerick KS, Deschler DG, Varvares MA, Mylvaganam R, Rozenblatt-Rosen O, Rocco JW, Faquin WC, Lin DT, Regev A, Bernstein BE (2017). Single-cell transcriptomic analysis of primary and metastatic tumor ecosystems in head and neck cancer. Cell.

[51] Ximerakis M, Lipnick SL, Simmons SK, Adiconis X, Innes BT, Dionne D, Nguyen L, Mayweather BA, Ozek C, Niziolek Z, Butty VL, Isserlin R, Buchanan SM, Levine SR, Regev A, Bader GD, Levin JZ, Rubin LL (2018). Single-cell transcriptomics of the aged mouse brain reveals convergent, divergent and unique aging signatures. bioRxiv.

[52] Sivakamasundari V, Bolisetty M, Sivajothi S, Bessonett S, Ruan D, Robson P (2017). Comprehensive cell type specific transcriptomics of the kidney. bioRxiv.

[53] Peters AL, Luo Z, Li J, Mourya R, Wang Y, Dexheimer P, Shivakumar P, Aronow B, Bezerra JA (2019). Single cell RNA sequencing reveals regional heterogeneity of hepatobiliary innate lymphoid cells in a tissue-enriched fashion. PLoS ONE.

[54] Puram SV, Tirosh I, Parikh AS, Patel AP, Yizhak K, Gillespie S, Rodman C, Luo CL, Mroz EA, Emerick KS, Deschler DG, Varvares MA, Mylvaganam R, Rozenblatt-Rosen O, Rocco JW, Faquin WC, Lin DT, Regev A, Bernstein BE (2017). Single-cell transcriptomic analysis of primary and metastatic tumor ecosystems in head and neck cancer. Cell.

[55] Boisset JC, Vivie J, Murano MJ, Lyubimova A, van Oudenaarden A (2018). Mapping the physical network of cellular interactions. Nat Methods.

[56] Zheng GX, Terry JM, Belgrader P, Ryvkin P, Bent ZW, Wilson R, Ziraldo SB, Wheeler TD, McDermott GP, Zhu J, Gregory MT, Shuga J, Montesclaros L, Underwood JG, Masquelier DA, Nishimura SY, Schnall-Levin M, Wyatt PW, Hindson CM, Bharadwaj R, Wong A, Ness KD, Beppu LW, Deeg HJ, McFarland C, Loeb KR, Valente WJ, Ericson NG, Stevens EA, Radich JP, Mikkelsen TS, Hindson BJ, Bielas JH (2017). Massively parallel digital transcriptional profiling of single cells. Nat Commun.

[57] van Dijk D, Sharma R, Nainys J, Yim K, Kathail P, Carr AJ, Burdziak C, Moon KR, Chaffer CL, Pattabiraman D, Bierie B, Mazutis L, Wolf G, Krishnaswamy S, Pe’er D (2018). Recovering gene interactions from single-cell data using data diffusion. Cell.

[58] Shekhar K, Lapan SW, Whitney IE, Tran NM, Macosko EZ, Kowalczyk M, Adiconis X, Levin JZ, Nemesh J, Goldman M, McCarroll SA, Cepko CL, Regev A, Sanes JR (2016). Comprehensive classification of retinal bipolar neurons by single-cell transcriptomics. Cell.

[59] Kim Y-D, Choi S. Nonnegative tucker decomposition. In: IEEE conference on computer vision and pattern recognition (2007)

[60] Cichocki A, Zdunek R, Phan AH, Amari S (2009). Nonnegative matrix and tensor factorizations: applications to exploratory multi-way data analysis and blind source separation.

[61] Dries R, Zhu Q, Dong R, Eng C-HL, Li H, Liu K, Fu Y, Zhao T, Sarkar A, Bao F, George RE, Pierson N, Cai L, Yuan G-C (2021). Giotto: a toolbox for integrative analysis and visualization of spatial expression data. BMC Genome Biol.

[62] Nagai JS, Leimkühler NB, Schaub MT, Schneider RK, Costa IG (2021). Crosstalker: analysis and visualization of ligand-receptor networks. Bioinformatics.

[63] Palla G, Spitzer H, Klein M, Fischer D, Schaar AC, Kuemmerle LB, Rybakov S, Ibarra IL, Holmberg O, Virshup I, Lotfollahi M, Richter S, Theis FJ (2021). Squidpy: a scalable framework for spatial single cell analysis. bioRxiv.

[64] Hou R, Denisenko E, Ong HT, Ramilowski JA, Forrest ARR (2020). Predicting cell-to-cell communication networks using NATMI. Nat Commun.

[65] Jung S, Singh K, Sol A (2021). Funres: resolving tissue-specific functional cell states based on a cell–cell communication network model. Brief Bioinform.

[66] Noël F, Massenet-Regad L, Carmi-Levy I, Cappuccio A, Grandclaudon M, Trichot C, Kieffer Y, Mechta-Grigoriou F, Soumelis V (2021). Dissection of intercellular communication using the transcriptome-based framework ICELLNET. Nat Commun.

[67] Li D, Velazquez JJ, Ding J, Hislop J, Ebrahimkhani MR, Bar-Joseph Z (2021). Inferring cell–cell interactions from pseudotime ordering of scRNA-Seq data. bioRxiv.

[68] Halpern KB, Shenhav R, Massalha H, Toth B, Egozi A, Massasa EE, Medgalia C, David E, Giladi A, Moor AE, Porat Z, Amit I, Itzkovitz S (2018). Paired-cell sequencing enables spatial gene expression mapping of liver endothelial cells. Nat Biotechnol.

[69] Cabello-Aguilar S, Alame M, Kon-Sun-Tack F, Fau C, Lacroix M, Colinge J (2020). Singlecellsignalr: inference of intercellular networks from single-cell transcriptomics. Nucleic Acids Res.

[70] Shao X, Liao J, Li C, Lu X, Cheng J, Fan X (2021). Celltalkdb: a manually curated database of ligand-receptor interactions in humans and mice. Brief Bioinform.

[71] Armingol E, Baghdassarian HM, Martino C, Perez-Lopez A, Knight R, Lewis NE (2022). Context-aware deconvolution of cell–cell communication with tensor-cell2cell. Nat Commun.

[72] Dimitrov D, Türei D, Garrido-Rodriguez M, Burmedi PL, Nagai JS, Boys C, Ramirez Flores RO, Kim H, Szalai B, Costa IG, Valdeolivas A, Dugourd A, Saez-Rodriguez J (2022). Comparison of methods and resources for cell–cell communication inference from single-cell RNA-seq data. Nat Commun.

[73] Baghdassarian H, Dimitrov D, Armingol E, Saez-Rodriguez J, Lewis NE (2023). Combining liana and tensor-cell2cell to decipher cell–cell communication across multiple samples. bioRxiv.

[74] Tsuyuzaki KT, Ishii M, Nikaido I (2023). nnTensor: an R package for non-negative matrix/tensor decomposition. JOSS.

[75] Durinck S, Spellman P, Birney E, Huber W (2009). Mapping identifiers for the integration of genomic datasets with the R/Bioconductor package biomaRT. Nat Protocols.

[76] Fabregat A, Jupe S, Matthews L, Sidiropoulos K, Gillespie M, Garapati P, Haw R, Jassal B, Korninger F, May B, Milacic M, Roca CD, Rothfels K, Sevilla C, Shamovsky V, Shorser S, Varusai T, Viteri G, Weiser J, Wu G, Stein L, Hermjakob H, D’Eustachio P (2018). The reactome pathway knowledgebase. Nucleic Acids Res.

[77] Tsuyuzaki K, Morota G, Ishii M, Nakazato T, Miyazaki S, Nikaido I (2015). MeSH ORA framework: R/Bioconductor packages to support MeSH over-representation analysis. BMC Bioinform.

[78] Falcon S, Gentleman R (2007). Using GOstats to test gene lists for GO term association. Bioinformatics.

[79] Yu G, He Q (2016). ReactomePA: an R/Bioconductor package for reactome pathway analysis and visualization. Mol BioSyst.

[80] Yu G, Wang L, Yan G, He Q (2015). DOSE: an R/Bioconductor package for disease ontology semantic and enrichment analysis. Bioinformatics.

[81] Ono H, Ogasawara O, Okubo K, Bono H (2017). Refex, a reference gene expression dataset as a web tool for the functional analysis of genes. Sci Data.

[82] Papatheodorou I, Fonseca NA, Keays M, Tang YA, Barrera E, Bazant W, Burke M, Fullgrabe A, Fuentes AM, George N, Huerta L, Koskinen S, Mohammed S, Geniza M, Preece J, Jaiswal P, Jarnuczak AF, Huber W, Stegle O, Vizcaino JA, Brazma A, Petryszak R (2018). Expression atlas: gene and protein expression across multiple studies and organisms. Nucleic Acids Res.

[83] Papatheodorou I, Moreno P, Manning J, Fuentes AMP, George N, Fexova S, Fonseca NA, Füllgrabe A, Green M, Huang N, Huerta L, Iqbal H, Jianu M, Mohammed S, Zhao L, Jarnuczak AF, Jupp S, Marioni J, Meyer K, Petryszak R, Medina CAP, Talavera-López C, Teichmann S, Vizcaino JA, Brazma A (2019). Expression atlas update: from tissues to single cells. Nucleic Acids Res.

[84] Cao Y, Zhu J, Jia P, Zhao Z (2017). scRNASeqDB: a database for RNA-Seq based gene expression profiles in human single cells. Genes (Basel).

[85] Franzén O, Gan L-M, Björkegren JLM. Panglaodb: a web server for exploration of mouse and human single-cell RNA sequencing data. Database 2019, 046 10.1093/database/baz046 PMC6450036 30951143

[86] Carithers LJ, Ardlie K, Barcus M, Branton PA, Britton A, Buia SA, Compton CC, DeLuca DS, Peter-Demchok J, Gelfand ET, Guan P, Korzeniewski GE, Lockhart NC, Rabiner CA, Rao AK, Robinson KL, Roche NV, Sawyer SJ, Segre AV, Shive CE, Smith AM, Sobin LH, Undale AH, Valentino KM, Vaught J, Young TR, Moore HM, Consortium G (2015). A novel approach to high-quality postmortem tissue procurement: the GTEx project. Biopreservation Biobanking.

[87] RIKEN: Collection | 29 August 2017 The FANTOM5 project. https://www.nature.com/collections/jcxddjndxy

[88] Bernstein BE, Stamatoyannopoulos JA, Costello JF, Ren B, Milosavljevic A, Meissner A, Kellis M, Marra MA, Beaudet AL, Ecker JR, Farnham PJ, Hirst M, Lander ES, Mikkelsen TS, Thomson JA (2010). The NIH roadmap epigenomics mapping consortium. Nat Biotechnol.

[89] Consotium EP (2012). An integrated encyclopedia of DNA elements in the human genome. Nature.

[90] Uhlen M, Fagerberg L, Hallstrom BM, Lindskog C, Oksvold P, Mardinoglu A, Sivertsson A, Kampf C, Sjostedt E, Asplund A, Olsson I, Edlund K, Lundberg E, Navani S, Szigyarto CA, Odeberg J, Djureinovic D, Takanen JO, Hober S, Alm T, Edqvist PH, Berling H, Tegel H, Mulder J, Rockberg J, Nilsson P, Schwenk JM, Hamsten M, von Feilitzen K, Forsberg M, Persson L, Johansson F, Zwahlen M, von Heijne G, Nielsen JFP (2015). Proteomics tissue-based map of the human proteome. Science.

[91] Lamb J, Crawford ED, Peck D, Modell JW, Blat IC, Wrobel MJ, Lerner J, Brunet JP, Subramanian A, Ross KN, Reich M, Hieronymus H, Wei G, Armstrong SA, Haggarty SJ, Clemons PA, Wei R, Carr SA, Lander ES, Golub TR (2006). The connectivity map: using gene-expression signatures to connect small molecules, genes, and disease. Science.

[92] Wang Y, Tung H-Y, Smola A, Anandkumar A. Fast and guaranteed tensor decomposition via sketching. In: NIPS, vol 1, pp 991–999 (2015)

[93] Maehara, T., Hayashi, K., Kawarabayashi, K.: Expected tensor decomposition with stochastic gradient descent. In: AAAI’16, pp 1919–1925 (2016)

[94] Smith, S., Park, J., Karypis, G.: An exploration of optimization algorithms for high performance tensor completion. In: SC ’16 proceedings of the international conference for high performance computing, networking, storage and analysis, vol 31 (2016)

[95] Shin K, Sael L, Kang U (2017). Fully scalable methods for distributed tensor factorization. IEEE Trans Knowl Data Eng.

[96] Tsuyuzaki K. Guidelines to handle large-scale and complex tensor data in r. BioC Asia 2021 (2021). 10.7490/f1000research.1118833.1

[97] DelayedArray: A Unified framework for working transparently with on-disk and in-memory array-like datasets. R Package Version 0.24.0. https://bioconductor.org/packages/DelayedArray

[98] Tsuyuzaki, K., Nikaido, I.: Biological systems as heterogeneous information networks: a mini-review and perspectives. HeteroNAM’18 (2018)

[99] Baccin C, Al-Sabah J, Velten L, Helbling PM, Grünschläger F, Hernández-Malmierca P, Nombela-Arrieta C, Steinmetz LM, Trumpp A, Haas S (2020). Combined single-cell and spatial transcriptomics reveal the molecular, cellular and spatial bone marrow niche organization. Nat Cell Biol.

[100] Lagger C, Ursu E, Equey A, Avelar RA, Pisco AO, Tacutu R, Magalhães JP (2021). scAgeCom: a murine atlas of age-related changes in intercellular communication inferred with the package scDiffCom. bioRxiv.

[101] Yuan Y, Cosme C, Adams TS, Schupp J, Sakamoto K, Xylourgidis N, Ruffalo M, Kaminski N, Bar-Joseph Z (2021). Cins: cell interaction network inference from single cell expression data. bioRxiv.

[102] Jin S, Guerrero-Juarez CF, Zhang L, Chang I, Ramos R, Kuan C-H, Myung P, Plikus MV, Nie Q (2021). Inference and analysis of cell–cell communication using cellchat. Nat Commun.

[103] Raredon MSB, Yang J, Garritano J, Wang M, Kushnir D, Schupp JC, Adams TS, Greaney AM, Leiby KL, Kaminski N, Kluger Y, Levchenko A, Le N (2022). Connectome: computation and visualization of cell–cell signaling topologies in single-cell systems data. Sci Rep.

[104] Solovey M, Scialdone A (2020). Comunet: a tool to explore and visualize intercellular communication. Bioinformatics.

[105] Cillo AR, Kürten CHL, Tabib T, Qi Z, Onkar S, Wang T, Liu A, Duvvuri U, Kim S, Soose RJ, Oesterreich S, Chen W, Lafyatis R, Bruno TC, Ferris RL, Vignali DAA (2020). Immune landscape of viral- and carcinogen-driven head and neck cancer. Immunity.

[106] Yuanxin W, Ruiping W, Shaojun Z, Shumei S, Changying J, Guangchun H, Michael W, Jaffer A, Andy F, Wang L (2019). iTALK: an R package to characterize and illustrate intercellular communication. Nature.

[107] Hu Y, Peng T, Gao L, Tan K (2021). CytoTalk: De novo construction of signal transduction networks using single-cell transcriptomic data. Sci Adv.

[108] Browaeys R, Saelens W, Sayes Y (2019). NicheNet: modeling intercellular communication by linking ligands to target genes. Nat Method.

[109] Zhang Y, Liu T, Hu X, Wang M, Wang J, Zou B, Tan P, Cui T, Dou Y, Ning L, Huang Y, Rao S, Wang D, Zhao X (2021). Cellcall: integrating paired ligand–receptor and transcription factor activities for cell–cell communication. Nucleic Acids Res.

[110] Yu A, Li Y, Li I, Yeh C, Chiou AE, Ozawa MG, Taylor J, Plevritis SK (2021). Reconstructing co-dependent cellular crosstalk in lung adenocarcinoma using REMI. bioRxiv.

[111] Pham D, Tan X, Xu J, Grice LF, Lam PY, Raghubar A, Vukovic J, Ruitenberg MJ, Nguyen Q (2021). stLearn: integrating spatial location, tissue morphology and gene expression to find cell types, cell–cell interactions and spatial trajectories within undissociated tissues. bioRxiv.

[112] Wang S, MacLean A, Nie Q (2018). SoptSC: similarity matrix optimization for clustering, lineage, and signaling inference. bioRxiv.

[113] Liu Z, Sun D, Wang C (2022). Evaluation of cell–cell interaction methods by integrating single-cell RNA sequencing data with spatial information. BMC Genome Biol.

[114] Yokota T, Zhao Q, Cichocki A (2016). Smooth PARAFAC decomposition for tensor completion. IEEE Trans Signal Process.

[115] Ge H, Caverlee J, Zhang N, Squicciarini A. Uncovering the spatio-temporal dynamics of memes in the presence of incomplete information. In: Proceedings of CIKM’16, pp 1493–1502 (2016). 10.1145/2983323.2983782

[116] Park, J.Y., Carr, K.T., Zheng, S., Yue, Y., Yu, R.: Multiresolution tensor learning for efficient and interpretable spatial analysis. In: Proceedings of ICML2020 695, pp 7499–7509 (2020)

[117] Sasagawa Y, Nikaido I, Hayashi T, Danno H, Uno KD, Imai T, Ueda HR (2013). Quartz-Seq: a highly reproducible and sensitive single-cell RNA sequencing method, reveals non-genetic gene-expression heterogeneity. BMC Genome Biol.

[118] Pierson E, Yau C (2015). Zifa: dimensionality reduction for zero-inflated single-cell gene expression analysis. BMC Genome Biol.

[119] Graeber TG, Eisenberg D (2001). Bioinformatic identification of potential autocrine signaling loops in cancers from gene expression profiles. Nat Genet.

[120] Harding SD, Sharman JL, Faccenda E, Southan C, Pawson AJ, Ireland S, Gray AJG, Bruce L, Alexander SPH, Anderton S, Bryant C, Davenport AP, Doerig C, Fabbro D, Levi-Schaffer F, Spedding M, Davies N-I (2018). The IUPHAR/BPS guide to pharmacology in 2018: updates and expansion to encompass the new guide to immunopharmacology. Nucleic Acids Res.

[121] Ben-Shlomo I, Yu Hsu S, Rauch R, Kowalski HW, Hsueh AJ (2003). Signaling receptome: a genomic and evolutionary perspective of plasma membrane receptors involved in signal transduction. Sci Signal.

[122] Hutchins LN, Murphy SM, Singh P, Graber JH (2008). Position-dependent motif characterization using non-negative matrix factorization. Bioinformatics.

[123] Chicco D (2017). Ten quick tips for machine learning in computational biology. BMC BioData Min.

[124] Saito T, Rehmsmeier M (2015). The precision-recall plot is more informative than the ROC plot when evaluating binary classifiers on imbalanced datasets. PLoS ONE.

[125] Davis J, Goadrich M. The relationship between precision–recall and roc curves. In: Proceedings of ICML’06, pp 233–240 (2006). 10.1145/1143844.1143874

[126] Chicco D, Jurman G (2020). The advantages of the Matthews correlation coefficient (MCC) over f1 score and accuracy in binary classification evaluation. BMC Genom.

[127] Boughorbel S, Jarray F, El-Anbari M (2017). Optimal classifier for imbalanced data using Matthews correlation coefficient metric. PLoS ONE.

[128] Chicco D, Tötsch N, Jurman G (2021). The Matthews correlation coefficient (MCC) is more reliable than balanced accuracy, bookmaker informedness, and markedness in two-class confusion matrix evaluation. BMC BioData Min.

