# Supplementary material for: Sctensor detects many-to-many cell–cell interactions from single cell RNA-sequencing data
Source: BMC Bioinformatics. 2023 Nov 7;24:420. doi: 10.1186/s12859-023-05490-y (PMC10631077; doi:10.1186/s12859-023-05490-y)
Supplement: Supplementary file 6 — Additional file 6. Computational time values of all methods. [file 12859_2023_5490_MOESM6_ESM.pdf]

Simulated Datasets

E2 (Summary) The larger, the worse

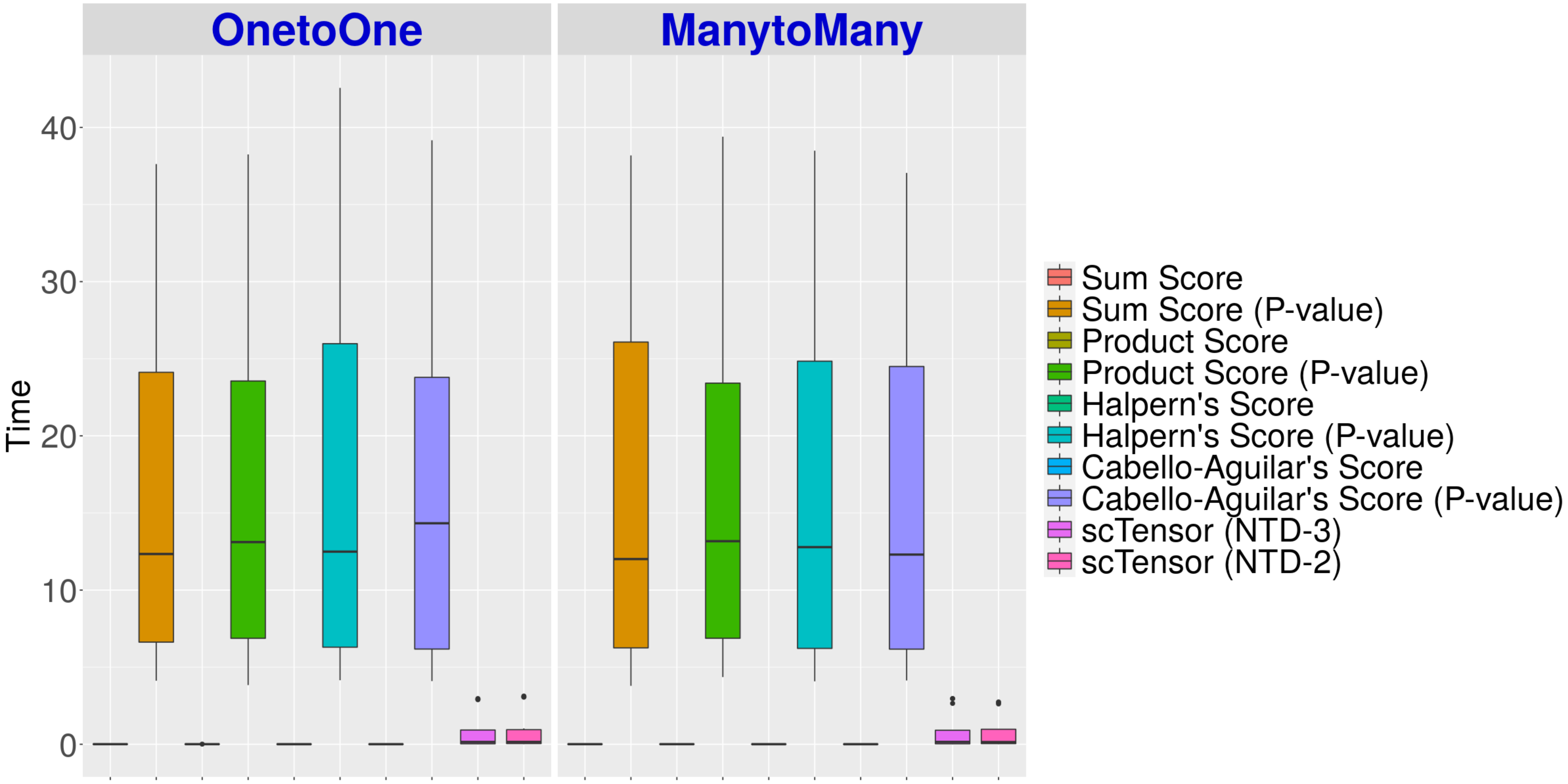

E5 (Summary) The larger, the worse

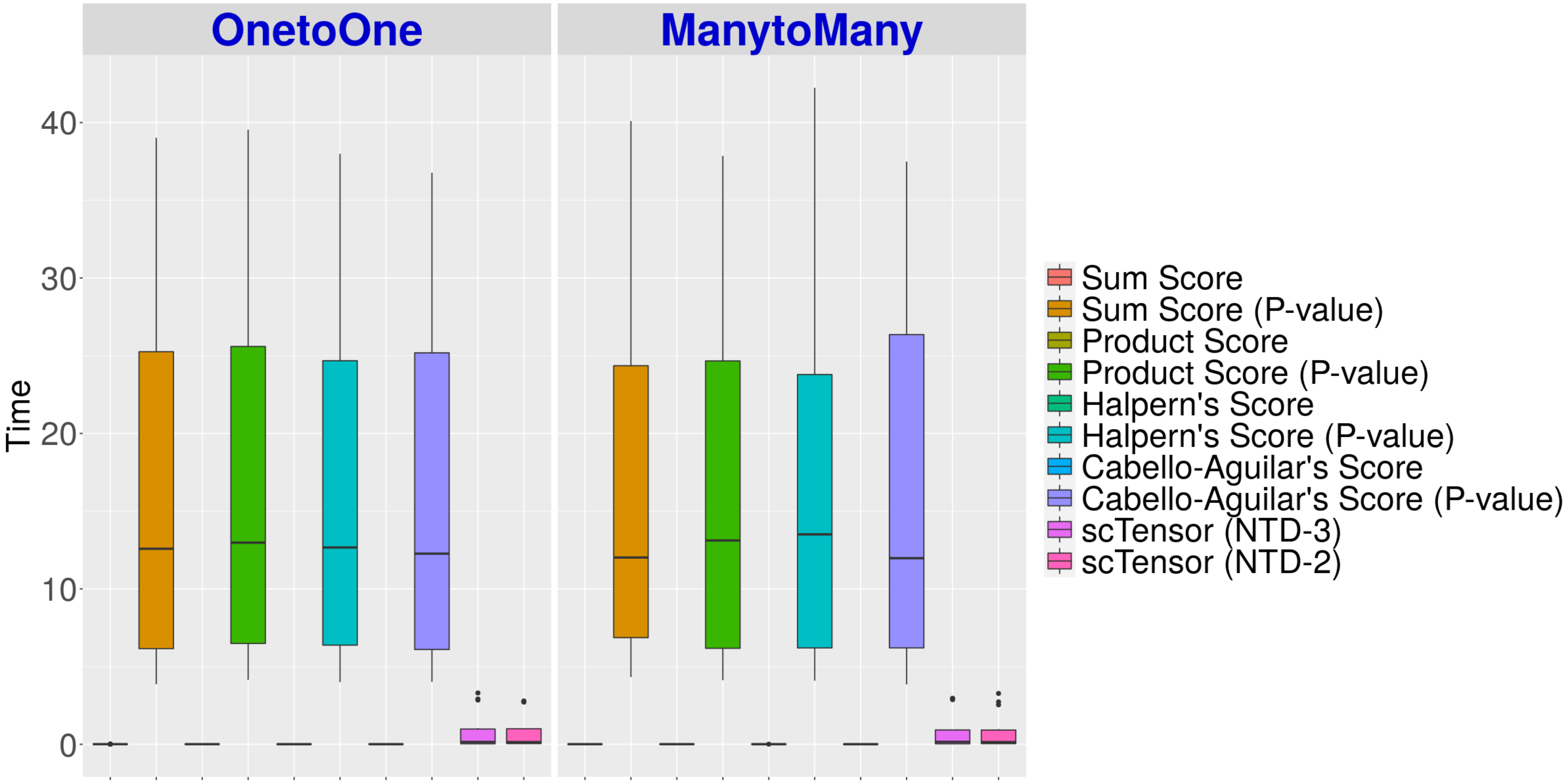

E10 (Summary) The larger, the worse

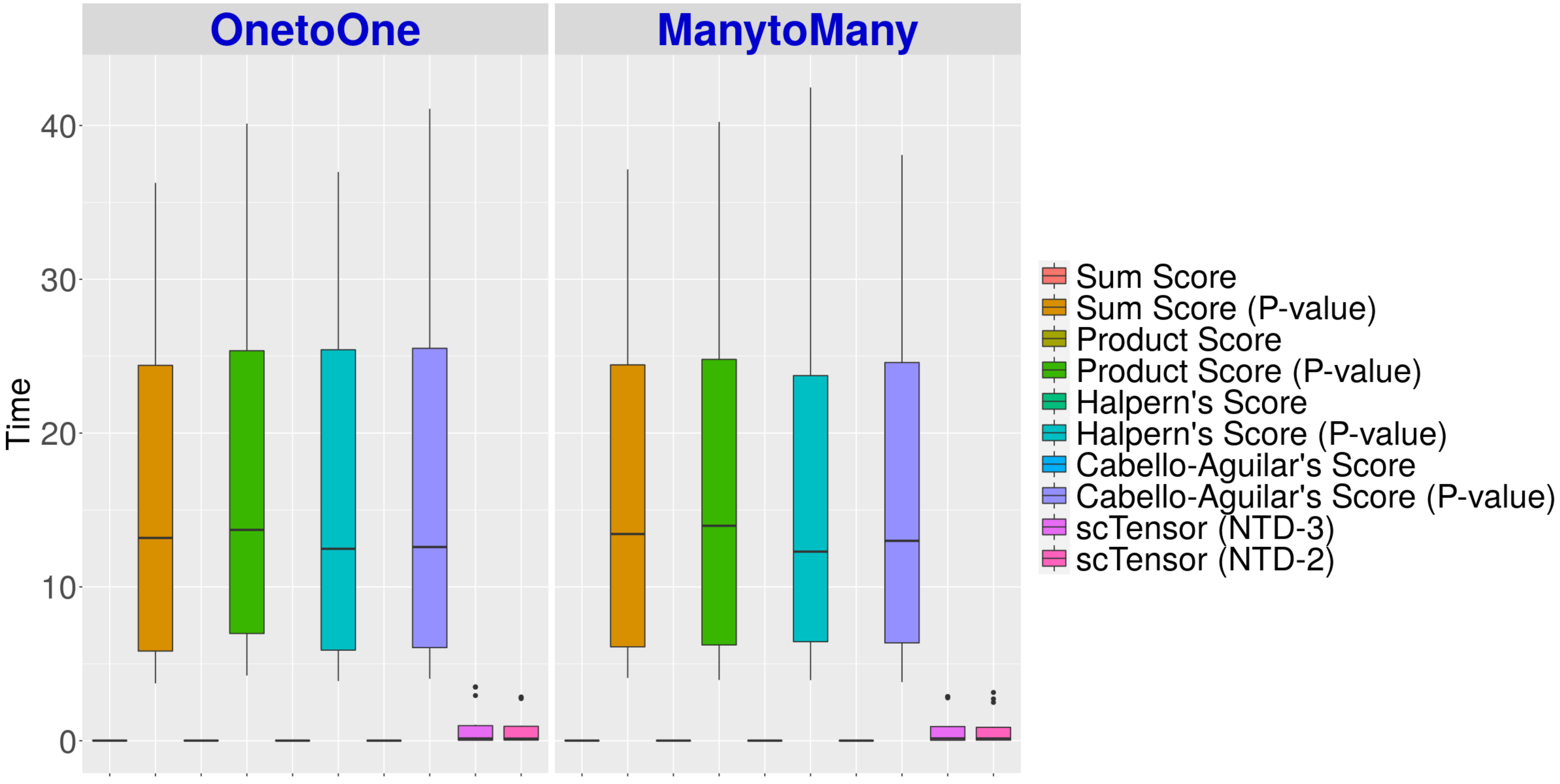

E2 (Details)

The larger, the worse

Sum Score

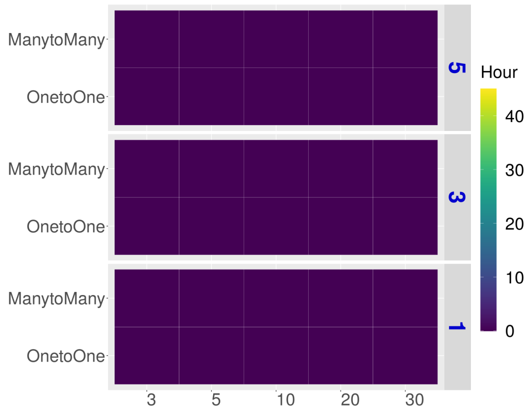

Product Score

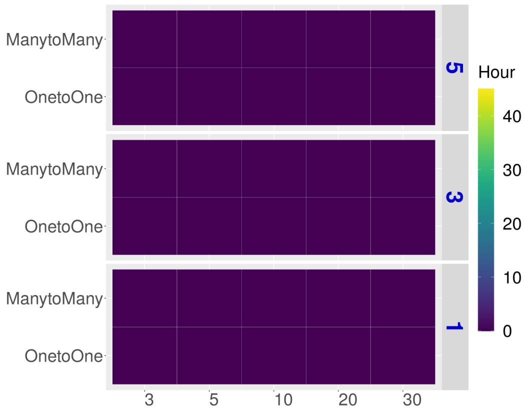

Halpern's Score

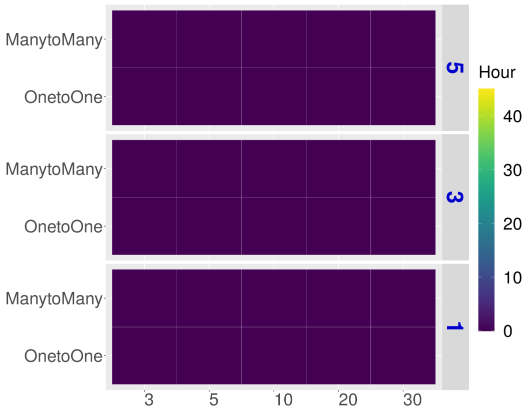

Cabello-Aguilar's Score

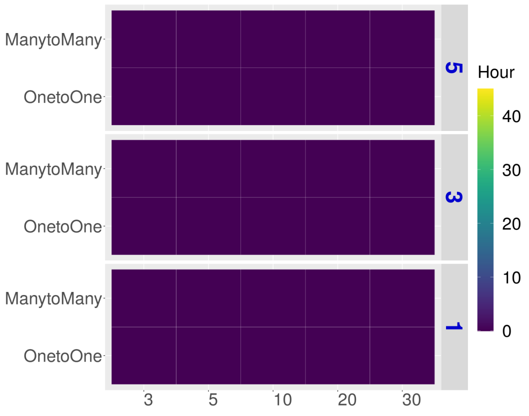

scTensor (NTD-3)

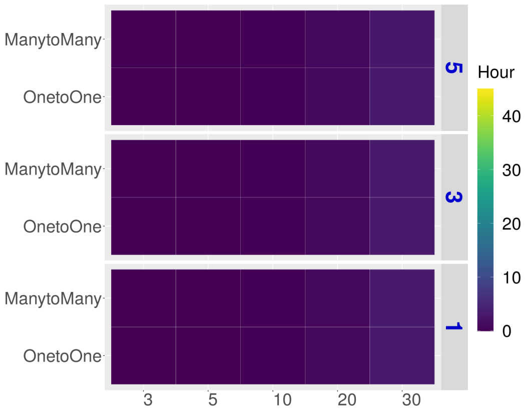

Sum Score (P-value)

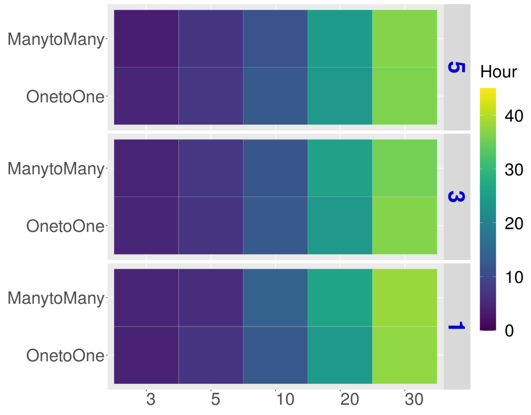

Product Score (P-value)

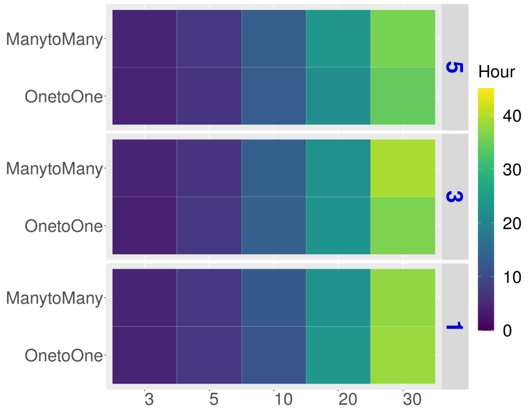

Halpern's Score (P-value)

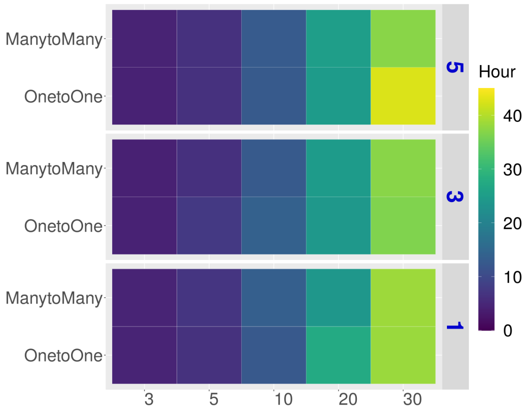

Cabello-Aguilar's Score (P-value)

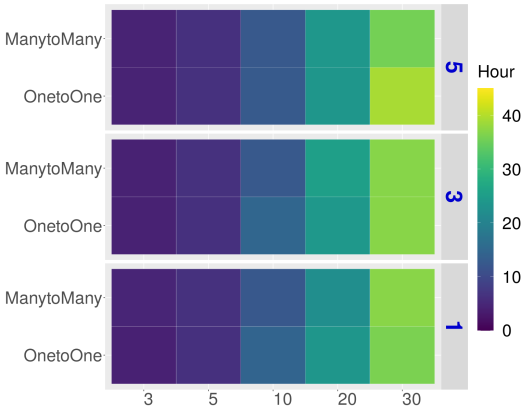

scTensor (NTD-2)

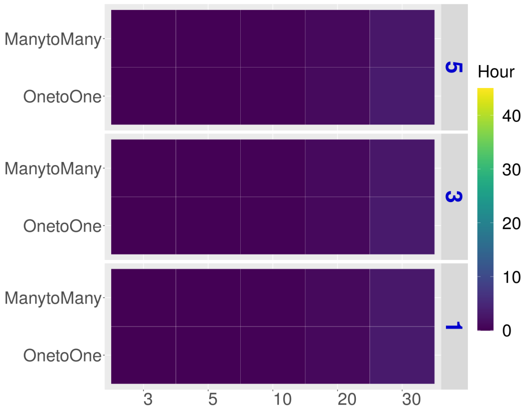

E5 (Details)

The larger, the worse

Sum Score

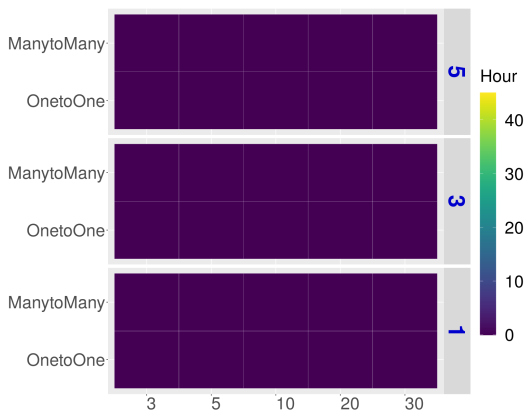

Product Score

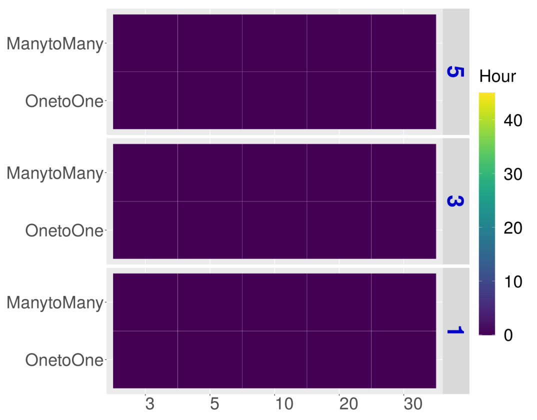

Halpern's Score

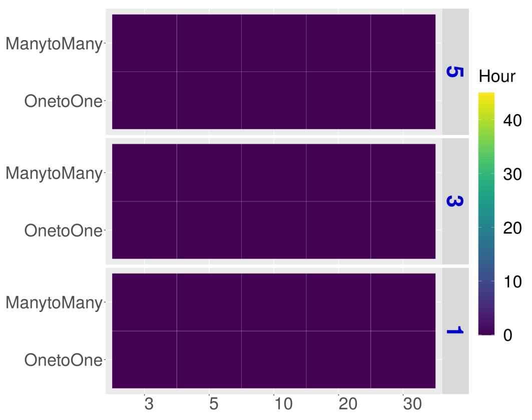

Cabello-Aguilar's Score

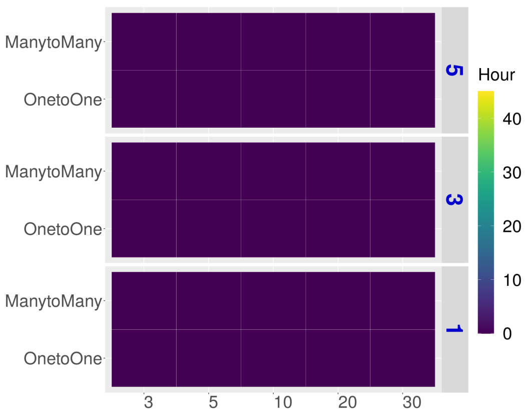

scTensor (NTD-3)

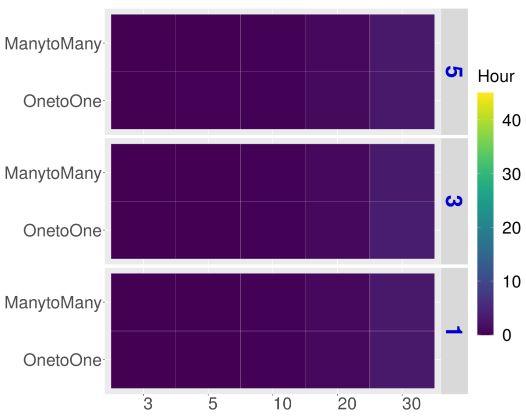

Sum Score (P-value)

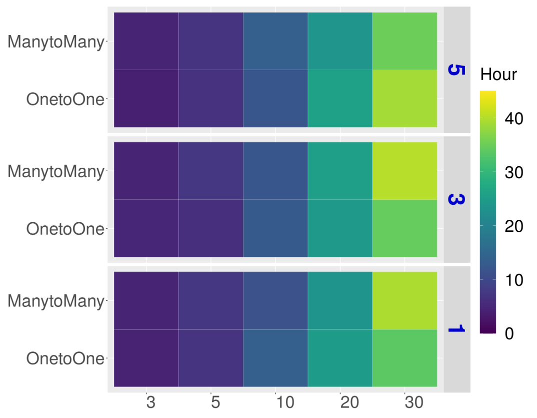

Product Score (P-value)

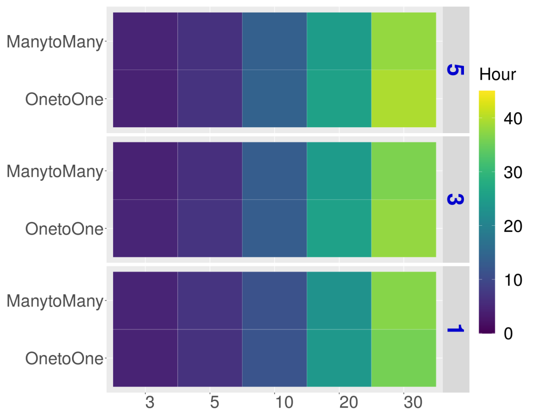

Halpern's Score (P-value)

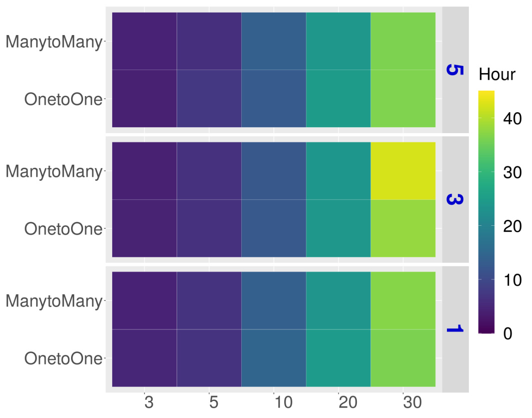

Cabello-Aguilar's Score (P-value)

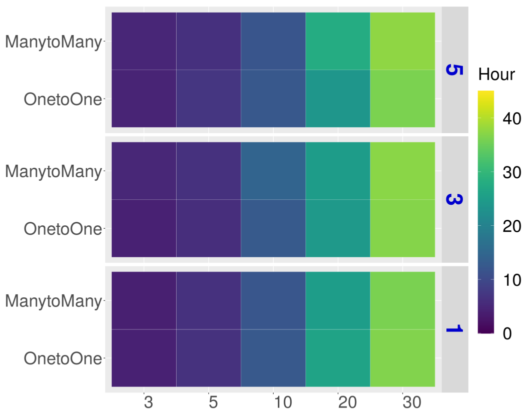

scTensor (NTD-2)

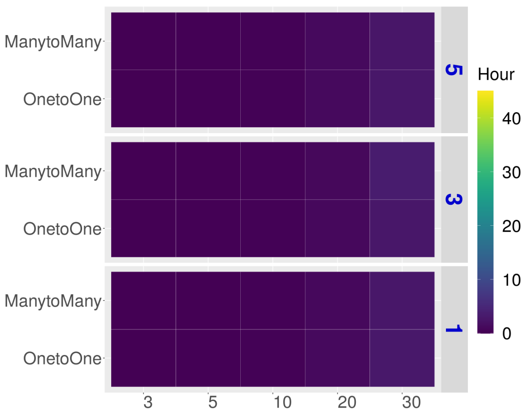

E10 (Details)

The larger, the worse

Sum Score

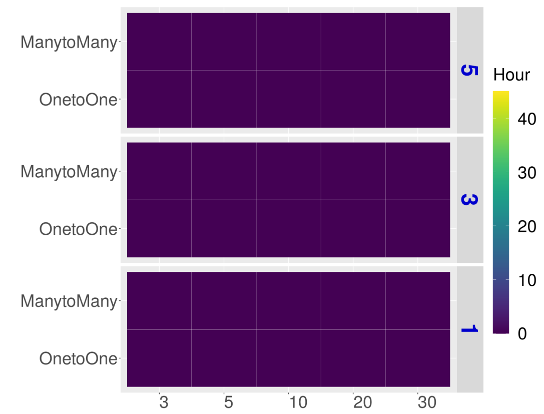

Product Score

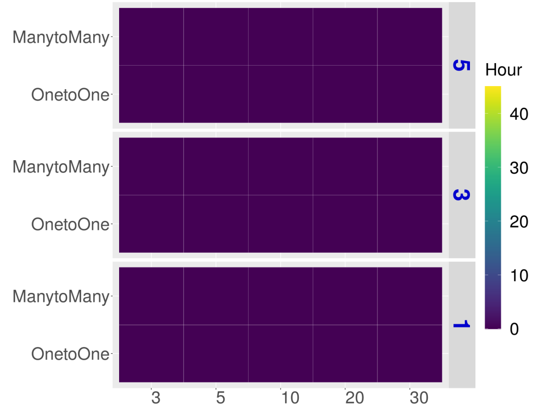

Halpern's Score

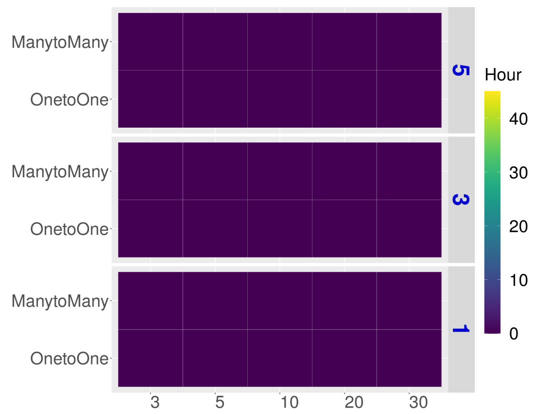

Cabello-Aguilar's Score

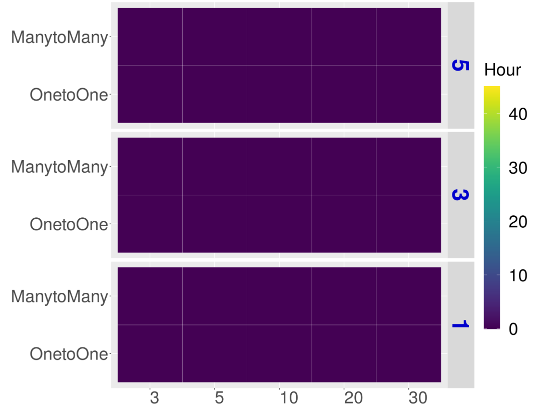

scTensor (NTD-3)

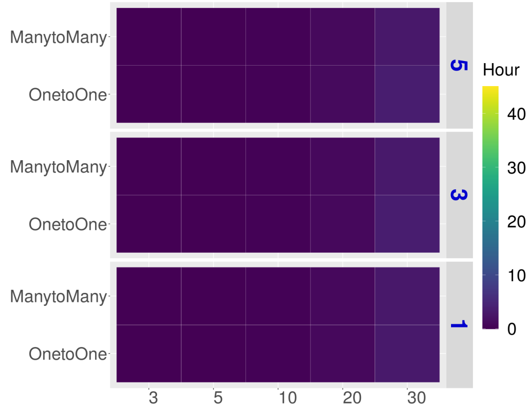

Sum Score (P-value)

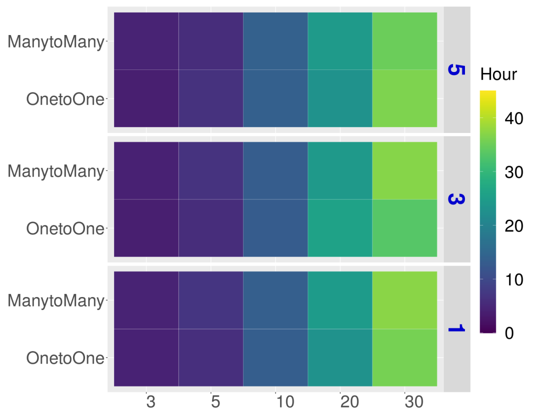

Product Score (P-value)

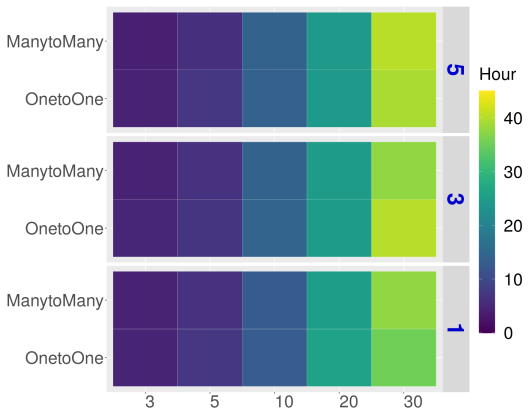

Halpern's Score (P-value)

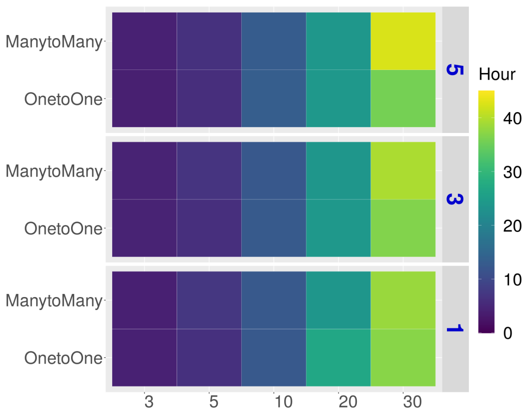

Cabello-Aguilar's Score (P-value)

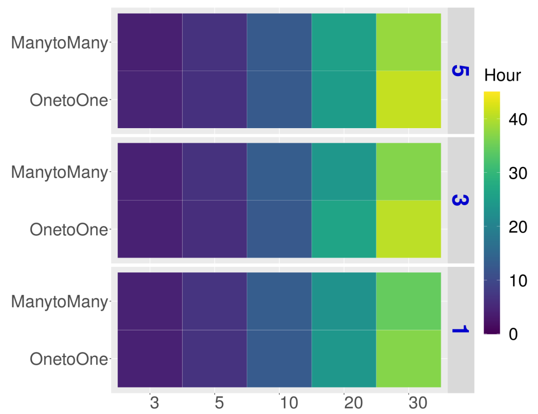

scTensor (NTD-2)

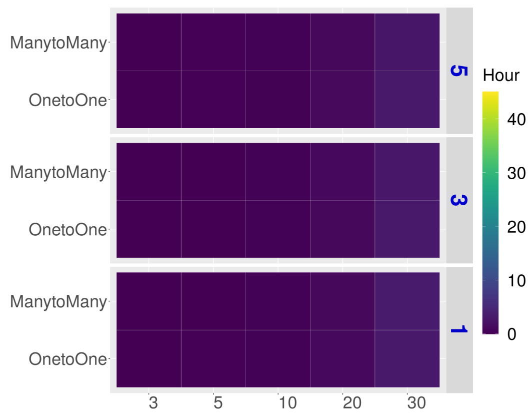

Real Datasets

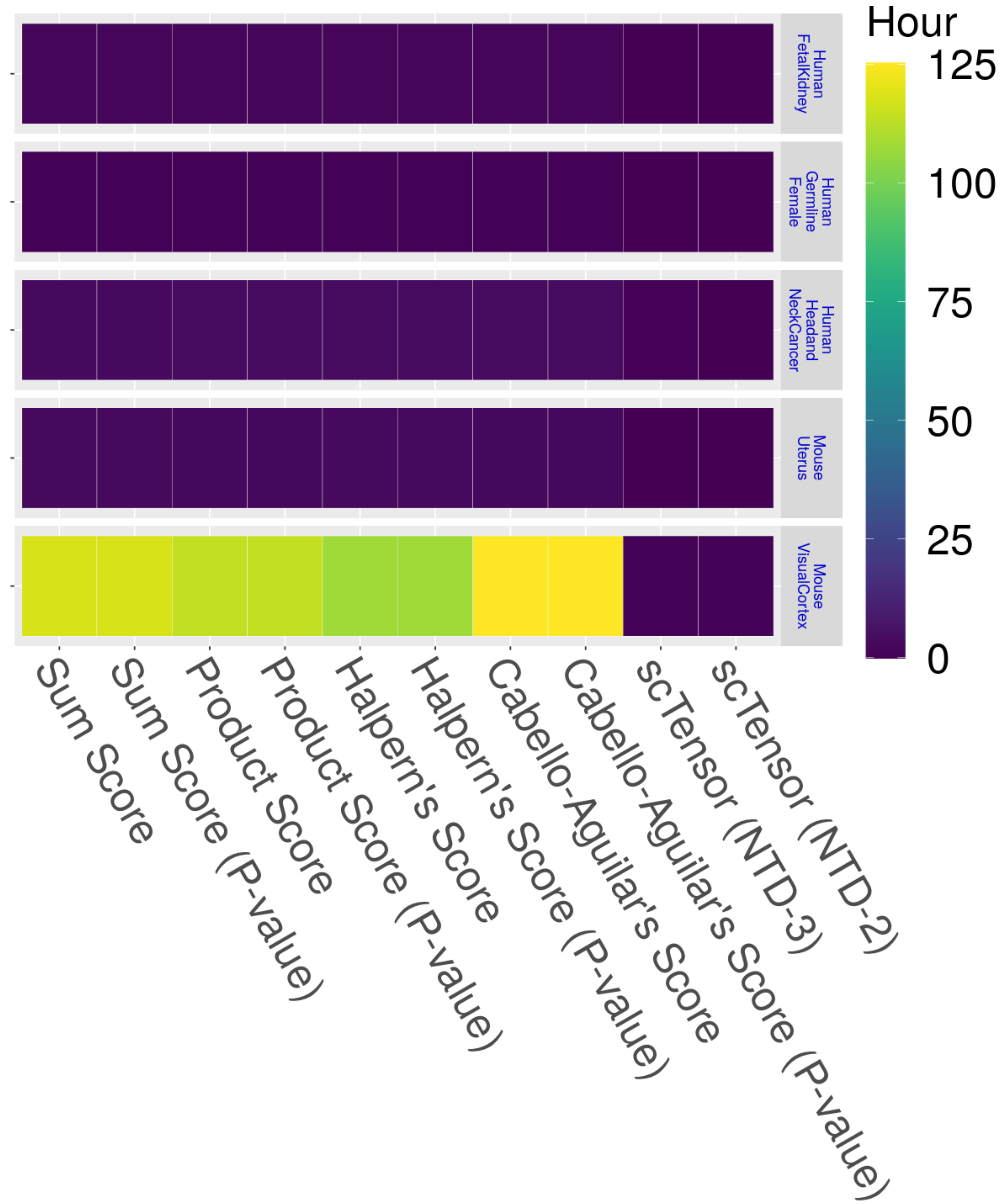
Hour
125
100
75
50
25
0
Human FetalKidney
Human Germline Female
Human Headand NeckCancer
Mouse Uterus
Mouse VisualCortex
Sum Score
Sum Score (P-value)
Product Score
Product Score (P-value)
Halpern's Score
Halpern's Score (P-value)
Cabello-Aguilar's Score
Cabello-Aguilar's Score (P-value)
scTensor (NTD-3)
scTensor (NTD-2)
