# Supplementary material for: Sctensor detects many-to-many cell–cell interactions from single cell RNA-sequencing data
Source: BMC Bioinformatics. 2023 Nov 7;24:420. doi: 10.1186/s12859-023-05490-y (PMC10631077; doi:10.1186/s12859-023-05490-y)
Supplement: Supplementary file 7 — Additional file 7. F-measure values of all binarization methods. [file 12859_2023_5490_MOESM7_ESM.pdf]

Simulated Datasets

E2 (Summary) The value ranges 0 to 1 (the closer to 1, the better)

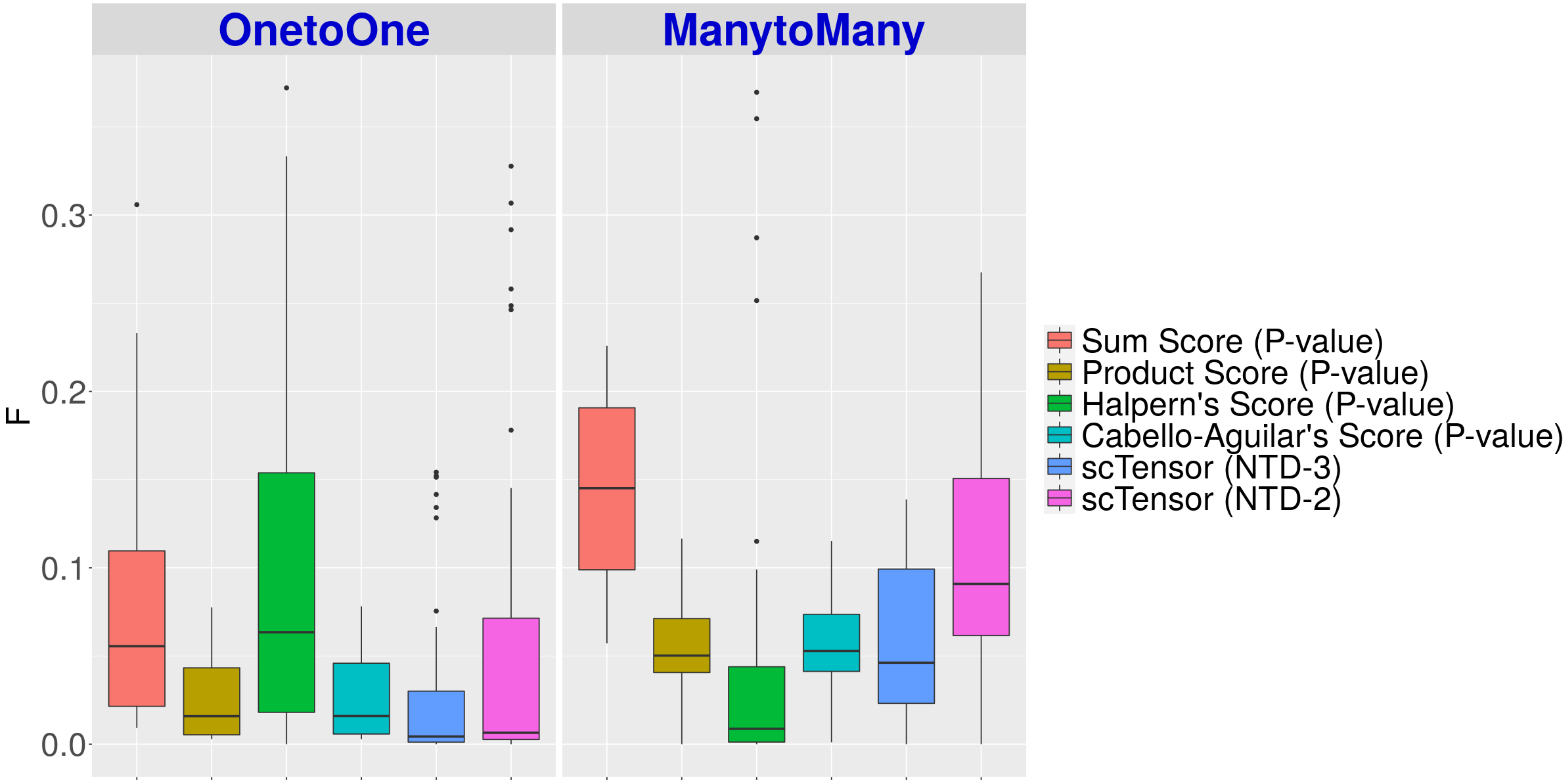

E5 (Summary) The value ranges 0 to 1 (the closer to 1, the better)

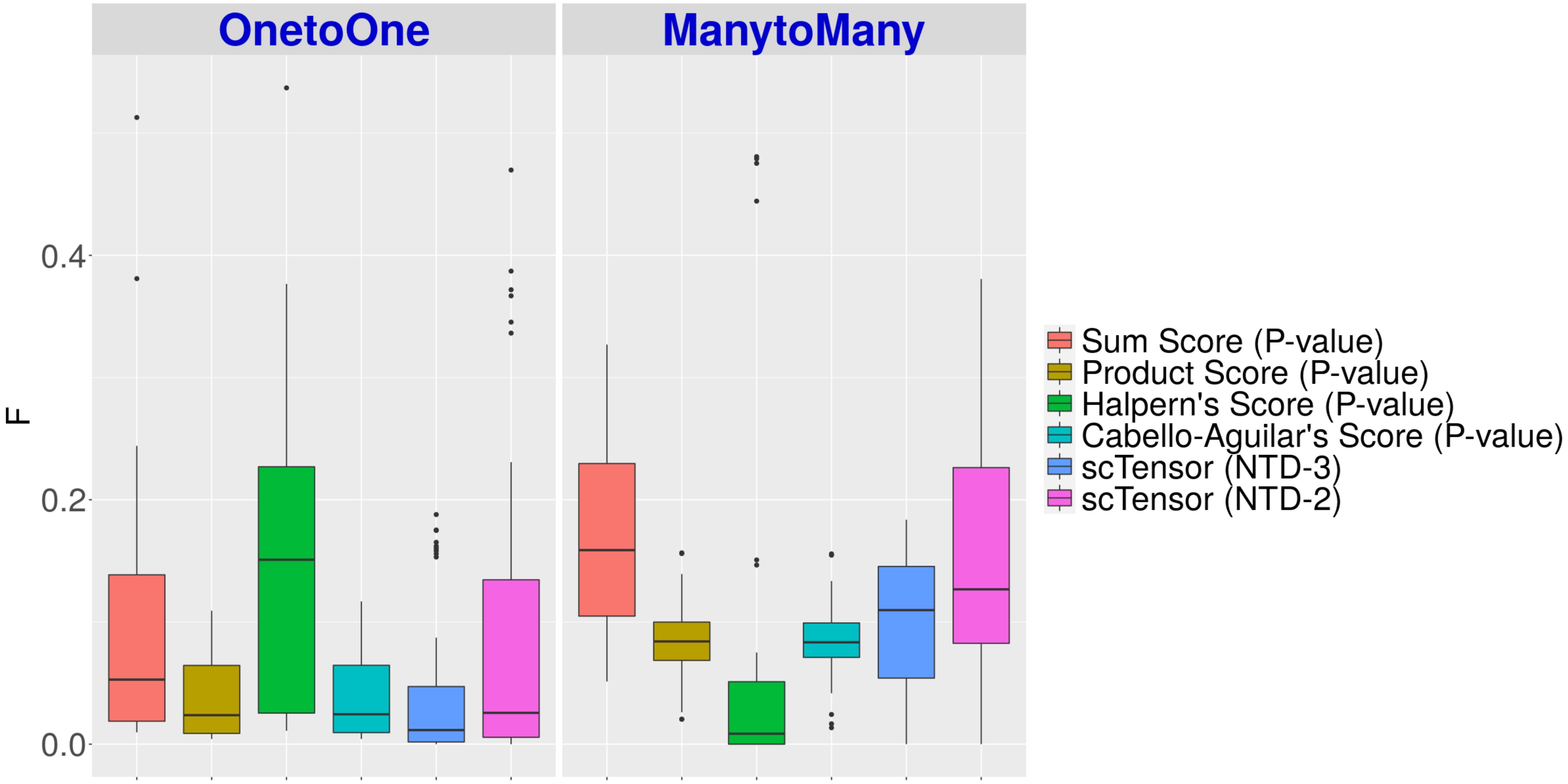

E10 (Summary) The value ranges 0 to 1 (the closer to 1, the better)

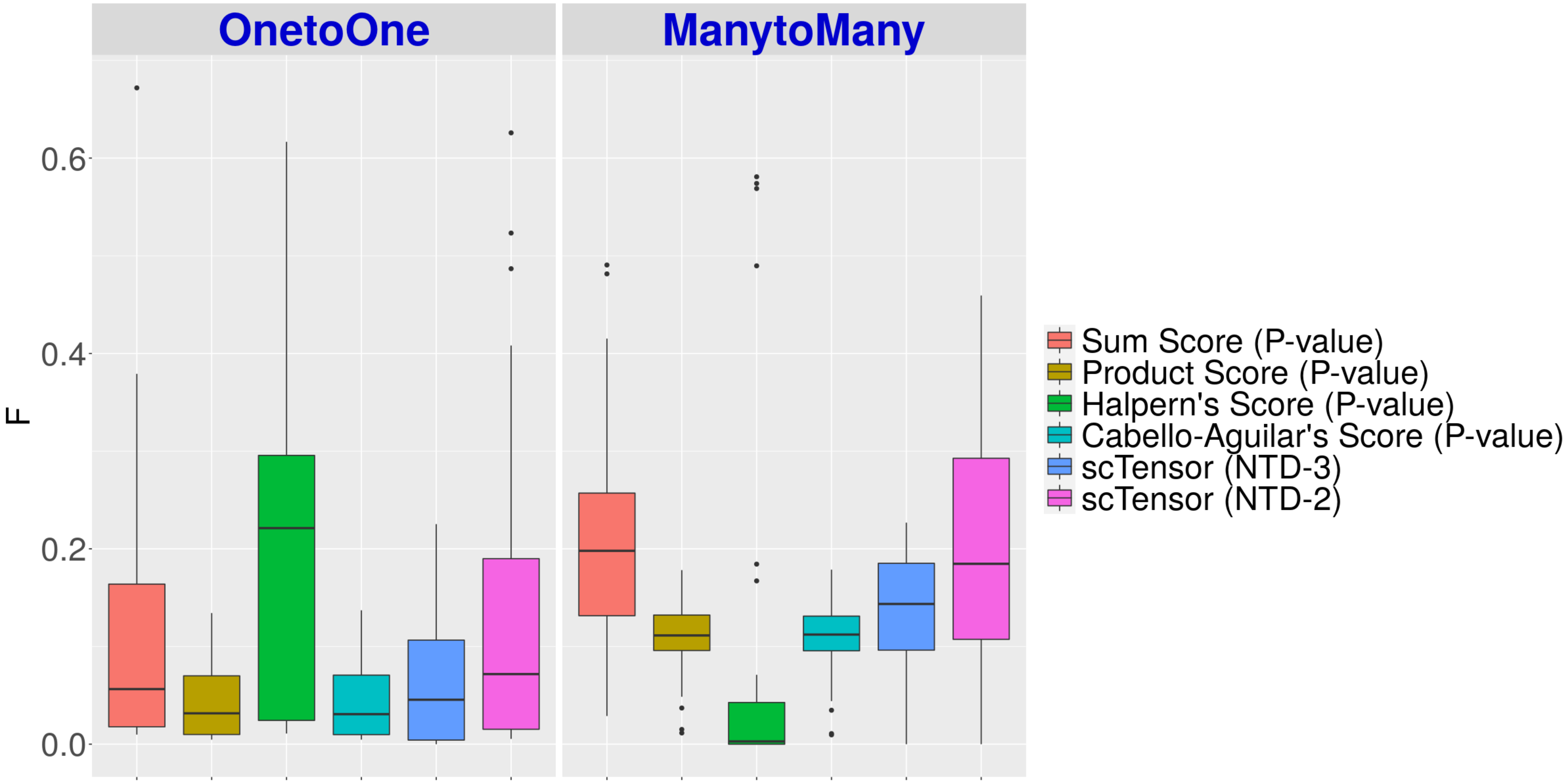

E2 (Details) The value ranges 0 to 1 (the closer to 1, the better)

These may only work in certain cases
but overall these do not work well.

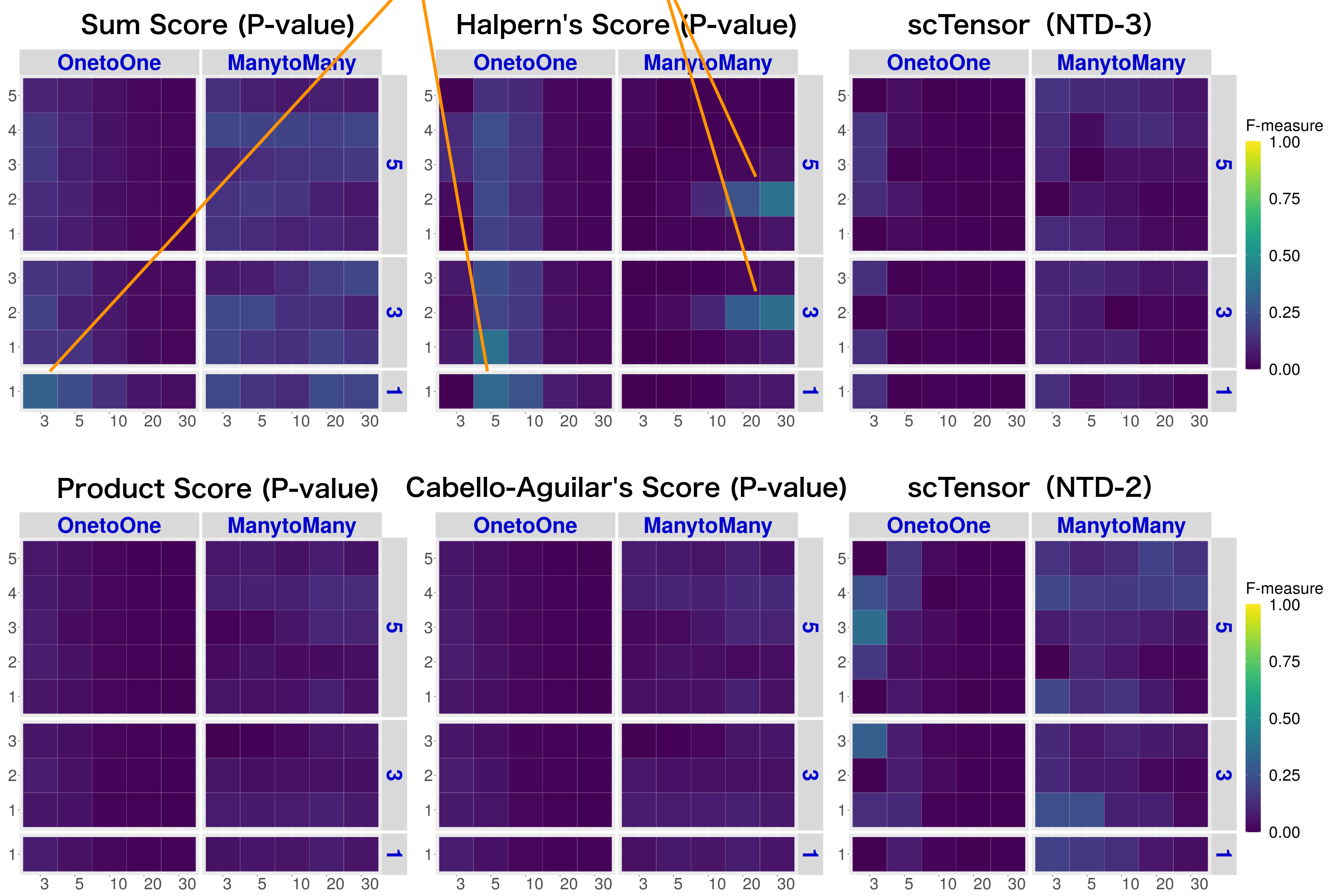

Stable in both 1vs1
and many vs many

E5 (Details) The value ranges 0 to 1 (the closer to 1, the better)

These may only work in certain cases
but overall these do not work well.

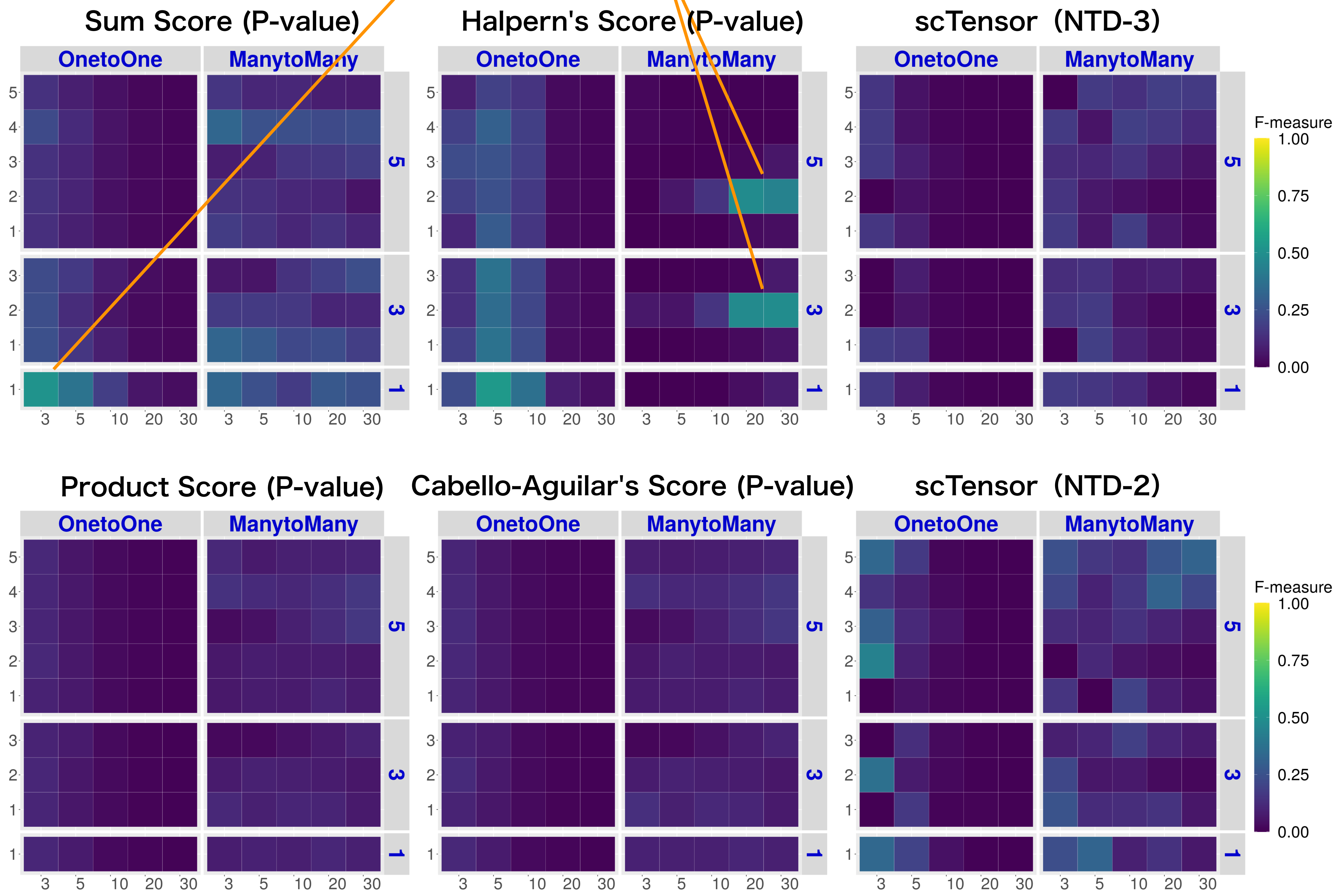

Stable in both 1vs1
and many vs many

E10 (Details) The value ranges 0 to 1 (the closer to 1, the better)

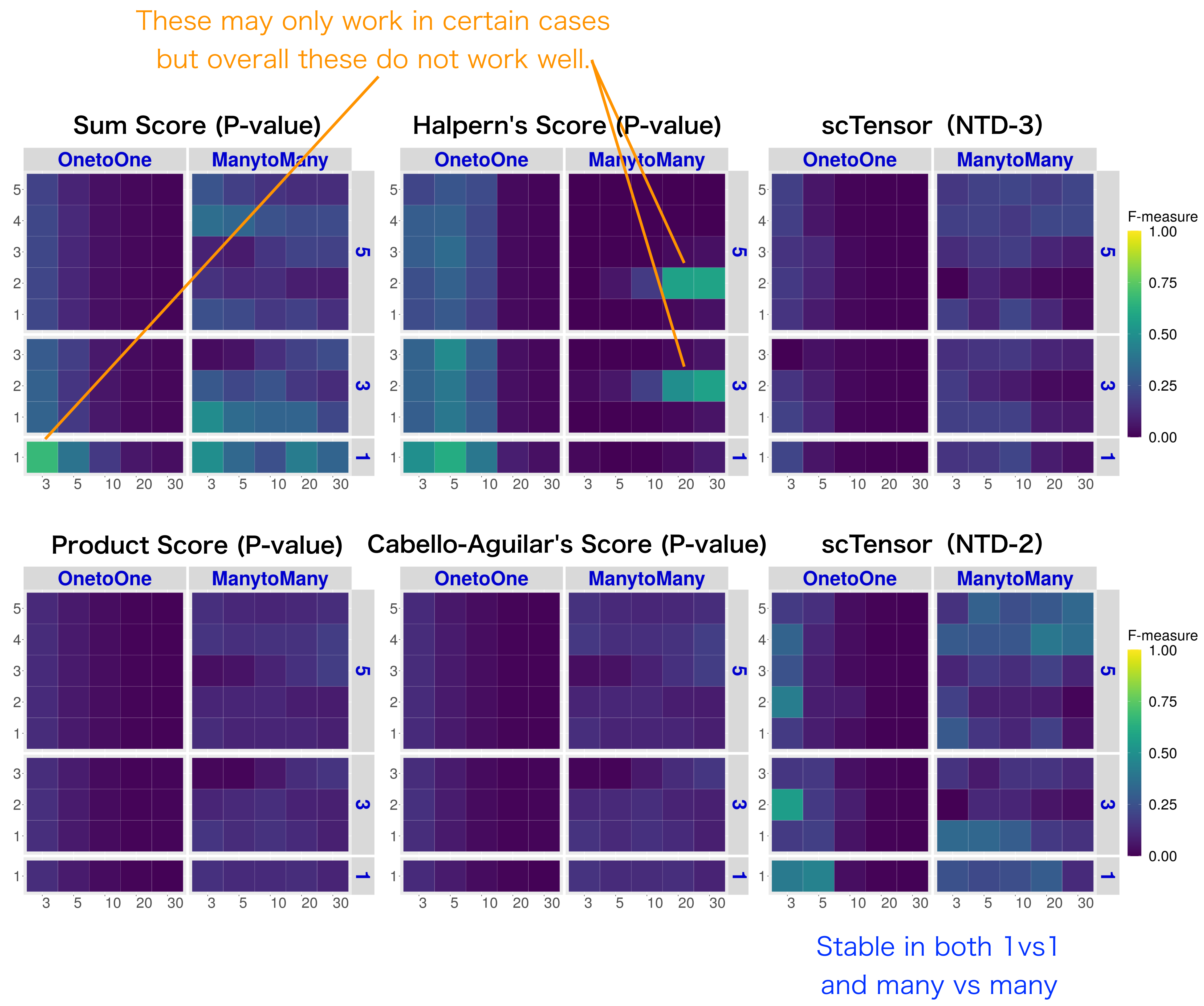

Real Datasets

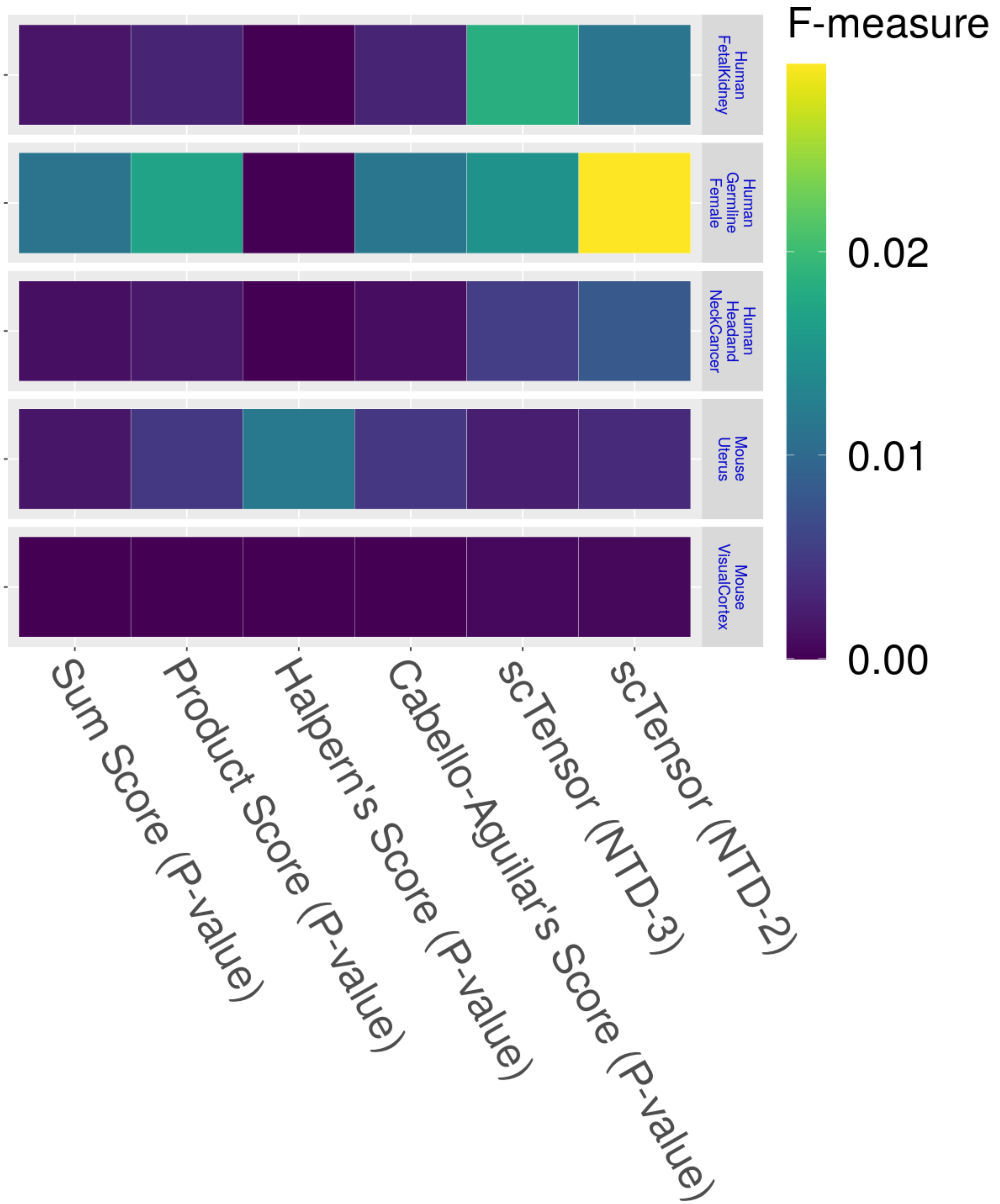

F-measure
0.02
0.01
0.00
Human FetalKidney
Human Germline Female
Human Headand NeckCancer
Mouse Uterus
Mouse VisualCortex
Sum Score (P-value)
Product Score (P-value)
Halpern's Score (P-value)
Cabello-Aguilar's Score (P-value)
scTensor (NTD-3)
scTensor (NTD-2)
