# Supplementary material for: Sctensor detects many-to-many cell–cell interactions from single cell RNA-sequencing data
Source: BMC Bioinformatics. 2023 Nov 7;24:420. doi: 10.1186/s12859-023-05490-y (PMC10631077; doi:10.1186/s12859-023-05490-y)
Supplement: Supplementary file 9 — Additional file 9. PR values of all binarization methods. [file 12859_2023_5490_MOESM9_ESM.pdf]

Simulated Datasets

E2 (Summary)

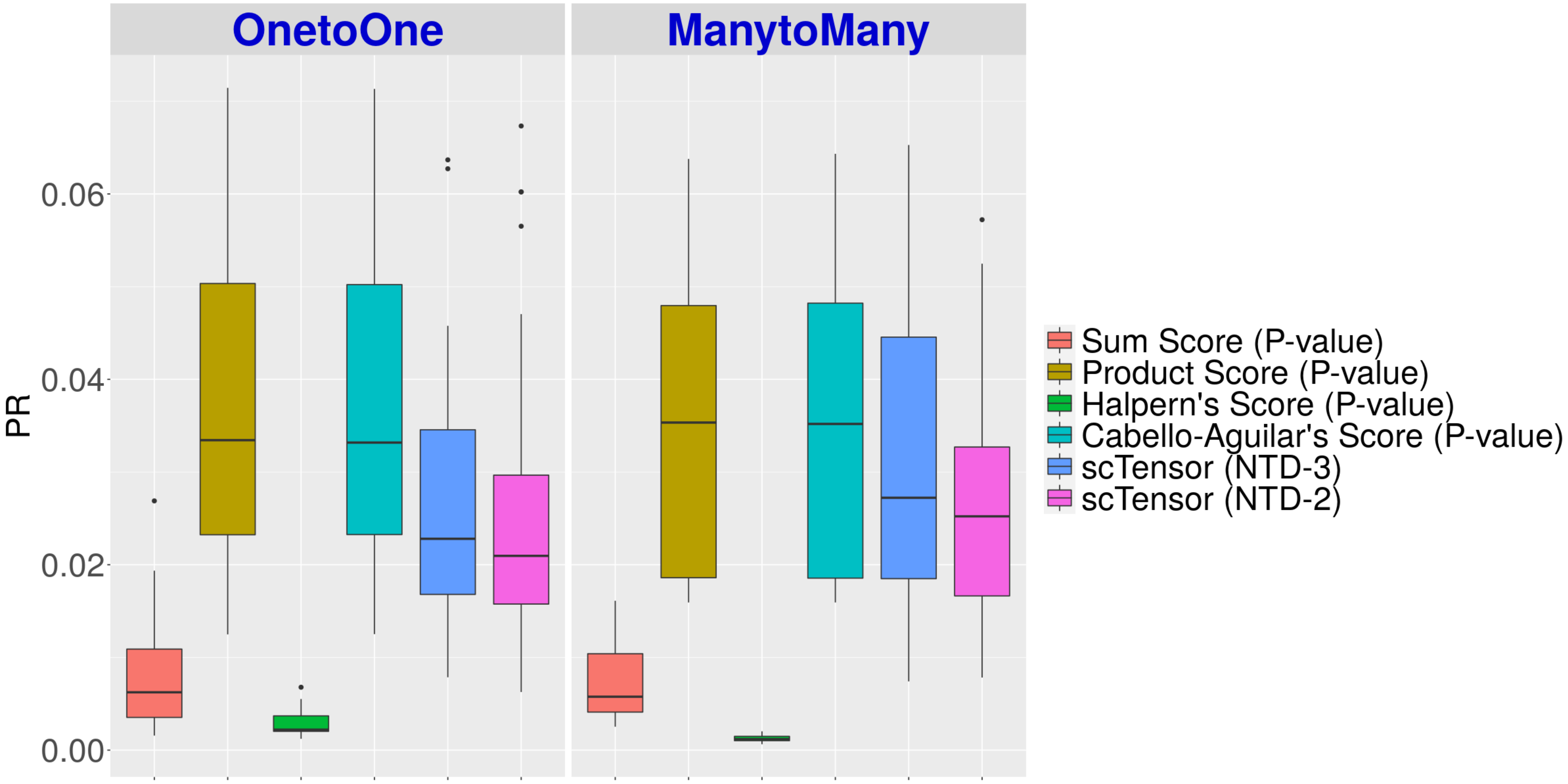

OnetoOne
ManytoMany
PR
0.06
0.04
0.02
0.00
Sum Score (P-value)
Product Score (P-value)
Halpern's Score (P-value)
Cabello-Aguilar's Score (P-value)
scTensor (NTD-3)
scTensor (NTD-2)

E5 (Summary)

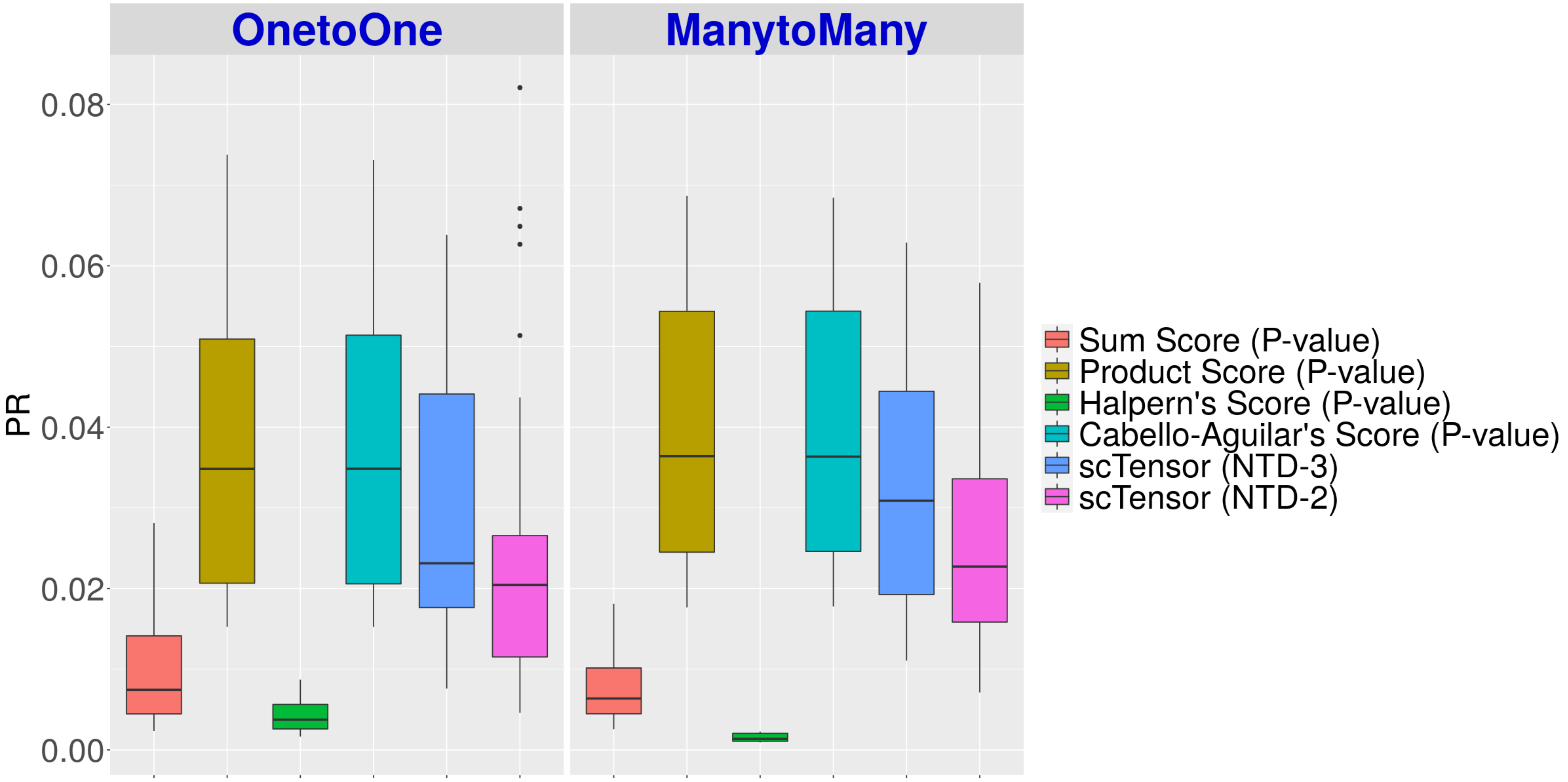

OnetoOne
ManytoMany
0.08
0.06
0.04
0.02
0.00
PR
Sum Score (P-value)
Product Score (P-value)
Halpern's Score (P-value)
Cabello-Aguilar's Score (P-value)
scTensor (NTD-3)
scTensor (NTD-2)

E10 (Summary)

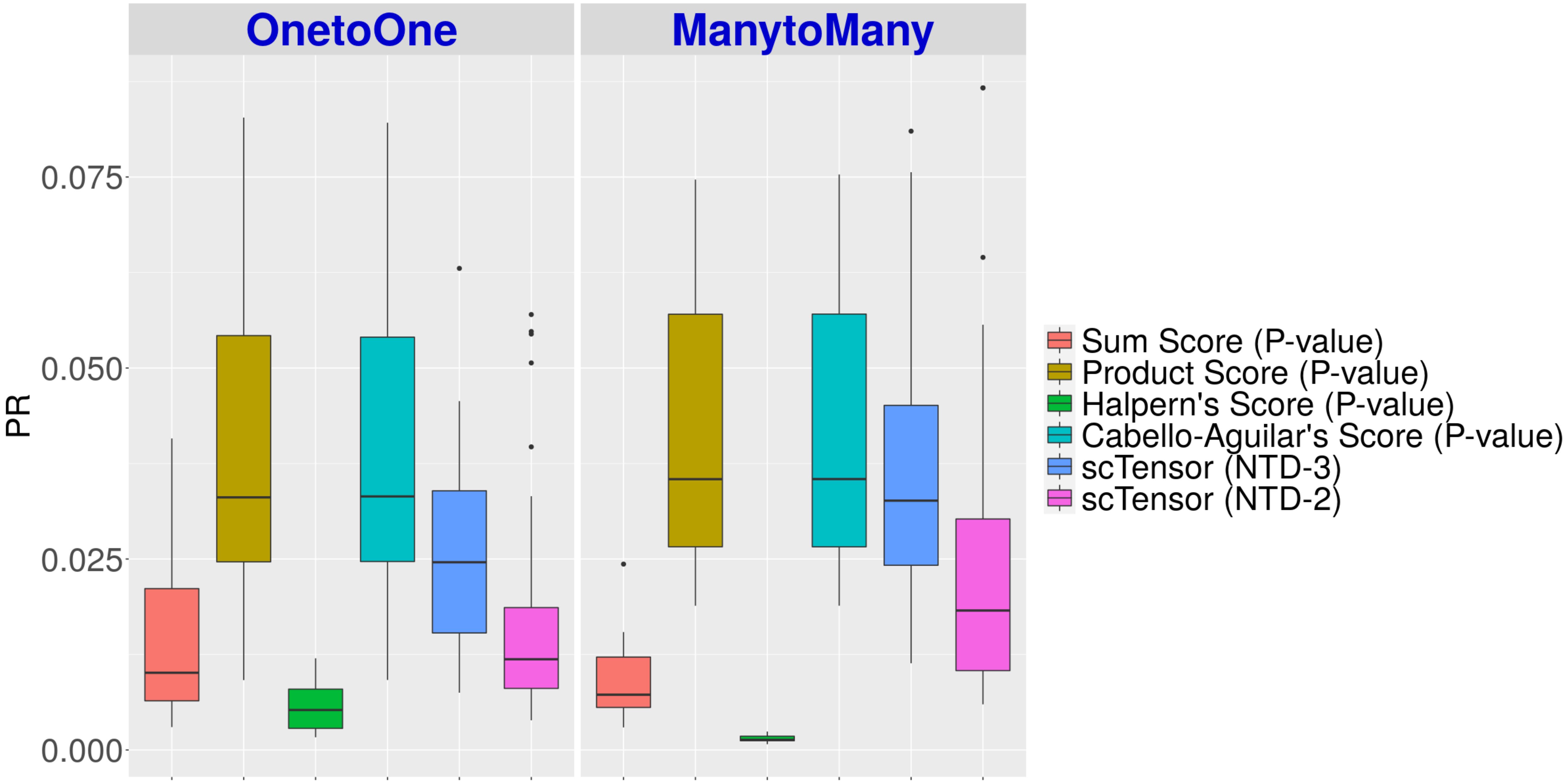
OnetoOne
ManytoMany
PR
0.000
0.025
0.050
0.075
Sum Score (P-value)
Product Score (P-value)
Halpern's Score (P-value)
Cabello-Aguilar's Score (P-value)
scTensor (NTD-3)
scTensor (NTD-2)

E2 (Details)

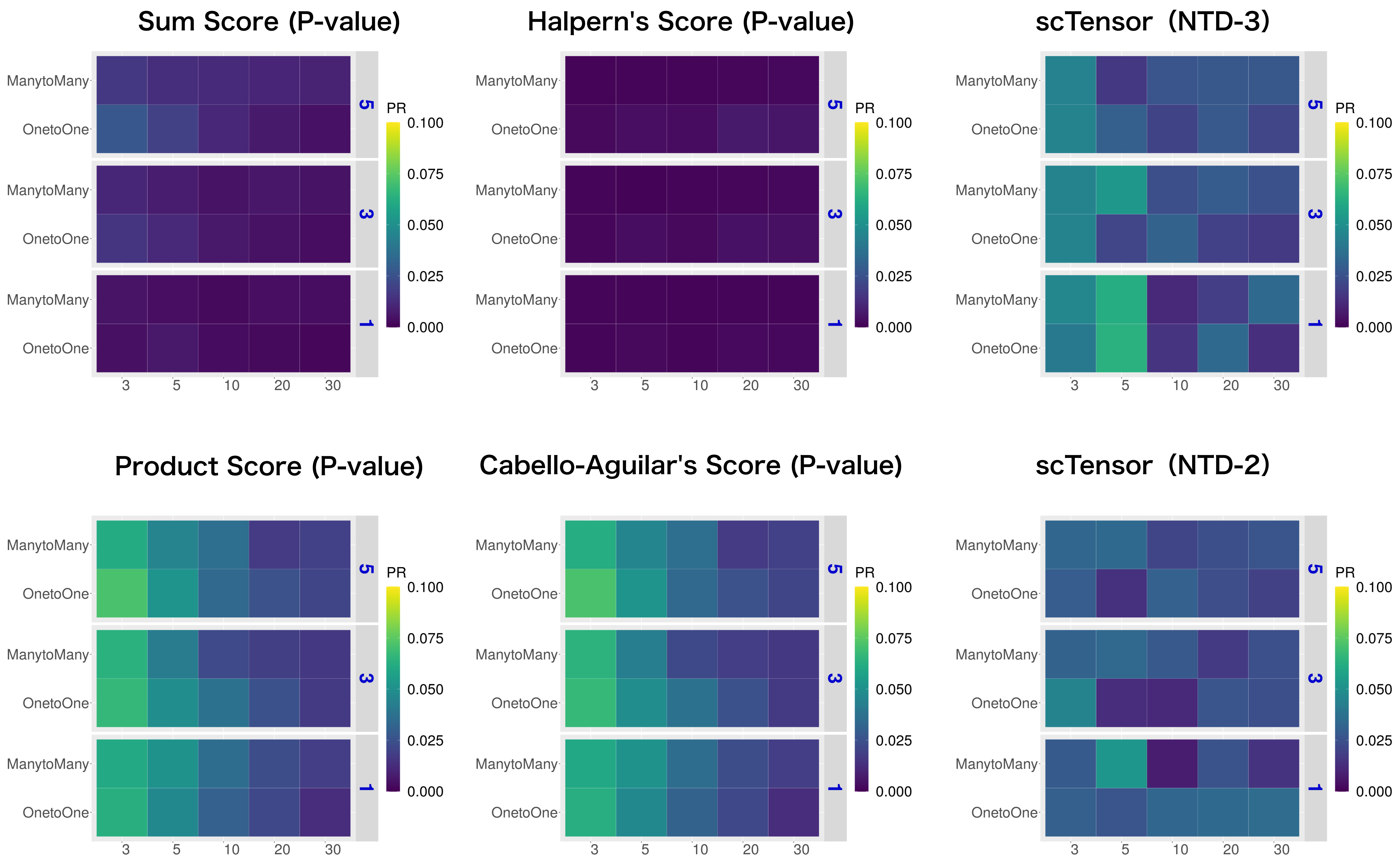

Sum Score (P-value)
Halpern's Score (P-value)
scTensor (NTD-3)
Product Score (P-value)
Cabello-Aguilar's Score (P-value)
scTensor (NTD-2)
ManytoMany
OnetoOne
5
3
1
PR
0.100
0.075
0.050
0.025
0.000
3
5
10
20
30

E5 (Details)

Sum Score (P-value)

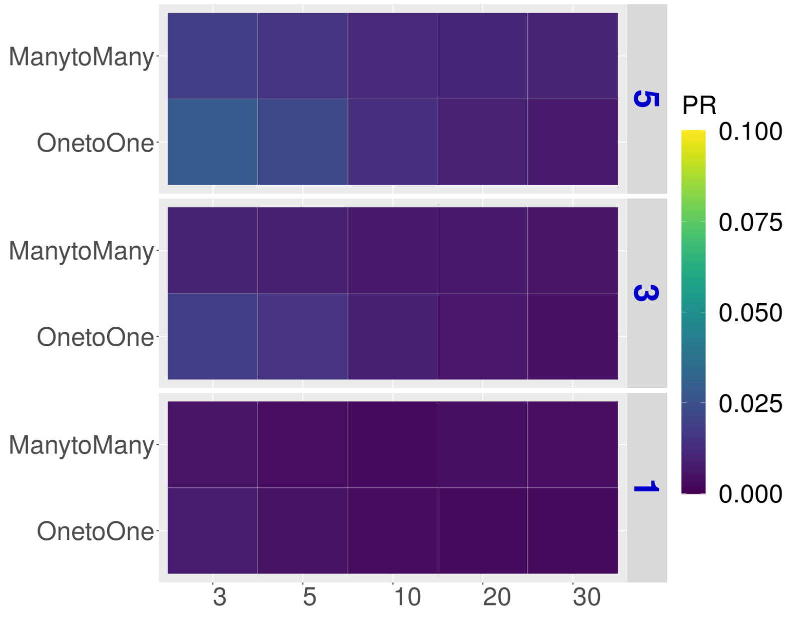

Halpern's Score (P-value)

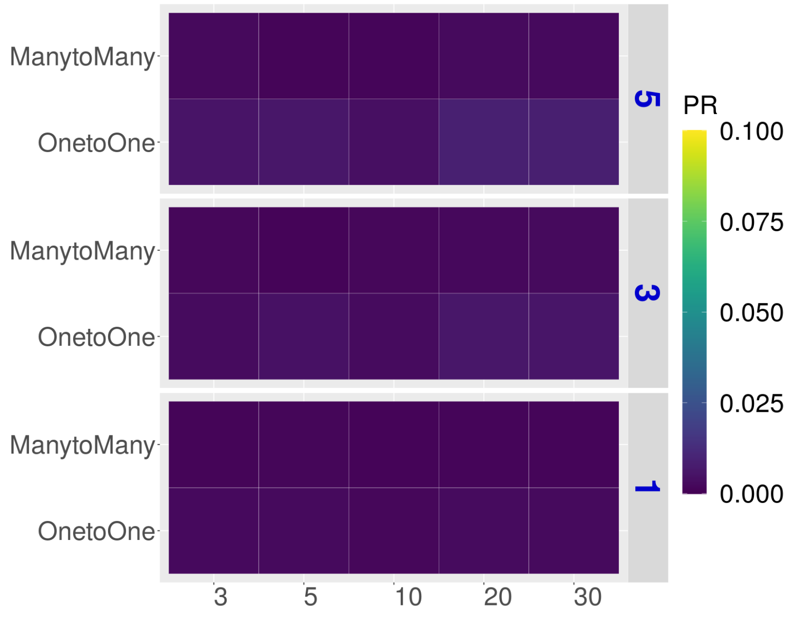

scTensor (NTD-3)

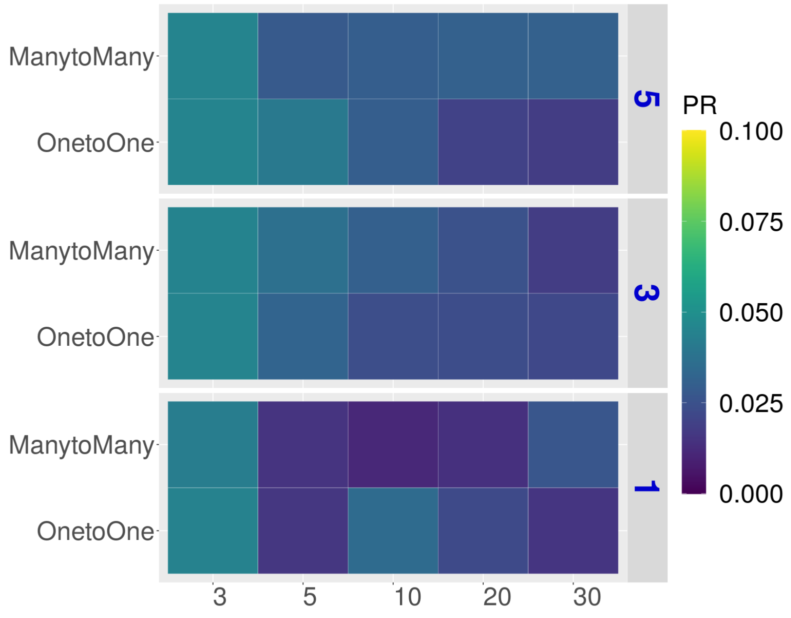

Product Score (P-value)

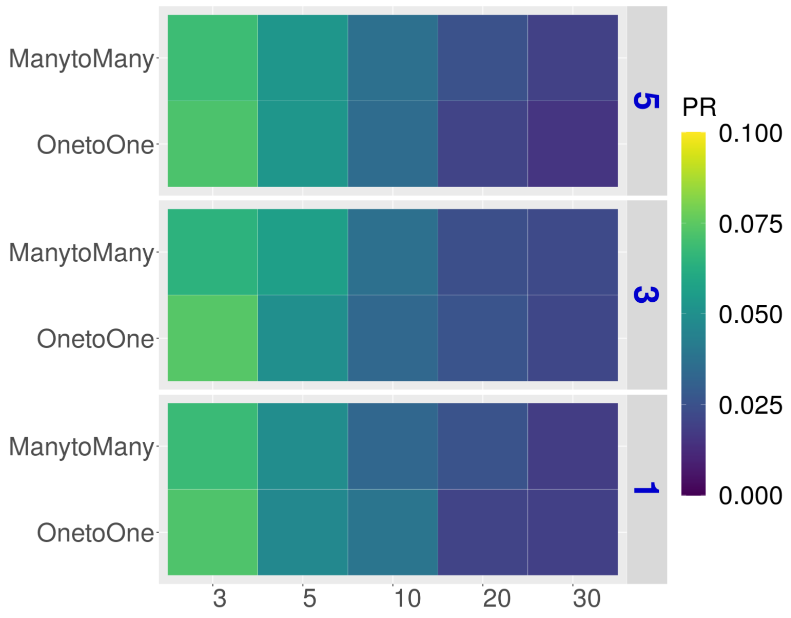

Cabello-Aguilar's Score (P-value)

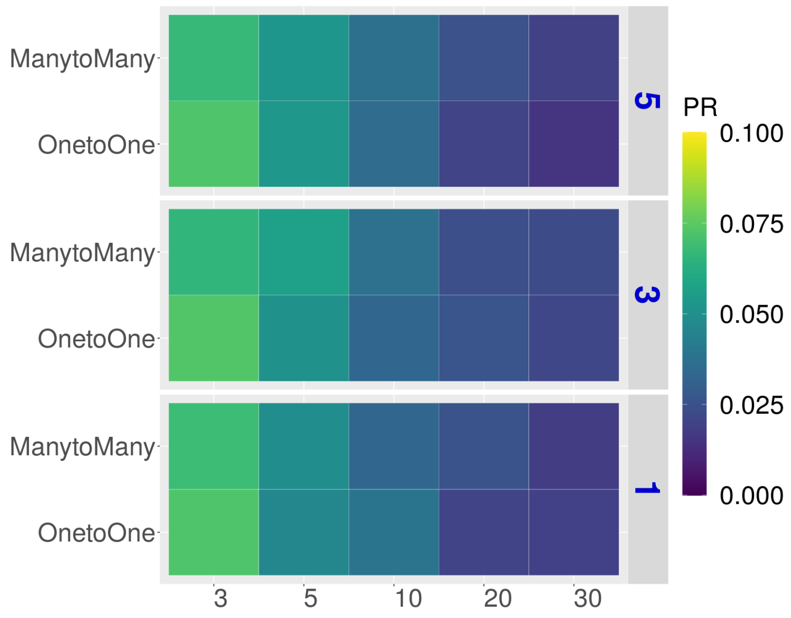

scTensor (NTD-2)

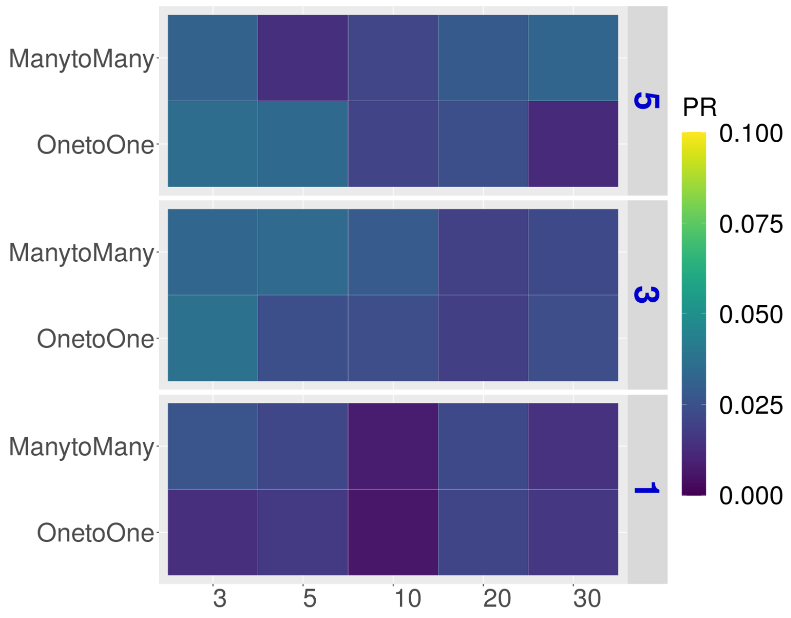

E10 (Details)

Sum Score (P-value)

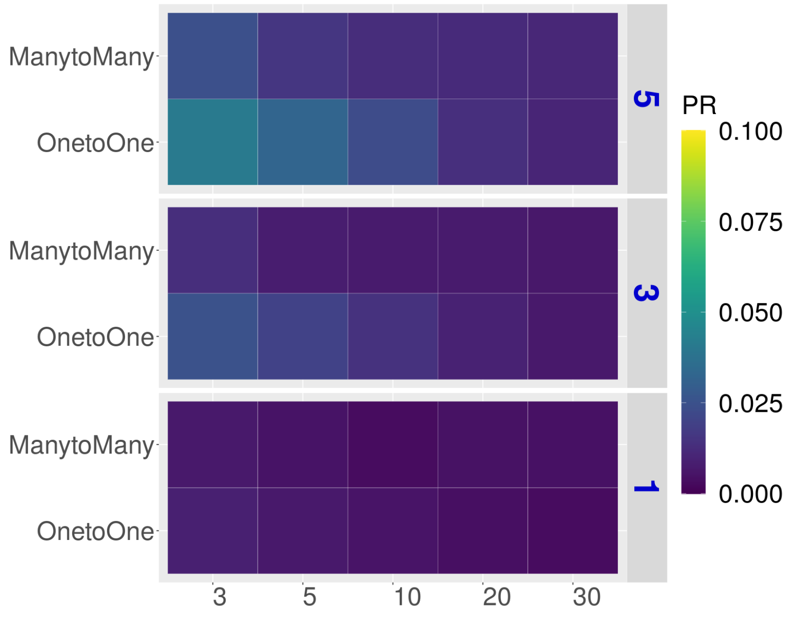

Halpern's Score (P-value)

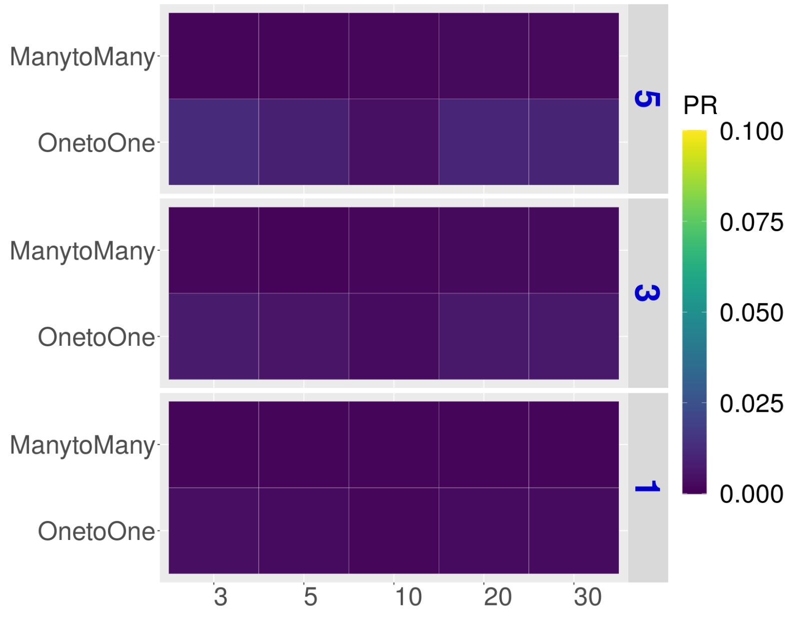

scTensor (NTD-3)

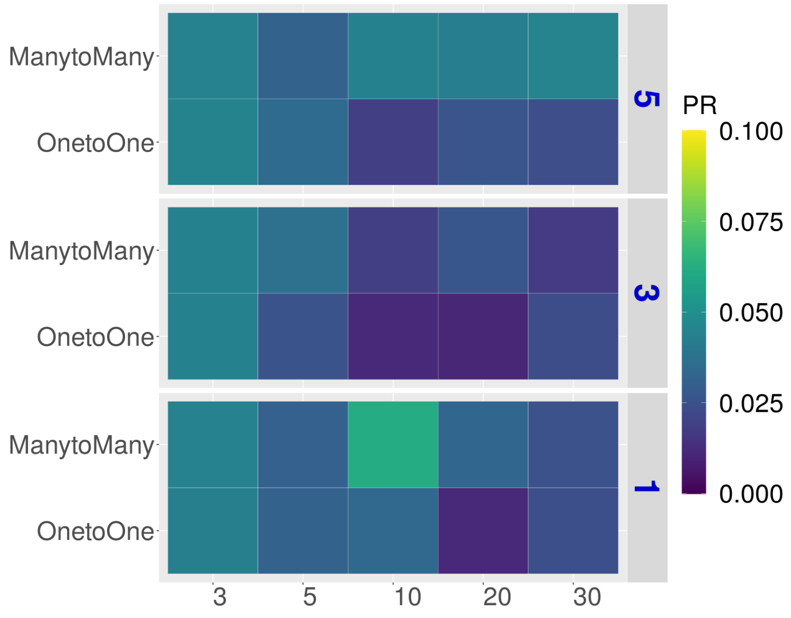

Product Score (P-value)

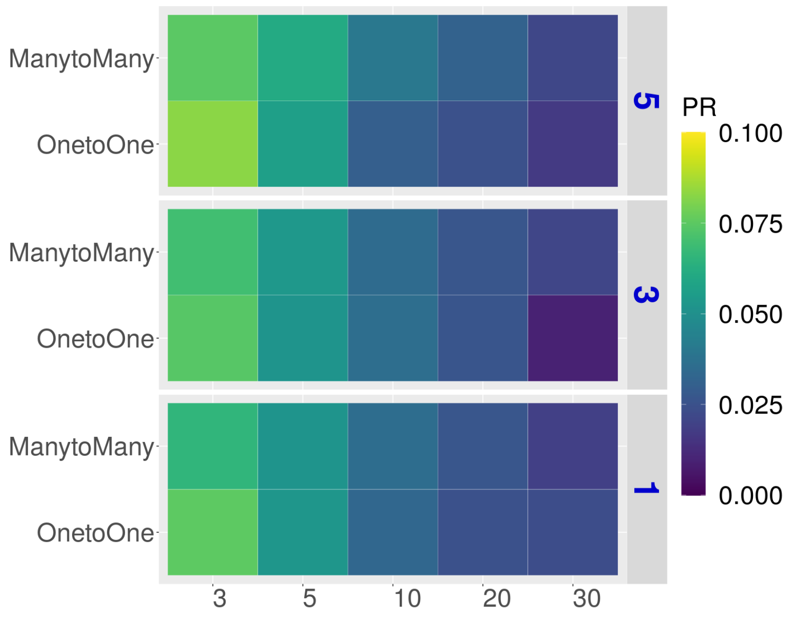

Cabello-Aguilar's Score (P-value)

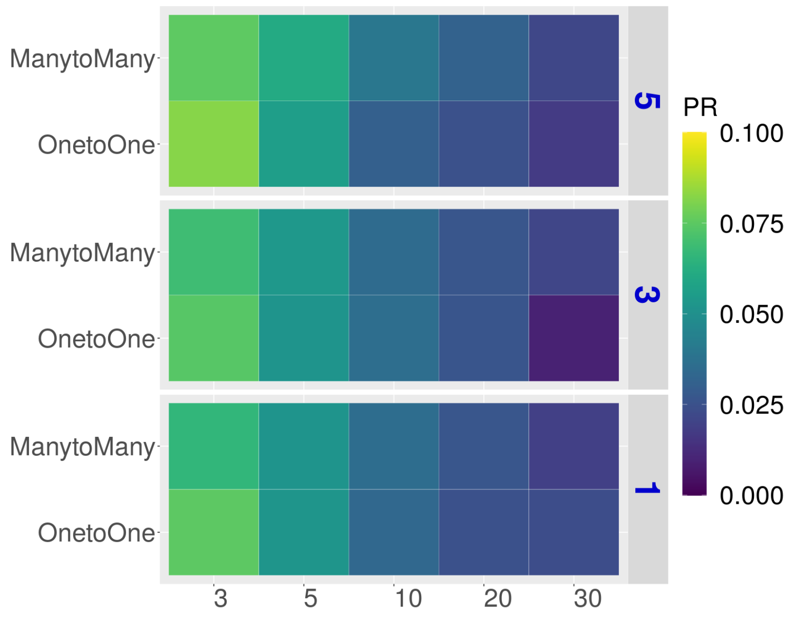

scTensor (NTD-2)

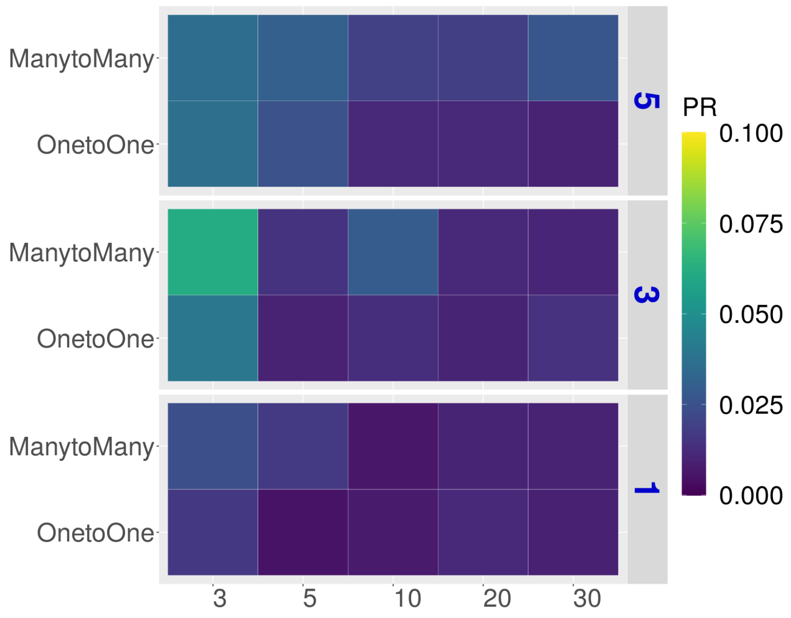

Real Datasets

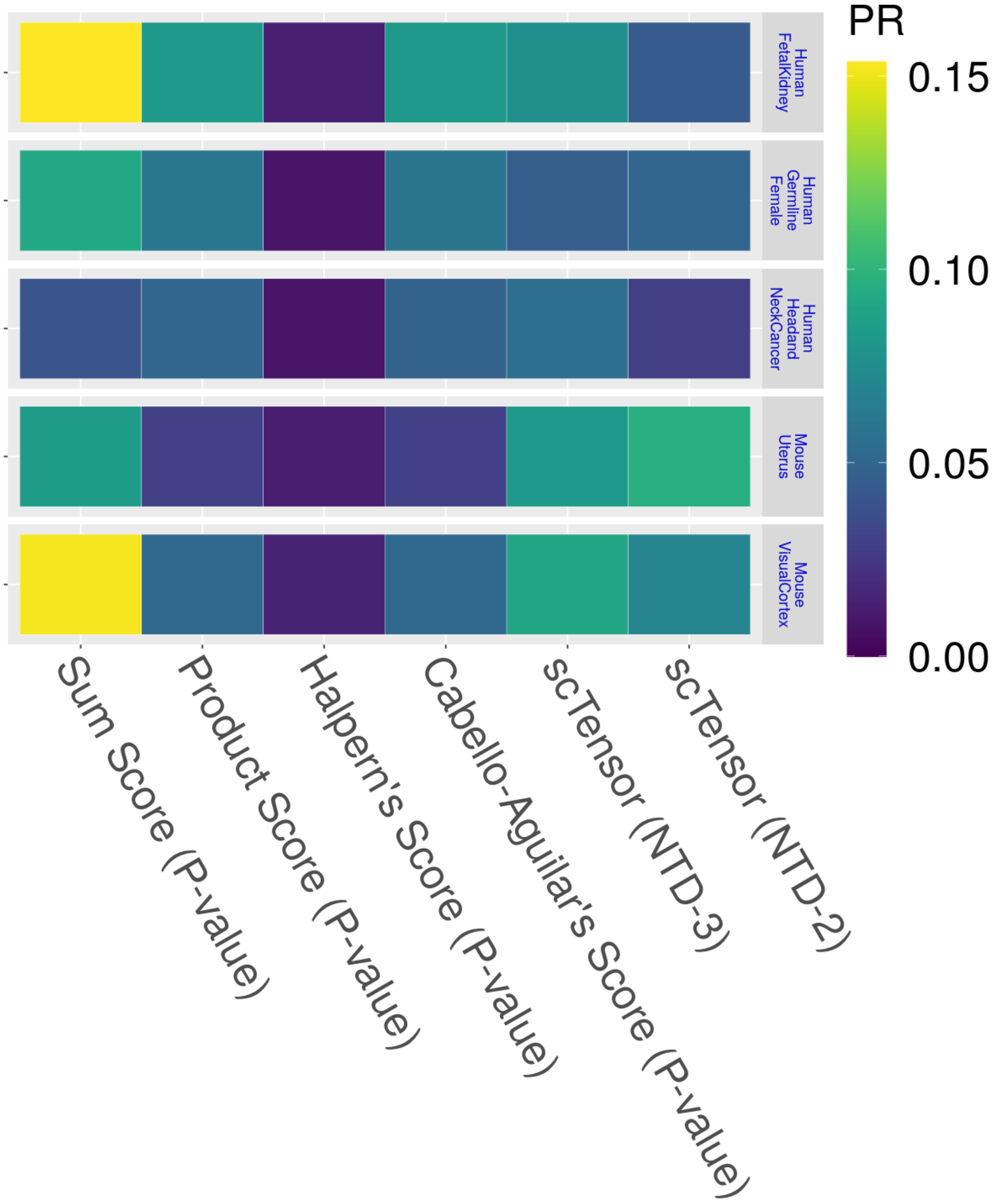

PR
0.15
0.10
0.05
0.00
Human FetalKidney
Human Germline Female
Human Headand NeckCancer
Mouse Uterus
Mouse VisualCortex
Sum Score (P-value)
Product Score (P-value)
Halpern's Score (P-value)
Cabello-Aguilar's Score (P-value)
scTensor (NTD-3)
scTensor (NTD-2)
