# Supplementary material for: Sctensor detects many-to-many cell–cell interactions from single cell RNA-sequencing data
Source: BMC Bioinformatics. 2023 Nov 7;24:420. doi: 10.1186/s12859-023-05490-y (PMC10631077; doi:10.1186/s12859-023-05490-y)
Supplement: Supplementary file 10 — Additional file 10. FPR values of all binarization methods. [file 12859_2023_5490_MOESM10_ESM.pdf]

Simulated Datasets

E2 (Summary) The value ranges 0 to 1 (the closer to 1, the worse)

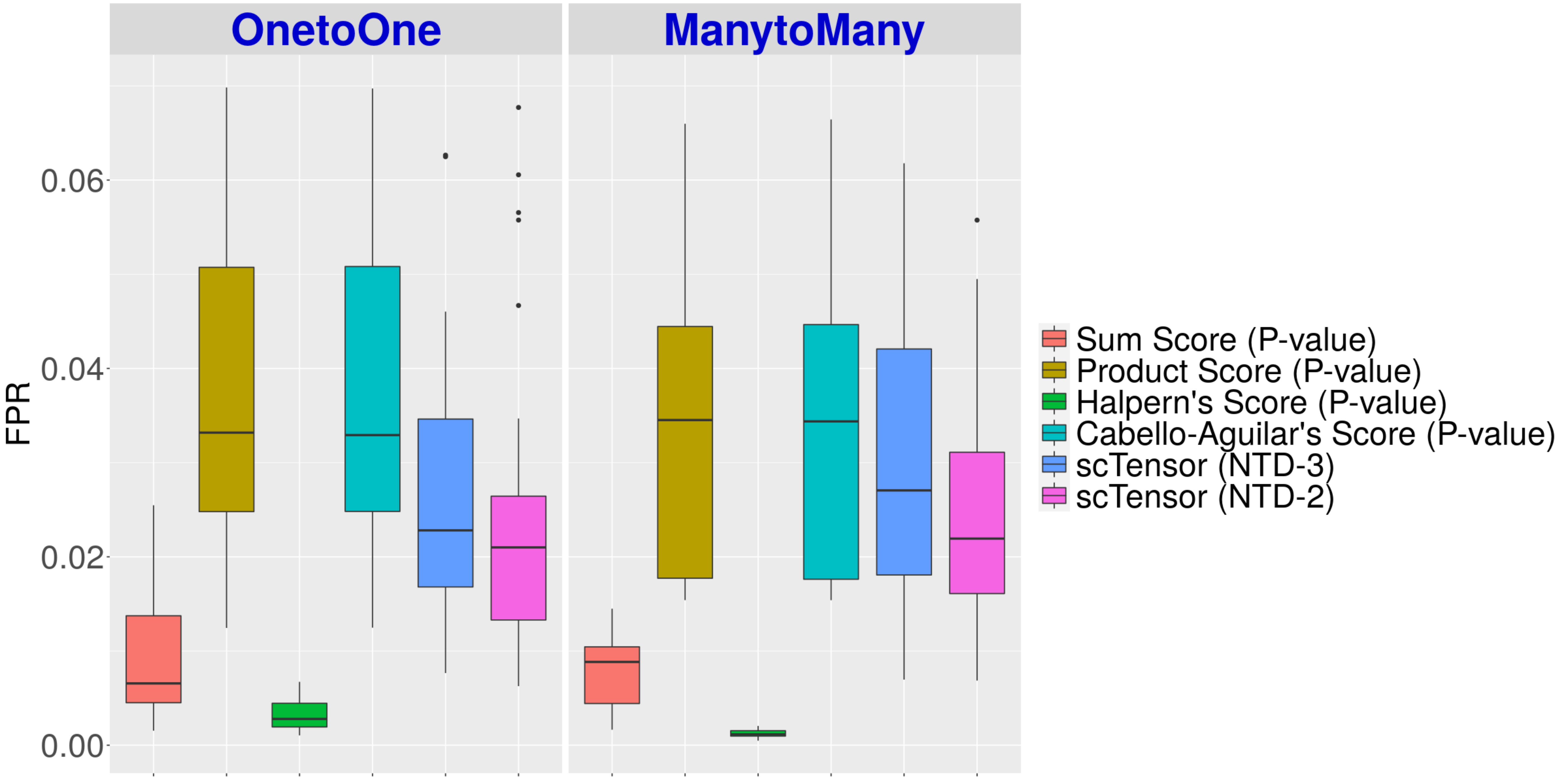

E5 (Summary) The value ranges 0 to 1 (the closer to 1, the worse)

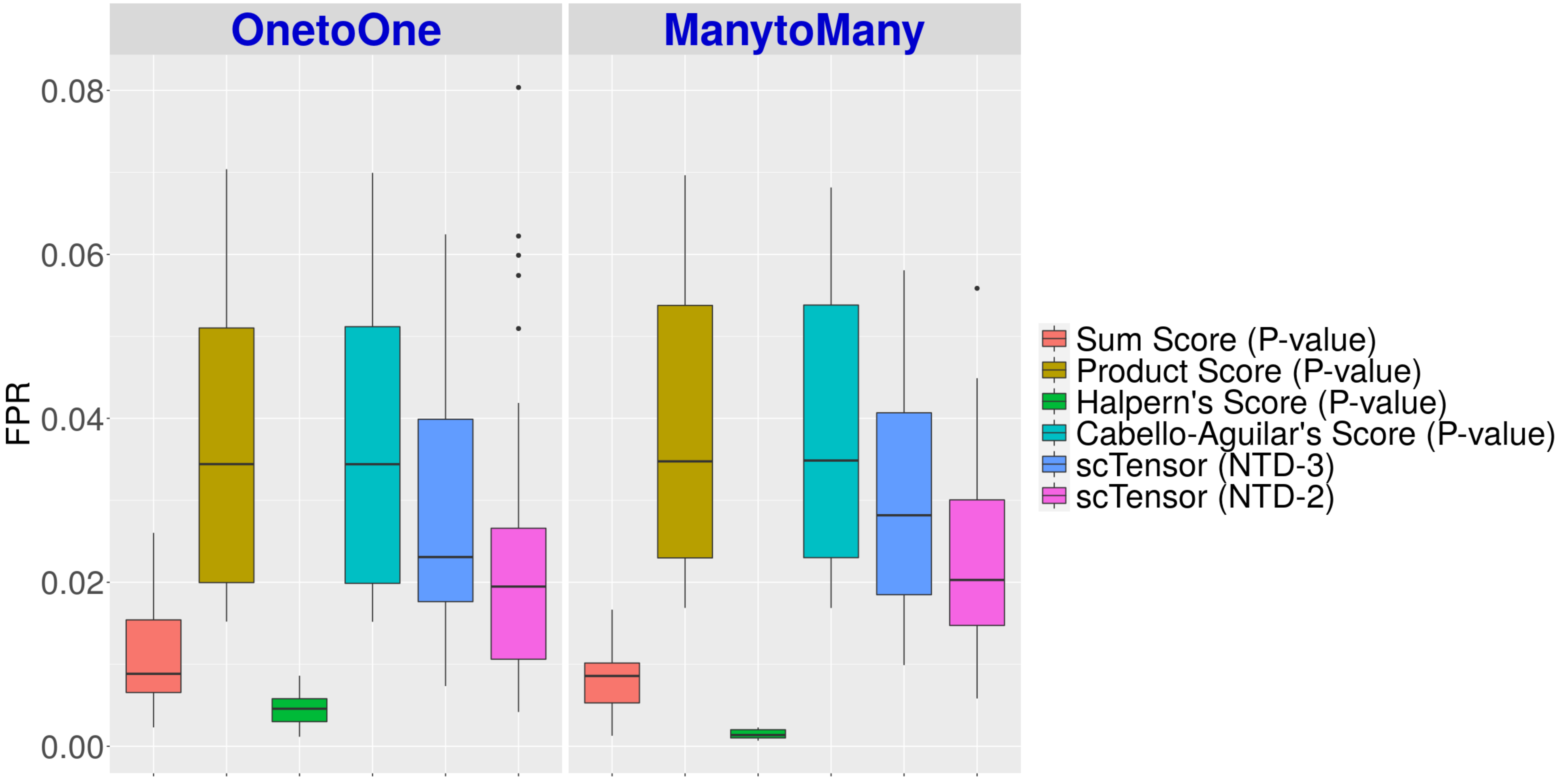

E10 (Summary) The value ranges 0 to 1 (the closer to 1, the worse)

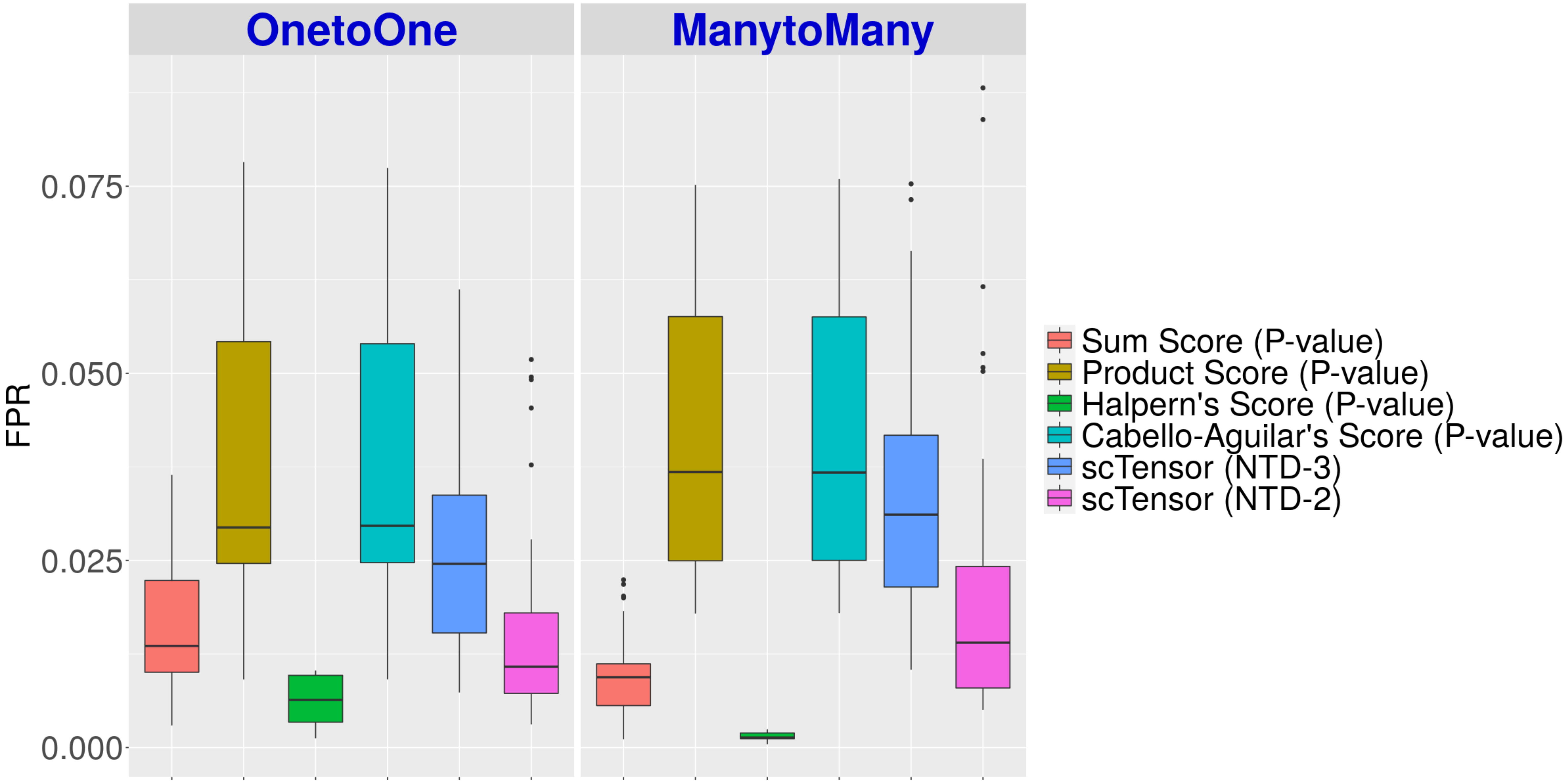

E2 (Details)

The value ranges 0 to 1 (the closer to 1, the worse)

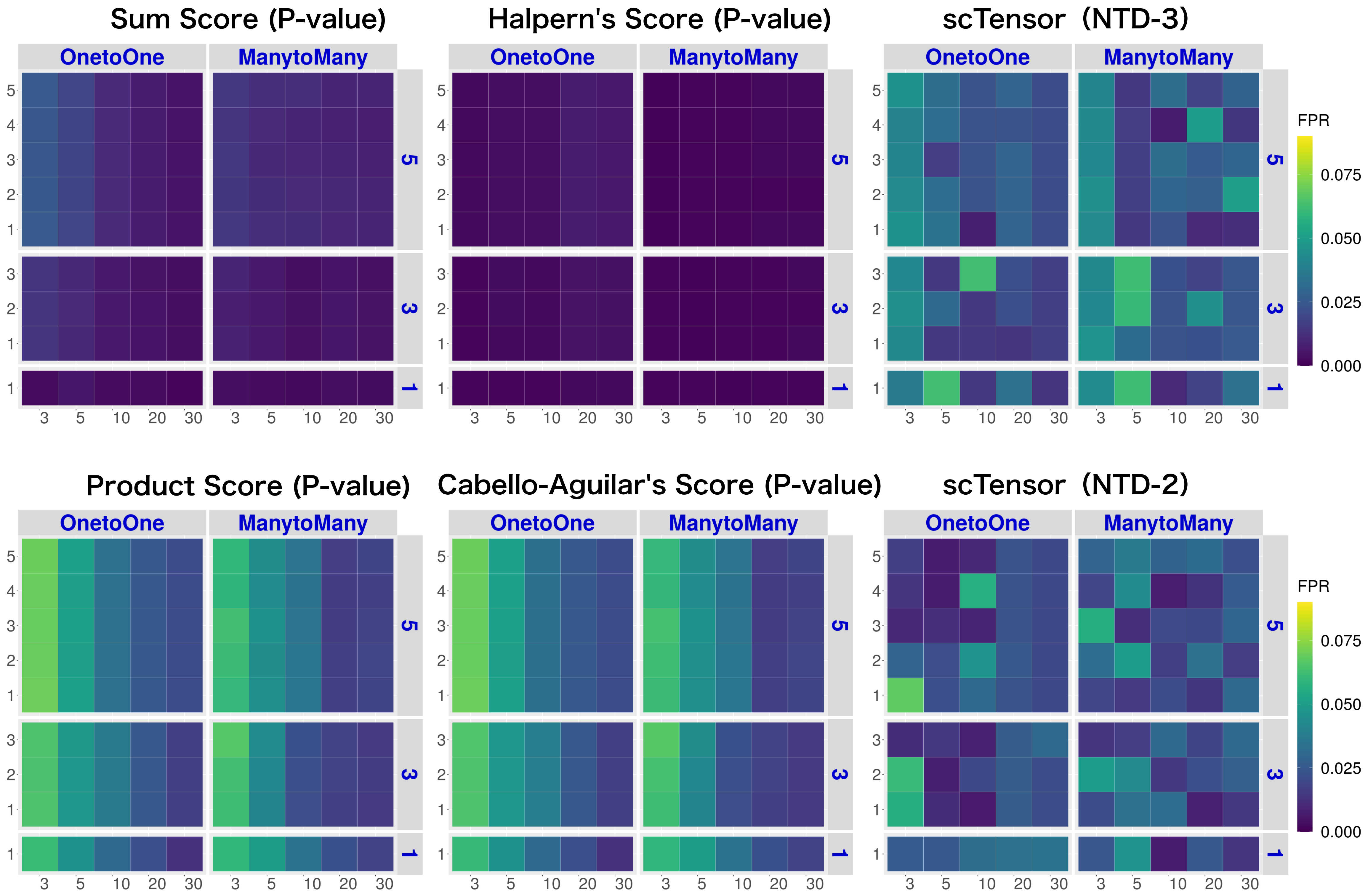

E5 (Details) The value ranges 0 to 1 (the closer to 1, the worse)

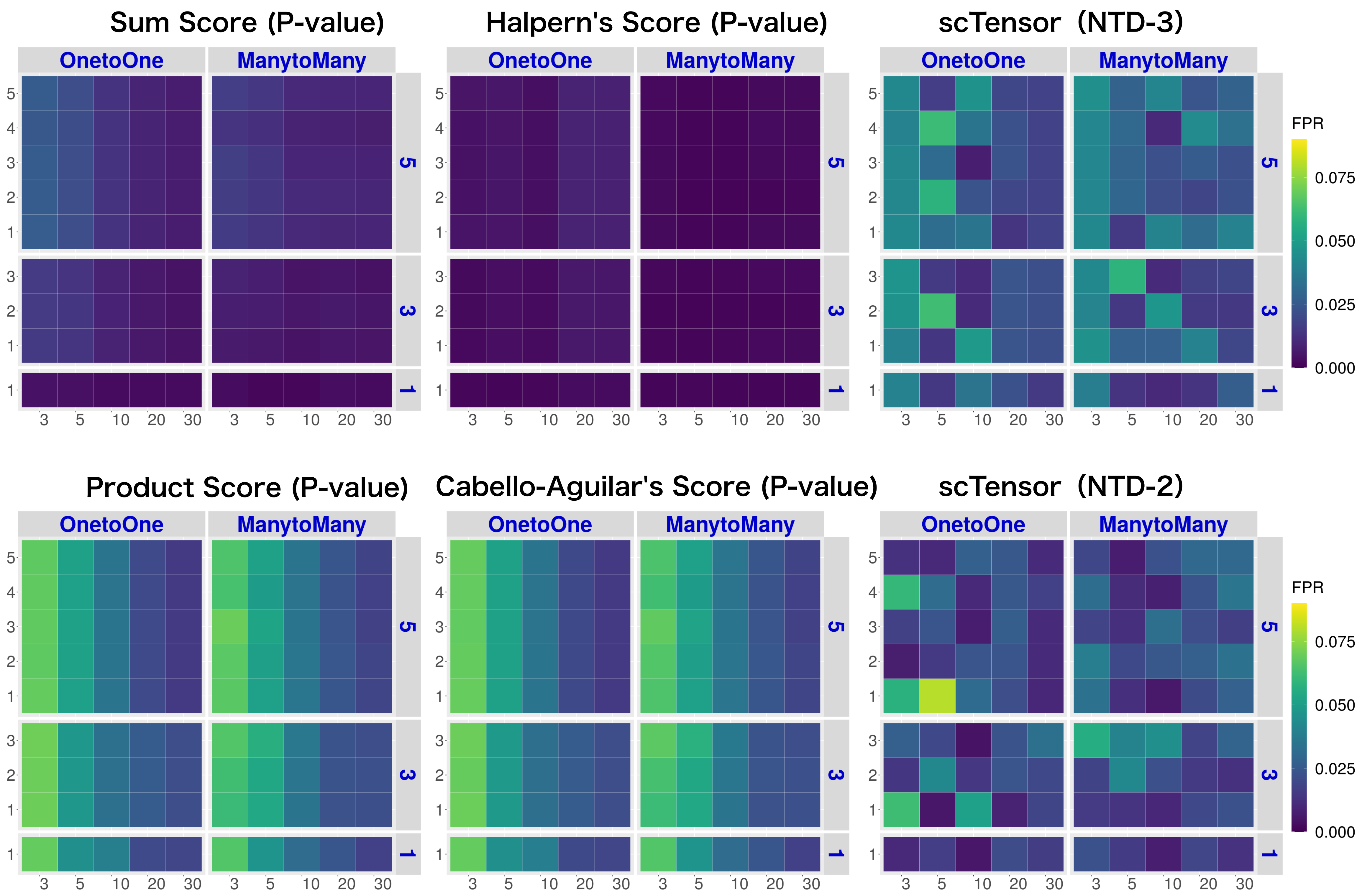

E10 (Details) The value ranges 0 to 1 (the closer to 1, the worse)

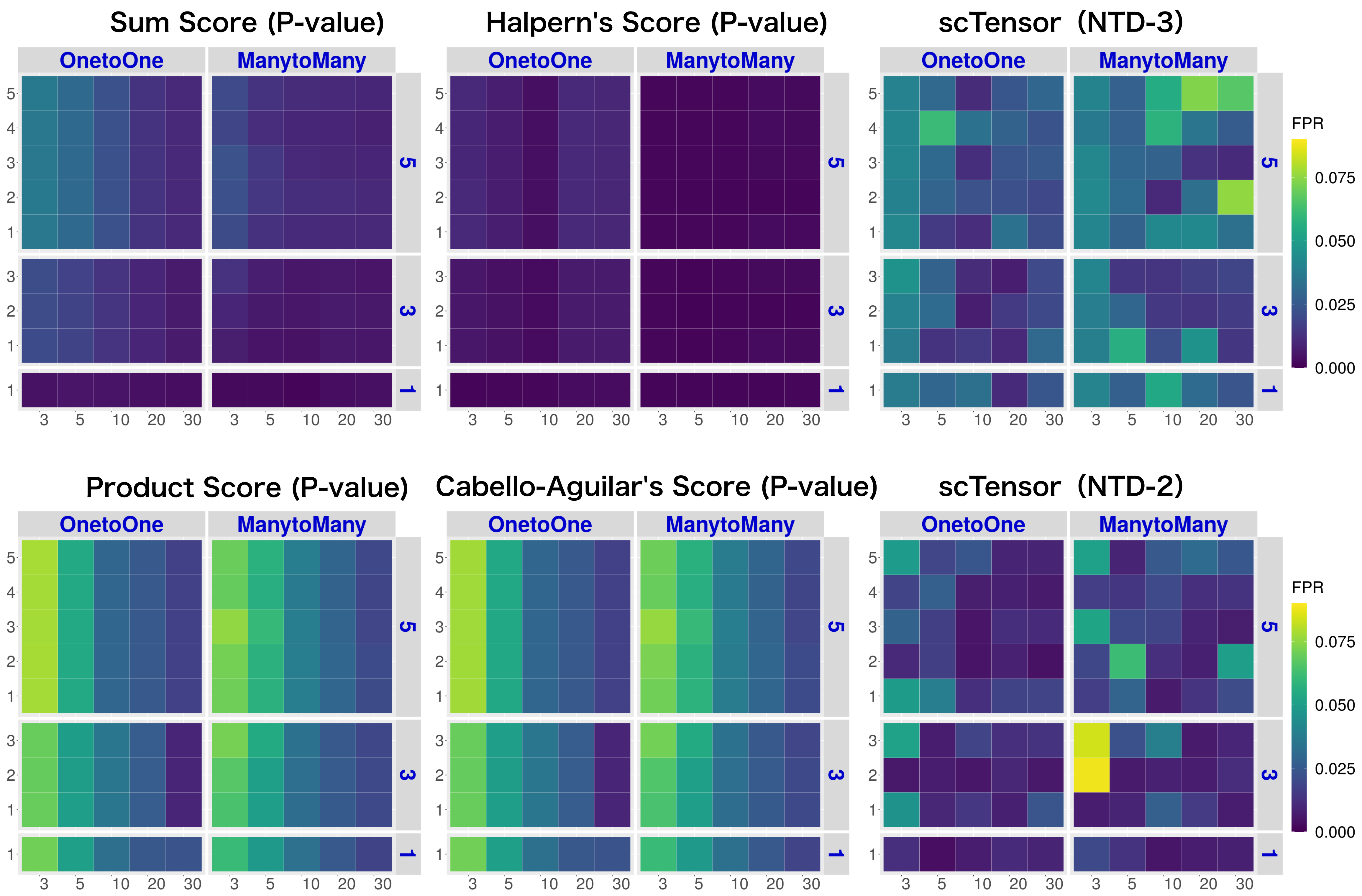

Real Datasets

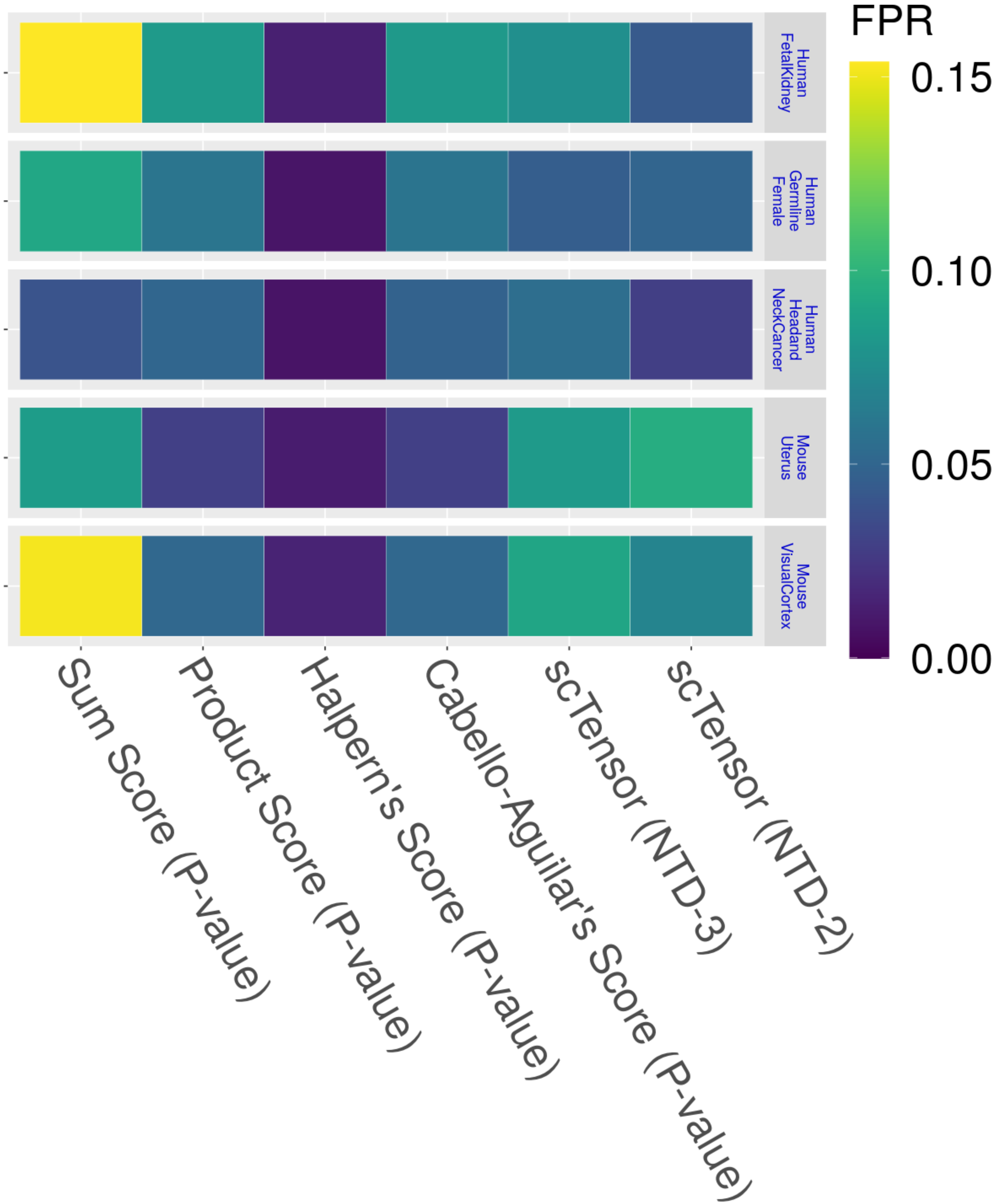

FPR
0.15
0.10
0.05
0.00
Human FetalKidney
Human Germline Female
Human Headand NeckCancer
Mouse Uterus
Mouse VisualCortex
Sum Score (P-value)
Product Score (P-value)
Halpern's Score (P-value)
Cabello-Aguilar's Score (P-value)
scTensor (NTD-3)
scTensor (NTD-2)
