# Supplementary material for: Sctensor detects many-to-many cell–cell interactions from single cell RNA-sequencing data
Source: BMC Bioinformatics. 2023 Nov 7;24:420. doi: 10.1186/s12859-023-05490-y (PMC10631077; doi:10.1186/s12859-023-05490-y)
Supplement: Supplementary file 13 — Additional file 13. Three L-R pairs in which each of the three methods excelled. [file 12859_2023_5490_MOESM13_ESM.docx]

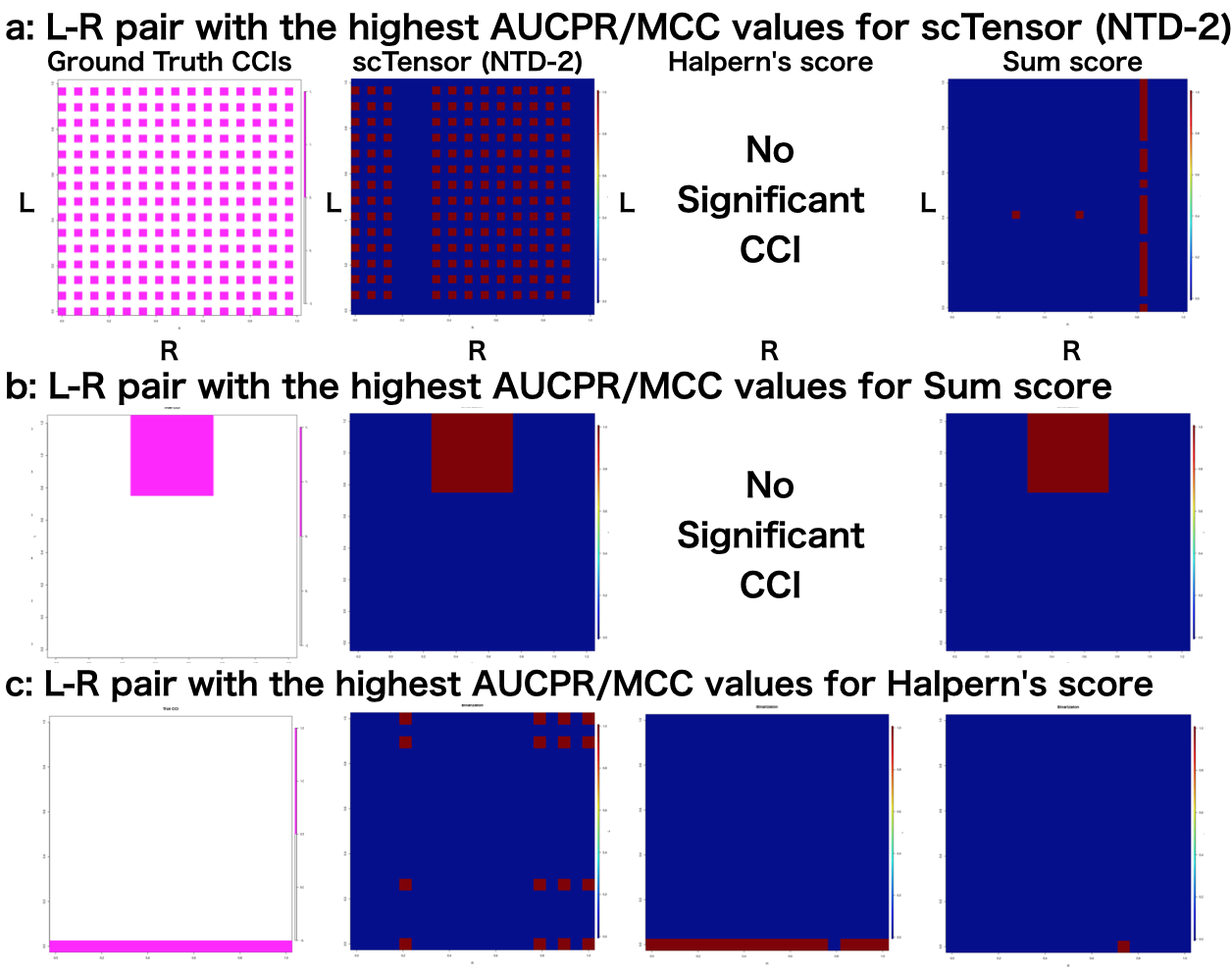


**Fig S1 Three L-R pairs in which each of the three methods excelled** Summary of the number of significant cell-cell interactions (CCIs) with (a) three cell types, one CCI types, one-to-one CCI style, and 1st-CCI type; (b) 20 cell types, five CCI types, many-to-many CCI style, 2nd-CCI type; and (c) 30 cell types, five CCI types, many-to-many CCI style, 5th-CCI type. The y-axis (L) and x-axis (R) indicate the ligand-expressing cell types and the receptor-expressing cell types, respectively.
