# Supplementary figures and images for: Sctensor detects many-to-many cell–cell interactions from single cell RNA-sequencing data (part 11 of 11)
Source: BMC Bioinformatics. 2023 Nov 7;24:420. doi: 10.1186/s12859-023-05490-y (PMC10631077; doi:10.1186/s12859-023-05490-y)

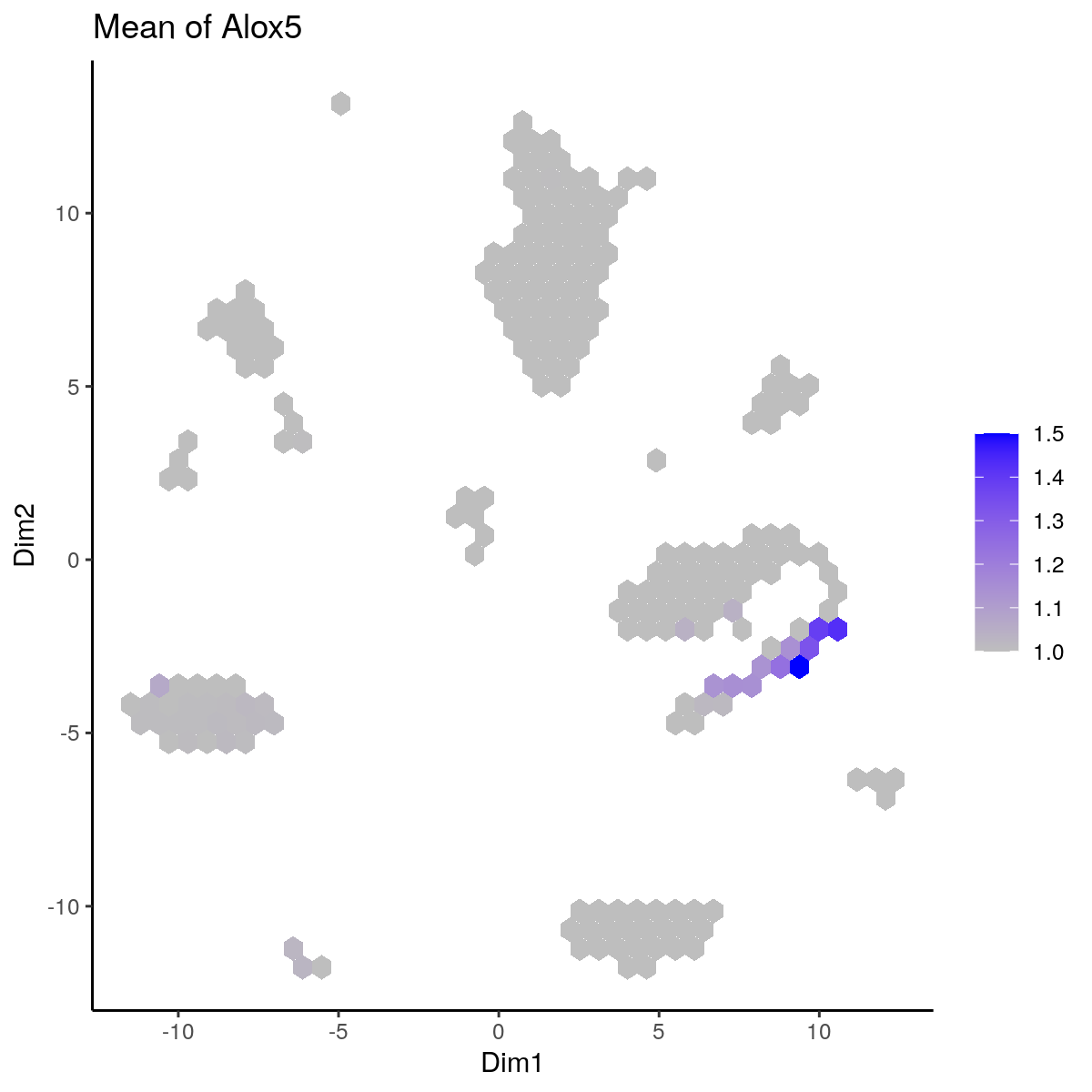

Supplement: Supplementary file 18 — Additional file 18. HTML report of VisualCortex. [file 12859_2023_5490_MOESM18_ESM.zip › output/report/Mouse_VisualCortex/figures/Receptor/11689.png]
