# Supplementary figures and images for: Sctensor detects many-to-many cell–cell interactions from single cell RNA-sequencing data (part 10 of 11)
Source: BMC Bioinformatics. 2023 Nov 7;24:420. doi: 10.1186/s12859-023-05490-y (PMC10631077; doi:10.1186/s12859-023-05490-y)

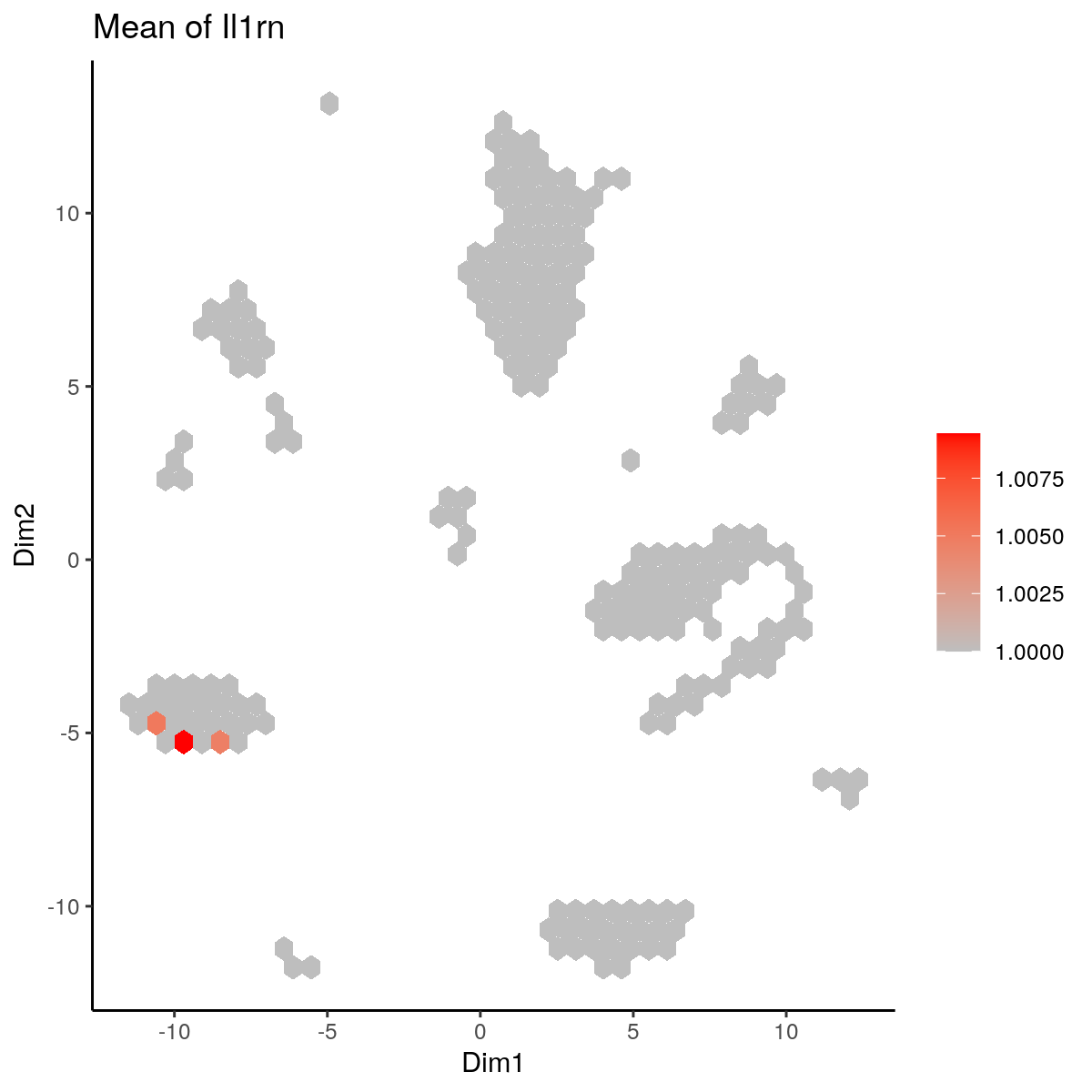

Supplement: Supplementary file 18 — Additional file 18. HTML report of VisualCortex. [file 12859_2023_5490_MOESM18_ESM.zip › output/report/Mouse_VisualCortex/figures/Ligand/16181.png]

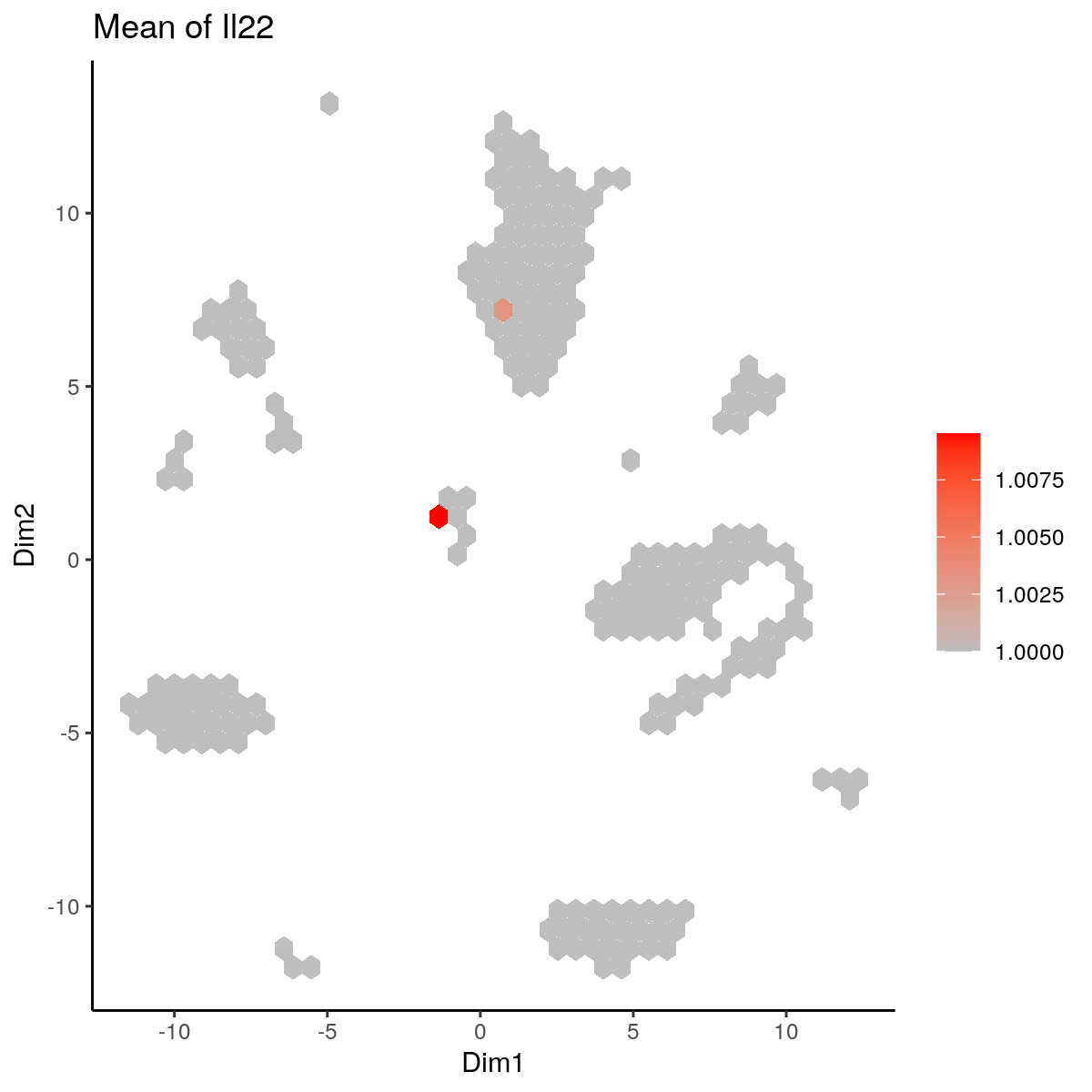

Supplement: Supplementary file 18 — Additional file 18. HTML report of VisualCortex. [file 12859_2023_5490_MOESM18_ESM.zip › output/report/Mouse_VisualCortex/figures/Ligand/50929.png]

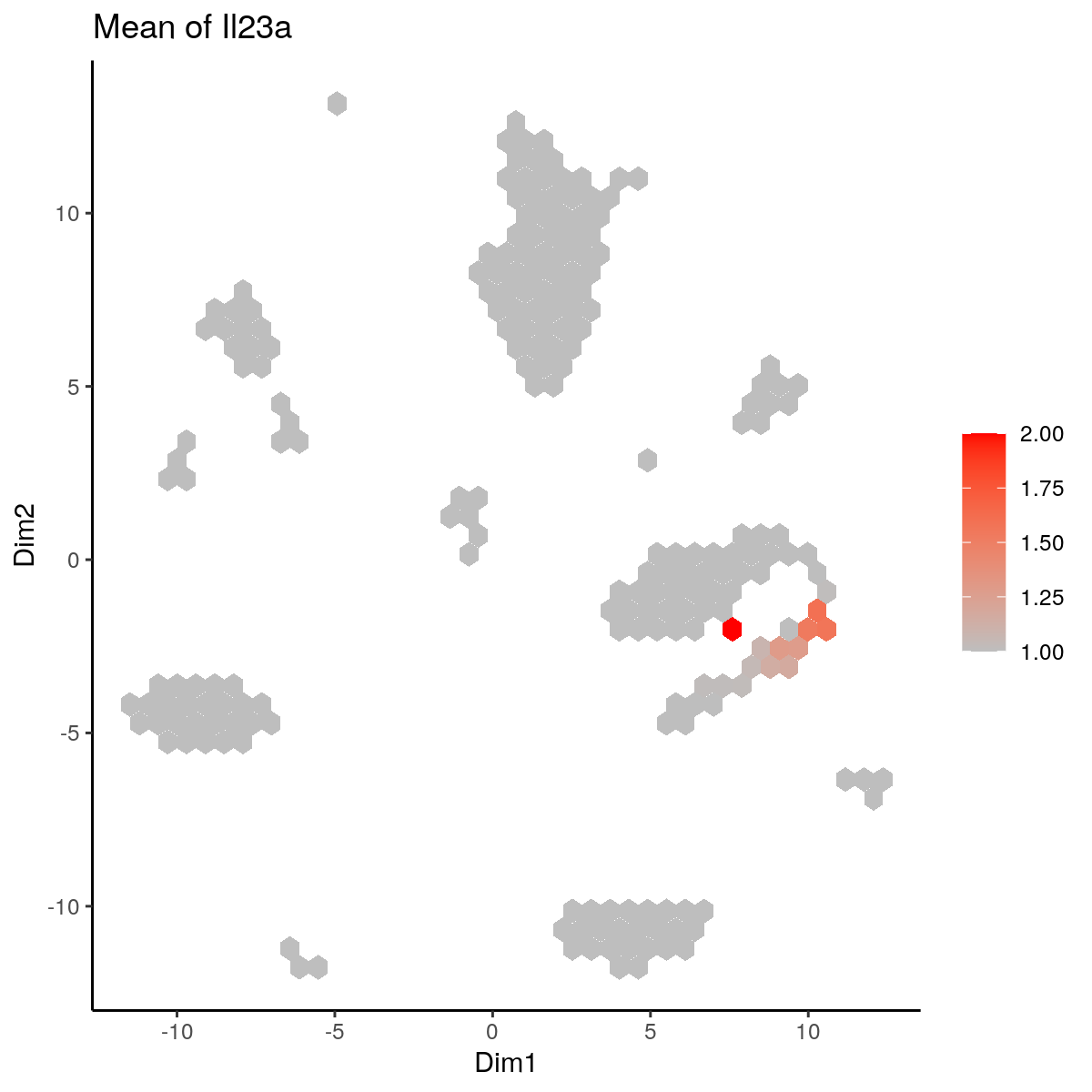

Supplement: Supplementary file 18 — Additional file 18. HTML report of VisualCortex. [file 12859_2023_5490_MOESM18_ESM.zip › output/report/Mouse_VisualCortex/figures/Ligand/83430.png]

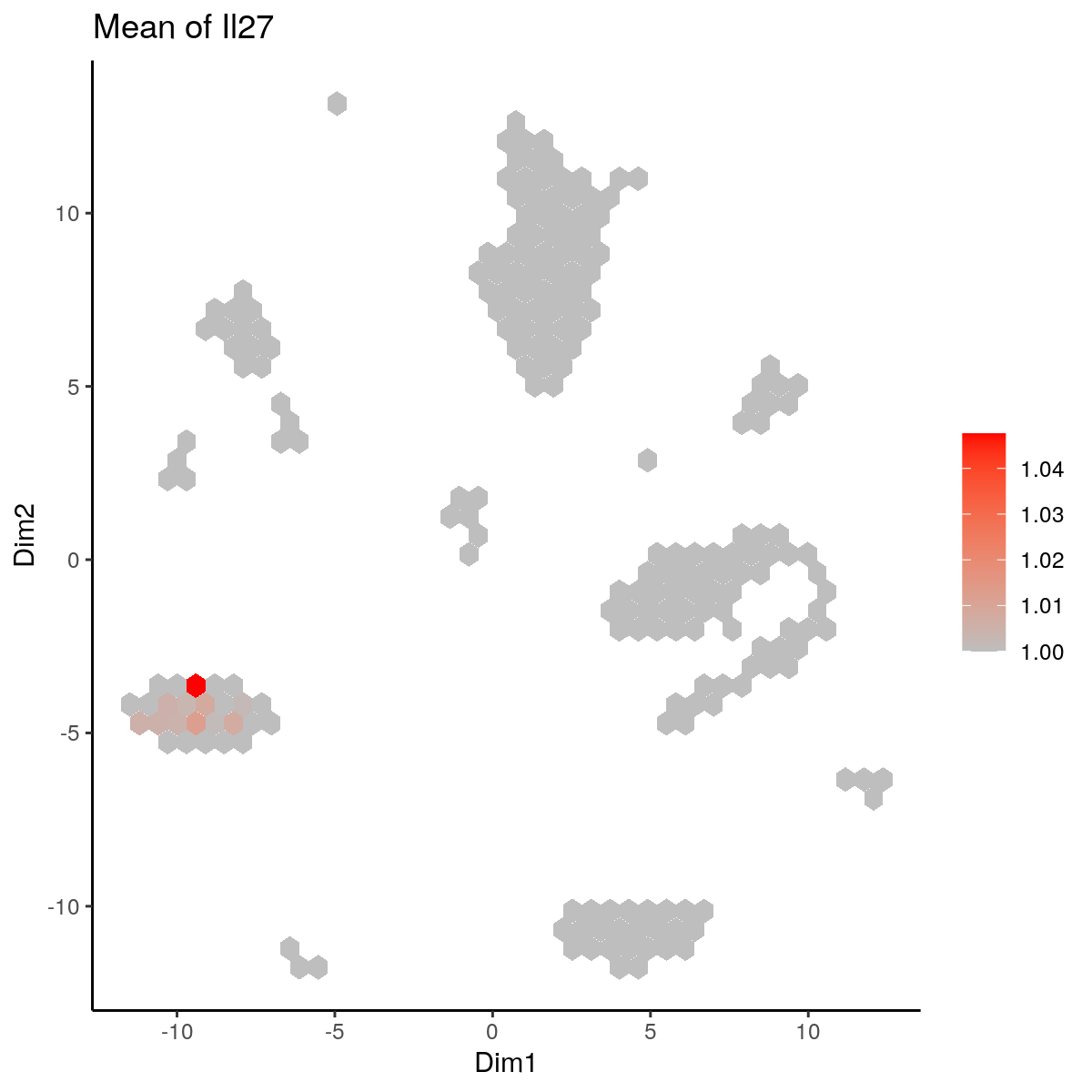

Supplement: Supplementary file 18 — Additional file 18. HTML report of VisualCortex. [file 12859_2023_5490_MOESM18_ESM.zip › output/report/Mouse_VisualCortex/figures/Ligand/246779.png]

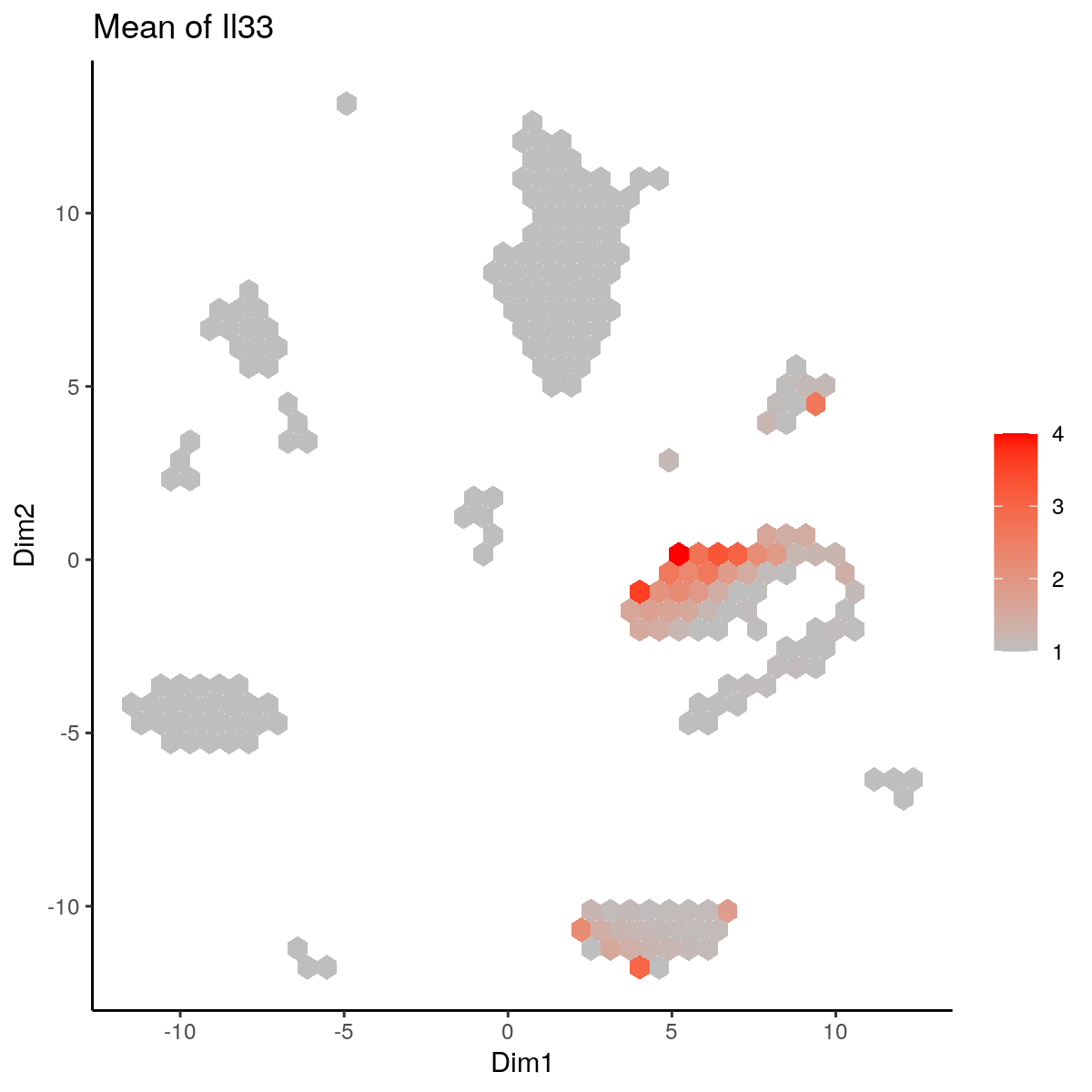

Supplement: Supplementary file 18 — Additional file 18. HTML report of VisualCortex. [file 12859_2023_5490_MOESM18_ESM.zip › output/report/Mouse_VisualCortex/figures/Ligand/77125.png]

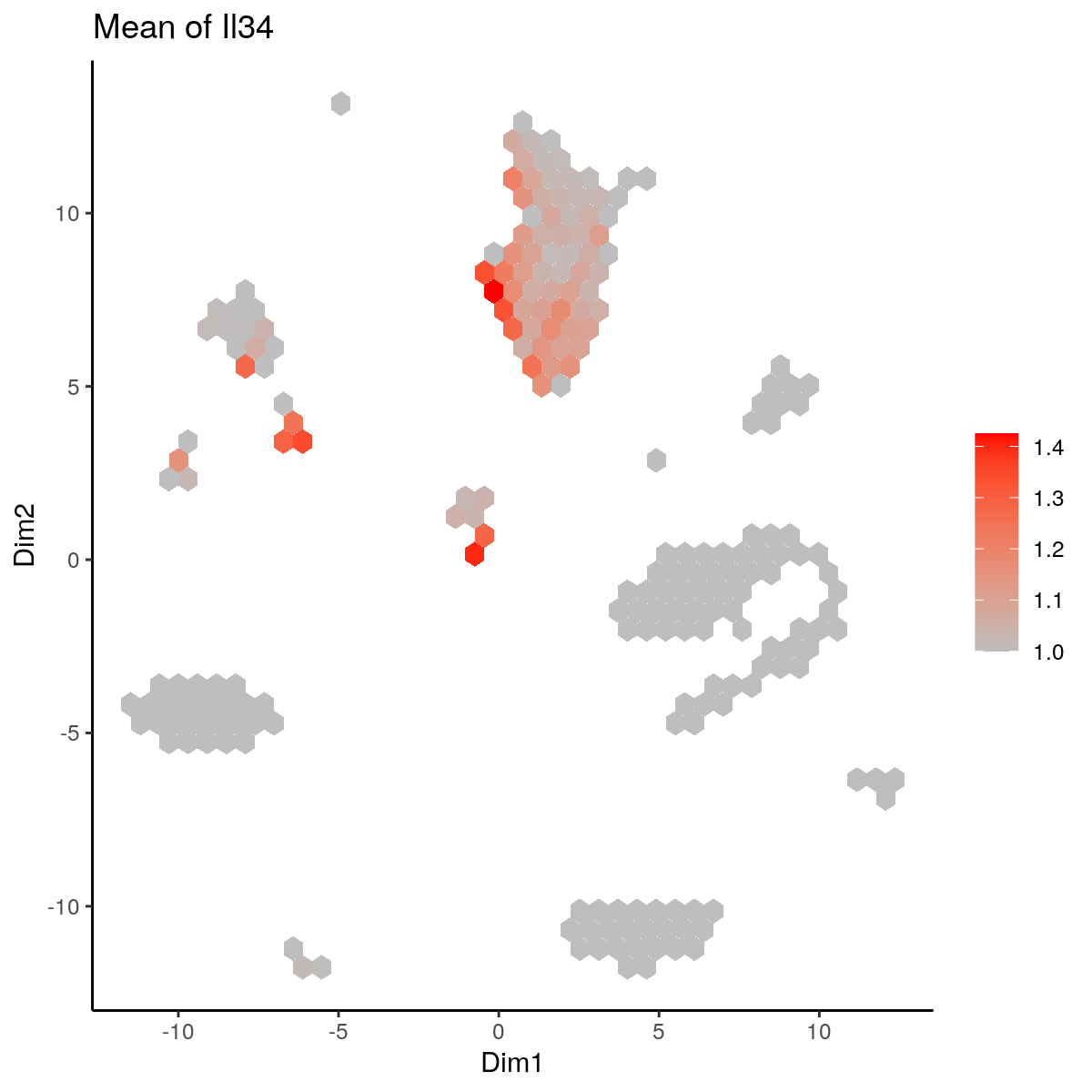

Supplement: Supplementary file 18 — Additional file 18. HTML report of VisualCortex. [file 12859_2023_5490_MOESM18_ESM.zip › output/report/Mouse_VisualCortex/figures/Ligand/76527.png]

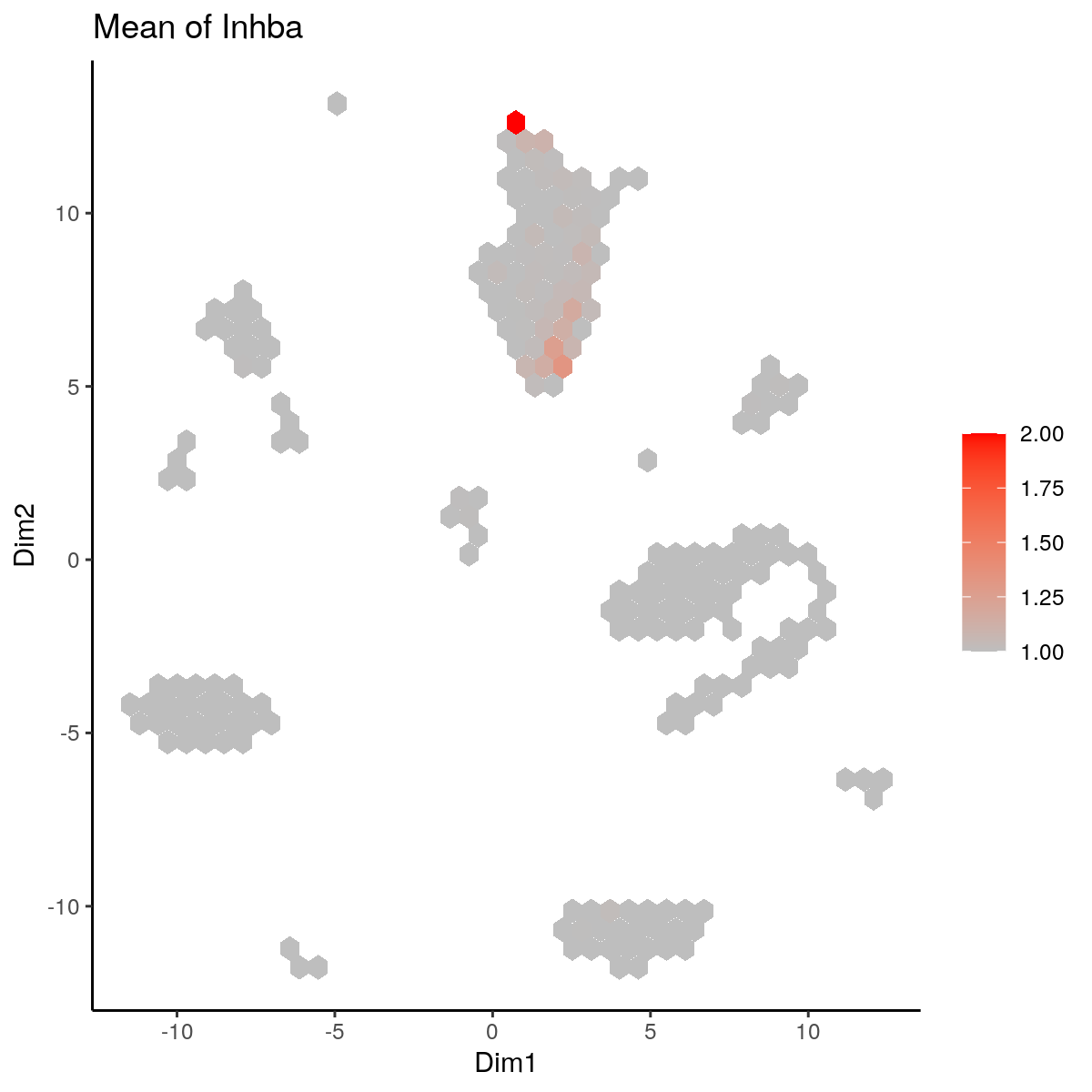

Supplement: Supplementary file 18 — Additional file 18. HTML report of VisualCortex. [file 12859_2023_5490_MOESM18_ESM.zip › output/report/Mouse_VisualCortex/figures/Ligand/16323.png]

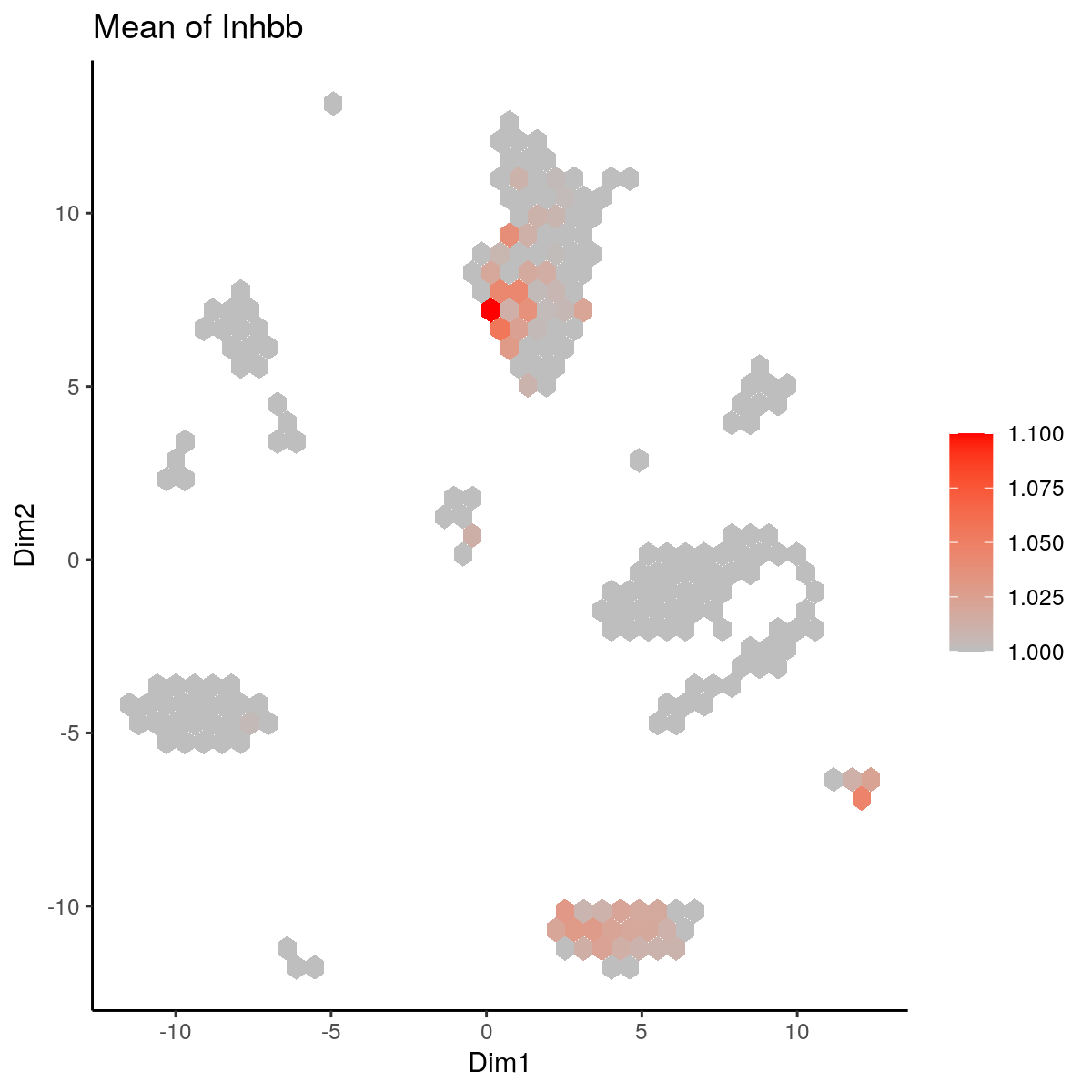

Supplement: Supplementary file 18 — Additional file 18. HTML report of VisualCortex. [file 12859_2023_5490_MOESM18_ESM.zip › output/report/Mouse_VisualCortex/figures/Ligand/16324.png]

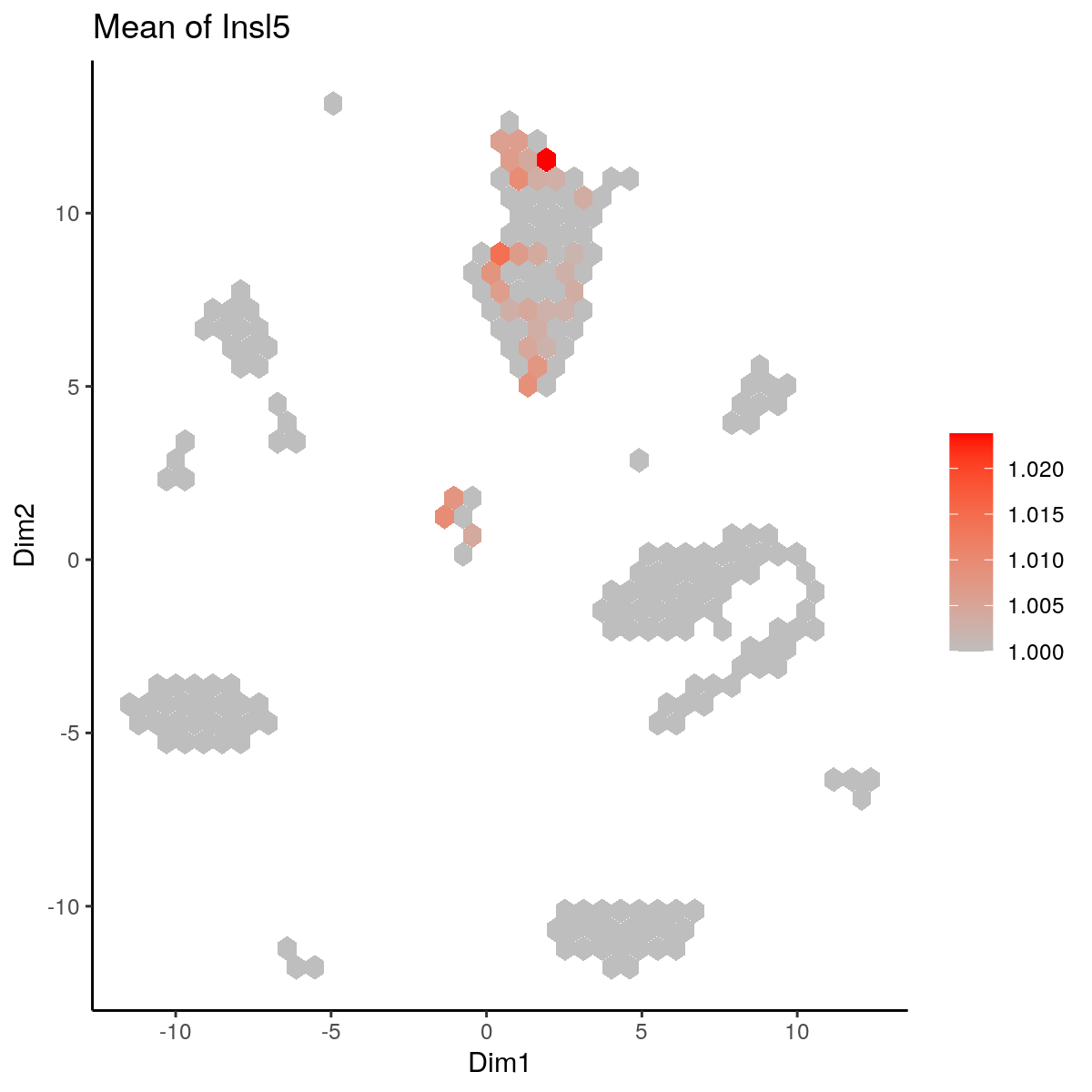

Supplement: Supplementary file 18 — Additional file 18. HTML report of VisualCortex. [file 12859_2023_5490_MOESM18_ESM.zip › output/report/Mouse_VisualCortex/figures/Ligand/23919.png]

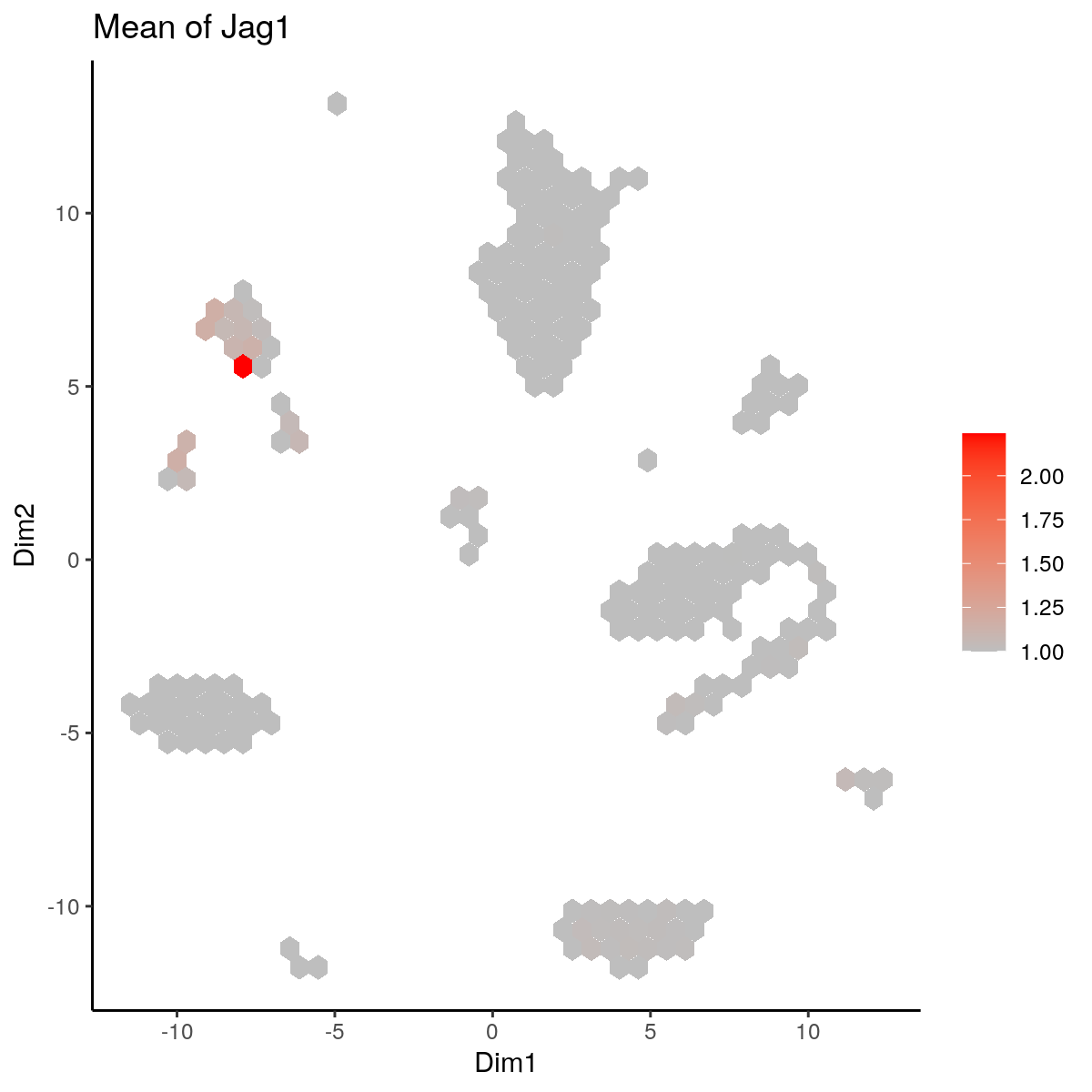

Supplement: Supplementary file 18 — Additional file 18. HTML report of VisualCortex. [file 12859_2023_5490_MOESM18_ESM.zip › output/report/Mouse_VisualCortex/figures/Ligand/16449.png]

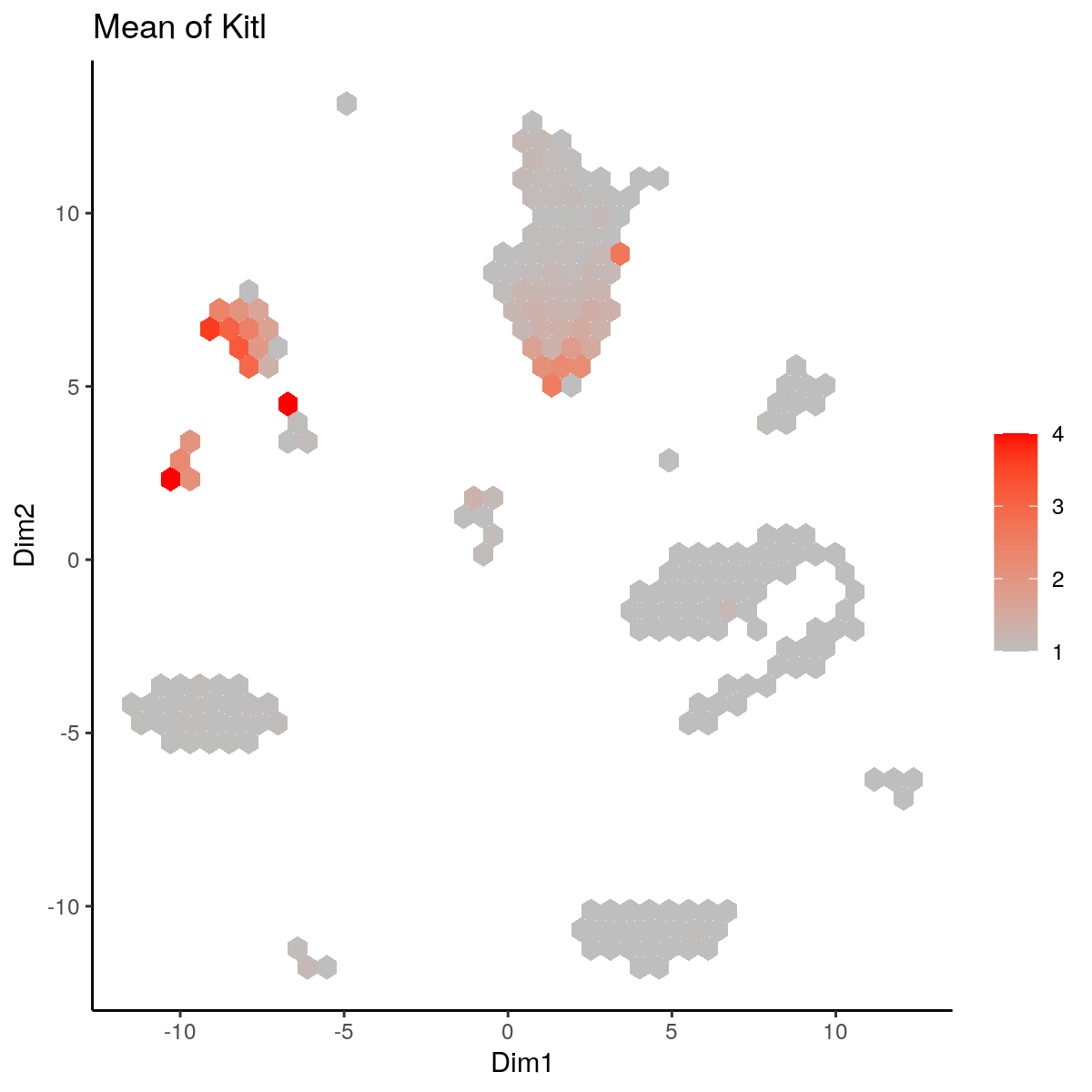

Supplement: Supplementary file 18 — Additional file 18. HTML report of VisualCortex. [file 12859_2023_5490_MOESM18_ESM.zip › output/report/Mouse_VisualCortex/figures/Ligand/17311.png]

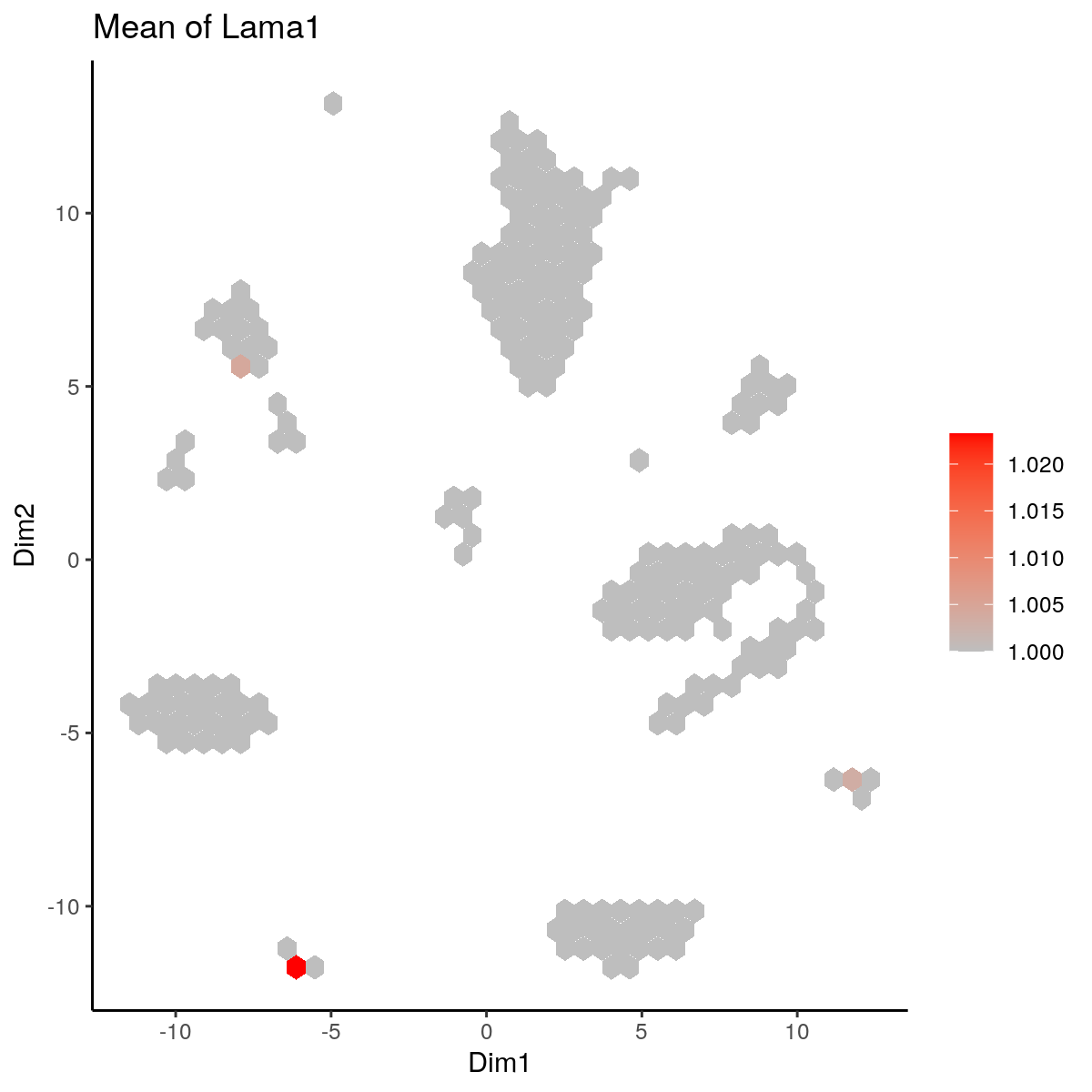

Supplement: Supplementary file 18 — Additional file 18. HTML report of VisualCortex. [file 12859_2023_5490_MOESM18_ESM.zip › output/report/Mouse_VisualCortex/figures/Ligand/16772.png]

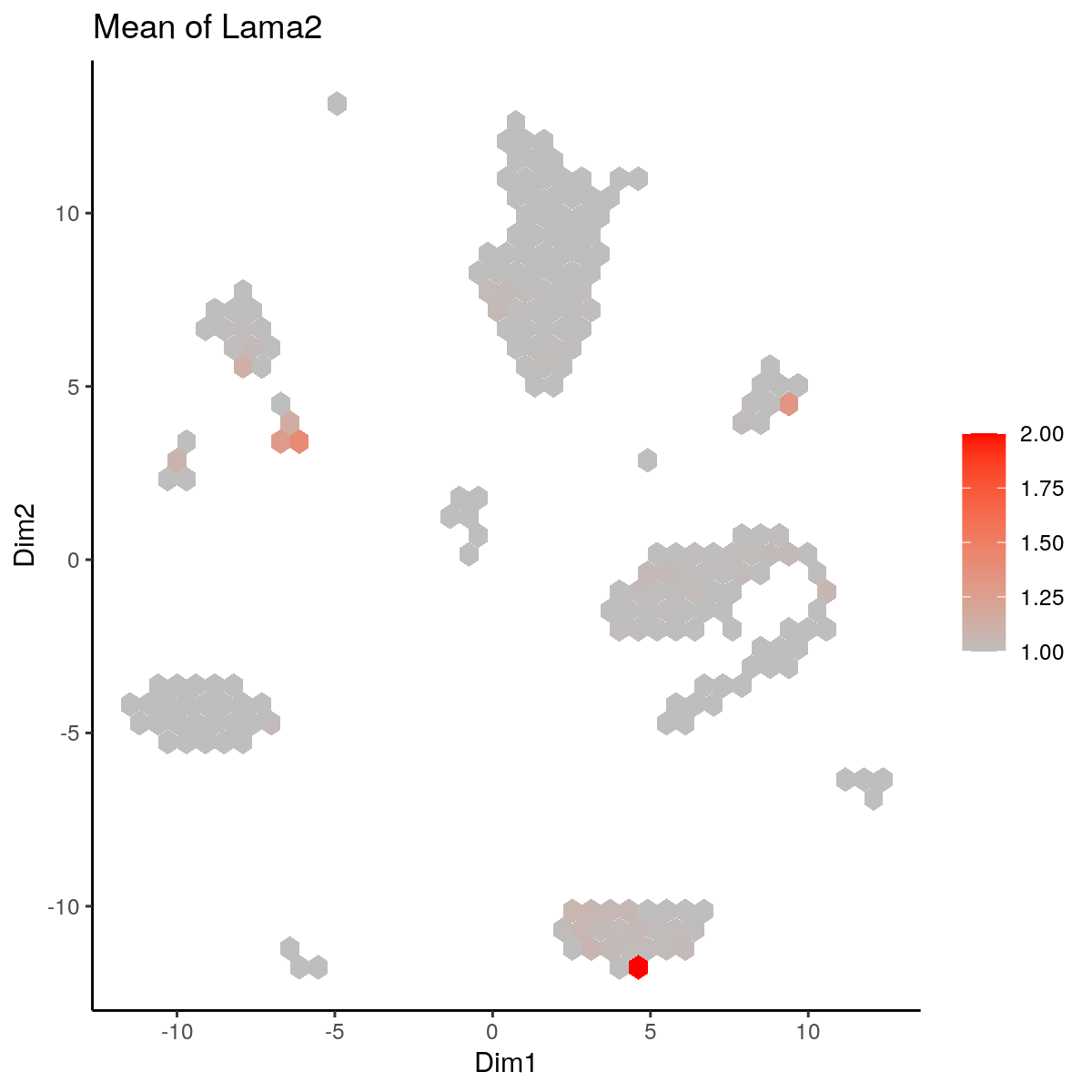

Supplement: Supplementary file 18 — Additional file 18. HTML report of VisualCortex. [file 12859_2023_5490_MOESM18_ESM.zip › output/report/Mouse_VisualCortex/figures/Ligand/16773.png]

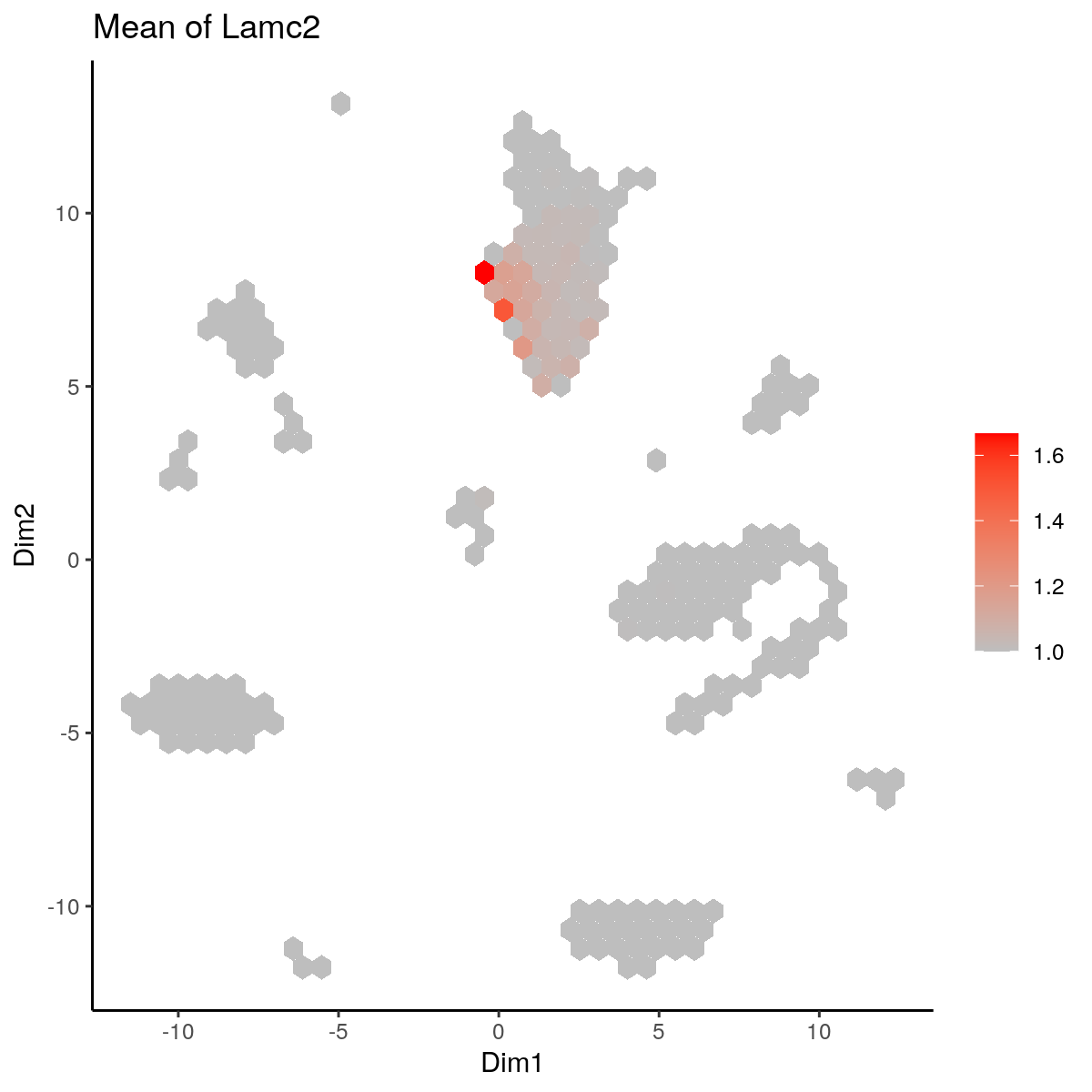

Supplement: Supplementary file 18 — Additional file 18. HTML report of VisualCortex. [file 12859_2023_5490_MOESM18_ESM.zip › output/report/Mouse_VisualCortex/figures/Ligand/16782.png]

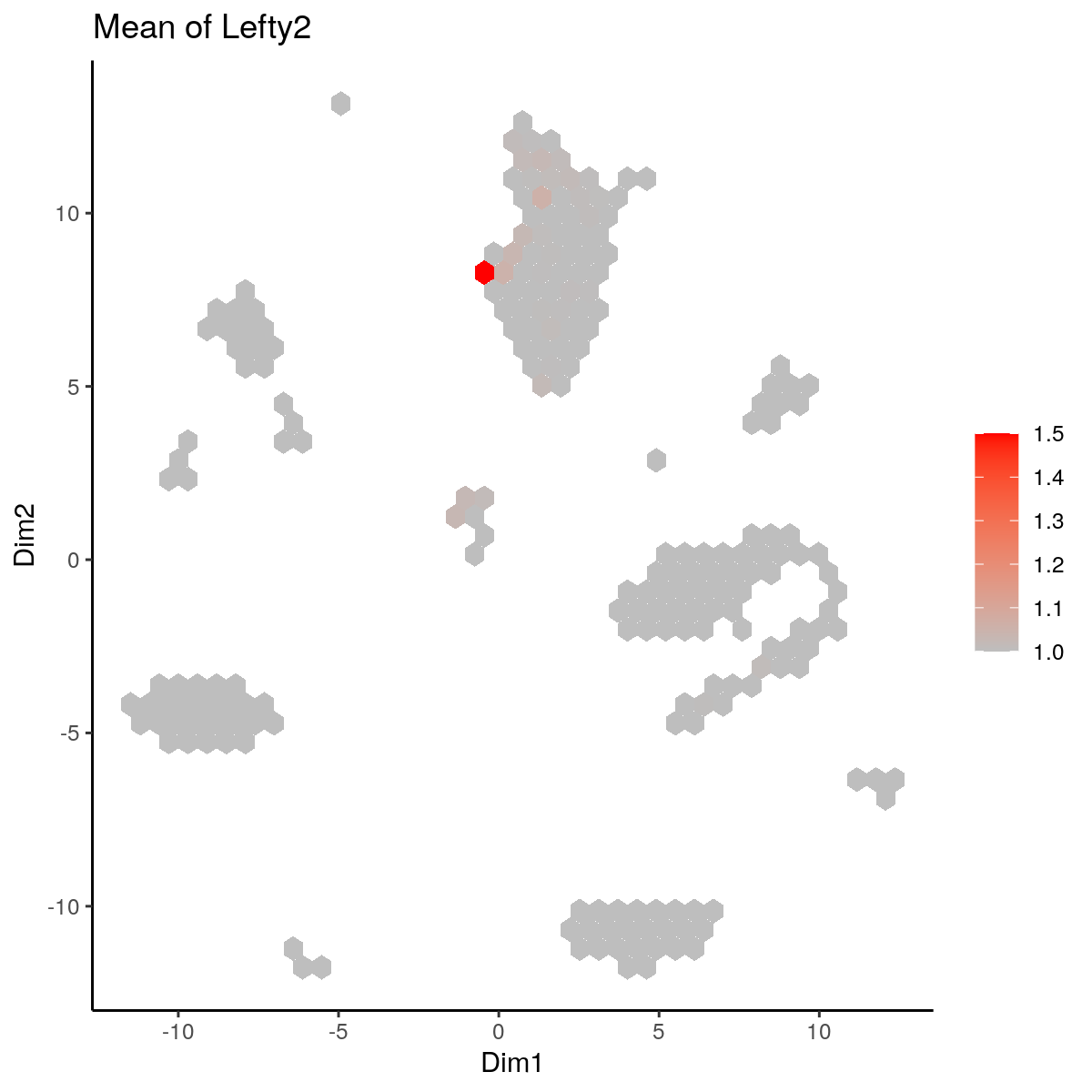

Supplement: Supplementary file 18 — Additional file 18. HTML report of VisualCortex. [file 12859_2023_5490_MOESM18_ESM.zip › output/report/Mouse_VisualCortex/figures/Ligand/320202.png]

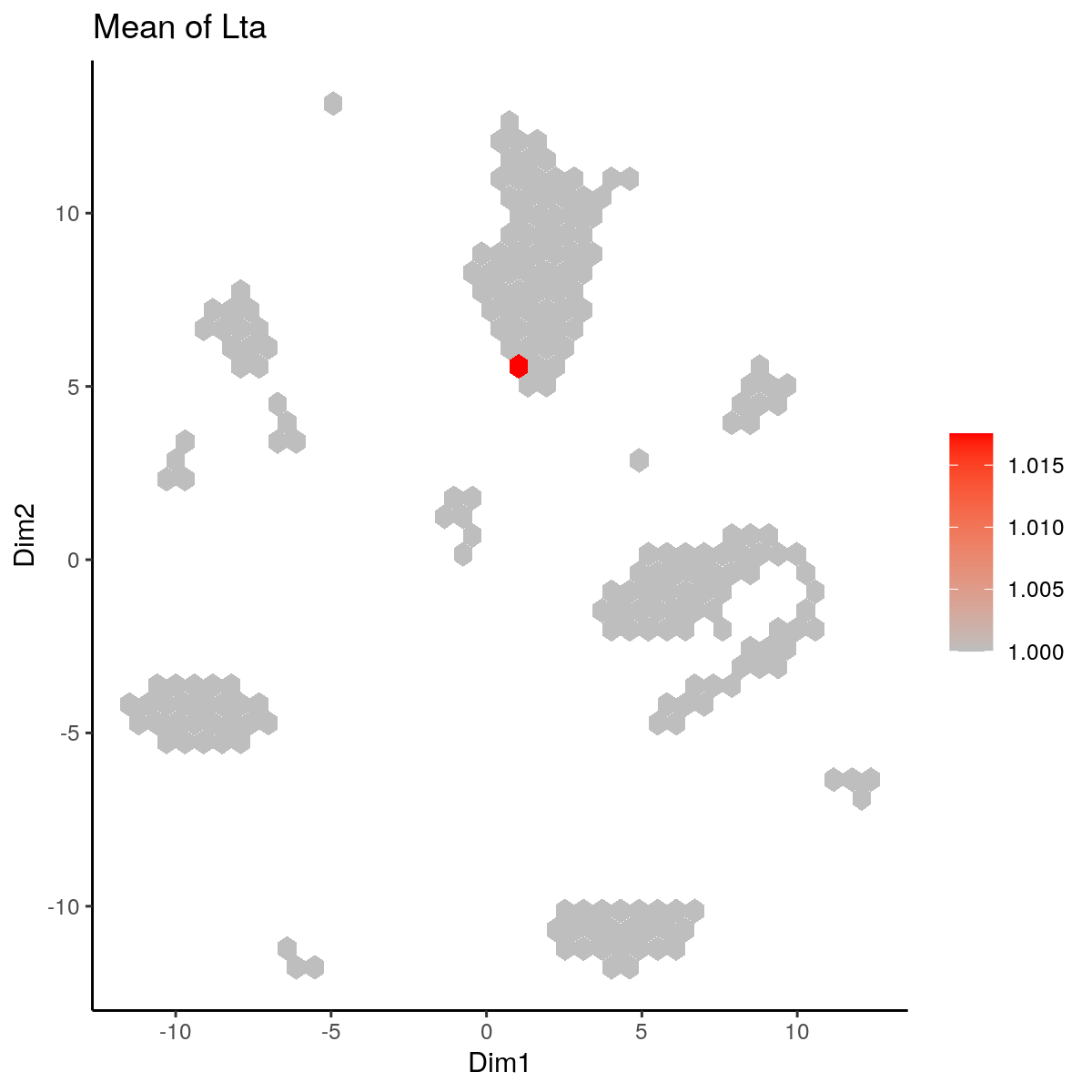

Supplement: Supplementary file 18 — Additional file 18. HTML report of VisualCortex. [file 12859_2023_5490_MOESM18_ESM.zip › output/report/Mouse_VisualCortex/figures/Ligand/16992.png]

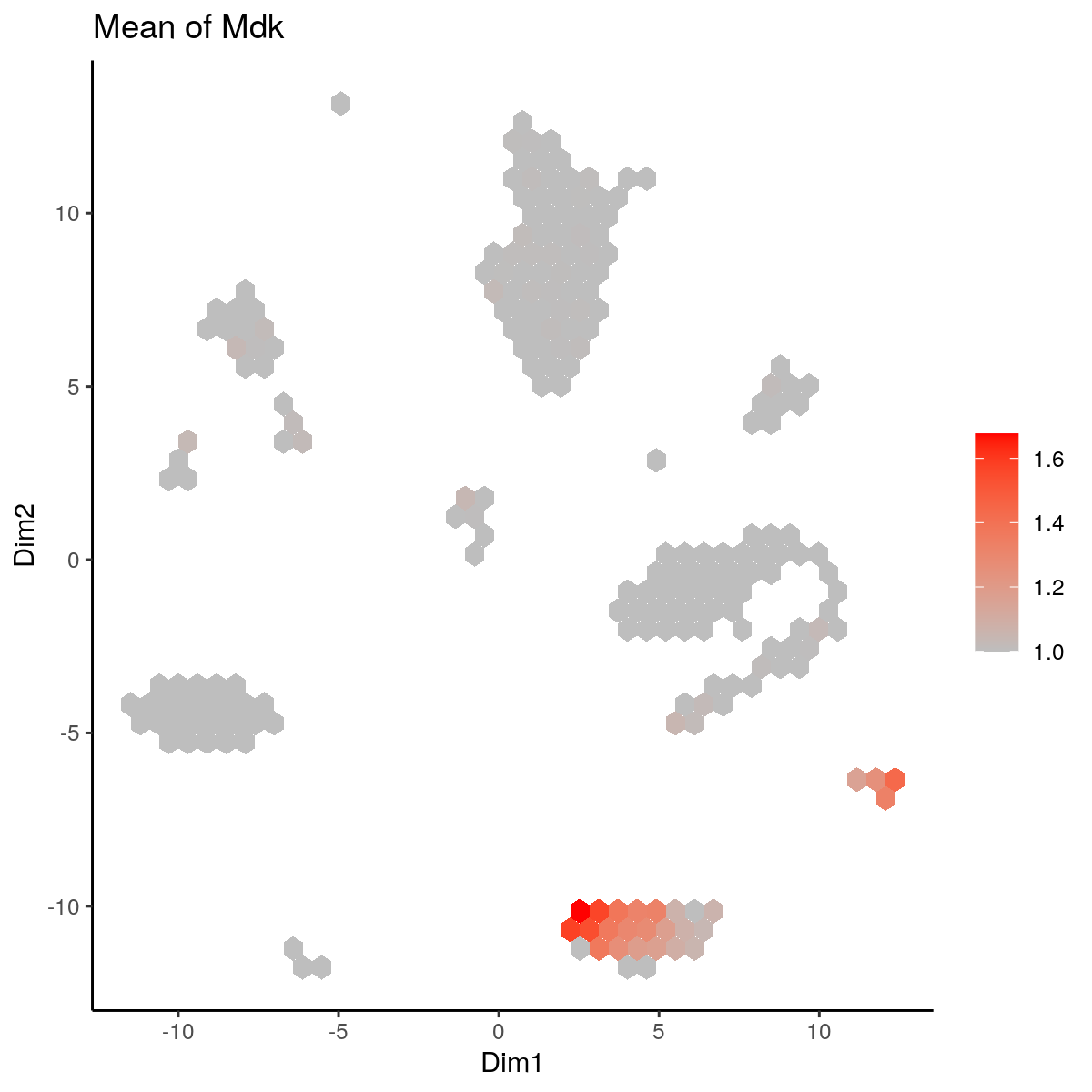

Supplement: Supplementary file 18 — Additional file 18. HTML report of VisualCortex. [file 12859_2023_5490_MOESM18_ESM.zip › output/report/Mouse_VisualCortex/figures/Ligand/17242.png]

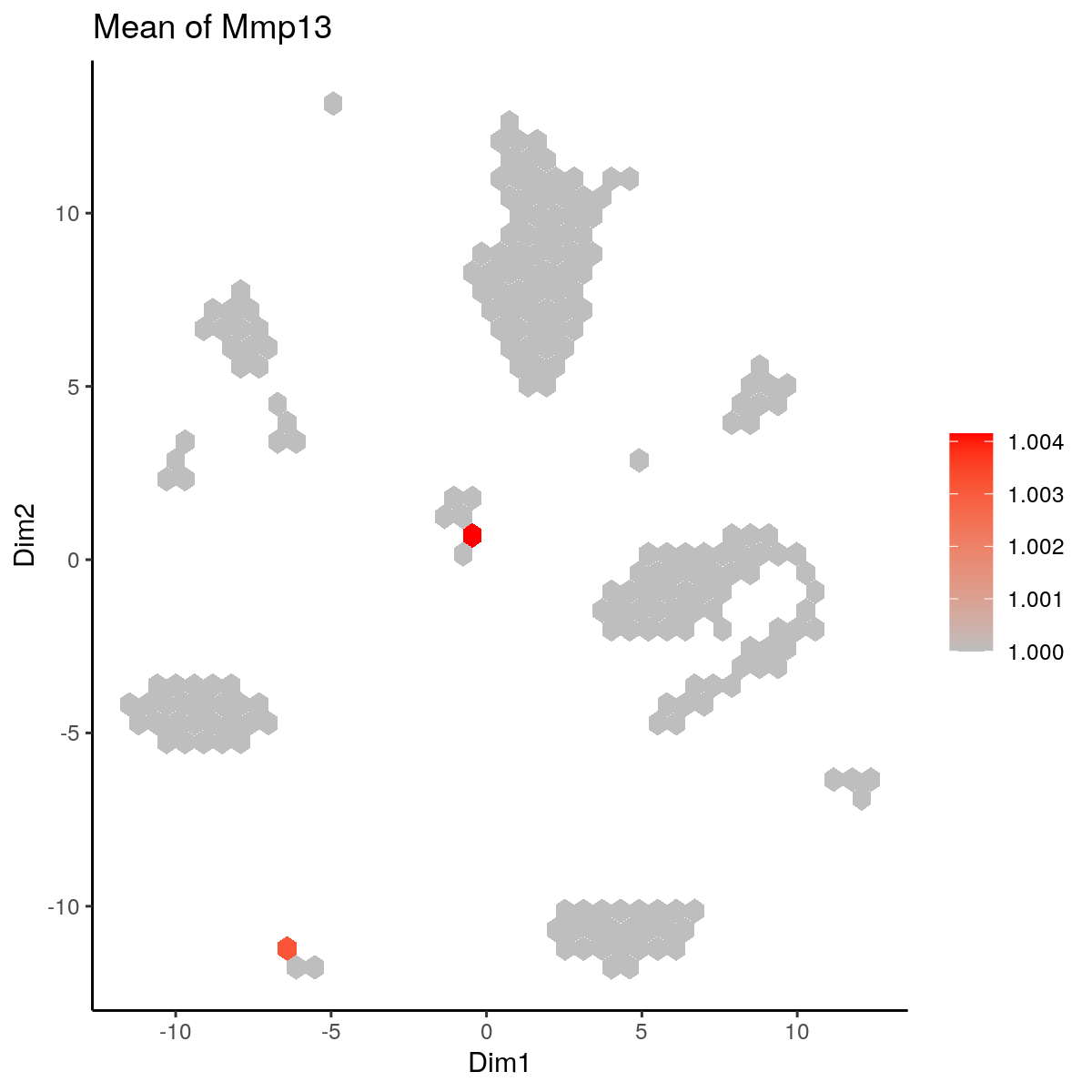

Supplement: Supplementary file 18 — Additional file 18. HTML report of VisualCortex. [file 12859_2023_5490_MOESM18_ESM.zip › output/report/Mouse_VisualCortex/figures/Ligand/17386.png]

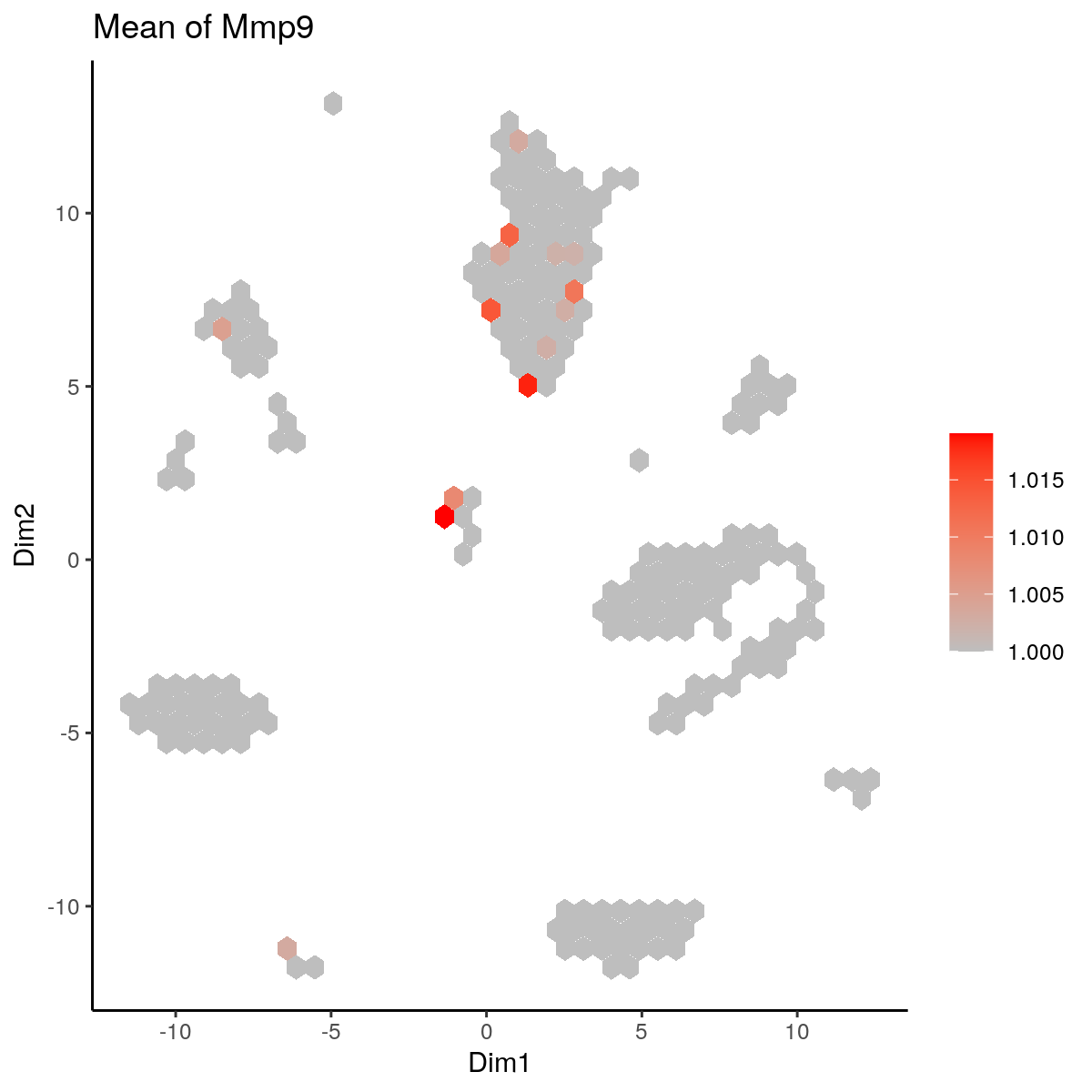

Supplement: Supplementary file 18 — Additional file 18. HTML report of VisualCortex. [file 12859_2023_5490_MOESM18_ESM.zip › output/report/Mouse_VisualCortex/figures/Ligand/17395.png]

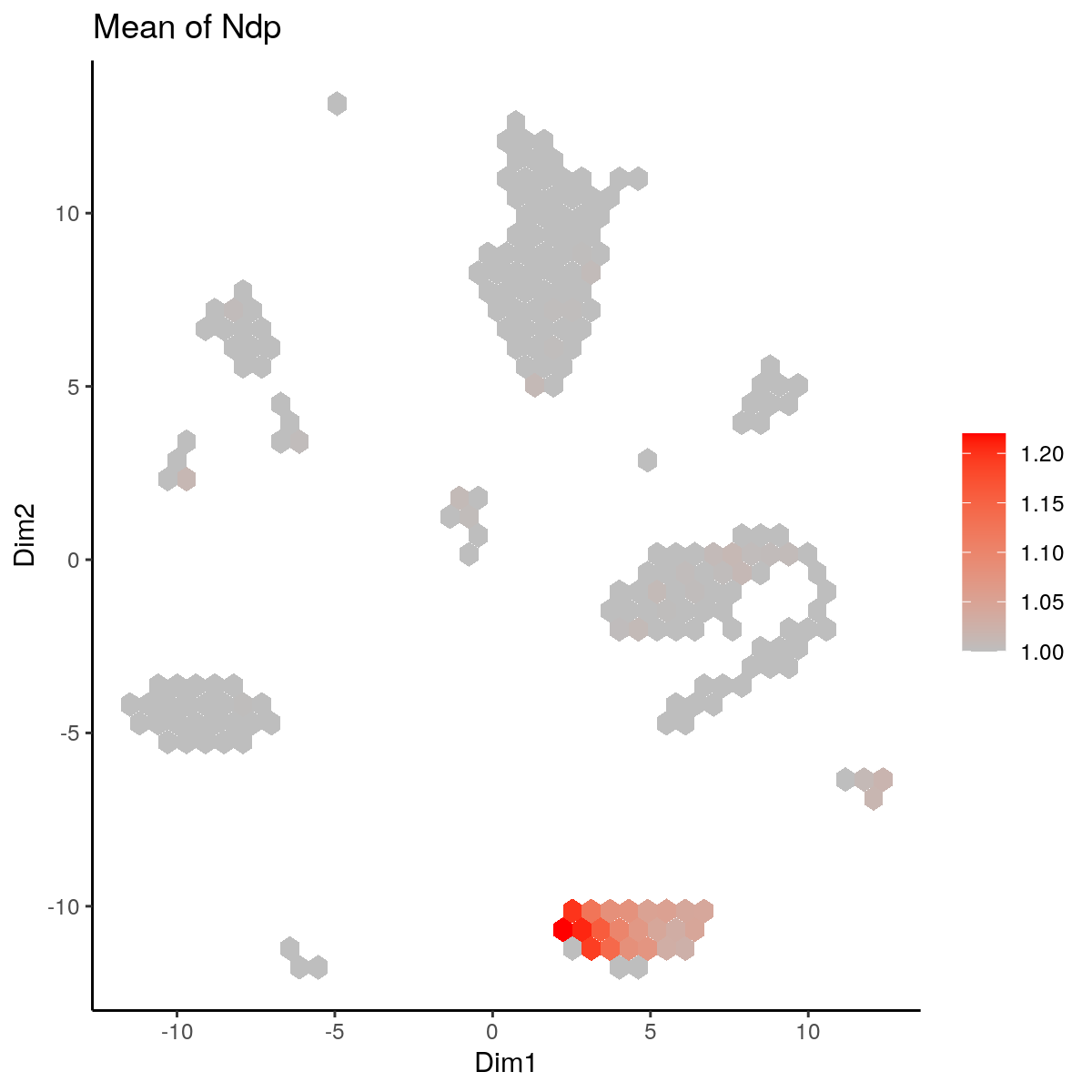

Supplement: Supplementary file 18 — Additional file 18. HTML report of VisualCortex. [file 12859_2023_5490_MOESM18_ESM.zip › output/report/Mouse_VisualCortex/figures/Ligand/17986.png]

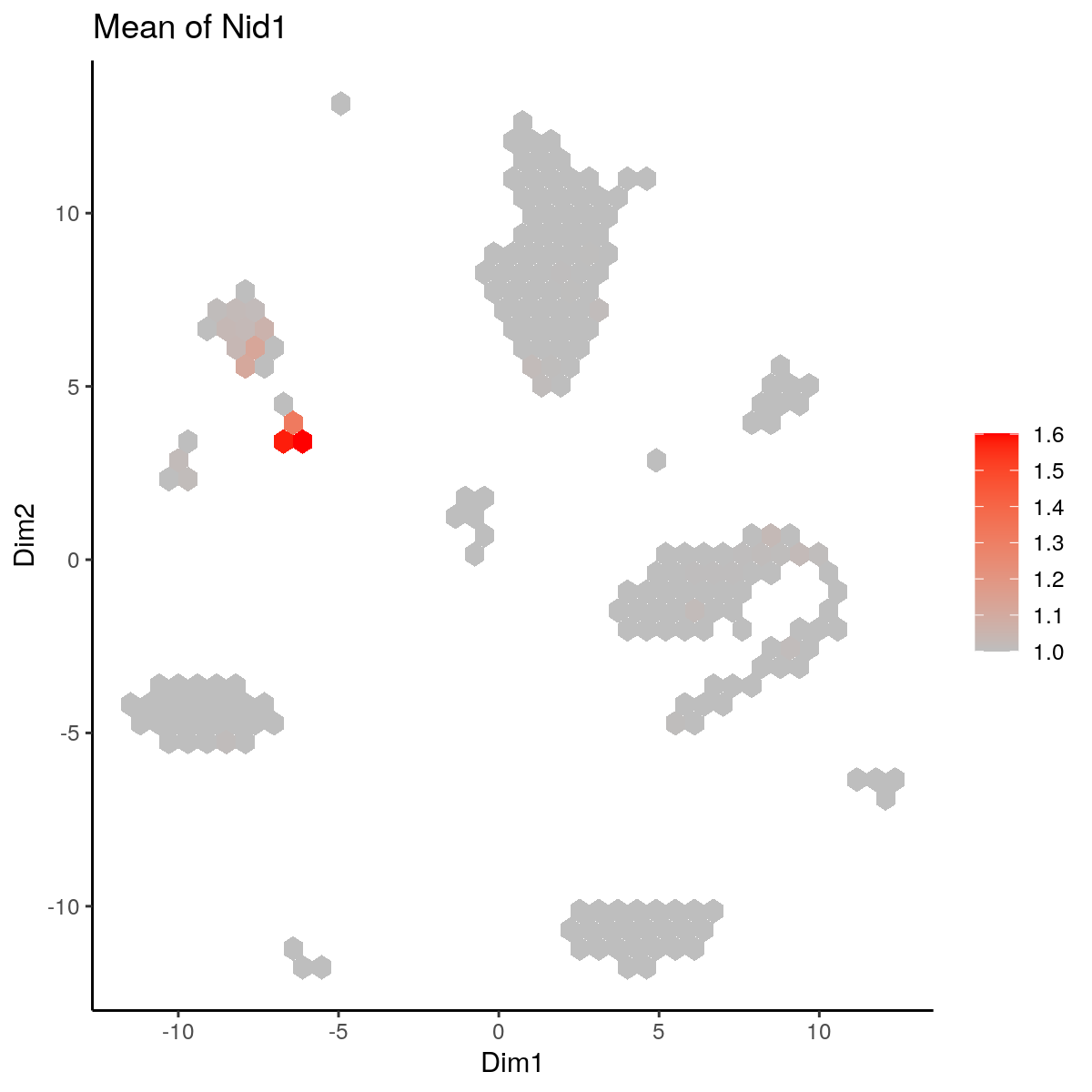

Supplement: Supplementary file 18 — Additional file 18. HTML report of VisualCortex. [file 12859_2023_5490_MOESM18_ESM.zip › output/report/Mouse_VisualCortex/figures/Ligand/18073.png]

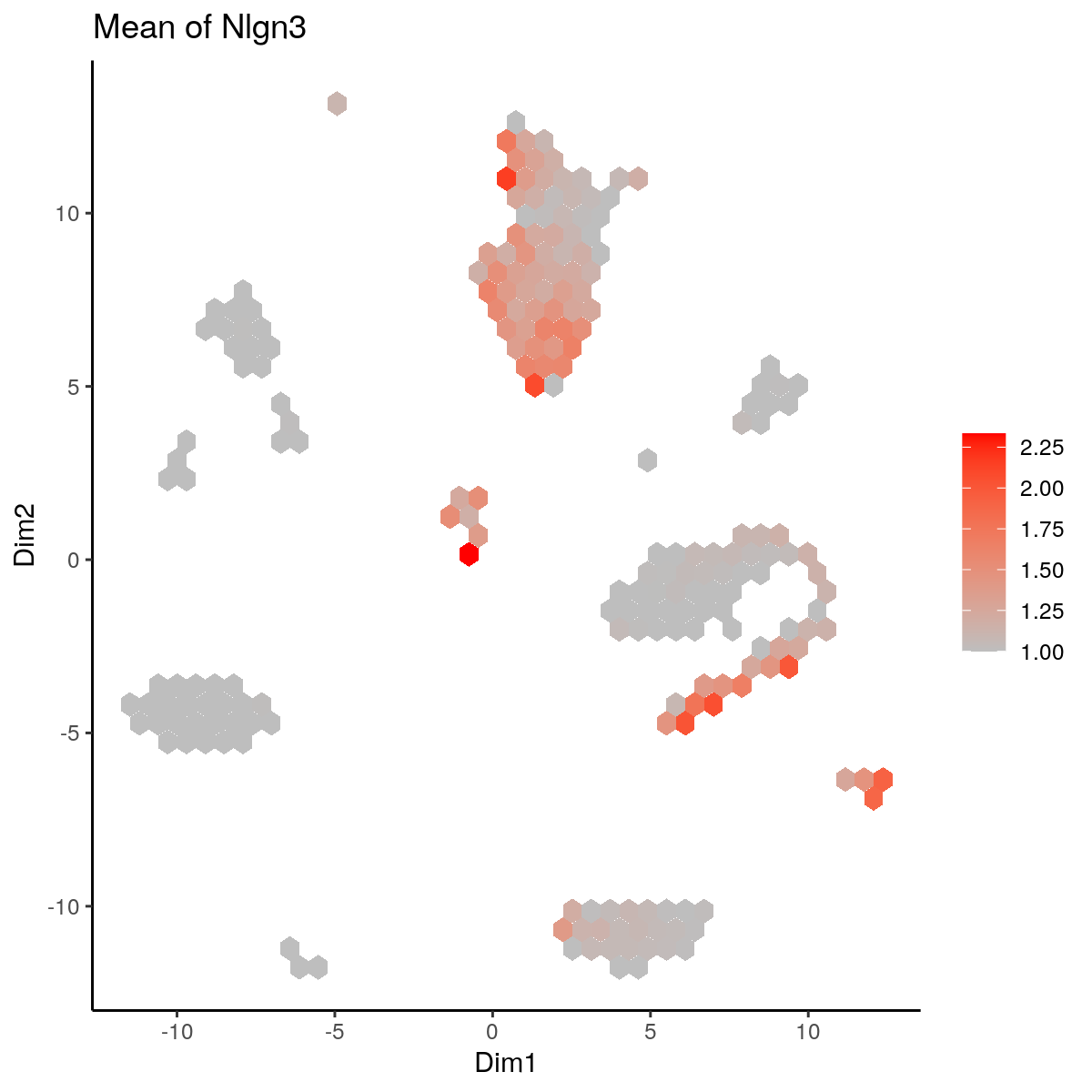

Supplement: Supplementary file 18 — Additional file 18. HTML report of VisualCortex. [file 12859_2023_5490_MOESM18_ESM.zip › output/report/Mouse_VisualCortex/figures/Ligand/245537.png]

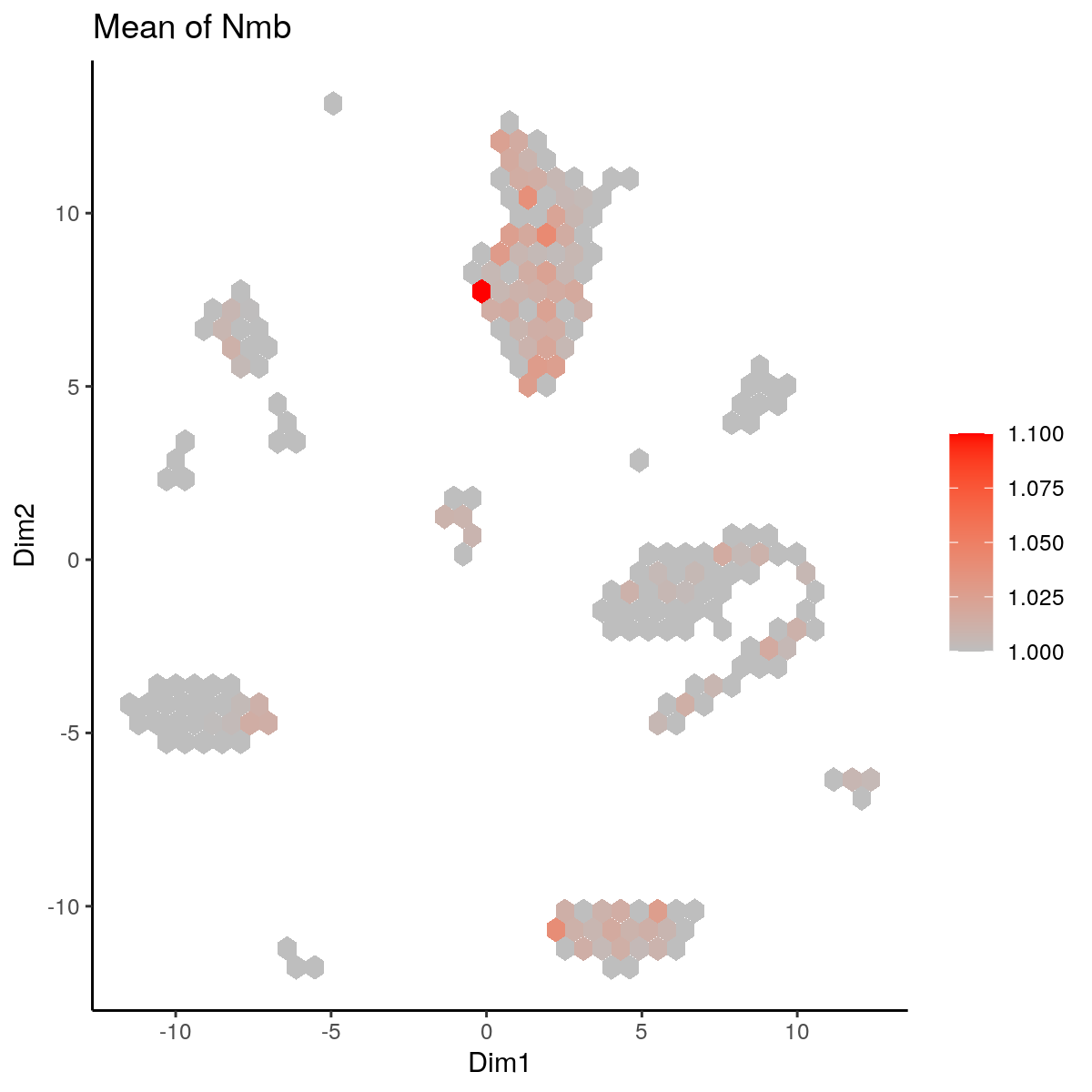

Supplement: Supplementary file 18 — Additional file 18. HTML report of VisualCortex. [file 12859_2023_5490_MOESM18_ESM.zip › output/report/Mouse_VisualCortex/figures/Ligand/68039.png]

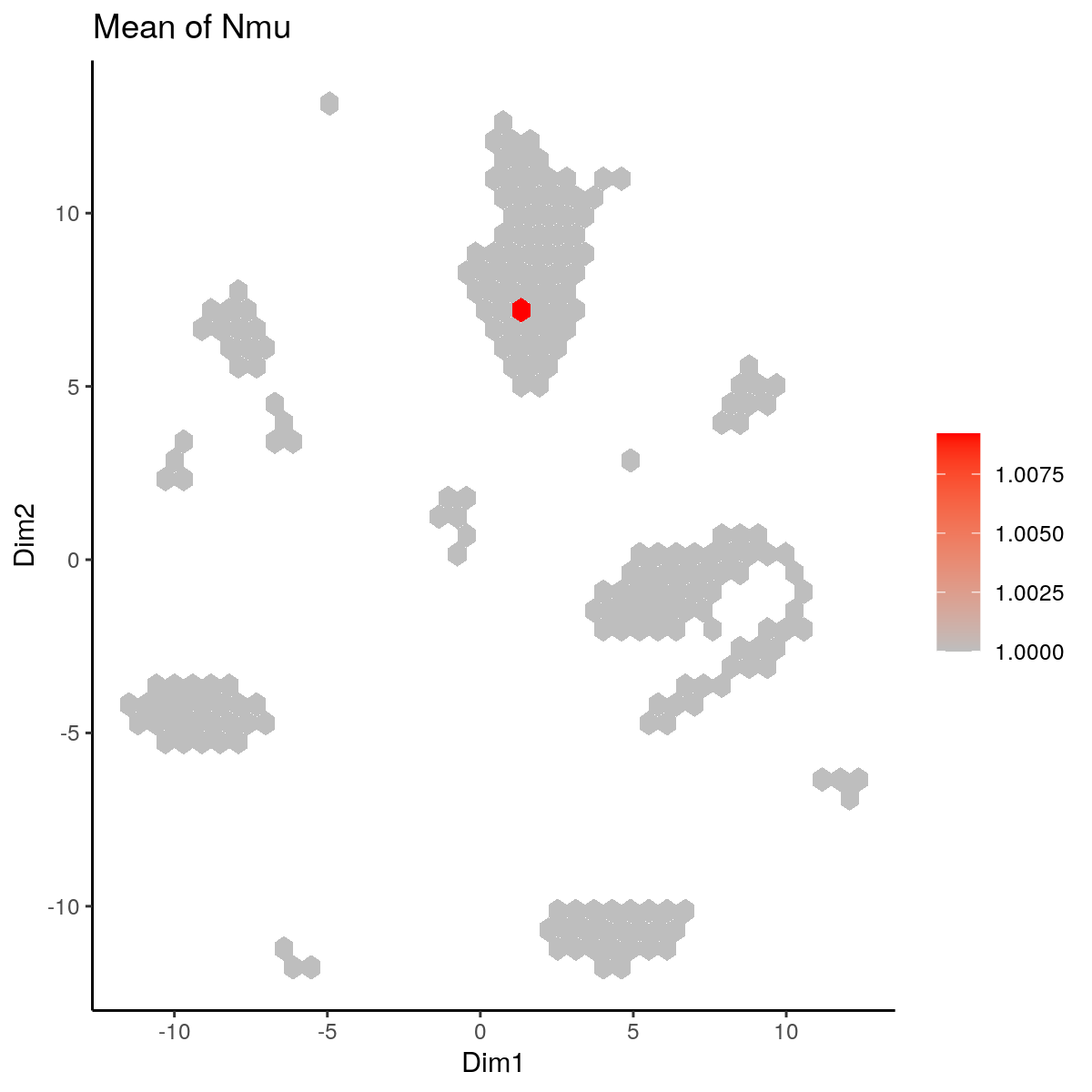

Supplement: Supplementary file 18 — Additional file 18. HTML report of VisualCortex. [file 12859_2023_5490_MOESM18_ESM.zip › output/report/Mouse_VisualCortex/figures/Ligand/56183.png]

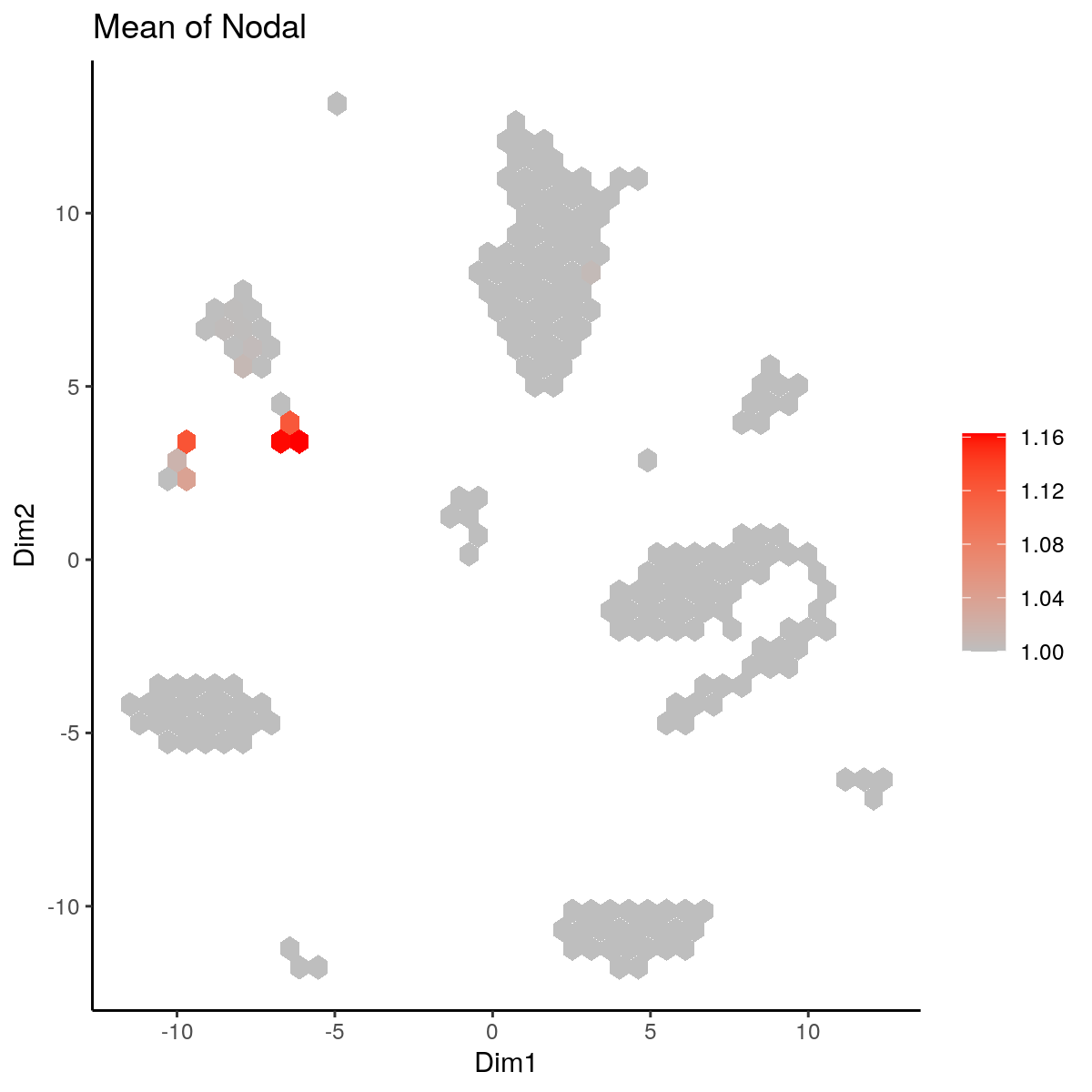

Supplement: Supplementary file 18 — Additional file 18. HTML report of VisualCortex. [file 12859_2023_5490_MOESM18_ESM.zip › output/report/Mouse_VisualCortex/figures/Ligand/18119.png]

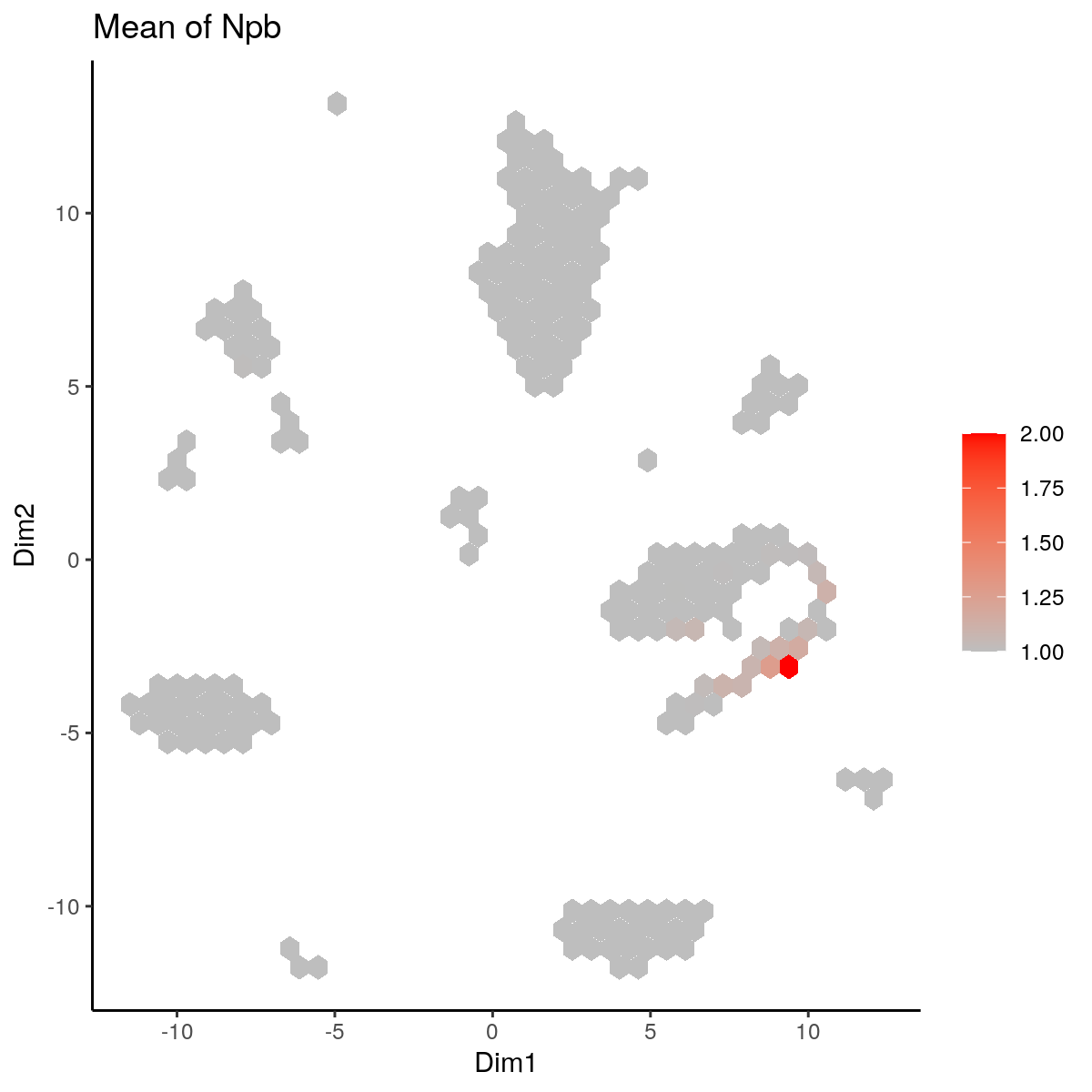

Supplement: Supplementary file 18 — Additional file 18. HTML report of VisualCortex. [file 12859_2023_5490_MOESM18_ESM.zip › output/report/Mouse_VisualCortex/figures/Ligand/208990.png]

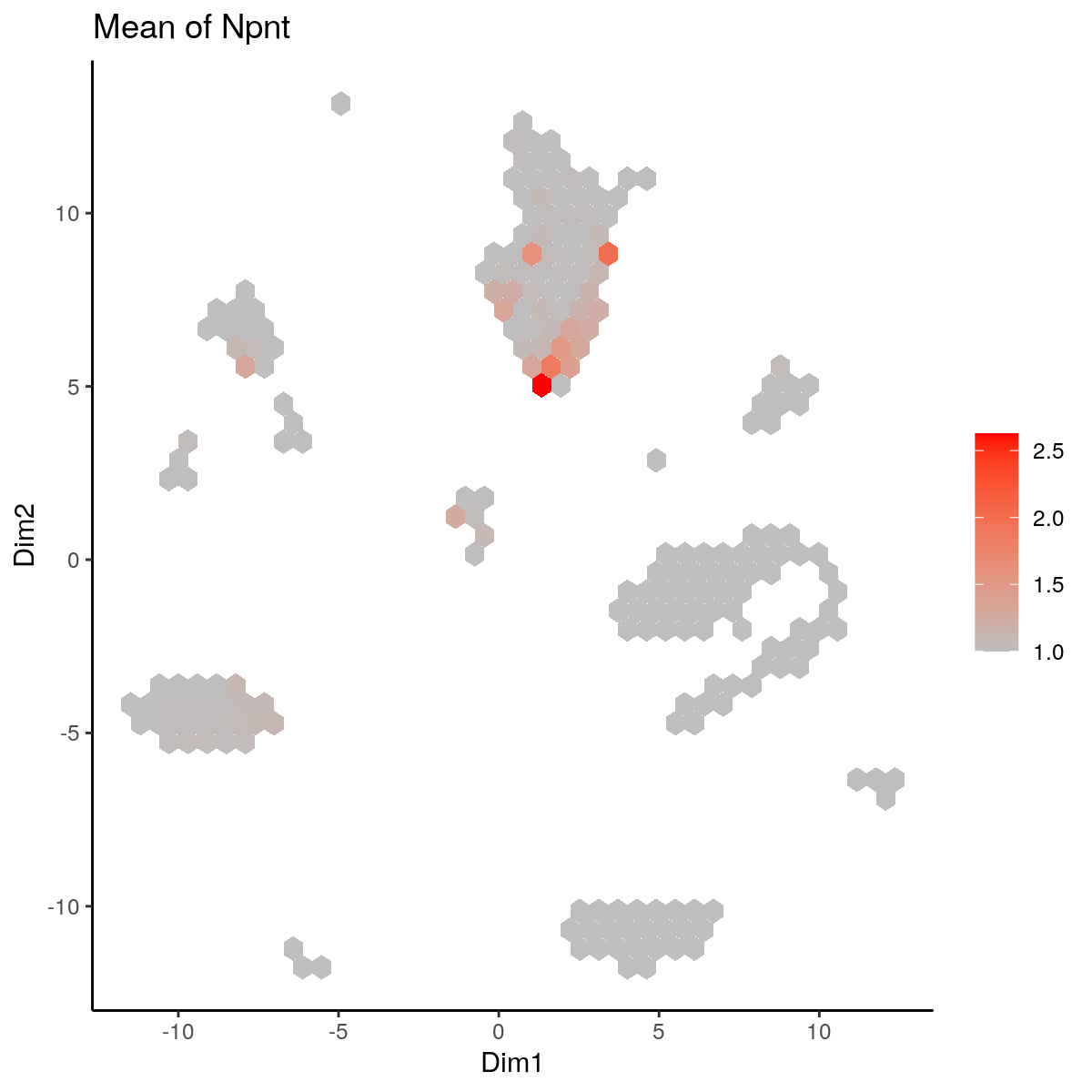

Supplement: Supplementary file 18 — Additional file 18. HTML report of VisualCortex. [file 12859_2023_5490_MOESM18_ESM.zip › output/report/Mouse_VisualCortex/figures/Ligand/114249.png]

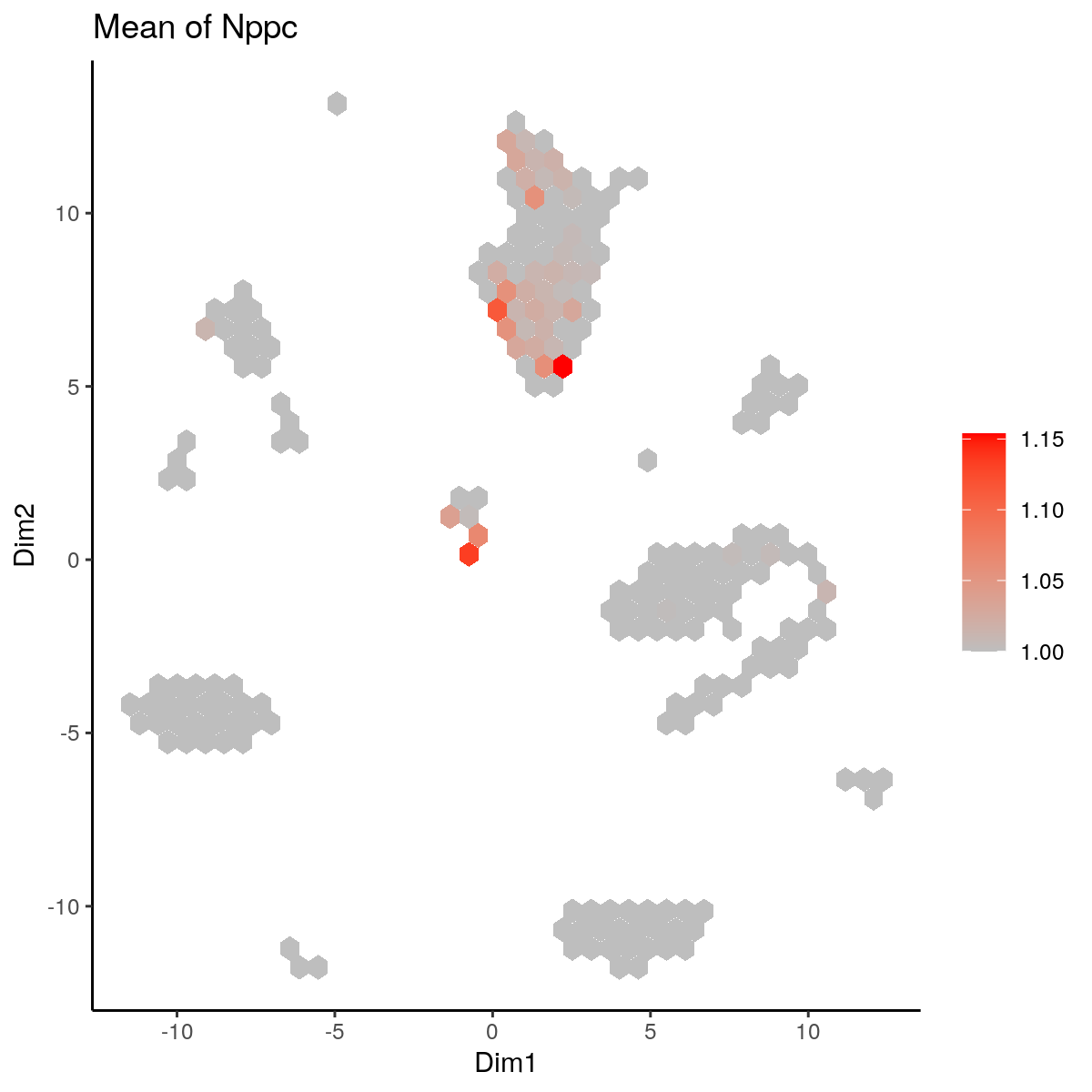

Supplement: Supplementary file 18 — Additional file 18. HTML report of VisualCortex. [file 12859_2023_5490_MOESM18_ESM.zip › output/report/Mouse_VisualCortex/figures/Ligand/18159.png]

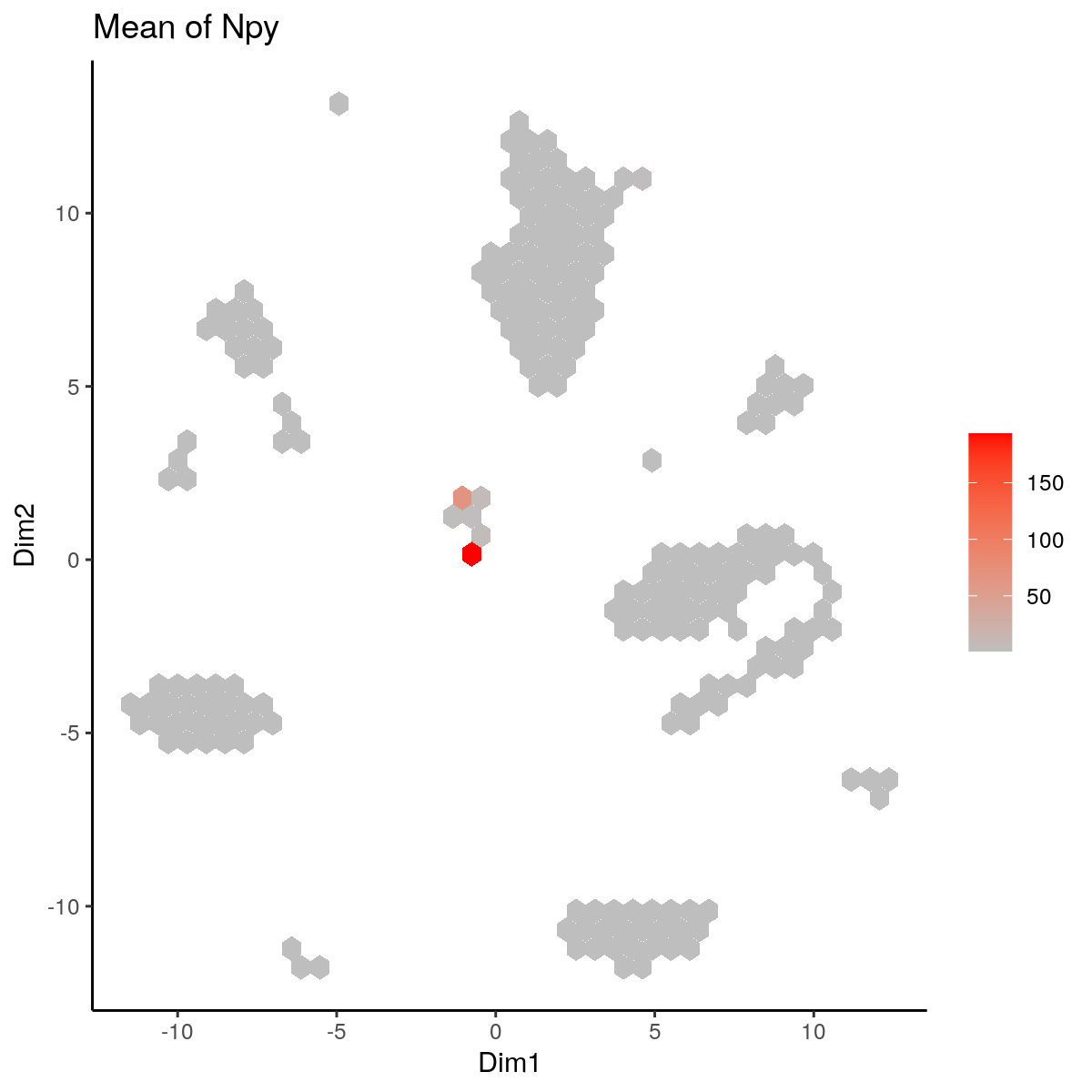

Supplement: Supplementary file 18 — Additional file 18. HTML report of VisualCortex. [file 12859_2023_5490_MOESM18_ESM.zip › output/report/Mouse_VisualCortex/figures/Ligand/109648.png]

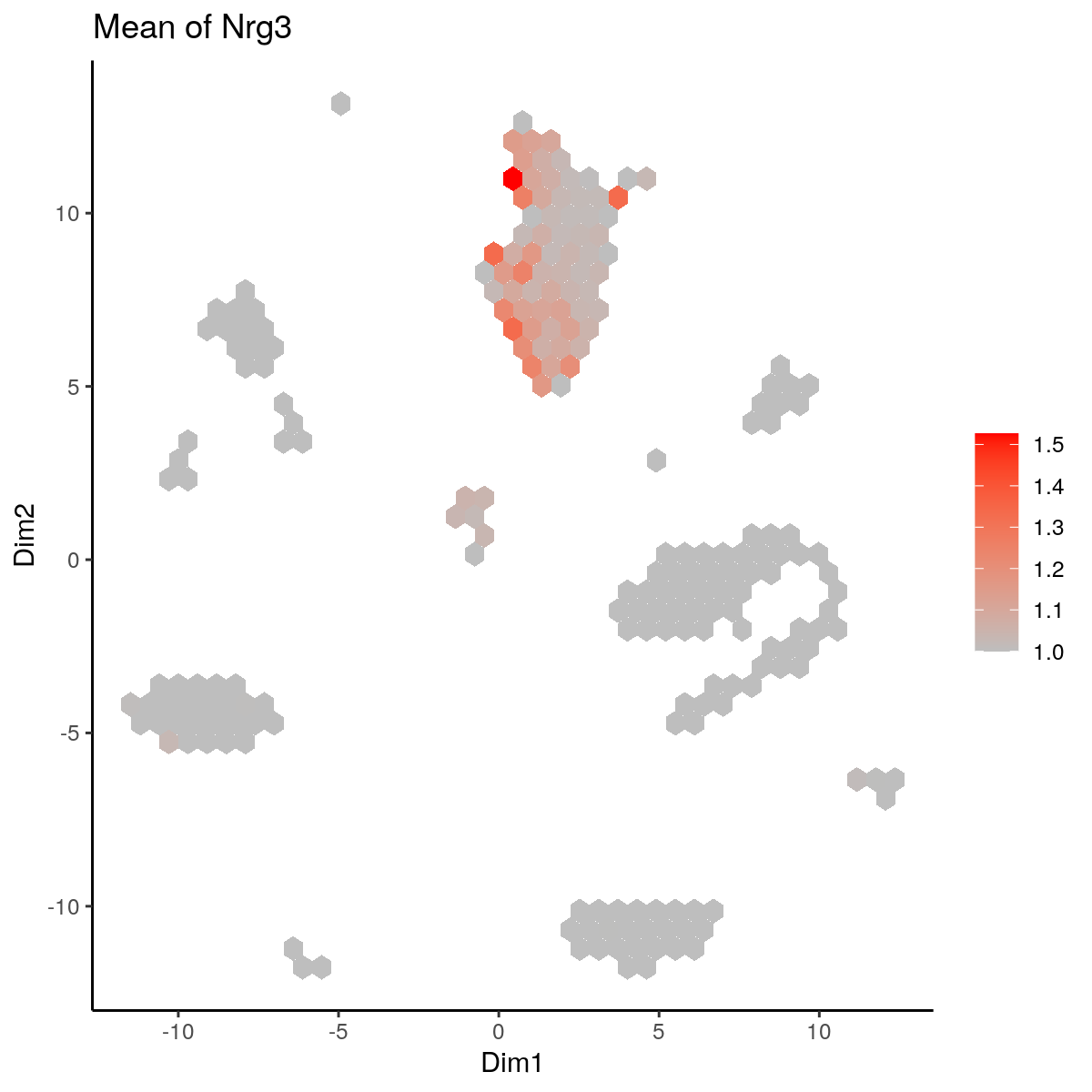

Supplement: Supplementary file 18 — Additional file 18. HTML report of VisualCortex. [file 12859_2023_5490_MOESM18_ESM.zip › output/report/Mouse_VisualCortex/figures/Ligand/18183.png]

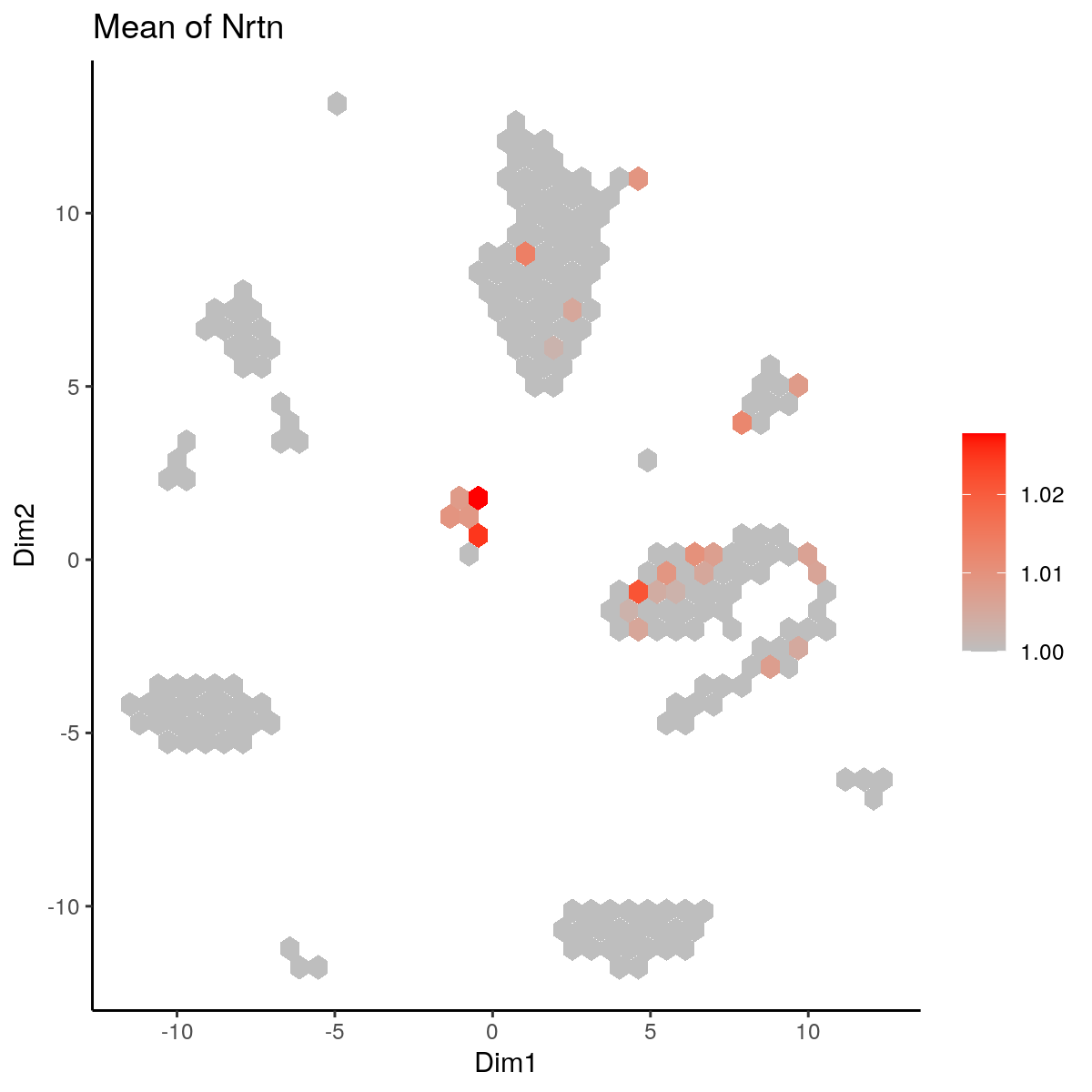

Supplement: Supplementary file 18 — Additional file 18. HTML report of VisualCortex. [file 12859_2023_5490_MOESM18_ESM.zip › output/report/Mouse_VisualCortex/figures/Ligand/18188.png]

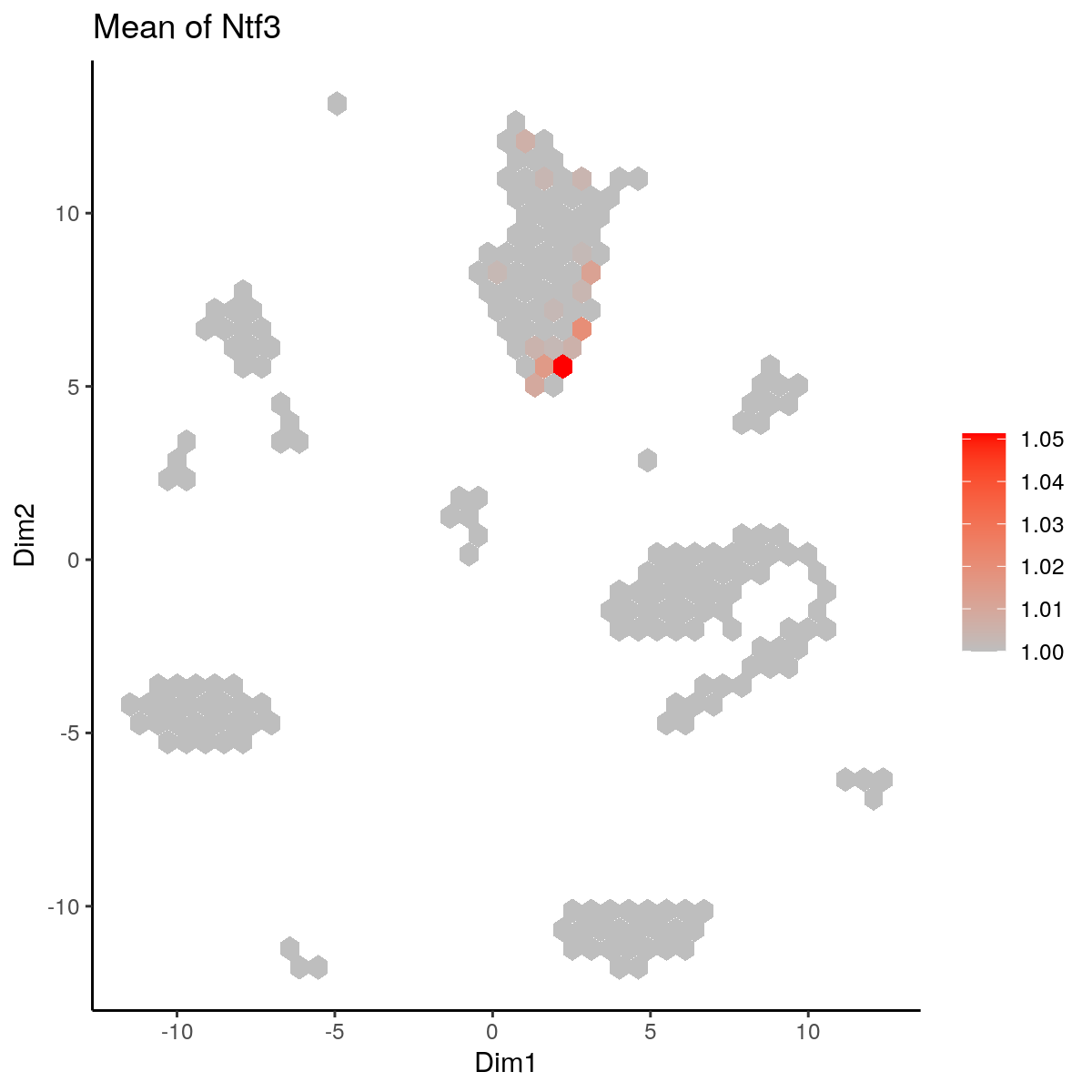

Supplement: Supplementary file 18 — Additional file 18. HTML report of VisualCortex. [file 12859_2023_5490_MOESM18_ESM.zip › output/report/Mouse_VisualCortex/figures/Ligand/18205.png]

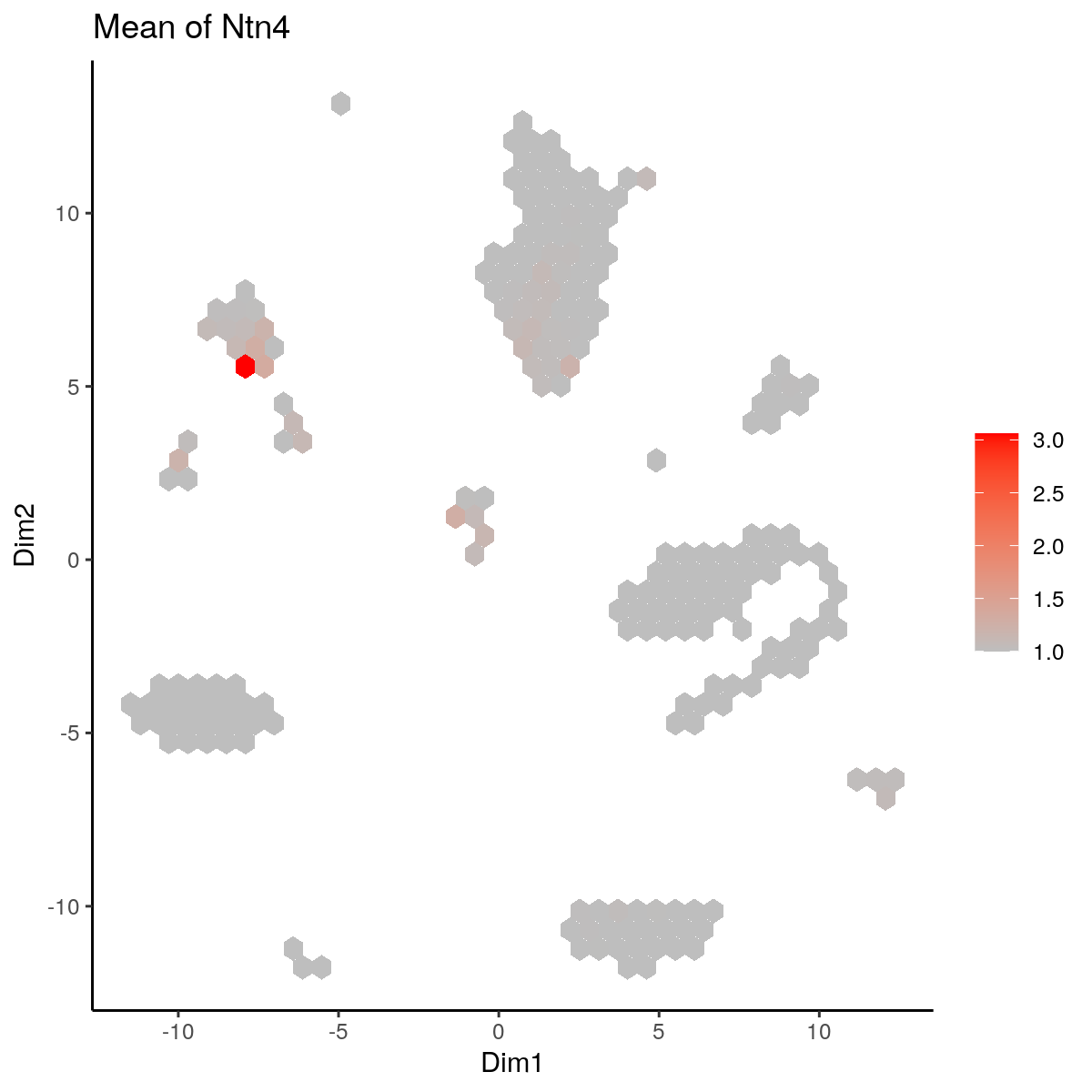

Supplement: Supplementary file 18 — Additional file 18. HTML report of VisualCortex. [file 12859_2023_5490_MOESM18_ESM.zip › output/report/Mouse_VisualCortex/figures/Ligand/57764.png]

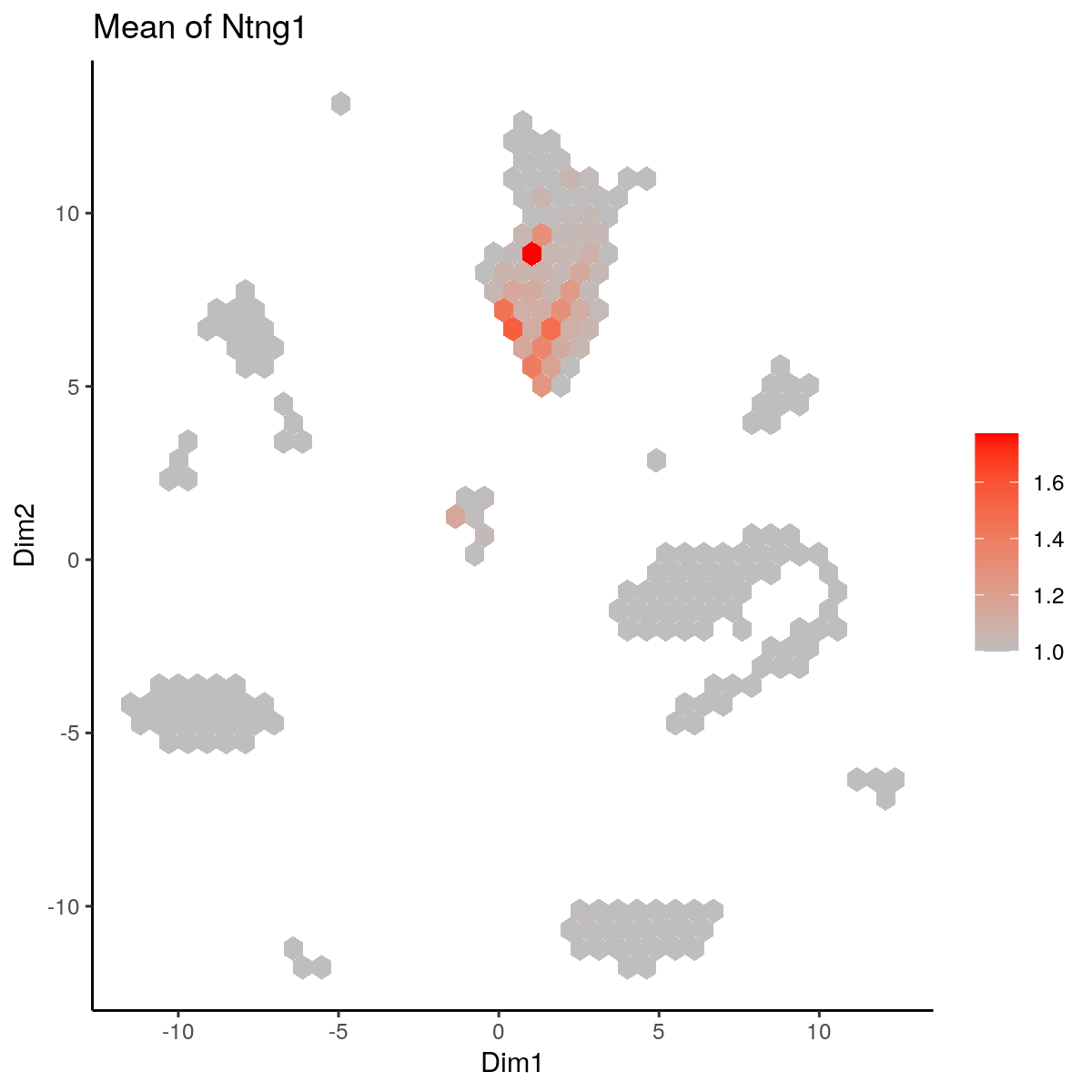

Supplement: Supplementary file 18 — Additional file 18. HTML report of VisualCortex. [file 12859_2023_5490_MOESM18_ESM.zip › output/report/Mouse_VisualCortex/figures/Ligand/80883.png]

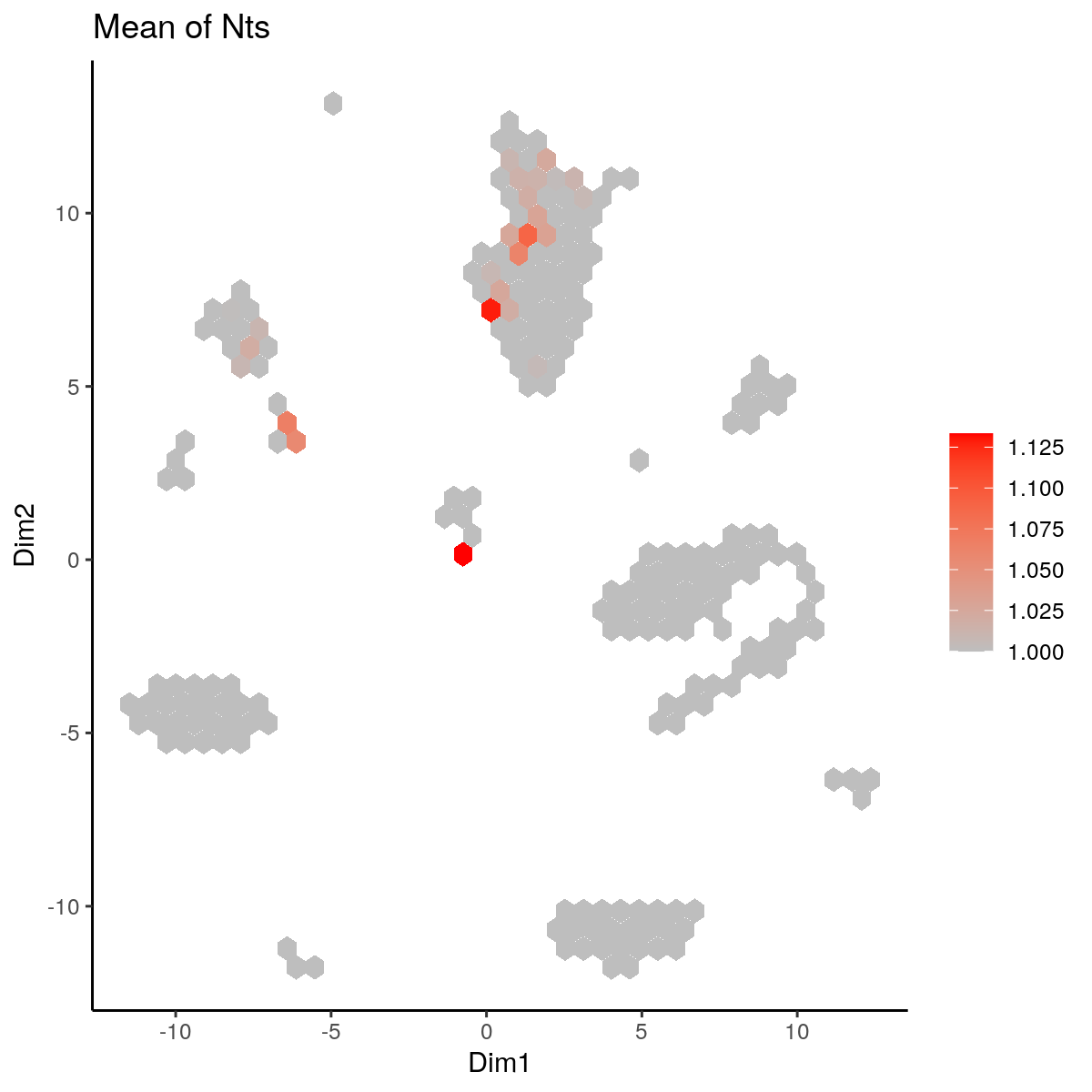

Supplement: Supplementary file 18 — Additional file 18. HTML report of VisualCortex. [file 12859_2023_5490_MOESM18_ESM.zip › output/report/Mouse_VisualCortex/figures/Ligand/67405.png]

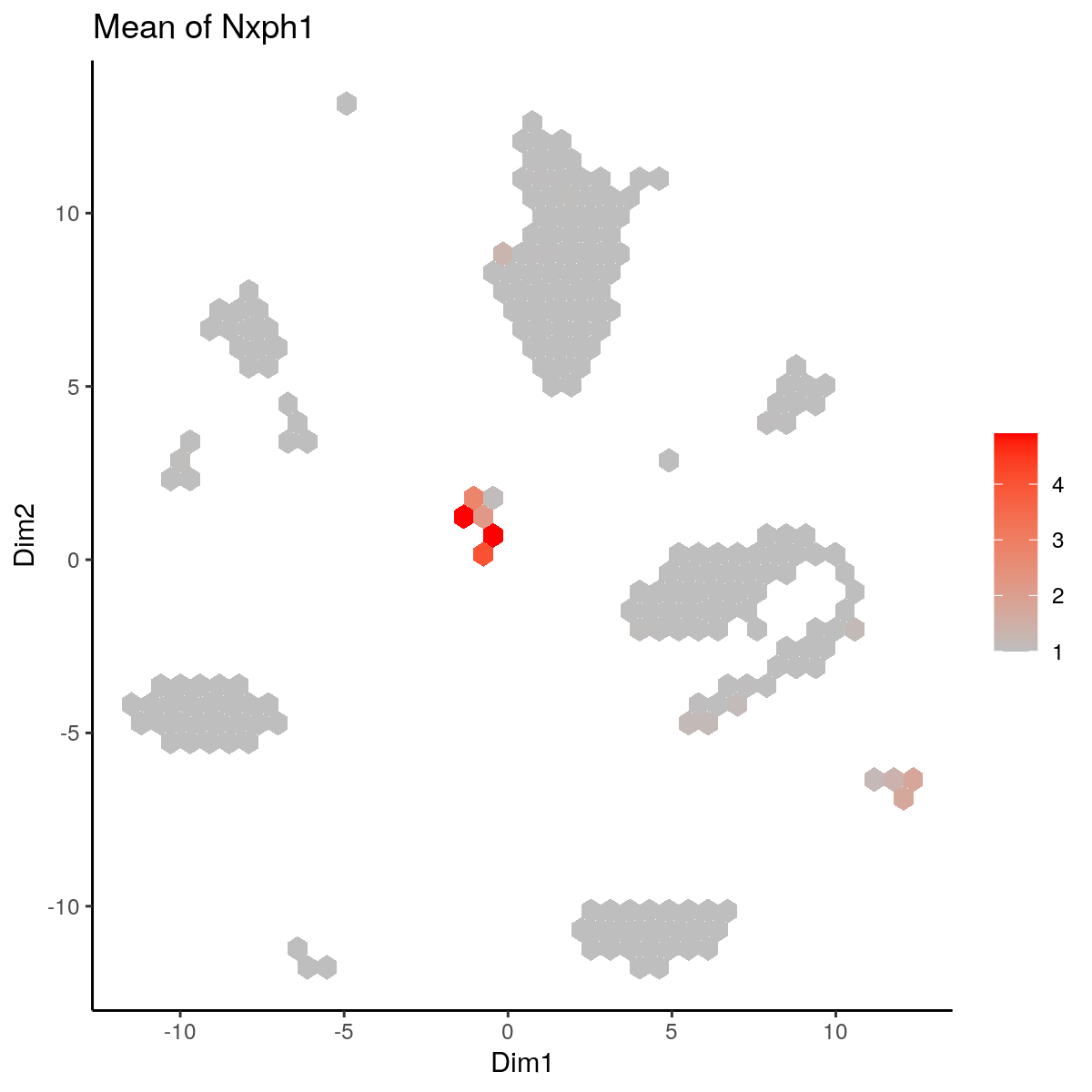

Supplement: Supplementary file 18 — Additional file 18. HTML report of VisualCortex. [file 12859_2023_5490_MOESM18_ESM.zip › output/report/Mouse_VisualCortex/figures/Ligand/18231.png]

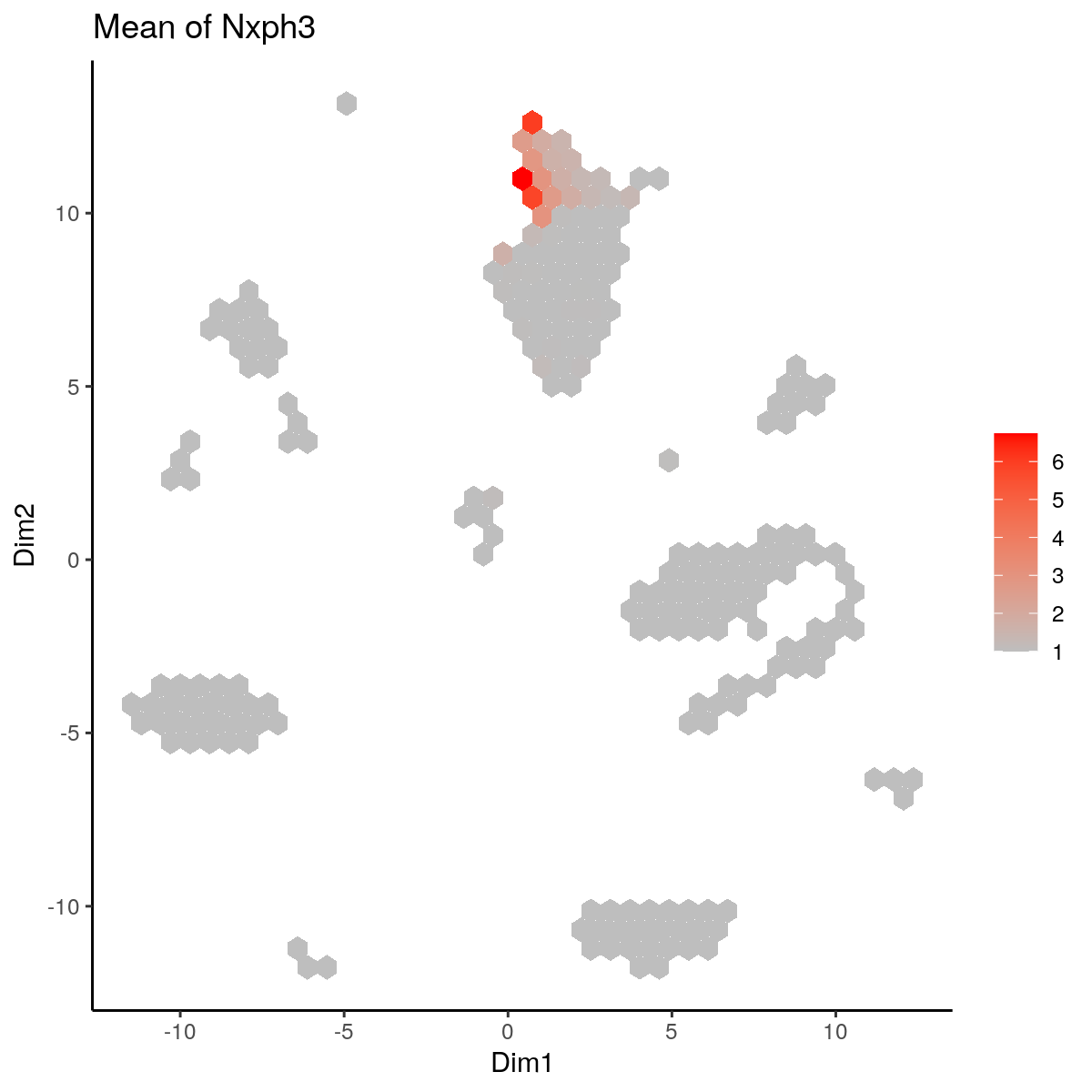

Supplement: Supplementary file 18 — Additional file 18. HTML report of VisualCortex. [file 12859_2023_5490_MOESM18_ESM.zip › output/report/Mouse_VisualCortex/figures/Ligand/104079.png]

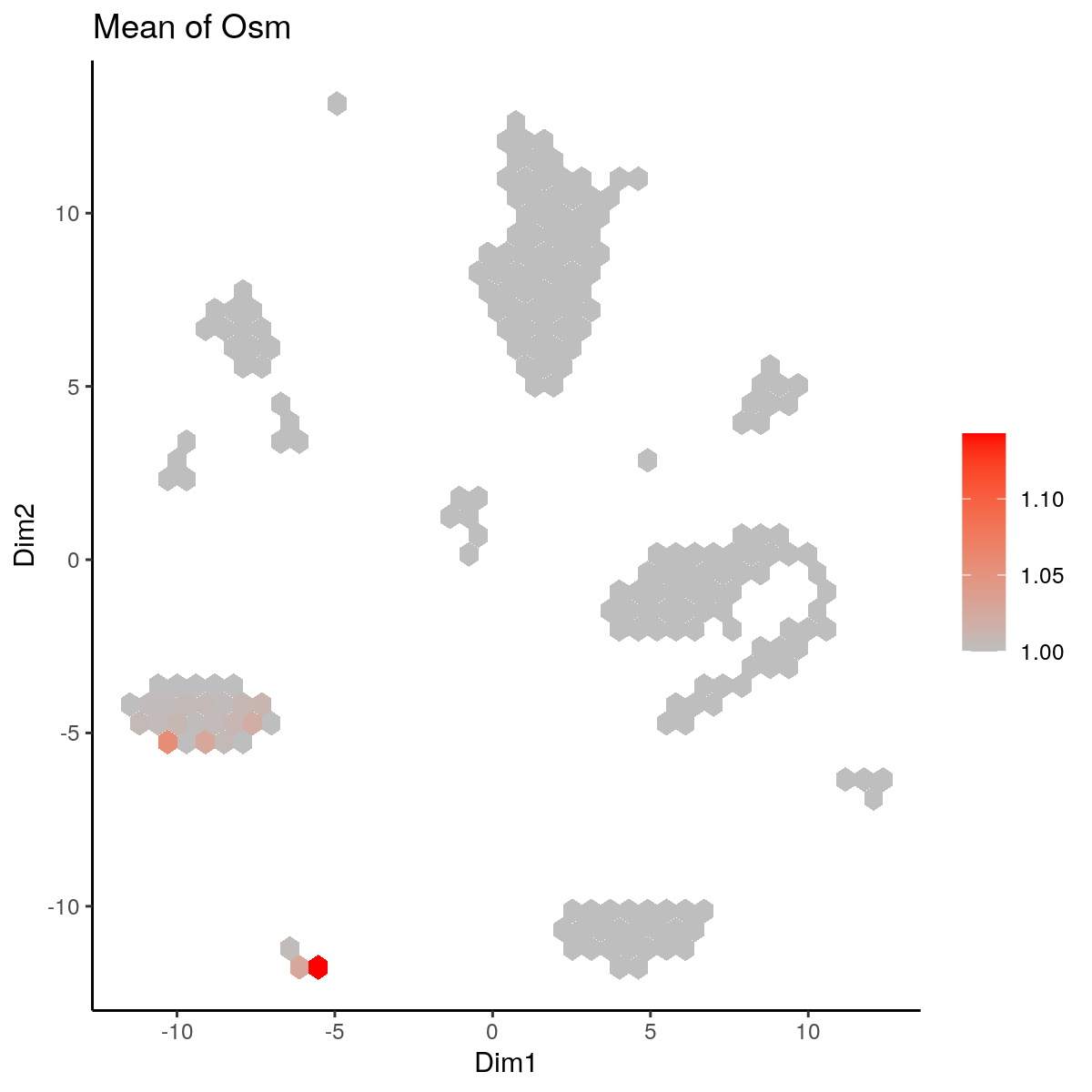

Supplement: Supplementary file 18 — Additional file 18. HTML report of VisualCortex. [file 12859_2023_5490_MOESM18_ESM.zip › output/report/Mouse_VisualCortex/figures/Ligand/18413.png]

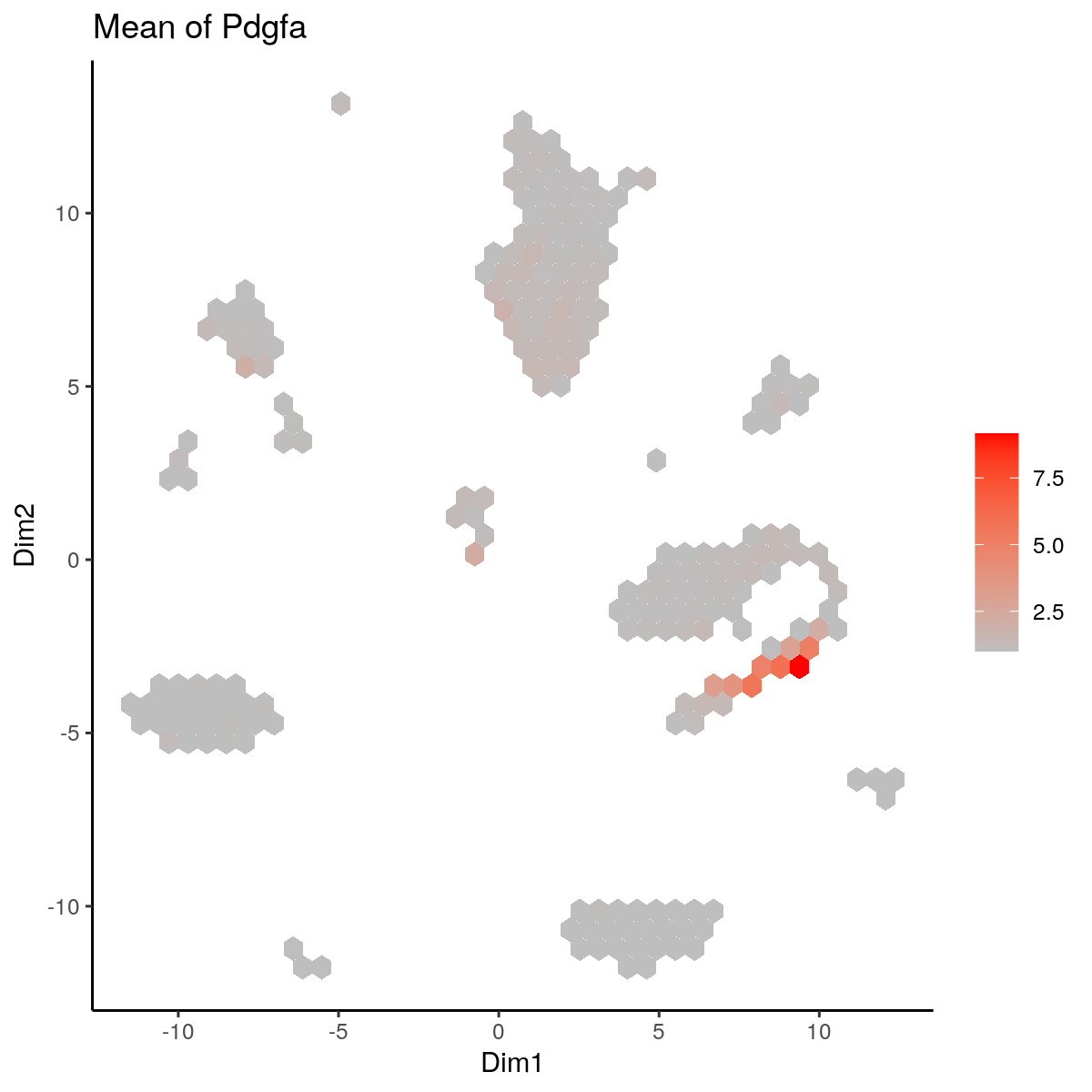

Supplement: Supplementary file 18 — Additional file 18. HTML report of VisualCortex. [file 12859_2023_5490_MOESM18_ESM.zip › output/report/Mouse_VisualCortex/figures/Ligand/18590.png]

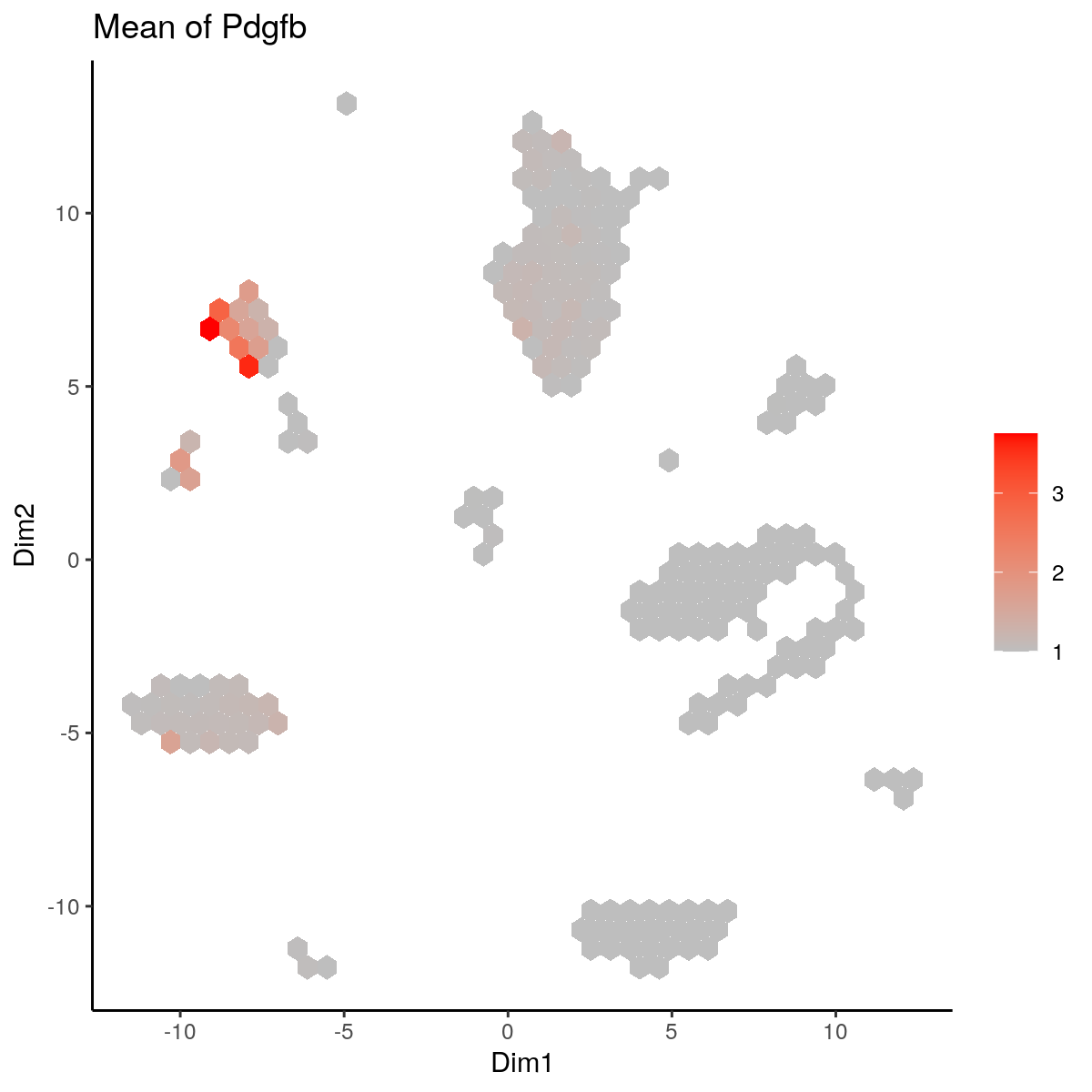

Supplement: Supplementary file 18 — Additional file 18. HTML report of VisualCortex. [file 12859_2023_5490_MOESM18_ESM.zip › output/report/Mouse_VisualCortex/figures/Ligand/18591.png]

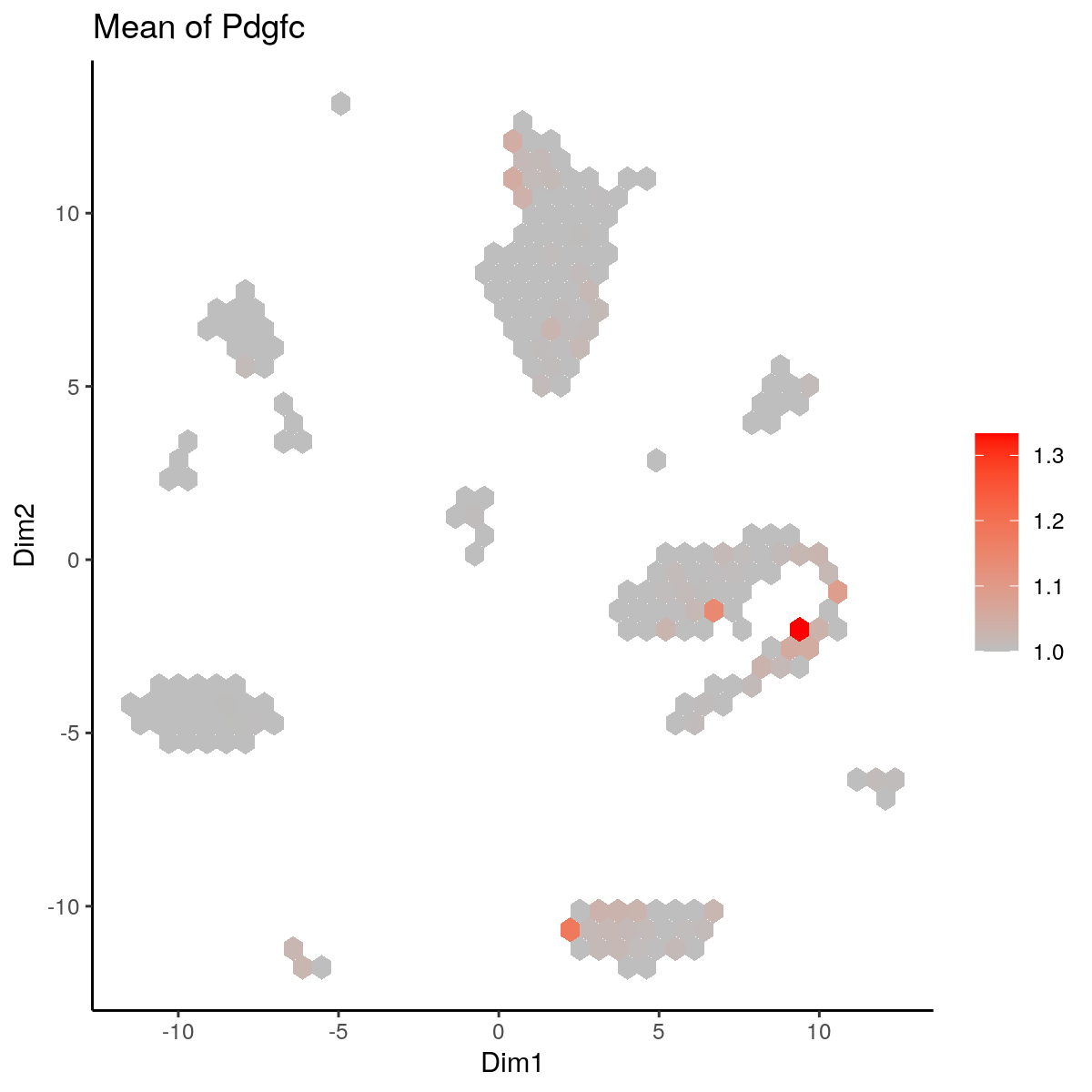

Supplement: Supplementary file 18 — Additional file 18. HTML report of VisualCortex. [file 12859_2023_5490_MOESM18_ESM.zip › output/report/Mouse_VisualCortex/figures/Ligand/54635.png]

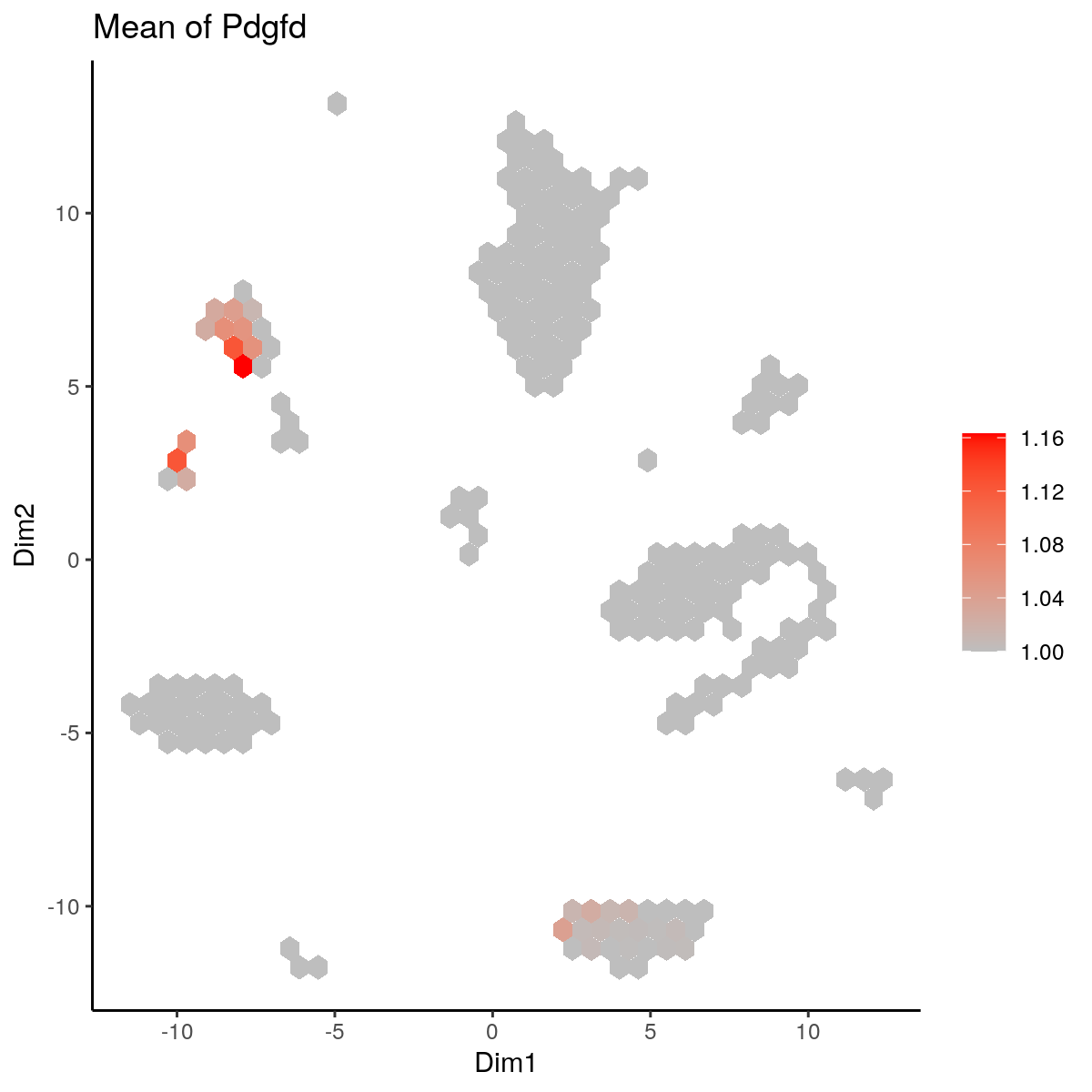

Supplement: Supplementary file 18 — Additional file 18. HTML report of VisualCortex. [file 12859_2023_5490_MOESM18_ESM.zip › output/report/Mouse_VisualCortex/figures/Ligand/71785.png]

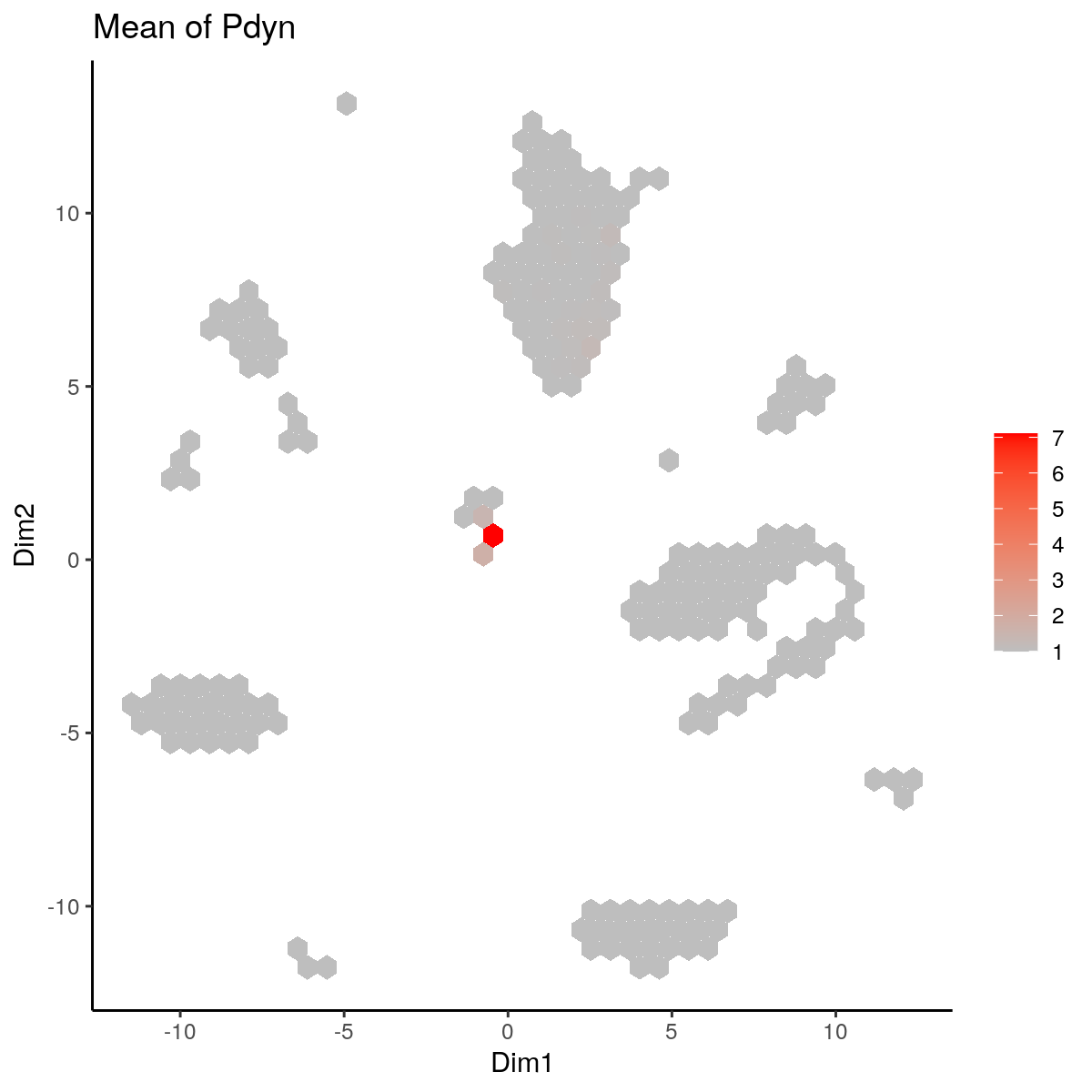

Supplement: Supplementary file 18 — Additional file 18. HTML report of VisualCortex. [file 12859_2023_5490_MOESM18_ESM.zip › output/report/Mouse_VisualCortex/figures/Ligand/18610.png]

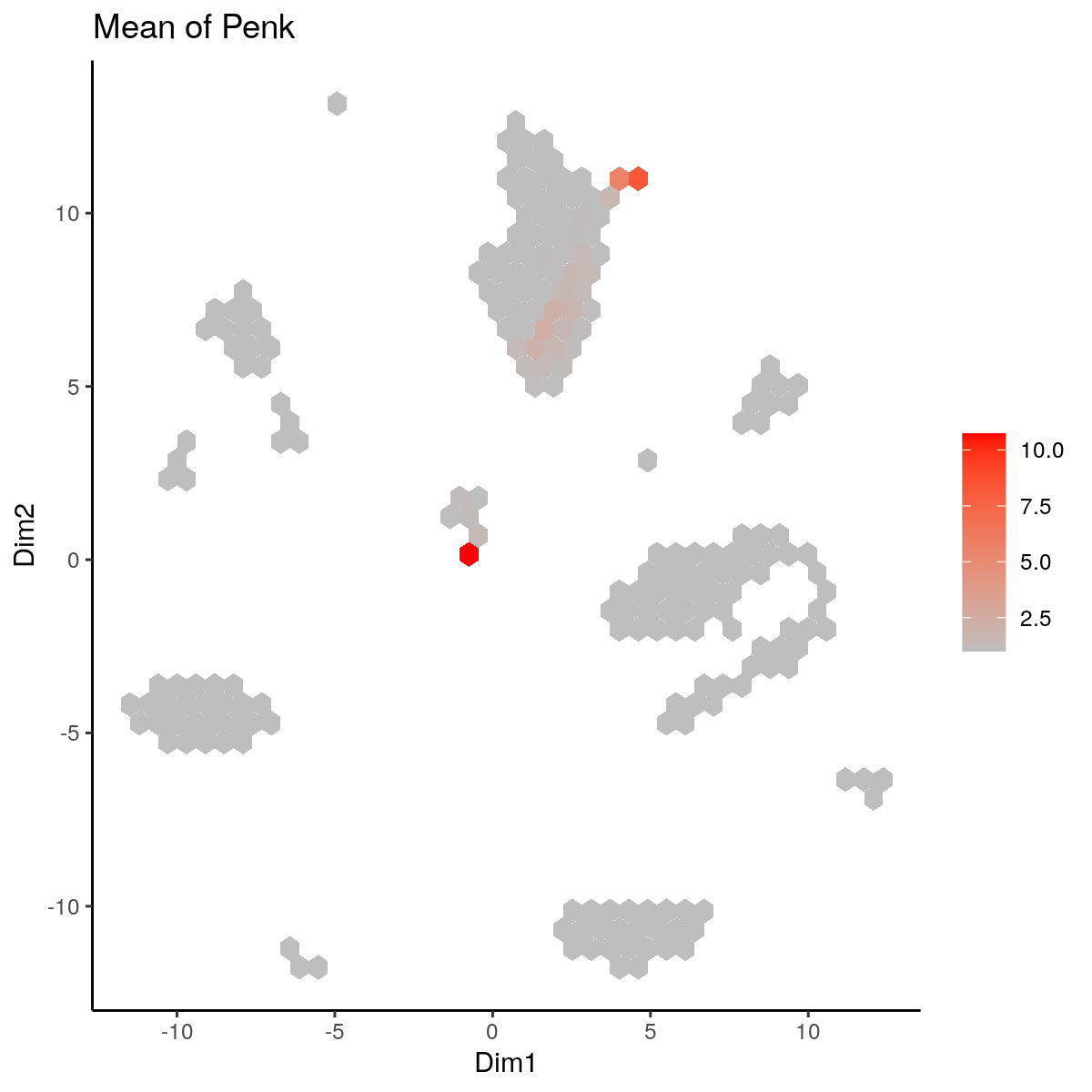

Supplement: Supplementary file 18 — Additional file 18. HTML report of VisualCortex. [file 12859_2023_5490_MOESM18_ESM.zip › output/report/Mouse_VisualCortex/figures/Ligand/18619.png]

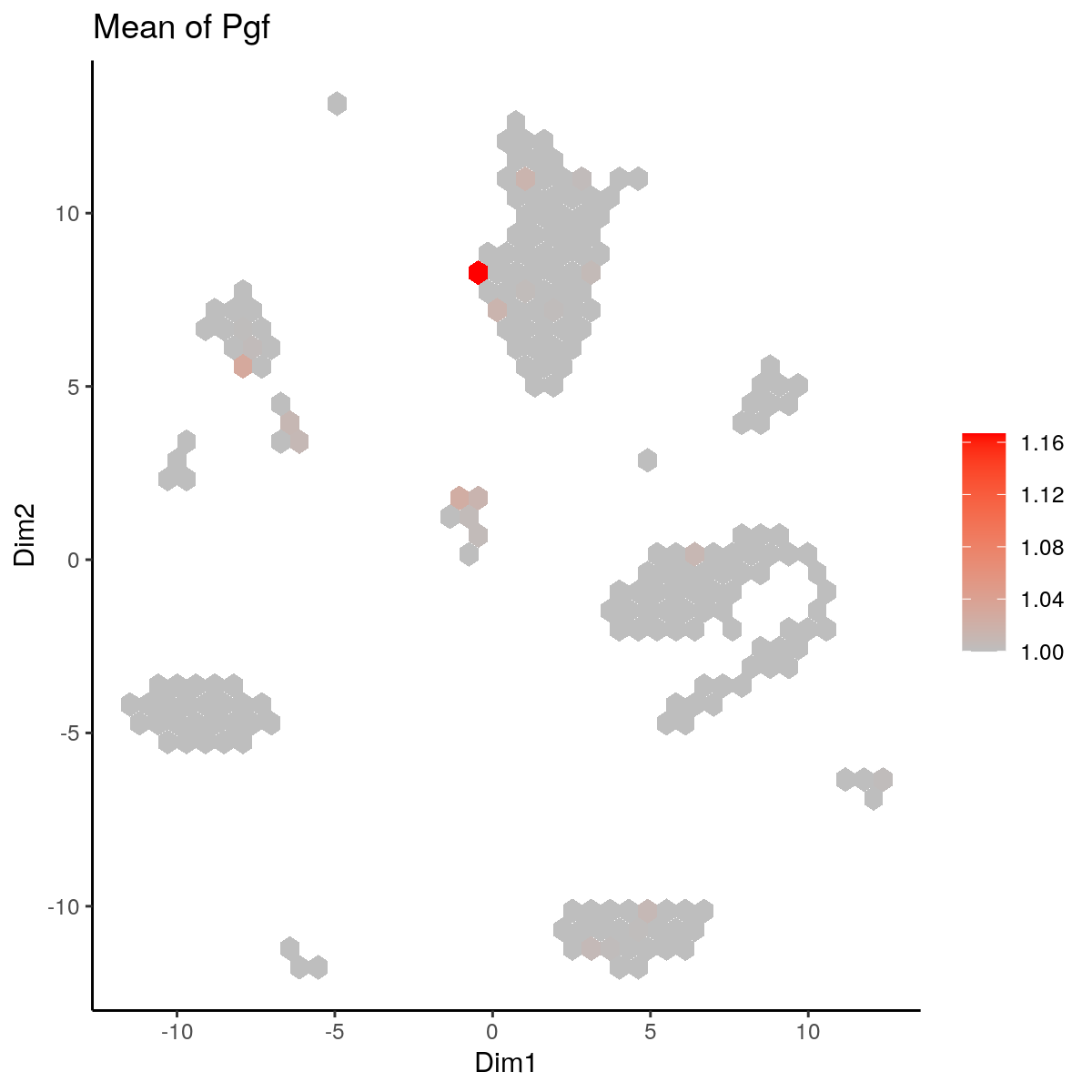

Supplement: Supplementary file 18 — Additional file 18. HTML report of VisualCortex. [file 12859_2023_5490_MOESM18_ESM.zip › output/report/Mouse_VisualCortex/figures/Ligand/18654.png]

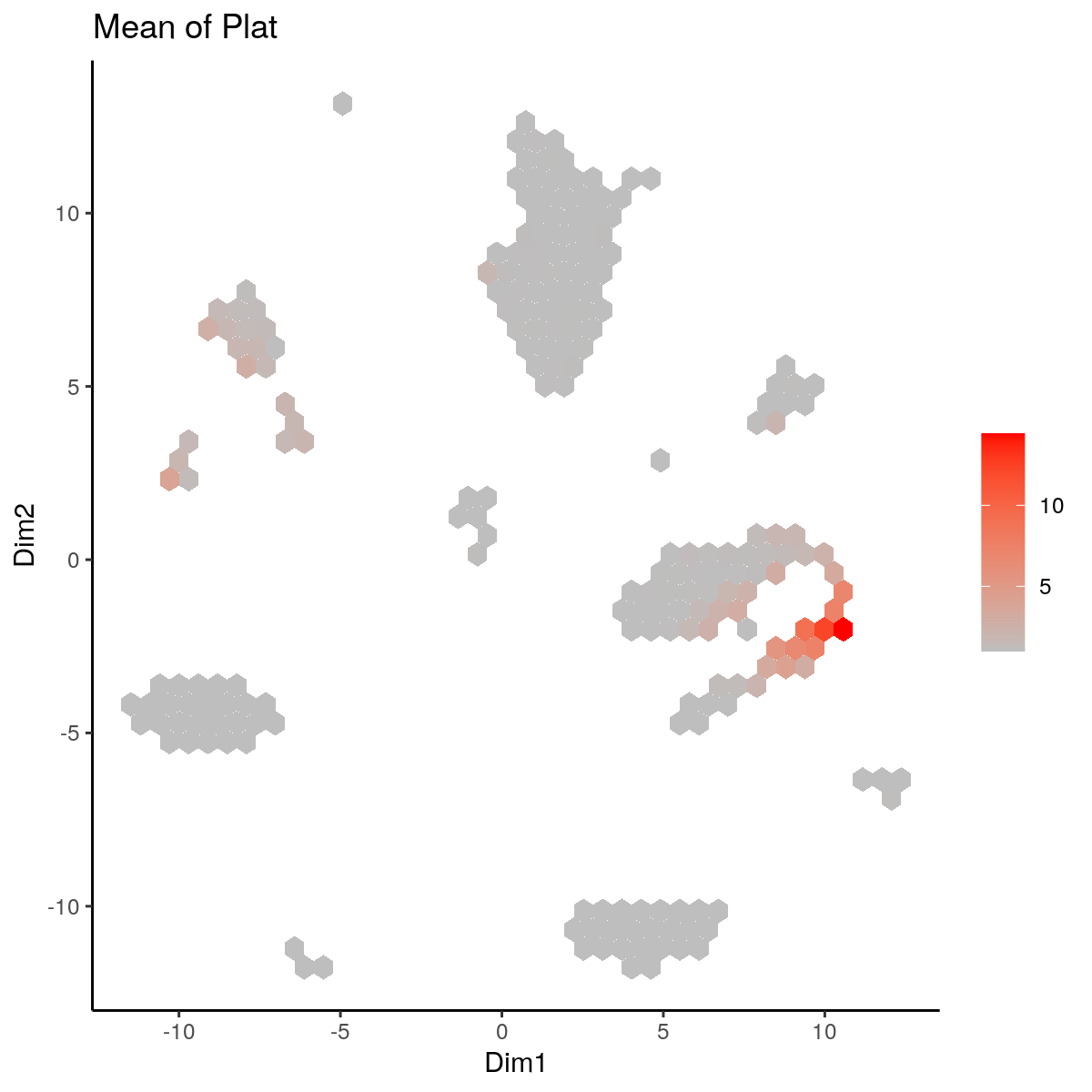

Supplement: Supplementary file 18 — Additional file 18. HTML report of VisualCortex. [file 12859_2023_5490_MOESM18_ESM.zip › output/report/Mouse_VisualCortex/figures/Ligand/18791.png]

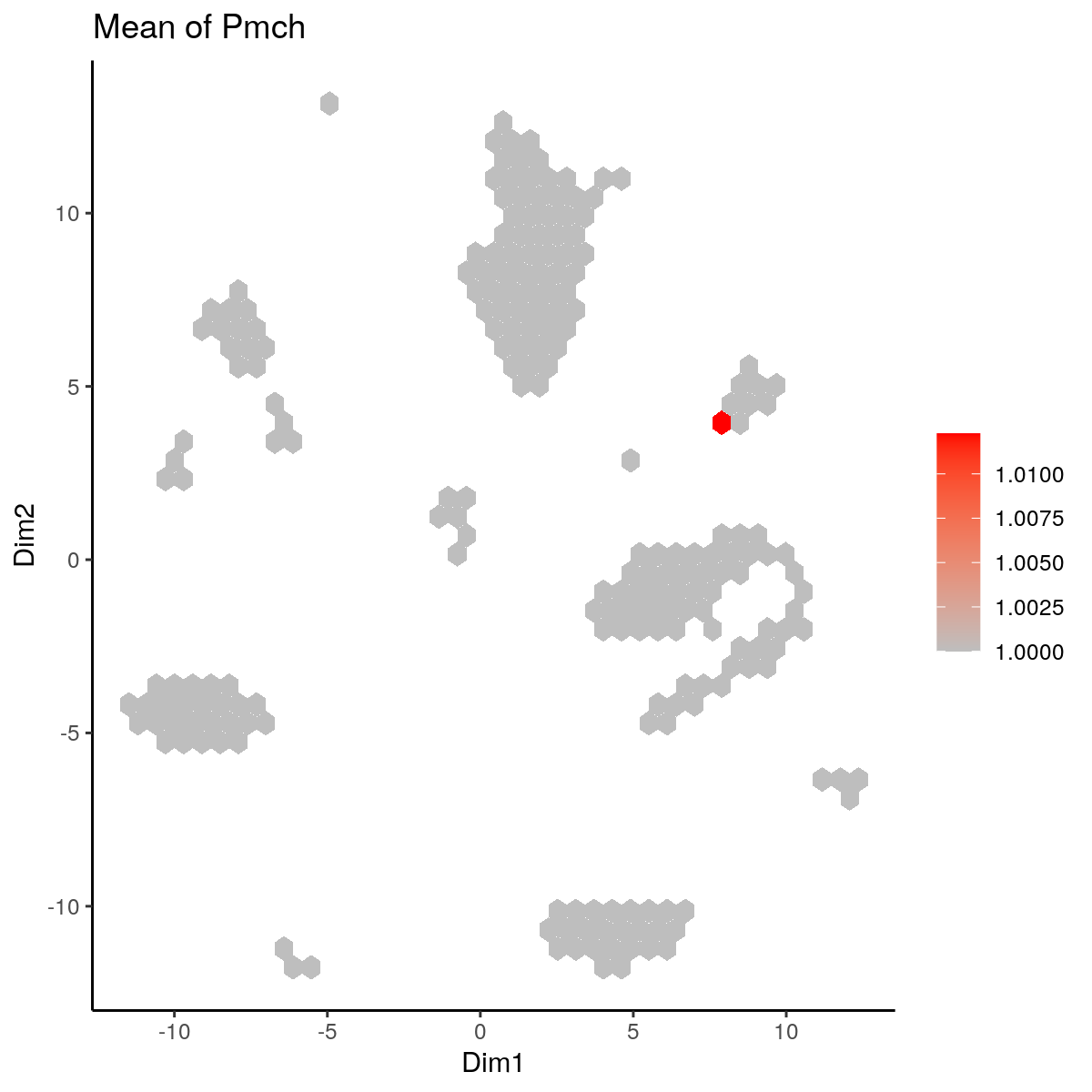

Supplement: Supplementary file 18 — Additional file 18. HTML report of VisualCortex. [file 12859_2023_5490_MOESM18_ESM.zip › output/report/Mouse_VisualCortex/figures/Ligand/110312.png]

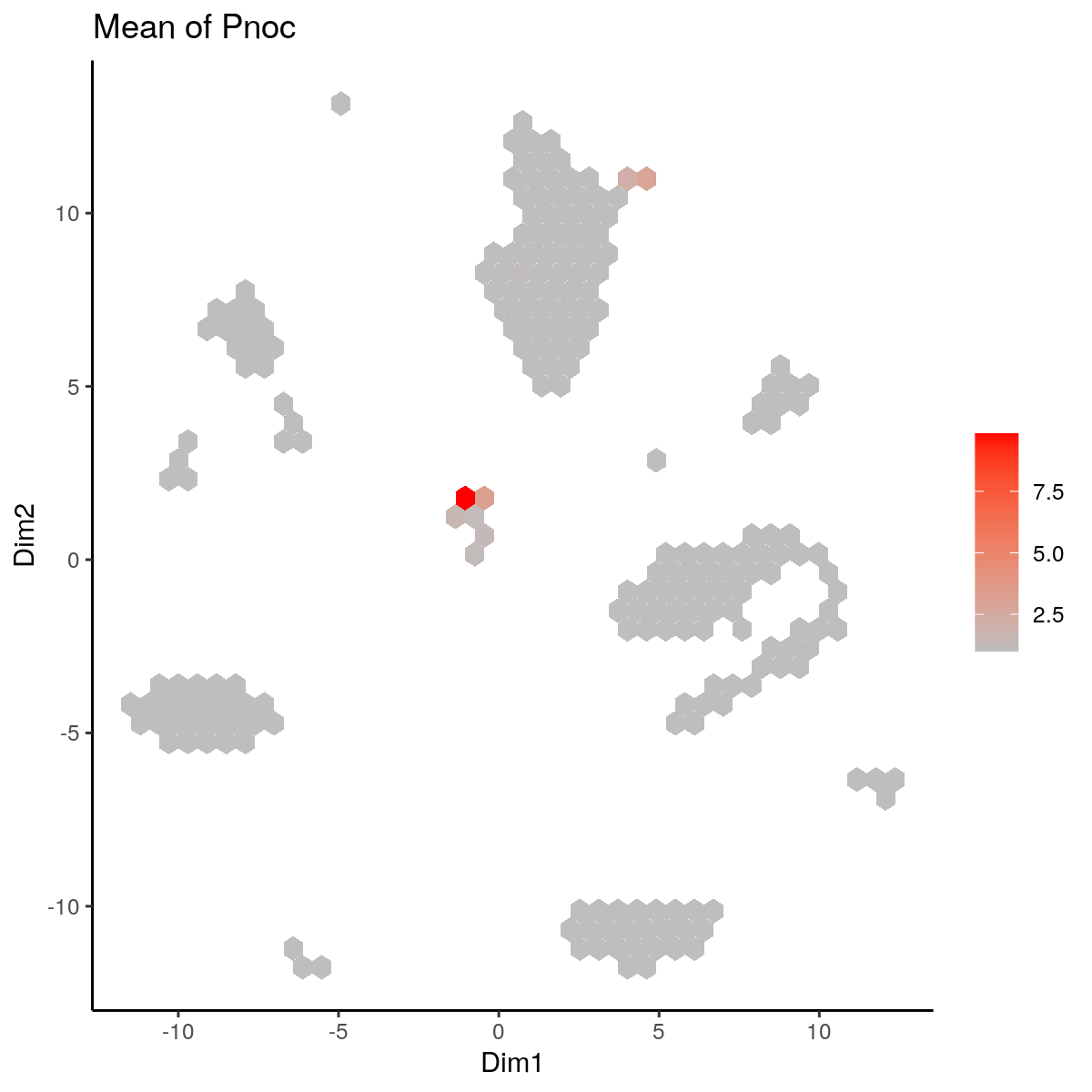

Supplement: Supplementary file 18 — Additional file 18. HTML report of VisualCortex. [file 12859_2023_5490_MOESM18_ESM.zip › output/report/Mouse_VisualCortex/figures/Ligand/18155.png]

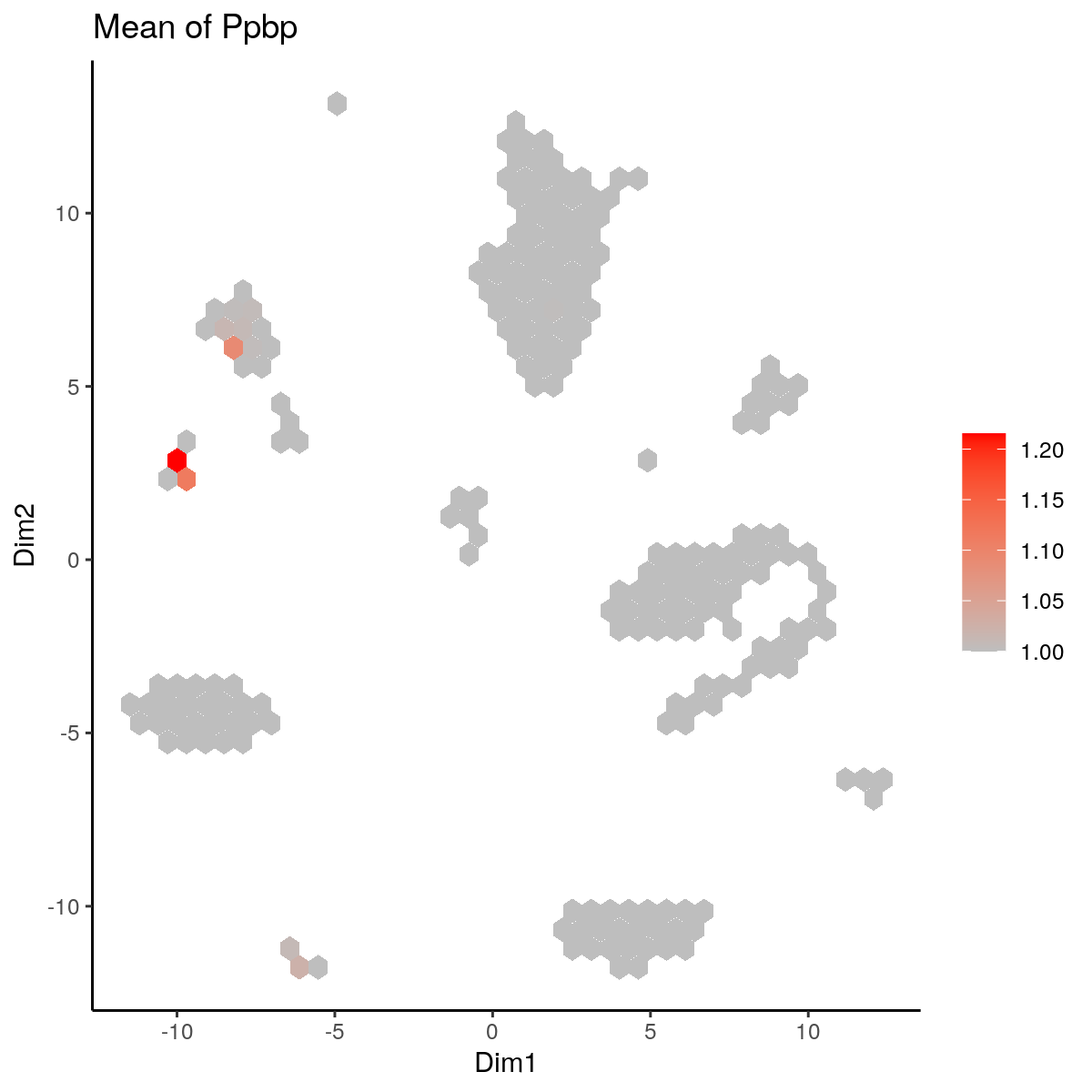

Supplement: Supplementary file 18 — Additional file 18. HTML report of VisualCortex. [file 12859_2023_5490_MOESM18_ESM.zip › output/report/Mouse_VisualCortex/figures/Ligand/57349.png]

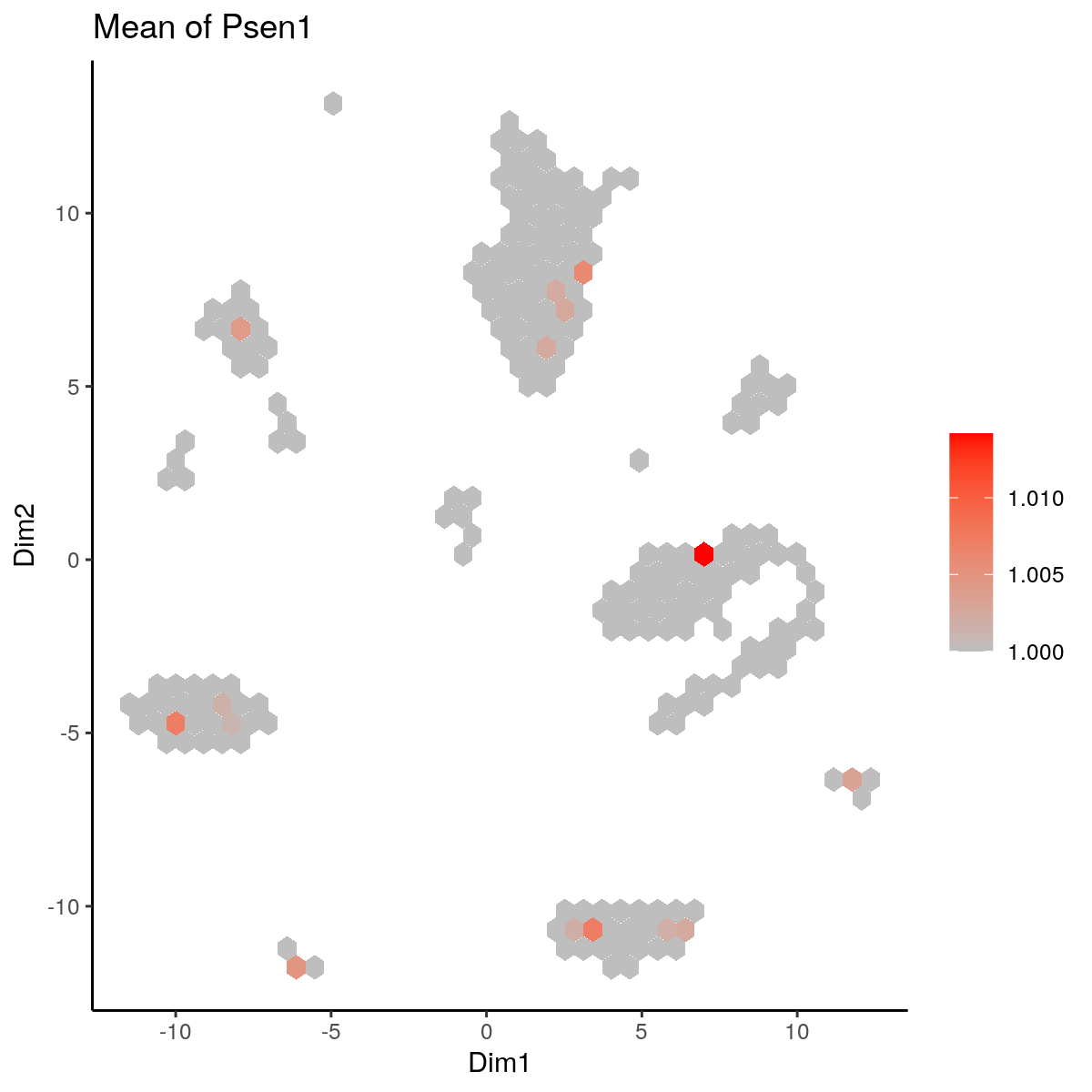

Supplement: Supplementary file 18 — Additional file 18. HTML report of VisualCortex. [file 12859_2023_5490_MOESM18_ESM.zip › output/report/Mouse_VisualCortex/figures/Ligand/19164.png]

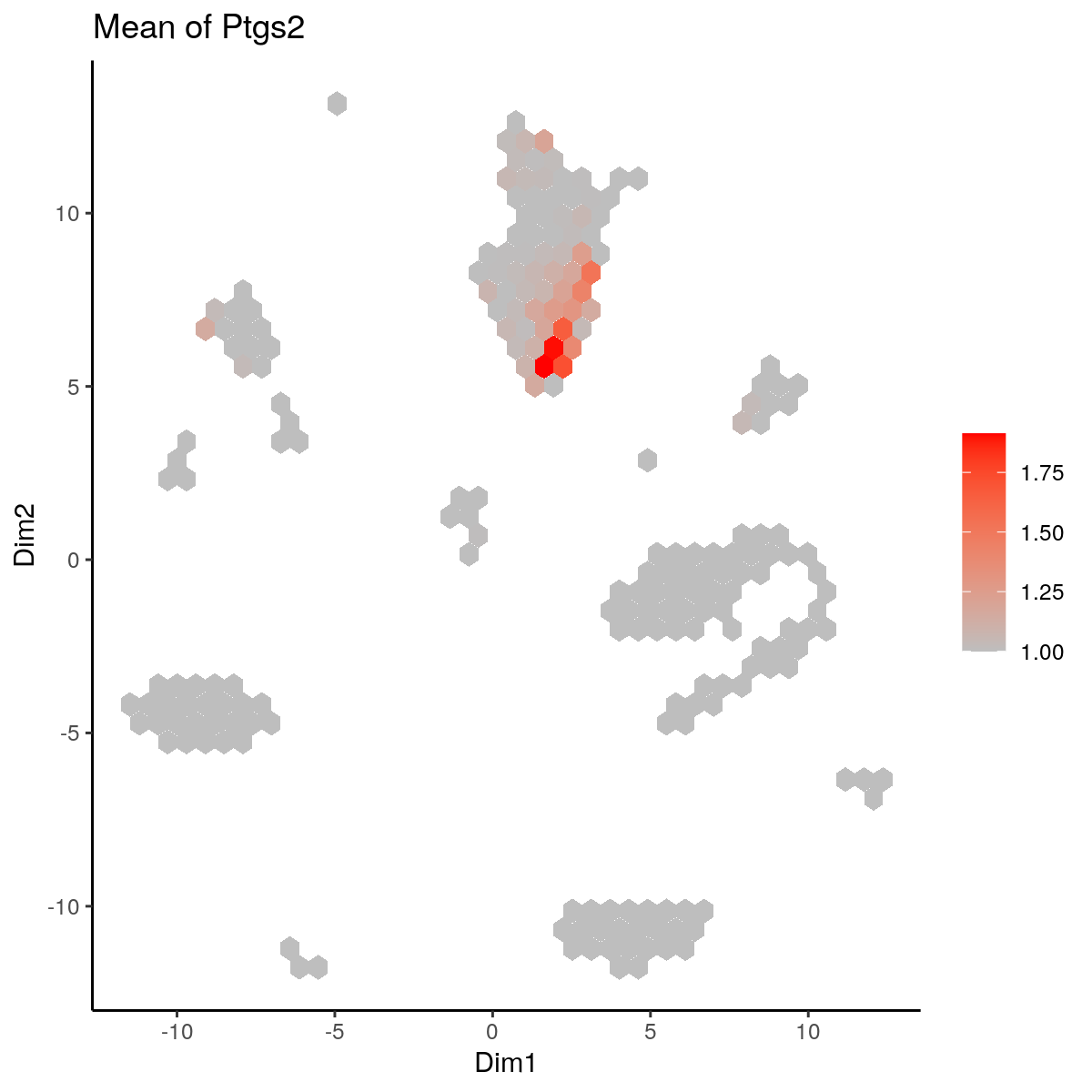

Supplement: Supplementary file 18 — Additional file 18. HTML report of VisualCortex. [file 12859_2023_5490_MOESM18_ESM.zip › output/report/Mouse_VisualCortex/figures/Ligand/19225.png]

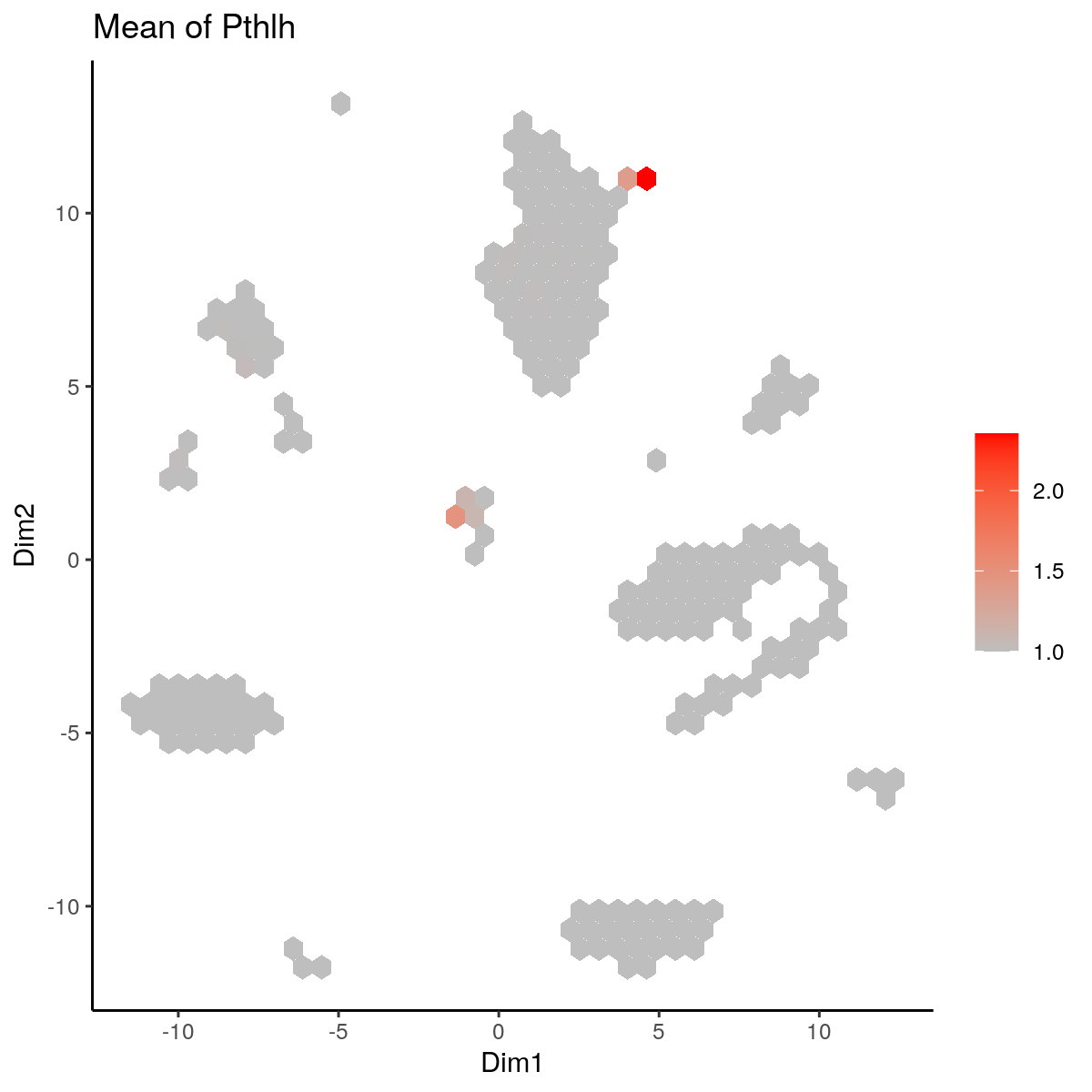

Supplement: Supplementary file 18 — Additional file 18. HTML report of VisualCortex. [file 12859_2023_5490_MOESM18_ESM.zip › output/report/Mouse_VisualCortex/figures/Ligand/19227.png]

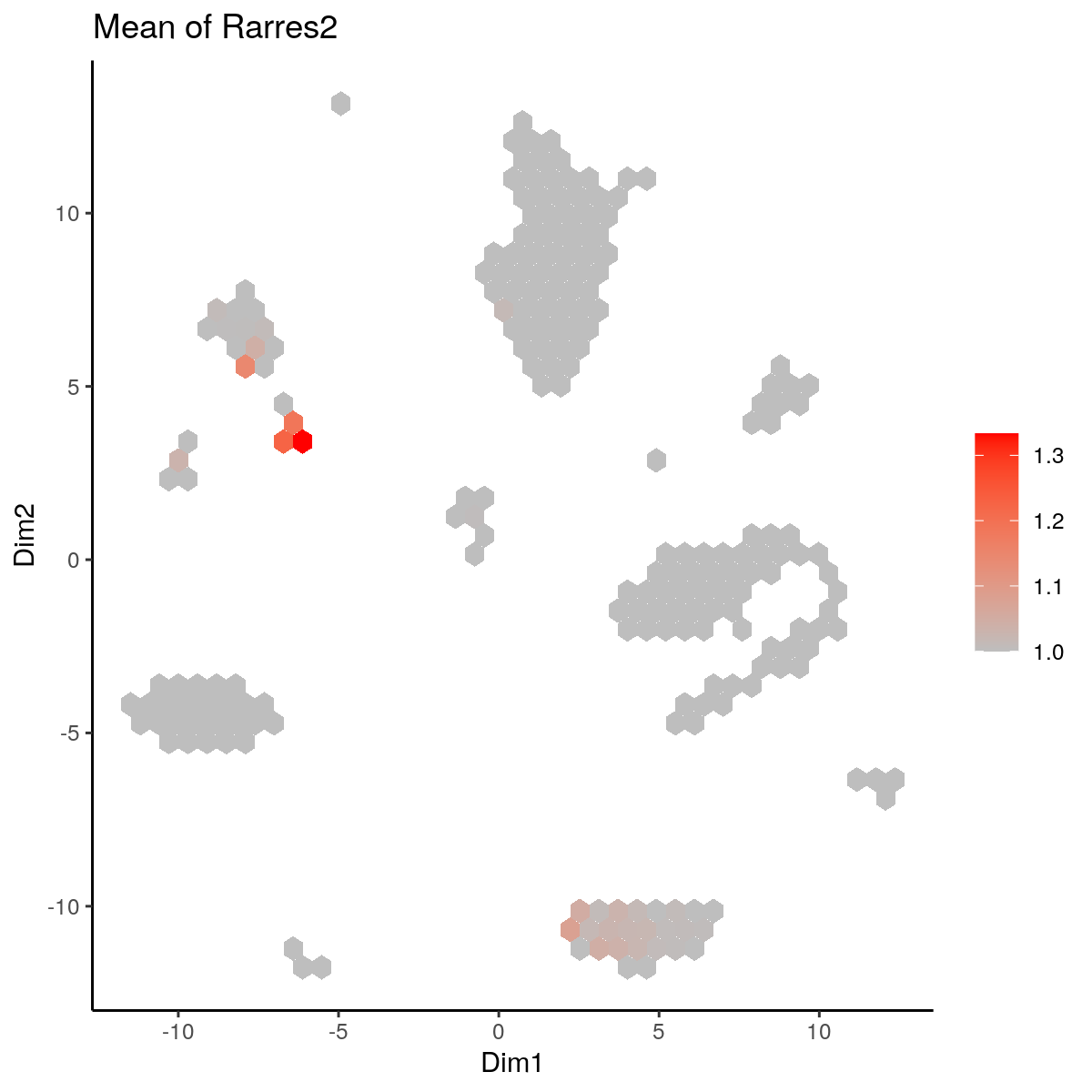

Supplement: Supplementary file 18 — Additional file 18. HTML report of VisualCortex. [file 12859_2023_5490_MOESM18_ESM.zip › output/report/Mouse_VisualCortex/figures/Ligand/71660.png]

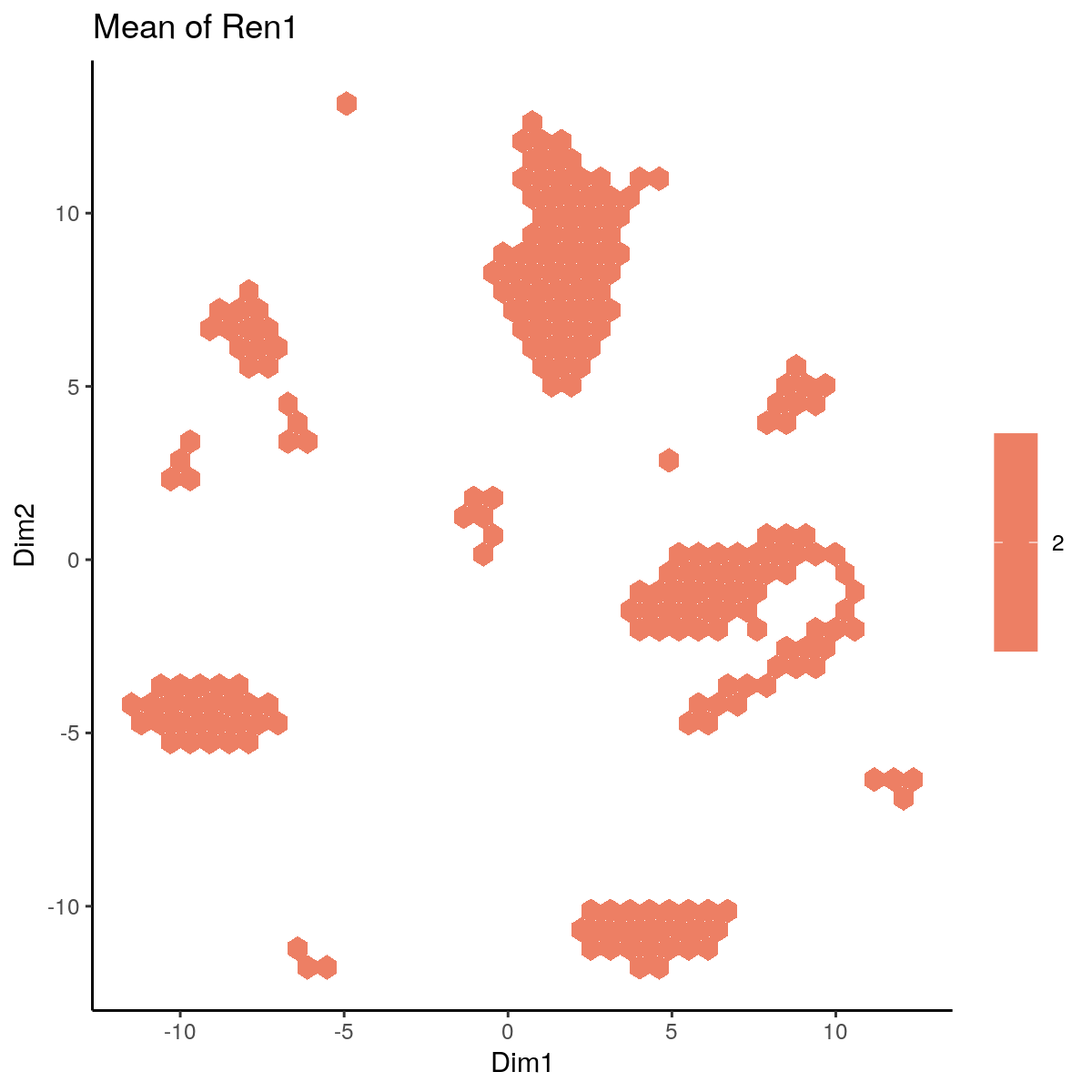

Supplement: Supplementary file 18 — Additional file 18. HTML report of VisualCortex. [file 12859_2023_5490_MOESM18_ESM.zip › output/report/Mouse_VisualCortex/figures/Ligand/19701.png]

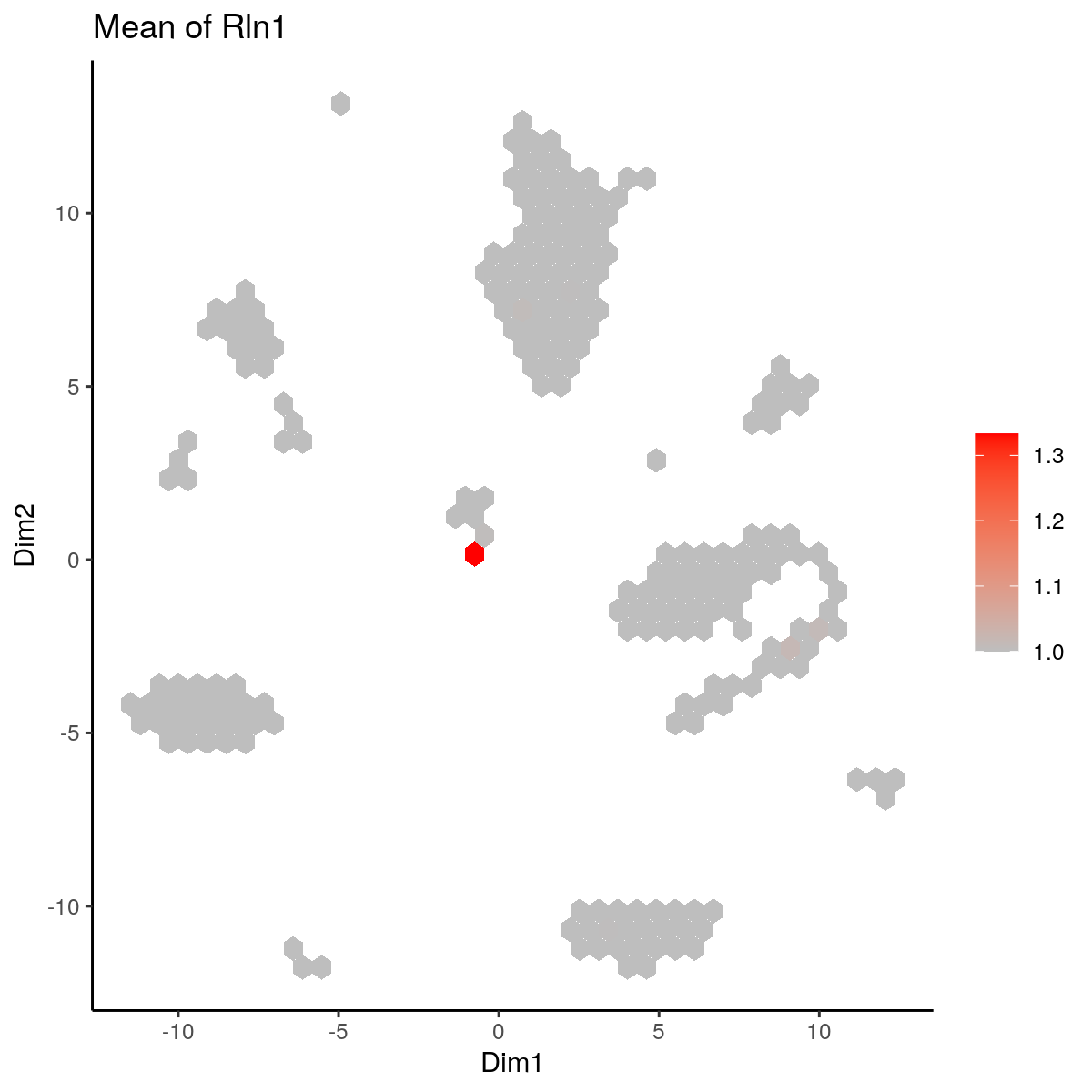

Supplement: Supplementary file 18 — Additional file 18. HTML report of VisualCortex. [file 12859_2023_5490_MOESM18_ESM.zip › output/report/Mouse_VisualCortex/figures/Ligand/19773.png]

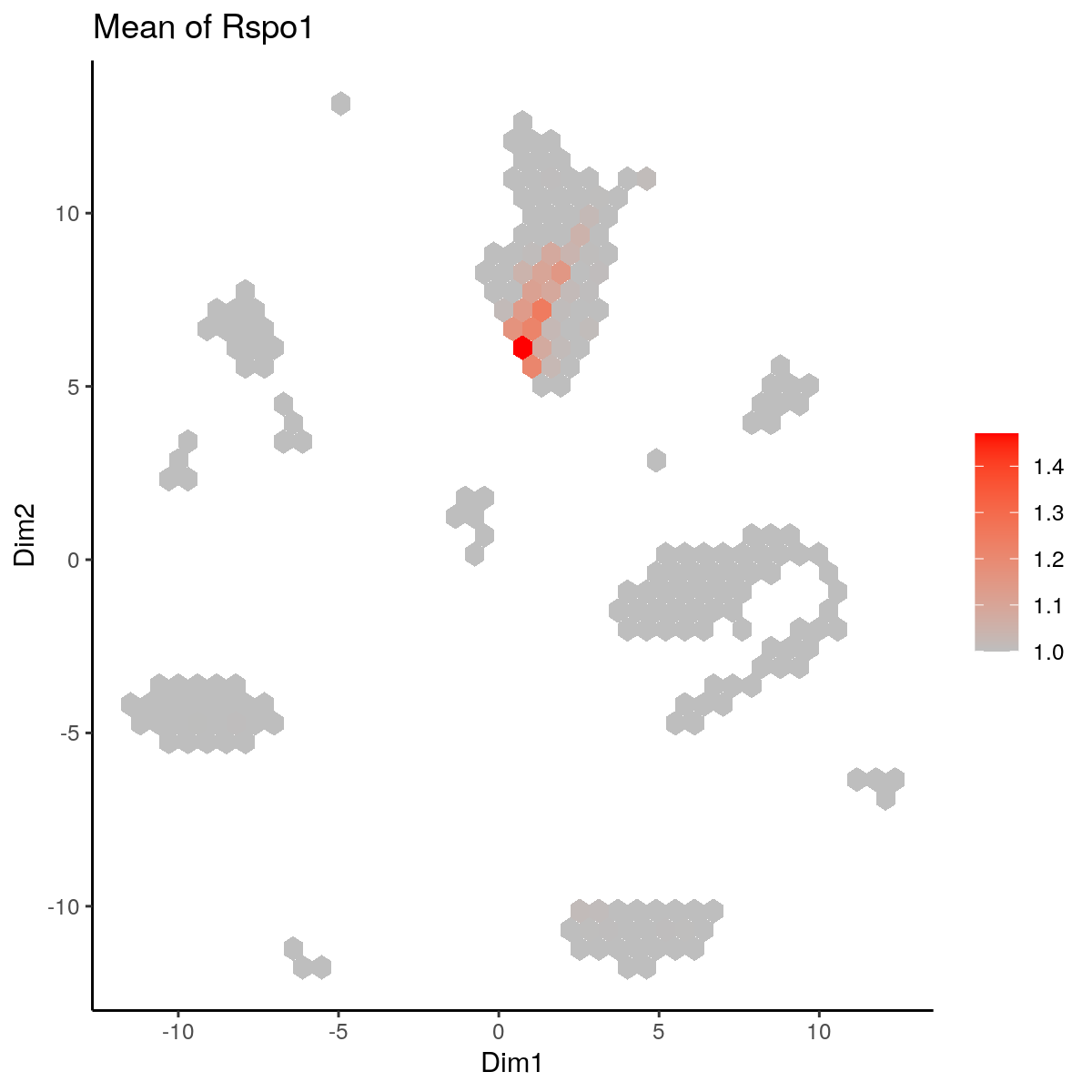

Supplement: Supplementary file 18 — Additional file 18. HTML report of VisualCortex. [file 12859_2023_5490_MOESM18_ESM.zip › output/report/Mouse_VisualCortex/figures/Ligand/192199.png]

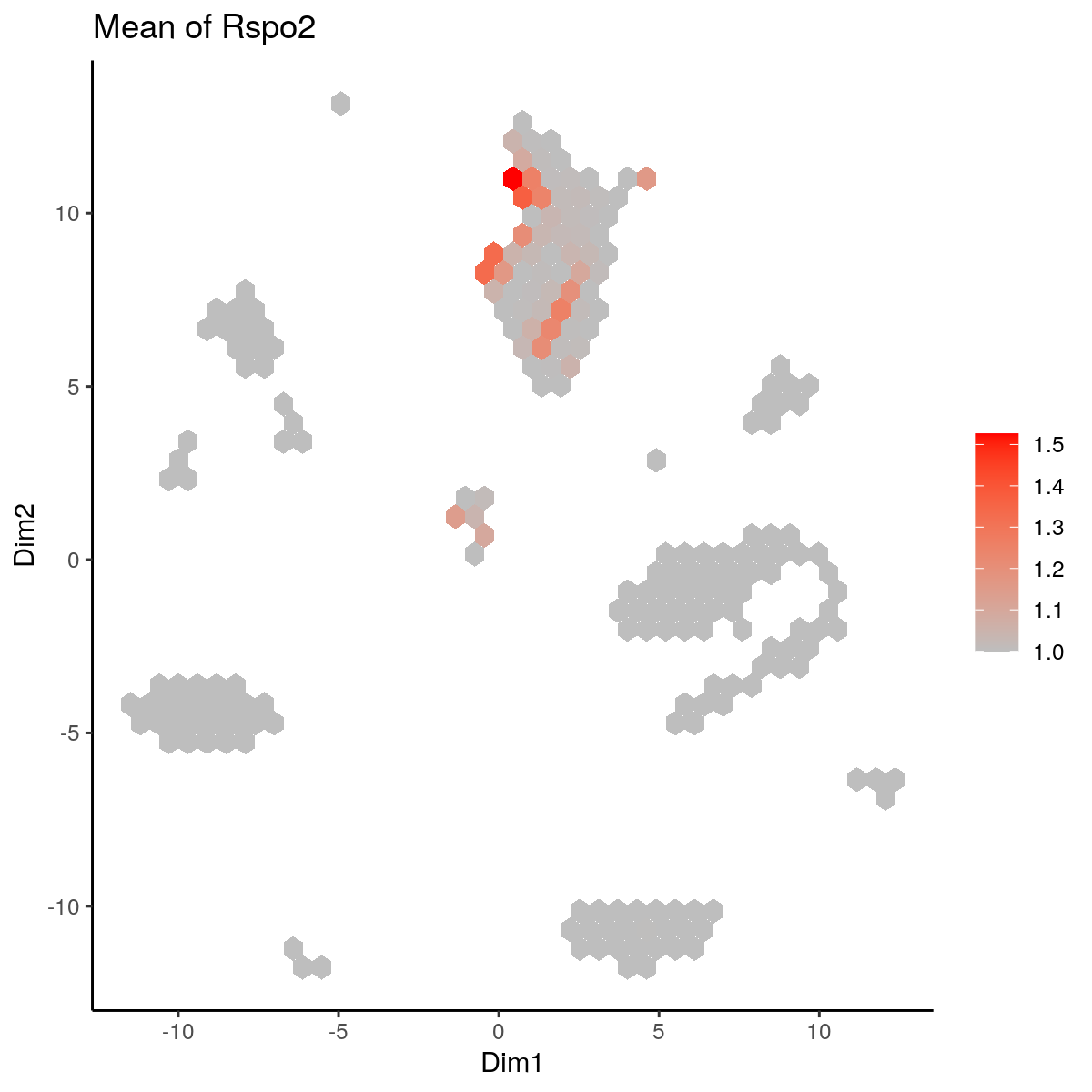

Supplement: Supplementary file 18 — Additional file 18. HTML report of VisualCortex. [file 12859_2023_5490_MOESM18_ESM.zip › output/report/Mouse_VisualCortex/figures/Ligand/239405.png]

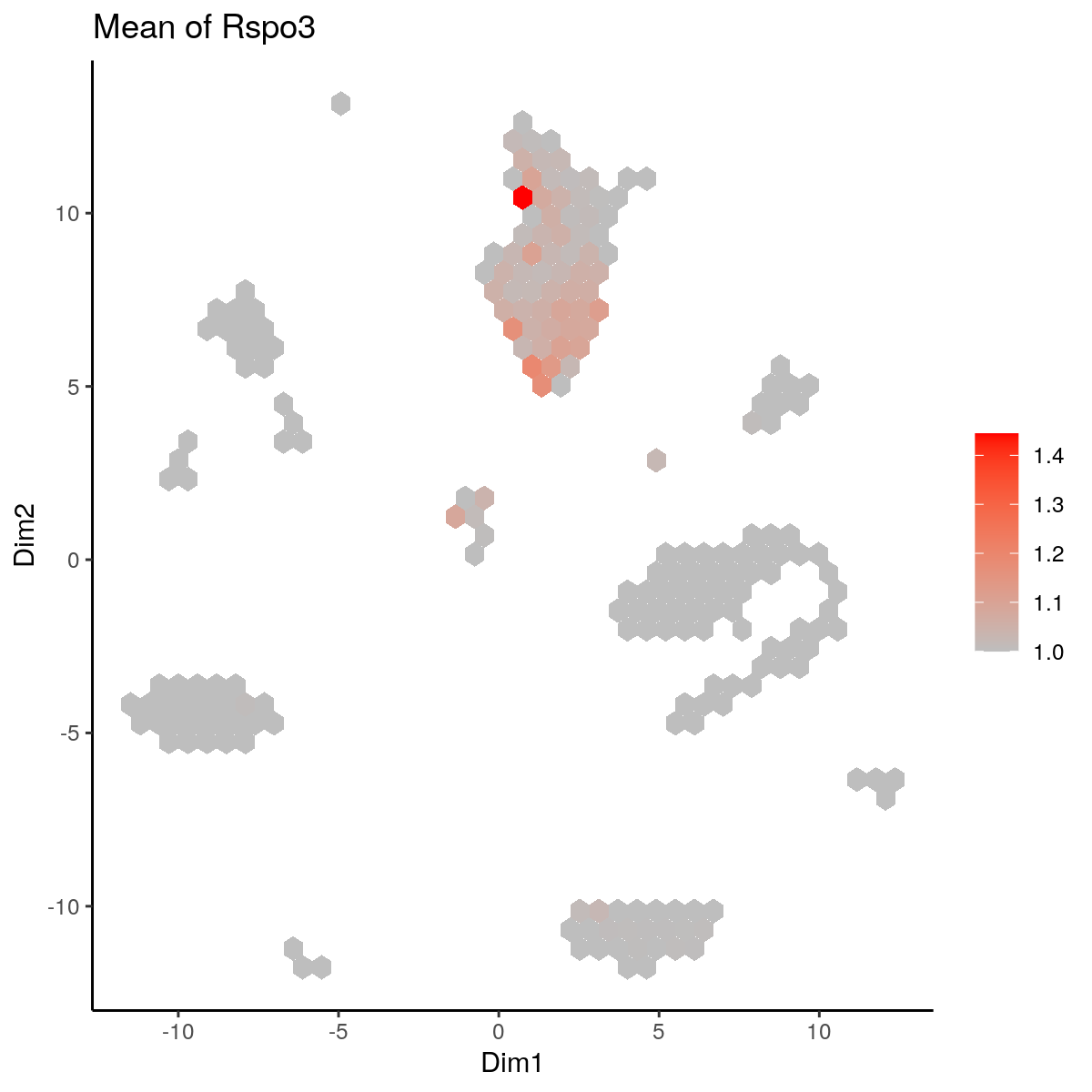

Supplement: Supplementary file 18 — Additional file 18. HTML report of VisualCortex. [file 12859_2023_5490_MOESM18_ESM.zip › output/report/Mouse_VisualCortex/figures/Ligand/72780.png]

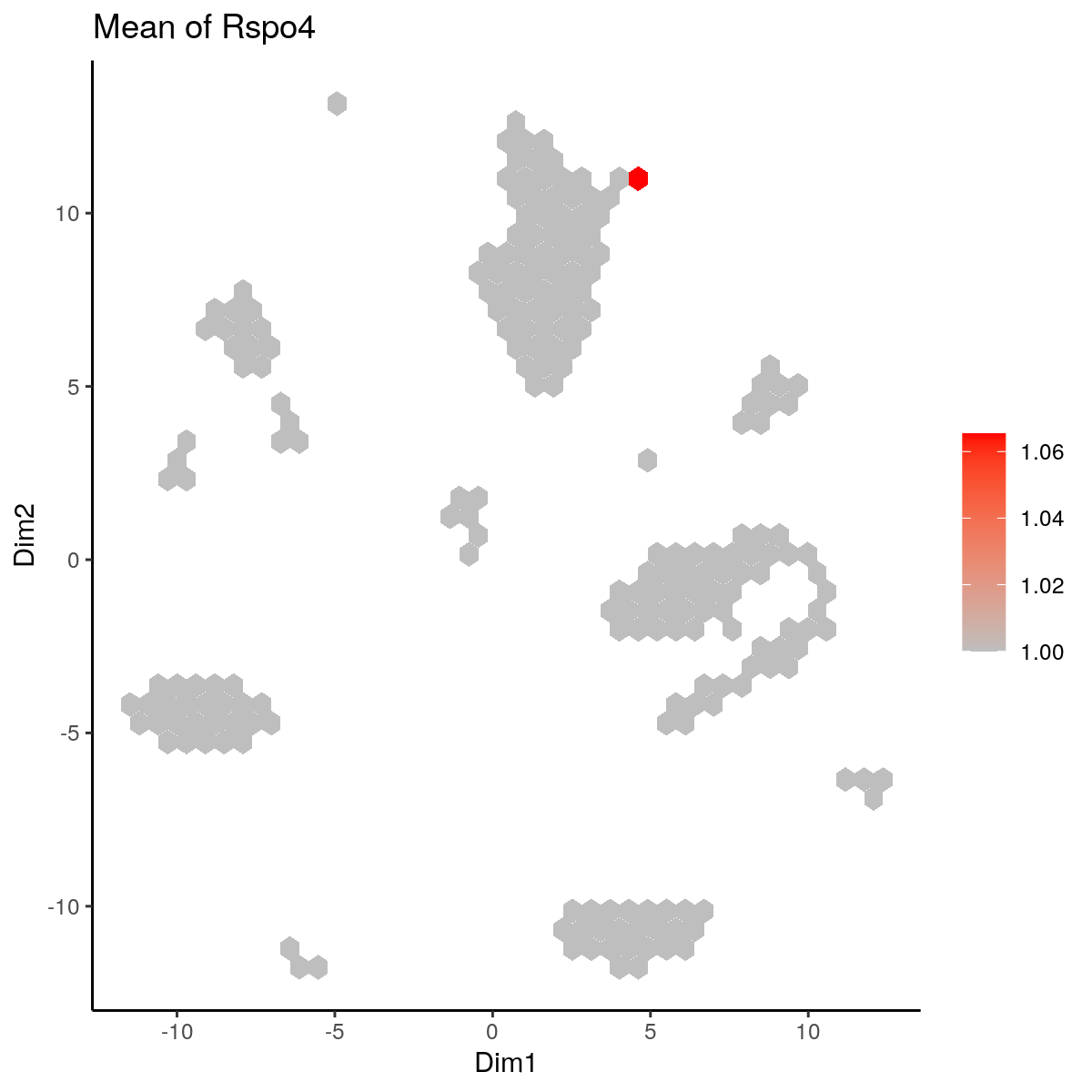

Supplement: Supplementary file 18 — Additional file 18. HTML report of VisualCortex. [file 12859_2023_5490_MOESM18_ESM.zip › output/report/Mouse_VisualCortex/figures/Ligand/228770.png]

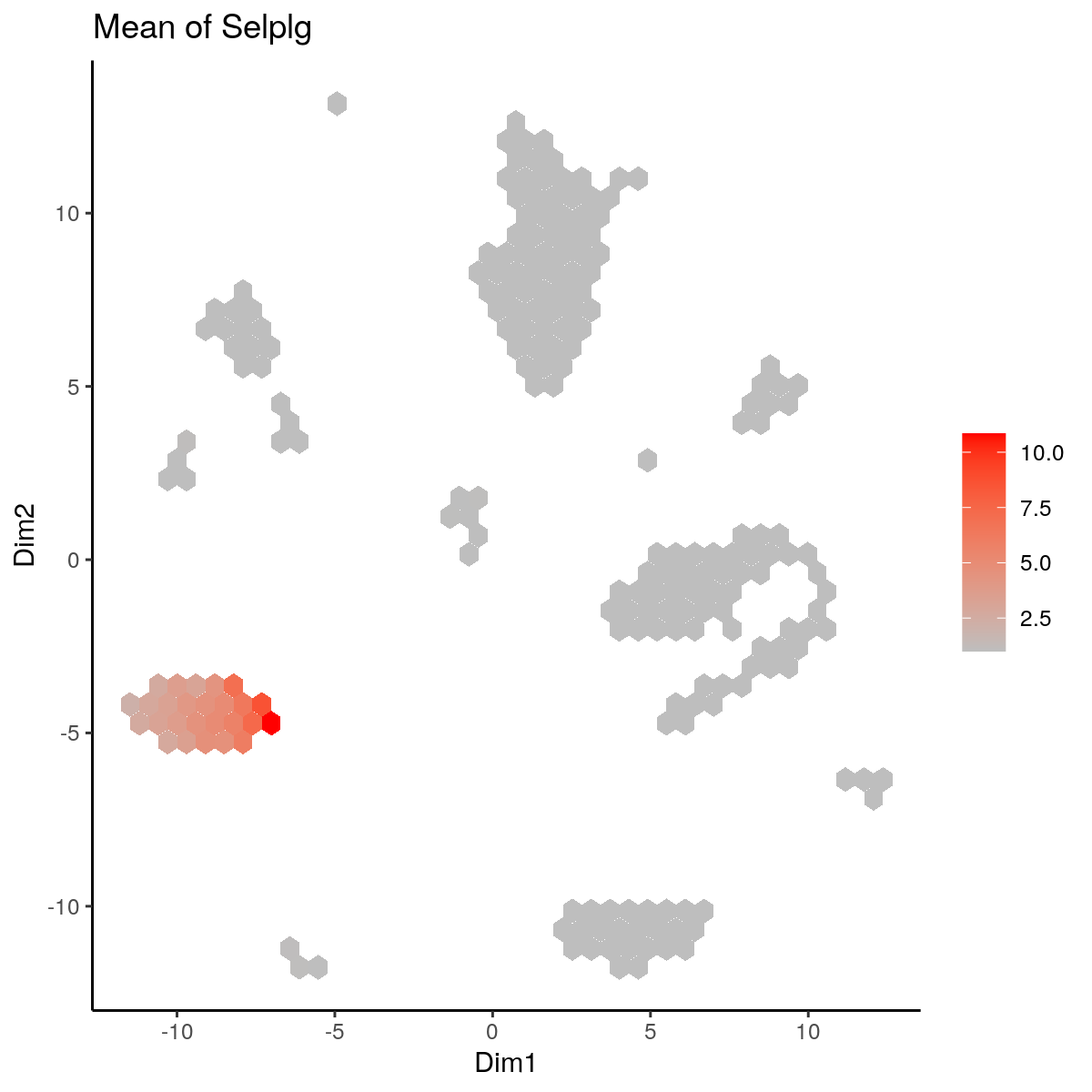

Supplement: Supplementary file 18 — Additional file 18. HTML report of VisualCortex. [file 12859_2023_5490_MOESM18_ESM.zip › output/report/Mouse_VisualCortex/figures/Ligand/20345.png]

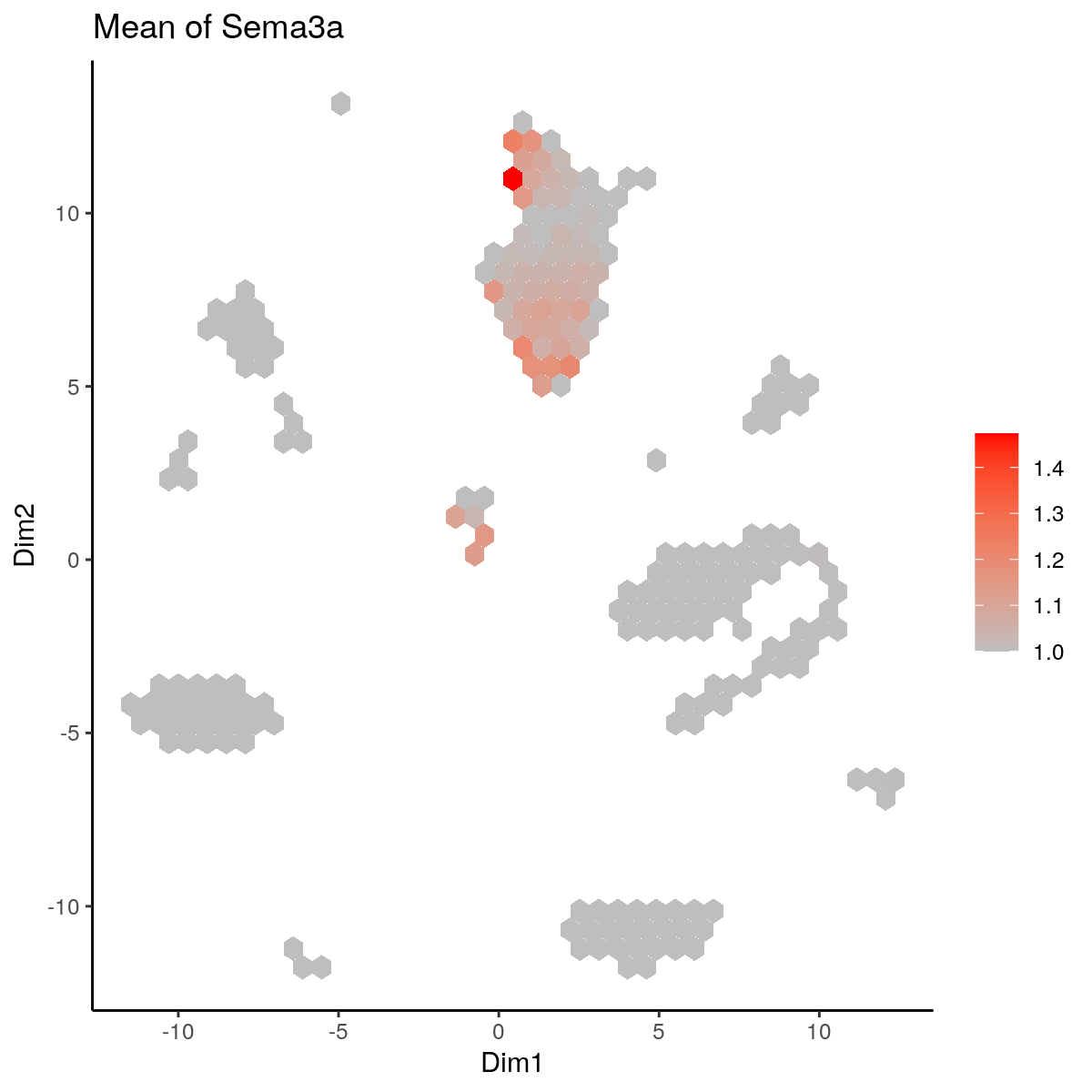

Supplement: Supplementary file 18 — Additional file 18. HTML report of VisualCortex. [file 12859_2023_5490_MOESM18_ESM.zip › output/report/Mouse_VisualCortex/figures/Ligand/20346.png]

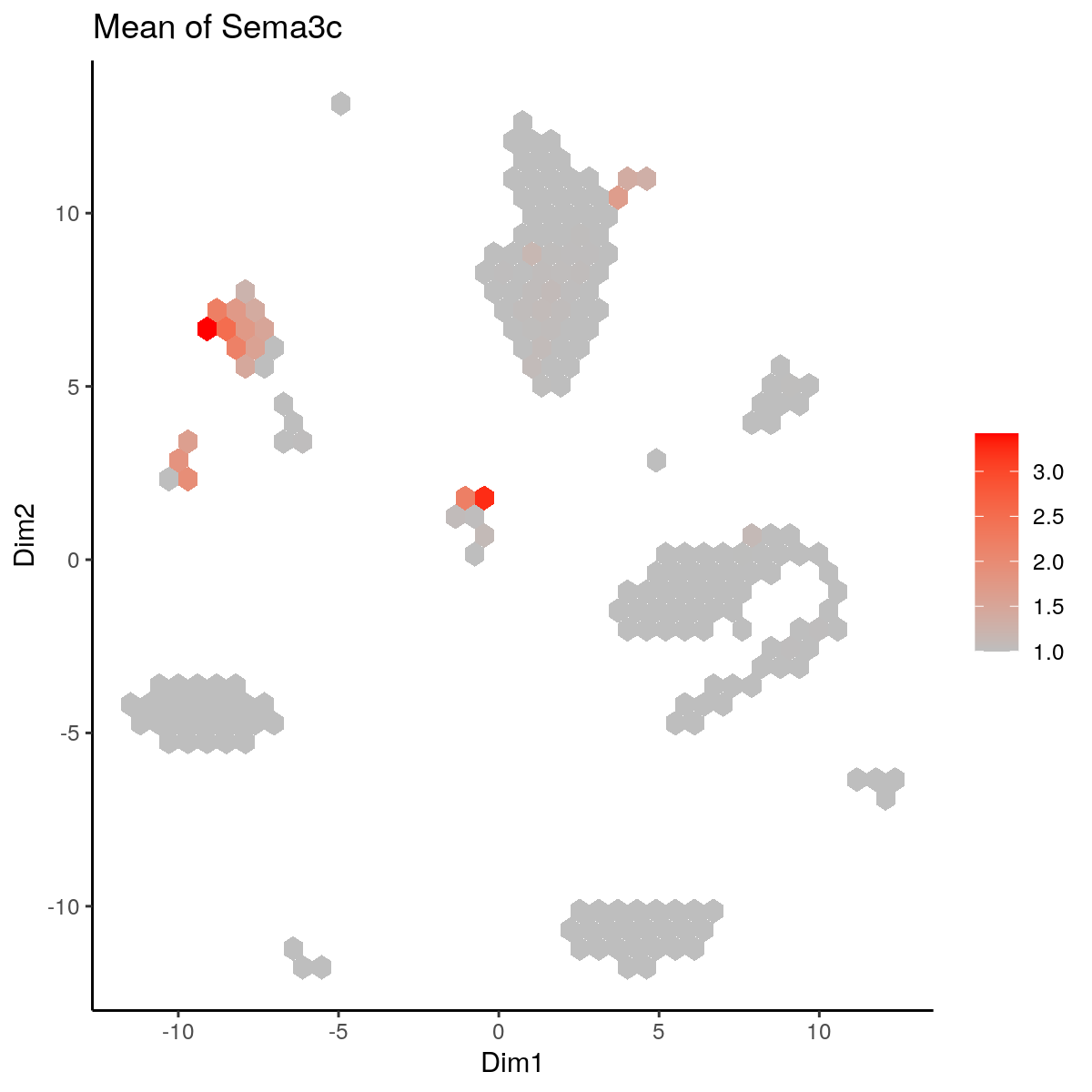

Supplement: Supplementary file 18 — Additional file 18. HTML report of VisualCortex. [file 12859_2023_5490_MOESM18_ESM.zip › output/report/Mouse_VisualCortex/figures/Ligand/20348.png]

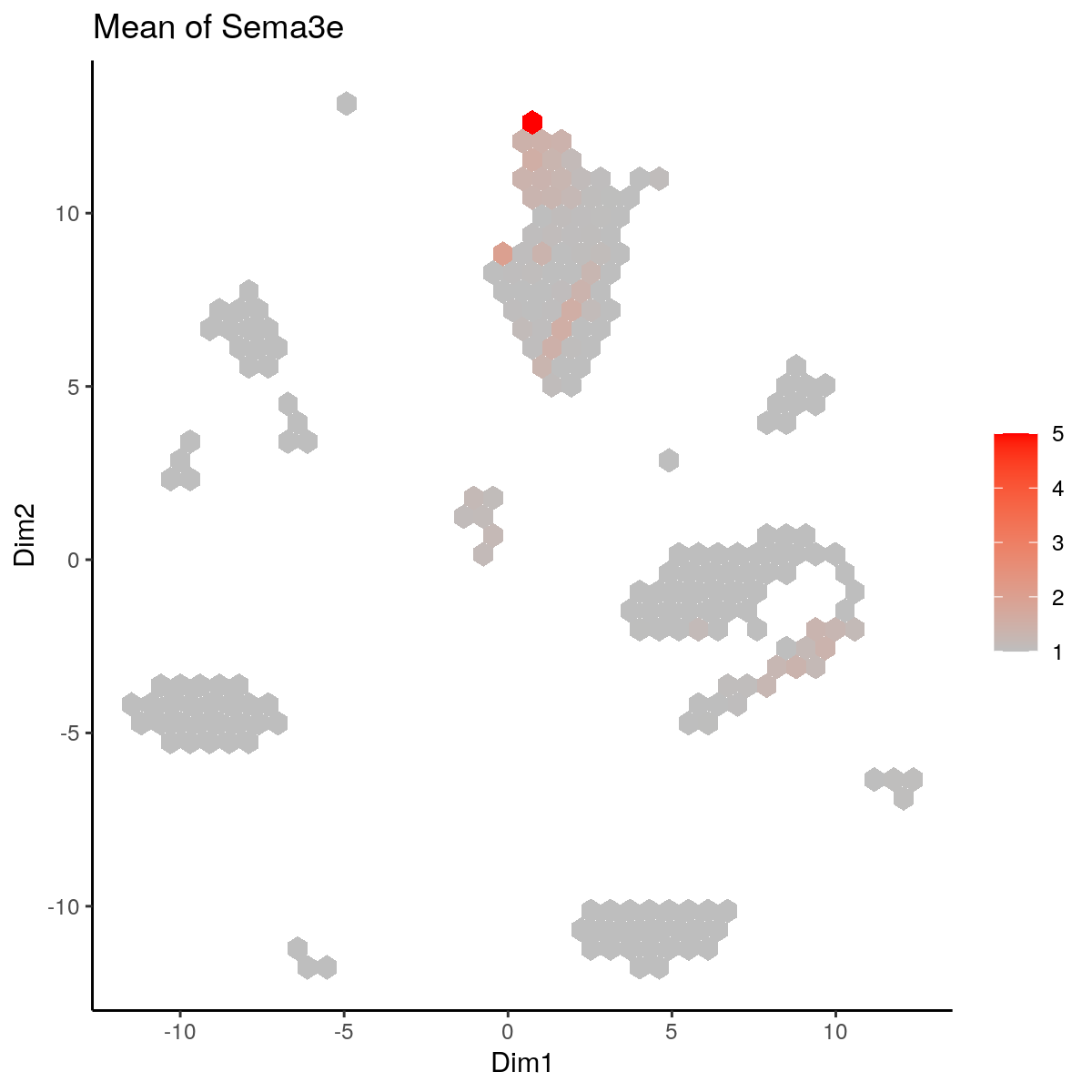

Supplement: Supplementary file 18 — Additional file 18. HTML report of VisualCortex. [file 12859_2023_5490_MOESM18_ESM.zip › output/report/Mouse_VisualCortex/figures/Ligand/20349.png]

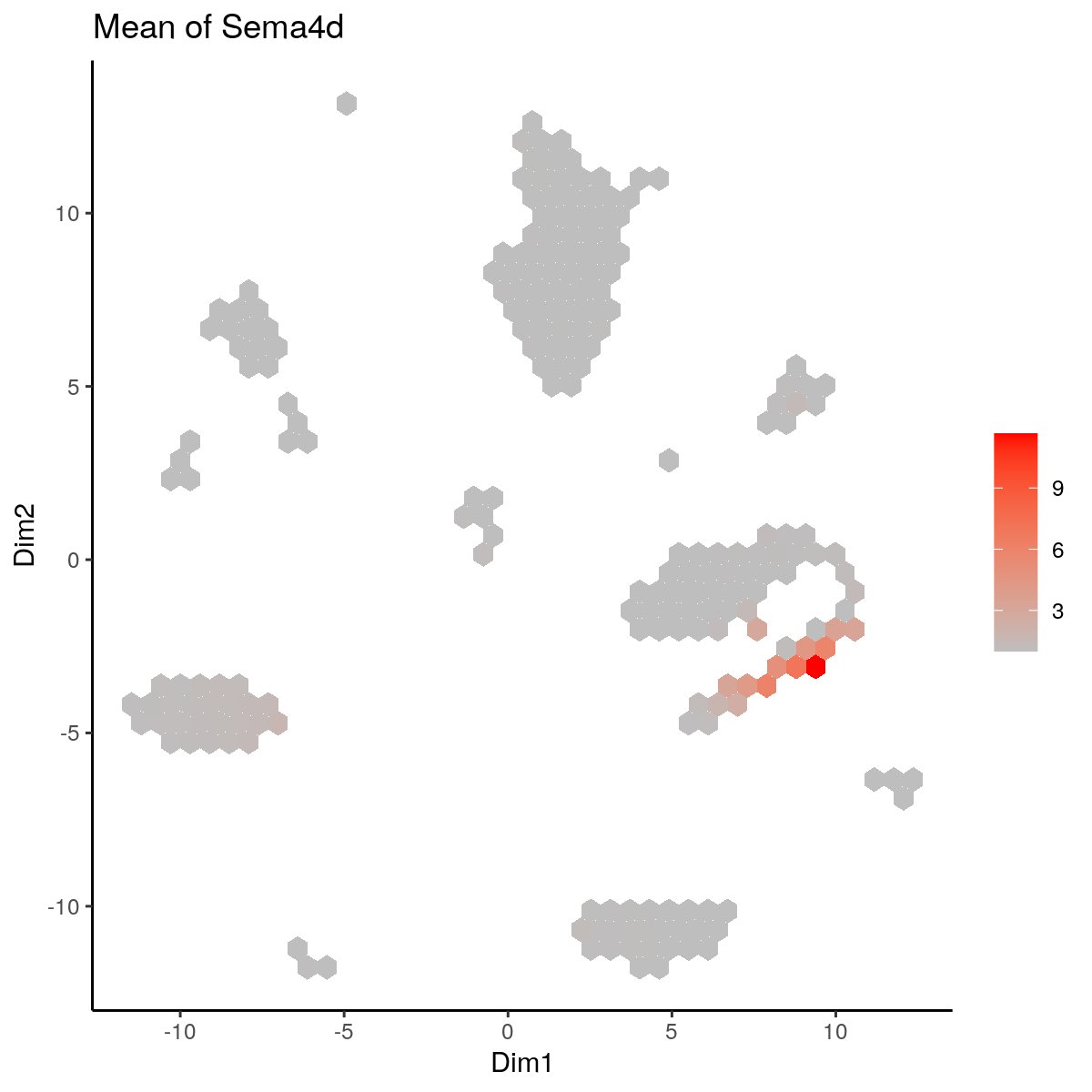

Supplement: Supplementary file 18 — Additional file 18. HTML report of VisualCortex. [file 12859_2023_5490_MOESM18_ESM.zip › output/report/Mouse_VisualCortex/figures/Ligand/20354.png]

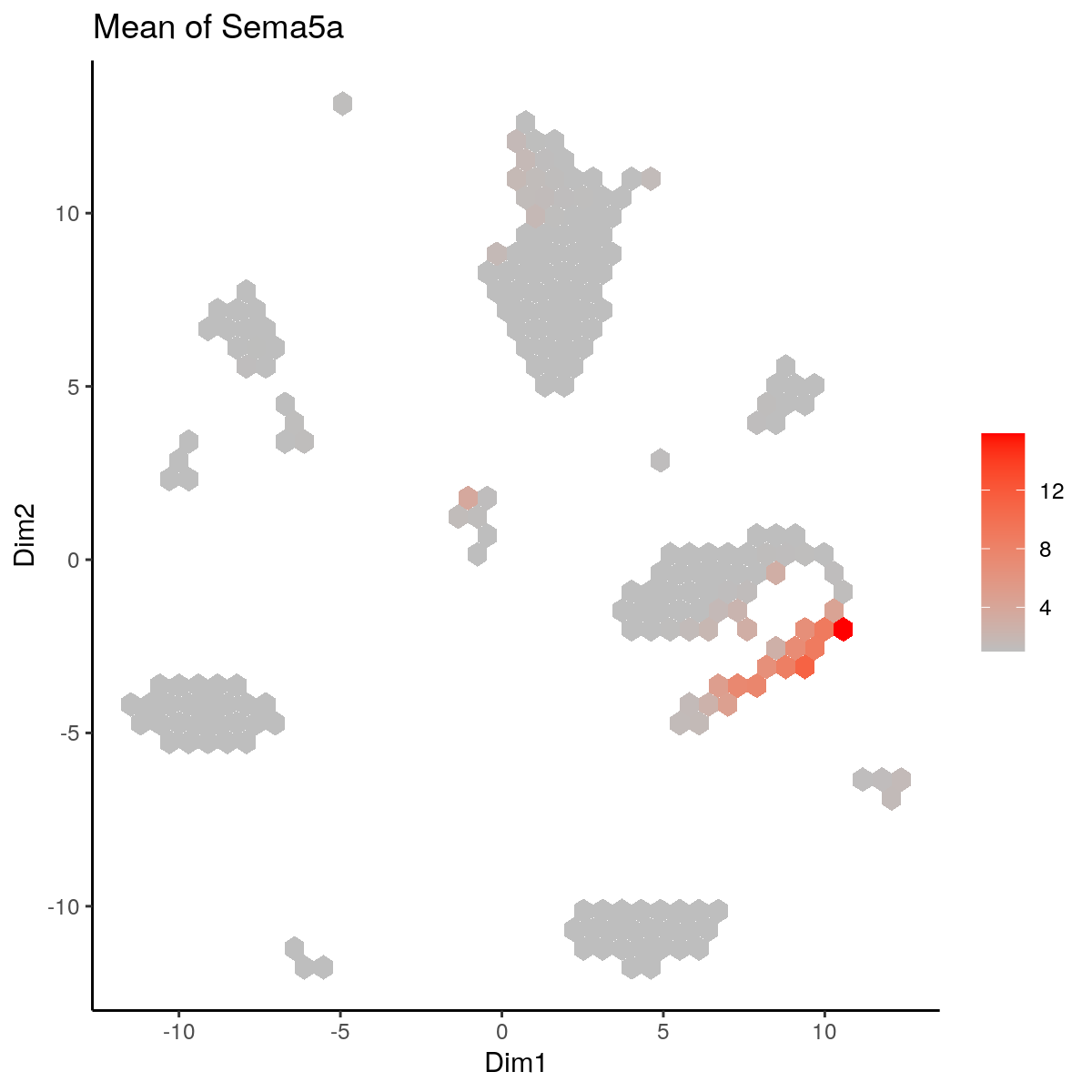

Supplement: Supplementary file 18 — Additional file 18. HTML report of VisualCortex. [file 12859_2023_5490_MOESM18_ESM.zip › output/report/Mouse_VisualCortex/figures/Ligand/20356.png]

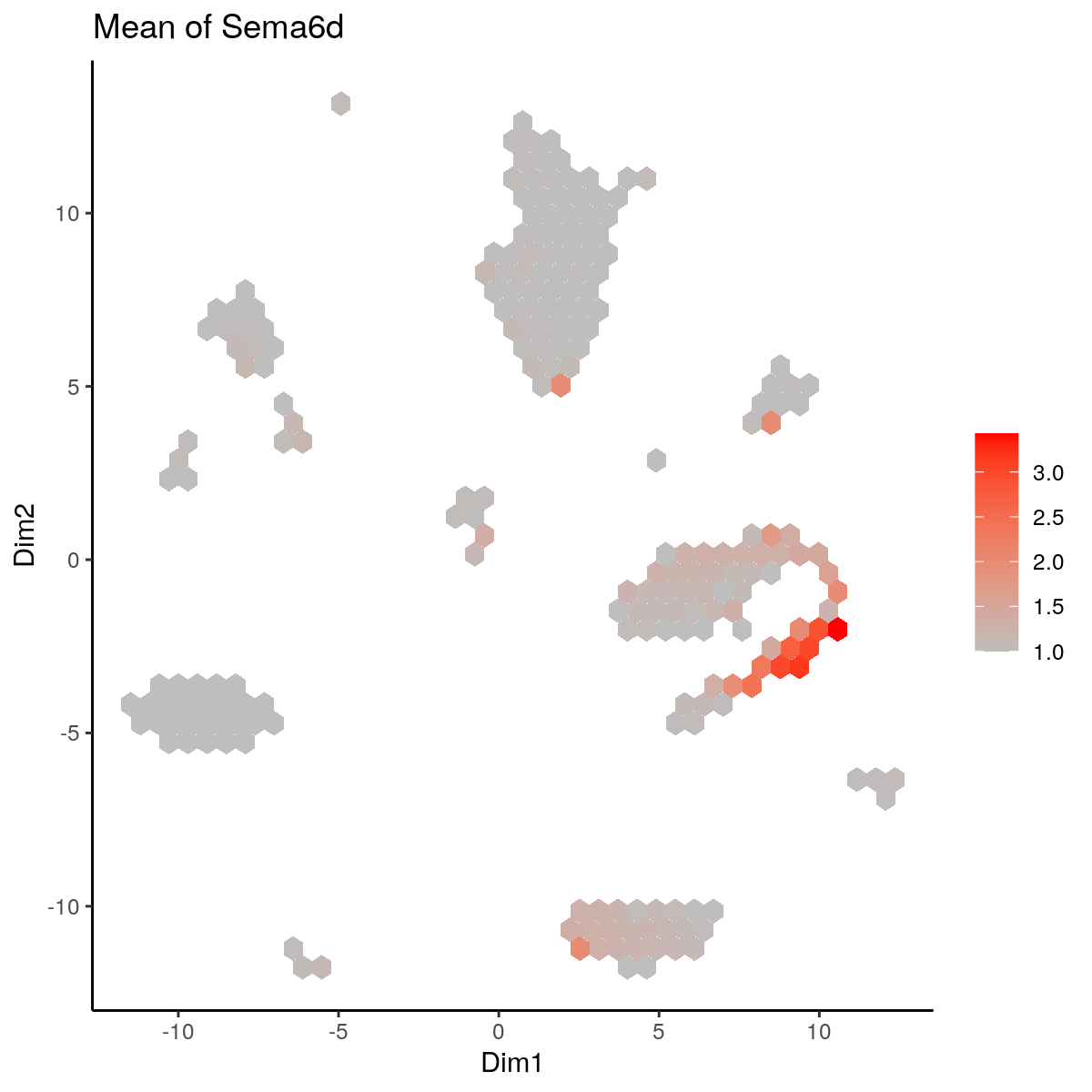

Supplement: Supplementary file 18 — Additional file 18. HTML report of VisualCortex. [file 12859_2023_5490_MOESM18_ESM.zip › output/report/Mouse_VisualCortex/figures/Ligand/214968.png]

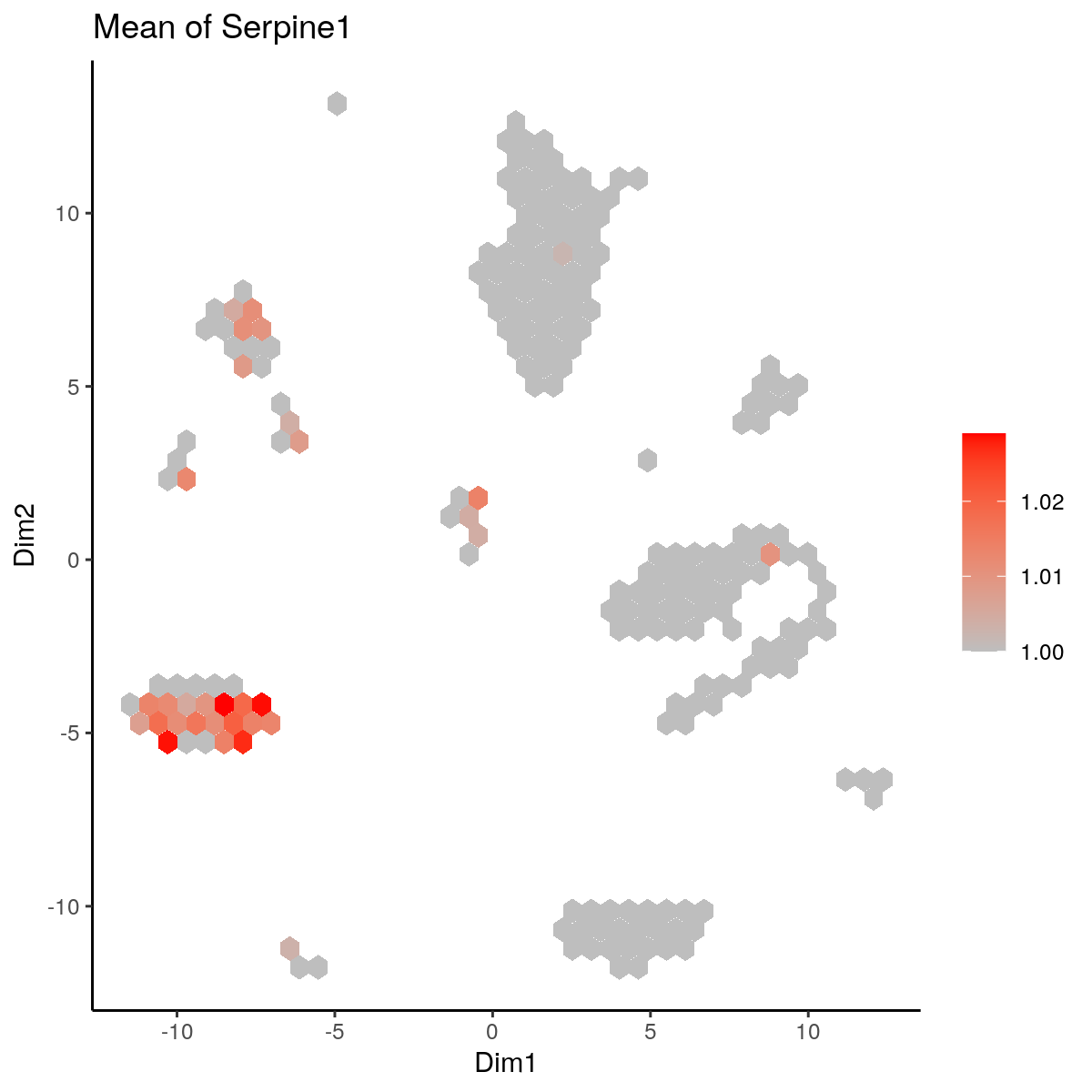

Supplement: Supplementary file 18 — Additional file 18. HTML report of VisualCortex. [file 12859_2023_5490_MOESM18_ESM.zip › output/report/Mouse_VisualCortex/figures/Ligand/18787.png]

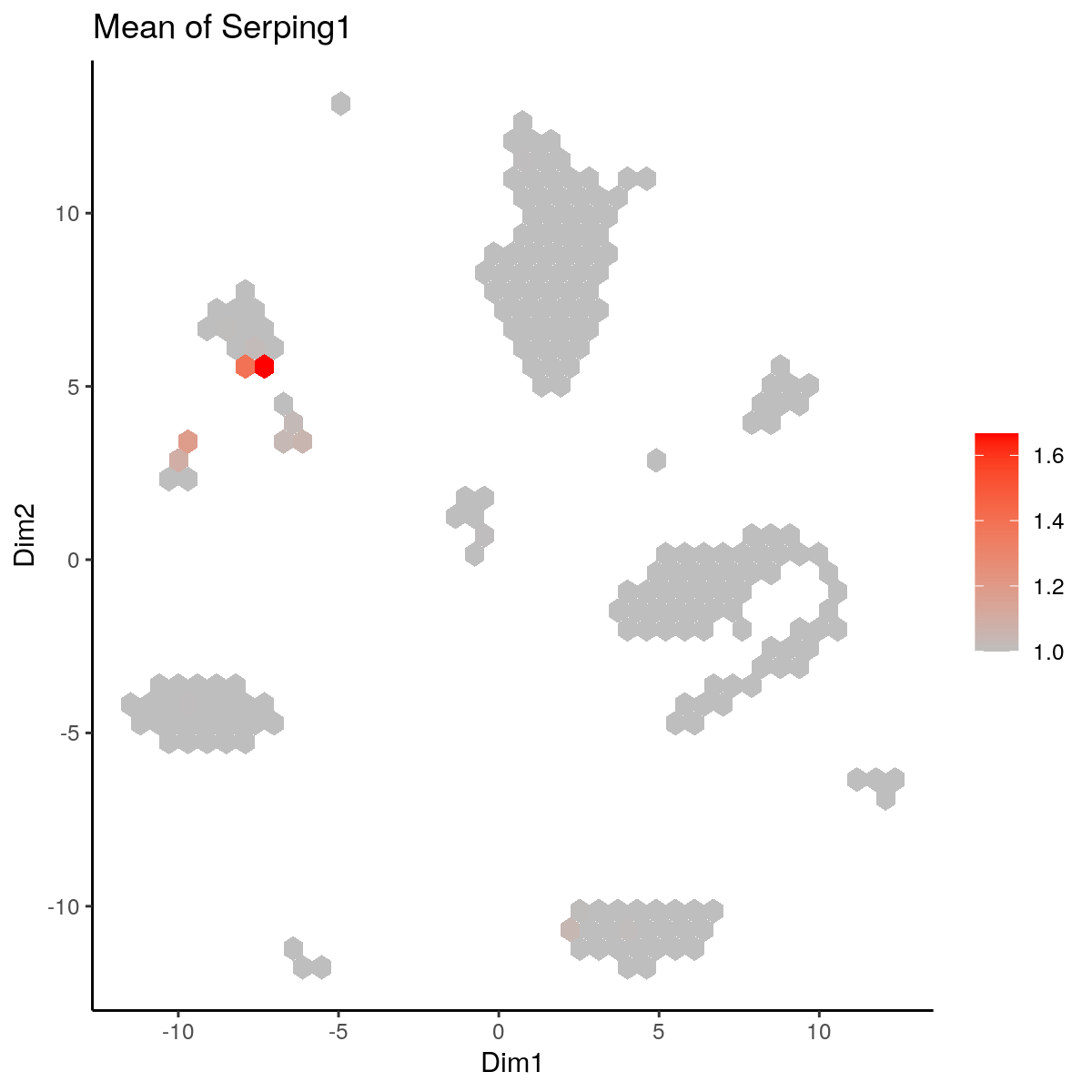

Supplement: Supplementary file 18 — Additional file 18. HTML report of VisualCortex. [file 12859_2023_5490_MOESM18_ESM.zip › output/report/Mouse_VisualCortex/figures/Ligand/12258.png]

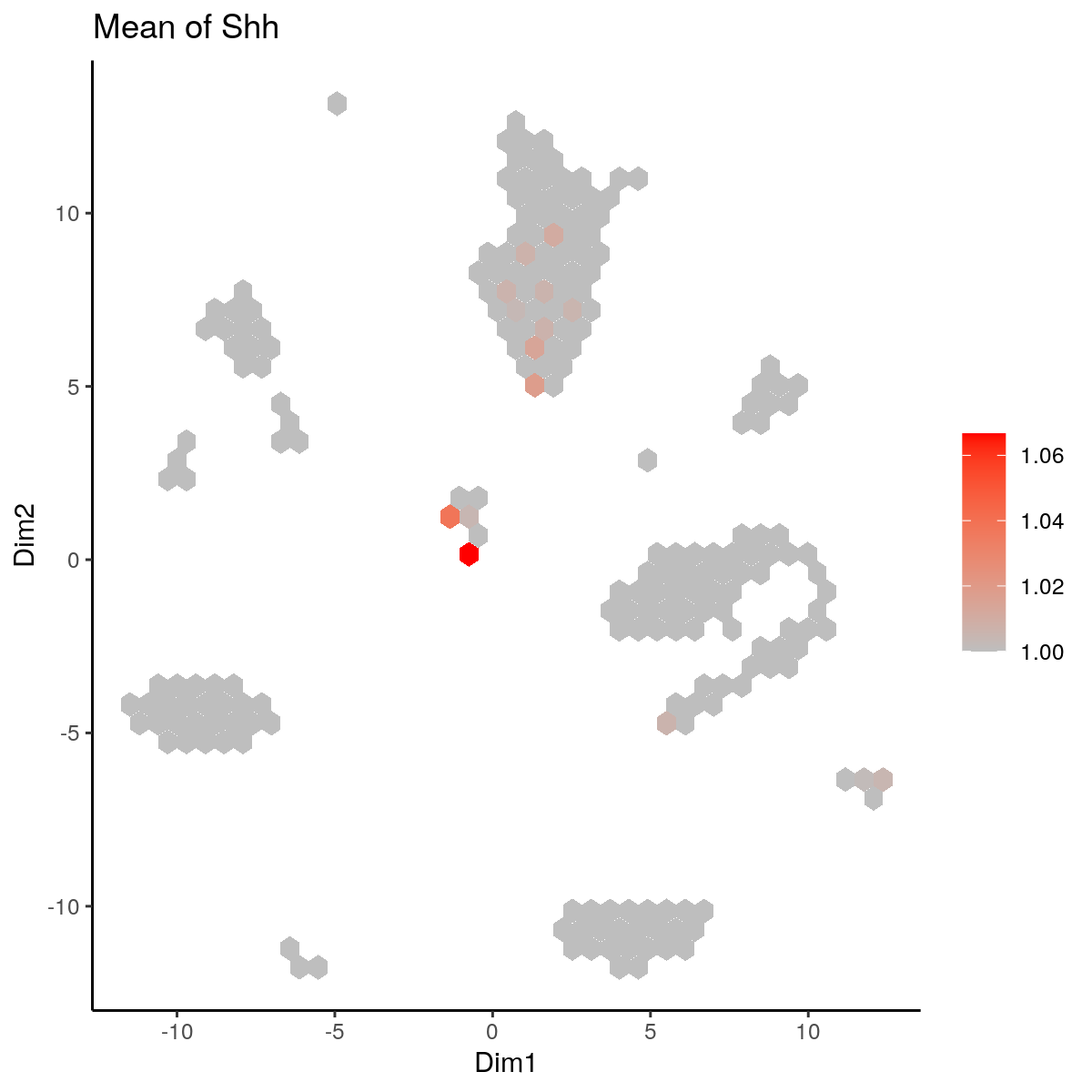

Supplement: Supplementary file 18 — Additional file 18. HTML report of VisualCortex. [file 12859_2023_5490_MOESM18_ESM.zip › output/report/Mouse_VisualCortex/figures/Ligand/20423.png]

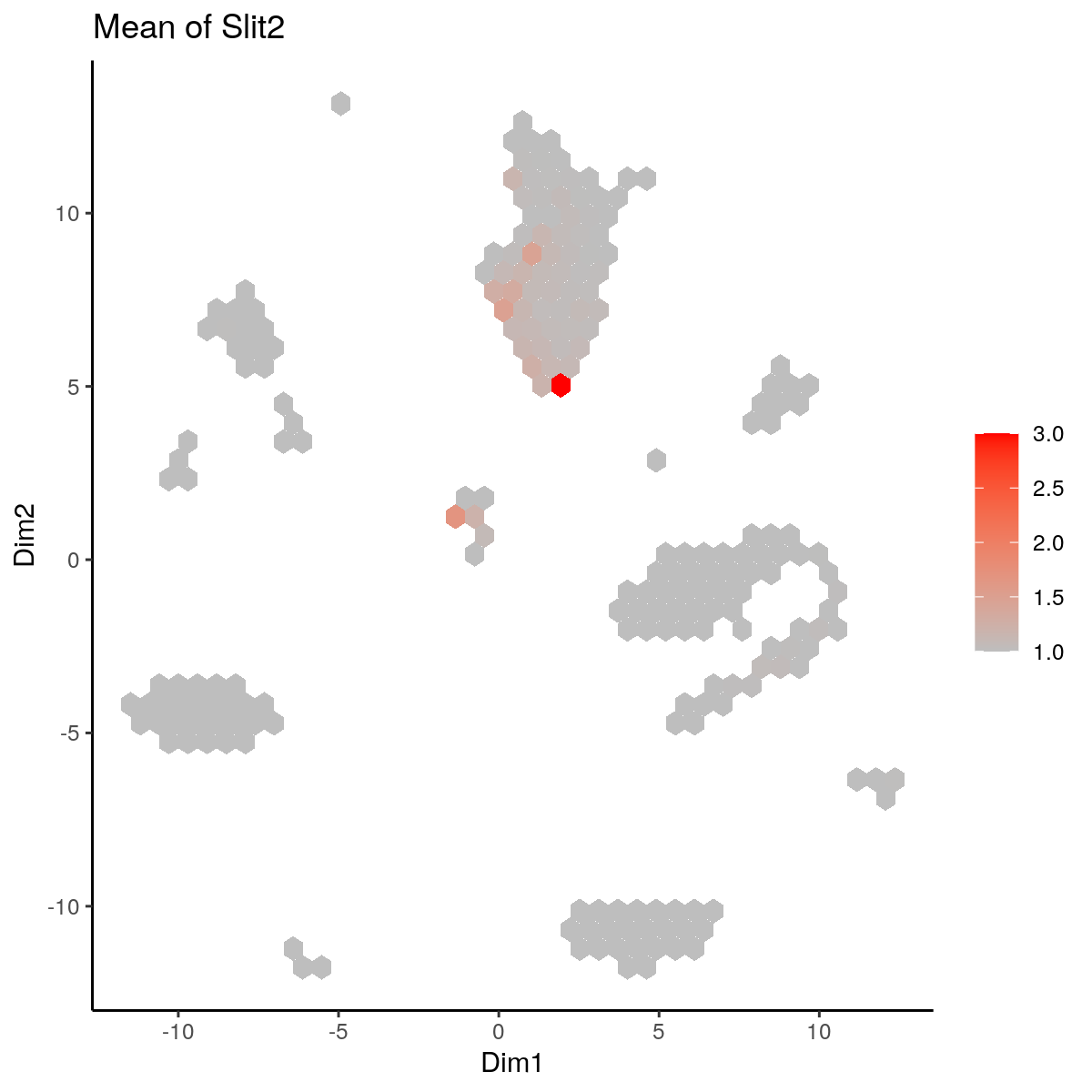

Supplement: Supplementary file 18 — Additional file 18. HTML report of VisualCortex. [file 12859_2023_5490_MOESM18_ESM.zip › output/report/Mouse_VisualCortex/figures/Ligand/20563.png]

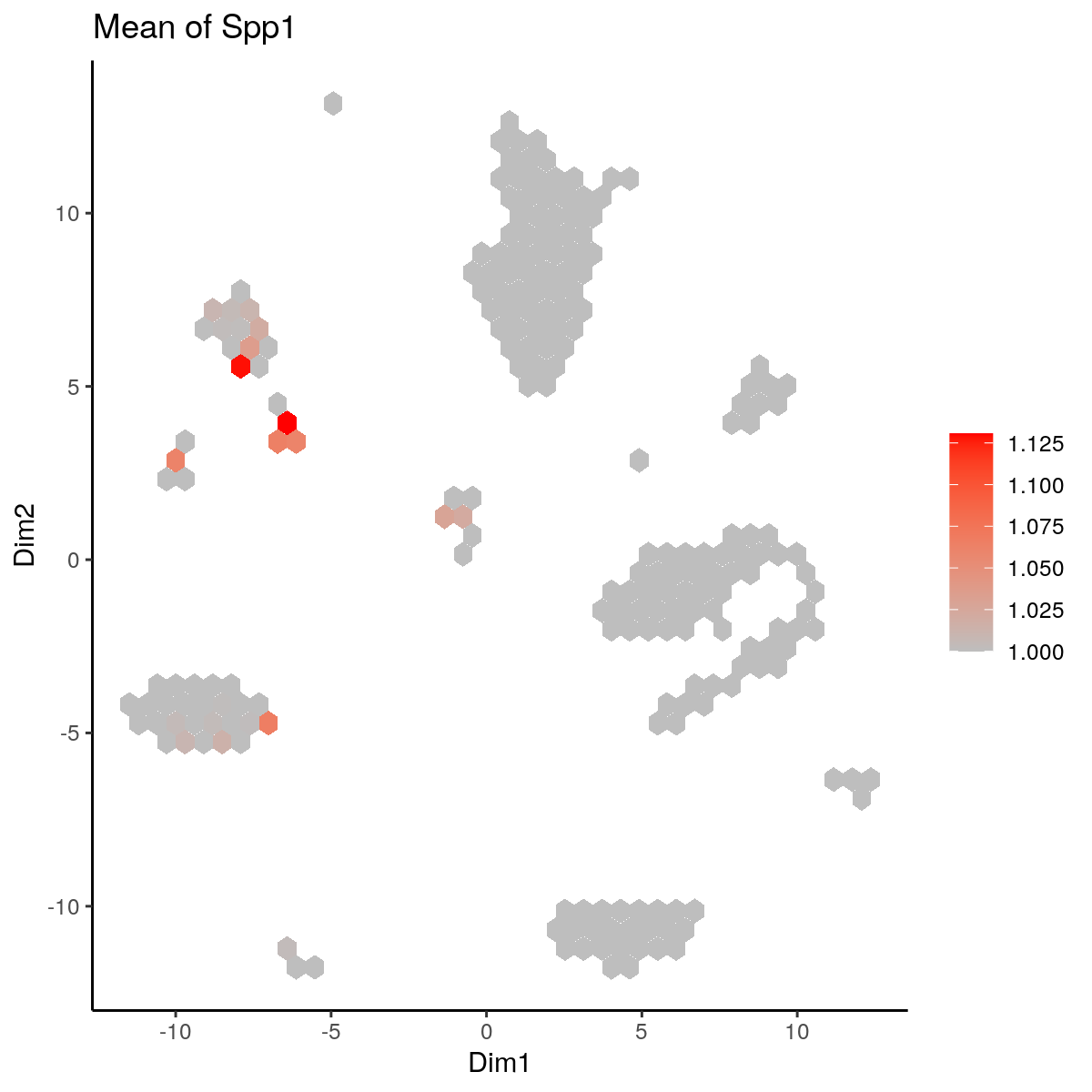

Supplement: Supplementary file 18 — Additional file 18. HTML report of VisualCortex. [file 12859_2023_5490_MOESM18_ESM.zip › output/report/Mouse_VisualCortex/figures/Ligand/20750.png]

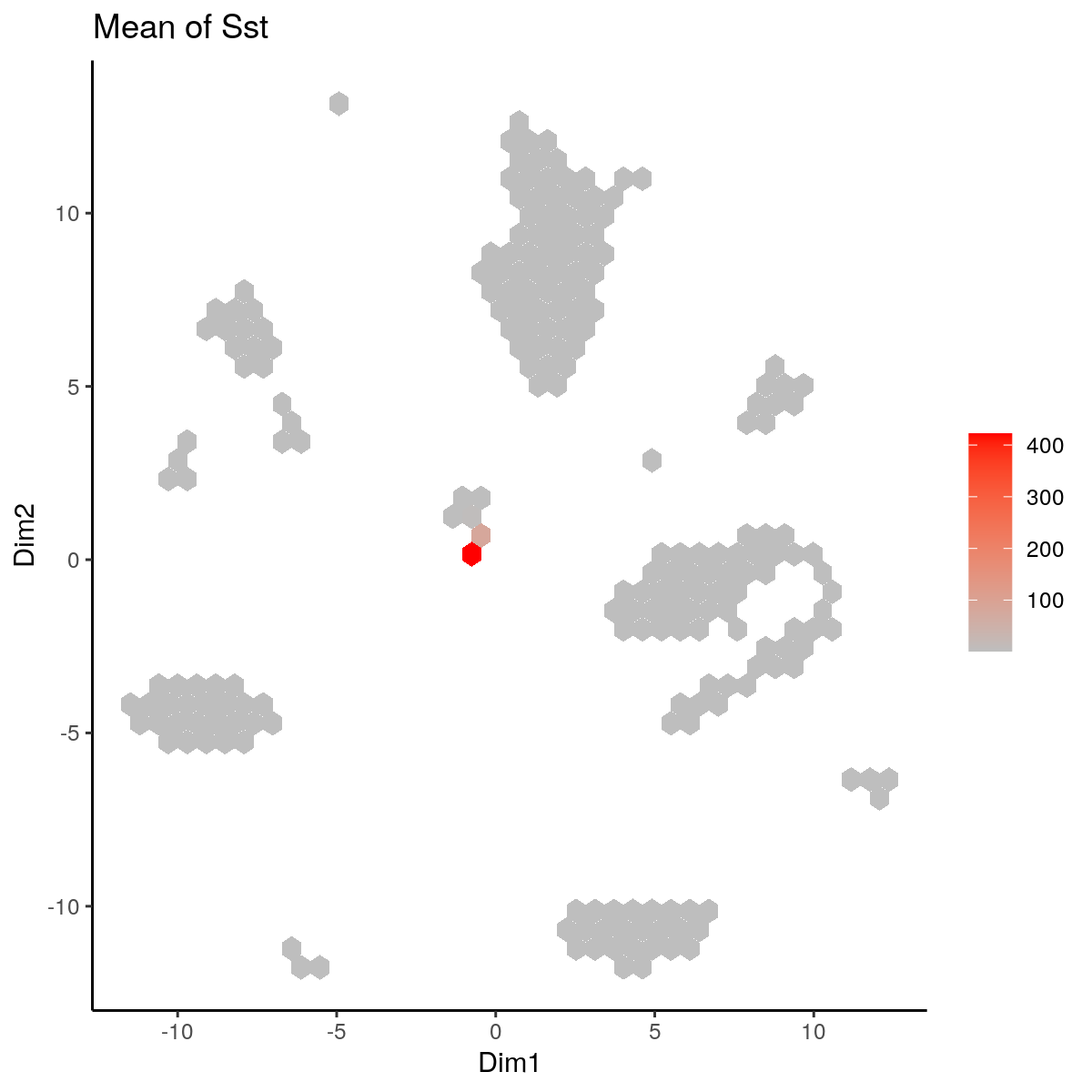

Supplement: Supplementary file 18 — Additional file 18. HTML report of VisualCortex. [file 12859_2023_5490_MOESM18_ESM.zip › output/report/Mouse_VisualCortex/figures/Ligand/20604.png]

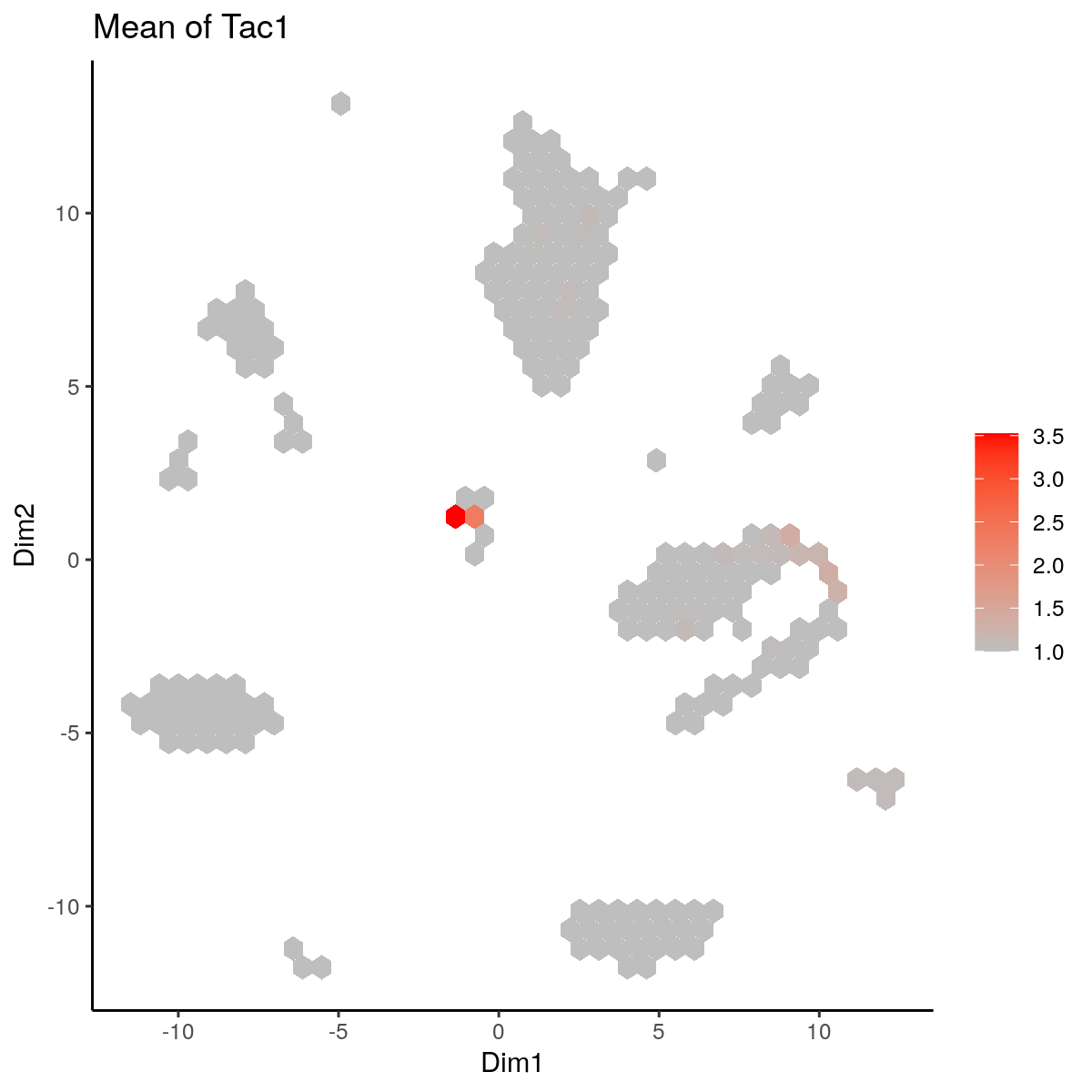

Supplement: Supplementary file 18 — Additional file 18. HTML report of VisualCortex. [file 12859_2023_5490_MOESM18_ESM.zip › output/report/Mouse_VisualCortex/figures/Ligand/21333.png]

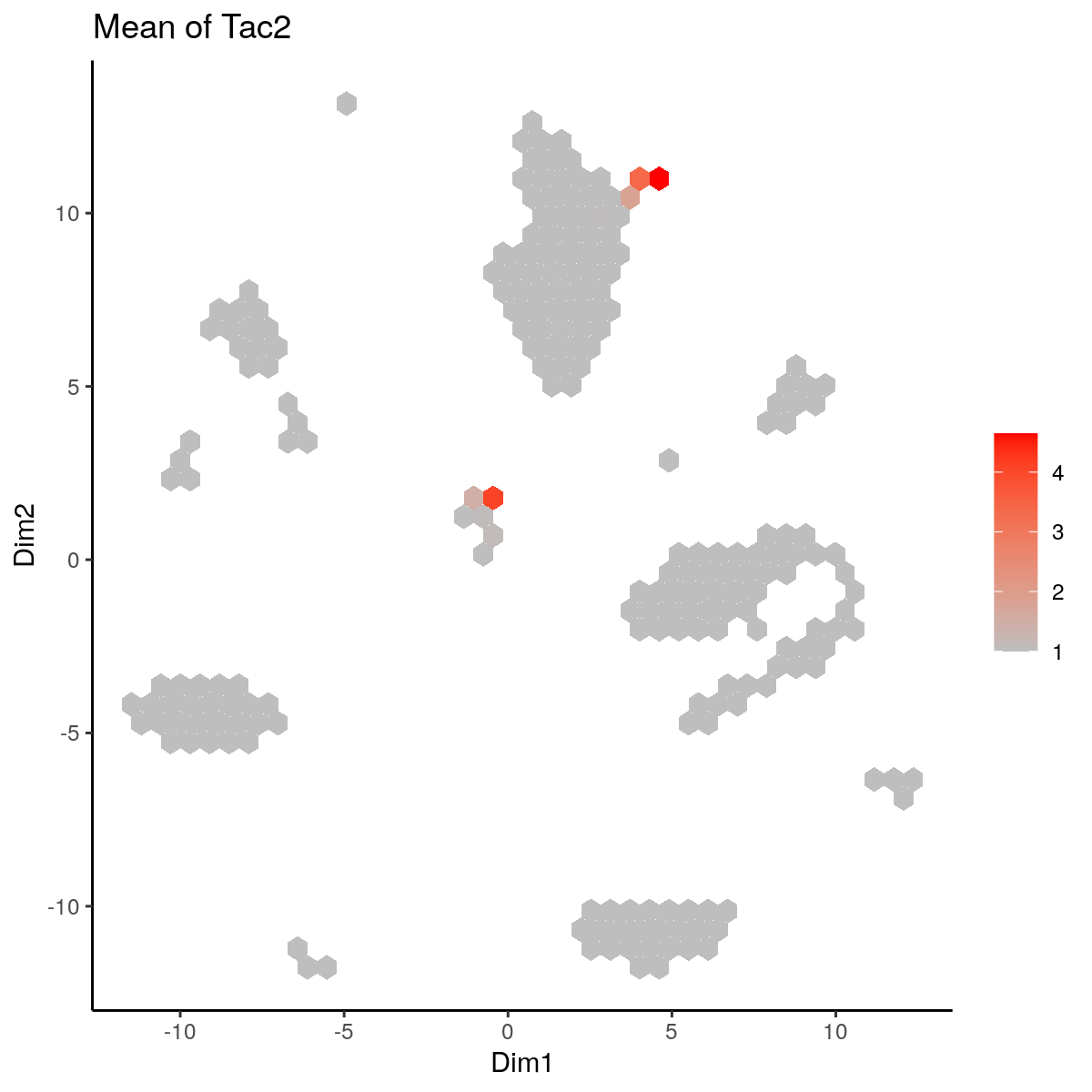

Supplement: Supplementary file 18 — Additional file 18. HTML report of VisualCortex. [file 12859_2023_5490_MOESM18_ESM.zip › output/report/Mouse_VisualCortex/figures/Ligand/21334.png]

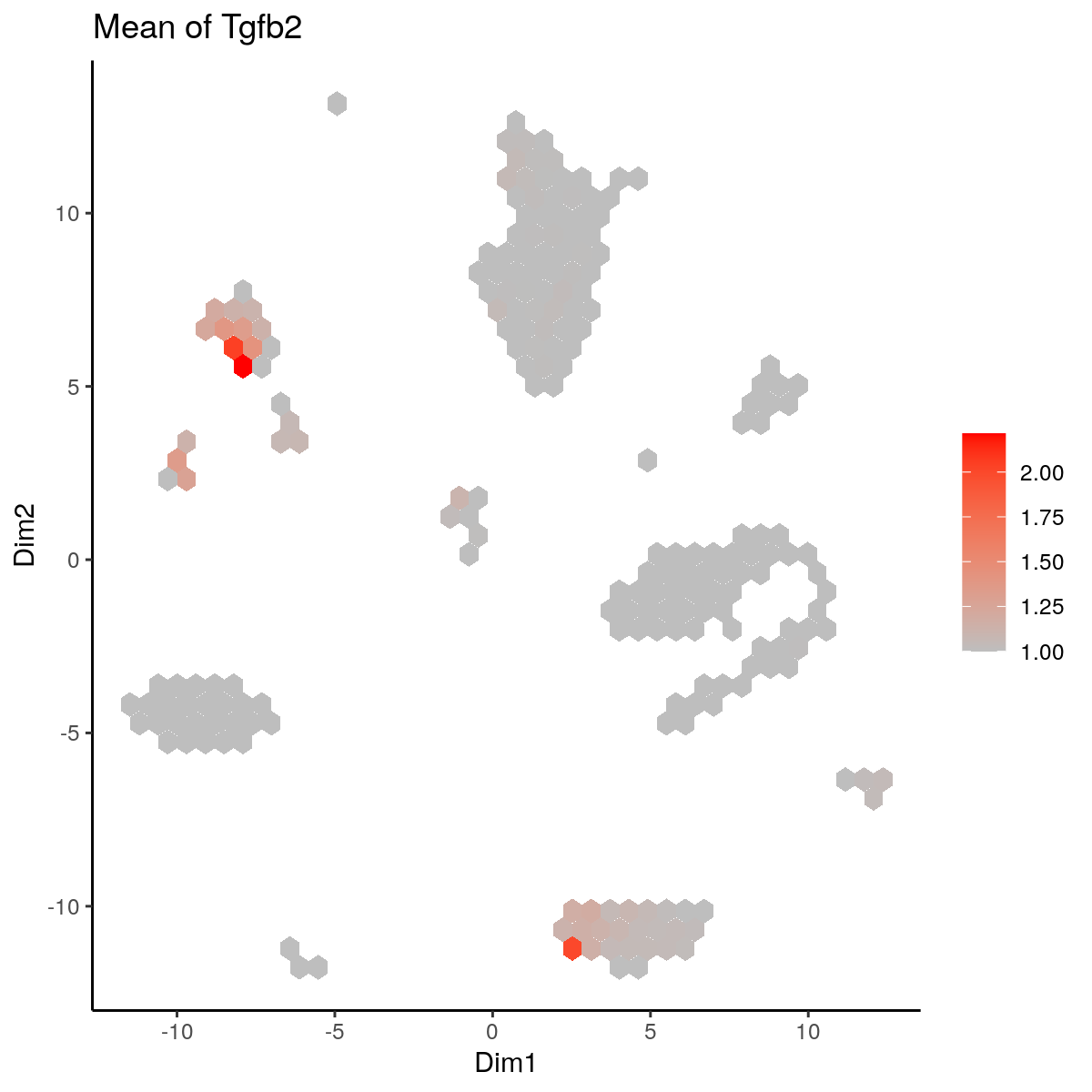

Supplement: Supplementary file 18 — Additional file 18. HTML report of VisualCortex. [file 12859_2023_5490_MOESM18_ESM.zip › output/report/Mouse_VisualCortex/figures/Ligand/21808.png]

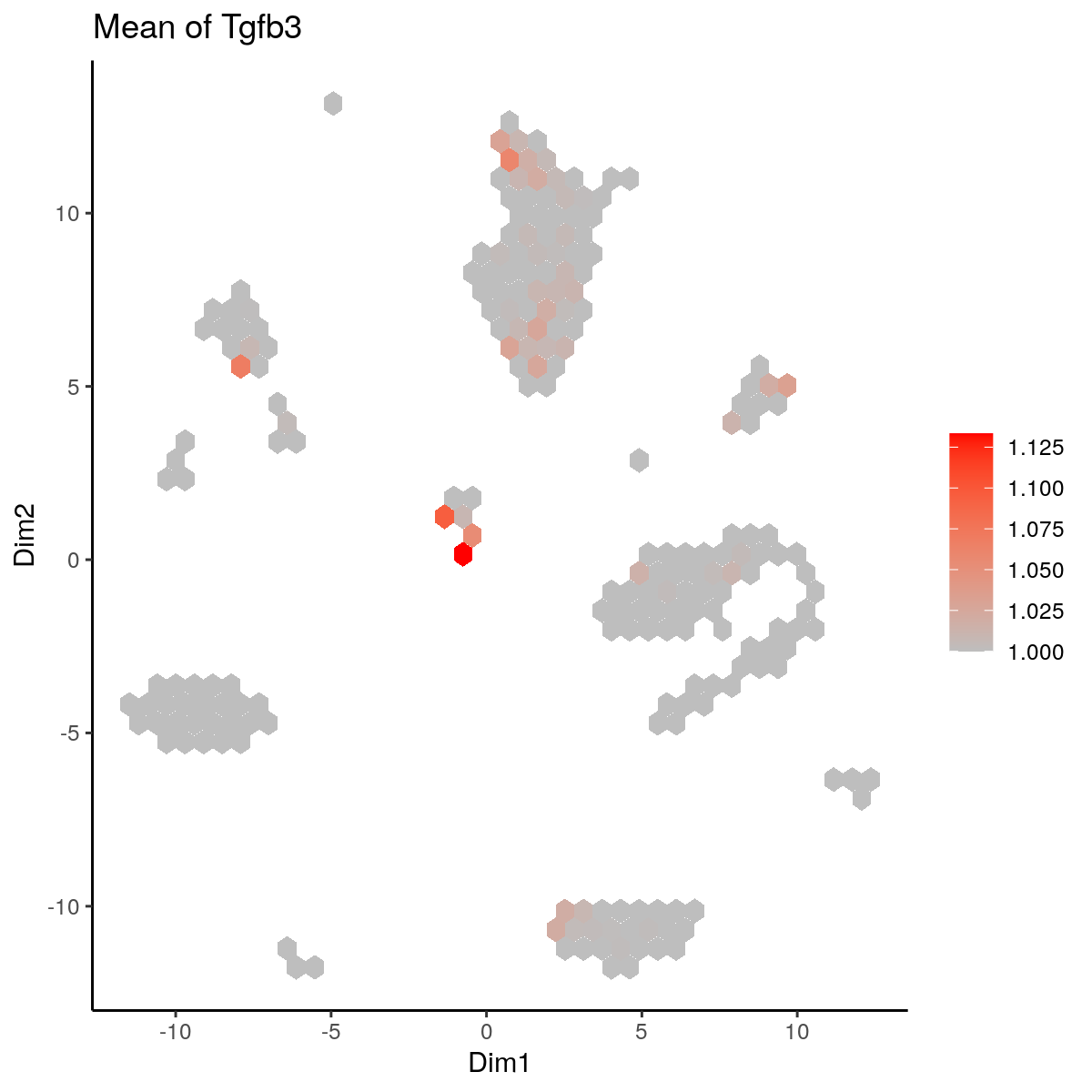

Supplement: Supplementary file 18 — Additional file 18. HTML report of VisualCortex. [file 12859_2023_5490_MOESM18_ESM.zip › output/report/Mouse_VisualCortex/figures/Ligand/21809.png]

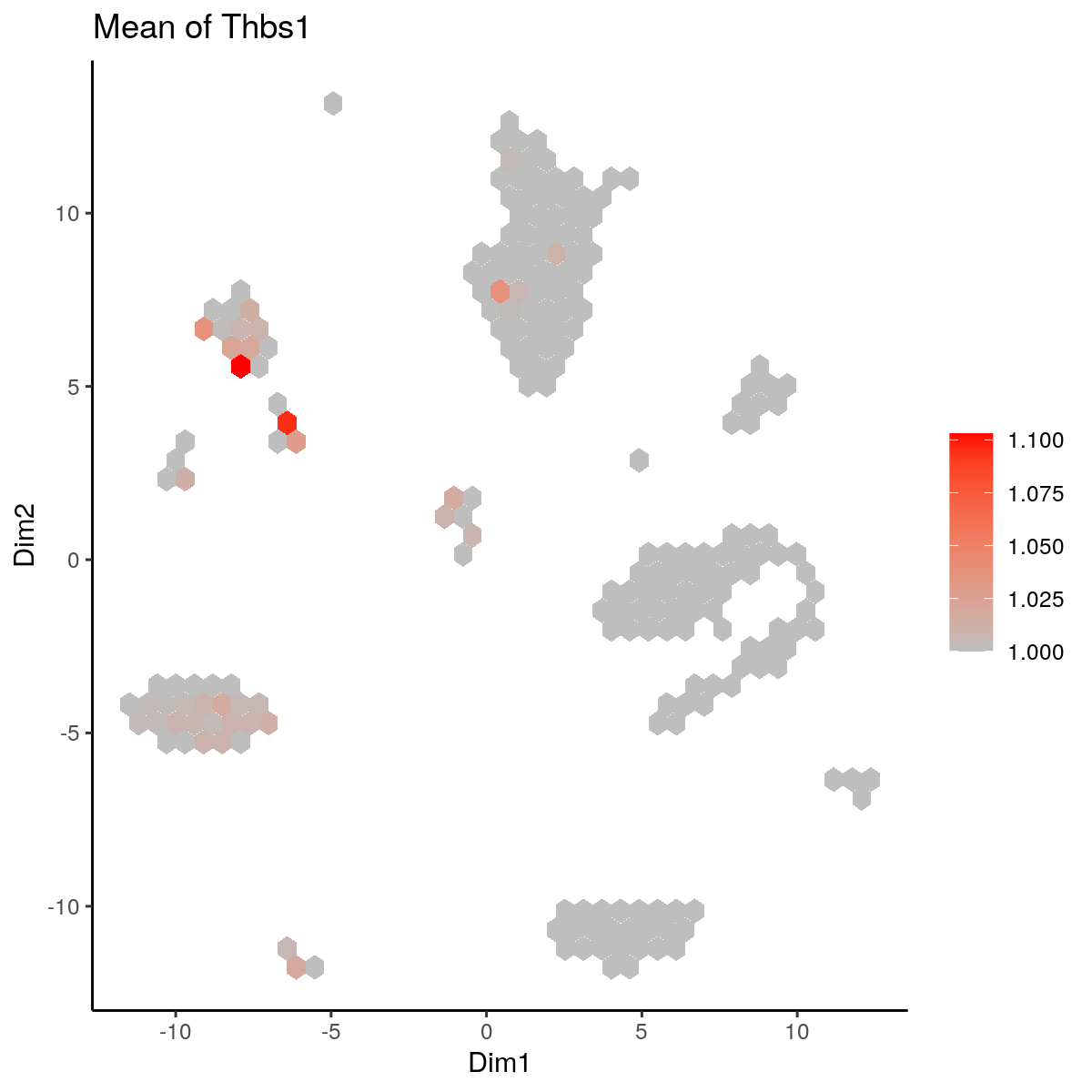

Supplement: Supplementary file 18 — Additional file 18. HTML report of VisualCortex. [file 12859_2023_5490_MOESM18_ESM.zip › output/report/Mouse_VisualCortex/figures/Ligand/21825.png]

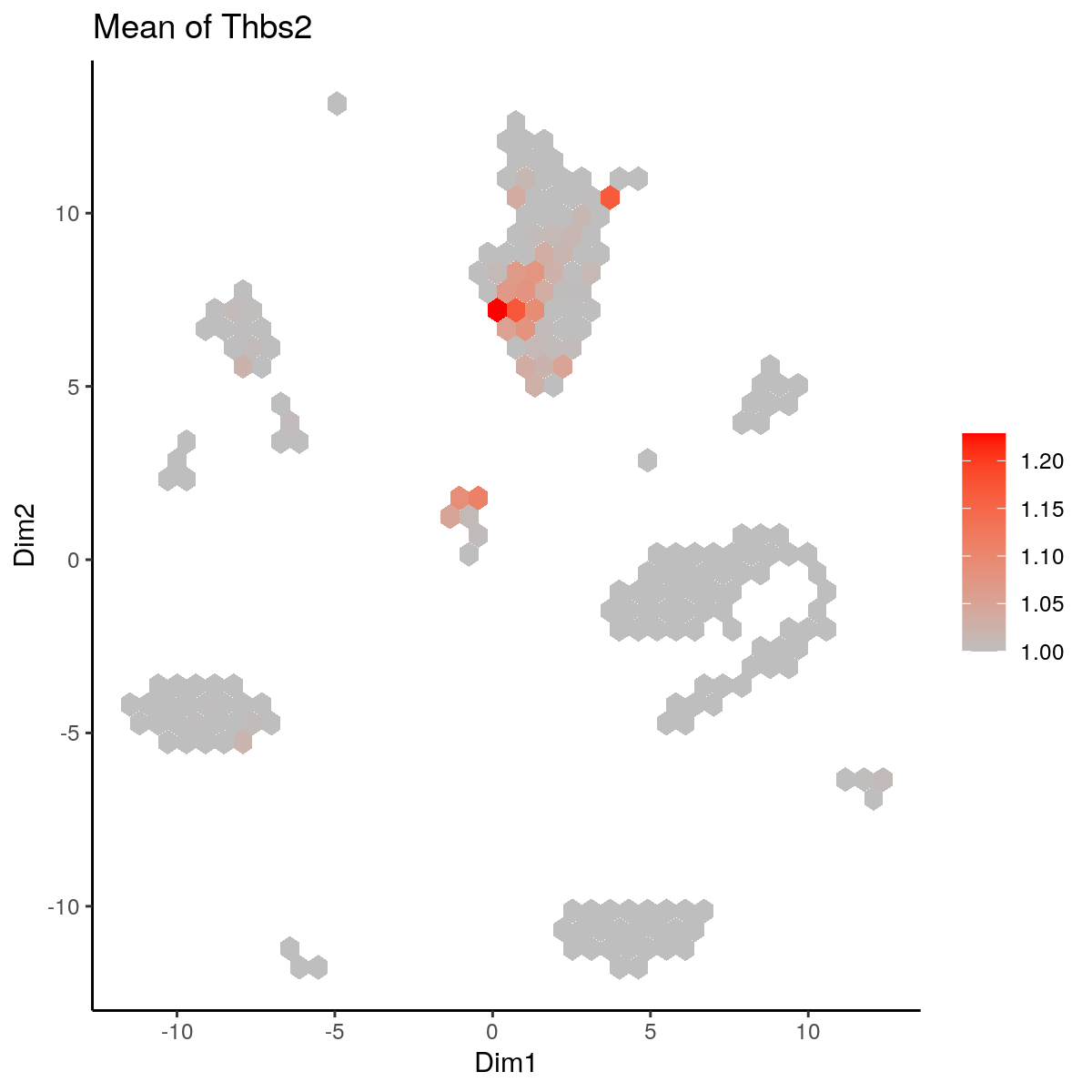

Supplement: Supplementary file 18 — Additional file 18. HTML report of VisualCortex. [file 12859_2023_5490_MOESM18_ESM.zip › output/report/Mouse_VisualCortex/figures/Ligand/21826.png]

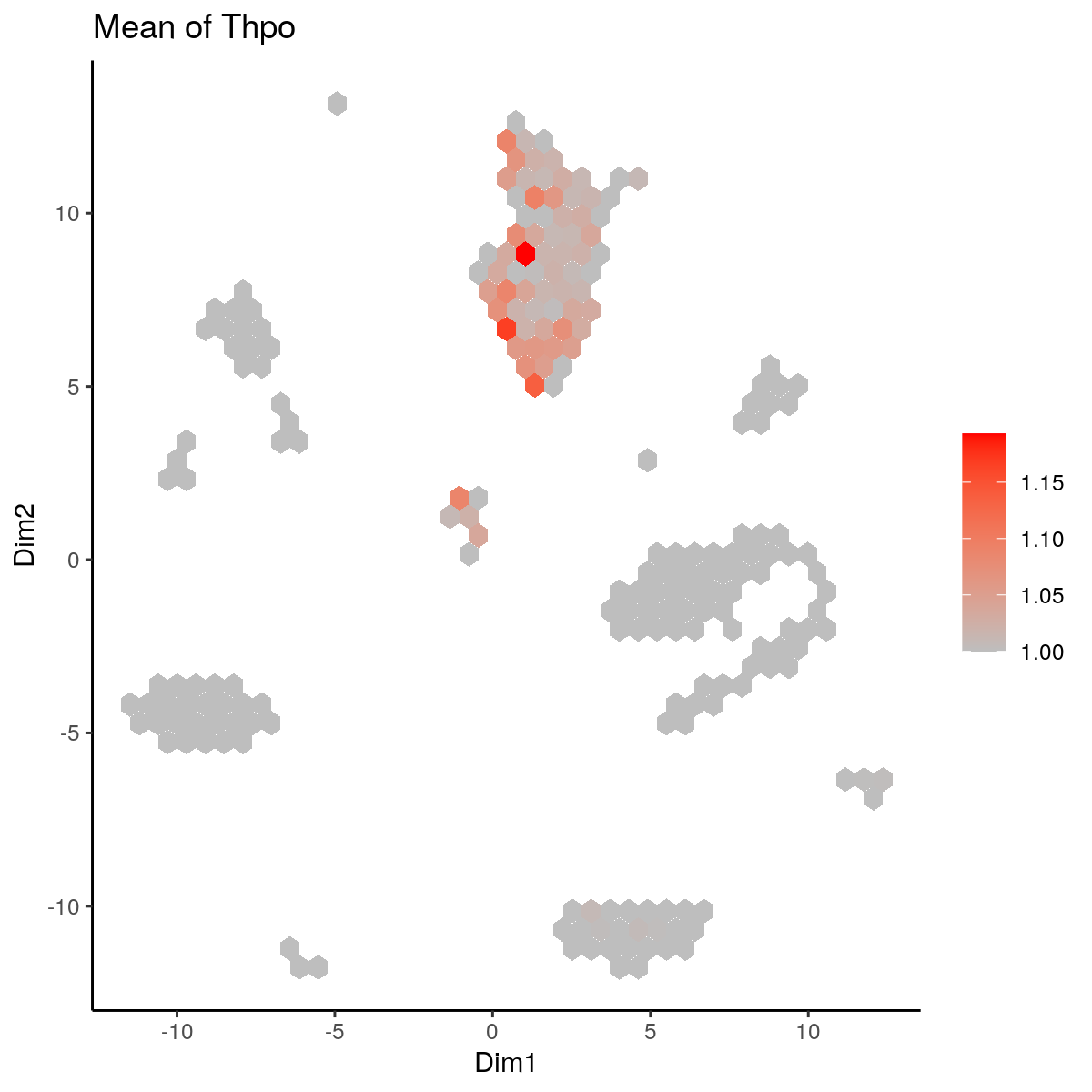

Supplement: Supplementary file 18 — Additional file 18. HTML report of VisualCortex. [file 12859_2023_5490_MOESM18_ESM.zip › output/report/Mouse_VisualCortex/figures/Ligand/21832.png]

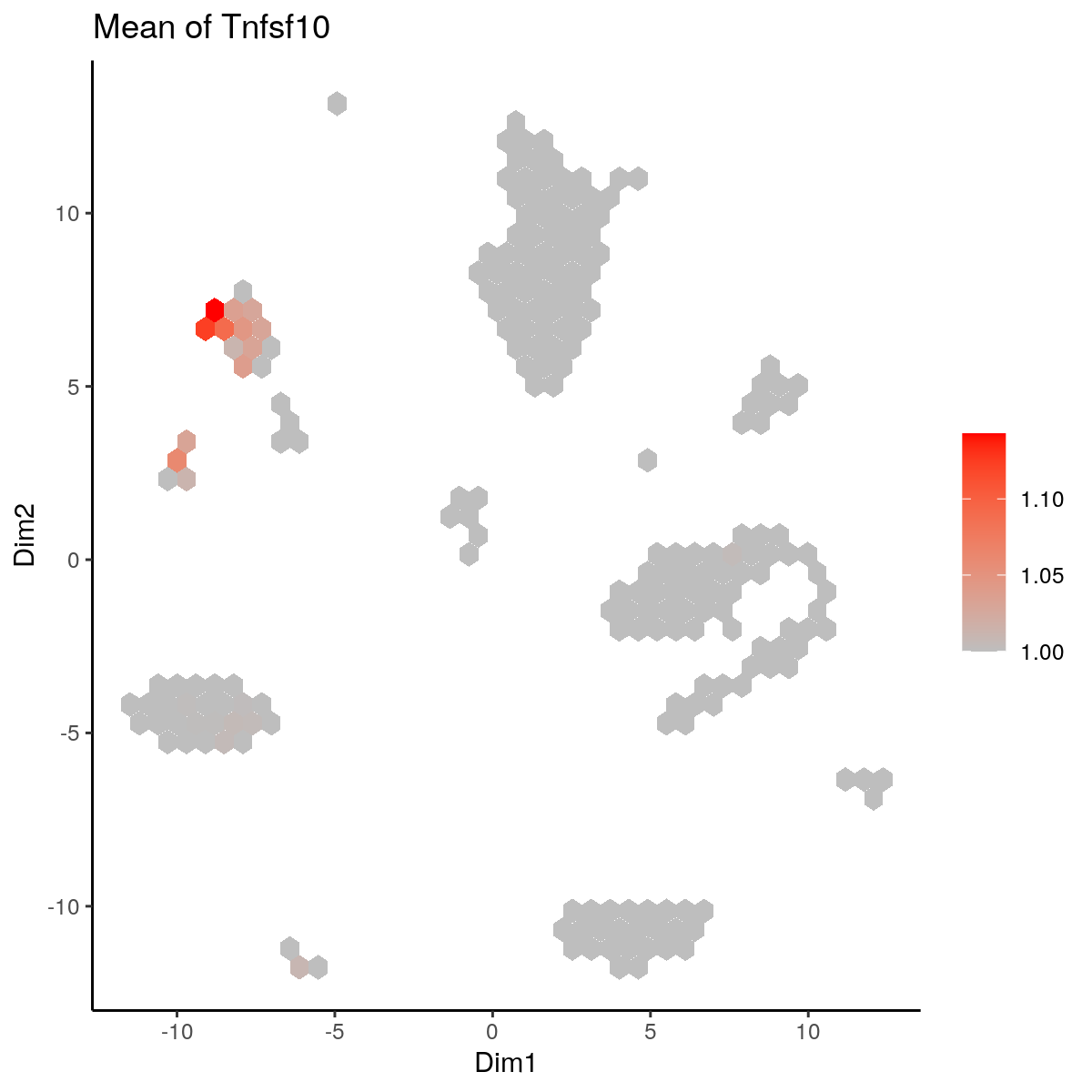

Supplement: Supplementary file 18 — Additional file 18. HTML report of VisualCortex. [file 12859_2023_5490_MOESM18_ESM.zip › output/report/Mouse_VisualCortex/figures/Ligand/22035.png]

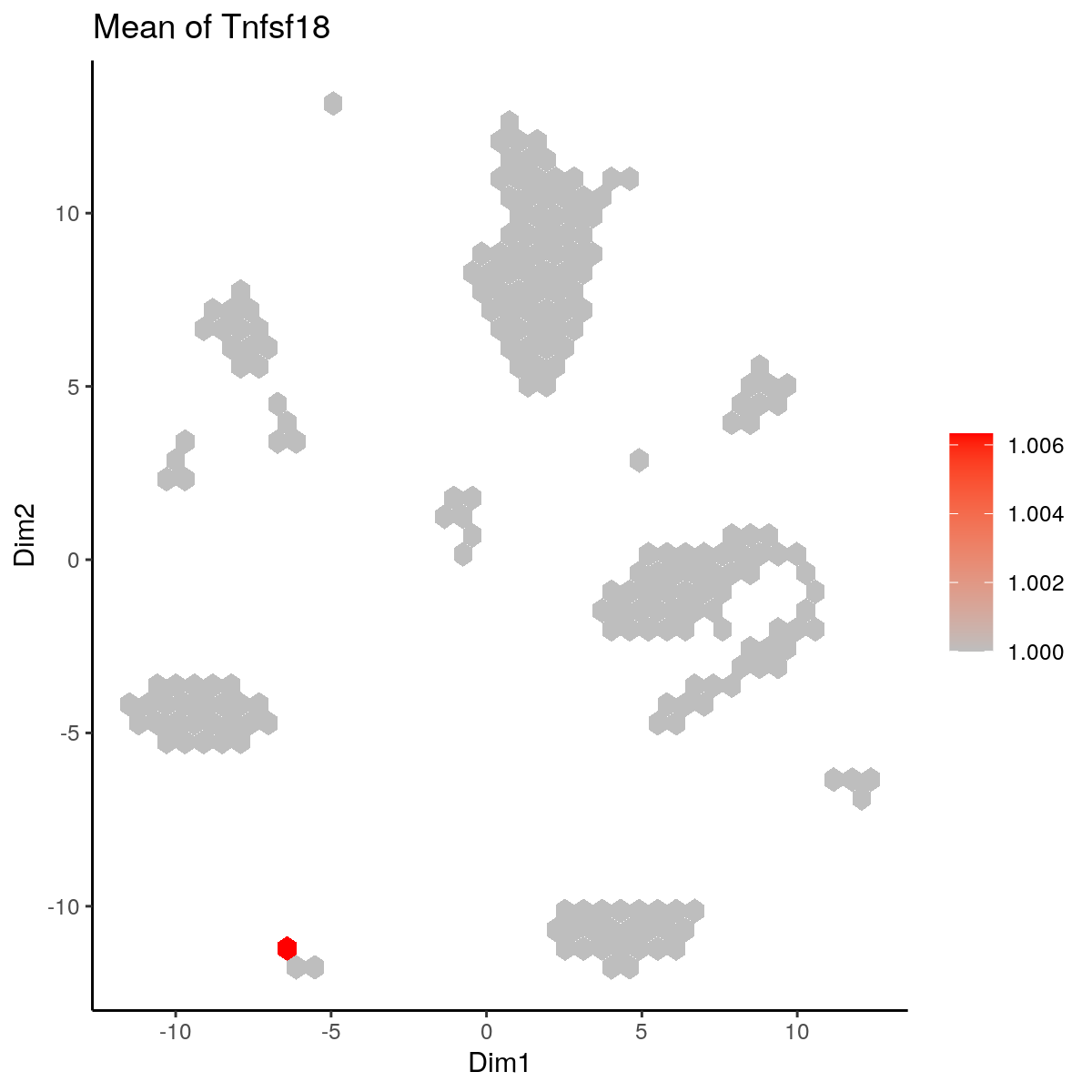

Supplement: Supplementary file 18 — Additional file 18. HTML report of VisualCortex. [file 12859_2023_5490_MOESM18_ESM.zip › output/report/Mouse_VisualCortex/figures/Ligand/240873.png]

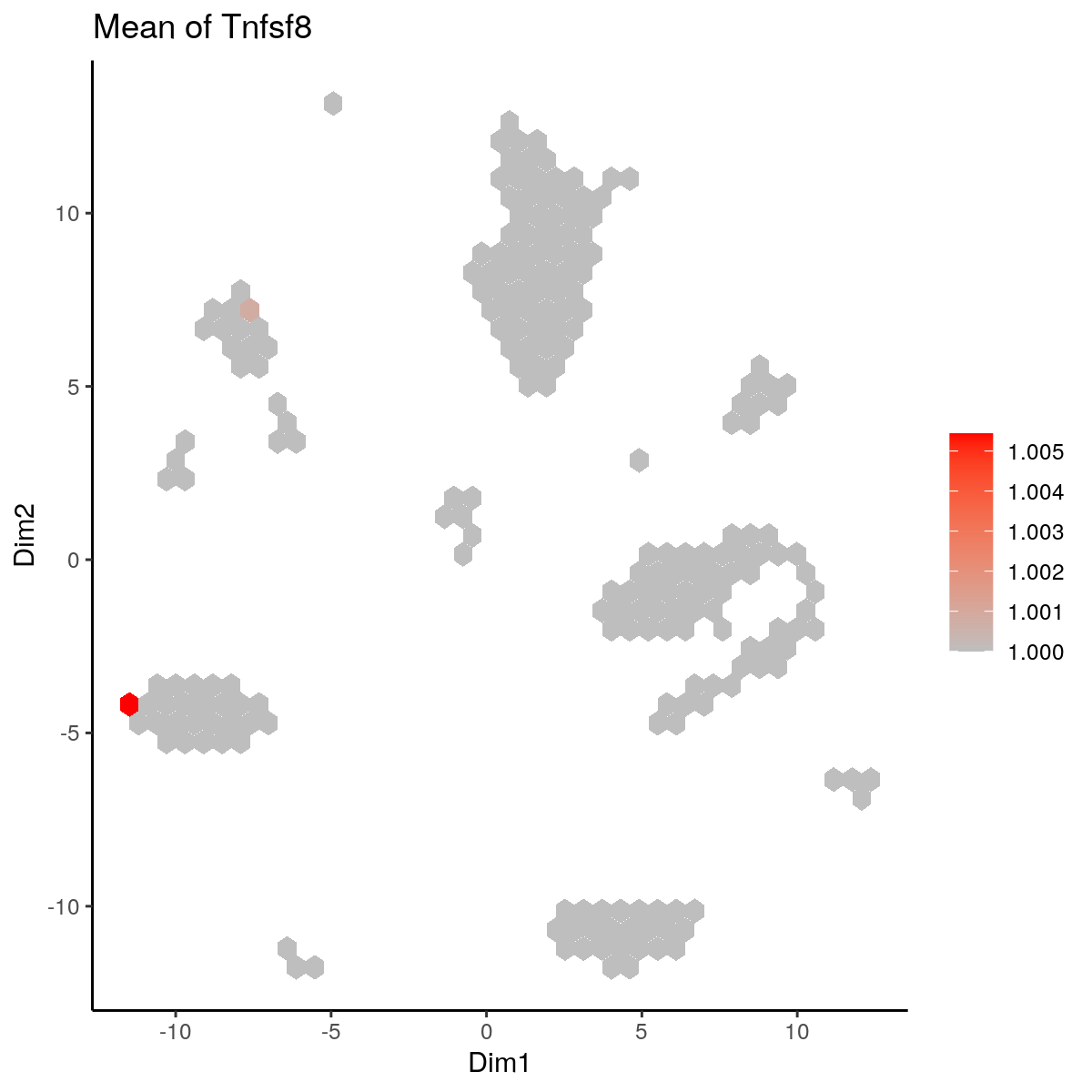

Supplement: Supplementary file 18 — Additional file 18. HTML report of VisualCortex. [file 12859_2023_5490_MOESM18_ESM.zip › output/report/Mouse_VisualCortex/figures/Ligand/21949.png]

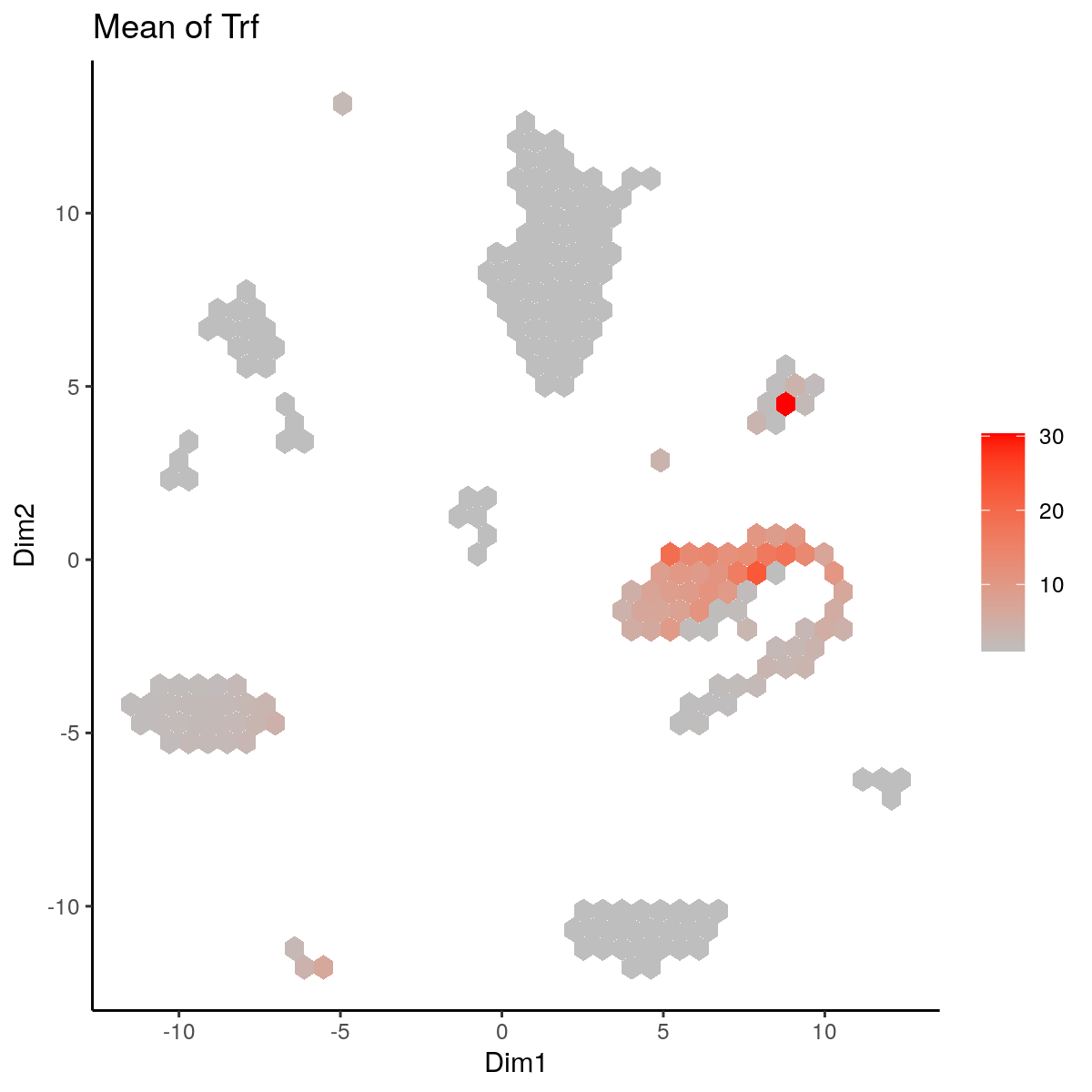

Supplement: Supplementary file 18 — Additional file 18. HTML report of VisualCortex. [file 12859_2023_5490_MOESM18_ESM.zip › output/report/Mouse_VisualCortex/figures/Ligand/22041.png]

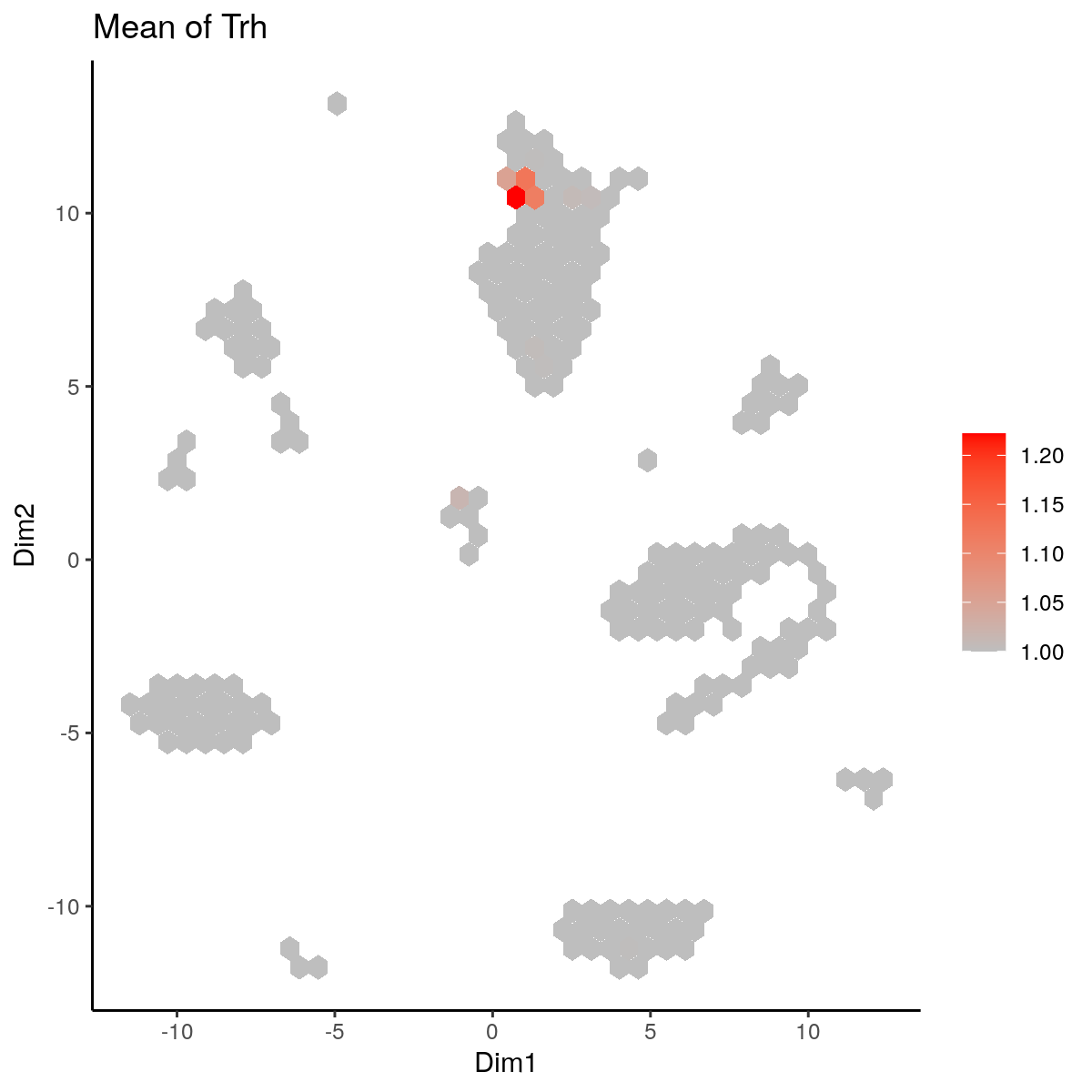

Supplement: Supplementary file 18 — Additional file 18. HTML report of VisualCortex. [file 12859_2023_5490_MOESM18_ESM.zip › output/report/Mouse_VisualCortex/figures/Ligand/22044.png]

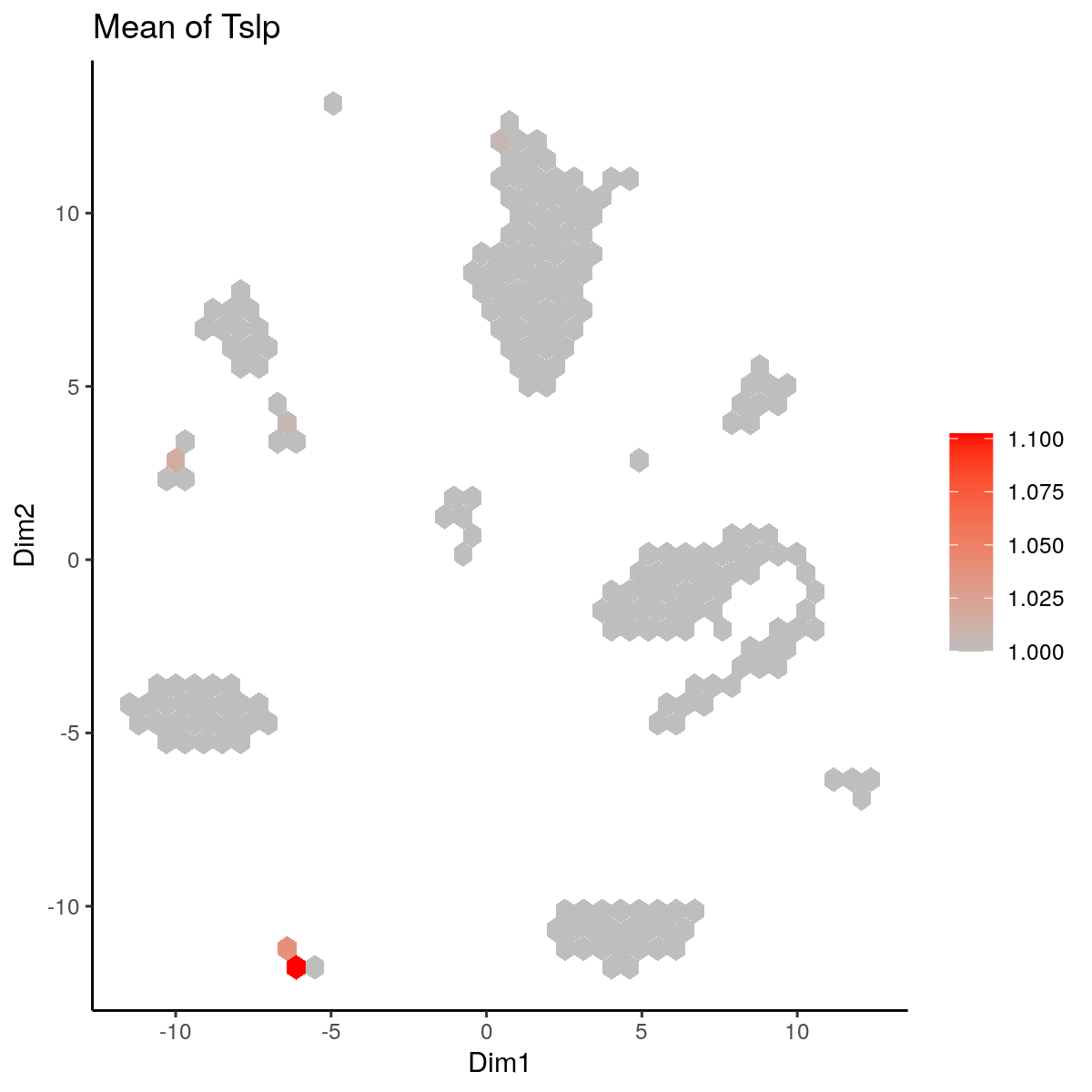

Supplement: Supplementary file 18 — Additional file 18. HTML report of VisualCortex. [file 12859_2023_5490_MOESM18_ESM.zip › output/report/Mouse_VisualCortex/figures/Ligand/53603.png]

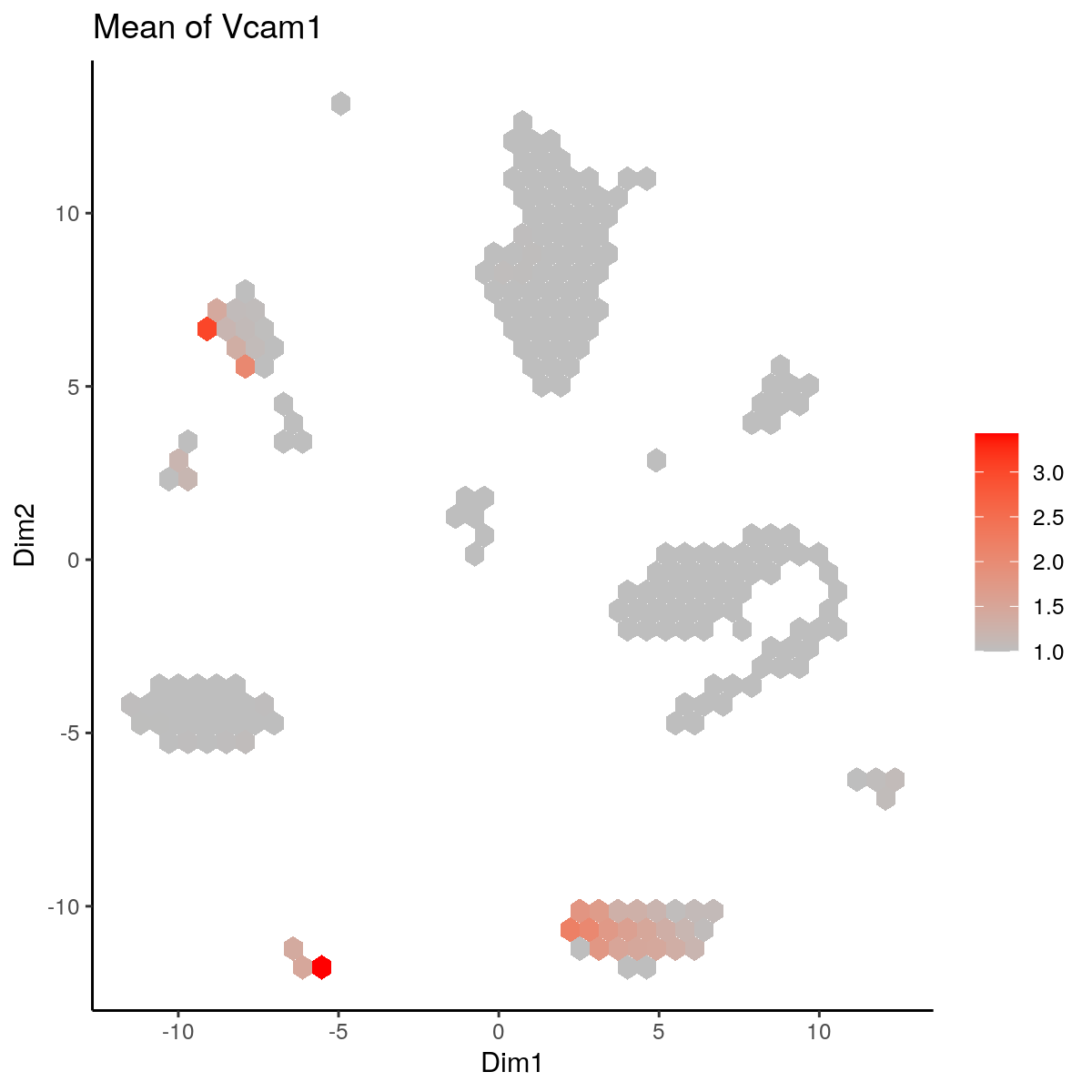

Supplement: Supplementary file 18 — Additional file 18. HTML report of VisualCortex. [file 12859_2023_5490_MOESM18_ESM.zip › output/report/Mouse_VisualCortex/figures/Ligand/22329.png]

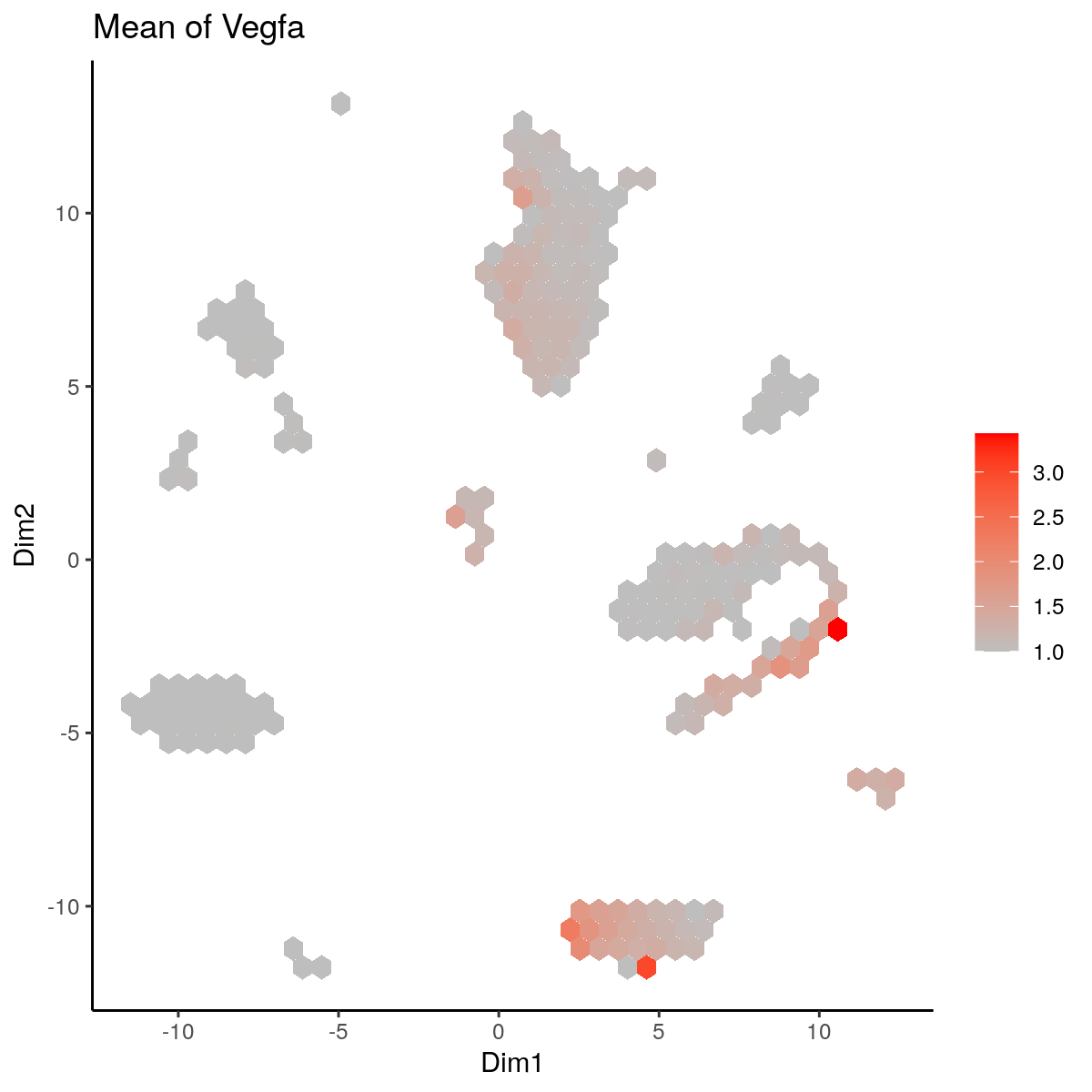

Supplement: Supplementary file 18 — Additional file 18. HTML report of VisualCortex. [file 12859_2023_5490_MOESM18_ESM.zip › output/report/Mouse_VisualCortex/figures/Ligand/22339.png]

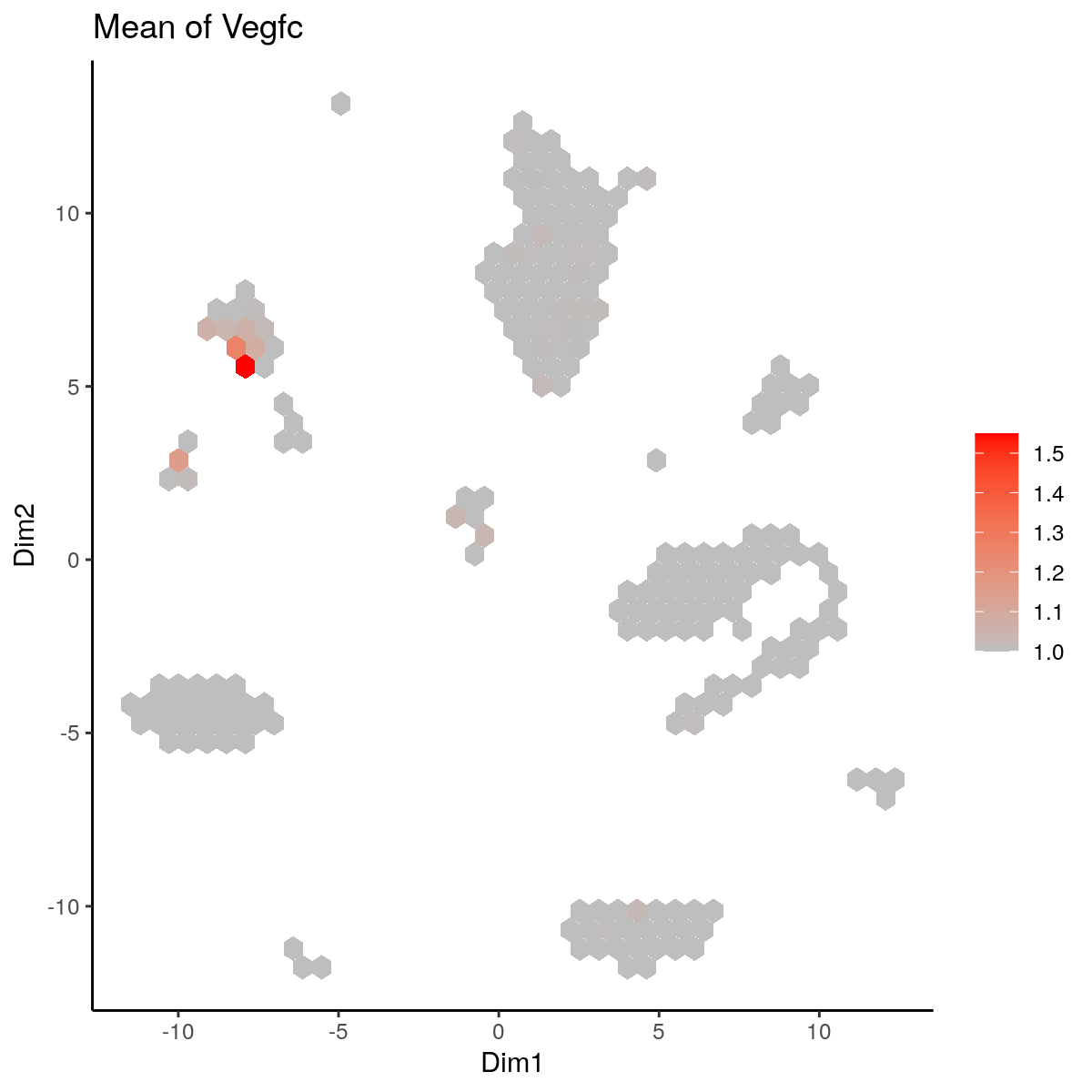

Supplement: Supplementary file 18 — Additional file 18. HTML report of VisualCortex. [file 12859_2023_5490_MOESM18_ESM.zip › output/report/Mouse_VisualCortex/figures/Ligand/22341.png]

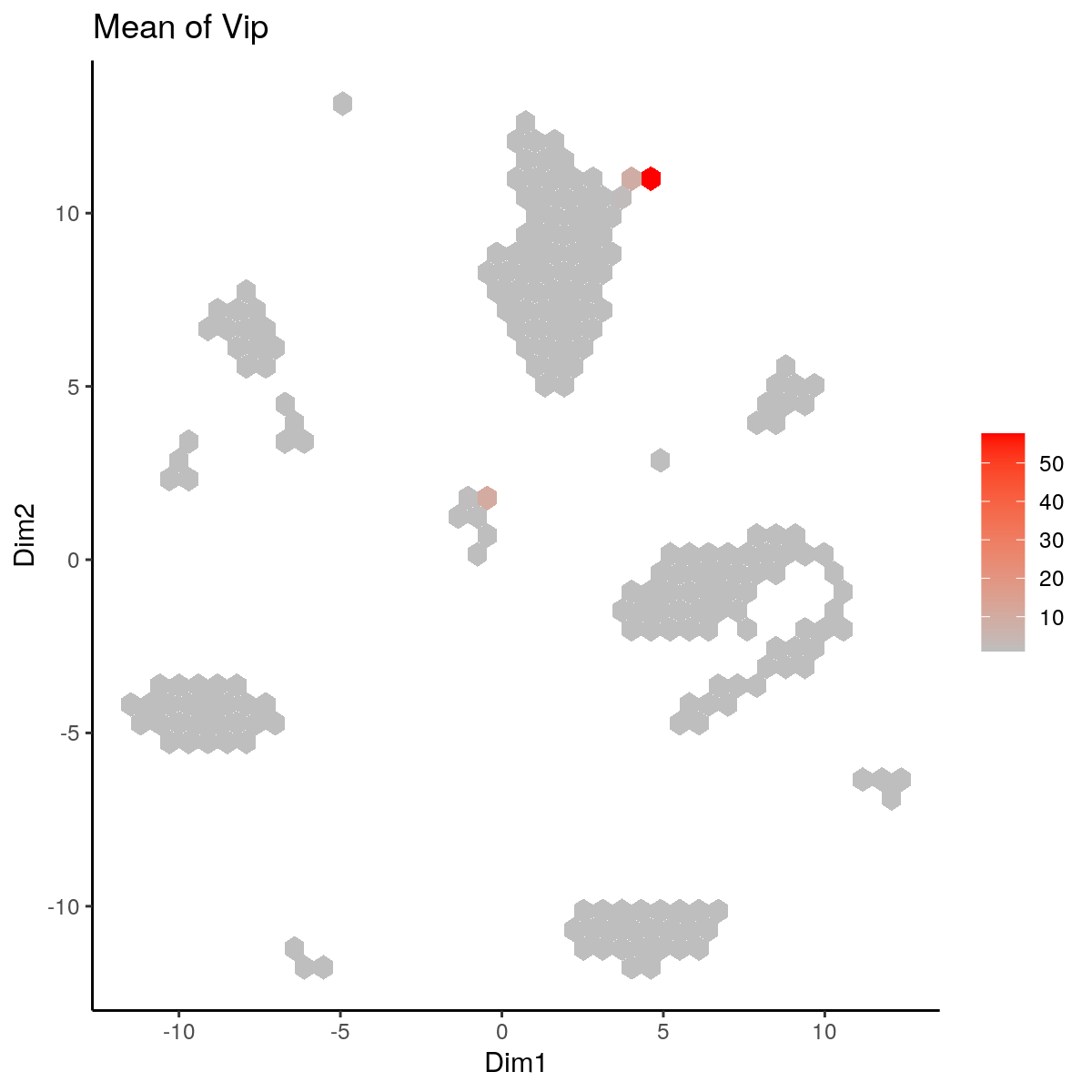

Supplement: Supplementary file 18 — Additional file 18. HTML report of VisualCortex. [file 12859_2023_5490_MOESM18_ESM.zip › output/report/Mouse_VisualCortex/figures/Ligand/22353.png]

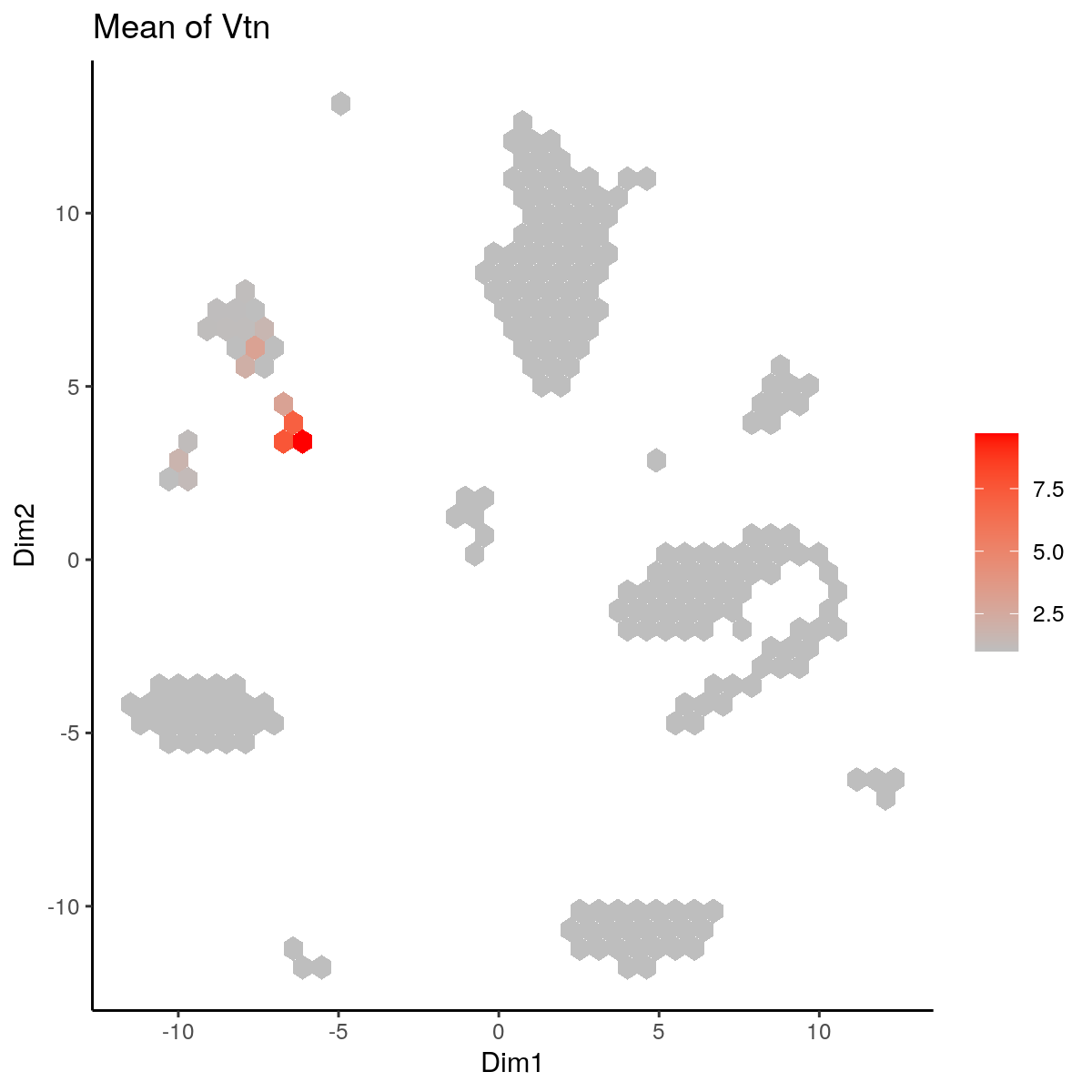

Supplement: Supplementary file 18 — Additional file 18. HTML report of VisualCortex. [file 12859_2023_5490_MOESM18_ESM.zip › output/report/Mouse_VisualCortex/figures/Ligand/22370.png]

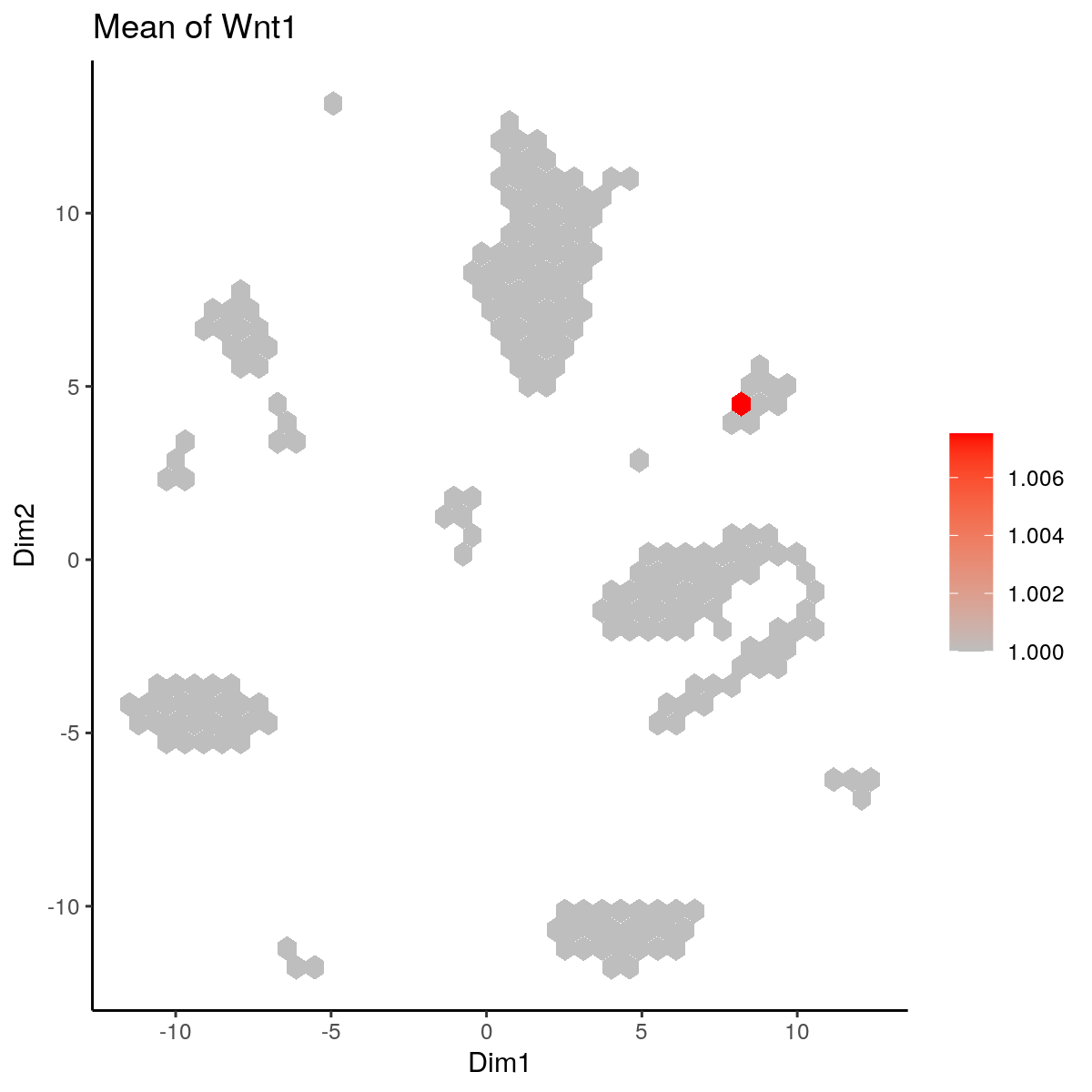

Supplement: Supplementary file 18 — Additional file 18. HTML report of VisualCortex. [file 12859_2023_5490_MOESM18_ESM.zip › output/report/Mouse_VisualCortex/figures/Ligand/22408.png]

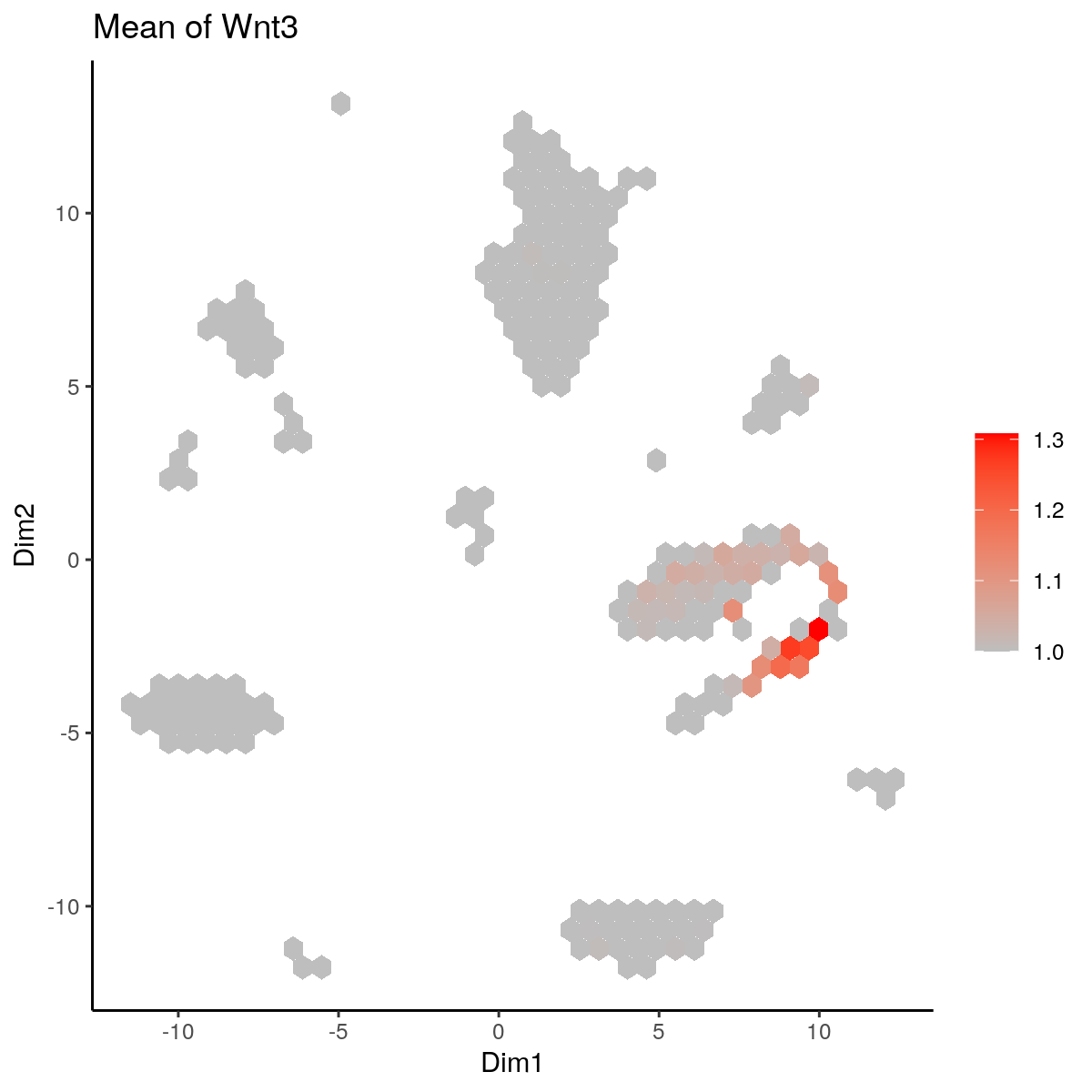

Supplement: Supplementary file 18 — Additional file 18. HTML report of VisualCortex. [file 12859_2023_5490_MOESM18_ESM.zip › output/report/Mouse_VisualCortex/figures/Ligand/22415.png]

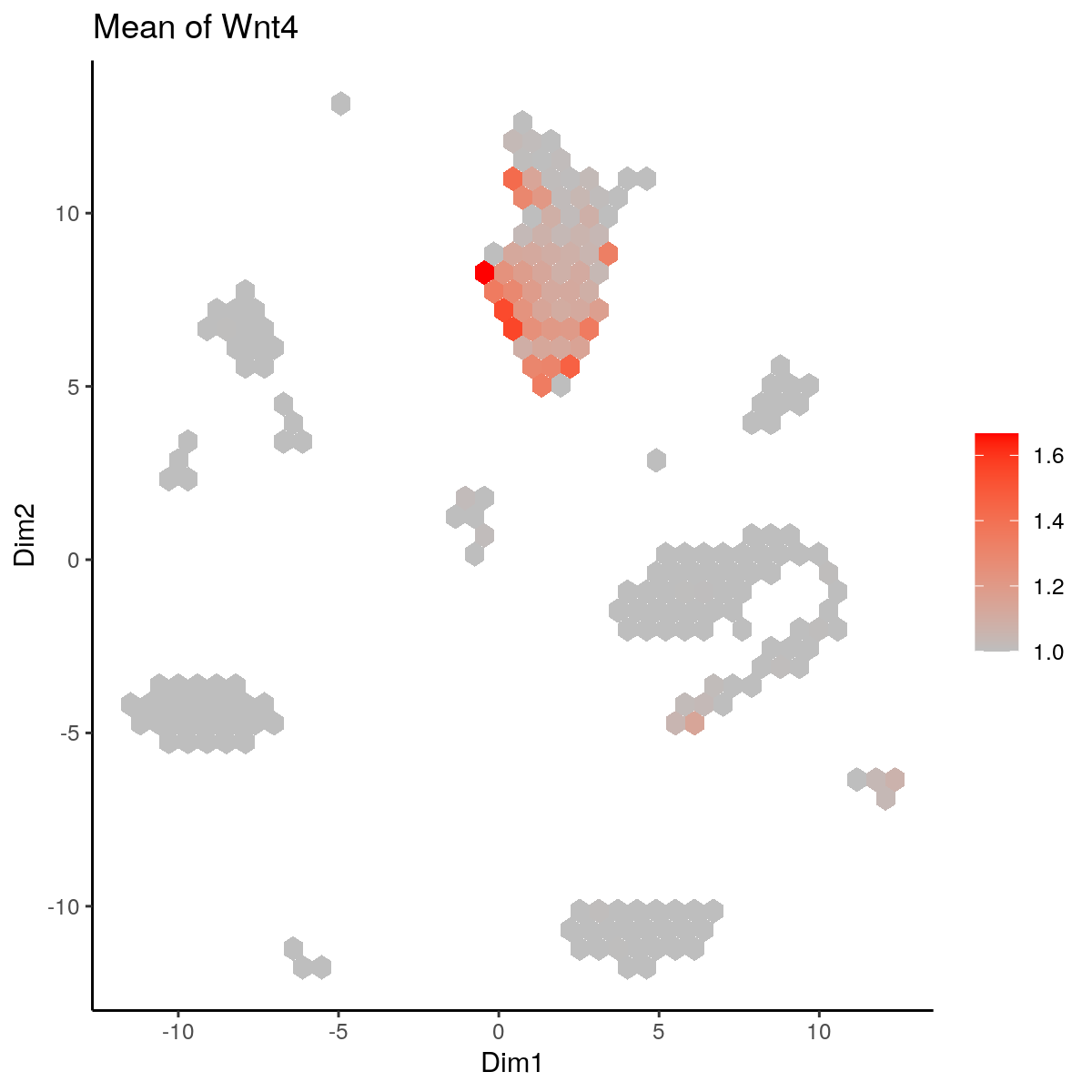

Supplement: Supplementary file 18 — Additional file 18. HTML report of VisualCortex. [file 12859_2023_5490_MOESM18_ESM.zip › output/report/Mouse_VisualCortex/figures/Ligand/22417.png]

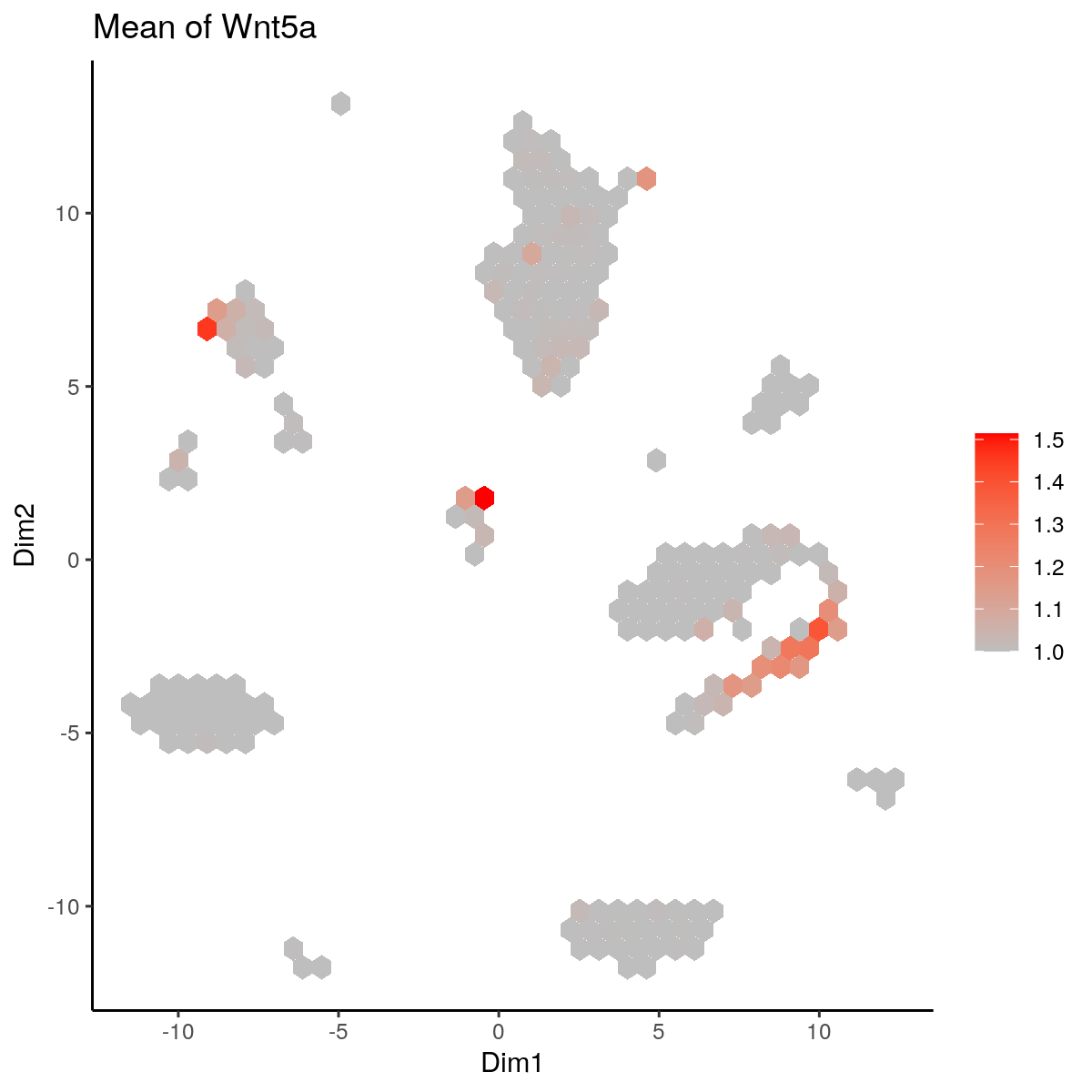

Supplement: Supplementary file 18 — Additional file 18. HTML report of VisualCortex. [file 12859_2023_5490_MOESM18_ESM.zip › output/report/Mouse_VisualCortex/figures/Ligand/22418.png]

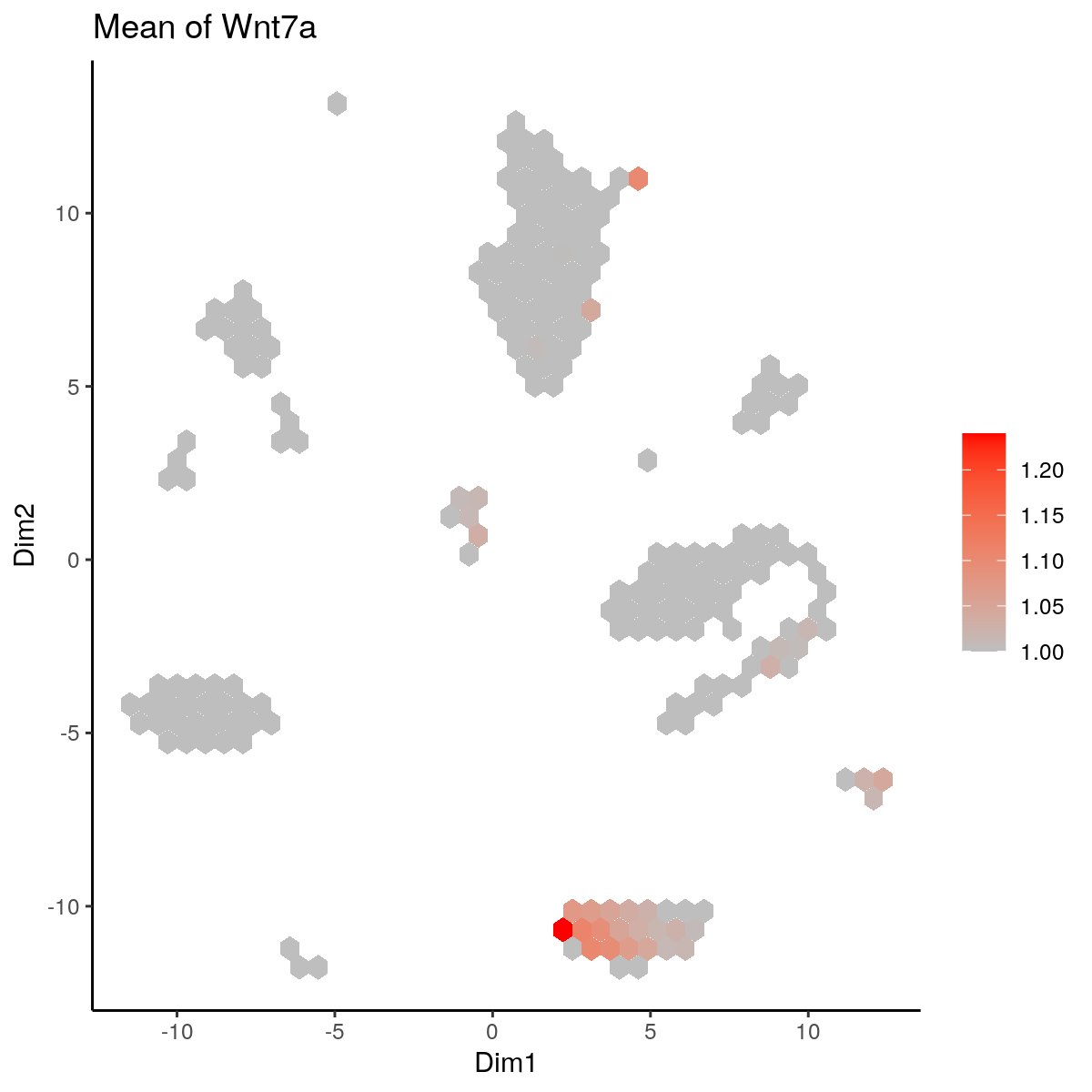

Supplement: Supplementary file 18 — Additional file 18. HTML report of VisualCortex. [file 12859_2023_5490_MOESM18_ESM.zip › output/report/Mouse_VisualCortex/figures/Ligand/22421.png]

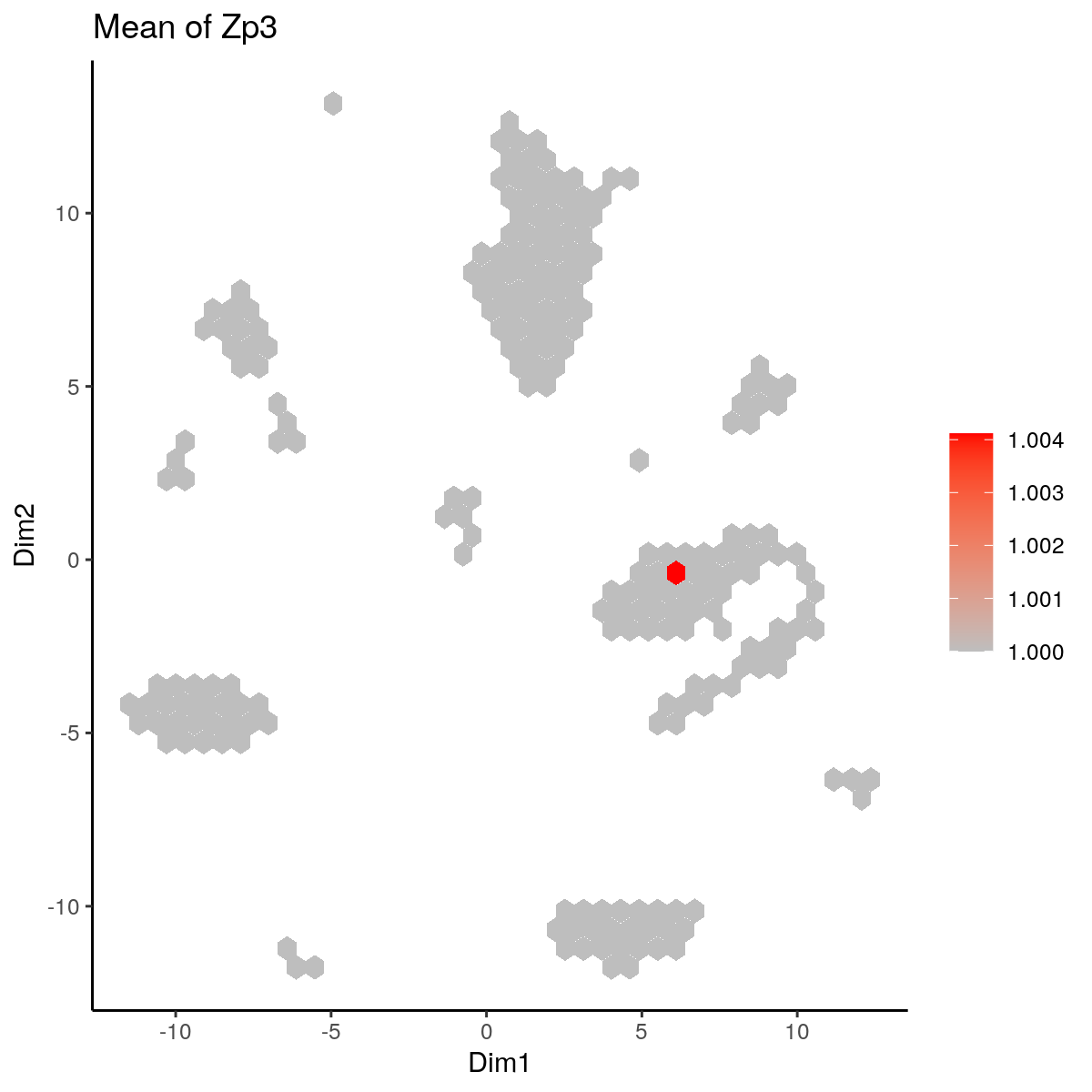

Supplement: Supplementary file 18 — Additional file 18. HTML report of VisualCortex. [file 12859_2023_5490_MOESM18_ESM.zip › output/report/Mouse_VisualCortex/figures/Ligand/22788.png]

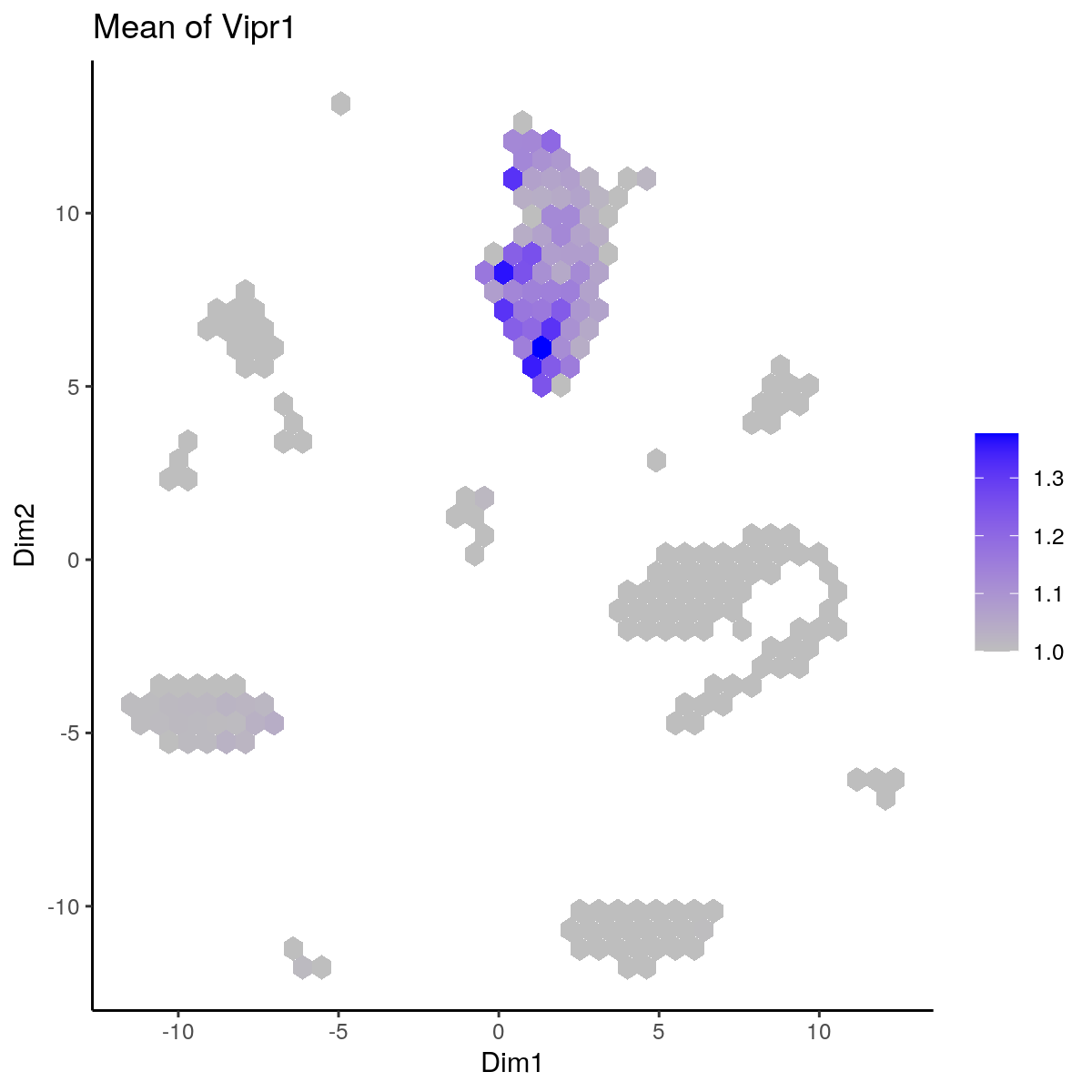

Supplement: Supplementary file 18 — Additional file 18. HTML report of VisualCortex. [file 12859_2023_5490_MOESM18_ESM.zip › output/report/Mouse_VisualCortex/figures/Receptor/22354.png]

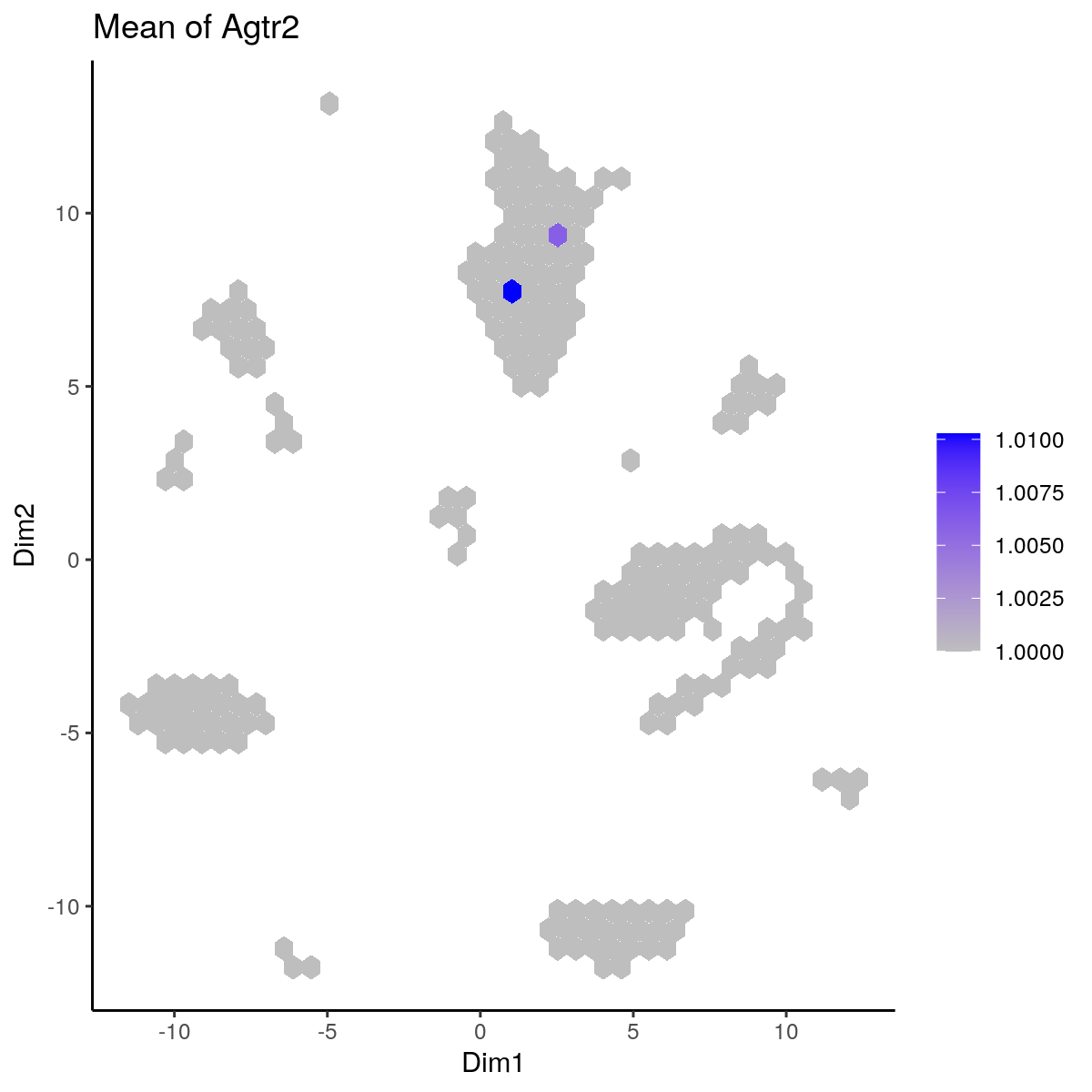

Supplement: Supplementary file 18 — Additional file 18. HTML report of VisualCortex. [file 12859_2023_5490_MOESM18_ESM.zip › output/report/Mouse_VisualCortex/figures/Receptor/11609.png]

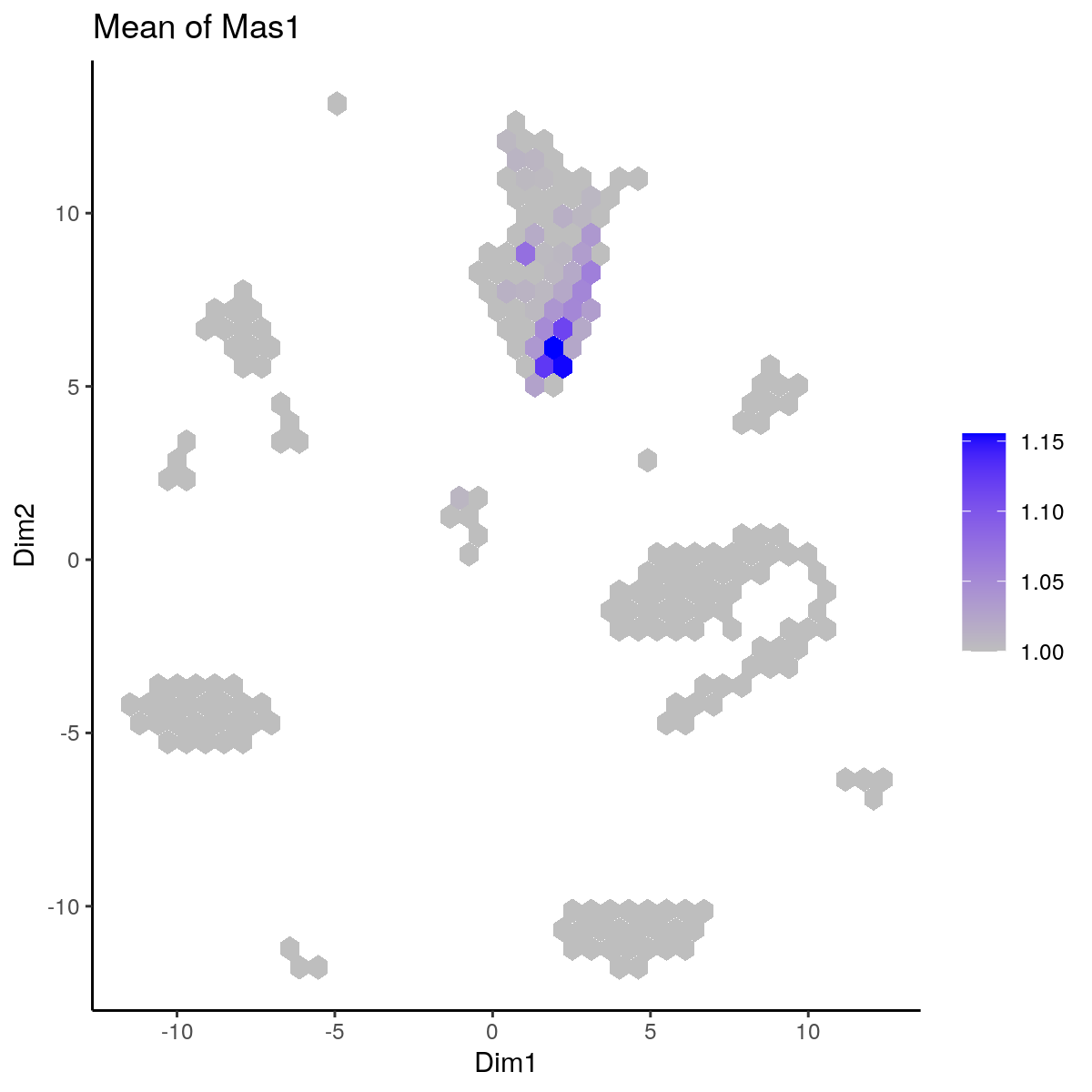

Supplement: Supplementary file 18 — Additional file 18. HTML report of VisualCortex. [file 12859_2023_5490_MOESM18_ESM.zip › output/report/Mouse_VisualCortex/figures/Receptor/17171.png]

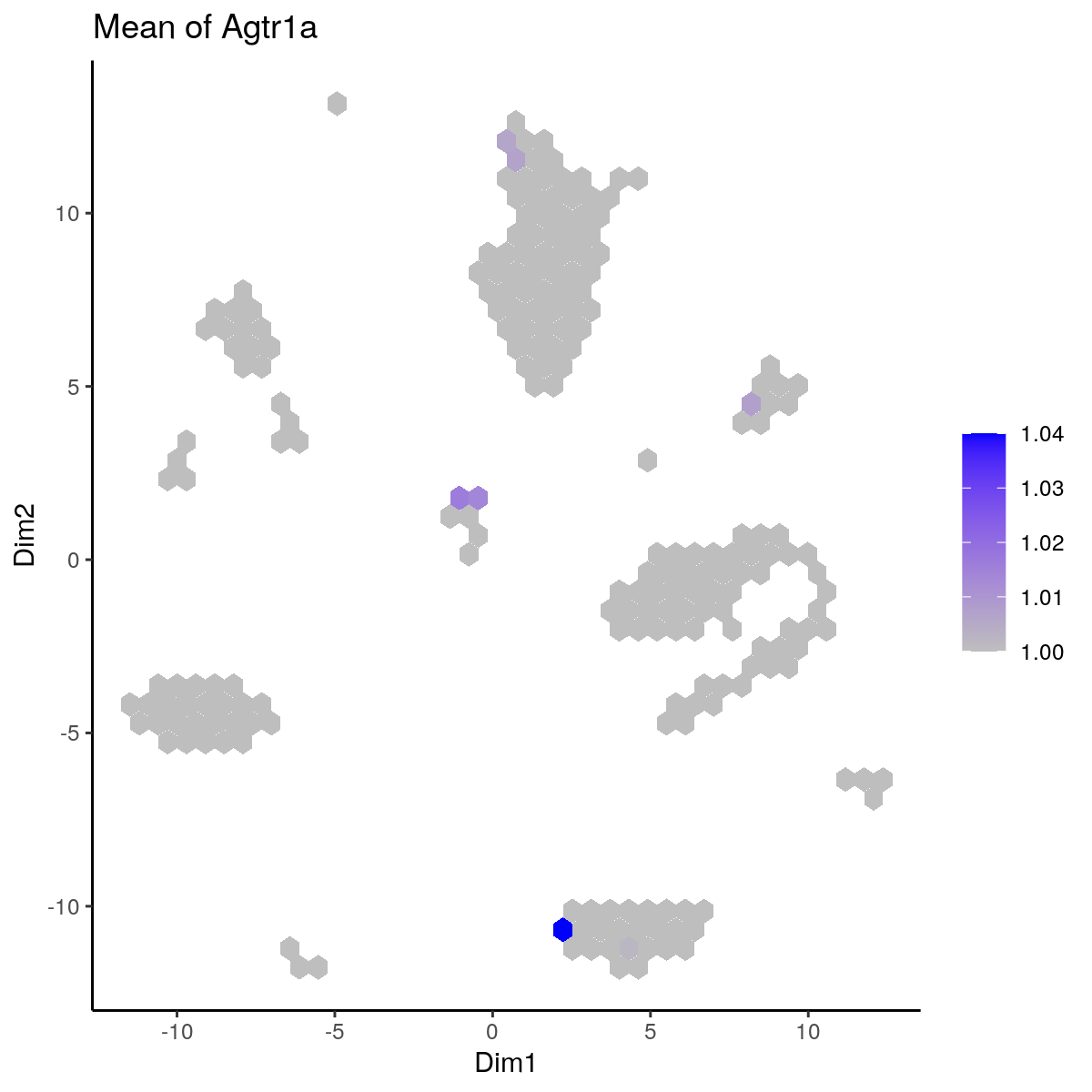

Supplement: Supplementary file 18 — Additional file 18. HTML report of VisualCortex. [file 12859_2023_5490_MOESM18_ESM.zip › output/report/Mouse_VisualCortex/figures/Receptor/11607.png]
