# Supplementary material for: Sctensor detects many-to-many cell–cell interactions from single cell RNA-sequencing data
Source: BMC Bioinformatics. 2023 Nov 7;24:420. doi: 10.1186/s12859-023-05490-y (PMC10631077; doi:10.1186/s12859-023-05490-y)
Supplement: Supplementary file 14 — Additional file 14. HTML report of FetalKidney. [file 12859_2023_5490_MOESM14_ESM.zip › output/report/Human_FetalKidney/ligand.html]

Details of Ligand Gene-centric Overview (selected)


# Details of Ligand Gene-centric Overview (selected)

```
library("scTensor")
load("reanalysis.RData")
scTensor:::.myvisNetwork(g)
```

| Rank | No. of related pairs | Ligand Gene | Receptor Genes | Related CCIs |
| --- | --- | --- | --- | --- |
| 1 | 16 | LAMB1 | ITGA6 | See the details of (1,1,)-related L-R patterns See the details of (1,2,)-related L-R patterns See the details of (4,2,)-related L-R patterns See the details of (1,3,)-related L-R patterns See the details of (2,2,)-related L-R patterns See the details of (1,5,)-related L-R patterns See the details of (3,2,)-related L-R patterns See the details of (1,4,)-related L-R patterns See the details of (4,1,)-related L-R patterns See the details of (4,5,)-related L-R patterns See the details of (3,1,)-related L-R patterns See the details of (4,3,)-related L-R patterns See the details of (3,5,)-related L-R patterns See the details of (2,5,)-related L-R patterns See the details of (4,4,)-related L-R patterns See the details of (2,1,)-related L-R patterns |
| 2 | 13 | COL4A1 | ITGA1  ITGB8 | See the details of (3,3,)-related L-R patterns See the details of (1,2,)-related L-R patterns See the details of (4,2,)-related L-R patterns See the details of (1,3,)-related L-R patterns See the details of (2,2,)-related L-R patterns See the details of (3,2,)-related L-R patterns See the details of (4,1,)-related L-R patterns See the details of (3,1,)-related L-R patterns See the details of (4,3,)-related L-R patterns See the details of (4,4,)-related L-R patterns See the details of (2,1,)-related L-R patterns |
| 3 | 11 | EFNB2 | EPHA4  EPHB3  EPHB6 | See the details of (1,2,)-related L-R patterns See the details of (2,2,)-related L-R patterns See the details of (3,2,)-related L-R patterns See the details of (3,5,)-related L-R patterns See the details of (2,5,)-related L-R patterns See the details of (2,1,)-related L-R patterns |
| 4 | 10 | CALM1 | MYLK | See the details of (1,1,)-related L-R patterns See the details of (3,3,)-related L-R patterns See the details of (2,3,)-related L-R patterns See the details of (1,3,)-related L-R patterns See the details of (1,4,)-related L-R patterns See the details of (4,1,)-related L-R patterns See the details of (3,1,)-related L-R patterns See the details of (4,3,)-related L-R patterns See the details of (4,4,)-related L-R patterns See the details of (2,1,)-related L-R patterns |
| 6 | 7 | FN1 | ITGA8  ITGB8  ITGB6 | See the details of (3,3,)-related L-R patterns See the details of (3,2,)-related L-R patterns See the details of (3,4,)-related L-R patterns See the details of (3,1,)-related L-R patterns See the details of (4,4,)-related L-R patterns See the details of (2,1,)-related L-R patterns |
| 6 | 7 | SEMA3B | NRP1 | See the details of (2,4,)-related L-R patterns See the details of (2,3,)-related L-R patterns See the details of (1,3,)-related L-R patterns See the details of (2,2,)-related L-R patterns See the details of (2,5,)-related L-R patterns See the details of (4,4,)-related L-R patterns See the details of (2,1,)-related L-R patterns |
| 6 | 7 | CXCL12 | CXCR4 | See the details of (2,4,)-related L-R patterns See the details of (4,2,)-related L-R patterns See the details of (2,2,)-related L-R patterns See the details of (3,2,)-related L-R patterns See the details of (3,5,)-related L-R patterns See the details of (2,5,)-related L-R patterns See the details of (4,4,)-related L-R patterns |
| 8 | 6 | VEGFA | NRP1 | See the details of (2,4,)-related L-R patterns See the details of (2,3,)-related L-R patterns See the details of (2,2,)-related L-R patterns See the details of (2,5,)-related L-R patterns See the details of (4,4,)-related L-R patterns See the details of (2,1,)-related L-R patterns |
| 9 | 5 | NPNT | ITGA8 | See the details of (2,4,)-related L-R patterns See the details of (2,3,)-related L-R patterns See the details of (1,3,)-related L-R patterns See the details of (1,4,)-related L-R patterns See the details of (2,1,)-related L-R patterns |
| 12 | 4 | SLIT2 | ROBO2 | See the details of (1,1,)-related L-R patterns See the details of (1,3,)-related L-R patterns See the details of (1,4,)-related L-R patterns See the details of (2,1,)-related L-R patterns |
| 12 | 4 | EFNA5 | EPHA7  EPHA4 | See the details of (1,2,)-related L-R patterns See the details of (4,2,)-related L-R patterns |
| 12 | 4 | EFNA1 | EPHA7  EPHA4 | See the details of (1,2,)-related L-R patterns See the details of (4,2,)-related L-R patterns |
| 12 | 4 | BMP7 | ACVR2B | See the details of (1,2,)-related L-R patterns See the details of (4,2,)-related L-R patterns See the details of (2,2,)-related L-R patterns See the details of (4,4,)-related L-R patterns |
| 12 | 4 | LAMA1 | ITGB8 | See the details of (1,2,)-related L-R patterns See the details of (4,2,)-related L-R patterns See the details of (4,1,)-related L-R patterns See the details of (4,4,)-related L-R patterns |
| 15 | 3 | FGF9 | FGFR3 | See the details of (4,2,)-related L-R patterns See the details of (2,2,)-related L-R patterns See the details of (4,4,)-related L-R patterns |
| 16.5 | 2 | PDGFA | PDGFRA | See the details of (1,3,)-related L-R patterns See the details of (4,3,)-related L-R patterns |
| 16.5 | 2 | FGF1 | FGFR3 | See the details of (2,2,)-related L-R patterns See the details of (2,1,)-related L-R patterns |
| 21.5 | 1 | COL1A1 | DDR2 | See the details of (3,3,)-related L-R patterns |
| 21.5 | 1 | NCAM1 | ROBO3 | See the details of (1,2,)-related L-R patterns |
| 21.5 | 1 | SOST | LRP5 | See the details of (2,2,)-related L-R patterns |
| 21.5 | 1 | HAS2 | HMMR | See the details of (3,5,)-related L-R patterns |
| 21.5 | 1 | APOE | SORL1 | See the details of (4,4,)-related L-R patterns |
| 21.5 | 1 | NTN4 | DCC | See the details of (4,4,)-related L-R patterns |
| 21.5 | 1 | KNG1 | GPR135 | See the details of (4,4,)-related L-R patterns |
| 21.5 | 1 | PROS1 | TYRO3 | See the details of (2,1,)-related L-R patterns |
