# Supplementary material for: Sctensor detects many-to-many cell–cell interactions from single cell RNA-sequencing data
Source: BMC Bioinformatics. 2023 Nov 7;24:420. doi: 10.1186/s12859-023-05490-y (PMC10631077; doi:10.1186/s12859-023-05490-y)
Supplement: Supplementary file 14 — Additional file 14. HTML report of FetalKidney. [file 12859_2023_5490_MOESM14_ESM.zip › output/report/Human_FetalKidney/ligand_all.html]

Details of Ligand Gene-centric Overview (all)


# Details of Ligand Gene-centric Overview (all)

| Ligand Gene | Receptor Gene |
| --- | --- |
| A2M | LRP1 |
| ADAM12 | ITGA9 |
| ADAM15 | ITGA9 ITGB3 |
| ADAM2 | ITGA6 |
| ADAM23 | ITGB3 |
| ADAM9 | ITGA6 |
| ADM | RAMP1 |
| ADM2 | CALCR CALCRL RAMP1 RAMP2 RAMP3 |
| AGT | AGTR1 AGTR2 LRP2 MAS1 |
| AMH | ACVR1 AMHR2 BMPR1A BMPR1B |
| ANGPT1 | TEK TIE1 |
| ANGPT2 | TEK |
| ANXA1 | FPR1 FPR2 FPR3 |
| APELA | APLNR |
| APOE | LDLR LRP5 LRP8 SORL1 VLDLR |
| ARTN | GFRA3 RET |
| AVP | AVPR1A |
| BDNF | NTRK2 |
| BMP10 | ACVR2B |
| BMP15 | ACVR2B |
| BMP2 | ACVR1 ACVR2A ACVR2B ACVRL1 BMPR1A BMPR1B BMPR2 |
| BMP3 | ACVR1 ACVR2B BMPR1A BMPR1B BMPR2 |
| BMP4 | ACVR2B |
| BMP5 | ACVR2B |
| BMP6 | ACVR2B |
| BMP7 | ACVR1 ACVR2A ACVR2B ACVRL1 BMPR1A BMPR1B BMPR2 |
| BMP8A | ACVR2B |
| BMP8B | ACVR2B |
| BTC | ERBB3 ERBB4 |
| CALCA | CALCR CALCRL RAMP1 RAMP2 RAMP3 |
| CALCB | CALCR CALCRL RAMP1 RAMP2 RAMP3 |
| CALM1 | MYLK MYLK2 PDE1A PDE1B PDE1C |
| CALM2 | MYLK PDE1A PDE1C |
| CALM3 | MYLK PDE1A PDE1C |
| CCL5 | CCR1 LOC105377067 CCR3 CCR5 CXCR3 GPR75 |
| CD24 | SELP |
| CD34 | SELL |
| CFH | SELL |
| CLCF1 | CNTFR LIFR |
| CNTF | CNTFR LIFR |
| COL1A1 | DDR1 DDR2 ITGA11 ITGA2 ITGB1 |
| COL2A1 | ITGA10 ITGA2 ITGB1 |
| COL4A1 | ITGA1 ITGB1 ITGB8 |
| CRLF1 | CNTFR LIFR |
| CSF2 | CSF2RB |
| CSF3 | CSF2RB |
| CSH1 | PRLR |
| CSH2 | PRLR |
| CSHL1 | PRLR |
| CTF1 | LIFR |
| CXCL12 | ACKR3 CXCR3 CXCR4 |
| DKK1 | KREMEN1 KREMEN2 LRP6 |
| DLK1 | NOTCH1 NOTCH2 NOTCH3 NOTCH4 |
| DLL1 | NOTCH1 NOTCH2 NOTCH3 NOTCH4 |
| DUSP18 | ITGA1 ITGA3 ITGA6 |
| EDN1 | EDNRA |
| EDN2 | EDNRA |
| EDN3 | EDNRB |
| EFNA1 | EPHA1 EPHA2 EPHA3 EPHA4 EPHA5 EPHA7 EPHA8 EPHA6 |
| EFNA2 | EPHA4 EPHA7 |
| EFNA3 | EPHA4 EPHA7 |
| EFNA4 | EPHA4 EPHA7 |
| EFNA5 | EPHA1 EPHA2 EPHA3 EPHA4 EPHA5 EPHA7 EPHA8 EPHA6 EPHB2 |
| EFNB1 | EPHA4 EPHB3 EPHB6 |
| EFNB2 | EPHA4 EPHB1 EPHB2 EPHB3 EPHB4 EPHB6 |
| EFNB3 | EPHA4 EPHB3 EPHB6 |
| EGF | EGFR |
| EREG | ERBB4 |
| F7 | F3 |
| F8 | LRP1 |
| FGF1 | FGFR1 FGFR2 FGFR3 FGFR4 |
| FGF10 | FGFR3 |
| FGF11 | FGFR3 |
| FGF12 | FGFR3 |
| FGF13 | FGFR1 FGFR2 FGFR3 FGFR4 |
| FGF14 | FGFR3 |
| FGF16 | FGFR3 |
| FGF17 | FGFR3 |
| FGF18 | FGFR3 |
| FGF19 | FGFR3 |
| FGF2 | FGFR3 |
| FGF4 | FGFR3 |
| FGF5 | FGFR3 |
| FGF8 | FGFR2 FGFR3 FGFR4 FGFR1 |
| FGF9 | FGFR1 FGFR2 FGFR3 FGFR4 |
| FN1 | ITGA2 ITGA2B ITGA4 ITGA5 ITGA8 ITGB1 ITGB3 ITGB6 ITGB8 SDC2 |
| GAS6 | TYRO3 |
| GDF1 | ACVR2B |
| GDF10 | ACVR2B |
| GDF11 | ACVR2B |
| GDF2 | ACVR2B |
| GDF3 | ACVR2B |
| GDF7 | ACVR2B |
| GDF9 | ACVR2B |
| GDNF | GFRA1 GFRA2 RET |
| GH1 | GHR |
| GH2 | GHR |
| GHRL | GHSR GPR39 |
| GPHA2 | TSHR |
| HAS2 | CD44 HMMR |
| HBEGF | ERBB4 |
| HDC | HRH1 |
| HLA-A | KIR3DL2 |
| HLA-B | KIR3DL1 |
| HLA-C | KIR2DL1 KIR2DL3 KIR2DS4 |
| IAPP | RAMP1 |
| ICAM4 | ITGA2B ITGB3 |
| IFNA1 | IFNAR1 IFNAR2 |
| IFNA10 | IFNAR1 IFNAR2 |
| IFNA13 | IFNAR1 IFNAR2 |
| IFNA14 | IFNAR1 IFNAR2 |
| IFNA16 | IFNAR1 IFNAR2 |
| IFNA17 | IFNAR1 IFNAR2 |
| IFNA2 | IFNAR1 IFNAR2 |
| IFNA21 | IFNAR1 IFNAR2 |
| IFNA4 | IFNAR1 IFNAR2 |
| IFNA5 | IFNAR1 IFNAR2 |
| IFNA6 | IFNAR1 IFNAR2 |
| IFNA7 | IFNAR1 IFNAR2 |
| IFNA8 | IFNAR1 IFNAR2 |
| IFNB1 | IFNAR1 IFNAR2 |
| IFNK | IFNAR1 IFNAR2 |
| IFNL1 | IFNLR1 |
| IFNL2 | IFNLR1 |
| IFNL3 | IFNLR1 |
| IFNW1 | IFNAR1 IFNAR2 |
| IGF2 | IGF1R IGF2R INSR |
| IL18 | IL18R1 IL1RAPL1 |
| IL1A | IL1R1 |
| IL1B | IL1R1 |
| IL1R1 | IL1R2 |
| IL1RN | IL1R1 |
| IL3 | CSF2RB |
| IL37 | IL18R1 |
| IL5 | CSF2RB |
| IL6 | IL6R |
| INHA | ACVR2B TGFBR3 |
| INHBA | ACVR1B ACVR1C ACVR2A ACVR2B ACVR1 BAMBI |
| INHBB | ACVR2B |
| INHBC | ACVR2B |
| JAG1 | NOTCH1 NOTCH2 NOTCH3 NOTCH4 |
| KITLG | KIT |
| KNG1 | BDKRB1 BDKRB2 GPR135 |
| LAMA1 | ITGA7 ITGB1 ITGB8 RPSA |
| LAMA2 | ITGA7 ITGB1 RPSA |
| LAMB1 | ITGA6 ITGB1 |
| LAMC2 | ITGA3 ITGA6 ITGB1 ITGB4 |
| LEFTY2 | ACVR2B |
| LEP | LEPR |
| LIF | LIFR |
| LTB | LTBR CD40 |
| MADCAM1 | ITGA4 |
| NCAM1 | ROBO1 ROBO3 |
| NID1 | PTPRF |
| NLGN1 | NRXN1 NRXN3 |
| NLGN2 | NRXN1 NRXN3 |
| NLGN3 | NRXN1 NRXN3 |
| NMU | NMUR1 NMUR2 |
| NODAL | ACVR2B |
| NPNT | ITGA8 ITGB1 |
| NPPA | NPR1 |
| NPPB | NPR1 |
| NPW | NPBWR1 NPBWR2 |
| NPY | NPY5R |
| NRG1 | ERBB3 ERBB4 ERBB2 |
| NRG2 | ERBB3 ERBB4 |
| NRG3 | ERBB4 |
| NRG4 | ERBB4 |
| NRTN | GFRA1 RET |
| NTF3 | NTRK2 NTRK3 |
| NTF4 | NTRK2 |
| NTN1 | DCC |
| NTN4 | DCC UNC5A |
| NTNG1 | LRRC4C |
| NUCB2 | ERAP1 |
| NXPH1 | NRXN1 NRXN3 |
| NXPH3 | NRXN1 NRXN3 |
| OSM | LIFR |
| OXT | AVPR1A |
| PDGFA | PDGFRA |
| PDGFB | PDGFRA PDGFRB |
| PDGFC | PDGFRA |
| PF4 | FGFR2 LDLR PROCR THBD |
| PGF | NRP1 |
| PLAU | LRP1 PLAUR |
| PLG | ITGA9 |
| PODXL2 | SELL |
| PPBP | CXCR2 CXCR1 |
| PPY | NPY5R |
| PRL | PRLR |
| PROS1 | AXL TYRO3 |
| PSPN | RET |
| PTH | PTH1R |
| PTH2 | PTH1R |
| PTHLH | PTH1R |
| PTN | PTPRB PTPRZ1 |
| PYY | NPY5R |
| RELN | ITGA3 |
| REN | ATP6AP2 |
| RSPO1 | LGR4 LGR5 LGR6 LRP6 |
| RSPO2 | LGR5 |
| RSPO3 | LGR4 LGR5 LGR6 |
| RSPO4 | LGR5 |
| SEMA3A | NRP1 PLXNA1 PLXNA2 PLXNA4 |
| SEMA3B | NRP1 |
| SEMA3C | NRP2 |
| SEMA3E | PLXND1 |
| SEMA3F | NRP2 |
| SEMA5A | MET PLXNB3 |
| SEMA6D | KDR PLXNA1 |
| SEMA7A | PLXNC1 |
| SERPINA1 | LRP1 |
| SLIT2 | GPC1 ROBO1 ROBO2 ROBO4 SDC1 |
| SOST | LRP5 LRP6 |
| SPP1 | CD44 ITGAV |
| SPX | GALR2 GALR3 |
| TGFB1 | TGFBR3 |
| TGFB2 | TGFBR3 |
| TGFB3 | TGFBR3 |
| THBS1 | CD36 CD47 SDC1 |
| TNFSF10 | TNFRSF10A TNFRSF10B TNFRSF10C TNFRSF10D TNFRSF11B |
| TNFSF11 | TNFRSF11B |
| TNFSF12 | TNFRSF12A |
| TNFSF15 | TNFRSF25 |
| TPH1 | HTR1F HTR2A |
| VCAM1 | ITGA4 ITGAD ITGB1 ITGB2 |
| VEGFA | FLT1 KDR NRP1 |
| VEGFB | NRP1 |
| VEGFC | NRP2 |
| VTN | ITGB3 |
| WNT1 | FZD3 |
| WNT11 | FZD7 |
| WNT4 | FZD6 FZD2 |
| WNT5A | FZD7 |
| WNT7A | FZD5 |
| WNT7B | FZD1 FZD10 LRP5 |
