# Supplementary material for: Sctensor detects many-to-many cell–cell interactions from single cell RNA-sequencing data
Source: BMC Bioinformatics. 2023 Nov 7;24:420. doi: 10.1186/s12859-023-05490-y (PMC10631077; doi:10.1186/s12859-023-05490-y)
Supplement: Supplementary file 14 — Additional file 14. HTML report of FetalKidney. [file 12859_2023_5490_MOESM14_ESM.zip › output/report/Human_FetalKidney/receptor.html]

Details of Receptor Gene-centric Overview (selected)


# Details of Receptor Gene-centric Overview (selected)

```
library("scTensor")
load("reanalysis.RData")
scTensor:::.myvisNetwork(g)
```

| Rank | No. of related pairs | Receptor Gene | Ligand Genes | Related CCIs |
| --- | --- | --- | --- | --- |
| 1 | 16 | ITGA6 | LAMB1 | See the details of (1,1,)-related L-R patterns See the details of (1,2,)-related L-R patterns See the details of (4,2,)-related L-R patterns See the details of (1,3,)-related L-R patterns See the details of (2,2,)-related L-R patterns See the details of (1,5,)-related L-R patterns See the details of (3,2,)-related L-R patterns See the details of (1,4,)-related L-R patterns See the details of (4,1,)-related L-R patterns See the details of (4,5,)-related L-R patterns See the details of (3,1,)-related L-R patterns See the details of (4,3,)-related L-R patterns See the details of (3,5,)-related L-R patterns See the details of (2,5,)-related L-R patterns See the details of (4,4,)-related L-R patterns See the details of (2,1,)-related L-R patterns |
| 2 | 15 | ITGB8 | COL4A1  LAMA1  FN1 | See the details of (1,2,)-related L-R patterns See the details of (4,2,)-related L-R patterns See the details of (2,2,)-related L-R patterns See the details of (3,2,)-related L-R patterns See the details of (4,1,)-related L-R patterns See the details of (3,1,)-related L-R patterns See the details of (4,4,)-related L-R patterns See the details of (2,1,)-related L-R patterns |
| 3 | 13 | NRP1 | VEGFA  SEMA3B | See the details of (2,4,)-related L-R patterns See the details of (2,3,)-related L-R patterns See the details of (1,3,)-related L-R patterns See the details of (2,2,)-related L-R patterns See the details of (2,5,)-related L-R patterns See the details of (4,4,)-related L-R patterns See the details of (2,1,)-related L-R patterns |
| 4 | 10 | MYLK | CALM1 | See the details of (1,1,)-related L-R patterns See the details of (3,3,)-related L-R patterns See the details of (2,3,)-related L-R patterns See the details of (1,3,)-related L-R patterns See the details of (1,4,)-related L-R patterns See the details of (4,1,)-related L-R patterns See the details of (3,1,)-related L-R patterns See the details of (4,3,)-related L-R patterns See the details of (4,4,)-related L-R patterns See the details of (2,1,)-related L-R patterns |
| 5.5 | 8 | ITGA8 | FN1  NPNT | See the details of (3,3,)-related L-R patterns See the details of (2,4,)-related L-R patterns See the details of (2,3,)-related L-R patterns See the details of (1,3,)-related L-R patterns See the details of (1,4,)-related L-R patterns See the details of (3,4,)-related L-R patterns See the details of (4,4,)-related L-R patterns See the details of (2,1,)-related L-R patterns |
| 5.5 | 8 | EPHA4 | EFNB2  EFNA5  EFNA1 | See the details of (1,2,)-related L-R patterns See the details of (4,2,)-related L-R patterns See the details of (2,2,)-related L-R patterns See the details of (3,2,)-related L-R patterns See the details of (2,5,)-related L-R patterns |
| 7 | 7 | CXCR4 | CXCL12 | See the details of (2,4,)-related L-R patterns See the details of (4,2,)-related L-R patterns See the details of (2,2,)-related L-R patterns See the details of (3,2,)-related L-R patterns See the details of (3,5,)-related L-R patterns See the details of (2,5,)-related L-R patterns See the details of (4,4,)-related L-R patterns |
| 8.5 | 5 | ITGA1 | COL4A1 | See the details of (3,3,)-related L-R patterns See the details of (4,2,)-related L-R patterns See the details of (1,3,)-related L-R patterns See the details of (4,3,)-related L-R patterns See the details of (4,4,)-related L-R patterns |
| 8.5 | 5 | FGFR3 | FGF9  FGF1 | See the details of (4,2,)-related L-R patterns See the details of (2,2,)-related L-R patterns See the details of (4,4,)-related L-R patterns See the details of (2,1,)-related L-R patterns |
| 11.5 | 4 | ROBO2 | SLIT2 | See the details of (1,1,)-related L-R patterns See the details of (1,3,)-related L-R patterns See the details of (1,4,)-related L-R patterns See the details of (2,1,)-related L-R patterns |
| 11.5 | 4 | EPHA7 | EFNA5  EFNA1 | See the details of (1,2,)-related L-R patterns See the details of (4,2,)-related L-R patterns |
| 11.5 | 4 | ACVR2B | BMP7 | See the details of (1,2,)-related L-R patterns See the details of (4,2,)-related L-R patterns See the details of (2,2,)-related L-R patterns See the details of (4,4,)-related L-R patterns |
| 11.5 | 4 | EPHB3 | EFNB2 | See the details of (1,2,)-related L-R patterns See the details of (2,2,)-related L-R patterns See the details of (3,5,)-related L-R patterns See the details of (2,5,)-related L-R patterns |
| 14 | 3 | EPHB6 | EFNB2 | See the details of (1,2,)-related L-R patterns See the details of (2,2,)-related L-R patterns See the details of (2,1,)-related L-R patterns |
| 15 | 2 | PDGFRA | PDGFA | See the details of (1,3,)-related L-R patterns See the details of (4,3,)-related L-R patterns |
| 20 | 1 | DDR2 | COL1A1 | See the details of (3,3,)-related L-R patterns |
| 20 | 1 | ROBO3 | NCAM1 | See the details of (1,2,)-related L-R patterns |
| 20 | 1 | LRP5 | SOST | See the details of (2,2,)-related L-R patterns |
| 20 | 1 | ITGB6 | FN1 | See the details of (3,2,)-related L-R patterns |
| 20 | 1 | HMMR | HAS2 | See the details of (3,5,)-related L-R patterns |
| 20 | 1 | SORL1 | APOE | See the details of (4,4,)-related L-R patterns |
| 20 | 1 | DCC | NTN4 | See the details of (4,4,)-related L-R patterns |
| 20 | 1 | GPR135 | KNG1 | See the details of (4,4,)-related L-R patterns |
| 20 | 1 | TYRO3 | PROS1 | See the details of (2,1,)-related L-R patterns |
