# Supplementary material for: Sctensor detects many-to-many cell–cell interactions from single cell RNA-sequencing data
Source: BMC Bioinformatics. 2023 Nov 7;24:420. doi: 10.1186/s12859-023-05490-y (PMC10631077; doi:10.1186/s12859-023-05490-y)
Supplement: Supplementary file 14 — Additional file 14. HTML report of FetalKidney. [file 12859_2023_5490_MOESM14_ESM.zip › output/report/Human_FetalKidney/receptor_all.html]

Details of Receptor Gene-centric Overview (all)


# Details of Receptor Gene-centric Overview (all)

| Receptor Gene | Ligand Gene |
| --- | --- |
| ACKR3 | CXCL12 |
| ACVR1 | AMH BMP2 BMP3 BMP7 INHBA |
| ACVR1B | INHBA |
| ACVR1C | INHBA |
| ACVR2A | BMP2 BMP7 INHBA |
| ACVR2B | BMP2 BMP3 BMP7 INHBA GDF2 BMP10 GDF3 BMP8B GDF9 BMP8A GDF10 BMP5 BMP4 INHBB GDF1 INHA BMP6 GDF7 BMP15 INHBC GDF11 LEFTY2 NODAL |
| ACVRL1 | BMP2 BMP7 |
| AGTR1 | AGT |
| AGTR2 | AGT |
| AMHR2 | AMH |
| APLNR | APELA |
| ATP6AP2 | REN |
| AVPR1A | AVP OXT |
| AXL | PROS1 |
| BAMBI | INHBA |
| BDKRB1 | KNG1 |
| BDKRB2 | KNG1 |
| BMPR1A | AMH BMP2 BMP3 BMP7 |
| BMPR1B | AMH BMP2 BMP3 BMP7 |
| BMPR2 | BMP2 BMP3 BMP7 |
| CALCR | ADM2 CALCA CALCB |
| CALCRL | ADM2 CALCA CALCB |
| CCR1 | CCL5 |
| CCR3 | CCL5 |
| CCR5 | CCL5 |
| CD36 | THBS1 |
| CD40 | LTB |
| CD44 | HAS2 SPP1 |
| CD47 | THBS1 |
| CNTFR | CLCF1 CRLF1 CNTF |
| CSF2RB | CSF2 IL3 CSF3 IL5 |
| CXCR1 | PPBP |
| CXCR2 | PPBP |
| CXCR3 | CCL5 CXCL12 |
| CXCR4 | CXCL12 |
| DCC | NTN4 NTN1 |
| DDR1 | COL1A1 |
| DDR2 | COL1A1 |
| EDNRA | EDN1 EDN2 |
| EDNRB | EDN3 |
| EGFR | EGF |
| EPHA1 | EFNA1 EFNA5 |
| EPHA2 | EFNA1 EFNA5 |
| EPHA3 | EFNA1 EFNA5 |
| EPHA4 | EFNA1 EFNA5 EFNB2 EFNA4 EFNB3 EFNB1 EFNA2 EFNA3 |
| EPHA5 | EFNA1 EFNA5 |
| EPHA6 | EFNA1 EFNA5 |
| EPHA7 | EFNA1 EFNA5 EFNA2 EFNA4 EFNA3 |
| EPHA8 | EFNA1 EFNA5 |
| EPHB1 | EFNB2 |
| EPHB2 | EFNA5 EFNB2 |
| EPHB3 | EFNB2 EFNB3 EFNB1 |
| EPHB4 | EFNB2 |
| EPHB6 | EFNB2 EFNB1 EFNB3 |
| ERAP1 | NUCB2 |
| ERBB2 | NRG1 |
| ERBB3 | NRG1 NRG2 BTC |
| ERBB4 | NRG1 BTC NRG2 HBEGF NRG3 NRG4 EREG |
| F3 | F7 |
| FGFR1 | FGF1 FGF13 FGF8 FGF9 |
| FGFR2 | FGF1 FGF13 FGF8 FGF9 PF4 |
| FGFR3 | FGF1 FGF13 FGF8 FGF9 FGF2 FGF18 FGF10 FGF17 FGF14 FGF19 FGF4 FGF12 FGF16 FGF11 FGF5 |
| FGFR4 | FGF1 FGF13 FGF8 FGF9 |
| FLT1 | VEGFA |
| FPR1 | ANXA1 |
| FPR2 | ANXA1 |
| FPR3 | ANXA1 |
| FZD1 | WNT7B |
| FZD10 | WNT7B |
| FZD2 | WNT4 |
| FZD3 | WNT1 |
| FZD5 | WNT7A |
| FZD6 | WNT4 |
| FZD7 | WNT11 WNT5A |
| GALR2 | SPX |
| GALR3 | SPX |
| GFRA1 | GDNF NRTN |
| GFRA2 | GDNF |
| GFRA3 | ARTN |
| GHR | GH1 GH2 |
| GHSR | GHRL |
| GPC1 | SLIT2 |
| GPR135 | KNG1 |
| GPR39 | GHRL |
| GPR75 | CCL5 |
| HMMR | HAS2 |
| HRH1 | HDC |
| HTR1F | TPH1 |
| HTR2A | TPH1 |
| IFNAR1 | IFNA13 IFNA16 IFNA7 IFNA21 IFNA2 IFNA1 IFNA4 IFNA17 IFNA10 IFNK IFNA5 IFNW1 IFNA6 IFNB1 IFNA14 IFNA8 |
| IFNAR2 | IFNA17 IFNA21 IFNA6 IFNA2 IFNA16 IFNA7 IFNA10 IFNA13 IFNW1 IFNA4 IFNK IFNA5 IFNA1 IFNA14 IFNB1 IFNA8 |
| IFNLR1 | IFNL2 IFNL3 IFNL1 |
| IGF1R | IGF2 |
| IGF2R | IGF2 |
| IL18R1 | IL37 IL18 |
| IL1R1 | IL1B IL1RN IL1A |
| IL1R2 | IL1R1 |
| IL1RAPL1 | IL18 |
| IL6R | IL6 |
| INSR | IGF2 |
| ITGA1 | COL4A1 DUSP18 |
| ITGA10 | COL2A1 |
| ITGA11 | COL1A1 |
| ITGA2 | COL1A1 COL2A1 FN1 |
| ITGA2B | FN1 ICAM4 |
| ITGA3 | LAMC2 DUSP18 RELN |
| ITGA4 | FN1 VCAM1 MADCAM1 |
| ITGA5 | FN1 |
| ITGA6 | LAMB1 LAMC2 ADAM9 ADAM2 DUSP18 |
| ITGA7 | LAMA1 LAMA2 |
| ITGA8 | FN1 NPNT |
| ITGA9 | PLG ADAM12 ADAM15 |
| ITGAD | VCAM1 |
| ITGAV | SPP1 |
| ITGB1 | COL1A1 COL2A1 COL4A1 FN1 LAMA1 LAMA2 LAMB1 LAMC2 NPNT VCAM1 |
| ITGB2 | VCAM1 |
| ITGB3 | FN1 ICAM4 VTN ADAM23 ADAM15 |
| ITGB4 | LAMC2 |
| ITGB6 | FN1 |
| ITGB8 | COL4A1 FN1 LAMA1 |
| KDR | SEMA6D VEGFA |
| KIR2DL1 | HLA-C |
| KIR2DL3 | HLA-C |
| KIR2DS4 | HLA-C |
| KIR3DL1 | HLA-B |
| KIR3DL2 | HLA-A |
| KIT | KITLG |
| KREMEN1 | DKK1 |
| KREMEN2 | DKK1 |
| LDLR | APOE PF4 |
| LEPR | LEP |
| LGR4 | RSPO1 RSPO3 |
| LGR5 | RSPO1 RSPO3 RSPO4 RSPO2 |
| LGR6 | RSPO1 RSPO3 |
| LIFR | CLCF1 CNTF CTF1 LIF CRLF1 OSM |
| LOC105377067 | CCL5 |
| LRP1 | A2M F8 PLAU SERPINA1 |
| LRP2 | AGT |
| LRP5 | APOE SOST WNT7B |
| LRP6 | DKK1 RSPO1 SOST |
| LRP8 | APOE |
| LRRC4C | NTNG1 |
| LTBR | LTB |
| MAS1 | AGT |
| MET | SEMA5A |
| MYLK | CALM1 CALM3 CALM2 |
| MYLK2 | CALM1 |
| NMUR1 | NMU |
| NMUR2 | NMU |
| NOTCH1 | DLK1 DLL1 JAG1 |
| NOTCH2 | DLK1 DLL1 JAG1 |
| NOTCH3 | DLK1 DLL1 JAG1 |
| NOTCH4 | DLK1 DLL1 JAG1 |
| NPBWR1 | NPW |
| NPBWR2 | NPW |
| NPR1 | NPPA NPPB |
| NPY5R | PYY NPY PPY |
| NRP1 | SEMA3A SEMA3B VEGFA VEGFB PGF |
| NRP2 | SEMA3C SEMA3F VEGFC |
| NRXN1 | NXPH3 NLGN1 NLGN2 NLGN3 NXPH1 |
| NRXN3 | NLGN1 NXPH3 NLGN2 NLGN3 NXPH1 |
| NTRK2 | BDNF NTF3 NTF4 |
| NTRK3 | NTF3 |
| PDE1A | CALM1 CALM3 CALM2 |
| PDE1B | CALM1 |
| PDE1C | CALM1 CALM3 CALM2 |
| PDGFRA | PDGFA PDGFB PDGFC |
| PDGFRB | PDGFB |
| PLAUR | PLAU |
| PLXNA1 | SEMA3A SEMA6D |
| PLXNA2 | SEMA3A |
| PLXNA4 | SEMA3A |
| PLXNB3 | SEMA5A |
| PLXNC1 | SEMA7A |
| PLXND1 | SEMA3E |
| PRLR | CSHL1 CSH1 CSH2 PRL |
| PROCR | PF4 |
| PTH1R | PTH2 PTHLH PTH |
| PTPRB | PTN |
| PTPRF | NID1 |
| PTPRZ1 | PTN |
| RAMP1 | ADM2 CALCA CALCB ADM IAPP |
| RAMP2 | ADM2 CALCA CALCB |
| RAMP3 | ADM2 CALCA CALCB |
| RET | GDNF NRTN ARTN PSPN |
| ROBO1 | NCAM1 SLIT2 |
| ROBO2 | SLIT2 |
| ROBO3 | NCAM1 |
| ROBO4 | SLIT2 |
| RPSA | LAMA1 LAMA2 |
| SDC1 | SLIT2 THBS1 |
| SDC2 | FN1 |
| SELL | CFH CD34 PODXL2 |
| SELP | CD24 |
| SORL1 | APOE |
| TEK | ANGPT1 ANGPT2 |
| TGFBR3 | TGFB2 TGFB1 TGFB3 INHA |
| THBD | PF4 |
| TIE1 | ANGPT1 |
| TNFRSF10A | TNFSF10 |
| TNFRSF10B | TNFSF10 |
| TNFRSF10C | TNFSF10 |
| TNFRSF10D | TNFSF10 |
| TNFRSF11B | TNFSF10 TNFSF11 |
| TNFRSF12A | TNFSF12 |
| TNFRSF25 | TNFSF15 |
| TSHR | GPHA2 |
| TYRO3 | PROS1 GAS6 |
| UNC5A | NTN4 |
| VLDLR | APOE |
