# Supplementary material for: Sctensor detects many-to-many cell–cell interactions from single cell RNA-sequencing data
Source: BMC Bioinformatics. 2023 Nov 7;24:420. doi: 10.1186/s12859-023-05490-y (PMC10631077; doi:10.1186/s12859-023-05490-y)
Supplement: Supplementary file 14 — Additional file 14. HTML report of FetalKidney. [file 12859_2023_5490_MOESM14_ESM.zip › output/report/Human_FetalKidney/pattern_4_2.html]

Details of (4,2,) -related L-R pairs


# Details of (4,2,) -related L-R pairs

# (4,2,:) Pattern

# (4,2,) -related L-R pairs (Top11 pairs)

```
library("scTensor")
load("reanalysis.RData")
col <- .setColor("many")[5]
scTensor:::.myvisNetwork(g, col)
```

| Rank | Ligand Gene | Receptor Gene | Ligand Expression (Log10(exp + 1)) | Receptor Expression (Log10(exp + 1)) | LR-pair factor value (Percentage) | P-value (Grubbs test) | Q-value (BH method) | L-R Evidence cf. rikenbit/lrbase-workflow | PubMed |
| --- | --- | --- | --- | --- | --- | --- | --- | --- | --- |
| 1 | LAMB1 Description: LAMB1laminin subunit beta 1 GO: 1-28 Reactome: 1-19 UniProtKB: 1-3 RefEx: LAMB1 Expression Atlas: LAMB1 Single Cell Expression Atlas: LAMB1 scRNASeqDB: LAMB1 PanglaoDB: LAMB1 CMap: LAMB1 MeSH: 1-100 101-200 201-300 301-400 401-500 501-600 601-700 701-794 | ITGA6 Description: LAMB1integrin subunit alpha 6 GO: 1-29 Reactome: 1-13 UniProtKB: 1 RefEx: LAMB1 Expression Atlas: ITGA6 Single Cell Expression Atlas: ITGA6 scRNASeqDB: ITGA6 PanglaoDB: ITGA6 CMap: ITGA6 MeSH: 1-94 95-188 189-282 283-376 377-470 471-564 565-658 659-752 753-846 847-940 941-1034 1035-1117 |  |  | 0.013 (22.297%) | 1 | 1 | DLRP | 1 |
| 2 | EFNA5 Description: EFNA5ephrin A5 GO: 1-23 Reactome: 1-6 UniProtKB: 1-2 RefEx: EFNA5 Expression Atlas: EFNA5 Single Cell Expression Atlas: EFNA5 scRNASeqDB: EFNA5 PanglaoDB: EFNA5 CMap: EFNA5 MeSH: 1-100 101-200 201-300 301-398 | EPHA7 Description: EFNA5EPH receptor A7 GO: 1-44 Reactome: 1-6 UniProtKB: 1 RefEx: EFNA5 Expression Atlas: EPHA7 Single Cell Expression Atlas: EPHA7 scRNASeqDB: EPHA7 PanglaoDB: EPHA7 CMap: EPHA7 MeSH: 1-99 100-198 199-297 298-394 |  |  | 0.006 (10.022%) | 0.001 | 0.002 | DLRP | 1 |
| 3 | FGF9 Description: FGF9fibroblast growth factor 9 GO: 1-25 Reactome: 1-70 UniProtKB: 1 STRING: 1 RefEx: FGF9 Expression Atlas: FGF9 Single Cell Expression Atlas: FGF9 scRNASeqDB: FGF9 PanglaoDB: FGF9 CMap: FGF9 MeSH: 1-91 92-182 183-273 274-364 365-455 456-546 547-637 638-724 | FGFR3 Description: FGF9fibroblast growth factor receptor 3 GO: 1-41 Reactome: 1-38 UniProtKB: 1-2 RefEx: FGF9 Expression Atlas: FGFR3 Single Cell Expression Atlas: FGFR3 scRNASeqDB: FGFR3 PanglaoDB: FGFR3 CMap: FGFR3 MeSH: 1-98 99-196 197-294 295-392 393-490 491-588 589-686 687-784 785-882 883-980 981-1078 1079-1176 1177-1274 1275-1372 1373-1470 1471-1568 1569-1666 1667-1753 |  |  | 0.005 (8.763%) | 0 | 0.001 | DLRP | 1 |
| 4 | EFNA5 Description: EFNA5ephrin A5 GO: 1-23 Reactome: 1-6 UniProtKB: 1-2 RefEx: EFNA5 Expression Atlas: EFNA5 Single Cell Expression Atlas: EFNA5 scRNASeqDB: EFNA5 PanglaoDB: EFNA5 CMap: EFNA5 MeSH: 1-100 101-200 201-300 301-398 | EPHA4 Description: EFNA5EPH receptor A4 GO: 1-77 Reactome: 1-6 UniProtKB: 1-2 RefEx: EFNA5 Expression Atlas: EPHA4 Single Cell Expression Atlas: EPHA4 scRNASeqDB: EPHA4 PanglaoDB: EPHA4 CMap: EPHA4 MeSH: 1-93 94-186 187-279 280-372 373-465 466-557 |  |  | 0.005 (8.173%) | 0.102 | 0.118 | DLRP | 1 |
| 5 | COL4A1 Description: COL4A1collagen type IV alpha 1 chain GO: 1-22 Reactome: 1-24 UniProtKB: 1-3 RefEx: COL4A1 Expression Atlas: COL4A1 Single Cell Expression Atlas: COL4A1 scRNASeqDB: COL4A1 PanglaoDB: COL4A1 CMap: COL4A1 MeSH: 1-94 95-188 189-282 283-376 377-470 471-564 565-658 659-752 753-846 847-940 941-1032 | ITGB8 Description: COL4A1integrin subunit beta 8 GO: 1-26 Reactome: 1-4 UniProtKB: 1-2 RefEx: COL4A1 Expression Atlas: ITGB8 Single Cell Expression Atlas: ITGB8 scRNASeqDB: ITGB8 PanglaoDB: ITGB8 CMap: ITGB8 MeSH: 1-100 101-200 201-300 301-400 |  |  | 0.005 (7.73%) | 0.036 | 0.052 | DLRP | 1 |
| 6 | CXCL12 Description: CXCL12C-X-C motif chemokine ligand 12 GO: 1-53 Reactome: 1-18 UniProtKB: 1 RefEx: CXCL12 Expression Atlas: CXCL12 Single Cell Expression Atlas: CXCL12 scRNASeqDB: CXCL12 PanglaoDB: CXCL12 CMap: CXCL12 MeSH: 1-100 101-200 201-300 301-400 401-500 501-600 601-700 701-800 801-900 901-1000 1001-1100 1101-1200 1201-1300 1301-1400 1401-1500 1501-1600 1601-1700 1701-1800 1801-1900 1901-2000 2001-2100 2101-2200 2201-2300 2301-2400 2401-2500 2501-2600 2601-2700 2701-2800 2801-2900 2901-3000 3001-3100 3101-3200 3201-3275 | CXCR4 Description: CXCL12C-X-C motif chemokine receptor 4 GO: 1-67 Reactome: 1-18 UniProtKB: 1-3 STRING: 1 2 RefEx: CXCL12 Expression Atlas: CXCR4 Single Cell Expression Atlas: CXCR4 scRNASeqDB: CXCR4 PanglaoDB: CXCR4 CMap: CXCR4 MeSH: 1-100 101-200 201-300 301-400 401-500 501-600 601-700 701-800 801-900 901-1000 1001-1100 1101-1200 1201-1300 1301-1400 1401-1500 1501-1600 1601-1700 1701-1800 1801-1900 1901-2000 2001-2100 2101-2200 2201-2300 2301-2400 2401-2500 2501-2600 2601-2700 2701-2780 |  |  | 0.004 (6.827%) | 0.026 | 0.04 | DLRP | 1 |
| 7 | LAMA1 Description: LAMA1laminin subunit alpha 1 GO: 1-30 Reactome: 1-13 UniProtKB: 1 RefEx: LAMA1 Expression Atlas: LAMA1 Single Cell Expression Atlas: LAMA1 scRNASeqDB: LAMA1 PanglaoDB: LAMA1 CMap: LAMA1 MeSH: 1-97 98-194 195-291 292-388 389-485 486-582 583-679 680-776 777-873 874-961 | ITGB8 Description: LAMA1integrin subunit beta 8 GO: 1-26 Reactome: 1-4 UniProtKB: 1-2 RefEx: LAMA1 Expression Atlas: ITGB8 Single Cell Expression Atlas: ITGB8 scRNASeqDB: ITGB8 PanglaoDB: ITGB8 CMap: ITGB8 MeSH: 1-100 101-200 201-300 301-400 |  |  | 0.003 (5.709%) | 0.093 | 0.113 | DLRP | 1 |
| 8 | EFNA1 Description: EFNA1ephrin A1 GO: 1-34 Reactome: 1-6 UniProtKB: 1 RefEx: EFNA1 Expression Atlas: EFNA1 Single Cell Expression Atlas: EFNA1 scRNASeqDB: EFNA1 PanglaoDB: EFNA1 CMap: EFNA1 MeSH: 1-94 95-188 189-282 283-376 377-466 | EPHA7 Description: EFNA1EPH receptor A7 GO: 1-44 Reactome: 1-6 UniProtKB: 1 RefEx: EFNA1 Expression Atlas: EPHA7 Single Cell Expression Atlas: EPHA7 scRNASeqDB: EPHA7 PanglaoDB: EPHA7 CMap: EPHA7 MeSH: 1-99 100-198 199-297 298-394 |  |  | 0.003 (5.224%) | 0 | 0 | DLRP | 1 |
| 9 | EFNA1 Description: EFNA1ephrin A1 GO: 1-34 Reactome: 1-6 UniProtKB: 1 RefEx: EFNA1 Expression Atlas: EFNA1 Single Cell Expression Atlas: EFNA1 scRNASeqDB: EFNA1 PanglaoDB: EFNA1 CMap: EFNA1 MeSH: 1-94 95-188 189-282 283-376 377-466 | EPHA4 Description: EFNA1EPH receptor A4 GO: 1-77 Reactome: 1-6 UniProtKB: 1-2 RefEx: EFNA1 Expression Atlas: EPHA4 Single Cell Expression Atlas: EPHA4 scRNASeqDB: EPHA4 PanglaoDB: EPHA4 CMap: EPHA4 MeSH: 1-93 94-186 187-279 280-372 373-465 466-557 |  |  | 0.002 (4.261%) | 1 | 1 | DLRP | 1 |
| 10 | BMP7 Description: BMP7bone morphogenetic protein 7 GO: 1-86 Reactome: 1-3 UniProtKB: 1-2 RefEx: BMP7 Expression Atlas: BMP7 Single Cell Expression Atlas: BMP7 scRNASeqDB: BMP7 PanglaoDB: BMP7 CMap: BMP7 MeSH: 1-95 96-190 191-285 286-380 381-475 476-570 571-665 666-760 761-855 856-950 951-1045 1046-1140 1141-1235 1236-1330 1331-1425 1426-1520 1521-1615 1616-1710 1711-1801 | ACVR2B Description: BMP7activin A receptor type 2B GO: 1-33 Reactome: 1-7 UniProtKB: 1 RefEx: BMP7 Expression Atlas: ACVR2B Single Cell Expression Atlas: ACVR2B scRNASeqDB: ACVR2B PanglaoDB: ACVR2B CMap: ACVR2B MeSH: 1-86 87-172 173-258 259-344 345-430 431-515 |  |  | 0.002 (4.062%) | 1 | 1 | DLRP | 1 |
| 11 | COL4A1 Description: COL4A1collagen type IV alpha 1 chain GO: 1-22 Reactome: 1-24 UniProtKB: 1-3 RefEx: COL4A1 Expression Atlas: COL4A1 Single Cell Expression Atlas: COL4A1 scRNASeqDB: COL4A1 PanglaoDB: COL4A1 CMap: COL4A1 MeSH: 1-94 95-188 189-282 283-376 377-470 471-564 565-658 659-752 753-846 847-940 941-1032 | ITGA1 Description: COL4A1integrin subunit alpha 1 GO: 1-29 Reactome: 1-14 UniProtKB: 1 RefEx: COL4A1 Expression Atlas: ITGA1 Single Cell Expression Atlas: ITGA1 scRNASeqDB: ITGA1 PanglaoDB: ITGA1 CMap: ITGA1 MeSH: 1-91 92-182 183-273 274-364 365-455 456-546 547-637 638-728 729-817 |  |  | 0.001 (2.41%) | 0.001 | 0.003 | DLRP | 1 |

# (4,2,) -related Enrichment Analysis

```
load("reanalysis.RData")
library("plotly")
```

## MeSH-Enrichment Analysis (A : Anatomy)

```
negLogPval <- -log10(out.vecLR[['pattern_4_2']]$Enrich$MeSH_A$Pvalue + 1E-10)
term <- out.vecLR[['pattern_4_2']]$Enrich$MeSH_A$Term
target <- seq_len(min(100, length(negLogPval)))
negLogPval <- negLogPval[target]
term <- term[target]
p <- plot_ly(x=seq_along(negLogPval), y=~negLogPval,
type="bar", color=~negLogPval, text=term,
colors=c("#4b61ba", "gray", "#a87963", "red"))
layout(p, title="MeSH-Enrichment Analysis (A)",
   xaxis=list(title="Term"),
   yaxis=list(title="-Log(P-value)"),
   legend=list(name=""))
```

```
## Warning: textfont.color doesn't (yet) support data arrays

## Warning: textfont.color doesn't (yet) support data arrays
```

## MeSH-Enrichment Analysis (B : Organisms)

```
negLogPval <- -log10(out.vecLR[['pattern_4_2']]$Enrich$MeSH_B$Pvalue + 1E-10)
term <- out.vecLR[['pattern_4_2']]$Enrich$MeSH_B$Term
target <- seq_len(min(100, length(negLogPval)))
negLogPval <- negLogPval[target]
term <- term[target]
p <- plot_ly(x=seq_along(negLogPval), y=~negLogPval,
type="bar", color=~negLogPval, text=term,
colors=c("#4b61ba", "gray", "#a87963", "red"))
layout(p, title="MeSH-Enrichment Analysis (B)",
   xaxis=list(title="Term"),
   yaxis=list(title="-Log(P-value)"),
   legend=list(name=""))
```

```
## Warning: textfont.color doesn't (yet) support data arrays

## Warning: textfont.color doesn't (yet) support data arrays
```

## MeSH-Enrichment Analysis (C : Diseases)

```
negLogPval <- -log10(out.vecLR[['pattern_4_2']]$Enrich$MeSH_C$Pvalue + 1E-10)
term <- out.vecLR[['pattern_4_2']]$Enrich$MeSH_C$Term
target <- seq_len(min(100, length(negLogPval)))
negLogPval <- negLogPval[target]
term <- term[target]
p <- plot_ly(x=seq_along(negLogPval), y=~negLogPval,
type="bar", color=~negLogPval, text=term,
colors=c("#4b61ba", "gray", "#a87963", "red"))
layout(p, title="MeSH-Enrichment Analysis (C)",
   xaxis=list(title="Term"),
   yaxis=list(title="-Log(P-value)"),
   legend=list(name=""))
```

```
## Warning: textfont.color doesn't (yet) support data arrays

## Warning: textfont.color doesn't (yet) support data arrays
```

## MeSH-Enrichment Analysis (D : Drugs)

```
negLogPval <- -log10(out.vecLR[['pattern_4_2']]$Enrich$MeSH_D$Pvalue + 1E-10)
term <- out.vecLR[['pattern_4_2']]$Enrich$MeSH_D$Term
target <- seq_len(min(100, length(negLogPval)))
negLogPval <- negLogPval[target]
term <- term[target]
p <- plot_ly(x=seq_along(negLogPval), y=~negLogPval,
type="bar", color=~negLogPval, text=term,
colors=c("#4b61ba", "gray", "#a87963", "red"))
layout(p, title="MeSH-Enrichment Analysis (D)",
   xaxis=list(title="Term"),
   yaxis=list(title="-Log(P-value)"),
   legend=list(name=""))
```

```
## Warning: textfont.color doesn't (yet) support data arrays

## Warning: textfont.color doesn't (yet) support data arrays
```

## MeSH-Enrichment Analysis (E : Analytical, Diagnostic and Therapeutic Techniques and Equipment)

```
negLogPval <- -log10(out.vecLR[['pattern_4_2']]$Enrich$MeSH_E$Pvalue + 1E-10)
term <- out.vecLR[['pattern_4_2']]$Enrich$MeSH_E$Term
target <- seq_len(min(100, length(negLogPval)))
negLogPval <- negLogPval[target]
term <- term[target]
p <- plot_ly(x=seq_along(negLogPval), y=~negLogPval,
type="bar", color=~negLogPval, text=term,
colors=c("#4b61ba", "gray", "#a87963", "red"))
layout(p, title="MeSH-Enrichment Analysis (E)",
   xaxis=list(title="Term"),
   yaxis=list(title="-Log(P-value)"),
   legend=list(name=""))
```

```
## Warning: textfont.color doesn't (yet) support data arrays

## Warning: textfont.color doesn't (yet) support data arrays
```

## MeSH-Enrichment Analysis (G : Phenomena and Processes)

```
negLogPval <- -log10(out.vecLR[['pattern_4_2']]$Enrich$MeSH_G$Pvalue + 1E-10)
term <- out.vecLR[['pattern_4_2']]$Enrich$MeSH_G$Term
target <- seq_len(min(100, length(negLogPval)))
negLogPval <- negLogPval[target]
term <- term[target]
p <- plot_ly(x=seq_along(negLogPval), y=~negLogPval,
type="bar", color=~negLogPval, text=term,
colors=c("#4b61ba", "gray", "#a87963", "red"))
layout(p, title="MeSH-Enrichment Analysis (G)",
   xaxis=list(title="Term"),
   yaxis=list(title="-Log(P-value)"),
   legend=list(name=""))
```

```
## Warning: textfont.color doesn't (yet) support data arrays

## Warning: textfont.color doesn't (yet) support data arrays
```

## MeSH-Enrichment Analysis (J : Technology and Food and Beverages)

```
negLogPval <- -log10(out.vecLR[['pattern_4_2']]$Enrich$MeSH_J$Pvalue + 1E-10)
term <- out.vecLR[['pattern_4_2']]$Enrich$MeSH_J$Term
target <- seq_len(min(100, length(negLogPval)))
negLogPval <- negLogPval[target]
term <- term[target]
p <- plot_ly(x=seq_along(negLogPval), y=~negLogPval,
type="bar", color=~negLogPval, text=term,
colors=c("#4b61ba", "gray", "#a87963", "red"))
layout(p, title="MeSH-Enrichment Analysis (J)",
   xaxis=list(title="Term"),
   yaxis=list(title="-Log(P-value)"),
   legend=list(name=""))
```

```
## Warning: textfont.color doesn't (yet) support data arrays

## Warning: textfont.color doesn't (yet) support data arrays
```

## MeSH-Enrichment Analysis (L : Information Science)

```
negLogPval <- -log10(out.vecLR[['pattern_4_2']]$Enrich$MeSH_L$Pvalue + 1E-10)
term <- out.vecLR[['pattern_4_2']]$Enrich$MeSH_L$Term
target <- seq_len(min(100, length(negLogPval)))
negLogPval <- negLogPval[target]
term <- term[target]
p <- plot_ly(x=seq_along(negLogPval), y=~negLogPval,
type="bar", color=~negLogPval, text=term,
colors=c("#4b61ba", "gray", "#a87963", "red"))
layout(p, title="MeSH-Enrichment Analysis (L)",
   xaxis=list(title="Term"),
   yaxis=list(title="-Log(P-value)"),
   legend=list(name=""))
```

```
## Warning: textfont.color doesn't (yet) support data arrays

## Warning: textfont.color doesn't (yet) support data arrays
```

## MeSH-Enrichment Analysis (N : Health Care)

```
negLogPval <- -log10(out.vecLR[['pattern_4_2']]$Enrich$MeSH_N$Pvalue + 1E-10)
term <- out.vecLR[['pattern_4_2']]$Enrich$MeSH_N$Term
target <- seq_len(min(100, length(negLogPval)))
negLogPval <- negLogPval[target]
term <- term[target]
p <- plot_ly(x=seq_along(negLogPval), y=~negLogPval,
type="bar", color=~negLogPval, text=term,
colors=c("#4b61ba", "gray", "#a87963", "red"))
layout(p, title="MeSH-Enrichment Analysis (N)",
   xaxis=list(title="Term"),
   yaxis=list(title="-Log(P-value)"),
   legend=list(name=""))
```

```
## Warning: textfont.color doesn't (yet) support data arrays

## Warning: textfont.color doesn't (yet) support data arrays
```

## MeSH-Enrichment Analysis (Z : Geographical Locations)

```
negLogPval <- -log10(out.vecLR[['pattern_4_2']]$Enrich$MeSH_Z$Pvalue + 1E-10)
term <- out.vecLR[['pattern_4_2']]$Enrich$MeSH_Z$Term
target <- seq_len(min(100, length(negLogPval)))
negLogPval <- negLogPval[target]
term <- term[target]
p <- plot_ly(x=seq_along(negLogPval), y=~negLogPval,
type="bar", color=~negLogPval, text=term,
colors=c("#4b61ba", "gray", "#a87963", "red"))
layout(p, title="MeSH-Enrichment Analysis (Z)",
   xaxis=list(title="Term"),
   yaxis=list(title="-Log(P-value)"),
   legend=list(name=""))
```

## Reactome Pathway-Enrichment Analysis

```
negLogPval <- -log10(out.vecLR[['pattern_4_2']]$Enrich$Reactome$Pvalue + 1E-10)
term <- out.vecLR[['pattern_4_2']]$Enrich$Reactome$Term
target <- seq_len(min(100, length(negLogPval)))
negLogPval <- negLogPval[target]
term <- term[target]
p <- plot_ly(x=seq_along(negLogPval), y=~negLogPval,
type="bar", color=~negLogPval, text=term,
colors=c("#4b61ba", "gray", "#a87963", "red"))
layout(p, title="Reactome-Enrichment Analysis",
   xaxis=list(title="Term"),
   yaxis=list(title="-Log(P-value)"),
   legend=list(name=""))
```

```
## Warning: textfont.color doesn't (yet) support data arrays

## Warning: textfont.color doesn't (yet) support data arrays
```

## DO-Enrichment Analysis

```
negLogPval <- -log10(out.vecLR[['pattern_4_2']]$Enrich$DO$Pvalue + 1E-10)
term <- out.vecLR[['pattern_4_2']]$Enrich$DO$Term
target <- seq_len(min(100, length(negLogPval)))
negLogPval <- negLogPval[target]
term <- term[target]
p <- plot_ly(x=seq_along(negLogPval), y=~negLogPval,
type="bar", color=~negLogPval, text=term,
colors=c("#4b61ba", "gray", "#a87963", "red"))
layout(p, title="DO-Enrichment Analysis",
   xaxis=list(title="Term"),
   yaxis=list(title="-Log(P-value)"),
   legend=list(name=""))
```

```
## Warning: textfont.color doesn't (yet) support data arrays

## Warning: textfont.color doesn't (yet) support data arrays
```

## NCG-Enrichment Analysis

```
negLogPval <- -log10(out.vecLR[['pattern_4_2']]$Enrich$NCG$Pvalue + 1E-10)
term <- out.vecLR[['pattern_4_2']]$Enrich$NCG$Term
target <- seq_len(min(100, length(negLogPval)))
negLogPval <- negLogPval[target]
term <- term[target]
p <- plot_ly(x=seq_along(negLogPval), y=~negLogPval,
type="bar", color=~negLogPval, text=term,
colors=c("#4b61ba", "gray", "#a87963", "red"))
layout(p, title="NCG-Enrichment Analysis",
   xaxis=list(title="Term"),
   yaxis=list(title="-Log(P-value)"),
   legend=list(name=""))
```

```
## Warning: textfont.color doesn't (yet) support data arrays

## Warning: textfont.color doesn't (yet) support data arrays
```

## DGN-Enrichment Analysis

```
negLogPval <- -log10(out.vecLR[['pattern_4_2']]$Enrich$DGN$Pvalue + 1E-10)
term <- out.vecLR[['pattern_4_2']]$Enrich$DGN$Term
target <- seq_len(min(100, length(negLogPval)))
negLogPval <- negLogPval[target]
term <- term[target]
p <- plot_ly(x=seq_along(negLogPval), y=~negLogPval,
type="bar", color=~negLogPval, text=term,
colors=c("#4b61ba", "gray", "#a87963", "red"))
layout(p, title="DGN-Enrichment Analysis",
   xaxis=list(title="Term"),
   yaxis=list(title="-Log(P-value)"),
   legend=list(name=""))
```

```
## Warning: textfont.color doesn't (yet) support data arrays

## Warning: textfont.color doesn't (yet) support data arrays
```
