# Supplementary material for: Sctensor detects many-to-many cell–cell interactions from single cell RNA-sequencing data
Source: BMC Bioinformatics. 2023 Nov 7;24:420. doi: 10.1186/s12859-023-05490-y (PMC10631077; doi:10.1186/s12859-023-05490-y)
Supplement: Supplementary file 14 — Additional file 14. HTML report of FetalKidney. [file 12859_2023_5490_MOESM14_ESM.zip › output/report/Human_FetalKidney/pattern_4_1.html]

Details of (4,1,) -related L-R pairs


# Details of (4,1,) -related L-R pairs

# (4,1,:) Pattern

# (4,1,) -related L-R pairs (Top4 pairs)

```
library("scTensor")
load("reanalysis.RData")
col <- .setColor("many")[12]
scTensor:::.myvisNetwork(g, col)
```

| Rank | Ligand Gene | Receptor Gene | Ligand Expression (Log10(exp + 1)) | Receptor Expression (Log10(exp + 1)) | LR-pair factor value (Percentage) | P-value (Grubbs test) | Q-value (BH method) | L-R Evidence cf. rikenbit/lrbase-workflow | PubMed |
| --- | --- | --- | --- | --- | --- | --- | --- | --- | --- |
| 1 | LAMB1 Description: LAMB1laminin subunit beta 1 GO: 1-28 Reactome: 1-19 UniProtKB: 1-3 RefEx: LAMB1 Expression Atlas: LAMB1 Single Cell Expression Atlas: LAMB1 scRNASeqDB: LAMB1 PanglaoDB: LAMB1 CMap: LAMB1 MeSH: 1-100 101-200 201-300 301-400 401-500 501-600 601-700 701-794 | ITGA6 Description: LAMB1integrin subunit alpha 6 GO: 1-29 Reactome: 1-13 UniProtKB: 1 RefEx: LAMB1 Expression Atlas: ITGA6 Single Cell Expression Atlas: ITGA6 scRNASeqDB: ITGA6 PanglaoDB: ITGA6 CMap: ITGA6 MeSH: 1-94 95-188 189-282 283-376 377-470 471-564 565-658 659-752 753-846 847-940 941-1034 1035-1117 |  |  | 0.019 (40.01%) | 0.036 | 0.05 | DLRP | 1 |
| 2 | COL4A1 Description: COL4A1collagen type IV alpha 1 chain GO: 1-22 Reactome: 1-24 UniProtKB: 1-3 RefEx: COL4A1 Expression Atlas: COL4A1 Single Cell Expression Atlas: COL4A1 scRNASeqDB: COL4A1 PanglaoDB: COL4A1 CMap: COL4A1 MeSH: 1-94 95-188 189-282 283-376 377-470 471-564 565-658 659-752 753-846 847-940 941-1032 | ITGB8 Description: COL4A1integrin subunit beta 8 GO: 1-26 Reactome: 1-4 UniProtKB: 1-2 RefEx: COL4A1 Expression Atlas: ITGB8 Single Cell Expression Atlas: ITGB8 scRNASeqDB: ITGB8 PanglaoDB: ITGB8 CMap: ITGB8 MeSH: 1-100 101-200 201-300 301-400 |  |  | 0.007 (14.89%) | 0.005 | 0.013 | DLRP | 1 |
| 3 | CALM1 Description: CALM1calmodulin 1 GO: 1-75 Reactome: 1-68 69-136 UniProtKB: 1-4 RefEx: CALM1 Expression Atlas: CALM1 Single Cell Expression Atlas: CALM1 scRNASeqDB: CALM1 PanglaoDB: CALM1 CMap: CALM1 MeSH: 1-100 101-200 201-300 301-400 401-500 501-600 601-700 701-800 801-900 901-1000 1001-1100 1101-1200 1201-1300 1301-1400 1401-1495 | MYLK Description: CALM1myosin light chain kinase GO: 1-23 Reactome: 1-7 UniProtKB: 1-3 RefEx: CALM1 Expression Atlas: MYLK Single Cell Expression Atlas: MYLK scRNASeqDB: MYLK PanglaoDB: MYLK CMap: MYLK MeSH: 1-97 98-194 195-291 292-388 389-485 486-582 583-679 680-775 |  |  | 0.006 (12.893%) | 0.035 | 0.05 | DLRP | 1 |
| 4 | LAMA1 Description: LAMA1laminin subunit alpha 1 GO: 1-30 Reactome: 1-13 UniProtKB: 1 RefEx: LAMA1 Expression Atlas: LAMA1 Single Cell Expression Atlas: LAMA1 scRNASeqDB: LAMA1 PanglaoDB: LAMA1 CMap: LAMA1 MeSH: 1-97 98-194 195-291 292-388 389-485 486-582 583-679 680-776 777-873 874-961 | ITGB8 Description: LAMA1integrin subunit beta 8 GO: 1-26 Reactome: 1-4 UniProtKB: 1-2 RefEx: LAMA1 Expression Atlas: ITGB8 Single Cell Expression Atlas: ITGB8 scRNASeqDB: ITGB8 PanglaoDB: ITGB8 CMap: ITGB8 MeSH: 1-100 101-200 201-300 301-400 |  |  | 0.005 (10.996%) | 0.002 | 0.007 | DLRP | 1 |

# (4,1,) -related Enrichment Analysis

```
load("reanalysis.RData")
library("plotly")
```

## MeSH-Enrichment Analysis (A : Anatomy)

```
negLogPval <- -log10(out.vecLR[['pattern_4_1']]$Enrich$MeSH_A$Pvalue + 1E-10)
term <- out.vecLR[['pattern_4_1']]$Enrich$MeSH_A$Term
target <- seq_len(min(100, length(negLogPval)))
negLogPval <- negLogPval[target]
term <- term[target]
p <- plot_ly(x=seq_along(negLogPval), y=~negLogPval,
type="bar", color=~negLogPval, text=term,
colors=c("#4b61ba", "gray", "#a87963", "red"))
layout(p, title="MeSH-Enrichment Analysis (A)",
   xaxis=list(title="Term"),
   yaxis=list(title="-Log(P-value)"),
   legend=list(name=""))
```

```
## Warning: textfont.color doesn't (yet) support data arrays

## Warning: textfont.color doesn't (yet) support data arrays
```

## MeSH-Enrichment Analysis (B : Organisms)

```
negLogPval <- -log10(out.vecLR[['pattern_4_1']]$Enrich$MeSH_B$Pvalue + 1E-10)
term <- out.vecLR[['pattern_4_1']]$Enrich$MeSH_B$Term
target <- seq_len(min(100, length(negLogPval)))
negLogPval <- negLogPval[target]
term <- term[target]
p <- plot_ly(x=seq_along(negLogPval), y=~negLogPval,
type="bar", color=~negLogPval, text=term,
colors=c("#4b61ba", "gray", "#a87963", "red"))
layout(p, title="MeSH-Enrichment Analysis (B)",
   xaxis=list(title="Term"),
   yaxis=list(title="-Log(P-value)"),
   legend=list(name=""))
```

```
## Warning: textfont.color doesn't (yet) support data arrays

## Warning: textfont.color doesn't (yet) support data arrays
```

## MeSH-Enrichment Analysis (C : Diseases)

```
negLogPval <- -log10(out.vecLR[['pattern_4_1']]$Enrich$MeSH_C$Pvalue + 1E-10)
term <- out.vecLR[['pattern_4_1']]$Enrich$MeSH_C$Term
target <- seq_len(min(100, length(negLogPval)))
negLogPval <- negLogPval[target]
term <- term[target]
p <- plot_ly(x=seq_along(negLogPval), y=~negLogPval,
type="bar", color=~negLogPval, text=term,
colors=c("#4b61ba", "gray", "#a87963", "red"))
layout(p, title="MeSH-Enrichment Analysis (C)",
   xaxis=list(title="Term"),
   yaxis=list(title="-Log(P-value)"),
   legend=list(name=""))
```

```
## Warning: textfont.color doesn't (yet) support data arrays

## Warning: textfont.color doesn't (yet) support data arrays
```

## MeSH-Enrichment Analysis (D : Drugs)

```
negLogPval <- -log10(out.vecLR[['pattern_4_1']]$Enrich$MeSH_D$Pvalue + 1E-10)
term <- out.vecLR[['pattern_4_1']]$Enrich$MeSH_D$Term
target <- seq_len(min(100, length(negLogPval)))
negLogPval <- negLogPval[target]
term <- term[target]
p <- plot_ly(x=seq_along(negLogPval), y=~negLogPval,
type="bar", color=~negLogPval, text=term,
colors=c("#4b61ba", "gray", "#a87963", "red"))
layout(p, title="MeSH-Enrichment Analysis (D)",
   xaxis=list(title="Term"),
   yaxis=list(title="-Log(P-value)"),
   legend=list(name=""))
```

```
## Warning: textfont.color doesn't (yet) support data arrays

## Warning: textfont.color doesn't (yet) support data arrays
```

## MeSH-Enrichment Analysis (E : Analytical, Diagnostic and Therapeutic Techniques and Equipment)

```
negLogPval <- -log10(out.vecLR[['pattern_4_1']]$Enrich$MeSH_E$Pvalue + 1E-10)
term <- out.vecLR[['pattern_4_1']]$Enrich$MeSH_E$Term
target <- seq_len(min(100, length(negLogPval)))
negLogPval <- negLogPval[target]
term <- term[target]
p <- plot_ly(x=seq_along(negLogPval), y=~negLogPval,
type="bar", color=~negLogPval, text=term,
colors=c("#4b61ba", "gray", "#a87963", "red"))
layout(p, title="MeSH-Enrichment Analysis (E)",
   xaxis=list(title="Term"),
   yaxis=list(title="-Log(P-value)"),
   legend=list(name=""))
```

```
## Warning: textfont.color doesn't (yet) support data arrays

## Warning: textfont.color doesn't (yet) support data arrays
```

## MeSH-Enrichment Analysis (F : Psychiatry and Psychology)

```
negLogPval <- -log10(out.vecLR[['pattern_4_1']]$Enrich$MeSH_F$Pvalue + 1E-10)
term <- out.vecLR[['pattern_4_1']]$Enrich$MeSH_F$Term
target <- seq_len(min(100, length(negLogPval)))
negLogPval <- negLogPval[target]
term <- term[target]
p <- plot_ly(x=seq_along(negLogPval), y=~negLogPval,
type="bar", color=~negLogPval, text=term,
colors=c("#4b61ba", "gray", "#a87963", "red"))
layout(p, title="MeSH-Enrichment Analysis (F)",
   xaxis=list(title="Term"),
   yaxis=list(title="-Log(P-value)"),
   legend=list(name=""))
```

## MeSH-Enrichment Analysis (G : Phenomena and Processes)

```
negLogPval <- -log10(out.vecLR[['pattern_4_1']]$Enrich$MeSH_G$Pvalue + 1E-10)
term <- out.vecLR[['pattern_4_1']]$Enrich$MeSH_G$Term
target <- seq_len(min(100, length(negLogPval)))
negLogPval <- negLogPval[target]
term <- term[target]
p <- plot_ly(x=seq_along(negLogPval), y=~negLogPval,
type="bar", color=~negLogPval, text=term,
colors=c("#4b61ba", "gray", "#a87963", "red"))
layout(p, title="MeSH-Enrichment Analysis (G)",
   xaxis=list(title="Term"),
   yaxis=list(title="-Log(P-value)"),
   legend=list(name=""))
```

```
## Warning: textfont.color doesn't (yet) support data arrays

## Warning: textfont.color doesn't (yet) support data arrays
```

## MeSH-Enrichment Analysis (J : Technology and Food and Beverages)

```
negLogPval <- -log10(out.vecLR[['pattern_4_1']]$Enrich$MeSH_J$Pvalue + 1E-10)
term <- out.vecLR[['pattern_4_1']]$Enrich$MeSH_J$Term
target <- seq_len(min(100, length(negLogPval)))
negLogPval <- negLogPval[target]
term <- term[target]
p <- plot_ly(x=seq_along(negLogPval), y=~negLogPval,
type="bar", color=~negLogPval, text=term,
colors=c("#4b61ba", "gray", "#a87963", "red"))
layout(p, title="MeSH-Enrichment Analysis (J)",
   xaxis=list(title="Term"),
   yaxis=list(title="-Log(P-value)"),
   legend=list(name=""))
```

## MeSH-Enrichment Analysis (L : Information Science)

```
negLogPval <- -log10(out.vecLR[['pattern_4_1']]$Enrich$MeSH_L$Pvalue + 1E-10)
term <- out.vecLR[['pattern_4_1']]$Enrich$MeSH_L$Term
target <- seq_len(min(100, length(negLogPval)))
negLogPval <- negLogPval[target]
term <- term[target]
p <- plot_ly(x=seq_along(negLogPval), y=~negLogPval,
type="bar", color=~negLogPval, text=term,
colors=c("#4b61ba", "gray", "#a87963", "red"))
layout(p, title="MeSH-Enrichment Analysis (L)",
   xaxis=list(title="Term"),
   yaxis=list(title="-Log(P-value)"),
   legend=list(name=""))
```

## MeSH-Enrichment Analysis (M : Persons)

```
negLogPval <- -log10(out.vecLR[['pattern_4_1']]$Enrich$MeSH_M$Pvalue + 1E-10)
term <- out.vecLR[['pattern_4_1']]$Enrich$MeSH_M$Term
target <- seq_len(min(100, length(negLogPval)))
negLogPval <- negLogPval[target]
term <- term[target]
p <- plot_ly(x=seq_along(negLogPval), y=~negLogPval,
type="bar", color=~negLogPval, text=term,
colors=c("#4b61ba", "gray", "#a87963", "red"))
layout(p, title="MeSH-Enrichment Analysis (M)",
   xaxis=list(title="Term"),
   yaxis=list(title="-Log(P-value)"),
   legend=list(name=""))
```

## MeSH-Enrichment Analysis (N : Health Care)

```
negLogPval <- -log10(out.vecLR[['pattern_4_1']]$Enrich$MeSH_N$Pvalue + 1E-10)
term <- out.vecLR[['pattern_4_1']]$Enrich$MeSH_N$Term
target <- seq_len(min(100, length(negLogPval)))
negLogPval <- negLogPval[target]
term <- term[target]
p <- plot_ly(x=seq_along(negLogPval), y=~negLogPval,
type="bar", color=~negLogPval, text=term,
colors=c("#4b61ba", "gray", "#a87963", "red"))
layout(p, title="MeSH-Enrichment Analysis (N)",
   xaxis=list(title="Term"),
   yaxis=list(title="-Log(P-value)"),
   legend=list(name=""))
```

## Reactome Pathway-Enrichment Analysis

```
negLogPval <- -log10(out.vecLR[['pattern_4_1']]$Enrich$Reactome$Pvalue + 1E-10)
term <- out.vecLR[['pattern_4_1']]$Enrich$Reactome$Term
target <- seq_len(min(100, length(negLogPval)))
negLogPval <- negLogPval[target]
term <- term[target]
p <- plot_ly(x=seq_along(negLogPval), y=~negLogPval,
type="bar", color=~negLogPval, text=term,
colors=c("#4b61ba", "gray", "#a87963", "red"))
layout(p, title="Reactome-Enrichment Analysis",
   xaxis=list(title="Term"),
   yaxis=list(title="-Log(P-value)"),
   legend=list(name=""))
```

```
## Warning: textfont.color doesn't (yet) support data arrays

## Warning: textfont.color doesn't (yet) support data arrays
```

## DO-Enrichment Analysis

```
negLogPval <- -log10(out.vecLR[['pattern_4_1']]$Enrich$DO$Pvalue + 1E-10)
term <- out.vecLR[['pattern_4_1']]$Enrich$DO$Term
target <- seq_len(min(100, length(negLogPval)))
negLogPval <- negLogPval[target]
term <- term[target]
p <- plot_ly(x=seq_along(negLogPval), y=~negLogPval,
type="bar", color=~negLogPval, text=term,
colors=c("#4b61ba", "gray", "#a87963", "red"))
layout(p, title="DO-Enrichment Analysis",
   xaxis=list(title="Term"),
   yaxis=list(title="-Log(P-value)"),
   legend=list(name=""))
```

```
## Warning: textfont.color doesn't (yet) support data arrays

## Warning: textfont.color doesn't (yet) support data arrays
```

## NCG-Enrichment Analysis

```
negLogPval <- -log10(out.vecLR[['pattern_4_1']]$Enrich$NCG$Pvalue + 1E-10)
term <- out.vecLR[['pattern_4_1']]$Enrich$NCG$Term
target <- seq_len(min(100, length(negLogPval)))
negLogPval <- negLogPval[target]
term <- term[target]
p <- plot_ly(x=seq_along(negLogPval), y=~negLogPval,
type="bar", color=~negLogPval, text=term,
colors=c("#4b61ba", "gray", "#a87963", "red"))
layout(p, title="NCG-Enrichment Analysis",
   xaxis=list(title="Term"),
   yaxis=list(title="-Log(P-value)"),
   legend=list(name=""))
```

## DGN-Enrichment Analysis

```
negLogPval <- -log10(out.vecLR[['pattern_4_1']]$Enrich$DGN$Pvalue + 1E-10)
term <- out.vecLR[['pattern_4_1']]$Enrich$DGN$Term
target <- seq_len(min(100, length(negLogPval)))
negLogPval <- negLogPval[target]
term <- term[target]
p <- plot_ly(x=seq_along(negLogPval), y=~negLogPval,
type="bar", color=~negLogPval, text=term,
colors=c("#4b61ba", "gray", "#a87963", "red"))
layout(p, title="DGN-Enrichment Analysis",
   xaxis=list(title="Term"),
   yaxis=list(title="-Log(P-value)"),
   legend=list(name=""))
```

```
## Warning: textfont.color doesn't (yet) support data arrays

## Warning: textfont.color doesn't (yet) support data arrays
```
