# Supplementary material for: Sctensor detects many-to-many cell–cell interactions from single cell RNA-sequencing data
Source: BMC Bioinformatics. 2023 Nov 7;24:420. doi: 10.1186/s12859-023-05490-y (PMC10631077; doi:10.1186/s12859-023-05490-y)
Supplement: Supplementary file 14 — Additional file 14. HTML report of FetalKidney. [file 12859_2023_5490_MOESM14_ESM.zip › output/report/Human_FetalKidney/pattern_3_4.html]

Details of (3,4,) -related L-R pairs


# Details of (3,4,) -related L-R pairs

# (3,4,:) Pattern

# (3,4,) -related L-R pairs (Top1 pairs)

```
library("scTensor")
load("reanalysis.RData")
col <- .setColor("many")[13]
scTensor:::.myvisNetwork(g, col)
```

| Rank | Ligand Gene | Receptor Gene | Ligand Expression (Log10(exp + 1)) | Receptor Expression (Log10(exp + 1)) | LR-pair factor value (Percentage) | P-value (Grubbs test) | Q-value (BH method) | L-R Evidence cf. rikenbit/lrbase-workflow | PubMed |
| --- | --- | --- | --- | --- | --- | --- | --- | --- | --- |
| 1 | FN1 Description: FN1fibronectin 1 GO: 1-55 Reactome: 1-49 UniProtKB: 1-6 RefEx: FN1 Expression Atlas: FN1 Single Cell Expression Atlas: FN1 scRNASeqDB: FN1 PanglaoDB: FN1 CMap: FN1 MeSH: 1-97 98-194 195-291 292-388 389-485 486-582 583-679 680-776 777-873 874-970 971-1067 1068-1164 1165-1261 1262-1358 1359-1455 1456-1552 1553-1649 1650-1746 1747-1843 1844-1940 1941-2033 | ITGA8 Description: FN1integrin subunit alpha 8 GO: 1-17 Reactome: 1-5 UniProtKB: 1-2 STRING: 1 RefEx: FN1 Expression Atlas: ITGA8 Single Cell Expression Atlas: ITGA8 scRNASeqDB: ITGA8 PanglaoDB: ITGA8 CMap: ITGA8 MeSH: 1-82 83-164 165-246 247-328 |  |  | 0.021 (45.457%) | 1 | 1 | DLRP | 1 |

# (3,4,) -related Enrichment Analysis

```
load("reanalysis.RData")
library("plotly")
```

## MeSH-Enrichment Analysis (A : Anatomy)

```
negLogPval <- -log10(out.vecLR[['pattern_3_4']]$Enrich$MeSH_A$Pvalue + 1E-10)
term <- out.vecLR[['pattern_3_4']]$Enrich$MeSH_A$Term
target <- seq_len(min(100, length(negLogPval)))
negLogPval <- negLogPval[target]
term <- term[target]
p <- plot_ly(x=seq_along(negLogPval), y=~negLogPval,
type="bar", color=~negLogPval, text=term,
colors=c("#4b61ba", "gray", "#a87963", "red"))
layout(p, title="MeSH-Enrichment Analysis (A)",
   xaxis=list(title="Term"),
   yaxis=list(title="-Log(P-value)"),
   legend=list(name=""))
```

```
## Warning: textfont.color doesn't (yet) support data arrays

## Warning: textfont.color doesn't (yet) support data arrays
```

## MeSH-Enrichment Analysis (B : Organisms)

```
negLogPval <- -log10(out.vecLR[['pattern_3_4']]$Enrich$MeSH_B$Pvalue + 1E-10)
term <- out.vecLR[['pattern_3_4']]$Enrich$MeSH_B$Term
target <- seq_len(min(100, length(negLogPval)))
negLogPval <- negLogPval[target]
term <- term[target]
p <- plot_ly(x=seq_along(negLogPval), y=~negLogPval,
type="bar", color=~negLogPval, text=term,
colors=c("#4b61ba", "gray", "#a87963", "red"))
layout(p, title="MeSH-Enrichment Analysis (B)",
   xaxis=list(title="Term"),
   yaxis=list(title="-Log(P-value)"),
   legend=list(name=""))
```

```
## Warning: textfont.color doesn't (yet) support data arrays

## Warning: textfont.color doesn't (yet) support data arrays
```

## MeSH-Enrichment Analysis (C : Diseases)

```
negLogPval <- -log10(out.vecLR[['pattern_3_4']]$Enrich$MeSH_C$Pvalue + 1E-10)
term <- out.vecLR[['pattern_3_4']]$Enrich$MeSH_C$Term
target <- seq_len(min(100, length(negLogPval)))
negLogPval <- negLogPval[target]
term <- term[target]
p <- plot_ly(x=seq_along(negLogPval), y=~negLogPval,
type="bar", color=~negLogPval, text=term,
colors=c("#4b61ba", "gray", "#a87963", "red"))
layout(p, title="MeSH-Enrichment Analysis (C)",
   xaxis=list(title="Term"),
   yaxis=list(title="-Log(P-value)"),
   legend=list(name=""))
```

```
## Warning: textfont.color doesn't (yet) support data arrays

## Warning: textfont.color doesn't (yet) support data arrays
```

## MeSH-Enrichment Analysis (D : Drugs)

```
negLogPval <- -log10(out.vecLR[['pattern_3_4']]$Enrich$MeSH_D$Pvalue + 1E-10)
term <- out.vecLR[['pattern_3_4']]$Enrich$MeSH_D$Term
target <- seq_len(min(100, length(negLogPval)))
negLogPval <- negLogPval[target]
term <- term[target]
p <- plot_ly(x=seq_along(negLogPval), y=~negLogPval,
type="bar", color=~negLogPval, text=term,
colors=c("#4b61ba", "gray", "#a87963", "red"))
layout(p, title="MeSH-Enrichment Analysis (D)",
   xaxis=list(title="Term"),
   yaxis=list(title="-Log(P-value)"),
   legend=list(name=""))
```

```
## Warning: textfont.color doesn't (yet) support data arrays

## Warning: textfont.color doesn't (yet) support data arrays
```

## MeSH-Enrichment Analysis (E : Analytical, Diagnostic and Therapeutic Techniques and Equipment)

```
negLogPval <- -log10(out.vecLR[['pattern_3_4']]$Enrich$MeSH_E$Pvalue + 1E-10)
term <- out.vecLR[['pattern_3_4']]$Enrich$MeSH_E$Term
target <- seq_len(min(100, length(negLogPval)))
negLogPval <- negLogPval[target]
term <- term[target]
p <- plot_ly(x=seq_along(negLogPval), y=~negLogPval,
type="bar", color=~negLogPval, text=term,
colors=c("#4b61ba", "gray", "#a87963", "red"))
layout(p, title="MeSH-Enrichment Analysis (E)",
   xaxis=list(title="Term"),
   yaxis=list(title="-Log(P-value)"),
   legend=list(name=""))
```

```
## Warning: textfont.color doesn't (yet) support data arrays

## Warning: textfont.color doesn't (yet) support data arrays
```

## MeSH-Enrichment Analysis (F : Psychiatry and Psychology)

```
negLogPval <- -log10(out.vecLR[['pattern_3_4']]$Enrich$MeSH_F$Pvalue + 1E-10)
term <- out.vecLR[['pattern_3_4']]$Enrich$MeSH_F$Term
target <- seq_len(min(100, length(negLogPval)))
negLogPval <- negLogPval[target]
term <- term[target]
p <- plot_ly(x=seq_along(negLogPval), y=~negLogPval,
type="bar", color=~negLogPval, text=term,
colors=c("#4b61ba", "gray", "#a87963", "red"))
layout(p, title="MeSH-Enrichment Analysis (F)",
   xaxis=list(title="Term"),
   yaxis=list(title="-Log(P-value)"),
   legend=list(name=""))
```

## MeSH-Enrichment Analysis (G : Phenomena and Processes)

```
negLogPval <- -log10(out.vecLR[['pattern_3_4']]$Enrich$MeSH_G$Pvalue + 1E-10)
term <- out.vecLR[['pattern_3_4']]$Enrich$MeSH_G$Term
target <- seq_len(min(100, length(negLogPval)))
negLogPval <- negLogPval[target]
term <- term[target]
p <- plot_ly(x=seq_along(negLogPval), y=~negLogPval,
type="bar", color=~negLogPval, text=term,
colors=c("#4b61ba", "gray", "#a87963", "red"))
layout(p, title="MeSH-Enrichment Analysis (G)",
   xaxis=list(title="Term"),
   yaxis=list(title="-Log(P-value)"),
   legend=list(name=""))
```

```
## Warning: textfont.color doesn't (yet) support data arrays

## Warning: textfont.color doesn't (yet) support data arrays
```

## MeSH-Enrichment Analysis (J : Technology and Food and Beverages)

```
negLogPval <- -log10(out.vecLR[['pattern_3_4']]$Enrich$MeSH_J$Pvalue + 1E-10)
term <- out.vecLR[['pattern_3_4']]$Enrich$MeSH_J$Term
target <- seq_len(min(100, length(negLogPval)))
negLogPval <- negLogPval[target]
term <- term[target]
p <- plot_ly(x=seq_along(negLogPval), y=~negLogPval,
type="bar", color=~negLogPval, text=term,
colors=c("#4b61ba", "gray", "#a87963", "red"))
layout(p, title="MeSH-Enrichment Analysis (J)",
   xaxis=list(title="Term"),
   yaxis=list(title="-Log(P-value)"),
   legend=list(name=""))
```

```
## Warning: textfont.color doesn't (yet) support data arrays

## Warning: textfont.color doesn't (yet) support data arrays
```

## MeSH-Enrichment Analysis (N : Health Care)

```
negLogPval <- -log10(out.vecLR[['pattern_3_4']]$Enrich$MeSH_N$Pvalue + 1E-10)
term <- out.vecLR[['pattern_3_4']]$Enrich$MeSH_N$Term
target <- seq_len(min(100, length(negLogPval)))
negLogPval <- negLogPval[target]
term <- term[target]
p <- plot_ly(x=seq_along(negLogPval), y=~negLogPval,
type="bar", color=~negLogPval, text=term,
colors=c("#4b61ba", "gray", "#a87963", "red"))
layout(p, title="MeSH-Enrichment Analysis (N)",
   xaxis=list(title="Term"),
   yaxis=list(title="-Log(P-value)"),
   legend=list(name=""))
```

```
## Warning: textfont.color doesn't (yet) support data arrays

## Warning: textfont.color doesn't (yet) support data arrays
```

## Reactome Pathway-Enrichment Analysis

```
negLogPval <- -log10(out.vecLR[['pattern_3_4']]$Enrich$Reactome$Pvalue + 1E-10)
term <- out.vecLR[['pattern_3_4']]$Enrich$Reactome$Term
target <- seq_len(min(100, length(negLogPval)))
negLogPval <- negLogPval[target]
term <- term[target]
p <- plot_ly(x=seq_along(negLogPval), y=~negLogPval,
type="bar", color=~negLogPval, text=term,
colors=c("#4b61ba", "gray", "#a87963", "red"))
layout(p, title="Reactome-Enrichment Analysis",
   xaxis=list(title="Term"),
   yaxis=list(title="-Log(P-value)"),
   legend=list(name=""))
```

```
## Warning: textfont.color doesn't (yet) support data arrays

## Warning: textfont.color doesn't (yet) support data arrays
```

## DO-Enrichment Analysis

```
negLogPval <- -log10(out.vecLR[['pattern_3_4']]$Enrich$DO$Pvalue + 1E-10)
term <- out.vecLR[['pattern_3_4']]$Enrich$DO$Term
target <- seq_len(min(100, length(negLogPval)))
negLogPval <- negLogPval[target]
term <- term[target]
p <- plot_ly(x=seq_along(negLogPval), y=~negLogPval,
type="bar", color=~negLogPval, text=term,
colors=c("#4b61ba", "gray", "#a87963", "red"))
layout(p, title="DO-Enrichment Analysis",
   xaxis=list(title="Term"),
   yaxis=list(title="-Log(P-value)"),
   legend=list(name=""))
```

```
## Warning: textfont.color doesn't (yet) support data arrays

## Warning: textfont.color doesn't (yet) support data arrays
```

## DGN-Enrichment Analysis

```
negLogPval <- -log10(out.vecLR[['pattern_3_4']]$Enrich$DGN$Pvalue + 1E-10)
term <- out.vecLR[['pattern_3_4']]$Enrich$DGN$Term
target <- seq_len(min(100, length(negLogPval)))
negLogPval <- negLogPval[target]
term <- term[target]
p <- plot_ly(x=seq_along(negLogPval), y=~negLogPval,
type="bar", color=~negLogPval, text=term,
colors=c("#4b61ba", "gray", "#a87963", "red"))
layout(p, title="DGN-Enrichment Analysis",
   xaxis=list(title="Term"),
   yaxis=list(title="-Log(P-value)"),
   legend=list(name=""))
```

```
## Warning: textfont.color doesn't (yet) support data arrays

## Warning: textfont.color doesn't (yet) support data arrays
```
