# Supplementary material for: Sctensor detects many-to-many cell–cell interactions from single cell RNA-sequencing data
Source: BMC Bioinformatics. 2023 Nov 7;24:420. doi: 10.1186/s12859-023-05490-y (PMC10631077; doi:10.1186/s12859-023-05490-y)
Supplement: Supplementary file 14 — Additional file 14. HTML report of FetalKidney. [file 12859_2023_5490_MOESM14_ESM.zip › output/report/Human_FetalKidney/pattern_3_5.html]

Details of (3,5,) -related L-R pairs


# Details of (3,5,) -related L-R pairs

# (3,5,:) Pattern

# (3,5,) -related L-R pairs (Top4 pairs)

```
library("scTensor")
load("reanalysis.RData")
col <- .setColor("many")[17]
scTensor:::.myvisNetwork(g, col)
```

| Rank | Ligand Gene | Receptor Gene | Ligand Expression (Log10(exp + 1)) | Receptor Expression (Log10(exp + 1)) | LR-pair factor value (Percentage) | P-value (Grubbs test) | Q-value (BH method) | L-R Evidence cf. rikenbit/lrbase-workflow | PubMed |
| --- | --- | --- | --- | --- | --- | --- | --- | --- | --- |
| 1 | LAMB1 Description: LAMB1laminin subunit beta 1 GO: 1-28 Reactome: 1-19 UniProtKB: 1-3 RefEx: LAMB1 Expression Atlas: LAMB1 Single Cell Expression Atlas: LAMB1 scRNASeqDB: LAMB1 PanglaoDB: LAMB1 CMap: LAMB1 MeSH: 1-100 101-200 201-300 301-400 401-500 501-600 601-700 701-794 | ITGA6 Description: LAMB1integrin subunit alpha 6 GO: 1-29 Reactome: 1-13 UniProtKB: 1 RefEx: LAMB1 Expression Atlas: ITGA6 Single Cell Expression Atlas: ITGA6 scRNASeqDB: ITGA6 PanglaoDB: ITGA6 CMap: ITGA6 MeSH: 1-94 95-188 189-282 283-376 377-470 471-564 565-658 659-752 753-846 847-940 941-1034 1035-1117 |  |  | 0.01 (26.723%) | 1 | 1 | DLRP | 1 |
| 2 | CXCL12 Description: CXCL12C-X-C motif chemokine ligand 12 GO: 1-53 Reactome: 1-18 UniProtKB: 1 RefEx: CXCL12 Expression Atlas: CXCL12 Single Cell Expression Atlas: CXCL12 scRNASeqDB: CXCL12 PanglaoDB: CXCL12 CMap: CXCL12 MeSH: 1-100 101-200 201-300 301-400 401-500 501-600 601-700 701-800 801-900 901-1000 1001-1100 1101-1200 1201-1300 1301-1400 1401-1500 1501-1600 1601-1700 1701-1800 1801-1900 1901-2000 2001-2100 2101-2200 2201-2300 2301-2400 2401-2500 2501-2600 2601-2700 2701-2800 2801-2900 2901-3000 3001-3100 3101-3200 3201-3275 | CXCR4 Description: CXCL12C-X-C motif chemokine receptor 4 GO: 1-67 Reactome: 1-18 UniProtKB: 1-3 STRING: 1 2 RefEx: CXCL12 Expression Atlas: CXCR4 Single Cell Expression Atlas: CXCR4 scRNASeqDB: CXCR4 PanglaoDB: CXCR4 CMap: CXCR4 MeSH: 1-100 101-200 201-300 301-400 401-500 501-600 601-700 701-800 801-900 901-1000 1001-1100 1101-1200 1201-1300 1301-1400 1401-1500 1501-1600 1601-1700 1701-1800 1801-1900 1901-2000 2001-2100 2101-2200 2201-2300 2301-2400 2401-2500 2501-2600 2601-2700 2701-2780 |  |  | 0.004 (10.017%) | 0.13 | 0.151 | DLRP | 1 |
| 3 | HAS2 Description: HAS2hyaluronan synthase 2 GO: 1-29 Reactome: 1-5 UniProtKB: 1 STRING: 1 RefEx: HAS2 Expression Atlas: HAS2 Single Cell Expression Atlas: HAS2 scRNASeqDB: HAS2 PanglaoDB: HAS2 CMap: HAS2 MeSH: 1-95 96-190 191-285 286-380 381-475 476-570 571-665 666-757 | HMMR Description: HAS2hyaluronan mediated motility receptor GO: 1-8 Reactome: 1-10 UniProtKB: 1 RefEx: HAS2 Expression Atlas: HMMR Single Cell Expression Atlas: HMMR scRNASeqDB: HMMR PanglaoDB: HMMR CMap: HMMR MeSH: 1-91 92-182 183-273 274-364 365-455 456-546 547-634 |  |  | 0.003 (8.483%) | 1 | 1 | DLRP | 1 |
| 4 | EFNB2 Description: EFNB2ephrin B2 GO: 1-23 Reactome: 1-7 UniProtKB: 1 STRING: 1 RefEx: EFNB2 Expression Atlas: EFNB2 Single Cell Expression Atlas: EFNB2 scRNASeqDB: EFNB2 PanglaoDB: EFNB2 CMap: EFNB2 MeSH: 1-98 99-196 197-294 295-392 393-490 491-588 589-686 687-782 | EPHB3 Description: EFNB2EPH receptor B3 GO: 1-37 Reactome: 1-7 UniProtKB: 1 STRING: 1 RefEx: EFNB2 Expression Atlas: EPHB3 Single Cell Expression Atlas: EPHB3 scRNASeqDB: EPHB3 PanglaoDB: EPHB3 CMap: EPHB3 MeSH: 1-84 85-168 169-252 253-335 |  |  | 0.003 (7.749%) | 0 | 0 | DLRP | 1 |

# (3,5,) -related Enrichment Analysis

```
load("reanalysis.RData")
library("plotly")
```

## MeSH-Enrichment Analysis (A : Anatomy)

```
negLogPval <- -log10(out.vecLR[['pattern_3_5']]$Enrich$MeSH_A$Pvalue + 1E-10)
term <- out.vecLR[['pattern_3_5']]$Enrich$MeSH_A$Term
target <- seq_len(min(100, length(negLogPval)))
negLogPval <- negLogPval[target]
term <- term[target]
p <- plot_ly(x=seq_along(negLogPval), y=~negLogPval,
type="bar", color=~negLogPval, text=term,
colors=c("#4b61ba", "gray", "#a87963", "red"))
layout(p, title="MeSH-Enrichment Analysis (A)",
   xaxis=list(title="Term"),
   yaxis=list(title="-Log(P-value)"),
   legend=list(name=""))
```

```
## Warning: textfont.color doesn't (yet) support data arrays

## Warning: textfont.color doesn't (yet) support data arrays
```

## MeSH-Enrichment Analysis (B : Organisms)

```
negLogPval <- -log10(out.vecLR[['pattern_3_5']]$Enrich$MeSH_B$Pvalue + 1E-10)
term <- out.vecLR[['pattern_3_5']]$Enrich$MeSH_B$Term
target <- seq_len(min(100, length(negLogPval)))
negLogPval <- negLogPval[target]
term <- term[target]
p <- plot_ly(x=seq_along(negLogPval), y=~negLogPval,
type="bar", color=~negLogPval, text=term,
colors=c("#4b61ba", "gray", "#a87963", "red"))
layout(p, title="MeSH-Enrichment Analysis (B)",
   xaxis=list(title="Term"),
   yaxis=list(title="-Log(P-value)"),
   legend=list(name=""))
```

```
## Warning: textfont.color doesn't (yet) support data arrays

## Warning: textfont.color doesn't (yet) support data arrays
```

## MeSH-Enrichment Analysis (C : Diseases)

```
negLogPval <- -log10(out.vecLR[['pattern_3_5']]$Enrich$MeSH_C$Pvalue + 1E-10)
term <- out.vecLR[['pattern_3_5']]$Enrich$MeSH_C$Term
target <- seq_len(min(100, length(negLogPval)))
negLogPval <- negLogPval[target]
term <- term[target]
p <- plot_ly(x=seq_along(negLogPval), y=~negLogPval,
type="bar", color=~negLogPval, text=term,
colors=c("#4b61ba", "gray", "#a87963", "red"))
layout(p, title="MeSH-Enrichment Analysis (C)",
   xaxis=list(title="Term"),
   yaxis=list(title="-Log(P-value)"),
   legend=list(name=""))
```

```
## Warning: textfont.color doesn't (yet) support data arrays

## Warning: textfont.color doesn't (yet) support data arrays
```

## MeSH-Enrichment Analysis (D : Drugs)

```
negLogPval <- -log10(out.vecLR[['pattern_3_5']]$Enrich$MeSH_D$Pvalue + 1E-10)
term <- out.vecLR[['pattern_3_5']]$Enrich$MeSH_D$Term
target <- seq_len(min(100, length(negLogPval)))
negLogPval <- negLogPval[target]
term <- term[target]
p <- plot_ly(x=seq_along(negLogPval), y=~negLogPval,
type="bar", color=~negLogPval, text=term,
colors=c("#4b61ba", "gray", "#a87963", "red"))
layout(p, title="MeSH-Enrichment Analysis (D)",
   xaxis=list(title="Term"),
   yaxis=list(title="-Log(P-value)"),
   legend=list(name=""))
```

```
## Warning: textfont.color doesn't (yet) support data arrays

## Warning: textfont.color doesn't (yet) support data arrays
```

## MeSH-Enrichment Analysis (E : Analytical, Diagnostic and Therapeutic Techniques and Equipment)

```
negLogPval <- -log10(out.vecLR[['pattern_3_5']]$Enrich$MeSH_E$Pvalue + 1E-10)
term <- out.vecLR[['pattern_3_5']]$Enrich$MeSH_E$Term
target <- seq_len(min(100, length(negLogPval)))
negLogPval <- negLogPval[target]
term <- term[target]
p <- plot_ly(x=seq_along(negLogPval), y=~negLogPval,
type="bar", color=~negLogPval, text=term,
colors=c("#4b61ba", "gray", "#a87963", "red"))
layout(p, title="MeSH-Enrichment Analysis (E)",
   xaxis=list(title="Term"),
   yaxis=list(title="-Log(P-value)"),
   legend=list(name=""))
```

```
## Warning: textfont.color doesn't (yet) support data arrays

## Warning: textfont.color doesn't (yet) support data arrays
```

## MeSH-Enrichment Analysis (G : Phenomena and Processes)

```
negLogPval <- -log10(out.vecLR[['pattern_3_5']]$Enrich$MeSH_G$Pvalue + 1E-10)
term <- out.vecLR[['pattern_3_5']]$Enrich$MeSH_G$Term
target <- seq_len(min(100, length(negLogPval)))
negLogPval <- negLogPval[target]
term <- term[target]
p <- plot_ly(x=seq_along(negLogPval), y=~negLogPval,
type="bar", color=~negLogPval, text=term,
colors=c("#4b61ba", "gray", "#a87963", "red"))
layout(p, title="MeSH-Enrichment Analysis (G)",
   xaxis=list(title="Term"),
   yaxis=list(title="-Log(P-value)"),
   legend=list(name=""))
```

```
## Warning: textfont.color doesn't (yet) support data arrays

## Warning: textfont.color doesn't (yet) support data arrays
```

## MeSH-Enrichment Analysis (H : Disciplines and Occupations)

```
negLogPval <- -log10(out.vecLR[['pattern_3_5']]$Enrich$MeSH_H$Pvalue + 1E-10)
term <- out.vecLR[['pattern_3_5']]$Enrich$MeSH_H$Term
target <- seq_len(min(100, length(negLogPval)))
negLogPval <- negLogPval[target]
term <- term[target]
p <- plot_ly(x=seq_along(negLogPval), y=~negLogPval,
type="bar", color=~negLogPval, text=term,
colors=c("#4b61ba", "gray", "#a87963", "red"))
layout(p, title="MeSH-Enrichment Analysis (H)",
   xaxis=list(title="Term"),
   yaxis=list(title="-Log(P-value)"),
   legend=list(name=""))
```

## MeSH-Enrichment Analysis (J : Technology and Food and Beverages)

```
negLogPval <- -log10(out.vecLR[['pattern_3_5']]$Enrich$MeSH_J$Pvalue + 1E-10)
term <- out.vecLR[['pattern_3_5']]$Enrich$MeSH_J$Term
target <- seq_len(min(100, length(negLogPval)))
negLogPval <- negLogPval[target]
term <- term[target]
p <- plot_ly(x=seq_along(negLogPval), y=~negLogPval,
type="bar", color=~negLogPval, text=term,
colors=c("#4b61ba", "gray", "#a87963", "red"))
layout(p, title="MeSH-Enrichment Analysis (J)",
   xaxis=list(title="Term"),
   yaxis=list(title="-Log(P-value)"),
   legend=list(name=""))
```

```
## Warning: textfont.color doesn't (yet) support data arrays

## Warning: textfont.color doesn't (yet) support data arrays
```

## MeSH-Enrichment Analysis (L : Information Science)

```
negLogPval <- -log10(out.vecLR[['pattern_3_5']]$Enrich$MeSH_L$Pvalue + 1E-10)
term <- out.vecLR[['pattern_3_5']]$Enrich$MeSH_L$Term
target <- seq_len(min(100, length(negLogPval)))
negLogPval <- negLogPval[target]
term <- term[target]
p <- plot_ly(x=seq_along(negLogPval), y=~negLogPval,
type="bar", color=~negLogPval, text=term,
colors=c("#4b61ba", "gray", "#a87963", "red"))
layout(p, title="MeSH-Enrichment Analysis (L)",
   xaxis=list(title="Term"),
   yaxis=list(title="-Log(P-value)"),
   legend=list(name=""))
```

```
## Warning: textfont.color doesn't (yet) support data arrays

## Warning: textfont.color doesn't (yet) support data arrays
```

## MeSH-Enrichment Analysis (N : Health Care)

```
negLogPval <- -log10(out.vecLR[['pattern_3_5']]$Enrich$MeSH_N$Pvalue + 1E-10)
term <- out.vecLR[['pattern_3_5']]$Enrich$MeSH_N$Term
target <- seq_len(min(100, length(negLogPval)))
negLogPval <- negLogPval[target]
term <- term[target]
p <- plot_ly(x=seq_along(negLogPval), y=~negLogPval,
type="bar", color=~negLogPval, text=term,
colors=c("#4b61ba", "gray", "#a87963", "red"))
layout(p, title="MeSH-Enrichment Analysis (N)",
   xaxis=list(title="Term"),
   yaxis=list(title="-Log(P-value)"),
   legend=list(name=""))
```

```
## Warning: textfont.color doesn't (yet) support data arrays

## Warning: textfont.color doesn't (yet) support data arrays
```

## MeSH-Enrichment Analysis (Z : Geographical Locations)

```
negLogPval <- -log10(out.vecLR[['pattern_3_5']]$Enrich$MeSH_Z$Pvalue + 1E-10)
term <- out.vecLR[['pattern_3_5']]$Enrich$MeSH_Z$Term
target <- seq_len(min(100, length(negLogPval)))
negLogPval <- negLogPval[target]
term <- term[target]
p <- plot_ly(x=seq_along(negLogPval), y=~negLogPval,
type="bar", color=~negLogPval, text=term,
colors=c("#4b61ba", "gray", "#a87963", "red"))
layout(p, title="MeSH-Enrichment Analysis (Z)",
   xaxis=list(title="Term"),
   yaxis=list(title="-Log(P-value)"),
   legend=list(name=""))
```

## Reactome Pathway-Enrichment Analysis

```
negLogPval <- -log10(out.vecLR[['pattern_3_5']]$Enrich$Reactome$Pvalue + 1E-10)
term <- out.vecLR[['pattern_3_5']]$Enrich$Reactome$Term
target <- seq_len(min(100, length(negLogPval)))
negLogPval <- negLogPval[target]
term <- term[target]
p <- plot_ly(x=seq_along(negLogPval), y=~negLogPval,
type="bar", color=~negLogPval, text=term,
colors=c("#4b61ba", "gray", "#a87963", "red"))
layout(p, title="Reactome-Enrichment Analysis",
   xaxis=list(title="Term"),
   yaxis=list(title="-Log(P-value)"),
   legend=list(name=""))
```

```
## Warning: textfont.color doesn't (yet) support data arrays

## Warning: textfont.color doesn't (yet) support data arrays
```

## DO-Enrichment Analysis

```
negLogPval <- -log10(out.vecLR[['pattern_3_5']]$Enrich$DO$Pvalue + 1E-10)
term <- out.vecLR[['pattern_3_5']]$Enrich$DO$Term
target <- seq_len(min(100, length(negLogPval)))
negLogPval <- negLogPval[target]
term <- term[target]
p <- plot_ly(x=seq_along(negLogPval), y=~negLogPval,
type="bar", color=~negLogPval, text=term,
colors=c("#4b61ba", "gray", "#a87963", "red"))
layout(p, title="DO-Enrichment Analysis",
   xaxis=list(title="Term"),
   yaxis=list(title="-Log(P-value)"),
   legend=list(name=""))
```

```
## Warning: textfont.color doesn't (yet) support data arrays

## Warning: textfont.color doesn't (yet) support data arrays
```

## NCG-Enrichment Analysis

```
negLogPval <- -log10(out.vecLR[['pattern_3_5']]$Enrich$NCG$Pvalue + 1E-10)
term <- out.vecLR[['pattern_3_5']]$Enrich$NCG$Term
target <- seq_len(min(100, length(negLogPval)))
negLogPval <- negLogPval[target]
term <- term[target]
p <- plot_ly(x=seq_along(negLogPval), y=~negLogPval,
type="bar", color=~negLogPval, text=term,
colors=c("#4b61ba", "gray", "#a87963", "red"))
layout(p, title="NCG-Enrichment Analysis",
   xaxis=list(title="Term"),
   yaxis=list(title="-Log(P-value)"),
   legend=list(name=""))
```

```
## Warning: textfont.color doesn't (yet) support data arrays

## Warning: textfont.color doesn't (yet) support data arrays
```

## DGN-Enrichment Analysis

```
negLogPval <- -log10(out.vecLR[['pattern_3_5']]$Enrich$DGN$Pvalue + 1E-10)
term <- out.vecLR[['pattern_3_5']]$Enrich$DGN$Term
target <- seq_len(min(100, length(negLogPval)))
negLogPval <- negLogPval[target]
term <- term[target]
p <- plot_ly(x=seq_along(negLogPval), y=~negLogPval,
type="bar", color=~negLogPval, text=term,
colors=c("#4b61ba", "gray", "#a87963", "red"))
layout(p, title="DGN-Enrichment Analysis",
   xaxis=list(title="Term"),
   yaxis=list(title="-Log(P-value)"),
   legend=list(name=""))
```

```
## Warning: textfont.color doesn't (yet) support data arrays

## Warning: textfont.color doesn't (yet) support data arrays
```
