# Supplementary material for: Sctensor detects many-to-many cell–cell interactions from single cell RNA-sequencing data
Source: BMC Bioinformatics. 2023 Nov 7;24:420. doi: 10.1186/s12859-023-05490-y (PMC10631077; doi:10.1186/s12859-023-05490-y)
Supplement: Supplementary file 14 — Additional file 14. HTML report of FetalKidney. [file 12859_2023_5490_MOESM14_ESM.zip › output/report/Human_FetalKidney/pattern_4_4.html]

Details of (4,4,) -related L-R pairs


# Details of (4,4,) -related L-R pairs

# (4,4,:) Pattern

# (4,4,) -related L-R pairs (Top14 pairs)

```
library("scTensor")
load("reanalysis.RData")
col <- .setColor("many")[19]
scTensor:::.myvisNetwork(g, col)
```

| Rank | Ligand Gene | Receptor Gene | Ligand Expression (Log10(exp + 1)) | Receptor Expression (Log10(exp + 1)) | LR-pair factor value (Percentage) | P-value (Grubbs test) | Q-value (BH method) | L-R Evidence cf. rikenbit/lrbase-workflow | PubMed |
| --- | --- | --- | --- | --- | --- | --- | --- | --- | --- |
| 1 | LAMB1 Description: LAMB1laminin subunit beta 1 GO: 1-28 Reactome: 1-19 UniProtKB: 1-3 RefEx: LAMB1 Expression Atlas: LAMB1 Single Cell Expression Atlas: LAMB1 scRNASeqDB: LAMB1 PanglaoDB: LAMB1 CMap: LAMB1 MeSH: 1-100 101-200 201-300 301-400 401-500 501-600 601-700 701-794 | ITGA6 Description: LAMB1integrin subunit alpha 6 GO: 1-29 Reactome: 1-13 UniProtKB: 1 RefEx: LAMB1 Expression Atlas: ITGA6 Single Cell Expression Atlas: ITGA6 scRNASeqDB: ITGA6 PanglaoDB: ITGA6 CMap: ITGA6 MeSH: 1-94 95-188 189-282 283-376 377-470 471-564 565-658 659-752 753-846 847-940 941-1034 1035-1117 |  |  | 0.01 (30.744%) | 0 | 0 | DLRP | 1 |
| 2 | FN1 Description: FN1fibronectin 1 GO: 1-55 Reactome: 1-49 UniProtKB: 1-6 RefEx: FN1 Expression Atlas: FN1 Single Cell Expression Atlas: FN1 scRNASeqDB: FN1 PanglaoDB: FN1 CMap: FN1 MeSH: 1-97 98-194 195-291 292-388 389-485 486-582 583-679 680-776 777-873 874-970 971-1067 1068-1164 1165-1261 1262-1358 1359-1455 1456-1552 1553-1649 1650-1746 1747-1843 1844-1940 1941-2033 | ITGA8 Description: FN1integrin subunit alpha 8 GO: 1-17 Reactome: 1-5 UniProtKB: 1-2 STRING: 1 RefEx: FN1 Expression Atlas: ITGA8 Single Cell Expression Atlas: ITGA8 scRNASeqDB: ITGA8 PanglaoDB: ITGA8 CMap: ITGA8 MeSH: 1-82 83-164 165-246 247-328 |  |  | 0.004 (10.465%) | 0.005 | 0.012 | DLRP | 1 |
| 3 | CXCL12 Description: CXCL12C-X-C motif chemokine ligand 12 GO: 1-53 Reactome: 1-18 UniProtKB: 1 RefEx: CXCL12 Expression Atlas: CXCL12 Single Cell Expression Atlas: CXCL12 scRNASeqDB: CXCL12 PanglaoDB: CXCL12 CMap: CXCL12 MeSH: 1-100 101-200 201-300 301-400 401-500 501-600 601-700 701-800 801-900 901-1000 1001-1100 1101-1200 1201-1300 1301-1400 1401-1500 1501-1600 1601-1700 1701-1800 1801-1900 1901-2000 2001-2100 2101-2200 2201-2300 2301-2400 2401-2500 2501-2600 2601-2700 2701-2800 2801-2900 2901-3000 3001-3100 3101-3200 3201-3275 | CXCR4 Description: CXCL12C-X-C motif chemokine receptor 4 GO: 1-67 Reactome: 1-18 UniProtKB: 1-3 STRING: 1 2 RefEx: CXCL12 Expression Atlas: CXCR4 Single Cell Expression Atlas: CXCR4 scRNASeqDB: CXCR4 PanglaoDB: CXCR4 CMap: CXCR4 MeSH: 1-100 101-200 201-300 301-400 401-500 501-600 601-700 701-800 801-900 901-1000 1001-1100 1101-1200 1201-1300 1301-1400 1401-1500 1501-1600 1601-1700 1701-1800 1801-1900 1901-2000 2001-2100 2101-2200 2201-2300 2301-2400 2401-2500 2501-2600 2601-2700 2701-2780 |  |  | 0.003 (10.093%) | 0 | 0 | DLRP | 1 |
| 4 | BMP7 Description: BMP7bone morphogenetic protein 7 GO: 1-86 Reactome: 1-3 UniProtKB: 1-2 RefEx: BMP7 Expression Atlas: BMP7 Single Cell Expression Atlas: BMP7 scRNASeqDB: BMP7 PanglaoDB: BMP7 CMap: BMP7 MeSH: 1-95 96-190 191-285 286-380 381-475 476-570 571-665 666-760 761-855 856-950 951-1045 1046-1140 1141-1235 1236-1330 1331-1425 1426-1520 1521-1615 1616-1710 1711-1801 | ACVR2B Description: BMP7activin A receptor type 2B GO: 1-33 Reactome: 1-7 UniProtKB: 1 RefEx: BMP7 Expression Atlas: ACVR2B Single Cell Expression Atlas: ACVR2B scRNASeqDB: ACVR2B PanglaoDB: ACVR2B CMap: ACVR2B MeSH: 1-86 87-172 173-258 259-344 345-430 431-515 |  |  | 0.002 (5.407%) | 0.039 | 0.063 | DLRP | 1 |
| 5 | APOE Description: APOEapolipoprotein E GO: 1-74 75-147 Reactome: 1-34 UniProtKB: 1-2 RefEx: APOE Expression Atlas: APOE Single Cell Expression Atlas: APOE scRNASeqDB: APOE PanglaoDB: APOE CMap: APOE MeSH: 1-99 100-198 199-297 298-396 397-495 496-594 595-693 694-792 793-891 892-990 991-1089 1090-1188 1189-1287 1288-1386 1387-1485 1486-1584 1585-1683 1684-1782 1783-1881 1882-1980 1981-2079 2080-2178 2179-2277 2278-2376 2377-2475 2476-2574 2575-2673 2674-2772 2773-2871 2872-2970 2971-3069 3070-3168 3169-3267 3268-3366 3367-3465 3466-3564 3565-3663 3664-3762 3763-3861 3862-3960 3961-4059 4060-4158 4159-4257 4258-4338 | SORL1 Description: APOEsortilin related receptor 1 GO: 1-65 Reactome: 1-2 UniProtKB: 1-2 RefEx: APOE Expression Atlas: SORL1 Single Cell Expression Atlas: SORL1 scRNASeqDB: SORL1 PanglaoDB: SORL1 CMap: SORL1 MeSH: 1-92 93-184 185-276 277-368 369-460 461-552 553-644 645-734 |  |  | 0.002 (5.386%) | 0.005 | 0.013 | DLRP | 1 |
| 6 | SEMA3B Description: SEMA3Bsemaphorin 3B GO: 1-14 UniProtKB: 1-4 RefEx: SEMA3B Expression Atlas: SEMA3B Single Cell Expression Atlas: SEMA3B scRNASeqDB: SEMA3B PanglaoDB: SEMA3B CMap: SEMA3B MeSH: 1-96 97-192 193-288 289-381 | NRP1 Description: SEMA3Bneuropilin 1 GO: 1-58 59-116 Reactome: 1-15 UniProtKB: 1-6 RefEx: SEMA3B Expression Atlas: NRP1 Single Cell Expression Atlas: NRP1 scRNASeqDB: NRP1 PanglaoDB: NRP1 CMap: NRP1 MeSH: 1-96 97-192 193-288 289-384 385-480 481-576 577-672 673-768 769-864 865-960 961-1056 1057-1147 |  |  | 0.002 (4.49%) | 0.176 | 0.217 | DLRP | 1 |
| 7 | COL4A1 Description: COL4A1collagen type IV alpha 1 chain GO: 1-22 Reactome: 1-24 UniProtKB: 1-3 RefEx: COL4A1 Expression Atlas: COL4A1 Single Cell Expression Atlas: COL4A1 scRNASeqDB: COL4A1 PanglaoDB: COL4A1 CMap: COL4A1 MeSH: 1-94 95-188 189-282 283-376 377-470 471-564 565-658 659-752 753-846 847-940 941-1032 | ITGB8 Description: COL4A1integrin subunit beta 8 GO: 1-26 Reactome: 1-4 UniProtKB: 1-2 RefEx: COL4A1 Expression Atlas: ITGB8 Single Cell Expression Atlas: ITGB8 scRNASeqDB: ITGB8 PanglaoDB: ITGB8 CMap: ITGB8 MeSH: 1-100 101-200 201-300 301-400 |  |  | 0.001 (4.184%) | 0.08 | 0.108 | DLRP | 1 |
| 8 | CALM1 Description: CALM1calmodulin 1 GO: 1-75 Reactome: 1-68 69-136 UniProtKB: 1-4 RefEx: CALM1 Expression Atlas: CALM1 Single Cell Expression Atlas: CALM1 scRNASeqDB: CALM1 PanglaoDB: CALM1 CMap: CALM1 MeSH: 1-100 101-200 201-300 301-400 401-500 501-600 601-700 701-800 801-900 901-1000 1001-1100 1101-1200 1201-1300 1301-1400 1401-1495 | MYLK Description: CALM1myosin light chain kinase GO: 1-23 Reactome: 1-7 UniProtKB: 1-3 RefEx: CALM1 Expression Atlas: MYLK Single Cell Expression Atlas: MYLK scRNASeqDB: MYLK PanglaoDB: MYLK CMap: MYLK MeSH: 1-97 98-194 195-291 292-388 389-485 486-582 583-679 680-775 |  |  | 0.001 (3.974%) | 0.002 | 0.005 | DLRP | 1 |
| 9 | LAMA1 Description: LAMA1laminin subunit alpha 1 GO: 1-30 Reactome: 1-13 UniProtKB: 1 RefEx: LAMA1 Expression Atlas: LAMA1 Single Cell Expression Atlas: LAMA1 scRNASeqDB: LAMA1 PanglaoDB: LAMA1 CMap: LAMA1 MeSH: 1-97 98-194 195-291 292-388 389-485 486-582 583-679 680-776 777-873 874-961 | ITGB8 Description: LAMA1integrin subunit beta 8 GO: 1-26 Reactome: 1-4 UniProtKB: 1-2 RefEx: LAMA1 Expression Atlas: ITGB8 Single Cell Expression Atlas: ITGB8 scRNASeqDB: ITGB8 PanglaoDB: ITGB8 CMap: ITGB8 MeSH: 1-100 101-200 201-300 301-400 |  |  | 0.001 (3.09%) | 1 | 1 | DLRP | 1 |
| 10 | FGF9 Description: FGF9fibroblast growth factor 9 GO: 1-25 Reactome: 1-70 UniProtKB: 1 STRING: 1 RefEx: FGF9 Expression Atlas: FGF9 Single Cell Expression Atlas: FGF9 scRNASeqDB: FGF9 PanglaoDB: FGF9 CMap: FGF9 MeSH: 1-91 92-182 183-273 274-364 365-455 456-546 547-637 638-724 | FGFR3 Description: FGF9fibroblast growth factor receptor 3 GO: 1-41 Reactome: 1-38 UniProtKB: 1-2 RefEx: FGF9 Expression Atlas: FGFR3 Single Cell Expression Atlas: FGFR3 scRNASeqDB: FGFR3 PanglaoDB: FGFR3 CMap: FGFR3 MeSH: 1-98 99-196 197-294 295-392 393-490 491-588 589-686 687-784 785-882 883-980 981-1078 1079-1176 1177-1274 1275-1372 1373-1470 1471-1568 1569-1666 1667-1753 |  |  | 0.001 (2.929%) | 0 | 0 | DLRP | 1 |
| 11 | NTN4 Description: NTN4netrin 4 GO: 1-12 Reactome: 1-6 UniProtKB: 1-3 RefEx: NTN4 Expression Atlas: NTN4 Single Cell Expression Atlas: NTN4 scRNASeqDB: NTN4 PanglaoDB: NTN4 CMap: NTN4 MeSH: 1-83 84-166 167-249 250-329 | DCC Description: NTN4DCC netrin 1 receptor GO: 1-20 Reactome: 1-14 UniProtKB: 1-2 RefEx: NTN4 Expression Atlas: DCC Single Cell Expression Atlas: DCC scRNASeqDB: DCC PanglaoDB: DCC CMap: DCC MeSH: 1-96 97-192 193-288 289-384 385-480 481-576 577-672 673-768 769-864 865-953 |  |  | 0.001 (2.586%) | 0.005 | 0.012 | DLRP | 1 |
| 12 | COL4A1 Description: COL4A1collagen type IV alpha 1 chain GO: 1-22 Reactome: 1-24 UniProtKB: 1-3 RefEx: COL4A1 Expression Atlas: COL4A1 Single Cell Expression Atlas: COL4A1 scRNASeqDB: COL4A1 PanglaoDB: COL4A1 CMap: COL4A1 MeSH: 1-94 95-188 189-282 283-376 377-470 471-564 565-658 659-752 753-846 847-940 941-1032 | ITGA1 Description: COL4A1integrin subunit alpha 1 GO: 1-29 Reactome: 1-14 UniProtKB: 1 RefEx: COL4A1 Expression Atlas: ITGA1 Single Cell Expression Atlas: ITGA1 scRNASeqDB: ITGA1 PanglaoDB: ITGA1 CMap: ITGA1 MeSH: 1-91 92-182 183-273 274-364 365-455 456-546 547-637 638-728 729-817 |  |  | 0.001 (2.465%) | 0.338 | 0.362 | DLRP | 1 |
| 13 | KNG1 Description: KNG1kininogen 1 GO: 1-23 Reactome: 1-18 UniProtKB: 1 RefEx: KNG1 Expression Atlas: KNG1 Single Cell Expression Atlas: KNG1 scRNASeqDB: KNG1 PanglaoDB: KNG1 CMap: KNG1 MeSH: 1-92 93-184 185-276 277-368 369-460 461-552 553-644 645-736 737-828 829-919 | GPR135 Description: KNG1G protein-coupled receptor 135 GO: 1-8 UniProtKB: 1 RefEx: KNG1 Expression Atlas: GPR135 Single Cell Expression Atlas: GPR135 scRNASeqDB: GPR135 PanglaoDB: GPR135 CMap: GPR135 MeSH: 1-72 73-144 |  |  | 0.001 (1.941%) | 0.018 | 0.037 | DLRP | 1 |
| 14 | VEGFA Description: VEGFAvascular endothelial growth factor A GO: 1-62 63-123 Reactome: 1-28 UniProtKB: 1-9 RefEx: VEGFA Expression Atlas: VEGFA Single Cell Expression Atlas: VEGFA scRNASeqDB: VEGFA PanglaoDB: VEGFA CMap: VEGFA MeSH: 1-100 101-200 201-300 301-400 401-500 501-600 601-700 701-800 801-900 901-1000 1001-1100 1101-1200 1201-1300 1301-1400 1401-1500 1501-1600 1601-1700 1701-1800 1801-1900 1901-2000 2001-2100 2101-2200 2201-2300 2301-2400 2401-2500 2501-2600 2601-2700 2701-2800 2801-2900 2901-3000 3001-3100 3101-3200 3201-3300 3301-3400 3401-3500 3501-3600 3601-3700 3701-3800 3801-3900 3901-4000 4001-4100 4101-4200 4201-4300 4301-4400 4401-4500 4501-4600 4601-4700 4701-4800 4801-4900 4901-5000 5001-5100 5101-5200 5201-5300 5301-5400 5401-5500 5501-5556 | NRP1 Description: VEGFAneuropilin 1 GO: 1-58 59-116 Reactome: 1-15 UniProtKB: 1-6 RefEx: VEGFA Expression Atlas: NRP1 Single Cell Expression Atlas: NRP1 scRNASeqDB: NRP1 PanglaoDB: NRP1 CMap: NRP1 MeSH: 1-96 97-192 193-288 289-384 385-480 481-576 577-672 673-768 769-864 865-960 961-1056 1057-1147 |  |  | 0.001 (1.909%) | 0.041 | 0.065 | DLRP | 1 |

# (4,4,) -related Enrichment Analysis

```
load("reanalysis.RData")
library("plotly")
```

## MeSH-Enrichment Analysis (A : Anatomy)

```
negLogPval <- -log10(out.vecLR[['pattern_4_4']]$Enrich$MeSH_A$Pvalue + 1E-10)
term <- out.vecLR[['pattern_4_4']]$Enrich$MeSH_A$Term
target <- seq_len(min(100, length(negLogPval)))
negLogPval <- negLogPval[target]
term <- term[target]
p <- plot_ly(x=seq_along(negLogPval), y=~negLogPval,
type="bar", color=~negLogPval, text=term,
colors=c("#4b61ba", "gray", "#a87963", "red"))
layout(p, title="MeSH-Enrichment Analysis (A)",
   xaxis=list(title="Term"),
   yaxis=list(title="-Log(P-value)"),
   legend=list(name=""))
```

```
## Warning: textfont.color doesn't (yet) support data arrays

## Warning: textfont.color doesn't (yet) support data arrays
```

## MeSH-Enrichment Analysis (B : Organisms)

```
negLogPval <- -log10(out.vecLR[['pattern_4_4']]$Enrich$MeSH_B$Pvalue + 1E-10)
term <- out.vecLR[['pattern_4_4']]$Enrich$MeSH_B$Term
target <- seq_len(min(100, length(negLogPval)))
negLogPval <- negLogPval[target]
term <- term[target]
p <- plot_ly(x=seq_along(negLogPval), y=~negLogPval,
type="bar", color=~negLogPval, text=term,
colors=c("#4b61ba", "gray", "#a87963", "red"))
layout(p, title="MeSH-Enrichment Analysis (B)",
   xaxis=list(title="Term"),
   yaxis=list(title="-Log(P-value)"),
   legend=list(name=""))
```

```
## Warning: textfont.color doesn't (yet) support data arrays

## Warning: textfont.color doesn't (yet) support data arrays
```

## MeSH-Enrichment Analysis (C : Diseases)

```
negLogPval <- -log10(out.vecLR[['pattern_4_4']]$Enrich$MeSH_C$Pvalue + 1E-10)
term <- out.vecLR[['pattern_4_4']]$Enrich$MeSH_C$Term
target <- seq_len(min(100, length(negLogPval)))
negLogPval <- negLogPval[target]
term <- term[target]
p <- plot_ly(x=seq_along(negLogPval), y=~negLogPval,
type="bar", color=~negLogPval, text=term,
colors=c("#4b61ba", "gray", "#a87963", "red"))
layout(p, title="MeSH-Enrichment Analysis (C)",
   xaxis=list(title="Term"),
   yaxis=list(title="-Log(P-value)"),
   legend=list(name=""))
```

```
## Warning: textfont.color doesn't (yet) support data arrays

## Warning: textfont.color doesn't (yet) support data arrays
```

## MeSH-Enrichment Analysis (D : Drugs)

```
negLogPval <- -log10(out.vecLR[['pattern_4_4']]$Enrich$MeSH_D$Pvalue + 1E-10)
term <- out.vecLR[['pattern_4_4']]$Enrich$MeSH_D$Term
target <- seq_len(min(100, length(negLogPval)))
negLogPval <- negLogPval[target]
term <- term[target]
p <- plot_ly(x=seq_along(negLogPval), y=~negLogPval,
type="bar", color=~negLogPval, text=term,
colors=c("#4b61ba", "gray", "#a87963", "red"))
layout(p, title="MeSH-Enrichment Analysis (D)",
   xaxis=list(title="Term"),
   yaxis=list(title="-Log(P-value)"),
   legend=list(name=""))
```

```
## Warning: textfont.color doesn't (yet) support data arrays

## Warning: textfont.color doesn't (yet) support data arrays
```

## MeSH-Enrichment Analysis (E : Analytical, Diagnostic and Therapeutic Techniques and Equipment)

```
negLogPval <- -log10(out.vecLR[['pattern_4_4']]$Enrich$MeSH_E$Pvalue + 1E-10)
term <- out.vecLR[['pattern_4_4']]$Enrich$MeSH_E$Term
target <- seq_len(min(100, length(negLogPval)))
negLogPval <- negLogPval[target]
term <- term[target]
p <- plot_ly(x=seq_along(negLogPval), y=~negLogPval,
type="bar", color=~negLogPval, text=term,
colors=c("#4b61ba", "gray", "#a87963", "red"))
layout(p, title="MeSH-Enrichment Analysis (E)",
   xaxis=list(title="Term"),
   yaxis=list(title="-Log(P-value)"),
   legend=list(name=""))
```

```
## Warning: textfont.color doesn't (yet) support data arrays

## Warning: textfont.color doesn't (yet) support data arrays
```

## MeSH-Enrichment Analysis (F : Psychiatry and Psychology)

```
negLogPval <- -log10(out.vecLR[['pattern_4_4']]$Enrich$MeSH_F$Pvalue + 1E-10)
term <- out.vecLR[['pattern_4_4']]$Enrich$MeSH_F$Term
target <- seq_len(min(100, length(negLogPval)))
negLogPval <- negLogPval[target]
term <- term[target]
p <- plot_ly(x=seq_along(negLogPval), y=~negLogPval,
type="bar", color=~negLogPval, text=term,
colors=c("#4b61ba", "gray", "#a87963", "red"))
layout(p, title="MeSH-Enrichment Analysis (F)",
   xaxis=list(title="Term"),
   yaxis=list(title="-Log(P-value)"),
   legend=list(name=""))
```

```
## Warning: textfont.color doesn't (yet) support data arrays

## Warning: textfont.color doesn't (yet) support data arrays
```

## MeSH-Enrichment Analysis (G : Phenomena and Processes)

```
negLogPval <- -log10(out.vecLR[['pattern_4_4']]$Enrich$MeSH_G$Pvalue + 1E-10)
term <- out.vecLR[['pattern_4_4']]$Enrich$MeSH_G$Term
target <- seq_len(min(100, length(negLogPval)))
negLogPval <- negLogPval[target]
term <- term[target]
p <- plot_ly(x=seq_along(negLogPval), y=~negLogPval,
type="bar", color=~negLogPval, text=term,
colors=c("#4b61ba", "gray", "#a87963", "red"))
layout(p, title="MeSH-Enrichment Analysis (G)",
   xaxis=list(title="Term"),
   yaxis=list(title="-Log(P-value)"),
   legend=list(name=""))
```

```
## Warning: textfont.color doesn't (yet) support data arrays

## Warning: textfont.color doesn't (yet) support data arrays
```

## MeSH-Enrichment Analysis (H : Disciplines and Occupations)

```
negLogPval <- -log10(out.vecLR[['pattern_4_4']]$Enrich$MeSH_H$Pvalue + 1E-10)
term <- out.vecLR[['pattern_4_4']]$Enrich$MeSH_H$Term
target <- seq_len(min(100, length(negLogPval)))
negLogPval <- negLogPval[target]
term <- term[target]
p <- plot_ly(x=seq_along(negLogPval), y=~negLogPval,
type="bar", color=~negLogPval, text=term,
colors=c("#4b61ba", "gray", "#a87963", "red"))
layout(p, title="MeSH-Enrichment Analysis (H)",
   xaxis=list(title="Term"),
   yaxis=list(title="-Log(P-value)"),
   legend=list(name=""))
```

```
## Warning: textfont.color doesn't (yet) support data arrays

## Warning: textfont.color doesn't (yet) support data arrays
```

## MeSH-Enrichment Analysis (I : Anthropology, Education, Sociology and Social Phenomena)

```
negLogPval <- -log10(out.vecLR[['pattern_4_4']]$Enrich$MeSH_I$Pvalue + 1E-10)
term <- out.vecLR[['pattern_4_4']]$Enrich$MeSH_I$Term
target <- seq_len(min(100, length(negLogPval)))
negLogPval <- negLogPval[target]
term <- term[target]
p <- plot_ly(x=seq_along(negLogPval), y=~negLogPval,
type="bar", color=~negLogPval, text=term,
colors=c("#4b61ba", "gray", "#a87963", "red"))
layout(p, title="MeSH-Enrichment Analysis (I)",
   xaxis=list(title="Term"),
   yaxis=list(title="-Log(P-value)"),
   legend=list(name=""))
```

## MeSH-Enrichment Analysis (J : Technology and Food and Beverages)

```
negLogPval <- -log10(out.vecLR[['pattern_4_4']]$Enrich$MeSH_J$Pvalue + 1E-10)
term <- out.vecLR[['pattern_4_4']]$Enrich$MeSH_J$Term
target <- seq_len(min(100, length(negLogPval)))
negLogPval <- negLogPval[target]
term <- term[target]
p <- plot_ly(x=seq_along(negLogPval), y=~negLogPval,
type="bar", color=~negLogPval, text=term,
colors=c("#4b61ba", "gray", "#a87963", "red"))
layout(p, title="MeSH-Enrichment Analysis (J)",
   xaxis=list(title="Term"),
   yaxis=list(title="-Log(P-value)"),
   legend=list(name=""))
```

```
## Warning: textfont.color doesn't (yet) support data arrays

## Warning: textfont.color doesn't (yet) support data arrays
```

## MeSH-Enrichment Analysis (L : Information Science)

```
negLogPval <- -log10(out.vecLR[['pattern_4_4']]$Enrich$MeSH_L$Pvalue + 1E-10)
term <- out.vecLR[['pattern_4_4']]$Enrich$MeSH_L$Term
target <- seq_len(min(100, length(negLogPval)))
negLogPval <- negLogPval[target]
term <- term[target]
p <- plot_ly(x=seq_along(negLogPval), y=~negLogPval,
type="bar", color=~negLogPval, text=term,
colors=c("#4b61ba", "gray", "#a87963", "red"))
layout(p, title="MeSH-Enrichment Analysis (L)",
   xaxis=list(title="Term"),
   yaxis=list(title="-Log(P-value)"),
   legend=list(name=""))
```

```
## Warning: textfont.color doesn't (yet) support data arrays

## Warning: textfont.color doesn't (yet) support data arrays
```

## MeSH-Enrichment Analysis (M : Persons)

```
negLogPval <- -log10(out.vecLR[['pattern_4_4']]$Enrich$MeSH_M$Pvalue + 1E-10)
term <- out.vecLR[['pattern_4_4']]$Enrich$MeSH_M$Term
target <- seq_len(min(100, length(negLogPval)))
negLogPval <- negLogPval[target]
term <- term[target]
p <- plot_ly(x=seq_along(negLogPval), y=~negLogPval,
type="bar", color=~negLogPval, text=term,
colors=c("#4b61ba", "gray", "#a87963", "red"))
layout(p, title="MeSH-Enrichment Analysis (M)",
   xaxis=list(title="Term"),
   yaxis=list(title="-Log(P-value)"),
   legend=list(name=""))
```

```
## Warning: textfont.color doesn't (yet) support data arrays

## Warning: textfont.color doesn't (yet) support data arrays
```

## MeSH-Enrichment Analysis (N : Health Care)

```
negLogPval <- -log10(out.vecLR[['pattern_4_4']]$Enrich$MeSH_N$Pvalue + 1E-10)
term <- out.vecLR[['pattern_4_4']]$Enrich$MeSH_N$Term
target <- seq_len(min(100, length(negLogPval)))
negLogPval <- negLogPval[target]
term <- term[target]
p <- plot_ly(x=seq_along(negLogPval), y=~negLogPval,
type="bar", color=~negLogPval, text=term,
colors=c("#4b61ba", "gray", "#a87963", "red"))
layout(p, title="MeSH-Enrichment Analysis (N)",
   xaxis=list(title="Term"),
   yaxis=list(title="-Log(P-value)"),
   legend=list(name=""))
```

```
## Warning: textfont.color doesn't (yet) support data arrays

## Warning: textfont.color doesn't (yet) support data arrays
```

## MeSH-Enrichment Analysis (Z : Geographical Locations)

```
negLogPval <- -log10(out.vecLR[['pattern_4_4']]$Enrich$MeSH_Z$Pvalue + 1E-10)
term <- out.vecLR[['pattern_4_4']]$Enrich$MeSH_Z$Term
target <- seq_len(min(100, length(negLogPval)))
negLogPval <- negLogPval[target]
term <- term[target]
p <- plot_ly(x=seq_along(negLogPval), y=~negLogPval,
type="bar", color=~negLogPval, text=term,
colors=c("#4b61ba", "gray", "#a87963", "red"))
layout(p, title="MeSH-Enrichment Analysis (Z)",
   xaxis=list(title="Term"),
   yaxis=list(title="-Log(P-value)"),
   legend=list(name=""))
```

## Reactome Pathway-Enrichment Analysis

```
negLogPval <- -log10(out.vecLR[['pattern_4_4']]$Enrich$Reactome$Pvalue + 1E-10)
term <- out.vecLR[['pattern_4_4']]$Enrich$Reactome$Term
target <- seq_len(min(100, length(negLogPval)))
negLogPval <- negLogPval[target]
term <- term[target]
p <- plot_ly(x=seq_along(negLogPval), y=~negLogPval,
type="bar", color=~negLogPval, text=term,
colors=c("#4b61ba", "gray", "#a87963", "red"))
layout(p, title="Reactome-Enrichment Analysis",
   xaxis=list(title="Term"),
   yaxis=list(title="-Log(P-value)"),
   legend=list(name=""))
```

```
## Warning: textfont.color doesn't (yet) support data arrays

## Warning: textfont.color doesn't (yet) support data arrays
```

## DO-Enrichment Analysis

```
negLogPval <- -log10(out.vecLR[['pattern_4_4']]$Enrich$DO$Pvalue + 1E-10)
term <- out.vecLR[['pattern_4_4']]$Enrich$DO$Term
target <- seq_len(min(100, length(negLogPval)))
negLogPval <- negLogPval[target]
term <- term[target]
p <- plot_ly(x=seq_along(negLogPval), y=~negLogPval,
type="bar", color=~negLogPval, text=term,
colors=c("#4b61ba", "gray", "#a87963", "red"))
layout(p, title="DO-Enrichment Analysis",
   xaxis=list(title="Term"),
   yaxis=list(title="-Log(P-value)"),
   legend=list(name=""))
```

```
## Warning: textfont.color doesn't (yet) support data arrays

## Warning: textfont.color doesn't (yet) support data arrays
```

## NCG-Enrichment Analysis

```
negLogPval <- -log10(out.vecLR[['pattern_4_4']]$Enrich$NCG$Pvalue + 1E-10)
term <- out.vecLR[['pattern_4_4']]$Enrich$NCG$Term
target <- seq_len(min(100, length(negLogPval)))
negLogPval <- negLogPval[target]
term <- term[target]
p <- plot_ly(x=seq_along(negLogPval), y=~negLogPval,
type="bar", color=~negLogPval, text=term,
colors=c("#4b61ba", "gray", "#a87963", "red"))
layout(p, title="NCG-Enrichment Analysis",
   xaxis=list(title="Term"),
   yaxis=list(title="-Log(P-value)"),
   legend=list(name=""))
```

```
## Warning: textfont.color doesn't (yet) support data arrays

## Warning: textfont.color doesn't (yet) support data arrays
```

## DGN-Enrichment Analysis

```
negLogPval <- -log10(out.vecLR[['pattern_4_4']]$Enrich$DGN$Pvalue + 1E-10)
term <- out.vecLR[['pattern_4_4']]$Enrich$DGN$Term
target <- seq_len(min(100, length(negLogPval)))
negLogPval <- negLogPval[target]
term <- term[target]
p <- plot_ly(x=seq_along(negLogPval), y=~negLogPval,
type="bar", color=~negLogPval, text=term,
colors=c("#4b61ba", "gray", "#a87963", "red"))
layout(p, title="DGN-Enrichment Analysis",
   xaxis=list(title="Term"),
   yaxis=list(title="-Log(P-value)"),
   legend=list(name=""))
```

```
## Warning: textfont.color doesn't (yet) support data arrays

## Warning: textfont.color doesn't (yet) support data arrays
```
