# Supplementary material for: Sctensor detects many-to-many cell–cell interactions from single cell RNA-sequencing data
Source: BMC Bioinformatics. 2023 Nov 7;24:420. doi: 10.1186/s12859-023-05490-y (PMC10631077; doi:10.1186/s12859-023-05490-y)
Supplement: Supplementary file 15 — Additional file 15. HTML report of GermlineFemale. [file 12859_2023_5490_MOESM15_ESM.zip › output/report/Human_Germline_Female/ligand.html]

Details of Ligand Gene-centric Overview (selected)


# Details of Ligand Gene-centric Overview (selected)

```
library("scTensor")
load("reanalysis.RData")
scTensor:::.myvisNetwork(g)
```

| Rank | No. of related pairs | Ligand Gene | Receptor Genes | Related CCIs |
| --- | --- | --- | --- | --- |
| 1 | 22 | FN1 | ITGA5  ITGA8  ITGA2 | See the details of (3,1,)-related L-R patterns See the details of (2,1,)-related L-R patterns See the details of (4,1,)-related L-R patterns See the details of (3,2,)-related L-R patterns See the details of (2,2,)-related L-R patterns See the details of (1,2,)-related L-R patterns See the details of (2,4,)-related L-R patterns See the details of (3,3,)-related L-R patterns See the details of (3,4,)-related L-R patterns See the details of (4,2,)-related L-R patterns See the details of (2,3,)-related L-R patterns See the details of (1,3,)-related L-R patterns |
| 2 | 21 | ADM | RAMP2  CALCRL  RAMP1 | See the details of (3,1,)-related L-R patterns See the details of (1,1,)-related L-R patterns See the details of (2,1,)-related L-R patterns See the details of (4,1,)-related L-R patterns See the details of (3,2,)-related L-R patterns See the details of (2,2,)-related L-R patterns See the details of (1,2,)-related L-R patterns See the details of (2,4,)-related L-R patterns See the details of (3,3,)-related L-R patterns See the details of (3,4,)-related L-R patterns See the details of (1,4,)-related L-R patterns See the details of (5,1,)-related L-R patterns See the details of (2,3,)-related L-R patterns See the details of (1,3,)-related L-R patterns See the details of (5,4,)-related L-R patterns |
| 3 | 16 | APOE | VLDLR  LDLR  SORL1 | See the details of (2,2,)-related L-R patterns See the details of (3,3,)-related L-R patterns See the details of (3,4,)-related L-R patterns See the details of (5,2,)-related L-R patterns See the details of (5,1,)-related L-R patterns See the details of (5,3,)-related L-R patterns See the details of (2,3,)-related L-R patterns See the details of (1,3,)-related L-R patterns See the details of (5,4,)-related L-R patterns |
| 4 | 15 | VEGFA | KDR  NRP1 | See the details of (3,1,)-related L-R patterns See the details of (1,1,)-related L-R patterns See the details of (2,1,)-related L-R patterns See the details of (2,2,)-related L-R patterns See the details of (1,2,)-related L-R patterns See the details of (2,4,)-related L-R patterns See the details of (3,4,)-related L-R patterns See the details of (1,4,)-related L-R patterns |
| 6.5 | 10 | BMP2 | BMPR2 | See the details of (3,1,)-related L-R patterns See the details of (1,1,)-related L-R patterns See the details of (3,2,)-related L-R patterns See the details of (2,2,)-related L-R patterns See the details of (1,2,)-related L-R patterns See the details of (2,4,)-related L-R patterns See the details of (3,3,)-related L-R patterns See the details of (3,4,)-related L-R patterns See the details of (1,4,)-related L-R patterns See the details of (1,3,)-related L-R patterns |
| 6.5 | 10 | PDGFC | KDR  PDGFRA | See the details of (3,1,)-related L-R patterns See the details of (1,1,)-related L-R patterns See the details of (2,1,)-related L-R patterns See the details of (2,2,)-related L-R patterns See the details of (1,2,)-related L-R patterns See the details of (2,4,)-related L-R patterns See the details of (3,4,)-related L-R patterns See the details of (1,4,)-related L-R patterns |
| 6.5 | 10 | DLK1 | NOTCH3  NOTCH2 | See the details of (2,1,)-related L-R patterns See the details of (3,2,)-related L-R patterns See the details of (2,2,)-related L-R patterns See the details of (2,4,)-related L-R patterns See the details of (3,3,)-related L-R patterns See the details of (3,4,)-related L-R patterns See the details of (5,2,)-related L-R patterns See the details of (2,3,)-related L-R patterns See the details of (5,4,)-related L-R patterns |
| 6.5 | 10 | SLIT2 | ROBO1  GPC1  ROBO2 | See the details of (3,2,)-related L-R patterns See the details of (2,2,)-related L-R patterns See the details of (2,4,)-related L-R patterns See the details of (3,4,)-related L-R patterns |
| 9.5 | 8 | COL4A1 | ITGA1 | See the details of (3,1,)-related L-R patterns See the details of (1,1,)-related L-R patterns See the details of (3,2,)-related L-R patterns See the details of (2,2,)-related L-R patterns See the details of (1,2,)-related L-R patterns See the details of (3,4,)-related L-R patterns See the details of (5,2,)-related L-R patterns See the details of (5,1,)-related L-R patterns |
| 9.5 | 8 | SEMA6D | KDR | See the details of (1,1,)-related L-R patterns See the details of (2,1,)-related L-R patterns See the details of (4,1,)-related L-R patterns See the details of (2,2,)-related L-R patterns See the details of (1,2,)-related L-R patterns See the details of (2,4,)-related L-R patterns See the details of (3,4,)-related L-R patterns See the details of (1,4,)-related L-R patterns |
| 13 | 7 | COL18A1 | ITGA5  GPC1 | See the details of (2,1,)-related L-R patterns See the details of (4,1,)-related L-R patterns See the details of (2,2,)-related L-R patterns See the details of (2,4,)-related L-R patterns See the details of (2,3,)-related L-R patterns See the details of (1,3,)-related L-R patterns |
| 13 | 7 | JAG1 | NOTCH3  NOTCH2 | See the details of (4,1,)-related L-R patterns See the details of (3,3,)-related L-R patterns See the details of (3,4,)-related L-R patterns See the details of (4,2,)-related L-R patterns See the details of (4,4,)-related L-R patterns See the details of (4,3,)-related L-R patterns |
| 13 | 7 | EFNA1 | EPHA4 | See the details of (3,2,)-related L-R patterns See the details of (2,2,)-related L-R patterns See the details of (1,2,)-related L-R patterns See the details of (3,3,)-related L-R patterns See the details of (3,4,)-related L-R patterns See the details of (2,3,)-related L-R patterns See the details of (1,3,)-related L-R patterns |
| 13 | 7 | GAS6 | AXL | See the details of (2,2,)-related L-R patterns See the details of (1,2,)-related L-R patterns See the details of (2,4,)-related L-R patterns See the details of (3,4,)-related L-R patterns See the details of (1,4,)-related L-R patterns See the details of (5,2,)-related L-R patterns See the details of (5,4,)-related L-R patterns |
| 13 | 7 | CTF1 | IL6ST  LIFR | See the details of (2,2,)-related L-R patterns See the details of (2,4,)-related L-R patterns See the details of (3,4,)-related L-R patterns See the details of (2,3,)-related L-R patterns |
| 18 | 6 | NCAM1 | ROBO1  ROBO3 | See the details of (2,2,)-related L-R patterns See the details of (1,2,)-related L-R patterns See the details of (2,4,)-related L-R patterns See the details of (3,4,)-related L-R patterns |
| 18 | 6 | WNT5A | FZD7  FZD2 | See the details of (2,2,)-related L-R patterns See the details of (1,2,)-related L-R patterns See the details of (2,4,)-related L-R patterns See the details of (1,4,)-related L-R patterns |
| 18 | 6 | EFNA5 | EPHA4  EPHB2 | See the details of (2,2,)-related L-R patterns See the details of (2,4,)-related L-R patterns See the details of (3,3,)-related L-R patterns See the details of (3,4,)-related L-R patterns See the details of (2,3,)-related L-R patterns |
| 18 | 6 | RELN | VLDLR | See the details of (2,2,)-related L-R patterns See the details of (1,2,)-related L-R patterns See the details of (4,2,)-related L-R patterns See the details of (4,3,)-related L-R patterns See the details of (2,3,)-related L-R patterns See the details of (1,3,)-related L-R patterns |
| 18 | 6 | EFNB1 | EPHA4  EPHB2  EPHB6 | See the details of (2,2,)-related L-R patterns See the details of (2,4,)-related L-R patterns See the details of (3,4,)-related L-R patterns See the details of (2,3,)-related L-R patterns See the details of (1,3,)-related L-R patterns |
| 23 | 5 | INHA | TGFBR3 | See the details of (1,1,)-related L-R patterns See the details of (2,2,)-related L-R patterns See the details of (1,2,)-related L-R patterns See the details of (2,3,)-related L-R patterns See the details of (1,3,)-related L-R patterns |
| 23 | 5 | NPNT | ITGA8 | See the details of (1,1,)-related L-R patterns See the details of (2,1,)-related L-R patterns See the details of (4,1,)-related L-R patterns See the details of (2,4,)-related L-R patterns See the details of (5,1,)-related L-R patterns |
| 23 | 5 | COL1A1 | ITGA2 | See the details of (2,1,)-related L-R patterns See the details of (2,2,)-related L-R patterns See the details of (1,2,)-related L-R patterns See the details of (2,3,)-related L-R patterns See the details of (1,3,)-related L-R patterns |
| 23 | 5 | NPY | NPY1R | See the details of (2,2,)-related L-R patterns See the details of (1,2,)-related L-R patterns See the details of (2,4,)-related L-R patterns See the details of (3,4,)-related L-R patterns See the details of (1,4,)-related L-R patterns |
| 23 | 5 | CRLF1 | IL6ST  LIFR | See the details of (2,2,)-related L-R patterns See the details of (2,4,)-related L-R patterns See the details of (2,3,)-related L-R patterns |
| 26.5 | 4 | GDF9 | BMPR2 | See the details of (4,1,)-related L-R patterns See the details of (4,2,)-related L-R patterns See the details of (4,4,)-related L-R patterns See the details of (4,3,)-related L-R patterns |
| 26.5 | 4 | SEMA5A | MET | See the details of (2,2,)-related L-R patterns See the details of (1,2,)-related L-R patterns See the details of (4,2,)-related L-R patterns See the details of (1,3,)-related L-R patterns |
| 29.5 | 3 | SEMA3E | PLXND1 | See the details of (2,1,)-related L-R patterns See the details of (4,1,)-related L-R patterns See the details of (5,1,)-related L-R patterns |
| 29.5 | 3 | NRG1 | ERBB4 | See the details of (2,2,)-related L-R patterns See the details of (2,3,)-related L-R patterns See the details of (1,3,)-related L-R patterns |
| 29.5 | 3 | TGFB2 | TGFBR3 | See the details of (2,2,)-related L-R patterns See the details of (1,2,)-related L-R patterns See the details of (1,3,)-related L-R patterns |
| 29.5 | 3 | NRG4 | ERBB4 | See the details of (4,2,)-related L-R patterns See the details of (4,3,)-related L-R patterns See the details of (1,3,)-related L-R patterns |
| 34.5 | 2 | SEMA3C | NRP2 | See the details of (2,1,)-related L-R patterns See the details of (2,4,)-related L-R patterns |
| 34.5 | 2 | FGF14 | FGFR1 | See the details of (4,1,)-related L-R patterns See the details of (4,2,)-related L-R patterns |
| 34.5 | 2 | TFPI | VLDLR | See the details of (3,2,)-related L-R patterns See the details of (3,3,)-related L-R patterns |
| 34.5 | 2 | RSPO3 | LGR4 | See the details of (2,2,)-related L-R patterns See the details of (2,4,)-related L-R patterns |
| 34.5 | 2 | PYY | NPY1R | See the details of (2,4,)-related L-R patterns See the details of (5,4,)-related L-R patterns |
| 34.5 | 2 | NPFF | NPFFR2 | See the details of (2,4,)-related L-R patterns See the details of (5,4,)-related L-R patterns |
| 41 | 1 | F2 | F2RL2 | See the details of (4,1,)-related L-R patterns |
| 41 | 1 | MDK | PTPRZ1 | See the details of (2,2,)-related L-R patterns |
| 41 | 1 | PTN | PTPRZ1 | See the details of (2,2,)-related L-R patterns |
| 41 | 1 | RSPO1 | LGR4 | See the details of (2,2,)-related L-R patterns |
| 41 | 1 | LAMA2 | ITGA7 | See the details of (1,3,)-related L-R patterns |
| 41 | 1 | VTN | ITGA5 | See the details of (1,3,)-related L-R patterns |
| 41 | 1 | BTC | ERBB4 | See the details of (1,3,)-related L-R patterns |
