# Supplementary material for: Sctensor detects many-to-many cell–cell interactions from single cell RNA-sequencing data
Source: BMC Bioinformatics. 2023 Nov 7;24:420. doi: 10.1186/s12859-023-05490-y (PMC10631077; doi:10.1186/s12859-023-05490-y)
Supplement: Supplementary file 15 — Additional file 15. HTML report of GermlineFemale. [file 12859_2023_5490_MOESM15_ESM.zip › output/report/Human_Germline_Female/ligand_all.html]

Details of Ligand Gene-centric Overview (all)


# Details of Ligand Gene-centric Overview (all)

| Ligand Gene | Receptor Gene |
| --- | --- |
| ADAM12 | SDC4 |
| ADAM15 | ITGA5 |
| ADAM17 | ITGA5 |
| ADAM23 | ITGA5 |
| ADCYAP1 | ADCYAP1R1 VIPR1 VIPR2 |
| ADM | CALCR CALCRL MRGPRX2 RAMP1 RAMP2 RAMP3 GPR182 |
| ADM2 | CALCRL RAMP1 RAMP2 |
| AGT | AGTR1 |
| ALOX5AP | ALOX5 |
| AMH | AMHR2 |
| ANGPT1 | TIE1 |
| ANGPT2 | TEK |
| ANGPTL1 | TEK |
| ANXA1 | FPR1 FPR2 FPR3 |
| APELA | APLNR |
| APLN | APLNR |
| APOB | LDLR |
| APOE | LDLR LRP5 LRP8 SORL1 VLDLR |
| AREG | EGFR |
| ASIP | ATRN MC1R MGRN1 |
| AVP | AVPR1A |
| BDNF | NTRK2 |
| BMP10 | BMPR2 |
| BMP15 | BMPR2 |
| BMP2 | ACVR1 ACVR2A ACVR2B ACVRL1 BMPR1A BMPR1B BMPR2 |
| BMP3 | BMPR2 |
| BMP4 | BMPR2 |
| BMP5 | BMPR2 |
| BMP6 | BMPR2 |
| BMP7 | BMPR2 |
| BMP8A | BMPR2 |
| BMP8B | BMPR2 |
| BTC | EGFR ERBB4 ERBB3 |
| BTLA | TNFRSF14 |
| C3 | C3AR1 ITGAX ITGB2 LRP1 |
| CALCA | CALCRL RAMP1 RAMP2 |
| CALCB | CALCRL RAMP1 RAMP2 |
| CALM1 | MYLK |
| CALM2 | MYLK |
| CALM3 | MYLK |
| CALR | SCARF1 |
| CAMP | P2RX7 |
| CCK | CCKBR |
| CCL2 | CCR2 LOC105377067 CCR3 CCR5 CCR1 CCR4 |
| CCL21 | ACKR4 CCR7 |
| CCL25 | ACKR4 CCR9 |
| CCL26 | CCR2 LOC105377067 CCR3 |
| CCL27 | CCR10 |
| CCL28 | CCR10 |
| CCL5 | CCR1 LOC105377067 CCR3 CCR5 CXCR3 GPR75 |
| CD34 | SELL |
| CDH1 | ITGAE ITGB7 |
| CFH | SELL |
| CGA | FSHR LHCGR TSHR |
| CHAD | ITGA2 |
| CLCF1 | CRLF1 IL6ST LIFR |
| CNTF | IL6ST LIFR |
| COL18A1 | GPC1 ITGA5 ITGB1 |
| COL1A1 | DDR1 DDR2 ITGA11 ITGA2 ITGB1 |
| COL2A1 | ITGA2 |
| COL4A1 | ITGA1 ITGB1 ITGB8 |
| CORT | SSTR2 SSTR3 |
| CRH | MC2R |
| CRLF1 | CNTFR IL6ST LIFR |
| CSF1 | CSF1R |
| CSF2 | CSF1R |
| CSF3 | CSF1R |
| CTF1 | IL6ST LIFR |
| CXCL10 | LOC105377067 CCR3 CXCR3 |
| CXCL11 | ACKR3 LOC105377067 CCR3 CXCR3 |
| CXCL12 | ACKR3 CXCR3 CXCR4 |
| CXCL17 | GPR35 |
| CXCL2 | CXCR2 CXCR1 XCR1 |
| CXCL3 | CXCR2 CXCR1 |
| CXCL5 | CXCR2 CXCR1 |
| DHH | HHIP PTCH2 |
| DKK1 | KREMEN1 KREMEN2 LRP6 |
| DLK1 | NOTCH1 NOTCH2 NOTCH3 NOTCH4 |
| DLL1 | NOTCH2 NOTCH3 |
| DLL3 | NOTCH2 NOTCH3 |
| DLL4 | NOTCH2 NOTCH3 |
| DUSP18 | ITGA1 ITGA2 ITGA7 |
| EBI3 | IL6ST |
| EDN1 | EDNRA |
| EDN2 | EDNRA |
| EDN3 | EDNRB |
| EFNA1 | EPHA1 EPHA2 EPHA3 EPHA4 EPHA5 EPHA7 EPHA8 EPHA6 |
| EFNA2 | EPHA4 EPHA5 |
| EFNA3 | EPHA4 EPHA5 EPHB1 |
| EFNA4 | EPHA4 EPHA5 |
| EFNA5 | EPHA1 EPHA2 EPHA3 EPHA4 EPHA5 EPHA7 EPHA8 EPHA6 EPHB2 |
| EFNB1 | EPHA4 EPHB1 EPHB2 EPHB3 EPHB4 EPHB6 |
| EFNB2 | EPHA4 EPHB1 EPHB2 EPHB6 |
| EFNB3 | EPHA4 EPHB1 EPHB2 EPHB6 |
| EGF | EGFR |
| EPGN | EGFR |
| EREG | EGFR ERBB4 |
| F2 | F2R F2RL1 F2RL2 F2RL3 |
| F7 | F3 |
| FASLG | FAS TNFRSF6B |
| FGA | ITGB2 |
| FGF1 | FGFR1 |
| FGF10 | FGFR1 |
| FGF11 | FGFR1 |
| FGF12 | FGFR1 |
| FGF13 | FGFR1 |
| FGF14 | FGFR1 FGFR2 FGFR3 FGFR4 |
| FGF16 | FGFR1 |
| FGF17 | FGFR1 |
| FGF18 | FGFR1 |
| FGF19 | FGFR1 |
| FGF2 | FGFR1 SDC4 |
| FGF23 | FGFR1 |
| FGF3 | FGFR1 |
| FGF4 | FGFR1 |
| FGF5 | FGFR1 |
| FGF6 | FGFR1 |
| FGF7 | FGFR2 |
| FGF8 | FGFR1 |
| FGF9 | FGFR1 |
| FN1 | ITGA2 ITGA2B ITGA4 ITGA5 ITGA8 ITGB1 ITGB3 ITGB6 ITGB8 SDC2 |
| GAS6 | AXL MERTK TYRO3 |
| GAST | CCKBR |
| GDF1 | BMPR2 |
| GDF10 | BMPR2 |
| GDF15 | GFRAL |
| GDF2 | BMPR2 |
| GDF3 | BMPR2 |
| GDF5 | BMPR2 |
| GDF6 | BMPR2 |
| GDF7 | BMPR2 |
| GDF9 | ACVR1B ACVR1C ACVR2A ACVR2B BMPR1A BMPR1B BMPR2 TGFBR1 |
| GDNF | GFRA1 |
| GH1 | GHR |
| GH2 | GHR |
| GHRH | VIPR1 |
| GIP | GIPR |
| GNRH1 | GNRHR |
| GNRH2 | GNRHR |
| GPHA2 | TSHR |
| HAS2 | HMMR |
| HBEGF | EGFR ERBB4 |
| HDC | HRH1 |
| HGF | MET |
| HLA-A | KIR3DL2 |
| HLA-B | KIR3DL1 |
| HLA-C | KIR2DL1 KIR2DL3 KIR2DS4 |
| HLA-E | KLRC1 KLRC2 KLRD1 |
| HLA-G | LILRB1 |
| IAPP | RAMP1 RAMP2 |
| ICAM1 | ITGAL ITGAX ITGB2 |
| ICAM2 | ITGB2 |
| ICAM3 | ITGB2 |
| ICAM4 | ITGB2 |
| ICAM5 | ITGB2 |
| IFNA1 | IFNAR1 |
| IFNA10 | IFNAR1 |
| IFNA13 | IFNAR1 |
| IFNA14 | IFNAR1 |
| IFNA16 | IFNAR1 |
| IFNA17 | IFNAR1 |
| IFNA2 | IFNAR1 |
| IFNA21 | IFNAR1 |
| IFNA4 | IFNAR1 |
| IFNA5 | IFNAR1 |
| IFNA6 | IFNAR1 |
| IFNA7 | IFNAR1 |
| IFNA8 | IFNAR1 |
| IFNB1 | IFNAR1 |
| IFNK | IFNAR1 |
| IFNL1 | IL10RB |
| IFNL2 | IL10RB |
| IFNL3 | IL10RB |
| IFNW1 | IFNAR1 |
| IGF1 | IGF1R |
| IGF2 | INSR |
| IHH | HHIP PTCH1 |
| IL10 | IL10RB |
| IL11 | IL11RA IL6ST |
| IL13 | IL13RA2 IL2RG |
| IL15 | IL2RG |
| IL17B | IL17RA IL17RB |
| IL18 | IL18R1 |
| IL1A | IL1R1 IL1R2 IL1RAP |
| IL1B | IL1R1 IL1R2 IL1RAP |
| IL1R1 | IL1R2 |
| IL1RN | IL1R1 IL1RAP IL2RG |
| IL2 | IL2RG |
| IL21 | IL2RG |
| IL22 | IL10RB |
| IL27 | IL6ST |
| IL31 | OSMR |
| IL33 | IL1RAP IL1RL1 |
| IL34 | CSF1R |
| IL36A | IL1RAP |
| IL36B | IL1RAP |
| IL36G | IL1RAP |
| IL36RN | IL1RAP |
| IL37 | IL18R1 |
| IL4 | IL2RG |
| IL6 | IL6ST |
| IL7 | IL2RG IL7R |
| IL9 | IL2RG |
| INHA | ACVR1B ACVR1C ACVR2A ACVR2B ACVR1 TGFBR3 |
| INHBA | ACVR1B ACVR1C ACVR2A ACVR2B ACVR1 BAMBI |
| INHBB | ACVR1B ACVR1C ACVR2A ACVR2B ACVR1 |
| INS | INSR |
| INSL5 | RXFP3 RXFP4 |
| JAG1 | NOTCH1 NOTCH2 NOTCH3 NOTCH4 |
| JAG2 | NOTCH2 NOTCH3 |
| KITLG | KIT |
| LAMA1 | ITGA7 |
| LAMA2 | ITGA7 ITGB1 RPSA |
| LAMB1 | ITGA6 ITGB1 |
| LAMB2 | RPSA |
| LIF | IL6ST LIFR |
| LOC101927057 | LINGO1 TNFRSF1B |
| LTA | LTBR TNFRSF1B |
| LTB | LTBR CD40 |
| MADCAM1 | ITGA4 ITGB7 |
| MDK | PTPRB PTPRZ1 ALK |
| MST1 | MST1R |
| NAMPT | INSR |
| NCAM1 | ROBO1 ROBO3 |
| NDP | LGR4 |
| NGF | NGFR NTRK1 SORT1 |
| NID1 | PTPRF |
| NLGN1 | NRXN3 |
| NLGN2 | NRXN3 |
| NLGN3 | NRXN3 |
| NMB | GRPR NMBR |
| NMU | NMUR1 NMUR2 |
| NPFF | NPFFR1 NPFFR2 |
| NPNT | ITGA8 ITGB1 |
| NPPA | NPR3 |
| NPPB | NPR3 |
| NPVF | NPFFR1 NPFFR2 |
| NPY | NPY1R NPY2R NPY4R NPY5R PRLHR |
| NRG1 | ERBB3 ERBB4 ERBB2 |
| NRG2 | ERBB4 |
| NRG3 | ERBB4 |
| NRG4 | ERBB4 |
| NRTN | GFRA1 GFRA2 RET |
| NTF3 | NTRK2 |
| NTF4 | NTRK2 |
| NTN1 | DCC UNC5C |
| NTNG1 | LRRC4C |
| NTS | NTSR1 NTSR2 |
| NXPH1 | NRXN3 |
| NXPH3 | NRXN3 |
| OMG | LINGO1 TNFRSF1B |
| OSM | IL6ST LIFR OSMR |
| OSTN | NPR3 |
| OXT | AVPR1A |
| PDGFA | PDGFRA |
| PDGFB | PDGFRA PDGFRB |
| PDGFC | FLT4 KDR PDGFRA |
| PDGFD | PDGFRB |
| PENK | MRGPRX1 OPRD1 OPRM1 OGFR |
| PF4 | LDLR PROCR THBD |
| PGF | NRP1 |
| PLAT | LRP1 |
| PNOC | OPRL1 |
| POMC | MC2R |
| PPBP | CXCR2 CXCR1 |
| PROC | PROCR |
| PROK1 | PROKR1 PROKR2 |
| PROS1 | AXL |
| PSAP | GPR37L1 |
| PTN | PTPRB PTPRZ1 |
| PYY | NPY1R NPY2R NPY4R NPY5R |
| QRFP | P2RY14 QRFPR |
| RARRES2 | CMKLR1 |
| RELN | ITGA3 ITGB1 LRP8 VLDLR |
| REN | ATP6AP2 |
| RSPO1 | LGR4 LGR5 LGR6 LRP6 |
| RSPO2 | LGR4 LGR5 LGR6 |
| RSPO3 | LGR4 LGR5 LGR6 |
| RSPO4 | LGR4 |
| RTN4 | LINGO1 RTN4R |
| SCT | VIPR1 |
| SELPLG | SELE |
| SEMA3A | NRP1 PLXNA4 |
| SEMA3B | NRP1 |
| SEMA3C | NRP2 |
| SEMA3E | PLXND1 |
| SEMA3F | NRP2 PLXNA3 |
| SEMA4D | MET PLXNB1 PLXNB2 |
| SEMA5A | MET PLXNB3 |
| SEMA6D | KDR PLXNA1 |
| SERPINA1 | LRP1 |
| SERPINE2 | LRP1 |
| SHH | HHIP PTCH1 |
| SLIT1 | GPC1 ROBO1 |
| SLIT2 | GPC1 ROBO1 ROBO2 ROBO4 SDC1 |
| SOST | LRP5 LRP6 |
| SST | MRGPRX2 SSTR1 SSTR2 SSTR3 SSTR4 SSTR5 |
| TAC1 | MRGPRX2 TACR1 TACR2 TACR3 |
| TAC3 | TACR1 |
| TAC4 | TACR1 |
| TDGF1 | ACVR2A |
| TF | TFRC |
| TFPI | LRP1 VLDLR |
| TGFA | EGFR |
| TGFB1 | TGFBR3 |
| TGFB2 | TGFBR1 TGFBR2 TGFBR3 |
| TGFB3 | TGFBR3 |
| THBS1 | CD36 CD47 SDC1 |
| TNF | LTBR TNFRSF1B |
| TNFSF10 | TNFRSF10A |
| TNFSF12 | TNFRSF12A |
| TNFSF13B | TNFRSF13B TNFRSF13C TNFRSF17 |
| TNFSF14 | LTBR |
| TNFSF8 | TNFRSF8 |
| TPH1 | HTR1D HTR1E HTR2B HTR2C |
| TSLP | IL7R |
| VCAM1 | ITGA4 ITGAD ITGB1 ITGB2 |
| VEGFA | FLT1 KDR NRP1 |
| VEGFB | NRP1 |
| VEGFC | KDR NRP2 |
| VIP | VIPR1 |
| VTN | ITGA5 ITGB3 |
| WNT11 | FZD7 |
| WNT4 | FZD2 |
| WNT5A | FZD6 FZD2 FZD4 FZD7 RYK |
| ZP3 | MERTK |
