# Supplementary material for: Sctensor detects many-to-many cell–cell interactions from single cell RNA-sequencing data
Source: BMC Bioinformatics. 2023 Nov 7;24:420. doi: 10.1186/s12859-023-05490-y (PMC10631077; doi:10.1186/s12859-023-05490-y)
Supplement: Supplementary file 15 — Additional file 15. HTML report of GermlineFemale. [file 12859_2023_5490_MOESM15_ESM.zip › output/report/Human_Germline_Female/receptor.html]

Details of Receptor Gene-centric Overview (selected)


# Details of Receptor Gene-centric Overview (selected)

```
library("scTensor")
load("reanalysis.RData")
scTensor:::.myvisNetwork(g)
```

| Rank | No. of related pairs | Receptor Gene | Ligand Genes | Related CCIs |
| --- | --- | --- | --- | --- |
| 1 | 23 | KDR | PDGFC  VEGFA  SEMA6D | See the details of (3,1,)-related L-R patterns See the details of (1,1,)-related L-R patterns See the details of (2,1,)-related L-R patterns See the details of (4,1,)-related L-R patterns See the details of (2,2,)-related L-R patterns See the details of (1,2,)-related L-R patterns See the details of (2,4,)-related L-R patterns See the details of (3,4,)-related L-R patterns See the details of (1,4,)-related L-R patterns |
| 2 | 15 | ITGA2 | FN1  COL1A1 | See the details of (3,1,)-related L-R patterns See the details of (2,1,)-related L-R patterns See the details of (4,1,)-related L-R patterns See the details of (3,2,)-related L-R patterns See the details of (2,2,)-related L-R patterns See the details of (1,2,)-related L-R patterns See the details of (3,3,)-related L-R patterns See the details of (4,2,)-related L-R patterns See the details of (2,3,)-related L-R patterns See the details of (1,3,)-related L-R patterns |
| 4.5 | 14 | ITGA5 | FN1  COL18A1  VTN | See the details of (3,1,)-related L-R patterns See the details of (2,1,)-related L-R patterns See the details of (4,1,)-related L-R patterns See the details of (2,4,)-related L-R patterns See the details of (3,3,)-related L-R patterns See the details of (3,4,)-related L-R patterns See the details of (2,3,)-related L-R patterns See the details of (1,3,)-related L-R patterns |
| 4.5 | 14 | BMPR2 | BMP2  GDF9 | See the details of (3,1,)-related L-R patterns See the details of (1,1,)-related L-R patterns See the details of (4,1,)-related L-R patterns See the details of (3,2,)-related L-R patterns See the details of (2,2,)-related L-R patterns See the details of (1,2,)-related L-R patterns See the details of (2,4,)-related L-R patterns See the details of (3,3,)-related L-R patterns See the details of (3,4,)-related L-R patterns See the details of (4,2,)-related L-R patterns See the details of (1,4,)-related L-R patterns See the details of (4,4,)-related L-R patterns See the details of (4,3,)-related L-R patterns See the details of (1,3,)-related L-R patterns |
| 4.5 | 14 | NOTCH2 | JAG1  DLK1 | See the details of (4,1,)-related L-R patterns See the details of (3,2,)-related L-R patterns See the details of (2,2,)-related L-R patterns See the details of (2,4,)-related L-R patterns See the details of (3,3,)-related L-R patterns See the details of (3,4,)-related L-R patterns See the details of (4,2,)-related L-R patterns See the details of (4,4,)-related L-R patterns See the details of (5,2,)-related L-R patterns See the details of (4,3,)-related L-R patterns See the details of (2,3,)-related L-R patterns See the details of (5,4,)-related L-R patterns |
| 4.5 | 14 | VLDLR | TFPI  RELN  APOE | See the details of (3,2,)-related L-R patterns See the details of (2,2,)-related L-R patterns See the details of (1,2,)-related L-R patterns See the details of (3,3,)-related L-R patterns See the details of (4,2,)-related L-R patterns See the details of (5,2,)-related L-R patterns See the details of (5,3,)-related L-R patterns See the details of (4,3,)-related L-R patterns See the details of (2,3,)-related L-R patterns See the details of (1,3,)-related L-R patterns |
| 7 | 13 | EPHA4 | EFNA1  EFNA5  EFNB1 | See the details of (3,2,)-related L-R patterns See the details of (2,2,)-related L-R patterns See the details of (1,2,)-related L-R patterns See the details of (3,3,)-related L-R patterns See the details of (3,4,)-related L-R patterns See the details of (2,3,)-related L-R patterns See the details of (1,3,)-related L-R patterns |
| 8 | 12 | RAMP2 | ADM | See the details of (3,1,)-related L-R patterns See the details of (1,1,)-related L-R patterns See the details of (2,1,)-related L-R patterns See the details of (4,1,)-related L-R patterns See the details of (2,4,)-related L-R patterns See the details of (3,3,)-related L-R patterns See the details of (3,4,)-related L-R patterns See the details of (1,4,)-related L-R patterns See the details of (5,1,)-related L-R patterns See the details of (2,3,)-related L-R patterns See the details of (1,3,)-related L-R patterns See the details of (5,4,)-related L-R patterns |
| 9 | 9 | ITGA8 | FN1  NPNT | See the details of (3,1,)-related L-R patterns See the details of (1,1,)-related L-R patterns See the details of (2,1,)-related L-R patterns See the details of (4,1,)-related L-R patterns See the details of (2,4,)-related L-R patterns See the details of (3,4,)-related L-R patterns See the details of (5,1,)-related L-R patterns |
| 11 | 8 | ITGA1 | COL4A1 | See the details of (3,1,)-related L-R patterns See the details of (1,1,)-related L-R patterns See the details of (3,2,)-related L-R patterns See the details of (2,2,)-related L-R patterns See the details of (1,2,)-related L-R patterns See the details of (3,4,)-related L-R patterns See the details of (5,2,)-related L-R patterns See the details of (5,1,)-related L-R patterns |
| 11 | 8 | TGFBR3 | INHA  TGFB2 | See the details of (1,1,)-related L-R patterns See the details of (2,2,)-related L-R patterns See the details of (1,2,)-related L-R patterns See the details of (2,3,)-related L-R patterns See the details of (1,3,)-related L-R patterns |
| 11 | 8 | ROBO1 | SLIT2  NCAM1 | See the details of (3,2,)-related L-R patterns See the details of (2,2,)-related L-R patterns See the details of (1,2,)-related L-R patterns See the details of (2,4,)-related L-R patterns See the details of (3,4,)-related L-R patterns |
| 15 | 7 | NRP1 | VEGFA | See the details of (1,1,)-related L-R patterns See the details of (2,1,)-related L-R patterns See the details of (2,2,)-related L-R patterns See the details of (1,2,)-related L-R patterns See the details of (2,4,)-related L-R patterns See the details of (3,4,)-related L-R patterns See the details of (1,4,)-related L-R patterns |
| 15 | 7 | AXL | GAS6 | See the details of (2,2,)-related L-R patterns See the details of (1,2,)-related L-R patterns See the details of (2,4,)-related L-R patterns See the details of (3,4,)-related L-R patterns See the details of (1,4,)-related L-R patterns See the details of (5,2,)-related L-R patterns See the details of (5,4,)-related L-R patterns |
| 15 | 7 | IL6ST | CTF1  CRLF1 | See the details of (2,2,)-related L-R patterns See the details of (2,4,)-related L-R patterns See the details of (3,4,)-related L-R patterns See the details of (2,3,)-related L-R patterns |
| 15 | 7 | NPY1R | NPY  PYY | See the details of (2,2,)-related L-R patterns See the details of (1,2,)-related L-R patterns See the details of (2,4,)-related L-R patterns See the details of (3,4,)-related L-R patterns See the details of (1,4,)-related L-R patterns See the details of (5,4,)-related L-R patterns |
| 15 | 7 | ERBB4 | NRG1  NRG4  BTC | See the details of (2,2,)-related L-R patterns See the details of (4,2,)-related L-R patterns See the details of (4,3,)-related L-R patterns See the details of (2,3,)-related L-R patterns See the details of (1,3,)-related L-R patterns |
| 18 | 6 | LDLR | APOE | See the details of (3,4,)-related L-R patterns See the details of (5,2,)-related L-R patterns See the details of (5,1,)-related L-R patterns See the details of (5,3,)-related L-R patterns See the details of (1,3,)-related L-R patterns See the details of (5,4,)-related L-R patterns |
| 20 | 5 | CALCRL | ADM | See the details of (3,1,)-related L-R patterns See the details of (3,2,)-related L-R patterns See the details of (2,2,)-related L-R patterns See the details of (1,2,)-related L-R patterns See the details of (5,1,)-related L-R patterns |
| 20 | 5 | GPC1 | COL18A1  SLIT2 | See the details of (2,2,)-related L-R patterns See the details of (2,4,)-related L-R patterns See the details of (3,4,)-related L-R patterns |
| 20 | 5 | LIFR | CTF1  CRLF1 | See the details of (2,2,)-related L-R patterns See the details of (2,4,)-related L-R patterns See the details of (2,3,)-related L-R patterns |
| 24 | 4 | MET | SEMA5A | See the details of (2,2,)-related L-R patterns See the details of (1,2,)-related L-R patterns See the details of (4,2,)-related L-R patterns See the details of (1,3,)-related L-R patterns |
| 24 | 4 | FZD7 | WNT5A | See the details of (2,2,)-related L-R patterns See the details of (1,2,)-related L-R patterns See the details of (2,4,)-related L-R patterns See the details of (1,4,)-related L-R patterns |
| 24 | 4 | EPHB2 | EFNA5  EFNB1 | See the details of (2,2,)-related L-R patterns See the details of (2,4,)-related L-R patterns See the details of (3,4,)-related L-R patterns |
| 24 | 4 | RAMP1 | ADM | See the details of (3,3,)-related L-R patterns See the details of (3,4,)-related L-R patterns See the details of (2,3,)-related L-R patterns See the details of (1,3,)-related L-R patterns |
| 24 | 4 | SORL1 | APOE | See the details of (5,2,)-related L-R patterns See the details of (5,3,)-related L-R patterns See the details of (1,3,)-related L-R patterns See the details of (5,4,)-related L-R patterns |
| 29 | 3 | NOTCH3 | DLK1  JAG1 | See the details of (2,1,)-related L-R patterns See the details of (4,1,)-related L-R patterns See the details of (2,4,)-related L-R patterns |
| 29 | 3 | PLXND1 | SEMA3E | See the details of (2,1,)-related L-R patterns See the details of (4,1,)-related L-R patterns See the details of (5,1,)-related L-R patterns |
| 29 | 3 | LGR4 | RSPO3  RSPO1 | See the details of (2,2,)-related L-R patterns See the details of (2,4,)-related L-R patterns |
| 29 | 3 | ROBO2 | SLIT2 | See the details of (2,2,)-related L-R patterns See the details of (2,4,)-related L-R patterns See the details of (3,4,)-related L-R patterns |
| 29 | 3 | PDGFRA | PDGFC | See the details of (2,4,)-related L-R patterns See the details of (3,4,)-related L-R patterns See the details of (1,4,)-related L-R patterns |
| 35 | 2 | NRP2 | SEMA3C | See the details of (2,1,)-related L-R patterns See the details of (2,4,)-related L-R patterns |
| 35 | 2 | FGFR1 | FGF14 | See the details of (4,1,)-related L-R patterns See the details of (4,2,)-related L-R patterns |
| 35 | 2 | PTPRZ1 | MDK  PTN | See the details of (2,2,)-related L-R patterns |
| 35 | 2 | ROBO3 | NCAM1 | See the details of (2,2,)-related L-R patterns See the details of (2,4,)-related L-R patterns |
| 35 | 2 | FZD2 | WNT5A | See the details of (2,4,)-related L-R patterns See the details of (1,4,)-related L-R patterns |
| 35 | 2 | EPHB6 | EFNB1 | See the details of (2,4,)-related L-R patterns See the details of (3,4,)-related L-R patterns |
| 35 | 2 | NPFFR2 | NPFF | See the details of (2,4,)-related L-R patterns See the details of (5,4,)-related L-R patterns |
| 39.5 | 1 | F2RL2 | F2 | See the details of (4,1,)-related L-R patterns |
| 39.5 | 1 | ITGA7 | LAMA2 | See the details of (1,3,)-related L-R patterns |
