# Supplementary material for: Sctensor detects many-to-many cell–cell interactions from single cell RNA-sequencing data
Source: BMC Bioinformatics. 2023 Nov 7;24:420. doi: 10.1186/s12859-023-05490-y (PMC10631077; doi:10.1186/s12859-023-05490-y)
Supplement: Supplementary file 15 — Additional file 15. HTML report of GermlineFemale. [file 12859_2023_5490_MOESM15_ESM.zip › output/report/Human_Germline_Female/receptor_all.html]

Details of Receptor Gene-centric Overview (all)


# Details of Receptor Gene-centric Overview (all)

| Receptor Gene | Ligand Gene |
| --- | --- |
| ACKR3 | CXCL11 CXCL12 |
| ACKR4 | CCL21 CCL25 |
| ACVR1 | BMP2 INHA INHBA INHBB |
| ACVR1B | GDF9 INHA INHBA INHBB |
| ACVR1C | GDF9 INHA INHBA INHBB |
| ACVR2A | BMP2 GDF9 INHA INHBA INHBB TDGF1 |
| ACVR2B | BMP2 GDF9 INHA INHBA INHBB |
| ACVRL1 | BMP2 |
| ADCYAP1R1 | ADCYAP1 |
| AGTR1 | AGT |
| ALK | MDK |
| ALOX5 | ALOX5AP |
| AMHR2 | AMH |
| APLNR | APELA APLN |
| ATP6AP2 | REN |
| ATRN | ASIP |
| AVPR1A | AVP OXT |
| AXL | GAS6 PROS1 |
| BAMBI | INHBA |
| BMPR1A | BMP2 GDF9 |
| BMPR1B | BMP2 GDF9 |
| BMPR2 | BMP2 GDF9 GDF1 BMP6 BMP5 GDF2 GDF3 BMP8A BMP8B GDF10 BMP7 BMP10 BMP4 GDF7 BMP3 BMP15 GDF6 GDF5 |
| C3AR1 | C3 |
| CALCR | ADM |
| CALCRL | ADM CALCA ADM2 CALCB |
| CCKBR | GAST CCK |
| CCR1 | CCL2 CCL5 |
| CCR10 | CCL27 CCL28 |
| CCR2 | CCL2 CCL26 |
| CCR3 | CCL2 CCL26 CCL5 CXCL10 CXCL11 |
| CCR4 | CCL2 |
| CCR5 | CCL2 CCL5 |
| CCR7 | CCL21 |
| CCR9 | CCL25 |
| CD36 | THBS1 |
| CD40 | LTB |
| CD47 | THBS1 |
| CMKLR1 | RARRES2 |
| CNTFR | CRLF1 |
| CRLF1 | CLCF1 |
| CSF1R | CSF2 CSF3 CSF1 IL34 |
| CXCR1 | CXCL2 CXCL3 CXCL5 PPBP |
| CXCR2 | CXCL2 CXCL3 CXCL5 PPBP |
| CXCR3 | CCL5 CXCL10 CXCL11 CXCL12 |
| CXCR4 | CXCL12 |
| DCC | NTN1 |
| DDR1 | COL1A1 |
| DDR2 | COL1A1 |
| EDNRA | EDN1 EDN2 |
| EDNRB | EDN3 |
| EGFR | BTC TGFA AREG EGF HBEGF EPGN EREG |
| EPHA1 | EFNA1 EFNA5 |
| EPHA2 | EFNA1 EFNA5 |
| EPHA3 | EFNA1 EFNA5 |
| EPHA4 | EFNA1 EFNA5 EFNB1 EFNA4 EFNB3 EFNA2 EFNB2 EFNA3 |
| EPHA5 | EFNA1 EFNA5 EFNA2 EFNA3 EFNA4 |
| EPHA6 | EFNA1 EFNA5 |
| EPHA7 | EFNA1 EFNA5 |
| EPHA8 | EFNA1 EFNA5 |
| EPHB1 | EFNB1 EFNB3 EFNB2 EFNA3 |
| EPHB2 | EFNA5 EFNB1 EFNB3 EFNB2 |
| EPHB3 | EFNB1 |
| EPHB4 | EFNB1 |
| EPHB6 | EFNB1 EFNB3 EFNB2 |
| ERBB2 | NRG1 |
| ERBB3 | BTC NRG1 |
| ERBB4 | BTC NRG1 NRG4 NRG2 HBEGF NRG3 EREG |
| F2R | F2 |
| F2RL1 | F2 |
| F2RL2 | F2 |
| F2RL3 | F2 |
| F3 | F7 |
| FAS | FASLG |
| FGFR1 | FGF14 FGF6 FGF18 FGF10 FGF2 FGF11 FGF3 FGF17 FGF1 FGF12 FGF4 FGF19 FGF13 FGF16 FGF5 FGF9 FGF8 FGF23 |
| FGFR2 | FGF14 FGF7 |
| FGFR3 | FGF14 |
| FGFR4 | FGF14 |
| FLT1 | VEGFA |
| FLT4 | PDGFC |
| FPR1 | ANXA1 |
| FPR2 | ANXA1 |
| FPR3 | ANXA1 |
| FSHR | CGA |
| FZD2 | WNT5A WNT4 |
| FZD4 | WNT5A |
| FZD6 | WNT5A |
| FZD7 | WNT5A WNT11 |
| GFRA1 | NRTN GDNF |
| GFRA2 | NRTN |
| GFRAL | GDF15 |
| GHR | GH1 GH2 |
| GIPR | GIP |
| GNRHR | GNRH2 GNRH1 |
| GPC1 | COL18A1 SLIT2 SLIT1 |
| GPR182 | ADM |
| GPR35 | CXCL17 |
| GPR37L1 | PSAP |
| GPR75 | CCL5 |
| GRPR | NMB |
| HHIP | DHH IHH SHH |
| HMMR | HAS2 |
| HRH1 | HDC |
| HTR1D | TPH1 |
| HTR1E | TPH1 |
| HTR2B | TPH1 |
| HTR2C | TPH1 |
| IFNAR1 | IFNA13 IFNA16 IFNA7 IFNA21 IFNA2 IFNA1 IFNA4 IFNA17 IFNA10 IFNK IFNA5 IFNW1 IFNA6 IFNB1 IFNA14 IFNA8 |
| IGF1R | IGF1 |
| IL10RB | IL22 IFNL2 IL10 IFNL3 IFNL1 |
| IL11RA | IL11 |
| IL13RA2 | IL13 |
| IL17RA | IL17B |
| IL17RB | IL17B |
| IL18R1 | IL37 IL18 |
| IL1R1 | IL1B IL1RN IL1A |
| IL1R2 | IL1R1 IL1B IL1A |
| IL1RAP | IL33 IL36A IL36G IL1B IL1A IL36B IL1RN IL36RN |
| IL1RL1 | IL33 |
| IL2RG | IL7 IL15 IL9 IL4 IL2 IL21 IL1RN IL13 |
| IL6ST | CRLF1 CTF1 OSM EBI3 CNTF IL6 IL11 IL27 LIF CLCF1 |
| IL7R | IL7 TSLP |
| INSR | INS IGF2 NAMPT |
| ITGA1 | COL4A1 DUSP18 |
| ITGA11 | COL1A1 |
| ITGA2 | COL1A1 FN1 COL2A1 CHAD DUSP18 |
| ITGA2B | FN1 |
| ITGA3 | RELN |
| ITGA4 | FN1 MADCAM1 VCAM1 |
| ITGA5 | COL18A1 FN1 VTN ADAM15 ADAM23 ADAM17 |
| ITGA6 | LAMB1 |
| ITGA7 | LAMA2 LAMA1 DUSP18 |
| ITGA8 | FN1 NPNT |
| ITGAD | VCAM1 |
| ITGAE | CDH1 |
| ITGAL | ICAM1 |
| ITGAX | C3 ICAM1 |
| ITGB1 | COL18A1 COL1A1 COL4A1 FN1 LAMA2 LAMB1 NPNT RELN VCAM1 |
| ITGB2 | C3 ICAM1 VCAM1 ICAM2 ICAM3 ICAM5 FGA ICAM4 |
| ITGB3 | FN1 VTN |
| ITGB6 | FN1 |
| ITGB7 | CDH1 MADCAM1 |
| ITGB8 | COL4A1 FN1 |
| KDR | PDGFC SEMA6D VEGFA VEGFC |
| KIR2DL1 | HLA-C |
| KIR2DL3 | HLA-C |
| KIR2DS4 | HLA-C |
| KIR3DL1 | HLA-B |
| KIR3DL2 | HLA-A |
| KIT | KITLG |
| KLRC1 | HLA-E |
| KLRC2 | HLA-E |
| KLRD1 | HLA-E |
| KREMEN1 | DKK1 |
| KREMEN2 | DKK1 |
| LDLR | APOE PF4 APOB |
| LGR4 | RSPO1 RSPO2 RSPO3 RSPO4 NDP |
| LGR5 | RSPO1 RSPO2 RSPO3 |
| LGR6 | RSPO1 RSPO2 RSPO3 |
| LHCGR | CGA |
| LIFR | CRLF1 CTF1 CLCF1 CNTF LIF OSM |
| LILRB1 | HLA-G |
| LINGO1 | RTN4 OMG LOC101927057 |
| LOC105377067 | CCL2 CCL26 CCL5 CXCL10 CXCL11 |
| LRP1 | C3 PLAT SERPINA1 SERPINE2 TFPI |
| LRP5 | APOE SOST |
| LRP6 | DKK1 RSPO1 SOST |
| LRP8 | APOE RELN |
| LRRC4C | NTNG1 |
| LTBR | LTB TNFSF14 TNF LTA |
| MC1R | ASIP |
| MC2R | CRH POMC |
| MERTK | GAS6 ZP3 |
| MET | HGF SEMA5A SEMA4D |
| MGRN1 | ASIP |
| MRGPRX1 | PENK |
| MRGPRX2 | ADM SST TAC1 |
| MST1R | MST1 |
| MYLK | CALM1 CALM3 CALM2 |
| NGFR | NGF |
| NMBR | NMB |
| NMUR1 | NMU |
| NMUR2 | NMU |
| NOTCH1 | DLK1 JAG1 |
| NOTCH2 | DLK1 JAG1 DLL1 DLL3 DLL4 JAG2 |
| NOTCH3 | DLK1 JAG1 JAG2 DLL4 DLL1 DLL3 |
| NOTCH4 | DLK1 JAG1 |
| NPFFR1 | NPFF NPVF |
| NPFFR2 | NPFF NPVF |
| NPR3 | OSTN NPPB NPPA |
| NPY1R | NPY PYY |
| NPY2R | NPY PYY |
| NPY4R | NPY PYY |
| NPY5R | NPY PYY |
| NRP1 | SEMA3B VEGFA VEGFB PGF SEMA3A |
| NRP2 | SEMA3C SEMA3F VEGFC |
| NRXN3 | NLGN1 NXPH3 NLGN2 NLGN3 NXPH1 |
| NTRK1 | NGF |
| NTRK2 | BDNF NTF3 NTF4 |
| NTSR1 | NTS |
| NTSR2 | NTS |
| OGFR | PENK |
| OPRD1 | PENK |
| OPRL1 | PNOC |
| OPRM1 | PENK |
| OSMR | OSM IL31 |
| P2RX7 | CAMP |
| P2RY14 | QRFP |
| PDGFRA | PDGFC PDGFB PDGFA |
| PDGFRB | PDGFB PDGFD |
| PLXNA1 | SEMA6D |
| PLXNA3 | SEMA3F |
| PLXNA4 | SEMA3A |
| PLXNB1 | SEMA4D |
| PLXNB2 | SEMA4D |
| PLXNB3 | SEMA5A |
| PLXND1 | SEMA3E |
| PRLHR | NPY |
| PROCR | PF4 PROC |
| PROKR1 | PROK1 |
| PROKR2 | PROK1 |
| PTCH1 | SHH IHH |
| PTCH2 | DHH |
| PTPRB | MDK PTN |
| PTPRF | NID1 |
| PTPRZ1 | MDK PTN |
| QRFPR | QRFP |
| RAMP1 | ADM CALCB CALCA ADM2 IAPP |
| RAMP2 | ADM IAPP CALCA ADM2 CALCB |
| RAMP3 | ADM |
| RET | NRTN |
| ROBO1 | NCAM1 SLIT2 SLIT1 |
| ROBO2 | SLIT2 |
| ROBO3 | NCAM1 |
| ROBO4 | SLIT2 |
| RPSA | LAMA2 LAMB2 |
| RTN4R | RTN4 |
| RXFP3 | INSL5 |
| RXFP4 | INSL5 |
| RYK | WNT5A |
| SCARF1 | CALR |
| SDC1 | SLIT2 THBS1 |
| SDC2 | FN1 |
| SDC4 | FGF2 ADAM12 |
| SELE | SELPLG |
| SELL | CD34 CFH |
| SORL1 | APOE |
| SORT1 | NGF |
| SSTR1 | SST |
| SSTR2 | SST CORT |
| SSTR3 | SST CORT |
| SSTR4 | SST |
| SSTR5 | SST |
| TACR1 | TAC1 TAC3 TAC4 |
| TACR2 | TAC1 |
| TACR3 | TAC1 |
| TEK | ANGPT2 ANGPTL1 |
| TFRC | TF |
| TGFBR1 | GDF9 TGFB2 |
| TGFBR2 | TGFB2 |
| TGFBR3 | INHA TGFB2 TGFB1 TGFB3 |
| THBD | PF4 |
| TIE1 | ANGPT1 |
| TNFRSF10A | TNFSF10 |
| TNFRSF12A | TNFSF12 |
| TNFRSF13B | TNFSF13B |
| TNFRSF13C | TNFSF13B |
| TNFRSF14 | BTLA |
| TNFRSF17 | TNFSF13B |
| TNFRSF1B | LTA TNF LOC101927057 OMG |
| TNFRSF6B | FASLG |
| TNFRSF8 | TNFSF8 |
| TSHR | CGA GPHA2 |
| TYRO3 | GAS6 |
| UNC5C | NTN1 |
| VIPR1 | ADCYAP1 VIP SCT GHRH |
| VIPR2 | ADCYAP1 |
| VLDLR | APOE RELN TFPI |
| XCR1 | CXCL2 |
