# Supplementary material for: Sctensor detects many-to-many cell–cell interactions from single cell RNA-sequencing data
Source: BMC Bioinformatics. 2023 Nov 7;24:420. doi: 10.1186/s12859-023-05490-y (PMC10631077; doi:10.1186/s12859-023-05490-y)
Supplement: Supplementary file 15 — Additional file 15. HTML report of GermlineFemale. [file 12859_2023_5490_MOESM15_ESM.zip › output/report/Human_Germline_Female/pattern_1_1.html]

Details of (1,1,) -related L-R pairs


# Details of (1,1,) -related L-R pairs

# (1,1,:) Pattern

# (1,1,) -related L-R pairs (Top9 pairs)

```
library("scTensor")
load("reanalysis.RData")
col <- .setColor("many")[2]
scTensor:::.myvisNetwork(g, col)
```

| Rank | Ligand Gene | Receptor Gene | Ligand Expression (Log10(exp + 1)) | Receptor Expression (Log10(exp + 1)) | LR-pair factor value (Percentage) | P-value (Grubbs test) | Q-value (BH method) | L-R Evidence cf. rikenbit/lrbase-workflow | PubMed |
| --- | --- | --- | --- | --- | --- | --- | --- | --- | --- |
| 1 | VEGFA Description: VEGFAvascular endothelial growth factor A GO: 1-62 63-123 Reactome: 1-28 UniProtKB: 1-9 RefEx: VEGFA Expression Atlas: VEGFA Single Cell Expression Atlas: VEGFA scRNASeqDB: VEGFA PanglaoDB: VEGFA CMap: VEGFA MeSH: 1-100 101-200 201-300 301-400 401-500 501-600 601-700 701-800 801-900 901-1000 1001-1100 1101-1200 1201-1300 1301-1400 1401-1500 1501-1600 1601-1700 1701-1800 1801-1900 1901-2000 2001-2100 2101-2200 2201-2300 2301-2400 2401-2500 2501-2600 2601-2700 2701-2800 2801-2900 2901-3000 3001-3100 3101-3200 3201-3300 3301-3400 3401-3500 3501-3600 3601-3700 3701-3800 3801-3900 3901-4000 4001-4100 4101-4200 4201-4300 4301-4400 4401-4500 4501-4600 4601-4700 4701-4800 4801-4900 4901-5000 5001-5100 5101-5200 5201-5300 5301-5400 5401-5500 5501-5556 | KDR Description: VEGFAkinase insert domain receptor GO: 1-70 Reactome: 1-14 UniProtKB: 1-2 RefEx: VEGFA Expression Atlas: KDR Single Cell Expression Atlas: KDR scRNASeqDB: KDR PanglaoDB: KDR CMap: KDR MeSH: 1-97 98-194 195-291 292-388 389-485 486-582 583-679 680-776 777-873 874-970 971-1067 1068-1164 1165-1261 1262-1358 1359-1455 1456-1552 1553-1649 1650-1746 1747-1843 1844-1940 1941-2037 2038-2113 |  |  | 4.362 (15.9%) | 0 | 0 | DLRP | 1 |
| 2 | VEGFA Description: VEGFAvascular endothelial growth factor A GO: 1-62 63-123 Reactome: 1-28 UniProtKB: 1-9 RefEx: VEGFA Expression Atlas: VEGFA Single Cell Expression Atlas: VEGFA scRNASeqDB: VEGFA PanglaoDB: VEGFA CMap: VEGFA MeSH: 1-100 101-200 201-300 301-400 401-500 501-600 601-700 701-800 801-900 901-1000 1001-1100 1101-1200 1201-1300 1301-1400 1401-1500 1501-1600 1601-1700 1701-1800 1801-1900 1901-2000 2001-2100 2101-2200 2201-2300 2301-2400 2401-2500 2501-2600 2601-2700 2701-2800 2801-2900 2901-3000 3001-3100 3101-3200 3201-3300 3301-3400 3401-3500 3501-3600 3601-3700 3701-3800 3801-3900 3901-4000 4001-4100 4101-4200 4201-4300 4301-4400 4401-4500 4501-4600 4601-4700 4701-4800 4801-4900 4901-5000 5001-5100 5101-5200 5201-5300 5301-5400 5401-5500 5501-5556 | NRP1 Description: VEGFAneuropilin 1 GO: 1-58 59-116 Reactome: 1-15 UniProtKB: 1-6 RefEx: VEGFA Expression Atlas: NRP1 Single Cell Expression Atlas: NRP1 scRNASeqDB: NRP1 PanglaoDB: NRP1 CMap: NRP1 MeSH: 1-96 97-192 193-288 289-384 385-480 481-576 577-672 673-768 769-864 865-960 961-1056 1057-1147 |  |  | 2.466 (8.987%) | 0.033 | 0.048 | DLRP | 1 |
| 3 | SEMA6D Description: SEMA6Dsemaphorin 6D GO: 1-15 Reactome: 1-5 UniProtKB: 1-2 RefEx: SEMA6D Expression Atlas: SEMA6D Single Cell Expression Atlas: SEMA6D scRNASeqDB: SEMA6D PanglaoDB: SEMA6D CMap: SEMA6D MeSH: 1-96 97-191 | KDR Description: SEMA6Dkinase insert domain receptor GO: 1-70 Reactome: 1-14 UniProtKB: 1-2 RefEx: SEMA6D Expression Atlas: KDR Single Cell Expression Atlas: KDR scRNASeqDB: KDR PanglaoDB: KDR CMap: KDR MeSH: 1-97 98-194 195-291 292-388 389-485 486-582 583-679 680-776 777-873 874-970 971-1067 1068-1164 1165-1261 1262-1358 1359-1455 1456-1552 1553-1649 1650-1746 1747-1843 1844-1940 1941-2037 2038-2113 |  |  | 2.224 (8.107%) | 0 | 0 | DLRP | 1 |
| 4 | COL4A1 Description: COL4A1collagen type IV alpha 1 chain GO: 1-22 Reactome: 1-24 UniProtKB: 1-3 RefEx: COL4A1 Expression Atlas: COL4A1 Single Cell Expression Atlas: COL4A1 scRNASeqDB: COL4A1 PanglaoDB: COL4A1 CMap: COL4A1 MeSH: 1-94 95-188 189-282 283-376 377-470 471-564 565-658 659-752 753-846 847-940 941-1032 | ITGA1 Description: COL4A1integrin subunit alpha 1 GO: 1-29 Reactome: 1-14 UniProtKB: 1 RefEx: COL4A1 Expression Atlas: ITGA1 Single Cell Expression Atlas: ITGA1 scRNASeqDB: ITGA1 PanglaoDB: ITGA1 CMap: ITGA1 MeSH: 1-91 92-182 183-273 274-364 365-455 456-546 547-637 638-728 729-817 |  |  | 1.827 (6.66%) | 0 | 0 | DLRP | 1 |
| 5 | BMP2 Description: BMP2bone morphogenetic protein 2 GO: 1-57 58-113 Reactome: 1-11 UniProtKB: 1-2 STRING: 1 RefEx: BMP2 Expression Atlas: BMP2 Single Cell Expression Atlas: BMP2 scRNASeqDB: BMP2 PanglaoDB: BMP2 CMap: BMP2 MeSH: 1-98 99-196 197-294 295-392 393-490 491-588 589-686 687-784 785-882 883-980 981-1078 1079-1176 1177-1274 1275-1372 1373-1470 1471-1568 1569-1666 1667-1764 1765-1862 1863-1960 1961-2058 2059-2156 2157-2254 2255-2352 2353-2450 2451-2548 2549-2646 2647-2742 | BMPR2 Description: BMP2bone morphogenetic protein receptor type 2 GO: 1-91 Reactome: 1-3 UniProtKB: 1 RefEx: BMP2 Expression Atlas: BMPR2 Single Cell Expression Atlas: BMPR2 scRNASeqDB: BMPR2 PanglaoDB: BMPR2 CMap: BMPR2 MeSH: 1-100 101-200 201-300 301-400 401-500 501-600 601-700 701-800 801-900 901-1000 1001-1098 |  |  | 1.631 (5.944%) | 0.292 | 0.309 | DLRP | 1 |
| 6 | INHA Description: INHAinhibin subunit alpha GO: 1-36 Reactome: 1-4 UniProtKB: 1 STRING: 1 RefEx: INHA Expression Atlas: INHA Single Cell Expression Atlas: INHA scRNASeqDB: INHA PanglaoDB: INHA CMap: INHA MeSH: 1-97 98-194 195-291 292-388 389-485 486-582 583-675 | TGFBR3 Description: INHAtransforming growth factor beta receptor 3 GO: 1-58 UniProtKB: 1-2 RefEx: INHA Expression Atlas: TGFBR3 Single Cell Expression Atlas: TGFBR3 scRNASeqDB: TGFBR3 PanglaoDB: TGFBR3 CMap: TGFBR3 MeSH: 1-91 92-182 183-273 274-364 365-455 456-546 547-637 638-726 |  |  | 1.577 (5.749%) | 0.008 | 0.017 | DLRP | 1 |
| 7 | PDGFC Description: PDGFCplatelet derived growth factor C GO: 1-32 Reactome: 1-3 UniProtKB: 1 RefEx: PDGFC Expression Atlas: PDGFC Single Cell Expression Atlas: PDGFC scRNASeqDB: PDGFC PanglaoDB: PDGFC CMap: PDGFC MeSH: 1-88 89-176 177-264 265-352 353-440 441-528 529-614 | KDR Description: PDGFCkinase insert domain receptor GO: 1-70 Reactome: 1-14 UniProtKB: 1-2 RefEx: PDGFC Expression Atlas: KDR Single Cell Expression Atlas: KDR scRNASeqDB: KDR PanglaoDB: KDR CMap: KDR MeSH: 1-97 98-194 195-291 292-388 389-485 486-582 583-679 680-776 777-873 874-970 971-1067 1068-1164 1165-1261 1262-1358 1359-1455 1456-1552 1553-1649 1650-1746 1747-1843 1844-1940 1941-2037 2038-2113 |  |  | 1.322 (4.82%) | 0.04 | 0.054 | DLRP | 1 |
| 8 | ADM Description: ADMadrenomedullin GO: 1-47 Reactome: 1-13 UniProtKB: 1 RefEx: ADM Expression Atlas: ADM Single Cell Expression Atlas: ADM scRNASeqDB: ADM PanglaoDB: ADM CMap: ADM MeSH: 1-99 100-198 199-297 298-396 397-495 496-594 595-693 694-792 793-891 892-990 991-1089 1090-1188 1189-1287 1288-1386 1387-1485 1486-1584 1585-1682 | RAMP2 Description: ADMreceptor activity modifying protein 2 GO: 1-45 Reactome: 1-13 UniProtKB: 1-2 RefEx: ADM Expression Atlas: RAMP2 Single Cell Expression Atlas: RAMP2 scRNASeqDB: RAMP2 PanglaoDB: RAMP2 CMap: RAMP2 MeSH: 1-89 90-178 179-267 268-355 |  |  | 1.284 (4.679%) | 0 | 0 | DLRP | 1 |
| 9 | NPNT Description: NPNTnephronectin GO: 1-24 UniProtKB: 1 RefEx: NPNT Expression Atlas: NPNT Single Cell Expression Atlas: NPNT scRNASeqDB: NPNT PanglaoDB: NPNT CMap: NPNT MeSH: 1-88 89-175 | ITGA8 Description: NPNTintegrin subunit alpha 8 GO: 1-17 Reactome: 1-5 UniProtKB: 1-2 STRING: 1 RefEx: NPNT Expression Atlas: ITGA8 Single Cell Expression Atlas: ITGA8 scRNASeqDB: ITGA8 PanglaoDB: ITGA8 CMap: ITGA8 MeSH: 1-82 83-164 165-246 247-328 |  |  | 1.253 (4.568%) | 0 | 0.002 | DLRP | 1 |

# (1,1,) -related Enrichment Analysis

```
load("reanalysis.RData")
library("plotly")
```

## MeSH-Enrichment Analysis (A : Anatomy)

```
negLogPval <- -log10(out.vecLR[['pattern_1_1']]$Enrich$MeSH_A$Pvalue + 1E-10)
term <- out.vecLR[['pattern_1_1']]$Enrich$MeSH_A$Term
target <- seq_len(min(100, length(negLogPval)))
negLogPval <- negLogPval[target]
term <- term[target]
p <- plot_ly(x=seq_along(negLogPval), y=~negLogPval,
type="bar", color=~negLogPval, text=term,
colors=c("#4b61ba", "gray", "#a87963", "red"))
layout(p, title="MeSH-Enrichment Analysis (A)",
   xaxis=list(title="Term"),
   yaxis=list(title="-Log(P-value)"),
   legend=list(name=""))
```

```
## Warning: textfont.color doesn't (yet) support data arrays

## Warning: textfont.color doesn't (yet) support data arrays
```

## MeSH-Enrichment Analysis (B : Organisms)

```
negLogPval <- -log10(out.vecLR[['pattern_1_1']]$Enrich$MeSH_B$Pvalue + 1E-10)
term <- out.vecLR[['pattern_1_1']]$Enrich$MeSH_B$Term
target <- seq_len(min(100, length(negLogPval)))
negLogPval <- negLogPval[target]
term <- term[target]
p <- plot_ly(x=seq_along(negLogPval), y=~negLogPval,
type="bar", color=~negLogPval, text=term,
colors=c("#4b61ba", "gray", "#a87963", "red"))
layout(p, title="MeSH-Enrichment Analysis (B)",
   xaxis=list(title="Term"),
   yaxis=list(title="-Log(P-value)"),
   legend=list(name=""))
```

```
## Warning: textfont.color doesn't (yet) support data arrays

## Warning: textfont.color doesn't (yet) support data arrays
```

## MeSH-Enrichment Analysis (C : Diseases)

```
negLogPval <- -log10(out.vecLR[['pattern_1_1']]$Enrich$MeSH_C$Pvalue + 1E-10)
term <- out.vecLR[['pattern_1_1']]$Enrich$MeSH_C$Term
target <- seq_len(min(100, length(negLogPval)))
negLogPval <- negLogPval[target]
term <- term[target]
p <- plot_ly(x=seq_along(negLogPval), y=~negLogPval,
type="bar", color=~negLogPval, text=term,
colors=c("#4b61ba", "gray", "#a87963", "red"))
layout(p, title="MeSH-Enrichment Analysis (C)",
   xaxis=list(title="Term"),
   yaxis=list(title="-Log(P-value)"),
   legend=list(name=""))
```

```
## Warning: textfont.color doesn't (yet) support data arrays

## Warning: textfont.color doesn't (yet) support data arrays
```

## MeSH-Enrichment Analysis (D : Drugs)

```
negLogPval <- -log10(out.vecLR[['pattern_1_1']]$Enrich$MeSH_D$Pvalue + 1E-10)
term <- out.vecLR[['pattern_1_1']]$Enrich$MeSH_D$Term
target <- seq_len(min(100, length(negLogPval)))
negLogPval <- negLogPval[target]
term <- term[target]
p <- plot_ly(x=seq_along(negLogPval), y=~negLogPval,
type="bar", color=~negLogPval, text=term,
colors=c("#4b61ba", "gray", "#a87963", "red"))
layout(p, title="MeSH-Enrichment Analysis (D)",
   xaxis=list(title="Term"),
   yaxis=list(title="-Log(P-value)"),
   legend=list(name=""))
```

```
## Warning: textfont.color doesn't (yet) support data arrays

## Warning: textfont.color doesn't (yet) support data arrays
```

## MeSH-Enrichment Analysis (E : Analytical, Diagnostic and Therapeutic Techniques and Equipment)

```
negLogPval <- -log10(out.vecLR[['pattern_1_1']]$Enrich$MeSH_E$Pvalue + 1E-10)
term <- out.vecLR[['pattern_1_1']]$Enrich$MeSH_E$Term
target <- seq_len(min(100, length(negLogPval)))
negLogPval <- negLogPval[target]
term <- term[target]
p <- plot_ly(x=seq_along(negLogPval), y=~negLogPval,
type="bar", color=~negLogPval, text=term,
colors=c("#4b61ba", "gray", "#a87963", "red"))
layout(p, title="MeSH-Enrichment Analysis (E)",
   xaxis=list(title="Term"),
   yaxis=list(title="-Log(P-value)"),
   legend=list(name=""))
```

```
## Warning: textfont.color doesn't (yet) support data arrays

## Warning: textfont.color doesn't (yet) support data arrays
```

## MeSH-Enrichment Analysis (F : Psychiatry and Psychology)

```
negLogPval <- -log10(out.vecLR[['pattern_1_1']]$Enrich$MeSH_F$Pvalue + 1E-10)
term <- out.vecLR[['pattern_1_1']]$Enrich$MeSH_F$Term
target <- seq_len(min(100, length(negLogPval)))
negLogPval <- negLogPval[target]
term <- term[target]
p <- plot_ly(x=seq_along(negLogPval), y=~negLogPval,
type="bar", color=~negLogPval, text=term,
colors=c("#4b61ba", "gray", "#a87963", "red"))
layout(p, title="MeSH-Enrichment Analysis (F)",
   xaxis=list(title="Term"),
   yaxis=list(title="-Log(P-value)"),
   legend=list(name=""))
```

```
## Warning: textfont.color doesn't (yet) support data arrays

## Warning: textfont.color doesn't (yet) support data arrays
```

## MeSH-Enrichment Analysis (G : Phenomena and Processes)

```
negLogPval <- -log10(out.vecLR[['pattern_1_1']]$Enrich$MeSH_G$Pvalue + 1E-10)
term <- out.vecLR[['pattern_1_1']]$Enrich$MeSH_G$Term
target <- seq_len(min(100, length(negLogPval)))
negLogPval <- negLogPval[target]
term <- term[target]
p <- plot_ly(x=seq_along(negLogPval), y=~negLogPval,
type="bar", color=~negLogPval, text=term,
colors=c("#4b61ba", "gray", "#a87963", "red"))
layout(p, title="MeSH-Enrichment Analysis (G)",
   xaxis=list(title="Term"),
   yaxis=list(title="-Log(P-value)"),
   legend=list(name=""))
```

```
## Warning: textfont.color doesn't (yet) support data arrays

## Warning: textfont.color doesn't (yet) support data arrays
```

## MeSH-Enrichment Analysis (H : Disciplines and Occupations)

```
negLogPval <- -log10(out.vecLR[['pattern_1_1']]$Enrich$MeSH_H$Pvalue + 1E-10)
term <- out.vecLR[['pattern_1_1']]$Enrich$MeSH_H$Term
target <- seq_len(min(100, length(negLogPval)))
negLogPval <- negLogPval[target]
term <- term[target]
p <- plot_ly(x=seq_along(negLogPval), y=~negLogPval,
type="bar", color=~negLogPval, text=term,
colors=c("#4b61ba", "gray", "#a87963", "red"))
layout(p, title="MeSH-Enrichment Analysis (H)",
   xaxis=list(title="Term"),
   yaxis=list(title="-Log(P-value)"),
   legend=list(name=""))
```

```
## Warning: textfont.color doesn't (yet) support data arrays

## Warning: textfont.color doesn't (yet) support data arrays
```

## MeSH-Enrichment Analysis (I : Anthropology, Education, Sociology and Social Phenomena)

```
negLogPval <- -log10(out.vecLR[['pattern_1_1']]$Enrich$MeSH_I$Pvalue + 1E-10)
term <- out.vecLR[['pattern_1_1']]$Enrich$MeSH_I$Term
target <- seq_len(min(100, length(negLogPval)))
negLogPval <- negLogPval[target]
term <- term[target]
p <- plot_ly(x=seq_along(negLogPval), y=~negLogPval,
type="bar", color=~negLogPval, text=term,
colors=c("#4b61ba", "gray", "#a87963", "red"))
layout(p, title="MeSH-Enrichment Analysis (I)",
   xaxis=list(title="Term"),
   yaxis=list(title="-Log(P-value)"),
   legend=list(name=""))
```

```
## Warning: textfont.color doesn't (yet) support data arrays

## Warning: textfont.color doesn't (yet) support data arrays
```

## MeSH-Enrichment Analysis (J : Technology and Food and Beverages)

```
negLogPval <- -log10(out.vecLR[['pattern_1_1']]$Enrich$MeSH_J$Pvalue + 1E-10)
term <- out.vecLR[['pattern_1_1']]$Enrich$MeSH_J$Term
target <- seq_len(min(100, length(negLogPval)))
negLogPval <- negLogPval[target]
term <- term[target]
p <- plot_ly(x=seq_along(negLogPval), y=~negLogPval,
type="bar", color=~negLogPval, text=term,
colors=c("#4b61ba", "gray", "#a87963", "red"))
layout(p, title="MeSH-Enrichment Analysis (J)",
   xaxis=list(title="Term"),
   yaxis=list(title="-Log(P-value)"),
   legend=list(name=""))
```

```
## Warning: textfont.color doesn't (yet) support data arrays

## Warning: textfont.color doesn't (yet) support data arrays
```

## MeSH-Enrichment Analysis (L : Information Science)

```
negLogPval <- -log10(out.vecLR[['pattern_1_1']]$Enrich$MeSH_L$Pvalue + 1E-10)
term <- out.vecLR[['pattern_1_1']]$Enrich$MeSH_L$Term
target <- seq_len(min(100, length(negLogPval)))
negLogPval <- negLogPval[target]
term <- term[target]
p <- plot_ly(x=seq_along(negLogPval), y=~negLogPval,
type="bar", color=~negLogPval, text=term,
colors=c("#4b61ba", "gray", "#a87963", "red"))
layout(p, title="MeSH-Enrichment Analysis (L)",
   xaxis=list(title="Term"),
   yaxis=list(title="-Log(P-value)"),
   legend=list(name=""))
```

## MeSH-Enrichment Analysis (N : Health Care)

```
negLogPval <- -log10(out.vecLR[['pattern_1_1']]$Enrich$MeSH_N$Pvalue + 1E-10)
term <- out.vecLR[['pattern_1_1']]$Enrich$MeSH_N$Term
target <- seq_len(min(100, length(negLogPval)))
negLogPval <- negLogPval[target]
term <- term[target]
p <- plot_ly(x=seq_along(negLogPval), y=~negLogPval,
type="bar", color=~negLogPval, text=term,
colors=c("#4b61ba", "gray", "#a87963", "red"))
layout(p, title="MeSH-Enrichment Analysis (N)",
   xaxis=list(title="Term"),
   yaxis=list(title="-Log(P-value)"),
   legend=list(name=""))
```

```
## Warning: textfont.color doesn't (yet) support data arrays

## Warning: textfont.color doesn't (yet) support data arrays
```

## MeSH-Enrichment Analysis (Z : Geographical Locations)

```
negLogPval <- -log10(out.vecLR[['pattern_1_1']]$Enrich$MeSH_Z$Pvalue + 1E-10)
term <- out.vecLR[['pattern_1_1']]$Enrich$MeSH_Z$Term
target <- seq_len(min(100, length(negLogPval)))
negLogPval <- negLogPval[target]
term <- term[target]
p <- plot_ly(x=seq_along(negLogPval), y=~negLogPval,
type="bar", color=~negLogPval, text=term,
colors=c("#4b61ba", "gray", "#a87963", "red"))
layout(p, title="MeSH-Enrichment Analysis (Z)",
   xaxis=list(title="Term"),
   yaxis=list(title="-Log(P-value)"),
   legend=list(name=""))
```

## Reactome Pathway-Enrichment Analysis

```
negLogPval <- -log10(out.vecLR[['pattern_1_1']]$Enrich$Reactome$Pvalue + 1E-10)
term <- out.vecLR[['pattern_1_1']]$Enrich$Reactome$Term
target <- seq_len(min(100, length(negLogPval)))
negLogPval <- negLogPval[target]
term <- term[target]
p <- plot_ly(x=seq_along(negLogPval), y=~negLogPval,
type="bar", color=~negLogPval, text=term,
colors=c("#4b61ba", "gray", "#a87963", "red"))
layout(p, title="Reactome-Enrichment Analysis",
   xaxis=list(title="Term"),
   yaxis=list(title="-Log(P-value)"),
   legend=list(name=""))
```

```
## Warning: textfont.color doesn't (yet) support data arrays

## Warning: textfont.color doesn't (yet) support data arrays
```

## DO-Enrichment Analysis

```
negLogPval <- -log10(out.vecLR[['pattern_1_1']]$Enrich$DO$Pvalue + 1E-10)
term <- out.vecLR[['pattern_1_1']]$Enrich$DO$Term
target <- seq_len(min(100, length(negLogPval)))
negLogPval <- negLogPval[target]
term <- term[target]
p <- plot_ly(x=seq_along(negLogPval), y=~negLogPval,
type="bar", color=~negLogPval, text=term,
colors=c("#4b61ba", "gray", "#a87963", "red"))
layout(p, title="DO-Enrichment Analysis",
   xaxis=list(title="Term"),
   yaxis=list(title="-Log(P-value)"),
   legend=list(name=""))
```

```
## Warning: textfont.color doesn't (yet) support data arrays

## Warning: textfont.color doesn't (yet) support data arrays
```

## NCG-Enrichment Analysis

```
negLogPval <- -log10(out.vecLR[['pattern_1_1']]$Enrich$NCG$Pvalue + 1E-10)
term <- out.vecLR[['pattern_1_1']]$Enrich$NCG$Term
target <- seq_len(min(100, length(negLogPval)))
negLogPval <- negLogPval[target]
term <- term[target]
p <- plot_ly(x=seq_along(negLogPval), y=~negLogPval,
type="bar", color=~negLogPval, text=term,
colors=c("#4b61ba", "gray", "#a87963", "red"))
layout(p, title="NCG-Enrichment Analysis",
   xaxis=list(title="Term"),
   yaxis=list(title="-Log(P-value)"),
   legend=list(name=""))
```

```
## Warning: textfont.color doesn't (yet) support data arrays

## Warning: textfont.color doesn't (yet) support data arrays
```

## DGN-Enrichment Analysis

```
negLogPval <- -log10(out.vecLR[['pattern_1_1']]$Enrich$DGN$Pvalue + 1E-10)
term <- out.vecLR[['pattern_1_1']]$Enrich$DGN$Term
target <- seq_len(min(100, length(negLogPval)))
negLogPval <- negLogPval[target]
term <- term[target]
p <- plot_ly(x=seq_along(negLogPval), y=~negLogPval,
type="bar", color=~negLogPval, text=term,
colors=c("#4b61ba", "gray", "#a87963", "red"))
layout(p, title="DGN-Enrichment Analysis",
   xaxis=list(title="Term"),
   yaxis=list(title="-Log(P-value)"),
   legend=list(name=""))
```

```
## Warning: textfont.color doesn't (yet) support data arrays

## Warning: textfont.color doesn't (yet) support data arrays
```
