# Supplementary material for: Sctensor detects many-to-many cell–cell interactions from single cell RNA-sequencing data
Source: BMC Bioinformatics. 2023 Nov 7;24:420. doi: 10.1186/s12859-023-05490-y (PMC10631077; doi:10.1186/s12859-023-05490-y)
Supplement: Supplementary file 15 — Additional file 15. HTML report of GermlineFemale. [file 12859_2023_5490_MOESM15_ESM.zip › output/report/Human_Germline_Female/pattern_4_1.html]

Details of (4,1,) -related L-R pairs


# Details of (4,1,) -related L-R pairs

# (4,1,:) Pattern

# (4,1,) -related L-R pairs (Top13 pairs)

```
library("scTensor")
load("reanalysis.RData")
col <- .setColor("many")[4]
scTensor:::.myvisNetwork(g, col)
```

| Rank | Ligand Gene | Receptor Gene | Ligand Expression (Log10(exp + 1)) | Receptor Expression (Log10(exp + 1)) | LR-pair factor value (Percentage) | P-value (Grubbs test) | Q-value (BH method) | L-R Evidence cf. rikenbit/lrbase-workflow | PubMed |
| --- | --- | --- | --- | --- | --- | --- | --- | --- | --- |
| 1 | GDF9 Description: GDF9growth differentiation factor 9 GO: 1-14 UniProtKB: 1-2 STRING: 1 2 3 4 5 RefEx: GDF9 Expression Atlas: GDF9 Single Cell Expression Atlas: GDF9 scRNASeqDB: GDF9 PanglaoDB: GDF9 CMap: GDF9 MeSH: 1-93 94-186 187-279 280-372 373-465 466-558 559-645 | BMPR2 Description: GDF9bone morphogenetic protein receptor type 2 GO: 1-91 Reactome: 1-3 UniProtKB: 1 RefEx: GDF9 Expression Atlas: BMPR2 Single Cell Expression Atlas: BMPR2 scRNASeqDB: BMPR2 PanglaoDB: BMPR2 CMap: BMPR2 MeSH: 1-100 101-200 201-300 301-400 401-500 501-600 601-700 701-800 801-900 901-1000 1001-1098 |  |  | 4.115 (19.547%) | 0 | 0 | DLRP | 1 |
| 2 | SEMA3E Description: SEMA3Esemaphorin 3E GO: 1-19 Reactome: 1-5 UniProtKB: 1 RefEx: SEMA3E Expression Atlas: SEMA3E Single Cell Expression Atlas: SEMA3E scRNASeqDB: SEMA3E PanglaoDB: SEMA3E CMap: SEMA3E MeSH: 1-86 87-172 173-258 259-341 | PLXND1 Description: SEMA3Eplexin D1 GO: 1-27 Reactome: 1-13 UniProtKB: 1 RefEx: SEMA3E Expression Atlas: PLXND1 Single Cell Expression Atlas: PLXND1 scRNASeqDB: PLXND1 PanglaoDB: PLXND1 CMap: PLXND1 MeSH: 1-76 77-152 153-228 229-301 |  |  | 2.232 (10.603%) | 0.002 | 0.003 | DLRP | 1 |
| 3 | F2 Description: F2coagulation factor II, thrombin GO: 1-48 Reactome: 1-33 UniProtKB: 1 RefEx: F2 Expression Atlas: F2 Single Cell Expression Atlas: F2 scRNASeqDB: F2 PanglaoDB: F2 CMap: F2 MeSH: 1-100 101-200 201-300 301-400 401-500 501-600 601-700 701-800 801-900 901-1000 1001-1100 1101-1200 1201-1300 1301-1400 1401-1500 1501-1600 1601-1700 1701-1800 1801-1900 1901-2000 2001-2100 2101-2198 | F2RL2 Description: F2coagulation factor II thrombin receptor like 2 GO: 1-15 Reactome: 1-10 UniProtKB: 1 STRING: 1 2 RefEx: F2 Expression Atlas: F2RL2 Single Cell Expression Atlas: F2RL2 scRNASeqDB: F2RL2 PanglaoDB: F2RL2 CMap: F2RL2 MeSH: 1-85 86-170 171-255 256-340 341-421 |  |  | 2.025 (9.619%) | 0.011 | 0.014 | DLRP | 1 |
| 4 | FN1 Description: FN1fibronectin 1 GO: 1-55 Reactome: 1-49 UniProtKB: 1-6 RefEx: FN1 Expression Atlas: FN1 Single Cell Expression Atlas: FN1 scRNASeqDB: FN1 PanglaoDB: FN1 CMap: FN1 MeSH: 1-97 98-194 195-291 292-388 389-485 486-582 583-679 680-776 777-873 874-970 971-1067 1068-1164 1165-1261 1262-1358 1359-1455 1456-1552 1553-1649 1650-1746 1747-1843 1844-1940 1941-2033 | ITGA5 Description: FN1integrin subunit alpha 5 GO: 1-28 Reactome: 1-16 UniProtKB: 1-2 RefEx: FN1 Expression Atlas: ITGA5 Single Cell Expression Atlas: ITGA5 scRNASeqDB: ITGA5 PanglaoDB: ITGA5 CMap: ITGA5 MeSH: 1-95 96-190 191-285 286-380 381-475 476-570 571-665 666-760 761-855 856-950 951-1045 1046-1140 1141-1235 1236-1330 1331-1414 |  |  | 1.413 (6.71%) | 0 | 0.001 | DLRP | 1 |
| 5 | JAG1 Description: JAG1jagged canonical Notch ligand 1 GO: 1-53 Reactome: 1-31 UniProtKB: 1-2 RefEx: JAG1 Expression Atlas: JAG1 Single Cell Expression Atlas: JAG1 scRNASeqDB: JAG1 PanglaoDB: JAG1 CMap: JAG1 MeSH: 1-99 100-198 199-297 298-396 397-495 496-594 595-693 694-792 793-891 892-990 991-1089 1090-1188 1189-1287 1288-1386 1387-1485 1486-1584 1585-1683 | NOTCH3 Description: JAG1notch receptor 3 GO: 1-19 Reactome: 1-19 UniProtKB: 1 STRING: 1 2 3 4 RefEx: JAG1 Expression Atlas: NOTCH3 Single Cell Expression Atlas: NOTCH3 scRNASeqDB: NOTCH3 PanglaoDB: NOTCH3 CMap: NOTCH3 MeSH: 1-99 100-198 199-297 298-396 397-495 496-594 595-693 694-792 793-891 892-990 991-1089 1090-1188 1189-1275 |  |  | 1.284 (6.1%) | 0 | 0.001 | DLRP | 1 |
| 6 | FN1 Description: FN1fibronectin 1 GO: 1-55 Reactome: 1-49 UniProtKB: 1-6 RefEx: FN1 Expression Atlas: FN1 Single Cell Expression Atlas: FN1 scRNASeqDB: FN1 PanglaoDB: FN1 CMap: FN1 MeSH: 1-97 98-194 195-291 292-388 389-485 486-582 583-679 680-776 777-873 874-970 971-1067 1068-1164 1165-1261 1262-1358 1359-1455 1456-1552 1553-1649 1650-1746 1747-1843 1844-1940 1941-2033 | ITGA8 Description: FN1integrin subunit alpha 8 GO: 1-17 Reactome: 1-5 UniProtKB: 1-2 STRING: 1 RefEx: FN1 Expression Atlas: ITGA8 Single Cell Expression Atlas: ITGA8 scRNASeqDB: ITGA8 PanglaoDB: ITGA8 CMap: ITGA8 MeSH: 1-82 83-164 165-246 247-328 |  |  | 1.264 (6.005%) | 0.009 | 0.013 | DLRP | 1 |
| 7 | FN1 Description: FN1fibronectin 1 GO: 1-55 Reactome: 1-49 UniProtKB: 1-6 RefEx: FN1 Expression Atlas: FN1 Single Cell Expression Atlas: FN1 scRNASeqDB: FN1 PanglaoDB: FN1 CMap: FN1 MeSH: 1-97 98-194 195-291 292-388 389-485 486-582 583-679 680-776 777-873 874-970 971-1067 1068-1164 1165-1261 1262-1358 1359-1455 1456-1552 1553-1649 1650-1746 1747-1843 1844-1940 1941-2033 | ITGA2 Description: FN1integrin subunit alpha 2 GO: 1-65 Reactome: 1-18 UniProtKB: 1 RefEx: FN1 Expression Atlas: ITGA2 Single Cell Expression Atlas: ITGA2 scRNASeqDB: ITGA2 PanglaoDB: ITGA2 CMap: ITGA2 MeSH: 1-95 96-190 191-285 286-380 381-475 476-570 571-665 666-760 761-855 856-950 951-1045 1046-1140 1141-1235 1236-1330 1331-1413 |  |  | 1.196 (5.681%) | 0.01 | 0.014 | DLRP | 1 |
| 8 | NPNT Description: NPNTnephronectin GO: 1-24 UniProtKB: 1 RefEx: NPNT Expression Atlas: NPNT Single Cell Expression Atlas: NPNT scRNASeqDB: NPNT PanglaoDB: NPNT CMap: NPNT MeSH: 1-88 89-175 | ITGA8 Description: NPNTintegrin subunit alpha 8 GO: 1-17 Reactome: 1-5 UniProtKB: 1-2 STRING: 1 RefEx: NPNT Expression Atlas: ITGA8 Single Cell Expression Atlas: ITGA8 scRNASeqDB: ITGA8 PanglaoDB: ITGA8 CMap: ITGA8 MeSH: 1-82 83-164 165-246 247-328 |  |  | 1.086 (5.157%) | 0.012 | 0.015 | DLRP | 1 |
| 9 | SEMA6D Description: SEMA6Dsemaphorin 6D GO: 1-15 Reactome: 1-5 UniProtKB: 1-2 RefEx: SEMA6D Expression Atlas: SEMA6D Single Cell Expression Atlas: SEMA6D scRNASeqDB: SEMA6D PanglaoDB: SEMA6D CMap: SEMA6D MeSH: 1-96 97-191 | KDR Description: SEMA6Dkinase insert domain receptor GO: 1-70 Reactome: 1-14 UniProtKB: 1-2 RefEx: SEMA6D Expression Atlas: KDR Single Cell Expression Atlas: KDR scRNASeqDB: KDR PanglaoDB: KDR CMap: KDR MeSH: 1-97 98-194 195-291 292-388 389-485 486-582 583-679 680-776 777-873 874-970 971-1067 1068-1164 1165-1261 1262-1358 1359-1455 1456-1552 1553-1649 1650-1746 1747-1843 1844-1940 1941-2037 2038-2113 |  |  | 0.863 (4.097%) | 0.003 | 0.006 | DLRP | 1 |
| 10 | COL18A1 Description: COL18A1collagen type XVIII alpha 1 chain GO: 1-20 Reactome: 1-10 UniProtKB: 1-3 RefEx: COL18A1 Expression Atlas: COL18A1 Single Cell Expression Atlas: COL18A1 scRNASeqDB: COL18A1 PanglaoDB: COL18A1 CMap: COL18A1 MeSH: 1-96 97-192 193-288 289-384 385-480 481-576 577-672 673-768 769-864 865-960 961-1054 | ITGA5 Description: COL18A1integrin subunit alpha 5 GO: 1-28 Reactome: 1-16 UniProtKB: 1-2 RefEx: COL18A1 Expression Atlas: ITGA5 Single Cell Expression Atlas: ITGA5 scRNASeqDB: ITGA5 PanglaoDB: ITGA5 CMap: ITGA5 MeSH: 1-95 96-190 191-285 286-380 381-475 476-570 571-665 666-760 761-855 856-950 951-1045 1046-1140 1141-1235 1236-1330 1331-1414 |  |  | 0.705 (3.35%) | 0 | 0.001 | DLRP | 1 |
| 11 | JAG1 Description: JAG1jagged canonical Notch ligand 1 GO: 1-53 Reactome: 1-31 UniProtKB: 1-2 RefEx: JAG1 Expression Atlas: JAG1 Single Cell Expression Atlas: JAG1 scRNASeqDB: JAG1 PanglaoDB: JAG1 CMap: JAG1 MeSH: 1-99 100-198 199-297 298-396 397-495 496-594 595-693 694-792 793-891 892-990 991-1089 1090-1188 1189-1287 1288-1386 1387-1485 1486-1584 1585-1683 | NOTCH2 Description: JAG1notch receptor 2 GO: 1-63 Reactome: 1-20 UniProtKB: 1-3 RefEx: JAG1 Expression Atlas: NOTCH2 Single Cell Expression Atlas: NOTCH2 scRNASeqDB: NOTCH2 PanglaoDB: NOTCH2 CMap: NOTCH2 MeSH: 1-93 94-186 187-279 280-372 373-465 466-558 559-651 652-744 745-837 838-930 931-1023 1024-1116 1117-1203 |  |  | 0.623 (2.957%) | 0 | 0 | DLRP | 1 |
| 12 | FGF14 Description: FGF14fibroblast growth factor 14 GO: 1-14 Reactome: 1-3 UniProtKB: 1 RefEx: FGF14 Expression Atlas: FGF14 Single Cell Expression Atlas: FGF14 scRNASeqDB: FGF14 PanglaoDB: FGF14 CMap: FGF14 MeSH: 1-86 87-172 173-256 | FGFR1 Description: FGF14fibroblast growth factor receptor 1 GO: 1-47 Reactome: 1-44 UniProtKB: 1-2 RefEx: FGF14 Expression Atlas: FGFR1 Single Cell Expression Atlas: FGFR1 scRNASeqDB: FGFR1 PanglaoDB: FGFR1 CMap: FGFR1 MeSH: 1-96 97-192 193-288 289-384 385-480 481-576 577-672 673-768 769-864 865-960 961-1056 1057-1152 1153-1248 1249-1344 1345-1440 1441-1536 1537-1632 1633-1728 1729-1819 |  |  | 0.566 (2.687%) | 0.038 | 0.042 | DLRP | 1 |
| 13 | ADM Description: ADMadrenomedullin GO: 1-47 Reactome: 1-13 UniProtKB: 1 RefEx: ADM Expression Atlas: ADM Single Cell Expression Atlas: ADM scRNASeqDB: ADM PanglaoDB: ADM CMap: ADM MeSH: 1-99 100-198 199-297 298-396 397-495 496-594 595-693 694-792 793-891 892-990 991-1089 1090-1188 1189-1287 1288-1386 1387-1485 1486-1584 1585-1682 | RAMP2 Description: ADMreceptor activity modifying protein 2 GO: 1-45 Reactome: 1-13 UniProtKB: 1-2 RefEx: ADM Expression Atlas: RAMP2 Single Cell Expression Atlas: RAMP2 scRNASeqDB: RAMP2 PanglaoDB: RAMP2 CMap: RAMP2 MeSH: 1-89 90-178 179-267 268-355 |  |  | 0.498 (2.363%) | 0.004 | 0.006 | DLRP | 1 |

# (4,1,) -related Enrichment Analysis

```
load("reanalysis.RData")
library("plotly")
```

## MeSH-Enrichment Analysis (A : Anatomy)

```
negLogPval <- -log10(out.vecLR[['pattern_4_1']]$Enrich$MeSH_A$Pvalue + 1E-10)
term <- out.vecLR[['pattern_4_1']]$Enrich$MeSH_A$Term
target <- seq_len(min(100, length(negLogPval)))
negLogPval <- negLogPval[target]
term <- term[target]
p <- plot_ly(x=seq_along(negLogPval), y=~negLogPval,
type="bar", color=~negLogPval, text=term,
colors=c("#4b61ba", "gray", "#a87963", "red"))
layout(p, title="MeSH-Enrichment Analysis (A)",
   xaxis=list(title="Term"),
   yaxis=list(title="-Log(P-value)"),
   legend=list(name=""))
```

```
## Warning: textfont.color doesn't (yet) support data arrays

## Warning: textfont.color doesn't (yet) support data arrays
```

## MeSH-Enrichment Analysis (B : Organisms)

```
negLogPval <- -log10(out.vecLR[['pattern_4_1']]$Enrich$MeSH_B$Pvalue + 1E-10)
term <- out.vecLR[['pattern_4_1']]$Enrich$MeSH_B$Term
target <- seq_len(min(100, length(negLogPval)))
negLogPval <- negLogPval[target]
term <- term[target]
p <- plot_ly(x=seq_along(negLogPval), y=~negLogPval,
type="bar", color=~negLogPval, text=term,
colors=c("#4b61ba", "gray", "#a87963", "red"))
layout(p, title="MeSH-Enrichment Analysis (B)",
   xaxis=list(title="Term"),
   yaxis=list(title="-Log(P-value)"),
   legend=list(name=""))
```

```
## Warning: textfont.color doesn't (yet) support data arrays

## Warning: textfont.color doesn't (yet) support data arrays
```

## MeSH-Enrichment Analysis (C : Diseases)

```
negLogPval <- -log10(out.vecLR[['pattern_4_1']]$Enrich$MeSH_C$Pvalue + 1E-10)
term <- out.vecLR[['pattern_4_1']]$Enrich$MeSH_C$Term
target <- seq_len(min(100, length(negLogPval)))
negLogPval <- negLogPval[target]
term <- term[target]
p <- plot_ly(x=seq_along(negLogPval), y=~negLogPval,
type="bar", color=~negLogPval, text=term,
colors=c("#4b61ba", "gray", "#a87963", "red"))
layout(p, title="MeSH-Enrichment Analysis (C)",
   xaxis=list(title="Term"),
   yaxis=list(title="-Log(P-value)"),
   legend=list(name=""))
```

```
## Warning: textfont.color doesn't (yet) support data arrays

## Warning: textfont.color doesn't (yet) support data arrays
```

## MeSH-Enrichment Analysis (D : Drugs)

```
negLogPval <- -log10(out.vecLR[['pattern_4_1']]$Enrich$MeSH_D$Pvalue + 1E-10)
term <- out.vecLR[['pattern_4_1']]$Enrich$MeSH_D$Term
target <- seq_len(min(100, length(negLogPval)))
negLogPval <- negLogPval[target]
term <- term[target]
p <- plot_ly(x=seq_along(negLogPval), y=~negLogPval,
type="bar", color=~negLogPval, text=term,
colors=c("#4b61ba", "gray", "#a87963", "red"))
layout(p, title="MeSH-Enrichment Analysis (D)",
   xaxis=list(title="Term"),
   yaxis=list(title="-Log(P-value)"),
   legend=list(name=""))
```

```
## Warning: textfont.color doesn't (yet) support data arrays

## Warning: textfont.color doesn't (yet) support data arrays
```

## MeSH-Enrichment Analysis (E : Analytical, Diagnostic and Therapeutic Techniques and Equipment)

```
negLogPval <- -log10(out.vecLR[['pattern_4_1']]$Enrich$MeSH_E$Pvalue + 1E-10)
term <- out.vecLR[['pattern_4_1']]$Enrich$MeSH_E$Term
target <- seq_len(min(100, length(negLogPval)))
negLogPval <- negLogPval[target]
term <- term[target]
p <- plot_ly(x=seq_along(negLogPval), y=~negLogPval,
type="bar", color=~negLogPval, text=term,
colors=c("#4b61ba", "gray", "#a87963", "red"))
layout(p, title="MeSH-Enrichment Analysis (E)",
   xaxis=list(title="Term"),
   yaxis=list(title="-Log(P-value)"),
   legend=list(name=""))
```

```
## Warning: textfont.color doesn't (yet) support data arrays

## Warning: textfont.color doesn't (yet) support data arrays
```

## MeSH-Enrichment Analysis (F : Psychiatry and Psychology)

```
negLogPval <- -log10(out.vecLR[['pattern_4_1']]$Enrich$MeSH_F$Pvalue + 1E-10)
term <- out.vecLR[['pattern_4_1']]$Enrich$MeSH_F$Term
target <- seq_len(min(100, length(negLogPval)))
negLogPval <- negLogPval[target]
term <- term[target]
p <- plot_ly(x=seq_along(negLogPval), y=~negLogPval,
type="bar", color=~negLogPval, text=term,
colors=c("#4b61ba", "gray", "#a87963", "red"))
layout(p, title="MeSH-Enrichment Analysis (F)",
   xaxis=list(title="Term"),
   yaxis=list(title="-Log(P-value)"),
   legend=list(name=""))
```

## MeSH-Enrichment Analysis (G : Phenomena and Processes)

```
negLogPval <- -log10(out.vecLR[['pattern_4_1']]$Enrich$MeSH_G$Pvalue + 1E-10)
term <- out.vecLR[['pattern_4_1']]$Enrich$MeSH_G$Term
target <- seq_len(min(100, length(negLogPval)))
negLogPval <- negLogPval[target]
term <- term[target]
p <- plot_ly(x=seq_along(negLogPval), y=~negLogPval,
type="bar", color=~negLogPval, text=term,
colors=c("#4b61ba", "gray", "#a87963", "red"))
layout(p, title="MeSH-Enrichment Analysis (G)",
   xaxis=list(title="Term"),
   yaxis=list(title="-Log(P-value)"),
   legend=list(name=""))
```

```
## Warning: textfont.color doesn't (yet) support data arrays

## Warning: textfont.color doesn't (yet) support data arrays
```

## MeSH-Enrichment Analysis (H : Disciplines and Occupations)

```
negLogPval <- -log10(out.vecLR[['pattern_4_1']]$Enrich$MeSH_H$Pvalue + 1E-10)
term <- out.vecLR[['pattern_4_1']]$Enrich$MeSH_H$Term
target <- seq_len(min(100, length(negLogPval)))
negLogPval <- negLogPval[target]
term <- term[target]
p <- plot_ly(x=seq_along(negLogPval), y=~negLogPval,
type="bar", color=~negLogPval, text=term,
colors=c("#4b61ba", "gray", "#a87963", "red"))
layout(p, title="MeSH-Enrichment Analysis (H)",
   xaxis=list(title="Term"),
   yaxis=list(title="-Log(P-value)"),
   legend=list(name=""))
```

## MeSH-Enrichment Analysis (J : Technology and Food and Beverages)

```
negLogPval <- -log10(out.vecLR[['pattern_4_1']]$Enrich$MeSH_J$Pvalue + 1E-10)
term <- out.vecLR[['pattern_4_1']]$Enrich$MeSH_J$Term
target <- seq_len(min(100, length(negLogPval)))
negLogPval <- negLogPval[target]
term <- term[target]
p <- plot_ly(x=seq_along(negLogPval), y=~negLogPval,
type="bar", color=~negLogPval, text=term,
colors=c("#4b61ba", "gray", "#a87963", "red"))
layout(p, title="MeSH-Enrichment Analysis (J)",
   xaxis=list(title="Term"),
   yaxis=list(title="-Log(P-value)"),
   legend=list(name=""))
```

```
## Warning: textfont.color doesn't (yet) support data arrays

## Warning: textfont.color doesn't (yet) support data arrays
```

## MeSH-Enrichment Analysis (L : Information Science)

```
negLogPval <- -log10(out.vecLR[['pattern_4_1']]$Enrich$MeSH_L$Pvalue + 1E-10)
term <- out.vecLR[['pattern_4_1']]$Enrich$MeSH_L$Term
target <- seq_len(min(100, length(negLogPval)))
negLogPval <- negLogPval[target]
term <- term[target]
p <- plot_ly(x=seq_along(negLogPval), y=~negLogPval,
type="bar", color=~negLogPval, text=term,
colors=c("#4b61ba", "gray", "#a87963", "red"))
layout(p, title="MeSH-Enrichment Analysis (L)",
   xaxis=list(title="Term"),
   yaxis=list(title="-Log(P-value)"),
   legend=list(name=""))
```

## MeSH-Enrichment Analysis (N : Health Care)

```
negLogPval <- -log10(out.vecLR[['pattern_4_1']]$Enrich$MeSH_N$Pvalue + 1E-10)
term <- out.vecLR[['pattern_4_1']]$Enrich$MeSH_N$Term
target <- seq_len(min(100, length(negLogPval)))
negLogPval <- negLogPval[target]
term <- term[target]
p <- plot_ly(x=seq_along(negLogPval), y=~negLogPval,
type="bar", color=~negLogPval, text=term,
colors=c("#4b61ba", "gray", "#a87963", "red"))
layout(p, title="MeSH-Enrichment Analysis (N)",
   xaxis=list(title="Term"),
   yaxis=list(title="-Log(P-value)"),
   legend=list(name=""))
```

```
## Warning: textfont.color doesn't (yet) support data arrays

## Warning: textfont.color doesn't (yet) support data arrays
```

## MeSH-Enrichment Analysis (Z : Geographical Locations)

```
negLogPval <- -log10(out.vecLR[['pattern_4_1']]$Enrich$MeSH_Z$Pvalue + 1E-10)
term <- out.vecLR[['pattern_4_1']]$Enrich$MeSH_Z$Term
target <- seq_len(min(100, length(negLogPval)))
negLogPval <- negLogPval[target]
term <- term[target]
p <- plot_ly(x=seq_along(negLogPval), y=~negLogPval,
type="bar", color=~negLogPval, text=term,
colors=c("#4b61ba", "gray", "#a87963", "red"))
layout(p, title="MeSH-Enrichment Analysis (Z)",
   xaxis=list(title="Term"),
   yaxis=list(title="-Log(P-value)"),
   legend=list(name=""))
```

## Reactome Pathway-Enrichment Analysis

```
negLogPval <- -log10(out.vecLR[['pattern_4_1']]$Enrich$Reactome$Pvalue + 1E-10)
term <- out.vecLR[['pattern_4_1']]$Enrich$Reactome$Term
target <- seq_len(min(100, length(negLogPval)))
negLogPval <- negLogPval[target]
term <- term[target]
p <- plot_ly(x=seq_along(negLogPval), y=~negLogPval,
type="bar", color=~negLogPval, text=term,
colors=c("#4b61ba", "gray", "#a87963", "red"))
layout(p, title="Reactome-Enrichment Analysis",
   xaxis=list(title="Term"),
   yaxis=list(title="-Log(P-value)"),
   legend=list(name=""))
```

```
## Warning: textfont.color doesn't (yet) support data arrays

## Warning: textfont.color doesn't (yet) support data arrays
```

## DO-Enrichment Analysis

```
negLogPval <- -log10(out.vecLR[['pattern_4_1']]$Enrich$DO$Pvalue + 1E-10)
term <- out.vecLR[['pattern_4_1']]$Enrich$DO$Term
target <- seq_len(min(100, length(negLogPval)))
negLogPval <- negLogPval[target]
term <- term[target]
p <- plot_ly(x=seq_along(negLogPval), y=~negLogPval,
type="bar", color=~negLogPval, text=term,
colors=c("#4b61ba", "gray", "#a87963", "red"))
layout(p, title="DO-Enrichment Analysis",
   xaxis=list(title="Term"),
   yaxis=list(title="-Log(P-value)"),
   legend=list(name=""))
```

```
## Warning: textfont.color doesn't (yet) support data arrays

## Warning: textfont.color doesn't (yet) support data arrays
```

## NCG-Enrichment Analysis

```
negLogPval <- -log10(out.vecLR[['pattern_4_1']]$Enrich$NCG$Pvalue + 1E-10)
term <- out.vecLR[['pattern_4_1']]$Enrich$NCG$Term
target <- seq_len(min(100, length(negLogPval)))
negLogPval <- negLogPval[target]
term <- term[target]
p <- plot_ly(x=seq_along(negLogPval), y=~negLogPval,
type="bar", color=~negLogPval, text=term,
colors=c("#4b61ba", "gray", "#a87963", "red"))
layout(p, title="NCG-Enrichment Analysis",
   xaxis=list(title="Term"),
   yaxis=list(title="-Log(P-value)"),
   legend=list(name=""))
```

```
## Warning: textfont.color doesn't (yet) support data arrays

## Warning: textfont.color doesn't (yet) support data arrays
```

## DGN-Enrichment Analysis

```
negLogPval <- -log10(out.vecLR[['pattern_4_1']]$Enrich$DGN$Pvalue + 1E-10)
term <- out.vecLR[['pattern_4_1']]$Enrich$DGN$Term
target <- seq_len(min(100, length(negLogPval)))
negLogPval <- negLogPval[target]
term <- term[target]
p <- plot_ly(x=seq_along(negLogPval), y=~negLogPval,
type="bar", color=~negLogPval, text=term,
colors=c("#4b61ba", "gray", "#a87963", "red"))
layout(p, title="DGN-Enrichment Analysis",
   xaxis=list(title="Term"),
   yaxis=list(title="-Log(P-value)"),
   legend=list(name=""))
```

```
## Warning: textfont.color doesn't (yet) support data arrays

## Warning: textfont.color doesn't (yet) support data arrays
```
