# Supplementary material for: Sctensor detects many-to-many cell–cell interactions from single cell RNA-sequencing data
Source: BMC Bioinformatics. 2023 Nov 7;24:420. doi: 10.1186/s12859-023-05490-y (PMC10631077; doi:10.1186/s12859-023-05490-y)
Supplement: Supplementary file 15 — Additional file 15. HTML report of GermlineFemale. [file 12859_2023_5490_MOESM15_ESM.zip › output/report/Human_Germline_Female/pattern_2_2.html]

Details of (2,2,) -related L-R pairs


# Details of (2,2,) -related L-R pairs

# (2,2,:) Pattern

# (2,2,) -related L-R pairs (Top38 pairs)

```
library("scTensor")
load("reanalysis.RData")
col <- .setColor("many")[6]
scTensor:::.myvisNetwork(g, col)
```

| Rank | Ligand Gene | Receptor Gene | Ligand Expression (Log10(exp + 1)) | Receptor Expression (Log10(exp + 1)) | LR-pair factor value (Percentage) | P-value (Grubbs test) | Q-value (BH method) | L-R Evidence cf. rikenbit/lrbase-workflow | PubMed |
| --- | --- | --- | --- | --- | --- | --- | --- | --- | --- |
| 1 | COL1A1 Description: COL1A1collagen type I alpha 1 chain GO: 1-61 Reactome: 1-37 UniProtKB: 1 RefEx: COL1A1 Expression Atlas: COL1A1 Single Cell Expression Atlas: COL1A1 scRNASeqDB: COL1A1 PanglaoDB: COL1A1 CMap: COL1A1 MeSH: 1-98 99-196 197-294 295-392 393-490 491-588 589-686 687-784 785-882 883-980 981-1078 1079-1176 1177-1274 1275-1372 1373-1470 1471-1568 1569-1666 1667-1764 1765-1845 | ITGA2 Description: COL1A1integrin subunit alpha 2 GO: 1-65 Reactome: 1-18 UniProtKB: 1 RefEx: COL1A1 Expression Atlas: ITGA2 Single Cell Expression Atlas: ITGA2 scRNASeqDB: ITGA2 PanglaoDB: ITGA2 CMap: ITGA2 MeSH: 1-95 96-190 191-285 286-380 381-475 476-570 571-665 666-760 761-855 856-950 951-1045 1046-1140 1141-1235 1236-1330 1331-1413 |  |  | 2.458 (12.794%) | 0 | 0 | DLRP | 1 |
| 2 | DLK1 Description: DLK1delta like non-canonical Notch ligand 1 GO: 1-9 Reactome: 1-4 UniProtKB: 1-3 RefEx: DLK1 Expression Atlas: DLK1 Single Cell Expression Atlas: DLK1 scRNASeqDB: DLK1 PanglaoDB: DLK1 CMap: DLK1 MeSH: 1-92 93-184 185-276 277-368 369-460 461-552 553-644 645-730 | NOTCH2 Description: DLK1notch receptor 2 GO: 1-63 Reactome: 1-20 UniProtKB: 1-3 RefEx: DLK1 Expression Atlas: NOTCH2 Single Cell Expression Atlas: NOTCH2 scRNASeqDB: NOTCH2 PanglaoDB: NOTCH2 CMap: NOTCH2 MeSH: 1-93 94-186 187-279 280-372 373-465 466-558 559-651 652-744 745-837 838-930 931-1023 1024-1116 1117-1203 |  |  | 2.316 (12.057%) | 0.074 | 0.116 | DLRP | 1 |
| 3 | FN1 Description: FN1fibronectin 1 GO: 1-55 Reactome: 1-49 UniProtKB: 1-6 RefEx: FN1 Expression Atlas: FN1 Single Cell Expression Atlas: FN1 scRNASeqDB: FN1 PanglaoDB: FN1 CMap: FN1 MeSH: 1-97 98-194 195-291 292-388 389-485 486-582 583-679 680-776 777-873 874-970 971-1067 1068-1164 1165-1261 1262-1358 1359-1455 1456-1552 1553-1649 1650-1746 1747-1843 1844-1940 1941-2033 | ITGA2 Description: FN1integrin subunit alpha 2 GO: 1-65 Reactome: 1-18 UniProtKB: 1 RefEx: FN1 Expression Atlas: ITGA2 Single Cell Expression Atlas: ITGA2 scRNASeqDB: ITGA2 PanglaoDB: ITGA2 CMap: ITGA2 MeSH: 1-95 96-190 191-285 286-380 381-475 476-570 571-665 666-760 761-855 856-950 951-1045 1046-1140 1141-1235 1236-1330 1331-1413 |  |  | 0.98 (5.099%) | 0.198 | 0.225 | DLRP | 1 |
| 4 | NCAM1 Description: NCAM1neural cell adhesion molecule 1 GO: 1-17 Reactome: 1-17 UniProtKB: 1-2 RefEx: NCAM1 Expression Atlas: NCAM1 Single Cell Expression Atlas: NCAM1 scRNASeqDB: NCAM1 PanglaoDB: NCAM1 CMap: NCAM1 MeSH: 1-94 95-188 189-282 283-376 377-470 471-564 565-658 659-752 753-846 847-940 941-1034 1035-1128 1129-1222 1223-1313 | ROBO1 Description: NCAM1roundabout guidance receptor 1 GO: 1-35 Reactome: 1-12 UniProtKB: 1-2 RefEx: NCAM1 Expression Atlas: ROBO1 Single Cell Expression Atlas: ROBO1 scRNASeqDB: ROBO1 PanglaoDB: ROBO1 CMap: ROBO1 MeSH: 1-95 96-190 191-285 286-380 381-475 476-570 571-659 |  |  | 0.841 (4.379%) | 0.014 | 0.039 | DLRP | 1 |
| 5 | SLIT2 Description: SLIT2slit guidance ligand 2 GO: 1-53 Reactome: 1-11 UniProtKB: 1 RefEx: SLIT2 Expression Atlas: SLIT2 Single Cell Expression Atlas: SLIT2 scRNASeqDB: SLIT2 PanglaoDB: SLIT2 CMap: SLIT2 MeSH: 1-89 90-178 179-267 268-356 357-445 446-534 535-623 624-712 713-801 | ROBO1 Description: SLIT2roundabout guidance receptor 1 GO: 1-35 Reactome: 1-12 UniProtKB: 1-2 RefEx: SLIT2 Expression Atlas: ROBO1 Single Cell Expression Atlas: ROBO1 scRNASeqDB: ROBO1 PanglaoDB: ROBO1 CMap: ROBO1 MeSH: 1-95 96-190 191-285 286-380 381-475 476-570 571-659 |  |  | 0.756 (3.933%) | 0.001 | 0.005 | DLRP | 1 |
| 6 | SEMA6D Description: SEMA6Dsemaphorin 6D GO: 1-15 Reactome: 1-5 UniProtKB: 1-2 RefEx: SEMA6D Expression Atlas: SEMA6D Single Cell Expression Atlas: SEMA6D scRNASeqDB: SEMA6D PanglaoDB: SEMA6D CMap: SEMA6D MeSH: 1-96 97-191 | KDR Description: SEMA6Dkinase insert domain receptor GO: 1-70 Reactome: 1-14 UniProtKB: 1-2 RefEx: SEMA6D Expression Atlas: KDR Single Cell Expression Atlas: KDR scRNASeqDB: KDR PanglaoDB: KDR CMap: KDR MeSH: 1-97 98-194 195-291 292-388 389-485 486-582 583-679 680-776 777-873 874-970 971-1067 1068-1164 1165-1261 1262-1358 1359-1455 1456-1552 1553-1649 1650-1746 1747-1843 1844-1940 1941-2037 2038-2113 |  |  | 0.746 (3.884%) | 0.337 | 0.355 | DLRP | 1 |
| 7 | VEGFA Description: VEGFAvascular endothelial growth factor A GO: 1-62 63-123 Reactome: 1-28 UniProtKB: 1-9 RefEx: VEGFA Expression Atlas: VEGFA Single Cell Expression Atlas: VEGFA scRNASeqDB: VEGFA PanglaoDB: VEGFA CMap: VEGFA MeSH: 1-100 101-200 201-300 301-400 401-500 501-600 601-700 701-800 801-900 901-1000 1001-1100 1101-1200 1201-1300 1301-1400 1401-1500 1501-1600 1601-1700 1701-1800 1801-1900 1901-2000 2001-2100 2101-2200 2201-2300 2301-2400 2401-2500 2501-2600 2601-2700 2701-2800 2801-2900 2901-3000 3001-3100 3101-3200 3201-3300 3301-3400 3401-3500 3501-3600 3601-3700 3701-3800 3801-3900 3901-4000 4001-4100 4101-4200 4201-4300 4301-4400 4401-4500 4501-4600 4601-4700 4701-4800 4801-4900 4901-5000 5001-5100 5101-5200 5201-5300 5301-5400 5401-5500 5501-5556 | KDR Description: VEGFAkinase insert domain receptor GO: 1-70 Reactome: 1-14 UniProtKB: 1-2 RefEx: VEGFA Expression Atlas: KDR Single Cell Expression Atlas: KDR scRNASeqDB: KDR PanglaoDB: KDR CMap: KDR MeSH: 1-97 98-194 195-291 292-388 389-485 486-582 583-679 680-776 777-873 874-970 971-1067 1068-1164 1165-1261 1262-1358 1359-1455 1456-1552 1553-1649 1650-1746 1747-1843 1844-1940 1941-2037 2038-2113 |  |  | 0.713 (3.71%) | 0.106 | 0.148 | DLRP | 1 |
| 8 | VEGFA Description: VEGFAvascular endothelial growth factor A GO: 1-62 63-123 Reactome: 1-28 UniProtKB: 1-9 RefEx: VEGFA Expression Atlas: VEGFA Single Cell Expression Atlas: VEGFA scRNASeqDB: VEGFA PanglaoDB: VEGFA CMap: VEGFA MeSH: 1-100 101-200 201-300 301-400 401-500 501-600 601-700 701-800 801-900 901-1000 1001-1100 1101-1200 1201-1300 1301-1400 1401-1500 1501-1600 1601-1700 1701-1800 1801-1900 1901-2000 2001-2100 2101-2200 2201-2300 2301-2400 2401-2500 2501-2600 2601-2700 2701-2800 2801-2900 2901-3000 3001-3100 3101-3200 3201-3300 3301-3400 3401-3500 3501-3600 3601-3700 3701-3800 3801-3900 3901-4000 4001-4100 4101-4200 4201-4300 4301-4400 4401-4500 4501-4600 4601-4700 4701-4800 4801-4900 4901-5000 5001-5100 5101-5200 5201-5300 5301-5400 5401-5500 5501-5556 | NRP1 Description: VEGFAneuropilin 1 GO: 1-58 59-116 Reactome: 1-15 UniProtKB: 1-6 RefEx: VEGFA Expression Atlas: NRP1 Single Cell Expression Atlas: NRP1 scRNASeqDB: NRP1 PanglaoDB: NRP1 CMap: NRP1 MeSH: 1-96 97-192 193-288 289-384 385-480 481-576 577-672 673-768 769-864 865-960 961-1056 1057-1147 |  |  | 0.649 (3.38%) | 0.005 | 0.019 | DLRP | 1 |
| 9 | GAS6 Description: GAS6growth arrest specific 6 GO: 1-76 Reactome: 1-14 UniProtKB: 1 RefEx: GAS6 Expression Atlas: GAS6 Single Cell Expression Atlas: GAS6 scRNASeqDB: GAS6 PanglaoDB: GAS6 CMap: GAS6 MeSH: 1-98 99-196 197-294 295-392 393-490 491-588 589-686 687-778 | AXL Description: GAS6AXL receptor tyrosine kinase GO: 1-60 Reactome: 1-4 UniProtKB: 1-2 RefEx: GAS6 Expression Atlas: AXL Single Cell Expression Atlas: AXL scRNASeqDB: AXL PanglaoDB: AXL CMap: AXL MeSH: 1-93 94-186 187-279 280-372 373-465 466-558 559-651 652-744 745-837 838-930 931-1013 |  |  | 0.631 (3.287%) | 0.087 | 0.126 | DLRP | 1 |
| 10 | CTF1 Description: CTF1cardiotrophin 1 GO: 1-13 Reactome: 1-5 UniProtKB: 1 STRING: 1 2 RefEx: CTF1 Expression Atlas: CTF1 Single Cell Expression Atlas: CTF1 scRNASeqDB: CTF1 PanglaoDB: CTF1 CMap: CTF1 MeSH: 1-100 101-200 201-300 301-398 | IL6ST Description: CTF1interleukin 6 cytokine family signal transducer GO: 1-50 Reactome: 1-15 UniProtKB: 1-3 RefEx: CTF1 Expression Atlas: IL6ST Single Cell Expression Atlas: IL6ST scRNASeqDB: IL6ST PanglaoDB: IL6ST CMap: IL6ST MeSH: 1-100 101-200 201-300 301-400 401-500 501-600 601-700 701-800 801-900 901-1000 1001-1100 1101-1200 1201-1300 1301-1400 1401-1500 1501-1600 1601-1692 |  |  | 0.583 (3.036%) | 0 | 0 | DLRP | 1 |
| 11 | EFNA1 Description: EFNA1ephrin A1 GO: 1-34 Reactome: 1-6 UniProtKB: 1 RefEx: EFNA1 Expression Atlas: EFNA1 Single Cell Expression Atlas: EFNA1 scRNASeqDB: EFNA1 PanglaoDB: EFNA1 CMap: EFNA1 MeSH: 1-94 95-188 189-282 283-376 377-466 | EPHA4 Description: EFNA1EPH receptor A4 GO: 1-77 Reactome: 1-6 UniProtKB: 1-2 RefEx: EFNA1 Expression Atlas: EPHA4 Single Cell Expression Atlas: EPHA4 scRNASeqDB: EPHA4 PanglaoDB: EPHA4 CMap: EPHA4 MeSH: 1-93 94-186 187-279 280-372 373-465 466-557 |  |  | 0.473 (2.461%) | 0.065 | 0.107 | DLRP | 1 |
| 12 | INHA Description: INHAinhibin subunit alpha GO: 1-36 Reactome: 1-4 UniProtKB: 1 STRING: 1 RefEx: INHA Expression Atlas: INHA Single Cell Expression Atlas: INHA scRNASeqDB: INHA PanglaoDB: INHA CMap: INHA MeSH: 1-97 98-194 195-291 292-388 389-485 486-582 583-675 | TGFBR3 Description: INHAtransforming growth factor beta receptor 3 GO: 1-58 UniProtKB: 1-2 RefEx: INHA Expression Atlas: TGFBR3 Single Cell Expression Atlas: TGFBR3 scRNASeqDB: TGFBR3 PanglaoDB: TGFBR3 CMap: TGFBR3 MeSH: 1-91 92-182 183-273 274-364 365-455 456-546 547-637 638-726 |  |  | 0.47 (2.445%) | 0.001 | 0.006 | DLRP | 1 |
| 13 | SEMA5A Description: SEMA5Asemaphorin 5A GO: 1-34 Reactome: 1-14 UniProtKB: 1-2 RefEx: SEMA5A Expression Atlas: SEMA5A Single Cell Expression Atlas: SEMA5A scRNASeqDB: SEMA5A PanglaoDB: SEMA5A CMap: SEMA5A MeSH: 1-88 89-176 177-264 265-349 | MET Description: SEMA5AMET proto-oncogene, receptor tyrosine kinase GO: 1-43 Reactome: 1-39 UniProtKB: 1-5 RefEx: SEMA5A Expression Atlas: MET Single Cell Expression Atlas: MET scRNASeqDB: MET PanglaoDB: MET CMap: MET MeSH: 1-99 100-198 199-297 298-396 397-495 496-594 595-693 694-792 793-891 892-990 991-1089 1090-1188 1189-1287 1288-1386 1387-1485 1486-1584 1585-1683 1684-1782 1783-1881 1882-1980 1981-2066 |  |  | 0.461 (2.398%) | 0.126 | 0.164 | DLRP | 1 |
| 14 | COL18A1 Description: COL18A1collagen type XVIII alpha 1 chain GO: 1-20 Reactome: 1-10 UniProtKB: 1-3 RefEx: COL18A1 Expression Atlas: COL18A1 Single Cell Expression Atlas: COL18A1 scRNASeqDB: COL18A1 PanglaoDB: COL18A1 CMap: COL18A1 MeSH: 1-96 97-192 193-288 289-384 385-480 481-576 577-672 673-768 769-864 865-960 961-1054 | GPC1 Description: COL18A1glypican 1 GO: 1-26 Reactome: 1-28 UniProtKB: 1-2 STRING: 1 2 3 4 5 6 RefEx: COL18A1 Expression Atlas: GPC1 Single Cell Expression Atlas: GPC1 scRNASeqDB: GPC1 PanglaoDB: GPC1 CMap: GPC1 MeSH: 1-95 96-190 191-285 286-380 381-472 |  |  | 0.43 (2.24%) | 0.001 | 0.004 | DLRP | 1 |
| 15 | WNT5A Description: WNT5AWnt family member 5A GO: 1-83 84-165 Reactome: 1-23 UniProtKB: 1-3 RefEx: WNT5A Expression Atlas: WNT5A Single Cell Expression Atlas: WNT5A scRNASeqDB: WNT5A PanglaoDB: WNT5A CMap: WNT5A MeSH: 1-100 101-200 201-300 301-400 401-500 501-600 601-700 701-800 801-900 901-1000 1001-1100 1101-1200 1201-1300 1301-1399 | FZD7 Description: WNT5Afrizzled class receptor 7 GO: 1-33 Reactome: 1-13 UniProtKB: 1 STRING: 1 RefEx: WNT5A Expression Atlas: FZD7 Single Cell Expression Atlas: FZD7 scRNASeqDB: FZD7 PanglaoDB: FZD7 CMap: FZD7 MeSH: 1-96 97-192 193-288 289-384 385-480 |  |  | 0.43 (2.239%) | 0 | 0 | DLRP | 1 |
| 16 | MDK Description: MDKmidkine GO: 1-69 Reactome: 1-6 UniProtKB: 1 RefEx: MDK Expression Atlas: MDK Single Cell Expression Atlas: MDK scRNASeqDB: MDK PanglaoDB: MDK CMap: MDK MeSH: 1-93 94-186 187-279 280-372 373-465 466-558 559-651 652-744 745-837 838-930 | PTPRZ1 Description: MDKprotein tyrosine phosphatase receptor type Z1 GO: 1-21 Reactome: 1-7 UniProtKB: 1-2 RefEx: MDK Expression Atlas: PTPRZ1 Single Cell Expression Atlas: PTPRZ1 scRNASeqDB: PTPRZ1 PanglaoDB: PTPRZ1 CMap: PTPRZ1 MeSH: 1-96 97-192 193-288 289-384 385-480 481-576 577-668 |  |  | 0.424 (2.206%) | 0.037 | 0.079 | DLRP | 1 |
| 17 | SLIT2 Description: SLIT2slit guidance ligand 2 GO: 1-53 Reactome: 1-11 UniProtKB: 1 RefEx: SLIT2 Expression Atlas: SLIT2 Single Cell Expression Atlas: SLIT2 scRNASeqDB: SLIT2 PanglaoDB: SLIT2 CMap: SLIT2 MeSH: 1-89 90-178 179-267 268-356 357-445 446-534 535-623 624-712 713-801 | GPC1 Description: SLIT2glypican 1 GO: 1-26 Reactome: 1-28 UniProtKB: 1-2 STRING: 1 2 3 4 5 6 RefEx: SLIT2 Expression Atlas: GPC1 Single Cell Expression Atlas: GPC1 scRNASeqDB: GPC1 PanglaoDB: GPC1 CMap: GPC1 MeSH: 1-95 96-190 191-285 286-380 381-472 |  |  | 0.357 (1.857%) | 0.072 | 0.116 | DLRP | 1 |
| 18 | PDGFC Description: PDGFCplatelet derived growth factor C GO: 1-32 Reactome: 1-3 UniProtKB: 1 RefEx: PDGFC Expression Atlas: PDGFC Single Cell Expression Atlas: PDGFC scRNASeqDB: PDGFC PanglaoDB: PDGFC CMap: PDGFC MeSH: 1-88 89-176 177-264 265-352 353-440 441-528 529-614 | KDR Description: PDGFCkinase insert domain receptor GO: 1-70 Reactome: 1-14 UniProtKB: 1-2 RefEx: PDGFC Expression Atlas: KDR Single Cell Expression Atlas: KDR scRNASeqDB: KDR PanglaoDB: KDR CMap: KDR MeSH: 1-97 98-194 195-291 292-388 389-485 486-582 583-679 680-776 777-873 874-970 971-1067 1068-1164 1165-1261 1262-1358 1359-1455 1456-1552 1553-1649 1650-1746 1747-1843 1844-1940 1941-2037 2038-2113 |  |  | 0.352 (1.834%) | 0.053 | 0.093 | DLRP | 1 |
| 19 | NPY Description: NPYneuropeptide Y GO: 1-24 Reactome: 1-12 UniProtKB: 1-2 STRING: 1 2 3 RefEx: NPY Expression Atlas: NPY Single Cell Expression Atlas: NPY scRNASeqDB: NPY PanglaoDB: NPY CMap: NPY MeSH: 1-100 101-200 201-300 301-400 401-500 501-600 601-700 701-800 801-900 901-1000 1001-1100 1101-1200 1201-1300 1301-1400 1401-1500 1501-1600 1601-1700 1701-1800 1801-1900 1901-2000 2001-2100 2101-2200 2201-2300 2301-2400 2401-2500 2501-2600 2601-2700 2701-2800 2801-2900 2901-3000 3001-3100 3101-3200 3201-3300 3301-3400 3401-3500 3501-3600 3601-3700 3701-3800 3801-3900 3901-3983 | NPY1R Description: NPYneuropeptide Y receptor Y1 GO: 1-9 Reactome: 1-7 UniProtKB: 1 STRING: 1 2 3 4 5 6 7 RefEx: NPY Expression Atlas: NPY1R Single Cell Expression Atlas: NPY1R scRNASeqDB: NPY1R PanglaoDB: NPY1R CMap: NPY1R MeSH: 1-98 99-196 197-294 295-392 393-489 |  |  | 0.339 (1.767%) | 0.339 | 0.355 | DLRP | 1 |
| 20 | CTF1 Description: CTF1cardiotrophin 1 GO: 1-13 Reactome: 1-5 UniProtKB: 1 STRING: 1 2 RefEx: CTF1 Expression Atlas: CTF1 Single Cell Expression Atlas: CTF1 scRNASeqDB: CTF1 PanglaoDB: CTF1 CMap: CTF1 MeSH: 1-100 101-200 201-300 301-398 | LIFR Description: CTF1LIF receptor subunit alpha GO: 1-20 Reactome: 1-10 UniProtKB: 1-2 RefEx: CTF1 Expression Atlas: LIFR Single Cell Expression Atlas: LIFR scRNASeqDB: LIFR PanglaoDB: LIFR CMap: LIFR MeSH: 1-99 100-198 199-297 298-396 397-495 496-594 595-693 694-792 793-891 892-990 |  |  | 0.334 (1.74%) | 0.161 | 0.201 | DLRP | 1 |
| 21 | EFNA5 Description: EFNA5ephrin A5 GO: 1-23 Reactome: 1-6 UniProtKB: 1-2 RefEx: EFNA5 Expression Atlas: EFNA5 Single Cell Expression Atlas: EFNA5 scRNASeqDB: EFNA5 PanglaoDB: EFNA5 CMap: EFNA5 MeSH: 1-100 101-200 201-300 301-398 | EPHA4 Description: EFNA5EPH receptor A4 GO: 1-77 Reactome: 1-6 UniProtKB: 1-2 RefEx: EFNA5 Expression Atlas: EPHA4 Single Cell Expression Atlas: EPHA4 scRNASeqDB: EPHA4 PanglaoDB: EPHA4 CMap: EPHA4 MeSH: 1-93 94-186 187-279 280-372 373-465 466-557 |  |  | 0.308 (1.601%) | 0.046 | 0.088 | DLRP | 1 |
| 22 | CRLF1 Description: CRLF1cytokine receptor like factor 1 GO: 1-17 Reactome: 1-7 UniProtKB: 1 RefEx: CRLF1 Expression Atlas: CRLF1 Single Cell Expression Atlas: CRLF1 scRNASeqDB: CRLF1 PanglaoDB: CRLF1 CMap: CRLF1 MeSH: 1-74 75-148 149-222 | IL6ST Description: CRLF1interleukin 6 cytokine family signal transducer GO: 1-50 Reactome: 1-15 UniProtKB: 1-3 RefEx: CRLF1 Expression Atlas: IL6ST Single Cell Expression Atlas: IL6ST scRNASeqDB: IL6ST PanglaoDB: IL6ST CMap: IL6ST MeSH: 1-100 101-200 201-300 301-400 401-500 501-600 601-700 701-800 801-900 901-1000 1001-1100 1101-1200 1201-1300 1301-1400 1401-1500 1501-1600 1601-1692 |  |  | 0.307 (1.599%) | 0.006 | 0.021 | DLRP | 1 |
| 23 | RSPO3 Description: RSPO3R-spondin 3 GO: 1-12 Reactome: 1-9 UniProtKB: 1 RefEx: RSPO3 Expression Atlas: RSPO3 Single Cell Expression Atlas: RSPO3 scRNASeqDB: RSPO3 PanglaoDB: RSPO3 CMap: RSPO3 MeSH: 1-100 101-200 201-300 301-398 | LGR4 Description: RSPO3leucine rich repeat containing G protein-coupled receptor 4 GO: 1-31 Reactome: 1-4 UniProtKB: 1-2 RefEx: RSPO3 Expression Atlas: LGR4 Single Cell Expression Atlas: LGR4 scRNASeqDB: LGR4 PanglaoDB: LGR4 CMap: LGR4 MeSH: 1-97 98-194 195-291 292-387 |  |  | 0.291 (1.517%) | 0.01 | 0.031 | DLRP | 1 |
| 24 | EFNA5 Description: EFNA5ephrin A5 GO: 1-23 Reactome: 1-6 UniProtKB: 1-2 RefEx: EFNA5 Expression Atlas: EFNA5 Single Cell Expression Atlas: EFNA5 scRNASeqDB: EFNA5 PanglaoDB: EFNA5 CMap: EFNA5 MeSH: 1-100 101-200 201-300 301-398 | EPHB2 Description: EFNA5EPH receptor B2 GO: 1-72 Reactome: 1-8 UniProtKB: 1-4 RefEx: EFNA5 Expression Atlas: EPHB2 Single Cell Expression Atlas: EPHB2 scRNASeqDB: EPHB2 PanglaoDB: EPHB2 CMap: EPHB2 MeSH: 1-99 100-198 199-297 298-396 397-495 496-594 595-693 694-792 793-885 |  |  | 0.278 (1.448%) | 0 | 0 | DLRP | 1 |
| 25 | ADM Description: ADMadrenomedullin GO: 1-47 Reactome: 1-13 UniProtKB: 1 RefEx: ADM Expression Atlas: ADM Single Cell Expression Atlas: ADM scRNASeqDB: ADM PanglaoDB: ADM CMap: ADM MeSH: 1-99 100-198 199-297 298-396 397-495 496-594 595-693 694-792 793-891 892-990 991-1089 1090-1188 1189-1287 1288-1386 1387-1485 1486-1584 1585-1682 | CALCRL Description: ADMcalcitonin receptor like receptor GO: 1-30 Reactome: 1-13 UniProtKB: 1-2 RefEx: ADM Expression Atlas: CALCRL Single Cell Expression Atlas: CALCRL scRNASeqDB: CALCRL PanglaoDB: CALCRL CMap: CALCRL MeSH: 1-98 99-196 197-294 295-392 393-488 |  |  | 0.246 (1.283%) | 0.024 | 0.057 | DLRP | 1 |
| 26 | RELN Description: RELNreelin GO: 1-55 Reactome: 1-4 UniProtKB: 1 RefEx: RELN Expression Atlas: RELN Single Cell Expression Atlas: RELN scRNASeqDB: RELN PanglaoDB: RELN CMap: RELN MeSH: 1-95 96-190 191-285 286-380 381-475 476-570 571-665 666-760 761-855 856-950 951-1045 1046-1140 1141-1235 1236-1330 1331-1416 | VLDLR Description: RELNvery low density lipoprotein receptor GO: 1-29 Reactome: 1-9 UniProtKB: 1-2 RefEx: RELN Expression Atlas: VLDLR Single Cell Expression Atlas: VLDLR scRNASeqDB: VLDLR PanglaoDB: VLDLR CMap: VLDLR MeSH: 1-88 89-176 177-264 265-352 353-440 441-528 529-616 617-702 |  |  | 0.239 (1.245%) | 0.235 | 0.257 | DLRP | 1 |
| 27 | SLIT2 Description: SLIT2slit guidance ligand 2 GO: 1-53 Reactome: 1-11 UniProtKB: 1 RefEx: SLIT2 Expression Atlas: SLIT2 Single Cell Expression Atlas: SLIT2 scRNASeqDB: SLIT2 PanglaoDB: SLIT2 CMap: SLIT2 MeSH: 1-89 90-178 179-267 268-356 357-445 446-534 535-623 624-712 713-801 | ROBO2 Description: SLIT2roundabout guidance receptor 2 GO: 1-30 Reactome: 1-8 UniProtKB: 1 RefEx: SLIT2 Expression Atlas: ROBO2 Single Cell Expression Atlas: ROBO2 scRNASeqDB: ROBO2 PanglaoDB: ROBO2 CMap: ROBO2 MeSH: 1-91 92-182 183-273 274-363 |  |  | 0.198 (1.032%) | 0.013 | 0.039 | DLRP | 1 |
| 28 | NRG1 Description: NRG1neuregulin 1 GO: 1-62 Reactome: 1-37 UniProtKB: 1-18 RefEx: NRG1 Expression Atlas: NRG1 Single Cell Expression Atlas: NRG1 scRNASeqDB: NRG1 PanglaoDB: NRG1 CMap: NRG1 MeSH: 1-98 99-196 197-294 295-392 393-490 491-588 589-686 687-784 785-882 883-980 981-1078 1079-1176 1177-1274 1275-1372 1373-1470 1471-1568 | ERBB4 Description: NRG1erb-b2 receptor tyrosine kinase 4 GO: 1-56 Reactome: 1-39 UniProtKB: 1 RefEx: NRG1 Expression Atlas: ERBB4 Single Cell Expression Atlas: ERBB4 scRNASeqDB: ERBB4 PanglaoDB: ERBB4 CMap: ERBB4 MeSH: 1-100 101-200 201-300 301-400 401-500 501-600 601-700 701-800 801-900 901-1000 1001-1100 1101-1200 1201-1300 1301-1400 1401-1500 1501-1600 1601-1698 |  |  | 0.197 (1.027%) | 0.083 | 0.124 | DLRP | 1 |
| 29 | EFNB1 Description: EFNB1ephrin B1 GO: 1-17 Reactome: 1-7 UniProtKB: 1 STRING: 1 RefEx: EFNB1 Expression Atlas: EFNB1 Single Cell Expression Atlas: EFNB1 scRNASeqDB: EFNB1 PanglaoDB: EFNB1 CMap: EFNB1 MeSH: 1-89 90-178 179-267 268-356 357-445 446-534 535-620 | EPHA4 Description: EFNB1EPH receptor A4 GO: 1-77 Reactome: 1-6 UniProtKB: 1-2 RefEx: EFNB1 Expression Atlas: EPHA4 Single Cell Expression Atlas: EPHA4 scRNASeqDB: EPHA4 PanglaoDB: EPHA4 CMap: EPHA4 MeSH: 1-93 94-186 187-279 280-372 373-465 466-557 |  |  | 0.188 (0.979%) | 0 | 0.001 | DLRP | 1 |
| 30 | BMP2 Description: BMP2bone morphogenetic protein 2 GO: 1-57 58-113 Reactome: 1-11 UniProtKB: 1-2 STRING: 1 RefEx: BMP2 Expression Atlas: BMP2 Single Cell Expression Atlas: BMP2 scRNASeqDB: BMP2 PanglaoDB: BMP2 CMap: BMP2 MeSH: 1-98 99-196 197-294 295-392 393-490 491-588 589-686 687-784 785-882 883-980 981-1078 1079-1176 1177-1274 1275-1372 1373-1470 1471-1568 1569-1666 1667-1764 1765-1862 1863-1960 1961-2058 2059-2156 2157-2254 2255-2352 2353-2450 2451-2548 2549-2646 2647-2742 | BMPR2 Description: BMP2bone morphogenetic protein receptor type 2 GO: 1-91 Reactome: 1-3 UniProtKB: 1 RefEx: BMP2 Expression Atlas: BMPR2 Single Cell Expression Atlas: BMPR2 scRNASeqDB: BMPR2 PanglaoDB: BMPR2 CMap: BMPR2 MeSH: 1-100 101-200 201-300 301-400 401-500 501-600 601-700 701-800 801-900 901-1000 1001-1098 |  |  | 0.177 (0.92%) | 0.111 | 0.151 | DLRP | 1 |
| 31 | CRLF1 Description: CRLF1cytokine receptor like factor 1 GO: 1-17 Reactome: 1-7 UniProtKB: 1 RefEx: CRLF1 Expression Atlas: CRLF1 Single Cell Expression Atlas: CRLF1 scRNASeqDB: CRLF1 PanglaoDB: CRLF1 CMap: CRLF1 MeSH: 1-74 75-148 149-222 | LIFR Description: CRLF1LIF receptor subunit alpha GO: 1-20 Reactome: 1-10 UniProtKB: 1-2 RefEx: CRLF1 Expression Atlas: LIFR Single Cell Expression Atlas: LIFR scRNASeqDB: LIFR PanglaoDB: LIFR CMap: LIFR MeSH: 1-99 100-198 199-297 298-396 397-495 496-594 595-693 694-792 793-891 892-990 |  |  | 0.176 (0.916%) | 0.172 | 0.208 | DLRP | 1 |
| 32 | EFNB1 Description: EFNB1ephrin B1 GO: 1-17 Reactome: 1-7 UniProtKB: 1 STRING: 1 RefEx: EFNB1 Expression Atlas: EFNB1 Single Cell Expression Atlas: EFNB1 scRNASeqDB: EFNB1 PanglaoDB: EFNB1 CMap: EFNB1 MeSH: 1-89 90-178 179-267 268-356 357-445 446-534 535-620 | EPHB2 Description: EFNB1EPH receptor B2 GO: 1-72 Reactome: 1-8 UniProtKB: 1-4 RefEx: EFNB1 Expression Atlas: EPHB2 Single Cell Expression Atlas: EPHB2 scRNASeqDB: EPHB2 PanglaoDB: EPHB2 CMap: EPHB2 MeSH: 1-99 100-198 199-297 298-396 397-495 496-594 595-693 694-792 793-885 |  |  | 0.17 (0.885%) | 0.176 | 0.208 | DLRP | 1 |
| 33 | COL4A1 Description: COL4A1collagen type IV alpha 1 chain GO: 1-22 Reactome: 1-24 UniProtKB: 1-3 RefEx: COL4A1 Expression Atlas: COL4A1 Single Cell Expression Atlas: COL4A1 scRNASeqDB: COL4A1 PanglaoDB: COL4A1 CMap: COL4A1 MeSH: 1-94 95-188 189-282 283-376 377-470 471-564 565-658 659-752 753-846 847-940 941-1032 | ITGA1 Description: COL4A1integrin subunit alpha 1 GO: 1-29 Reactome: 1-14 UniProtKB: 1 RefEx: COL4A1 Expression Atlas: ITGA1 Single Cell Expression Atlas: ITGA1 scRNASeqDB: ITGA1 PanglaoDB: ITGA1 CMap: ITGA1 MeSH: 1-91 92-182 183-273 274-364 365-455 456-546 547-637 638-728 729-817 |  |  | 0.167 (0.868%) | 0.022 | 0.054 | DLRP | 1 |
| 34 | TGFB2 Description: TGFB2transforming growth factor beta 2 GO: 1-57 58-113 Reactome: 1-8 UniProtKB: 1-2 RefEx: TGFB2 Expression Atlas: TGFB2 Single Cell Expression Atlas: TGFB2 scRNASeqDB: TGFB2 PanglaoDB: TGFB2 CMap: TGFB2 MeSH: 1-97 98-194 195-291 292-388 389-485 486-582 583-679 680-776 777-873 874-970 971-1067 1068-1164 1165-1261 1262-1358 1359-1455 1456-1552 1553-1649 1650-1732 | TGFBR3 Description: TGFB2transforming growth factor beta receptor 3 GO: 1-58 UniProtKB: 1-2 RefEx: TGFB2 Expression Atlas: TGFBR3 Single Cell Expression Atlas: TGFBR3 scRNASeqDB: TGFBR3 PanglaoDB: TGFBR3 CMap: TGFBR3 MeSH: 1-91 92-182 183-273 274-364 365-455 456-546 547-637 638-726 |  |  | 0.147 (0.764%) | 0 | 0 | DLRP | 1 |
| 35 | PTN Description: PTNpleiotrophin GO: 1-89 Reactome: 1-3 UniProtKB: 1-2 RefEx: PTN Expression Atlas: PTN Single Cell Expression Atlas: PTN scRNASeqDB: PTN PanglaoDB: PTN CMap: PTN MeSH: 1-97 98-194 195-291 292-388 389-485 486-582 583-679 680-776 777-873 874-970 971-1067 1068-1164 1165-1261 1262-1354 | PTPRZ1 Description: PTNprotein tyrosine phosphatase receptor type Z1 GO: 1-21 Reactome: 1-7 UniProtKB: 1-2 RefEx: PTN Expression Atlas: PTPRZ1 Single Cell Expression Atlas: PTPRZ1 scRNASeqDB: PTPRZ1 PanglaoDB: PTPRZ1 CMap: PTPRZ1 MeSH: 1-96 97-192 193-288 289-384 385-480 481-576 577-668 |  |  | 0.127 (0.66%) | 1 | 1 | DLRP | 1 |
| 36 | NCAM1 Description: NCAM1neural cell adhesion molecule 1 GO: 1-17 Reactome: 1-17 UniProtKB: 1-2 RefEx: NCAM1 Expression Atlas: NCAM1 Single Cell Expression Atlas: NCAM1 scRNASeqDB: NCAM1 PanglaoDB: NCAM1 CMap: NCAM1 MeSH: 1-94 95-188 189-282 283-376 377-470 471-564 565-658 659-752 753-846 847-940 941-1034 1035-1128 1129-1222 1223-1313 | ROBO3 Description: NCAM1roundabout guidance receptor 3 GO: 1-9 Reactome: 1-7 UniProtKB: 1-2 RefEx: NCAM1 Expression Atlas: ROBO3 Single Cell Expression Atlas: ROBO3 scRNASeqDB: ROBO3 PanglaoDB: ROBO3 CMap: ROBO3 MeSH: 1-89 90-178 179-265 |  |  | 0.126 (0.656%) | 0.048 | 0.089 | DLRP | 1 |
| 37 | APOE Description: APOEapolipoprotein E GO: 1-74 75-147 Reactome: 1-34 UniProtKB: 1-2 RefEx: APOE Expression Atlas: APOE Single Cell Expression Atlas: APOE scRNASeqDB: APOE PanglaoDB: APOE CMap: APOE MeSH: 1-99 100-198 199-297 298-396 397-495 496-594 595-693 694-792 793-891 892-990 991-1089 1090-1188 1189-1287 1288-1386 1387-1485 1486-1584 1585-1683 1684-1782 1783-1881 1882-1980 1981-2079 2080-2178 2179-2277 2278-2376 2377-2475 2476-2574 2575-2673 2674-2772 2773-2871 2872-2970 2971-3069 3070-3168 3169-3267 3268-3366 3367-3465 3466-3564 3565-3663 3664-3762 3763-3861 3862-3960 3961-4059 4060-4158 4159-4257 4258-4338 | VLDLR Description: APOEvery low density lipoprotein receptor GO: 1-29 Reactome: 1-9 UniProtKB: 1-2 RefEx: APOE Expression Atlas: VLDLR Single Cell Expression Atlas: VLDLR scRNASeqDB: VLDLR PanglaoDB: VLDLR CMap: VLDLR MeSH: 1-88 89-176 177-264 265-352 353-440 441-528 529-616 617-702 |  |  | 0.12 (0.624%) | 0.039 | 0.08 | DLRP | 1 |
| 38 | RSPO1 Description: RSPO1R-spondin 1 GO: 1-11 Reactome: 1-4 UniProtKB: 1 RefEx: RSPO1 Expression Atlas: RSPO1 Single Cell Expression Atlas: RSPO1 scRNASeqDB: RSPO1 PanglaoDB: RSPO1 CMap: RSPO1 MeSH: 1-100 101-200 201-300 301-399 | LGR4 Description: RSPO1leucine rich repeat containing G protein-coupled receptor 4 GO: 1-31 Reactome: 1-4 UniProtKB: 1-2 RefEx: RSPO1 Expression Atlas: LGR4 Single Cell Expression Atlas: LGR4 scRNASeqDB: LGR4 PanglaoDB: LGR4 CMap: LGR4 MeSH: 1-97 98-194 195-291 292-387 |  |  | 0.115 (0.6%) | 0.001 | 0.005 | DLRP | 1 |

# (2,2,) -related Enrichment Analysis

```
load("reanalysis.RData")
library("plotly")
```

## MeSH-Enrichment Analysis (A : Anatomy)

```
negLogPval <- -log10(out.vecLR[['pattern_2_2']]$Enrich$MeSH_A$Pvalue + 1E-10)
term <- out.vecLR[['pattern_2_2']]$Enrich$MeSH_A$Term
target <- seq_len(min(100, length(negLogPval)))
negLogPval <- negLogPval[target]
term <- term[target]
p <- plot_ly(x=seq_along(negLogPval), y=~negLogPval,
type="bar", color=~negLogPval, text=term,
colors=c("#4b61ba", "gray", "#a87963", "red"))
layout(p, title="MeSH-Enrichment Analysis (A)",
   xaxis=list(title="Term"),
   yaxis=list(title="-Log(P-value)"),
   legend=list(name=""))
```

```
## Warning: textfont.color doesn't (yet) support data arrays

## Warning: textfont.color doesn't (yet) support data arrays
```

## MeSH-Enrichment Analysis (B : Organisms)

```
negLogPval <- -log10(out.vecLR[['pattern_2_2']]$Enrich$MeSH_B$Pvalue + 1E-10)
term <- out.vecLR[['pattern_2_2']]$Enrich$MeSH_B$Term
target <- seq_len(min(100, length(negLogPval)))
negLogPval <- negLogPval[target]
term <- term[target]
p <- plot_ly(x=seq_along(negLogPval), y=~negLogPval,
type="bar", color=~negLogPval, text=term,
colors=c("#4b61ba", "gray", "#a87963", "red"))
layout(p, title="MeSH-Enrichment Analysis (B)",
   xaxis=list(title="Term"),
   yaxis=list(title="-Log(P-value)"),
   legend=list(name=""))
```

```
## Warning: textfont.color doesn't (yet) support data arrays

## Warning: textfont.color doesn't (yet) support data arrays
```

## MeSH-Enrichment Analysis (C : Diseases)

```
negLogPval <- -log10(out.vecLR[['pattern_2_2']]$Enrich$MeSH_C$Pvalue + 1E-10)
term <- out.vecLR[['pattern_2_2']]$Enrich$MeSH_C$Term
target <- seq_len(min(100, length(negLogPval)))
negLogPval <- negLogPval[target]
term <- term[target]
p <- plot_ly(x=seq_along(negLogPval), y=~negLogPval,
type="bar", color=~negLogPval, text=term,
colors=c("#4b61ba", "gray", "#a87963", "red"))
layout(p, title="MeSH-Enrichment Analysis (C)",
   xaxis=list(title="Term"),
   yaxis=list(title="-Log(P-value)"),
   legend=list(name=""))
```

```
## Warning: textfont.color doesn't (yet) support data arrays

## Warning: textfont.color doesn't (yet) support data arrays
```

## MeSH-Enrichment Analysis (D : Drugs)

```
negLogPval <- -log10(out.vecLR[['pattern_2_2']]$Enrich$MeSH_D$Pvalue + 1E-10)
term <- out.vecLR[['pattern_2_2']]$Enrich$MeSH_D$Term
target <- seq_len(min(100, length(negLogPval)))
negLogPval <- negLogPval[target]
term <- term[target]
p <- plot_ly(x=seq_along(negLogPval), y=~negLogPval,
type="bar", color=~negLogPval, text=term,
colors=c("#4b61ba", "gray", "#a87963", "red"))
layout(p, title="MeSH-Enrichment Analysis (D)",
   xaxis=list(title="Term"),
   yaxis=list(title="-Log(P-value)"),
   legend=list(name=""))
```

```
## Warning: textfont.color doesn't (yet) support data arrays

## Warning: textfont.color doesn't (yet) support data arrays
```

## MeSH-Enrichment Analysis (E : Analytical, Diagnostic and Therapeutic Techniques and Equipment)

```
negLogPval <- -log10(out.vecLR[['pattern_2_2']]$Enrich$MeSH_E$Pvalue + 1E-10)
term <- out.vecLR[['pattern_2_2']]$Enrich$MeSH_E$Term
target <- seq_len(min(100, length(negLogPval)))
negLogPval <- negLogPval[target]
term <- term[target]
p <- plot_ly(x=seq_along(negLogPval), y=~negLogPval,
type="bar", color=~negLogPval, text=term,
colors=c("#4b61ba", "gray", "#a87963", "red"))
layout(p, title="MeSH-Enrichment Analysis (E)",
   xaxis=list(title="Term"),
   yaxis=list(title="-Log(P-value)"),
   legend=list(name=""))
```

```
## Warning: textfont.color doesn't (yet) support data arrays

## Warning: textfont.color doesn't (yet) support data arrays
```

## MeSH-Enrichment Analysis (F : Psychiatry and Psychology)

```
negLogPval <- -log10(out.vecLR[['pattern_2_2']]$Enrich$MeSH_F$Pvalue + 1E-10)
term <- out.vecLR[['pattern_2_2']]$Enrich$MeSH_F$Term
target <- seq_len(min(100, length(negLogPval)))
negLogPval <- negLogPval[target]
term <- term[target]
p <- plot_ly(x=seq_along(negLogPval), y=~negLogPval,
type="bar", color=~negLogPval, text=term,
colors=c("#4b61ba", "gray", "#a87963", "red"))
layout(p, title="MeSH-Enrichment Analysis (F)",
   xaxis=list(title="Term"),
   yaxis=list(title="-Log(P-value)"),
   legend=list(name=""))
```

```
## Warning: textfont.color doesn't (yet) support data arrays

## Warning: textfont.color doesn't (yet) support data arrays
```

## MeSH-Enrichment Analysis (G : Phenomena and Processes)

```
negLogPval <- -log10(out.vecLR[['pattern_2_2']]$Enrich$MeSH_G$Pvalue + 1E-10)
term <- out.vecLR[['pattern_2_2']]$Enrich$MeSH_G$Term
target <- seq_len(min(100, length(negLogPval)))
negLogPval <- negLogPval[target]
term <- term[target]
p <- plot_ly(x=seq_along(negLogPval), y=~negLogPval,
type="bar", color=~negLogPval, text=term,
colors=c("#4b61ba", "gray", "#a87963", "red"))
layout(p, title="MeSH-Enrichment Analysis (G)",
   xaxis=list(title="Term"),
   yaxis=list(title="-Log(P-value)"),
   legend=list(name=""))
```

```
## Warning: textfont.color doesn't (yet) support data arrays

## Warning: textfont.color doesn't (yet) support data arrays
```

## MeSH-Enrichment Analysis (H : Disciplines and Occupations)

```
negLogPval <- -log10(out.vecLR[['pattern_2_2']]$Enrich$MeSH_H$Pvalue + 1E-10)
term <- out.vecLR[['pattern_2_2']]$Enrich$MeSH_H$Term
target <- seq_len(min(100, length(negLogPval)))
negLogPval <- negLogPval[target]
term <- term[target]
p <- plot_ly(x=seq_along(negLogPval), y=~negLogPval,
type="bar", color=~negLogPval, text=term,
colors=c("#4b61ba", "gray", "#a87963", "red"))
layout(p, title="MeSH-Enrichment Analysis (H)",
   xaxis=list(title="Term"),
   yaxis=list(title="-Log(P-value)"),
   legend=list(name=""))
```

```
## Warning: textfont.color doesn't (yet) support data arrays

## Warning: textfont.color doesn't (yet) support data arrays
```

## MeSH-Enrichment Analysis (I : Anthropology, Education, Sociology and Social Phenomena)

```
negLogPval <- -log10(out.vecLR[['pattern_2_2']]$Enrich$MeSH_I$Pvalue + 1E-10)
term <- out.vecLR[['pattern_2_2']]$Enrich$MeSH_I$Term
target <- seq_len(min(100, length(negLogPval)))
negLogPval <- negLogPval[target]
term <- term[target]
p <- plot_ly(x=seq_along(negLogPval), y=~negLogPval,
type="bar", color=~negLogPval, text=term,
colors=c("#4b61ba", "gray", "#a87963", "red"))
layout(p, title="MeSH-Enrichment Analysis (I)",
   xaxis=list(title="Term"),
   yaxis=list(title="-Log(P-value)"),
   legend=list(name=""))
```

```
## Warning: textfont.color doesn't (yet) support data arrays

## Warning: textfont.color doesn't (yet) support data arrays
```

## MeSH-Enrichment Analysis (J : Technology and Food and Beverages)

```
negLogPval <- -log10(out.vecLR[['pattern_2_2']]$Enrich$MeSH_J$Pvalue + 1E-10)
term <- out.vecLR[['pattern_2_2']]$Enrich$MeSH_J$Term
target <- seq_len(min(100, length(negLogPval)))
negLogPval <- negLogPval[target]
term <- term[target]
p <- plot_ly(x=seq_along(negLogPval), y=~negLogPval,
type="bar", color=~negLogPval, text=term,
colors=c("#4b61ba", "gray", "#a87963", "red"))
layout(p, title="MeSH-Enrichment Analysis (J)",
   xaxis=list(title="Term"),
   yaxis=list(title="-Log(P-value)"),
   legend=list(name=""))
```

```
## Warning: textfont.color doesn't (yet) support data arrays

## Warning: textfont.color doesn't (yet) support data arrays
```

## MeSH-Enrichment Analysis (L : Information Science)

```
negLogPval <- -log10(out.vecLR[['pattern_2_2']]$Enrich$MeSH_L$Pvalue + 1E-10)
term <- out.vecLR[['pattern_2_2']]$Enrich$MeSH_L$Term
target <- seq_len(min(100, length(negLogPval)))
negLogPval <- negLogPval[target]
term <- term[target]
p <- plot_ly(x=seq_along(negLogPval), y=~negLogPval,
type="bar", color=~negLogPval, text=term,
colors=c("#4b61ba", "gray", "#a87963", "red"))
layout(p, title="MeSH-Enrichment Analysis (L)",
   xaxis=list(title="Term"),
   yaxis=list(title="-Log(P-value)"),
   legend=list(name=""))
```

```
## Warning: textfont.color doesn't (yet) support data arrays

## Warning: textfont.color doesn't (yet) support data arrays
```

## MeSH-Enrichment Analysis (M : Persons)

```
negLogPval <- -log10(out.vecLR[['pattern_2_2']]$Enrich$MeSH_M$Pvalue + 1E-10)
term <- out.vecLR[['pattern_2_2']]$Enrich$MeSH_M$Term
target <- seq_len(min(100, length(negLogPval)))
negLogPval <- negLogPval[target]
term <- term[target]
p <- plot_ly(x=seq_along(negLogPval), y=~negLogPval,
type="bar", color=~negLogPval, text=term,
colors=c("#4b61ba", "gray", "#a87963", "red"))
layout(p, title="MeSH-Enrichment Analysis (M)",
   xaxis=list(title="Term"),
   yaxis=list(title="-Log(P-value)"),
   legend=list(name=""))
```

```
## Warning: textfont.color doesn't (yet) support data arrays

## Warning: textfont.color doesn't (yet) support data arrays
```

## MeSH-Enrichment Analysis (N : Health Care)

```
negLogPval <- -log10(out.vecLR[['pattern_2_2']]$Enrich$MeSH_N$Pvalue + 1E-10)
term <- out.vecLR[['pattern_2_2']]$Enrich$MeSH_N$Term
target <- seq_len(min(100, length(negLogPval)))
negLogPval <- negLogPval[target]
term <- term[target]
p <- plot_ly(x=seq_along(negLogPval), y=~negLogPval,
type="bar", color=~negLogPval, text=term,
colors=c("#4b61ba", "gray", "#a87963", "red"))
layout(p, title="MeSH-Enrichment Analysis (N)",
   xaxis=list(title="Term"),
   yaxis=list(title="-Log(P-value)"),
   legend=list(name=""))
```

```
## Warning: textfont.color doesn't (yet) support data arrays

## Warning: textfont.color doesn't (yet) support data arrays
```

## Reactome Pathway-Enrichment Analysis

```
negLogPval <- -log10(out.vecLR[['pattern_2_2']]$Enrich$Reactome$Pvalue + 1E-10)
term <- out.vecLR[['pattern_2_2']]$Enrich$Reactome$Term
target <- seq_len(min(100, length(negLogPval)))
negLogPval <- negLogPval[target]
term <- term[target]
p <- plot_ly(x=seq_along(negLogPval), y=~negLogPval,
type="bar", color=~negLogPval, text=term,
colors=c("#4b61ba", "gray", "#a87963", "red"))
layout(p, title="Reactome-Enrichment Analysis",
   xaxis=list(title="Term"),
   yaxis=list(title="-Log(P-value)"),
   legend=list(name=""))
```

```
## Warning: textfont.color doesn't (yet) support data arrays

## Warning: textfont.color doesn't (yet) support data arrays
```

## DO-Enrichment Analysis

```
negLogPval <- -log10(out.vecLR[['pattern_2_2']]$Enrich$DO$Pvalue + 1E-10)
term <- out.vecLR[['pattern_2_2']]$Enrich$DO$Term
target <- seq_len(min(100, length(negLogPval)))
negLogPval <- negLogPval[target]
term <- term[target]
p <- plot_ly(x=seq_along(negLogPval), y=~negLogPval,
type="bar", color=~negLogPval, text=term,
colors=c("#4b61ba", "gray", "#a87963", "red"))
layout(p, title="DO-Enrichment Analysis",
   xaxis=list(title="Term"),
   yaxis=list(title="-Log(P-value)"),
   legend=list(name=""))
```

```
## Warning: textfont.color doesn't (yet) support data arrays

## Warning: textfont.color doesn't (yet) support data arrays
```

## NCG-Enrichment Analysis

```
negLogPval <- -log10(out.vecLR[['pattern_2_2']]$Enrich$NCG$Pvalue + 1E-10)
term <- out.vecLR[['pattern_2_2']]$Enrich$NCG$Term
target <- seq_len(min(100, length(negLogPval)))
negLogPval <- negLogPval[target]
term <- term[target]
p <- plot_ly(x=seq_along(negLogPval), y=~negLogPval,
type="bar", color=~negLogPval, text=term,
colors=c("#4b61ba", "gray", "#a87963", "red"))
layout(p, title="NCG-Enrichment Analysis",
   xaxis=list(title="Term"),
   yaxis=list(title="-Log(P-value)"),
   legend=list(name=""))
```

```
## Warning: textfont.color doesn't (yet) support data arrays

## Warning: textfont.color doesn't (yet) support data arrays
```

## DGN-Enrichment Analysis

```
negLogPval <- -log10(out.vecLR[['pattern_2_2']]$Enrich$DGN$Pvalue + 1E-10)
term <- out.vecLR[['pattern_2_2']]$Enrich$DGN$Term
target <- seq_len(min(100, length(negLogPval)))
negLogPval <- negLogPval[target]
term <- term[target]
p <- plot_ly(x=seq_along(negLogPval), y=~negLogPval,
type="bar", color=~negLogPval, text=term,
colors=c("#4b61ba", "gray", "#a87963", "red"))
layout(p, title="DGN-Enrichment Analysis",
   xaxis=list(title="Term"),
   yaxis=list(title="-Log(P-value)"),
   legend=list(name=""))
```

```
## Warning: textfont.color doesn't (yet) support data arrays

## Warning: textfont.color doesn't (yet) support data arrays
```
