# Supplementary material for: Sctensor detects many-to-many cell–cell interactions from single cell RNA-sequencing data
Source: BMC Bioinformatics. 2023 Nov 7;24:420. doi: 10.1186/s12859-023-05490-y (PMC10631077; doi:10.1186/s12859-023-05490-y)
Supplement: Supplementary file 15 — Additional file 15. HTML report of GermlineFemale. [file 12859_2023_5490_MOESM15_ESM.zip › output/report/Human_Germline_Female/pattern_2_4.html]

Details of (2,4,) -related L-R pairs


# Details of (2,4,) -related L-R pairs

# (2,4,:) Pattern

# (2,4,) -related L-R pairs (Top31 pairs)

```
library("scTensor")
load("reanalysis.RData")
col <- .setColor("many")[8]
scTensor:::.myvisNetwork(g, col)
```

| Rank | Ligand Gene | Receptor Gene | Ligand Expression (Log10(exp + 1)) | Receptor Expression (Log10(exp + 1)) | LR-pair factor value (Percentage) | P-value (Grubbs test) | Q-value (BH method) | L-R Evidence cf. rikenbit/lrbase-workflow | PubMed |
| --- | --- | --- | --- | --- | --- | --- | --- | --- | --- |
| 1 | DLK1 Description: DLK1delta like non-canonical Notch ligand 1 GO: 1-9 Reactome: 1-4 UniProtKB: 1-3 RefEx: DLK1 Expression Atlas: DLK1 Single Cell Expression Atlas: DLK1 scRNASeqDB: DLK1 PanglaoDB: DLK1 CMap: DLK1 MeSH: 1-92 93-184 185-276 277-368 369-460 461-552 553-644 645-730 | NOTCH2 Description: DLK1notch receptor 2 GO: 1-63 Reactome: 1-20 UniProtKB: 1-3 RefEx: DLK1 Expression Atlas: NOTCH2 Single Cell Expression Atlas: NOTCH2 scRNASeqDB: NOTCH2 PanglaoDB: NOTCH2 CMap: NOTCH2 MeSH: 1-93 94-186 187-279 280-372 373-465 466-558 559-651 652-744 745-837 838-930 931-1023 1024-1116 1117-1203 |  |  | 2.534 (17.326%) | 0 | 0 | DLRP | 1 |
| 2 | NPY Description: NPYneuropeptide Y GO: 1-24 Reactome: 1-12 UniProtKB: 1-2 STRING: 1 2 3 RefEx: NPY Expression Atlas: NPY Single Cell Expression Atlas: NPY scRNASeqDB: NPY PanglaoDB: NPY CMap: NPY MeSH: 1-100 101-200 201-300 301-400 401-500 501-600 601-700 701-800 801-900 901-1000 1001-1100 1101-1200 1201-1300 1301-1400 1401-1500 1501-1600 1601-1700 1701-1800 1801-1900 1901-2000 2001-2100 2101-2200 2201-2300 2301-2400 2401-2500 2501-2600 2601-2700 2701-2800 2801-2900 2901-3000 3001-3100 3101-3200 3201-3300 3301-3400 3401-3500 3501-3600 3601-3700 3701-3800 3801-3900 3901-3983 | NPY1R Description: NPYneuropeptide Y receptor Y1 GO: 1-9 Reactome: 1-7 UniProtKB: 1 STRING: 1 2 3 4 5 6 7 RefEx: NPY Expression Atlas: NPY1R Single Cell Expression Atlas: NPY1R scRNASeqDB: NPY1R PanglaoDB: NPY1R CMap: NPY1R MeSH: 1-98 99-196 197-294 295-392 393-489 |  |  | 1.322 (9.038%) | 0.08 | 0.194 | DLRP | 1 |
| 3 | CTF1 Description: CTF1cardiotrophin 1 GO: 1-13 Reactome: 1-5 UniProtKB: 1 STRING: 1 2 RefEx: CTF1 Expression Atlas: CTF1 Single Cell Expression Atlas: CTF1 scRNASeqDB: CTF1 PanglaoDB: CTF1 CMap: CTF1 MeSH: 1-100 101-200 201-300 301-398 | IL6ST Description: CTF1interleukin 6 cytokine family signal transducer GO: 1-50 Reactome: 1-15 UniProtKB: 1-3 RefEx: CTF1 Expression Atlas: IL6ST Single Cell Expression Atlas: IL6ST scRNASeqDB: IL6ST PanglaoDB: IL6ST CMap: IL6ST MeSH: 1-100 101-200 201-300 301-400 401-500 501-600 601-700 701-800 801-900 901-1000 1001-1100 1101-1200 1201-1300 1301-1400 1401-1500 1501-1600 1601-1692 |  |  | 0.597 (4.081%) | 0.161 | 0.235 | DLRP | 1 |
| 4 | NCAM1 Description: NCAM1neural cell adhesion molecule 1 GO: 1-17 Reactome: 1-17 UniProtKB: 1-2 RefEx: NCAM1 Expression Atlas: NCAM1 Single Cell Expression Atlas: NCAM1 scRNASeqDB: NCAM1 PanglaoDB: NCAM1 CMap: NCAM1 MeSH: 1-94 95-188 189-282 283-376 377-470 471-564 565-658 659-752 753-846 847-940 941-1034 1035-1128 1129-1222 1223-1313 | ROBO1 Description: NCAM1roundabout guidance receptor 1 GO: 1-35 Reactome: 1-12 UniProtKB: 1-2 RefEx: NCAM1 Expression Atlas: ROBO1 Single Cell Expression Atlas: ROBO1 scRNASeqDB: ROBO1 PanglaoDB: ROBO1 CMap: ROBO1 MeSH: 1-95 96-190 191-285 286-380 381-475 476-570 571-659 |  |  | 0.554 (3.787%) | 0.205 | 0.273 | DLRP | 1 |
| 5 | GAS6 Description: GAS6growth arrest specific 6 GO: 1-76 Reactome: 1-14 UniProtKB: 1 RefEx: GAS6 Expression Atlas: GAS6 Single Cell Expression Atlas: GAS6 scRNASeqDB: GAS6 PanglaoDB: GAS6 CMap: GAS6 MeSH: 1-98 99-196 197-294 295-392 393-490 491-588 589-686 687-778 | AXL Description: GAS6AXL receptor tyrosine kinase GO: 1-60 Reactome: 1-4 UniProtKB: 1-2 RefEx: GAS6 Expression Atlas: AXL Single Cell Expression Atlas: AXL scRNASeqDB: AXL PanglaoDB: AXL CMap: AXL MeSH: 1-93 94-186 187-279 280-372 373-465 466-558 559-651 652-744 745-837 838-930 931-1013 |  |  | 0.541 (3.696%) | 0.001 | 0.003 | DLRP | 1 |
| 6 | VEGFA Description: VEGFAvascular endothelial growth factor A GO: 1-62 63-123 Reactome: 1-28 UniProtKB: 1-9 RefEx: VEGFA Expression Atlas: VEGFA Single Cell Expression Atlas: VEGFA scRNASeqDB: VEGFA PanglaoDB: VEGFA CMap: VEGFA MeSH: 1-100 101-200 201-300 301-400 401-500 501-600 601-700 701-800 801-900 901-1000 1001-1100 1101-1200 1201-1300 1301-1400 1401-1500 1501-1600 1601-1700 1701-1800 1801-1900 1901-2000 2001-2100 2101-2200 2201-2300 2301-2400 2401-2500 2501-2600 2601-2700 2701-2800 2801-2900 2901-3000 3001-3100 3101-3200 3201-3300 3301-3400 3401-3500 3501-3600 3601-3700 3701-3800 3801-3900 3901-4000 4001-4100 4101-4200 4201-4300 4301-4400 4401-4500 4501-4600 4601-4700 4701-4800 4801-4900 4901-5000 5001-5100 5101-5200 5201-5300 5301-5400 5401-5500 5501-5556 | NRP1 Description: VEGFAneuropilin 1 GO: 1-58 59-116 Reactome: 1-15 UniProtKB: 1-6 RefEx: VEGFA Expression Atlas: NRP1 Single Cell Expression Atlas: NRP1 scRNASeqDB: NRP1 PanglaoDB: NRP1 CMap: NRP1 MeSH: 1-96 97-192 193-288 289-384 385-480 481-576 577-672 673-768 769-864 865-960 961-1056 1057-1147 |  |  | 0.514 (3.517%) | 0.129 | 0.221 | DLRP | 1 |
| 7 | SLIT2 Description: SLIT2slit guidance ligand 2 GO: 1-53 Reactome: 1-11 UniProtKB: 1 RefEx: SLIT2 Expression Atlas: SLIT2 Single Cell Expression Atlas: SLIT2 scRNASeqDB: SLIT2 PanglaoDB: SLIT2 CMap: SLIT2 MeSH: 1-89 90-178 179-267 268-356 357-445 446-534 535-623 624-712 713-801 | ROBO1 Description: SLIT2roundabout guidance receptor 1 GO: 1-35 Reactome: 1-12 UniProtKB: 1-2 RefEx: SLIT2 Expression Atlas: ROBO1 Single Cell Expression Atlas: ROBO1 scRNASeqDB: ROBO1 PanglaoDB: ROBO1 CMap: ROBO1 MeSH: 1-95 96-190 191-285 286-380 381-475 476-570 571-659 |  |  | 0.498 (3.402%) | 0.228 | 0.288 | DLRP | 1 |
| 8 | SEMA6D Description: SEMA6Dsemaphorin 6D GO: 1-15 Reactome: 1-5 UniProtKB: 1-2 RefEx: SEMA6D Expression Atlas: SEMA6D Single Cell Expression Atlas: SEMA6D scRNASeqDB: SEMA6D PanglaoDB: SEMA6D CMap: SEMA6D MeSH: 1-96 97-191 | KDR Description: SEMA6Dkinase insert domain receptor GO: 1-70 Reactome: 1-14 UniProtKB: 1-2 RefEx: SEMA6D Expression Atlas: KDR Single Cell Expression Atlas: KDR scRNASeqDB: KDR PanglaoDB: KDR CMap: KDR MeSH: 1-97 98-194 195-291 292-388 389-485 486-582 583-679 680-776 777-873 874-970 971-1067 1068-1164 1165-1261 1262-1358 1359-1455 1456-1552 1553-1649 1650-1746 1747-1843 1844-1940 1941-2037 2038-2113 |  |  | 0.464 (3.169%) | 0.02 | 0.069 | DLRP | 1 |
| 9 | VEGFA Description: VEGFAvascular endothelial growth factor A GO: 1-62 63-123 Reactome: 1-28 UniProtKB: 1-9 RefEx: VEGFA Expression Atlas: VEGFA Single Cell Expression Atlas: VEGFA scRNASeqDB: VEGFA PanglaoDB: VEGFA CMap: VEGFA MeSH: 1-100 101-200 201-300 301-400 401-500 501-600 601-700 701-800 801-900 901-1000 1001-1100 1101-1200 1201-1300 1301-1400 1401-1500 1501-1600 1601-1700 1701-1800 1801-1900 1901-2000 2001-2100 2101-2200 2201-2300 2301-2400 2401-2500 2501-2600 2601-2700 2701-2800 2801-2900 2901-3000 3001-3100 3101-3200 3201-3300 3301-3400 3401-3500 3501-3600 3601-3700 3701-3800 3801-3900 3901-4000 4001-4100 4101-4200 4201-4300 4301-4400 4401-4500 4501-4600 4601-4700 4701-4800 4801-4900 4901-5000 5001-5100 5101-5200 5201-5300 5301-5400 5401-5500 5501-5556 | KDR Description: VEGFAkinase insert domain receptor GO: 1-70 Reactome: 1-14 UniProtKB: 1-2 RefEx: VEGFA Expression Atlas: KDR Single Cell Expression Atlas: KDR scRNASeqDB: KDR PanglaoDB: KDR CMap: KDR MeSH: 1-97 98-194 195-291 292-388 389-485 486-582 583-679 680-776 777-873 874-970 971-1067 1068-1164 1165-1261 1262-1358 1359-1455 1456-1552 1553-1649 1650-1746 1747-1843 1844-1940 1941-2037 2038-2113 |  |  | 0.443 (3.028%) | 0.144 | 0.234 | DLRP | 1 |
| 10 | WNT5A Description: WNT5AWnt family member 5A GO: 1-83 84-165 Reactome: 1-23 UniProtKB: 1-3 RefEx: WNT5A Expression Atlas: WNT5A Single Cell Expression Atlas: WNT5A scRNASeqDB: WNT5A PanglaoDB: WNT5A CMap: WNT5A MeSH: 1-100 101-200 201-300 301-400 401-500 501-600 601-700 701-800 801-900 901-1000 1001-1100 1101-1200 1201-1300 1301-1399 | FZD2 Description: WNT5Afrizzled class receptor 2 GO: 1-27 Reactome: 1-12 UniProtKB: 1-2 STRING: 1 RefEx: WNT5A Expression Atlas: FZD2 Single Cell Expression Atlas: FZD2 scRNASeqDB: FZD2 PanglaoDB: FZD2 CMap: FZD2 MeSH: 1-93 94-186 187-279 280-372 373-461 |  |  | 0.442 (3.02%) | 0.001 | 0.007 | DLRP | 1 |
| 11 | PDGFC Description: PDGFCplatelet derived growth factor C GO: 1-32 Reactome: 1-3 UniProtKB: 1 RefEx: PDGFC Expression Atlas: PDGFC Single Cell Expression Atlas: PDGFC scRNASeqDB: PDGFC PanglaoDB: PDGFC CMap: PDGFC MeSH: 1-88 89-176 177-264 265-352 353-440 441-528 529-614 | PDGFRA Description: PDGFCplatelet derived growth factor receptor alpha GO: 1-74 Reactome: 1-24 UniProtKB: 1 RefEx: PDGFC Expression Atlas: PDGFRA Single Cell Expression Atlas: PDGFRA scRNASeqDB: PDGFRA PanglaoDB: PDGFRA CMap: PDGFRA MeSH: 1-96 97-192 193-288 289-384 385-480 481-576 577-672 673-768 769-864 865-960 961-1056 1057-1152 1153-1248 1249-1333 |  |  | 0.44 (3.006%) | 1 | 1 | DLRP | 1 |
| 12 | WNT5A Description: WNT5AWnt family member 5A GO: 1-83 84-165 Reactome: 1-23 UniProtKB: 1-3 RefEx: WNT5A Expression Atlas: WNT5A Single Cell Expression Atlas: WNT5A scRNASeqDB: WNT5A PanglaoDB: WNT5A CMap: WNT5A MeSH: 1-100 101-200 201-300 301-400 401-500 501-600 601-700 701-800 801-900 901-1000 1001-1100 1101-1200 1201-1300 1301-1399 | FZD7 Description: WNT5Afrizzled class receptor 7 GO: 1-33 Reactome: 1-13 UniProtKB: 1 STRING: 1 RefEx: WNT5A Expression Atlas: FZD7 Single Cell Expression Atlas: FZD7 scRNASeqDB: FZD7 PanglaoDB: FZD7 CMap: FZD7 MeSH: 1-96 97-192 193-288 289-384 385-480 |  |  | 0.364 (2.488%) | 0.085 | 0.194 | DLRP | 1 |
| 13 | ADM Description: ADMadrenomedullin GO: 1-47 Reactome: 1-13 UniProtKB: 1 RefEx: ADM Expression Atlas: ADM Single Cell Expression Atlas: ADM scRNASeqDB: ADM PanglaoDB: ADM CMap: ADM MeSH: 1-99 100-198 199-297 298-396 397-495 496-594 595-693 694-792 793-891 892-990 991-1089 1090-1188 1189-1287 1288-1386 1387-1485 1486-1584 1585-1682 | RAMP2 Description: ADMreceptor activity modifying protein 2 GO: 1-45 Reactome: 1-13 UniProtKB: 1-2 RefEx: ADM Expression Atlas: RAMP2 Single Cell Expression Atlas: RAMP2 scRNASeqDB: RAMP2 PanglaoDB: RAMP2 CMap: RAMP2 MeSH: 1-89 90-178 179-267 268-355 |  |  | 0.355 (2.429%) | 0.329 | 0.374 | DLRP | 1 |
| 14 | CRLF1 Description: CRLF1cytokine receptor like factor 1 GO: 1-17 Reactome: 1-7 UniProtKB: 1 RefEx: CRLF1 Expression Atlas: CRLF1 Single Cell Expression Atlas: CRLF1 scRNASeqDB: CRLF1 PanglaoDB: CRLF1 CMap: CRLF1 MeSH: 1-74 75-148 149-222 | IL6ST Description: CRLF1interleukin 6 cytokine family signal transducer GO: 1-50 Reactome: 1-15 UniProtKB: 1-3 RefEx: CRLF1 Expression Atlas: IL6ST Single Cell Expression Atlas: IL6ST scRNASeqDB: IL6ST PanglaoDB: IL6ST CMap: IL6ST MeSH: 1-100 101-200 201-300 301-400 401-500 501-600 601-700 701-800 801-900 901-1000 1001-1100 1101-1200 1201-1300 1301-1400 1401-1500 1501-1600 1601-1692 |  |  | 0.314 (2.149%) | 0.446 | 0.477 | DLRP | 1 |
| 15 | DLK1 Description: DLK1delta like non-canonical Notch ligand 1 GO: 1-9 Reactome: 1-4 UniProtKB: 1-3 RefEx: DLK1 Expression Atlas: DLK1 Single Cell Expression Atlas: DLK1 scRNASeqDB: DLK1 PanglaoDB: DLK1 CMap: DLK1 MeSH: 1-92 93-184 185-276 277-368 369-460 461-552 553-644 645-730 | NOTCH3 Description: DLK1notch receptor 3 GO: 1-19 Reactome: 1-19 UniProtKB: 1 STRING: 1 2 3 4 RefEx: DLK1 Expression Atlas: NOTCH3 Single Cell Expression Atlas: NOTCH3 scRNASeqDB: NOTCH3 PanglaoDB: NOTCH3 CMap: NOTCH3 MeSH: 1-99 100-198 199-297 298-396 397-495 496-594 595-693 694-792 793-891 892-990 991-1089 1090-1188 1189-1275 |  |  | 0.312 (2.135%) | 0.126 | 0.221 | DLRP | 1 |
| 16 | COL18A1 Description: COL18A1collagen type XVIII alpha 1 chain GO: 1-20 Reactome: 1-10 UniProtKB: 1-3 RefEx: COL18A1 Expression Atlas: COL18A1 Single Cell Expression Atlas: COL18A1 scRNASeqDB: COL18A1 PanglaoDB: COL18A1 CMap: COL18A1 MeSH: 1-96 97-192 193-288 289-384 385-480 481-576 577-672 673-768 769-864 865-960 961-1054 | GPC1 Description: COL18A1glypican 1 GO: 1-26 Reactome: 1-28 UniProtKB: 1-2 STRING: 1 2 3 4 5 6 RefEx: COL18A1 Expression Atlas: GPC1 Single Cell Expression Atlas: GPC1 scRNASeqDB: GPC1 PanglaoDB: GPC1 CMap: GPC1 MeSH: 1-95 96-190 191-285 286-380 381-472 |  |  | 0.291 (1.991%) | 0.197 | 0.267 | DLRP | 1 |
| 17 | COL18A1 Description: COL18A1collagen type XVIII alpha 1 chain GO: 1-20 Reactome: 1-10 UniProtKB: 1-3 RefEx: COL18A1 Expression Atlas: COL18A1 Single Cell Expression Atlas: COL18A1 scRNASeqDB: COL18A1 PanglaoDB: COL18A1 CMap: COL18A1 MeSH: 1-96 97-192 193-288 289-384 385-480 481-576 577-672 673-768 769-864 865-960 961-1054 | ITGA5 Description: COL18A1integrin subunit alpha 5 GO: 1-28 Reactome: 1-16 UniProtKB: 1-2 RefEx: COL18A1 Expression Atlas: ITGA5 Single Cell Expression Atlas: ITGA5 scRNASeqDB: ITGA5 PanglaoDB: ITGA5 CMap: ITGA5 MeSH: 1-95 96-190 191-285 286-380 381-475 476-570 571-665 666-760 761-855 856-950 951-1045 1046-1140 1141-1235 1236-1330 1331-1414 |  |  | 0.26 (1.775%) | 0.125 | 0.221 | DLRP | 1 |
| 18 | FN1 Description: FN1fibronectin 1 GO: 1-55 Reactome: 1-49 UniProtKB: 1-6 RefEx: FN1 Expression Atlas: FN1 Single Cell Expression Atlas: FN1 scRNASeqDB: FN1 PanglaoDB: FN1 CMap: FN1 MeSH: 1-97 98-194 195-291 292-388 389-485 486-582 583-679 680-776 777-873 874-970 971-1067 1068-1164 1165-1261 1262-1358 1359-1455 1456-1552 1553-1649 1650-1746 1747-1843 1844-1940 1941-2033 | ITGA5 Description: FN1integrin subunit alpha 5 GO: 1-28 Reactome: 1-16 UniProtKB: 1-2 RefEx: FN1 Expression Atlas: ITGA5 Single Cell Expression Atlas: ITGA5 scRNASeqDB: ITGA5 PanglaoDB: ITGA5 CMap: ITGA5 MeSH: 1-95 96-190 191-285 286-380 381-475 476-570 571-665 666-760 761-855 856-950 951-1045 1046-1140 1141-1235 1236-1330 1331-1414 |  |  | 0.241 (1.651%) | 0.31 | 0.359 | DLRP | 1 |
| 19 | SLIT2 Description: SLIT2slit guidance ligand 2 GO: 1-53 Reactome: 1-11 UniProtKB: 1 RefEx: SLIT2 Expression Atlas: SLIT2 Single Cell Expression Atlas: SLIT2 scRNASeqDB: SLIT2 PanglaoDB: SLIT2 CMap: SLIT2 MeSH: 1-89 90-178 179-267 268-356 357-445 446-534 535-623 624-712 713-801 | GPC1 Description: SLIT2glypican 1 GO: 1-26 Reactome: 1-28 UniProtKB: 1-2 STRING: 1 2 3 4 5 6 RefEx: SLIT2 Expression Atlas: GPC1 Single Cell Expression Atlas: GPC1 scRNASeqDB: GPC1 PanglaoDB: GPC1 CMap: GPC1 MeSH: 1-95 96-190 191-285 286-380 381-472 |  |  | 0.241 (1.65%) | 0.137 | 0.231 | DLRP | 1 |
| 20 | SEMA3C Description: SEMA3Csemaphorin 3C GO: 1-24 UniProtKB: 1 RefEx: SEMA3C Expression Atlas: SEMA3C Single Cell Expression Atlas: SEMA3C scRNASeqDB: SEMA3C PanglaoDB: SEMA3C CMap: SEMA3C MeSH: 1-93 94-186 187-279 280-371 | NRP2 Description: SEMA3Cneuropilin 2 GO: 1-41 Reactome: 1-9 UniProtKB: 1-6 RefEx: SEMA3C Expression Atlas: NRP2 Single Cell Expression Atlas: NRP2 scRNASeqDB: NRP2 PanglaoDB: NRP2 CMap: NRP2 MeSH: 1-93 94-186 187-279 280-372 373-465 466-558 559-646 |  |  | 0.241 (1.649%) | 0.163 | 0.235 | DLRP | 1 |
| 21 | SLIT2 Description: SLIT2slit guidance ligand 2 GO: 1-53 Reactome: 1-11 UniProtKB: 1 RefEx: SLIT2 Expression Atlas: SLIT2 Single Cell Expression Atlas: SLIT2 scRNASeqDB: SLIT2 PanglaoDB: SLIT2 CMap: SLIT2 MeSH: 1-89 90-178 179-267 268-356 357-445 446-534 535-623 624-712 713-801 | ROBO2 Description: SLIT2roundabout guidance receptor 2 GO: 1-30 Reactome: 1-8 UniProtKB: 1 RefEx: SLIT2 Expression Atlas: ROBO2 Single Cell Expression Atlas: ROBO2 scRNASeqDB: ROBO2 PanglaoDB: ROBO2 CMap: ROBO2 MeSH: 1-91 92-182 183-273 274-363 |  |  | 0.233 (1.592%) | 0 | 0 | DLRP | 1 |
| 22 | PDGFC Description: PDGFCplatelet derived growth factor C GO: 1-32 Reactome: 1-3 UniProtKB: 1 RefEx: PDGFC Expression Atlas: PDGFC Single Cell Expression Atlas: PDGFC scRNASeqDB: PDGFC PanglaoDB: PDGFC CMap: PDGFC MeSH: 1-88 89-176 177-264 265-352 353-440 441-528 529-614 | KDR Description: PDGFCkinase insert domain receptor GO: 1-70 Reactome: 1-14 UniProtKB: 1-2 RefEx: PDGFC Expression Atlas: KDR Single Cell Expression Atlas: KDR scRNASeqDB: KDR PanglaoDB: KDR CMap: KDR MeSH: 1-97 98-194 195-291 292-388 389-485 486-582 583-679 680-776 777-873 874-970 971-1067 1068-1164 1165-1261 1262-1358 1359-1455 1456-1552 1553-1649 1650-1746 1747-1843 1844-1940 1941-2037 2038-2113 |  |  | 0.219 (1.497%) | 0.193 | 0.266 | DLRP | 1 |
| 23 | CTF1 Description: CTF1cardiotrophin 1 GO: 1-13 Reactome: 1-5 UniProtKB: 1 STRING: 1 2 RefEx: CTF1 Expression Atlas: CTF1 Single Cell Expression Atlas: CTF1 scRNASeqDB: CTF1 PanglaoDB: CTF1 CMap: CTF1 MeSH: 1-100 101-200 201-300 301-398 | LIFR Description: CTF1LIF receptor subunit alpha GO: 1-20 Reactome: 1-10 UniProtKB: 1-2 RefEx: CTF1 Expression Atlas: LIFR Single Cell Expression Atlas: LIFR scRNASeqDB: LIFR PanglaoDB: LIFR CMap: LIFR MeSH: 1-99 100-198 199-297 298-396 397-495 496-594 595-693 694-792 793-891 892-990 |  |  | 0.218 (1.493%) | 0 | 0 | DLRP | 1 |
| 24 | RSPO3 Description: RSPO3R-spondin 3 GO: 1-12 Reactome: 1-9 UniProtKB: 1 RefEx: RSPO3 Expression Atlas: RSPO3 Single Cell Expression Atlas: RSPO3 scRNASeqDB: RSPO3 PanglaoDB: RSPO3 CMap: RSPO3 MeSH: 1-100 101-200 201-300 301-398 | LGR4 Description: RSPO3leucine rich repeat containing G protein-coupled receptor 4 GO: 1-31 Reactome: 1-4 UniProtKB: 1-2 RefEx: RSPO3 Expression Atlas: LGR4 Single Cell Expression Atlas: LGR4 scRNASeqDB: LGR4 PanglaoDB: LGR4 CMap: LGR4 MeSH: 1-97 98-194 195-291 292-387 |  |  | 0.195 (1.333%) | 0.157 | 0.235 | DLRP | 1 |
| 25 | NPNT Description: NPNTnephronectin GO: 1-24 UniProtKB: 1 RefEx: NPNT Expression Atlas: NPNT Single Cell Expression Atlas: NPNT scRNASeqDB: NPNT PanglaoDB: NPNT CMap: NPNT MeSH: 1-88 89-175 | ITGA8 Description: NPNTintegrin subunit alpha 8 GO: 1-17 Reactome: 1-5 UniProtKB: 1-2 STRING: 1 RefEx: NPNT Expression Atlas: ITGA8 Single Cell Expression Atlas: ITGA8 scRNASeqDB: ITGA8 PanglaoDB: ITGA8 CMap: ITGA8 MeSH: 1-82 83-164 165-246 247-328 |  |  | 0.189 (1.289%) | 0.793 | 0.82 | DLRP | 1 |
| 26 | EFNB1 Description: EFNB1ephrin B1 GO: 1-17 Reactome: 1-7 UniProtKB: 1 STRING: 1 RefEx: EFNB1 Expression Atlas: EFNB1 Single Cell Expression Atlas: EFNB1 scRNASeqDB: EFNB1 PanglaoDB: EFNB1 CMap: EFNB1 MeSH: 1-89 90-178 179-267 268-356 357-445 446-534 535-620 | EPHB6 Description: EFNB1EPH receptor B6 GO: 1-18 Reactome: 1-7 UniProtKB: 1-3 STRING: 1 2 3 4 5 6 7 RefEx: EFNB1 Expression Atlas: EPHB6 Single Cell Expression Atlas: EPHB6 scRNASeqDB: EPHB6 PanglaoDB: EPHB6 CMap: EPHB6 MeSH: 1-92 93-184 185-276 277-368 369-456 |  |  | 0.188 (1.286%) | 0.066 | 0.178 | DLRP | 1 |
| 27 | BMP2 Description: BMP2bone morphogenetic protein 2 GO: 1-57 58-113 Reactome: 1-11 UniProtKB: 1-2 STRING: 1 RefEx: BMP2 Expression Atlas: BMP2 Single Cell Expression Atlas: BMP2 scRNASeqDB: BMP2 PanglaoDB: BMP2 CMap: BMP2 MeSH: 1-98 99-196 197-294 295-392 393-490 491-588 589-686 687-784 785-882 883-980 981-1078 1079-1176 1177-1274 1275-1372 1373-1470 1471-1568 1569-1666 1667-1764 1765-1862 1863-1960 1961-2058 2059-2156 2157-2254 2255-2352 2353-2450 2451-2548 2549-2646 2647-2742 | BMPR2 Description: BMP2bone morphogenetic protein receptor type 2 GO: 1-91 Reactome: 1-3 UniProtKB: 1 RefEx: BMP2 Expression Atlas: BMPR2 Single Cell Expression Atlas: BMPR2 scRNASeqDB: BMPR2 PanglaoDB: BMPR2 CMap: BMPR2 MeSH: 1-100 101-200 201-300 301-400 401-500 501-600 601-700 701-800 801-900 901-1000 1001-1098 |  |  | 0.164 (1.123%) | 0.007 | 0.03 | DLRP | 1 |
| 28 | PYY Description: PYYpeptide YY GO: 1-10 Reactome: 1-7 UniProtKB: 1 RefEx: PYY Expression Atlas: PYY Single Cell Expression Atlas: PYY scRNASeqDB: PYY PanglaoDB: PYY CMap: PYY MeSH: 1-94 95-188 189-282 283-376 377-470 471-564 565-658 659-752 753-846 847-940 941-1034 1035-1128 1129-1222 1223-1316 1317-1410 1411-1503 | NPY1R Description: PYYneuropeptide Y receptor Y1 GO: 1-9 Reactome: 1-7 UniProtKB: 1 STRING: 1 2 3 4 5 6 7 RefEx: PYY Expression Atlas: NPY1R Single Cell Expression Atlas: NPY1R scRNASeqDB: NPY1R PanglaoDB: NPY1R CMap: NPY1R MeSH: 1-98 99-196 197-294 295-392 393-489 |  |  | 0.155 (1.06%) | 0.108 | 0.206 | DLRP | 1 |
| 29 | NCAM1 Description: NCAM1neural cell adhesion molecule 1 GO: 1-17 Reactome: 1-17 UniProtKB: 1-2 RefEx: NCAM1 Expression Atlas: NCAM1 Single Cell Expression Atlas: NCAM1 scRNASeqDB: NCAM1 PanglaoDB: NCAM1 CMap: NCAM1 MeSH: 1-94 95-188 189-282 283-376 377-470 471-564 565-658 659-752 753-846 847-940 941-1034 1035-1128 1129-1222 1223-1313 | ROBO3 Description: NCAM1roundabout guidance receptor 3 GO: 1-9 Reactome: 1-7 UniProtKB: 1-2 RefEx: NCAM1 Expression Atlas: ROBO3 Single Cell Expression Atlas: ROBO3 scRNASeqDB: ROBO3 PanglaoDB: ROBO3 CMap: ROBO3 MeSH: 1-89 90-178 179-265 |  |  | 0.143 (0.976%) | 0 | 0 | DLRP | 1 |
| 30 | NPFF Description: NPFFneuropeptide FF-amide peptide precursor GO: 1-12 Reactome: 1-8 UniProtKB: 1 STRING: 1 2 RefEx: NPFF Expression Atlas: NPFF Single Cell Expression Atlas: NPFF scRNASeqDB: NPFF PanglaoDB: NPFF CMap: NPFF MeSH: 1-100 101-200 | NPFFR2 Description: NPFFneuropeptide FF receptor 2 GO: 1-13 Reactome: 1-8 UniProtKB: 1-2 RefEx: NPFF Expression Atlas: NPFFR2 Single Cell Expression Atlas: NPFFR2 scRNASeqDB: NPFFR2 PanglaoDB: NPFFR2 CMap: NPFFR2 MeSH: 1-71 72-142 143-212 |  |  | 0.137 (0.939%) | 0.312 | 0.359 | DLRP | 1 |
| 31 | EFNA5 Description: EFNA5ephrin A5 GO: 1-23 Reactome: 1-6 UniProtKB: 1-2 RefEx: EFNA5 Expression Atlas: EFNA5 Single Cell Expression Atlas: EFNA5 scRNASeqDB: EFNA5 PanglaoDB: EFNA5 CMap: EFNA5 MeSH: 1-100 101-200 201-300 301-398 | EPHB2 Description: EFNA5EPH receptor B2 GO: 1-72 Reactome: 1-8 UniProtKB: 1-4 RefEx: EFNA5 Expression Atlas: EPHB2 Single Cell Expression Atlas: EPHB2 scRNASeqDB: EPHB2 PanglaoDB: EPHB2 CMap: EPHB2 MeSH: 1-99 100-198 199-297 298-396 397-495 496-594 595-693 694-792 793-885 |  |  | 0.126 (0.864%) | 0.067 | 0.178 | DLRP | 1 |

# (2,4,) -related Enrichment Analysis

```
load("reanalysis.RData")
library("plotly")
```

## MeSH-Enrichment Analysis (A : Anatomy)

```
negLogPval <- -log10(out.vecLR[['pattern_2_4']]$Enrich$MeSH_A$Pvalue + 1E-10)
term <- out.vecLR[['pattern_2_4']]$Enrich$MeSH_A$Term
target <- seq_len(min(100, length(negLogPval)))
negLogPval <- negLogPval[target]
term <- term[target]
p <- plot_ly(x=seq_along(negLogPval), y=~negLogPval,
type="bar", color=~negLogPval, text=term,
colors=c("#4b61ba", "gray", "#a87963", "red"))
layout(p, title="MeSH-Enrichment Analysis (A)",
   xaxis=list(title="Term"),
   yaxis=list(title="-Log(P-value)"),
   legend=list(name=""))
```

```
## Warning: textfont.color doesn't (yet) support data arrays

## Warning: textfont.color doesn't (yet) support data arrays
```

## MeSH-Enrichment Analysis (B : Organisms)

```
negLogPval <- -log10(out.vecLR[['pattern_2_4']]$Enrich$MeSH_B$Pvalue + 1E-10)
term <- out.vecLR[['pattern_2_4']]$Enrich$MeSH_B$Term
target <- seq_len(min(100, length(negLogPval)))
negLogPval <- negLogPval[target]
term <- term[target]
p <- plot_ly(x=seq_along(negLogPval), y=~negLogPval,
type="bar", color=~negLogPval, text=term,
colors=c("#4b61ba", "gray", "#a87963", "red"))
layout(p, title="MeSH-Enrichment Analysis (B)",
   xaxis=list(title="Term"),
   yaxis=list(title="-Log(P-value)"),
   legend=list(name=""))
```

```
## Warning: textfont.color doesn't (yet) support data arrays

## Warning: textfont.color doesn't (yet) support data arrays
```

## MeSH-Enrichment Analysis (C : Diseases)

```
negLogPval <- -log10(out.vecLR[['pattern_2_4']]$Enrich$MeSH_C$Pvalue + 1E-10)
term <- out.vecLR[['pattern_2_4']]$Enrich$MeSH_C$Term
target <- seq_len(min(100, length(negLogPval)))
negLogPval <- negLogPval[target]
term <- term[target]
p <- plot_ly(x=seq_along(negLogPval), y=~negLogPval,
type="bar", color=~negLogPval, text=term,
colors=c("#4b61ba", "gray", "#a87963", "red"))
layout(p, title="MeSH-Enrichment Analysis (C)",
   xaxis=list(title="Term"),
   yaxis=list(title="-Log(P-value)"),
   legend=list(name=""))
```

```
## Warning: textfont.color doesn't (yet) support data arrays

## Warning: textfont.color doesn't (yet) support data arrays
```

## MeSH-Enrichment Analysis (D : Drugs)

```
negLogPval <- -log10(out.vecLR[['pattern_2_4']]$Enrich$MeSH_D$Pvalue + 1E-10)
term <- out.vecLR[['pattern_2_4']]$Enrich$MeSH_D$Term
target <- seq_len(min(100, length(negLogPval)))
negLogPval <- negLogPval[target]
term <- term[target]
p <- plot_ly(x=seq_along(negLogPval), y=~negLogPval,
type="bar", color=~negLogPval, text=term,
colors=c("#4b61ba", "gray", "#a87963", "red"))
layout(p, title="MeSH-Enrichment Analysis (D)",
   xaxis=list(title="Term"),
   yaxis=list(title="-Log(P-value)"),
   legend=list(name=""))
```

```
## Warning: textfont.color doesn't (yet) support data arrays

## Warning: textfont.color doesn't (yet) support data arrays
```

## MeSH-Enrichment Analysis (E : Analytical, Diagnostic and Therapeutic Techniques and Equipment)

```
negLogPval <- -log10(out.vecLR[['pattern_2_4']]$Enrich$MeSH_E$Pvalue + 1E-10)
term <- out.vecLR[['pattern_2_4']]$Enrich$MeSH_E$Term
target <- seq_len(min(100, length(negLogPval)))
negLogPval <- negLogPval[target]
term <- term[target]
p <- plot_ly(x=seq_along(negLogPval), y=~negLogPval,
type="bar", color=~negLogPval, text=term,
colors=c("#4b61ba", "gray", "#a87963", "red"))
layout(p, title="MeSH-Enrichment Analysis (E)",
   xaxis=list(title="Term"),
   yaxis=list(title="-Log(P-value)"),
   legend=list(name=""))
```

```
## Warning: textfont.color doesn't (yet) support data arrays

## Warning: textfont.color doesn't (yet) support data arrays
```

## MeSH-Enrichment Analysis (F : Psychiatry and Psychology)

```
negLogPval <- -log10(out.vecLR[['pattern_2_4']]$Enrich$MeSH_F$Pvalue + 1E-10)
term <- out.vecLR[['pattern_2_4']]$Enrich$MeSH_F$Term
target <- seq_len(min(100, length(negLogPval)))
negLogPval <- negLogPval[target]
term <- term[target]
p <- plot_ly(x=seq_along(negLogPval), y=~negLogPval,
type="bar", color=~negLogPval, text=term,
colors=c("#4b61ba", "gray", "#a87963", "red"))
layout(p, title="MeSH-Enrichment Analysis (F)",
   xaxis=list(title="Term"),
   yaxis=list(title="-Log(P-value)"),
   legend=list(name=""))
```

```
## Warning: textfont.color doesn't (yet) support data arrays

## Warning: textfont.color doesn't (yet) support data arrays
```

## MeSH-Enrichment Analysis (G : Phenomena and Processes)

```
negLogPval <- -log10(out.vecLR[['pattern_2_4']]$Enrich$MeSH_G$Pvalue + 1E-10)
term <- out.vecLR[['pattern_2_4']]$Enrich$MeSH_G$Term
target <- seq_len(min(100, length(negLogPval)))
negLogPval <- negLogPval[target]
term <- term[target]
p <- plot_ly(x=seq_along(negLogPval), y=~negLogPval,
type="bar", color=~negLogPval, text=term,
colors=c("#4b61ba", "gray", "#a87963", "red"))
layout(p, title="MeSH-Enrichment Analysis (G)",
   xaxis=list(title="Term"),
   yaxis=list(title="-Log(P-value)"),
   legend=list(name=""))
```

```
## Warning: textfont.color doesn't (yet) support data arrays

## Warning: textfont.color doesn't (yet) support data arrays
```

## MeSH-Enrichment Analysis (H : Disciplines and Occupations)

```
negLogPval <- -log10(out.vecLR[['pattern_2_4']]$Enrich$MeSH_H$Pvalue + 1E-10)
term <- out.vecLR[['pattern_2_4']]$Enrich$MeSH_H$Term
target <- seq_len(min(100, length(negLogPval)))
negLogPval <- negLogPval[target]
term <- term[target]
p <- plot_ly(x=seq_along(negLogPval), y=~negLogPval,
type="bar", color=~negLogPval, text=term,
colors=c("#4b61ba", "gray", "#a87963", "red"))
layout(p, title="MeSH-Enrichment Analysis (H)",
   xaxis=list(title="Term"),
   yaxis=list(title="-Log(P-value)"),
   legend=list(name=""))
```

```
## Warning: textfont.color doesn't (yet) support data arrays

## Warning: textfont.color doesn't (yet) support data arrays
```

## MeSH-Enrichment Analysis (I : Anthropology, Education, Sociology and Social Phenomena)

```
negLogPval <- -log10(out.vecLR[['pattern_2_4']]$Enrich$MeSH_I$Pvalue + 1E-10)
term <- out.vecLR[['pattern_2_4']]$Enrich$MeSH_I$Term
target <- seq_len(min(100, length(negLogPval)))
negLogPval <- negLogPval[target]
term <- term[target]
p <- plot_ly(x=seq_along(negLogPval), y=~negLogPval,
type="bar", color=~negLogPval, text=term,
colors=c("#4b61ba", "gray", "#a87963", "red"))
layout(p, title="MeSH-Enrichment Analysis (I)",
   xaxis=list(title="Term"),
   yaxis=list(title="-Log(P-value)"),
   legend=list(name=""))
```

## MeSH-Enrichment Analysis (J : Technology and Food and Beverages)

```
negLogPval <- -log10(out.vecLR[['pattern_2_4']]$Enrich$MeSH_J$Pvalue + 1E-10)
term <- out.vecLR[['pattern_2_4']]$Enrich$MeSH_J$Term
target <- seq_len(min(100, length(negLogPval)))
negLogPval <- negLogPval[target]
term <- term[target]
p <- plot_ly(x=seq_along(negLogPval), y=~negLogPval,
type="bar", color=~negLogPval, text=term,
colors=c("#4b61ba", "gray", "#a87963", "red"))
layout(p, title="MeSH-Enrichment Analysis (J)",
   xaxis=list(title="Term"),
   yaxis=list(title="-Log(P-value)"),
   legend=list(name=""))
```

## MeSH-Enrichment Analysis (N : Health Care)

```
negLogPval <- -log10(out.vecLR[['pattern_2_4']]$Enrich$MeSH_N$Pvalue + 1E-10)
term <- out.vecLR[['pattern_2_4']]$Enrich$MeSH_N$Term
target <- seq_len(min(100, length(negLogPval)))
negLogPval <- negLogPval[target]
term <- term[target]
p <- plot_ly(x=seq_along(negLogPval), y=~negLogPval,
type="bar", color=~negLogPval, text=term,
colors=c("#4b61ba", "gray", "#a87963", "red"))
layout(p, title="MeSH-Enrichment Analysis (N)",
   xaxis=list(title="Term"),
   yaxis=list(title="-Log(P-value)"),
   legend=list(name=""))
```

```
## Warning: textfont.color doesn't (yet) support data arrays

## Warning: textfont.color doesn't (yet) support data arrays
```

## Reactome Pathway-Enrichment Analysis

```
negLogPval <- -log10(out.vecLR[['pattern_2_4']]$Enrich$Reactome$Pvalue + 1E-10)
term <- out.vecLR[['pattern_2_4']]$Enrich$Reactome$Term
target <- seq_len(min(100, length(negLogPval)))
negLogPval <- negLogPval[target]
term <- term[target]
p <- plot_ly(x=seq_along(negLogPval), y=~negLogPval,
type="bar", color=~negLogPval, text=term,
colors=c("#4b61ba", "gray", "#a87963", "red"))
layout(p, title="Reactome-Enrichment Analysis",
   xaxis=list(title="Term"),
   yaxis=list(title="-Log(P-value)"),
   legend=list(name=""))
```

```
## Warning: textfont.color doesn't (yet) support data arrays

## Warning: textfont.color doesn't (yet) support data arrays
```

## DO-Enrichment Analysis

```
negLogPval <- -log10(out.vecLR[['pattern_2_4']]$Enrich$DO$Pvalue + 1E-10)
term <- out.vecLR[['pattern_2_4']]$Enrich$DO$Term
target <- seq_len(min(100, length(negLogPval)))
negLogPval <- negLogPval[target]
term <- term[target]
p <- plot_ly(x=seq_along(negLogPval), y=~negLogPval,
type="bar", color=~negLogPval, text=term,
colors=c("#4b61ba", "gray", "#a87963", "red"))
layout(p, title="DO-Enrichment Analysis",
   xaxis=list(title="Term"),
   yaxis=list(title="-Log(P-value)"),
   legend=list(name=""))
```

```
## Warning: textfont.color doesn't (yet) support data arrays

## Warning: textfont.color doesn't (yet) support data arrays
```

## NCG-Enrichment Analysis

```
negLogPval <- -log10(out.vecLR[['pattern_2_4']]$Enrich$NCG$Pvalue + 1E-10)
term <- out.vecLR[['pattern_2_4']]$Enrich$NCG$Term
target <- seq_len(min(100, length(negLogPval)))
negLogPval <- negLogPval[target]
term <- term[target]
p <- plot_ly(x=seq_along(negLogPval), y=~negLogPval,
type="bar", color=~negLogPval, text=term,
colors=c("#4b61ba", "gray", "#a87963", "red"))
layout(p, title="NCG-Enrichment Analysis",
   xaxis=list(title="Term"),
   yaxis=list(title="-Log(P-value)"),
   legend=list(name=""))
```

```
## Warning: textfont.color doesn't (yet) support data arrays

## Warning: textfont.color doesn't (yet) support data arrays
```

## DGN-Enrichment Analysis

```
negLogPval <- -log10(out.vecLR[['pattern_2_4']]$Enrich$DGN$Pvalue + 1E-10)
term <- out.vecLR[['pattern_2_4']]$Enrich$DGN$Term
target <- seq_len(min(100, length(negLogPval)))
negLogPval <- negLogPval[target]
term <- term[target]
p <- plot_ly(x=seq_along(negLogPval), y=~negLogPval,
type="bar", color=~negLogPval, text=term,
colors=c("#4b61ba", "gray", "#a87963", "red"))
layout(p, title="DGN-Enrichment Analysis",
   xaxis=list(title="Term"),
   yaxis=list(title="-Log(P-value)"),
   legend=list(name=""))
```

```
## Warning: textfont.color doesn't (yet) support data arrays

## Warning: textfont.color doesn't (yet) support data arrays
```
