# Supplementary material for: Sctensor detects many-to-many cell–cell interactions from single cell RNA-sequencing data
Source: BMC Bioinformatics. 2023 Nov 7;24:420. doi: 10.1186/s12859-023-05490-y (PMC10631077; doi:10.1186/s12859-023-05490-y)
Supplement: Supplementary file 15 — Additional file 15. HTML report of GermlineFemale. [file 12859_2023_5490_MOESM15_ESM.zip › output/report/Human_Germline_Female/pattern_3_3.html]

Details of (3,3,) -related L-R pairs


# Details of (3,3,) -related L-R pairs

# (3,3,:) Pattern

# (3,3,) -related L-R pairs (Top11 pairs)

```
library("scTensor")
load("reanalysis.RData")
col <- .setColor("many")[9]
scTensor:::.myvisNetwork(g, col)
```

| Rank | Ligand Gene | Receptor Gene | Ligand Expression (Log10(exp + 1)) | Receptor Expression (Log10(exp + 1)) | LR-pair factor value (Percentage) | P-value (Grubbs test) | Q-value (BH method) | L-R Evidence cf. rikenbit/lrbase-workflow | PubMed |
| --- | --- | --- | --- | --- | --- | --- | --- | --- | --- |
| 1 | TFPI Description: TFPItissue factor pathway inhibitor GO: 1-16 Reactome: 1-3 UniProtKB: 1 RefEx: TFPI Expression Atlas: TFPI Single Cell Expression Atlas: TFPI scRNASeqDB: TFPI PanglaoDB: TFPI CMap: TFPI MeSH: 1-97 98-194 195-291 292-388 389-485 486-582 583-679 680-776 777-873 874-970 971-1067 1068-1164 1165-1261 1262-1358 1359-1455 1456-1538 | VLDLR Description: TFPIvery low density lipoprotein receptor GO: 1-29 Reactome: 1-9 UniProtKB: 1-2 RefEx: TFPI Expression Atlas: VLDLR Single Cell Expression Atlas: VLDLR scRNASeqDB: VLDLR PanglaoDB: VLDLR CMap: VLDLR MeSH: 1-88 89-176 177-264 265-352 353-440 441-528 529-616 617-702 |  |  | 3.014 (20.693%) | 0 | 0 | DLRP | 1 |
| 2 | EFNA1 Description: EFNA1ephrin A1 GO: 1-34 Reactome: 1-6 UniProtKB: 1 RefEx: EFNA1 Expression Atlas: EFNA1 Single Cell Expression Atlas: EFNA1 scRNASeqDB: EFNA1 PanglaoDB: EFNA1 CMap: EFNA1 MeSH: 1-94 95-188 189-282 283-376 377-466 | EPHA4 Description: EFNA1EPH receptor A4 GO: 1-77 Reactome: 1-6 UniProtKB: 1-2 RefEx: EFNA1 Expression Atlas: EPHA4 Single Cell Expression Atlas: EPHA4 scRNASeqDB: EPHA4 PanglaoDB: EPHA4 CMap: EPHA4 MeSH: 1-93 94-186 187-279 280-372 373-465 466-557 |  |  | 1.551 (10.646%) | 0.008 | 0.01 | DLRP | 1 |
| 3 | ADM Description: ADMadrenomedullin GO: 1-47 Reactome: 1-13 UniProtKB: 1 RefEx: ADM Expression Atlas: ADM Single Cell Expression Atlas: ADM scRNASeqDB: ADM PanglaoDB: ADM CMap: ADM MeSH: 1-99 100-198 199-297 298-396 397-495 496-594 595-693 694-792 793-891 892-990 991-1089 1090-1188 1189-1287 1288-1386 1387-1485 1486-1584 1585-1682 | RAMP2 Description: ADMreceptor activity modifying protein 2 GO: 1-45 Reactome: 1-13 UniProtKB: 1-2 RefEx: ADM Expression Atlas: RAMP2 Single Cell Expression Atlas: RAMP2 scRNASeqDB: RAMP2 PanglaoDB: RAMP2 CMap: RAMP2 MeSH: 1-89 90-178 179-267 268-355 |  |  | 1.018 (6.987%) | 0 | 0 | DLRP | 1 |
| 4 | FN1 Description: FN1fibronectin 1 GO: 1-55 Reactome: 1-49 UniProtKB: 1-6 RefEx: FN1 Expression Atlas: FN1 Single Cell Expression Atlas: FN1 scRNASeqDB: FN1 PanglaoDB: FN1 CMap: FN1 MeSH: 1-97 98-194 195-291 292-388 389-485 486-582 583-679 680-776 777-873 874-970 971-1067 1068-1164 1165-1261 1262-1358 1359-1455 1456-1552 1553-1649 1650-1746 1747-1843 1844-1940 1941-2033 | ITGA5 Description: FN1integrin subunit alpha 5 GO: 1-28 Reactome: 1-16 UniProtKB: 1-2 RefEx: FN1 Expression Atlas: ITGA5 Single Cell Expression Atlas: ITGA5 scRNASeqDB: ITGA5 PanglaoDB: ITGA5 CMap: ITGA5 MeSH: 1-95 96-190 191-285 286-380 381-475 476-570 571-665 666-760 761-855 856-950 951-1045 1046-1140 1141-1235 1236-1330 1331-1414 |  |  | 0.988 (6.78%) | 0.005 | 0.007 | DLRP | 1 |
| 5 | FN1 Description: FN1fibronectin 1 GO: 1-55 Reactome: 1-49 UniProtKB: 1-6 RefEx: FN1 Expression Atlas: FN1 Single Cell Expression Atlas: FN1 scRNASeqDB: FN1 PanglaoDB: FN1 CMap: FN1 MeSH: 1-97 98-194 195-291 292-388 389-485 486-582 583-679 680-776 777-873 874-970 971-1067 1068-1164 1165-1261 1262-1358 1359-1455 1456-1552 1553-1649 1650-1746 1747-1843 1844-1940 1941-2033 | ITGA2 Description: FN1integrin subunit alpha 2 GO: 1-65 Reactome: 1-18 UniProtKB: 1 RefEx: FN1 Expression Atlas: ITGA2 Single Cell Expression Atlas: ITGA2 scRNASeqDB: ITGA2 PanglaoDB: ITGA2 CMap: ITGA2 MeSH: 1-95 96-190 191-285 286-380 381-475 476-570 571-665 666-760 761-855 856-950 951-1045 1046-1140 1141-1235 1236-1330 1331-1413 |  |  | 0.969 (6.653%) | 0 | 0.001 | DLRP | 1 |
| 6 | DLK1 Description: DLK1delta like non-canonical Notch ligand 1 GO: 1-9 Reactome: 1-4 UniProtKB: 1-3 RefEx: DLK1 Expression Atlas: DLK1 Single Cell Expression Atlas: DLK1 scRNASeqDB: DLK1 PanglaoDB: DLK1 CMap: DLK1 MeSH: 1-92 93-184 185-276 277-368 369-460 461-552 553-644 645-730 | NOTCH2 Description: DLK1notch receptor 2 GO: 1-63 Reactome: 1-20 UniProtKB: 1-3 RefEx: DLK1 Expression Atlas: NOTCH2 Single Cell Expression Atlas: NOTCH2 scRNASeqDB: NOTCH2 PanglaoDB: NOTCH2 CMap: NOTCH2 MeSH: 1-93 94-186 187-279 280-372 373-465 466-558 559-651 652-744 745-837 838-930 931-1023 1024-1116 1117-1203 |  |  | 0.934 (6.409%) | 0 | 0 | DLRP | 1 |
| 7 | ADM Description: ADMadrenomedullin GO: 1-47 Reactome: 1-13 UniProtKB: 1 RefEx: ADM Expression Atlas: ADM Single Cell Expression Atlas: ADM scRNASeqDB: ADM PanglaoDB: ADM CMap: ADM MeSH: 1-99 100-198 199-297 298-396 397-495 496-594 595-693 694-792 793-891 892-990 991-1089 1090-1188 1189-1287 1288-1386 1387-1485 1486-1584 1585-1682 | RAMP1 Description: ADMreceptor activity modifying protein 1 GO: 1-26 Reactome: 1-13 UniProtKB: 1-2 RefEx: ADM Expression Atlas: RAMP1 Single Cell Expression Atlas: RAMP1 scRNASeqDB: RAMP1 PanglaoDB: RAMP1 CMap: RAMP1 MeSH: 1-100 101-200 201-300 301-399 |  |  | 0.906 (6.219%) | 0 | 0 | DLRP | 1 |
| 8 | BMP2 Description: BMP2bone morphogenetic protein 2 GO: 1-57 58-113 Reactome: 1-11 UniProtKB: 1-2 STRING: 1 RefEx: BMP2 Expression Atlas: BMP2 Single Cell Expression Atlas: BMP2 scRNASeqDB: BMP2 PanglaoDB: BMP2 CMap: BMP2 MeSH: 1-98 99-196 197-294 295-392 393-490 491-588 589-686 687-784 785-882 883-980 981-1078 1079-1176 1177-1274 1275-1372 1373-1470 1471-1568 1569-1666 1667-1764 1765-1862 1863-1960 1961-2058 2059-2156 2157-2254 2255-2352 2353-2450 2451-2548 2549-2646 2647-2742 | BMPR2 Description: BMP2bone morphogenetic protein receptor type 2 GO: 1-91 Reactome: 1-3 UniProtKB: 1 RefEx: BMP2 Expression Atlas: BMPR2 Single Cell Expression Atlas: BMPR2 scRNASeqDB: BMPR2 PanglaoDB: BMPR2 CMap: BMPR2 MeSH: 1-100 101-200 201-300 301-400 401-500 501-600 601-700 701-800 801-900 901-1000 1001-1098 |  |  | 0.728 (4.998%) | 0 | 0 | DLRP | 1 |
| 9 | APOE Description: APOEapolipoprotein E GO: 1-74 75-147 Reactome: 1-34 UniProtKB: 1-2 RefEx: APOE Expression Atlas: APOE Single Cell Expression Atlas: APOE scRNASeqDB: APOE PanglaoDB: APOE CMap: APOE MeSH: 1-99 100-198 199-297 298-396 397-495 496-594 595-693 694-792 793-891 892-990 991-1089 1090-1188 1189-1287 1288-1386 1387-1485 1486-1584 1585-1683 1684-1782 1783-1881 1882-1980 1981-2079 2080-2178 2179-2277 2278-2376 2377-2475 2476-2574 2575-2673 2674-2772 2773-2871 2872-2970 2971-3069 3070-3168 3169-3267 3268-3366 3367-3465 3466-3564 3565-3663 3664-3762 3763-3861 3862-3960 3961-4059 4060-4158 4159-4257 4258-4338 | VLDLR Description: APOEvery low density lipoprotein receptor GO: 1-29 Reactome: 1-9 UniProtKB: 1-2 RefEx: APOE Expression Atlas: VLDLR Single Cell Expression Atlas: VLDLR scRNASeqDB: VLDLR PanglaoDB: VLDLR CMap: VLDLR MeSH: 1-88 89-176 177-264 265-352 353-440 441-528 529-616 617-702 |  |  | 0.703 (4.826%) | 0 | 0 | DLRP | 1 |
| 10 | JAG1 Description: JAG1jagged canonical Notch ligand 1 GO: 1-53 Reactome: 1-31 UniProtKB: 1-2 RefEx: JAG1 Expression Atlas: JAG1 Single Cell Expression Atlas: JAG1 scRNASeqDB: JAG1 PanglaoDB: JAG1 CMap: JAG1 MeSH: 1-99 100-198 199-297 298-396 397-495 496-594 595-693 694-792 793-891 892-990 991-1089 1090-1188 1189-1287 1288-1386 1387-1485 1486-1584 1585-1683 | NOTCH2 Description: JAG1notch receptor 2 GO: 1-63 Reactome: 1-20 UniProtKB: 1-3 RefEx: JAG1 Expression Atlas: NOTCH2 Single Cell Expression Atlas: NOTCH2 scRNASeqDB: NOTCH2 PanglaoDB: NOTCH2 CMap: NOTCH2 MeSH: 1-93 94-186 187-279 280-372 373-465 466-558 559-651 652-744 745-837 838-930 931-1023 1024-1116 1117-1203 |  |  | 0.59 (4.054%) | 0.003 | 0.005 | DLRP | 1 |
| 11 | EFNA5 Description: EFNA5ephrin A5 GO: 1-23 Reactome: 1-6 UniProtKB: 1-2 RefEx: EFNA5 Expression Atlas: EFNA5 Single Cell Expression Atlas: EFNA5 scRNASeqDB: EFNA5 PanglaoDB: EFNA5 CMap: EFNA5 MeSH: 1-100 101-200 201-300 301-398 | EPHA4 Description: EFNA5EPH receptor A4 GO: 1-77 Reactome: 1-6 UniProtKB: 1-2 RefEx: EFNA5 Expression Atlas: EPHA4 Single Cell Expression Atlas: EPHA4 scRNASeqDB: EPHA4 PanglaoDB: EPHA4 CMap: EPHA4 MeSH: 1-93 94-186 187-279 280-372 373-465 466-557 |  |  | 0.589 (4.041%) | 0 | 0 | DLRP | 1 |

# (3,3,) -related Enrichment Analysis

```
load("reanalysis.RData")
library("plotly")
```

## MeSH-Enrichment Analysis (A : Anatomy)

```
negLogPval <- -log10(out.vecLR[['pattern_3_3']]$Enrich$MeSH_A$Pvalue + 1E-10)
term <- out.vecLR[['pattern_3_3']]$Enrich$MeSH_A$Term
target <- seq_len(min(100, length(negLogPval)))
negLogPval <- negLogPval[target]
term <- term[target]
p <- plot_ly(x=seq_along(negLogPval), y=~negLogPval,
type="bar", color=~negLogPval, text=term,
colors=c("#4b61ba", "gray", "#a87963", "red"))
layout(p, title="MeSH-Enrichment Analysis (A)",
   xaxis=list(title="Term"),
   yaxis=list(title="-Log(P-value)"),
   legend=list(name=""))
```

```
## Warning: textfont.color doesn't (yet) support data arrays

## Warning: textfont.color doesn't (yet) support data arrays
```

## MeSH-Enrichment Analysis (B : Organisms)

```
negLogPval <- -log10(out.vecLR[['pattern_3_3']]$Enrich$MeSH_B$Pvalue + 1E-10)
term <- out.vecLR[['pattern_3_3']]$Enrich$MeSH_B$Term
target <- seq_len(min(100, length(negLogPval)))
negLogPval <- negLogPval[target]
term <- term[target]
p <- plot_ly(x=seq_along(negLogPval), y=~negLogPval,
type="bar", color=~negLogPval, text=term,
colors=c("#4b61ba", "gray", "#a87963", "red"))
layout(p, title="MeSH-Enrichment Analysis (B)",
   xaxis=list(title="Term"),
   yaxis=list(title="-Log(P-value)"),
   legend=list(name=""))
```

```
## Warning: textfont.color doesn't (yet) support data arrays

## Warning: textfont.color doesn't (yet) support data arrays
```

## MeSH-Enrichment Analysis (C : Diseases)

```
negLogPval <- -log10(out.vecLR[['pattern_3_3']]$Enrich$MeSH_C$Pvalue + 1E-10)
term <- out.vecLR[['pattern_3_3']]$Enrich$MeSH_C$Term
target <- seq_len(min(100, length(negLogPval)))
negLogPval <- negLogPval[target]
term <- term[target]
p <- plot_ly(x=seq_along(negLogPval), y=~negLogPval,
type="bar", color=~negLogPval, text=term,
colors=c("#4b61ba", "gray", "#a87963", "red"))
layout(p, title="MeSH-Enrichment Analysis (C)",
   xaxis=list(title="Term"),
   yaxis=list(title="-Log(P-value)"),
   legend=list(name=""))
```

```
## Warning: textfont.color doesn't (yet) support data arrays

## Warning: textfont.color doesn't (yet) support data arrays
```

## MeSH-Enrichment Analysis (D : Drugs)

```
negLogPval <- -log10(out.vecLR[['pattern_3_3']]$Enrich$MeSH_D$Pvalue + 1E-10)
term <- out.vecLR[['pattern_3_3']]$Enrich$MeSH_D$Term
target <- seq_len(min(100, length(negLogPval)))
negLogPval <- negLogPval[target]
term <- term[target]
p <- plot_ly(x=seq_along(negLogPval), y=~negLogPval,
type="bar", color=~negLogPval, text=term,
colors=c("#4b61ba", "gray", "#a87963", "red"))
layout(p, title="MeSH-Enrichment Analysis (D)",
   xaxis=list(title="Term"),
   yaxis=list(title="-Log(P-value)"),
   legend=list(name=""))
```

```
## Warning: textfont.color doesn't (yet) support data arrays

## Warning: textfont.color doesn't (yet) support data arrays
```

## MeSH-Enrichment Analysis (E : Analytical, Diagnostic and Therapeutic Techniques and Equipment)

```
negLogPval <- -log10(out.vecLR[['pattern_3_3']]$Enrich$MeSH_E$Pvalue + 1E-10)
term <- out.vecLR[['pattern_3_3']]$Enrich$MeSH_E$Term
target <- seq_len(min(100, length(negLogPval)))
negLogPval <- negLogPval[target]
term <- term[target]
p <- plot_ly(x=seq_along(negLogPval), y=~negLogPval,
type="bar", color=~negLogPval, text=term,
colors=c("#4b61ba", "gray", "#a87963", "red"))
layout(p, title="MeSH-Enrichment Analysis (E)",
   xaxis=list(title="Term"),
   yaxis=list(title="-Log(P-value)"),
   legend=list(name=""))
```

```
## Warning: textfont.color doesn't (yet) support data arrays

## Warning: textfont.color doesn't (yet) support data arrays
```

## MeSH-Enrichment Analysis (F : Psychiatry and Psychology)

```
negLogPval <- -log10(out.vecLR[['pattern_3_3']]$Enrich$MeSH_F$Pvalue + 1E-10)
term <- out.vecLR[['pattern_3_3']]$Enrich$MeSH_F$Term
target <- seq_len(min(100, length(negLogPval)))
negLogPval <- negLogPval[target]
term <- term[target]
p <- plot_ly(x=seq_along(negLogPval), y=~negLogPval,
type="bar", color=~negLogPval, text=term,
colors=c("#4b61ba", "gray", "#a87963", "red"))
layout(p, title="MeSH-Enrichment Analysis (F)",
   xaxis=list(title="Term"),
   yaxis=list(title="-Log(P-value)"),
   legend=list(name=""))
```

```
## Warning: textfont.color doesn't (yet) support data arrays

## Warning: textfont.color doesn't (yet) support data arrays
```

## MeSH-Enrichment Analysis (G : Phenomena and Processes)

```
negLogPval <- -log10(out.vecLR[['pattern_3_3']]$Enrich$MeSH_G$Pvalue + 1E-10)
term <- out.vecLR[['pattern_3_3']]$Enrich$MeSH_G$Term
target <- seq_len(min(100, length(negLogPval)))
negLogPval <- negLogPval[target]
term <- term[target]
p <- plot_ly(x=seq_along(negLogPval), y=~negLogPval,
type="bar", color=~negLogPval, text=term,
colors=c("#4b61ba", "gray", "#a87963", "red"))
layout(p, title="MeSH-Enrichment Analysis (G)",
   xaxis=list(title="Term"),
   yaxis=list(title="-Log(P-value)"),
   legend=list(name=""))
```

```
## Warning: textfont.color doesn't (yet) support data arrays

## Warning: textfont.color doesn't (yet) support data arrays
```

## MeSH-Enrichment Analysis (I : Anthropology, Education, Sociology and Social Phenomena)

```
negLogPval <- -log10(out.vecLR[['pattern_3_3']]$Enrich$MeSH_I$Pvalue + 1E-10)
term <- out.vecLR[['pattern_3_3']]$Enrich$MeSH_I$Term
target <- seq_len(min(100, length(negLogPval)))
negLogPval <- negLogPval[target]
term <- term[target]
p <- plot_ly(x=seq_along(negLogPval), y=~negLogPval,
type="bar", color=~negLogPval, text=term,
colors=c("#4b61ba", "gray", "#a87963", "red"))
layout(p, title="MeSH-Enrichment Analysis (I)",
   xaxis=list(title="Term"),
   yaxis=list(title="-Log(P-value)"),
   legend=list(name=""))
```

```
## Warning: textfont.color doesn't (yet) support data arrays

## Warning: textfont.color doesn't (yet) support data arrays
```

## MeSH-Enrichment Analysis (J : Technology and Food and Beverages)

```
negLogPval <- -log10(out.vecLR[['pattern_3_3']]$Enrich$MeSH_J$Pvalue + 1E-10)
term <- out.vecLR[['pattern_3_3']]$Enrich$MeSH_J$Term
target <- seq_len(min(100, length(negLogPval)))
negLogPval <- negLogPval[target]
term <- term[target]
p <- plot_ly(x=seq_along(negLogPval), y=~negLogPval,
type="bar", color=~negLogPval, text=term,
colors=c("#4b61ba", "gray", "#a87963", "red"))
layout(p, title="MeSH-Enrichment Analysis (J)",
   xaxis=list(title="Term"),
   yaxis=list(title="-Log(P-value)"),
   legend=list(name=""))
```

```
## Warning: textfont.color doesn't (yet) support data arrays

## Warning: textfont.color doesn't (yet) support data arrays
```

## MeSH-Enrichment Analysis (K : Humanities)

```
negLogPval <- -log10(out.vecLR[['pattern_3_3']]$Enrich$MeSH_K$Pvalue + 1E-10)
term <- out.vecLR[['pattern_3_3']]$Enrich$MeSH_K$Term
target <- seq_len(min(100, length(negLogPval)))
negLogPval <- negLogPval[target]
term <- term[target]
p <- plot_ly(x=seq_along(negLogPval), y=~negLogPval,
type="bar", color=~negLogPval, text=term,
colors=c("#4b61ba", "gray", "#a87963", "red"))
layout(p, title="MeSH-Enrichment Analysis (K)",
   xaxis=list(title="Term"),
   yaxis=list(title="-Log(P-value)"),
   legend=list(name=""))
```

## MeSH-Enrichment Analysis (M : Persons)

```
negLogPval <- -log10(out.vecLR[['pattern_3_3']]$Enrich$MeSH_M$Pvalue + 1E-10)
term <- out.vecLR[['pattern_3_3']]$Enrich$MeSH_M$Term
target <- seq_len(min(100, length(negLogPval)))
negLogPval <- negLogPval[target]
term <- term[target]
p <- plot_ly(x=seq_along(negLogPval), y=~negLogPval,
type="bar", color=~negLogPval, text=term,
colors=c("#4b61ba", "gray", "#a87963", "red"))
layout(p, title="MeSH-Enrichment Analysis (M)",
   xaxis=list(title="Term"),
   yaxis=list(title="-Log(P-value)"),
   legend=list(name=""))
```

```
## Warning: textfont.color doesn't (yet) support data arrays

## Warning: textfont.color doesn't (yet) support data arrays
```

## MeSH-Enrichment Analysis (N : Health Care)

```
negLogPval <- -log10(out.vecLR[['pattern_3_3']]$Enrich$MeSH_N$Pvalue + 1E-10)
term <- out.vecLR[['pattern_3_3']]$Enrich$MeSH_N$Term
target <- seq_len(min(100, length(negLogPval)))
negLogPval <- negLogPval[target]
term <- term[target]
p <- plot_ly(x=seq_along(negLogPval), y=~negLogPval,
type="bar", color=~negLogPval, text=term,
colors=c("#4b61ba", "gray", "#a87963", "red"))
layout(p, title="MeSH-Enrichment Analysis (N)",
   xaxis=list(title="Term"),
   yaxis=list(title="-Log(P-value)"),
   legend=list(name=""))
```

```
## Warning: textfont.color doesn't (yet) support data arrays

## Warning: textfont.color doesn't (yet) support data arrays
```

## Reactome Pathway-Enrichment Analysis

```
negLogPval <- -log10(out.vecLR[['pattern_3_3']]$Enrich$Reactome$Pvalue + 1E-10)
term <- out.vecLR[['pattern_3_3']]$Enrich$Reactome$Term
target <- seq_len(min(100, length(negLogPval)))
negLogPval <- negLogPval[target]
term <- term[target]
p <- plot_ly(x=seq_along(negLogPval), y=~negLogPval,
type="bar", color=~negLogPval, text=term,
colors=c("#4b61ba", "gray", "#a87963", "red"))
layout(p, title="Reactome-Enrichment Analysis",
   xaxis=list(title="Term"),
   yaxis=list(title="-Log(P-value)"),
   legend=list(name=""))
```

```
## Warning: textfont.color doesn't (yet) support data arrays

## Warning: textfont.color doesn't (yet) support data arrays
```

## DO-Enrichment Analysis

```
negLogPval <- -log10(out.vecLR[['pattern_3_3']]$Enrich$DO$Pvalue + 1E-10)
term <- out.vecLR[['pattern_3_3']]$Enrich$DO$Term
target <- seq_len(min(100, length(negLogPval)))
negLogPval <- negLogPval[target]
term <- term[target]
p <- plot_ly(x=seq_along(negLogPval), y=~negLogPval,
type="bar", color=~negLogPval, text=term,
colors=c("#4b61ba", "gray", "#a87963", "red"))
layout(p, title="DO-Enrichment Analysis",
   xaxis=list(title="Term"),
   yaxis=list(title="-Log(P-value)"),
   legend=list(name=""))
```

```
## Warning: textfont.color doesn't (yet) support data arrays

## Warning: textfont.color doesn't (yet) support data arrays
```

## NCG-Enrichment Analysis

```
negLogPval <- -log10(out.vecLR[['pattern_3_3']]$Enrich$NCG$Pvalue + 1E-10)
term <- out.vecLR[['pattern_3_3']]$Enrich$NCG$Term
target <- seq_len(min(100, length(negLogPval)))
negLogPval <- negLogPval[target]
term <- term[target]
p <- plot_ly(x=seq_along(negLogPval), y=~negLogPval,
type="bar", color=~negLogPval, text=term,
colors=c("#4b61ba", "gray", "#a87963", "red"))
layout(p, title="NCG-Enrichment Analysis",
   xaxis=list(title="Term"),
   yaxis=list(title="-Log(P-value)"),
   legend=list(name=""))
```

```
## Warning: textfont.color doesn't (yet) support data arrays

## Warning: textfont.color doesn't (yet) support data arrays
```

## DGN-Enrichment Analysis

```
negLogPval <- -log10(out.vecLR[['pattern_3_3']]$Enrich$DGN$Pvalue + 1E-10)
term <- out.vecLR[['pattern_3_3']]$Enrich$DGN$Term
target <- seq_len(min(100, length(negLogPval)))
negLogPval <- negLogPval[target]
term <- term[target]
p <- plot_ly(x=seq_along(negLogPval), y=~negLogPval,
type="bar", color=~negLogPval, text=term,
colors=c("#4b61ba", "gray", "#a87963", "red"))
layout(p, title="DGN-Enrichment Analysis",
   xaxis=list(title="Term"),
   yaxis=list(title="-Log(P-value)"),
   legend=list(name=""))
```

```
## Warning: textfont.color doesn't (yet) support data arrays

## Warning: textfont.color doesn't (yet) support data arrays
```
