# Supplementary material for: Sctensor detects many-to-many cell–cell interactions from single cell RNA-sequencing data
Source: BMC Bioinformatics. 2023 Nov 7;24:420. doi: 10.1186/s12859-023-05490-y (PMC10631077; doi:10.1186/s12859-023-05490-y)
Supplement: Supplementary file 15 — Additional file 15. HTML report of GermlineFemale. [file 12859_2023_5490_MOESM15_ESM.zip › output/report/Human_Germline_Female/pattern_5_2.html]

Details of (5,2,) -related L-R pairs


# Details of (5,2,) -related L-R pairs

# (5,2,:) Pattern

# (5,2,) -related L-R pairs (Top6 pairs)

```
library("scTensor")
load("reanalysis.RData")
col <- .setColor("many")[14]
scTensor:::.myvisNetwork(g, col)
```

| Rank | Ligand Gene | Receptor Gene | Ligand Expression (Log10(exp + 1)) | Receptor Expression (Log10(exp + 1)) | LR-pair factor value (Percentage) | P-value (Grubbs test) | Q-value (BH method) | L-R Evidence cf. rikenbit/lrbase-workflow | PubMed |
| --- | --- | --- | --- | --- | --- | --- | --- | --- | --- |
| 1 | APOE Description: APOEapolipoprotein E GO: 1-74 75-147 Reactome: 1-34 UniProtKB: 1-2 RefEx: APOE Expression Atlas: APOE Single Cell Expression Atlas: APOE scRNASeqDB: APOE PanglaoDB: APOE CMap: APOE MeSH: 1-99 100-198 199-297 298-396 397-495 496-594 595-693 694-792 793-891 892-990 991-1089 1090-1188 1189-1287 1288-1386 1387-1485 1486-1584 1585-1683 1684-1782 1783-1881 1882-1980 1981-2079 2080-2178 2179-2277 2278-2376 2377-2475 2476-2574 2575-2673 2674-2772 2773-2871 2872-2970 2971-3069 3070-3168 3169-3267 3268-3366 3367-3465 3466-3564 3565-3663 3664-3762 3763-3861 3862-3960 3961-4059 4060-4158 4159-4257 4258-4338 | VLDLR Description: APOEvery low density lipoprotein receptor GO: 1-29 Reactome: 1-9 UniProtKB: 1-2 RefEx: APOE Expression Atlas: VLDLR Single Cell Expression Atlas: VLDLR scRNASeqDB: VLDLR PanglaoDB: VLDLR CMap: VLDLR MeSH: 1-88 89-176 177-264 265-352 353-440 441-528 529-616 617-702 |  |  | 2.428 (22.529%) | 0.002 | 0.003 | DLRP | 1 |
| 2 | APOE Description: APOEapolipoprotein E GO: 1-74 75-147 Reactome: 1-34 UniProtKB: 1-2 RefEx: APOE Expression Atlas: APOE Single Cell Expression Atlas: APOE scRNASeqDB: APOE PanglaoDB: APOE CMap: APOE MeSH: 1-99 100-198 199-297 298-396 397-495 496-594 595-693 694-792 793-891 892-990 991-1089 1090-1188 1189-1287 1288-1386 1387-1485 1486-1584 1585-1683 1684-1782 1783-1881 1882-1980 1981-2079 2080-2178 2179-2277 2278-2376 2377-2475 2476-2574 2575-2673 2674-2772 2773-2871 2872-2970 2971-3069 3070-3168 3169-3267 3268-3366 3367-3465 3466-3564 3565-3663 3664-3762 3763-3861 3862-3960 3961-4059 4060-4158 4159-4257 4258-4338 | SORL1 Description: APOEsortilin related receptor 1 GO: 1-65 Reactome: 1-2 UniProtKB: 1-2 RefEx: APOE Expression Atlas: SORL1 Single Cell Expression Atlas: SORL1 scRNASeqDB: SORL1 PanglaoDB: SORL1 CMap: SORL1 MeSH: 1-92 93-184 185-276 277-368 369-460 461-552 553-644 645-734 |  |  | 1.515 (14.06%) | 0 | 0 | DLRP | 1 |
| 3 | APOE Description: APOEapolipoprotein E GO: 1-74 75-147 Reactome: 1-34 UniProtKB: 1-2 RefEx: APOE Expression Atlas: APOE Single Cell Expression Atlas: APOE scRNASeqDB: APOE PanglaoDB: APOE CMap: APOE MeSH: 1-99 100-198 199-297 298-396 397-495 496-594 595-693 694-792 793-891 892-990 991-1089 1090-1188 1189-1287 1288-1386 1387-1485 1486-1584 1585-1683 1684-1782 1783-1881 1882-1980 1981-2079 2080-2178 2179-2277 2278-2376 2377-2475 2476-2574 2575-2673 2674-2772 2773-2871 2872-2970 2971-3069 3070-3168 3169-3267 3268-3366 3367-3465 3466-3564 3565-3663 3664-3762 3763-3861 3862-3960 3961-4059 4060-4158 4159-4257 4258-4338 | LDLR Description: APOElow density lipoprotein receptor GO: 1-66 Reactome: 1-15 UniProtKB: 1-2 RefEx: APOE Expression Atlas: LDLR Single Cell Expression Atlas: LDLR scRNASeqDB: LDLR PanglaoDB: LDLR CMap: LDLR MeSH: 1-99 100-198 199-297 298-396 397-495 496-594 595-693 694-792 793-891 892-990 991-1089 1090-1188 1189-1287 1288-1386 1387-1485 1486-1584 1585-1683 1684-1779 |  |  | 0.972 (9.018%) | 0 | 0 | DLRP | 1 |
| 4 | COL4A1 Description: COL4A1collagen type IV alpha 1 chain GO: 1-22 Reactome: 1-24 UniProtKB: 1-3 RefEx: COL4A1 Expression Atlas: COL4A1 Single Cell Expression Atlas: COL4A1 scRNASeqDB: COL4A1 PanglaoDB: COL4A1 CMap: COL4A1 MeSH: 1-94 95-188 189-282 283-376 377-470 471-564 565-658 659-752 753-846 847-940 941-1032 | ITGA1 Description: COL4A1integrin subunit alpha 1 GO: 1-29 Reactome: 1-14 UniProtKB: 1 RefEx: COL4A1 Expression Atlas: ITGA1 Single Cell Expression Atlas: ITGA1 scRNASeqDB: ITGA1 PanglaoDB: ITGA1 CMap: ITGA1 MeSH: 1-91 92-182 183-273 274-364 365-455 456-546 547-637 638-728 729-817 |  |  | 0.921 (8.546%) | 0 | 0 | DLRP | 1 |
| 5 | GAS6 Description: GAS6growth arrest specific 6 GO: 1-76 Reactome: 1-14 UniProtKB: 1 RefEx: GAS6 Expression Atlas: GAS6 Single Cell Expression Atlas: GAS6 scRNASeqDB: GAS6 PanglaoDB: GAS6 CMap: GAS6 MeSH: 1-98 99-196 197-294 295-392 393-490 491-588 589-686 687-778 | AXL Description: GAS6AXL receptor tyrosine kinase GO: 1-60 Reactome: 1-4 UniProtKB: 1-2 RefEx: GAS6 Expression Atlas: AXL Single Cell Expression Atlas: AXL scRNASeqDB: AXL PanglaoDB: AXL CMap: AXL MeSH: 1-93 94-186 187-279 280-372 373-465 466-558 559-651 652-744 745-837 838-930 931-1013 |  |  | 0.765 (7.095%) | 0.002 | 0.003 | DLRP | 1 |
| 6 | DLK1 Description: DLK1delta like non-canonical Notch ligand 1 GO: 1-9 Reactome: 1-4 UniProtKB: 1-3 RefEx: DLK1 Expression Atlas: DLK1 Single Cell Expression Atlas: DLK1 scRNASeqDB: DLK1 PanglaoDB: DLK1 CMap: DLK1 MeSH: 1-92 93-184 185-276 277-368 369-460 461-552 553-644 645-730 | NOTCH2 Description: DLK1notch receptor 2 GO: 1-63 Reactome: 1-20 UniProtKB: 1-3 RefEx: DLK1 Expression Atlas: NOTCH2 Single Cell Expression Atlas: NOTCH2 scRNASeqDB: NOTCH2 PanglaoDB: NOTCH2 CMap: NOTCH2 MeSH: 1-93 94-186 187-279 280-372 373-465 466-558 559-651 652-744 745-837 838-930 931-1023 1024-1116 1117-1203 |  |  | 0.614 (5.697%) | 0 | 0 | DLRP | 1 |

# (5,2,) -related Enrichment Analysis

```
load("reanalysis.RData")
library("plotly")
```

## MeSH-Enrichment Analysis (A : Anatomy)

```
negLogPval <- -log10(out.vecLR[['pattern_5_2']]$Enrich$MeSH_A$Pvalue + 1E-10)
term <- out.vecLR[['pattern_5_2']]$Enrich$MeSH_A$Term
target <- seq_len(min(100, length(negLogPval)))
negLogPval <- negLogPval[target]
term <- term[target]
p <- plot_ly(x=seq_along(negLogPval), y=~negLogPval,
type="bar", color=~negLogPval, text=term,
colors=c("#4b61ba", "gray", "#a87963", "red"))
layout(p, title="MeSH-Enrichment Analysis (A)",
   xaxis=list(title="Term"),
   yaxis=list(title="-Log(P-value)"),
   legend=list(name=""))
```

```
## Warning: textfont.color doesn't (yet) support data arrays

## Warning: textfont.color doesn't (yet) support data arrays
```

## MeSH-Enrichment Analysis (B : Organisms)

```
negLogPval <- -log10(out.vecLR[['pattern_5_2']]$Enrich$MeSH_B$Pvalue + 1E-10)
term <- out.vecLR[['pattern_5_2']]$Enrich$MeSH_B$Term
target <- seq_len(min(100, length(negLogPval)))
negLogPval <- negLogPval[target]
term <- term[target]
p <- plot_ly(x=seq_along(negLogPval), y=~negLogPval,
type="bar", color=~negLogPval, text=term,
colors=c("#4b61ba", "gray", "#a87963", "red"))
layout(p, title="MeSH-Enrichment Analysis (B)",
   xaxis=list(title="Term"),
   yaxis=list(title="-Log(P-value)"),
   legend=list(name=""))
```

```
## Warning: textfont.color doesn't (yet) support data arrays

## Warning: textfont.color doesn't (yet) support data arrays
```

## MeSH-Enrichment Analysis (C : Diseases)

```
negLogPval <- -log10(out.vecLR[['pattern_5_2']]$Enrich$MeSH_C$Pvalue + 1E-10)
term <- out.vecLR[['pattern_5_2']]$Enrich$MeSH_C$Term
target <- seq_len(min(100, length(negLogPval)))
negLogPval <- negLogPval[target]
term <- term[target]
p <- plot_ly(x=seq_along(negLogPval), y=~negLogPval,
type="bar", color=~negLogPval, text=term,
colors=c("#4b61ba", "gray", "#a87963", "red"))
layout(p, title="MeSH-Enrichment Analysis (C)",
   xaxis=list(title="Term"),
   yaxis=list(title="-Log(P-value)"),
   legend=list(name=""))
```

```
## Warning: textfont.color doesn't (yet) support data arrays

## Warning: textfont.color doesn't (yet) support data arrays
```

## MeSH-Enrichment Analysis (D : Drugs)

```
negLogPval <- -log10(out.vecLR[['pattern_5_2']]$Enrich$MeSH_D$Pvalue + 1E-10)
term <- out.vecLR[['pattern_5_2']]$Enrich$MeSH_D$Term
target <- seq_len(min(100, length(negLogPval)))
negLogPval <- negLogPval[target]
term <- term[target]
p <- plot_ly(x=seq_along(negLogPval), y=~negLogPval,
type="bar", color=~negLogPval, text=term,
colors=c("#4b61ba", "gray", "#a87963", "red"))
layout(p, title="MeSH-Enrichment Analysis (D)",
   xaxis=list(title="Term"),
   yaxis=list(title="-Log(P-value)"),
   legend=list(name=""))
```

```
## Warning: textfont.color doesn't (yet) support data arrays

## Warning: textfont.color doesn't (yet) support data arrays
```

## MeSH-Enrichment Analysis (E : Analytical, Diagnostic and Therapeutic Techniques and Equipment)

```
negLogPval <- -log10(out.vecLR[['pattern_5_2']]$Enrich$MeSH_E$Pvalue + 1E-10)
term <- out.vecLR[['pattern_5_2']]$Enrich$MeSH_E$Term
target <- seq_len(min(100, length(negLogPval)))
negLogPval <- negLogPval[target]
term <- term[target]
p <- plot_ly(x=seq_along(negLogPval), y=~negLogPval,
type="bar", color=~negLogPval, text=term,
colors=c("#4b61ba", "gray", "#a87963", "red"))
layout(p, title="MeSH-Enrichment Analysis (E)",
   xaxis=list(title="Term"),
   yaxis=list(title="-Log(P-value)"),
   legend=list(name=""))
```

```
## Warning: textfont.color doesn't (yet) support data arrays

## Warning: textfont.color doesn't (yet) support data arrays
```

## MeSH-Enrichment Analysis (F : Psychiatry and Psychology)

```
negLogPval <- -log10(out.vecLR[['pattern_5_2']]$Enrich$MeSH_F$Pvalue + 1E-10)
term <- out.vecLR[['pattern_5_2']]$Enrich$MeSH_F$Term
target <- seq_len(min(100, length(negLogPval)))
negLogPval <- negLogPval[target]
term <- term[target]
p <- plot_ly(x=seq_along(negLogPval), y=~negLogPval,
type="bar", color=~negLogPval, text=term,
colors=c("#4b61ba", "gray", "#a87963", "red"))
layout(p, title="MeSH-Enrichment Analysis (F)",
   xaxis=list(title="Term"),
   yaxis=list(title="-Log(P-value)"),
   legend=list(name=""))
```

```
## Warning: textfont.color doesn't (yet) support data arrays

## Warning: textfont.color doesn't (yet) support data arrays
```

## MeSH-Enrichment Analysis (G : Phenomena and Processes)

```
negLogPval <- -log10(out.vecLR[['pattern_5_2']]$Enrich$MeSH_G$Pvalue + 1E-10)
term <- out.vecLR[['pattern_5_2']]$Enrich$MeSH_G$Term
target <- seq_len(min(100, length(negLogPval)))
negLogPval <- negLogPval[target]
term <- term[target]
p <- plot_ly(x=seq_along(negLogPval), y=~negLogPval,
type="bar", color=~negLogPval, text=term,
colors=c("#4b61ba", "gray", "#a87963", "red"))
layout(p, title="MeSH-Enrichment Analysis (G)",
   xaxis=list(title="Term"),
   yaxis=list(title="-Log(P-value)"),
   legend=list(name=""))
```

```
## Warning: textfont.color doesn't (yet) support data arrays

## Warning: textfont.color doesn't (yet) support data arrays
```

## MeSH-Enrichment Analysis (H : Disciplines and Occupations)

```
negLogPval <- -log10(out.vecLR[['pattern_5_2']]$Enrich$MeSH_H$Pvalue + 1E-10)
term <- out.vecLR[['pattern_5_2']]$Enrich$MeSH_H$Term
target <- seq_len(min(100, length(negLogPval)))
negLogPval <- negLogPval[target]
term <- term[target]
p <- plot_ly(x=seq_along(negLogPval), y=~negLogPval,
type="bar", color=~negLogPval, text=term,
colors=c("#4b61ba", "gray", "#a87963", "red"))
layout(p, title="MeSH-Enrichment Analysis (H)",
   xaxis=list(title="Term"),
   yaxis=list(title="-Log(P-value)"),
   legend=list(name=""))
```

```
## Warning: textfont.color doesn't (yet) support data arrays

## Warning: textfont.color doesn't (yet) support data arrays
```

## MeSH-Enrichment Analysis (I : Anthropology, Education, Sociology and Social Phenomena)

```
negLogPval <- -log10(out.vecLR[['pattern_5_2']]$Enrich$MeSH_I$Pvalue + 1E-10)
term <- out.vecLR[['pattern_5_2']]$Enrich$MeSH_I$Term
target <- seq_len(min(100, length(negLogPval)))
negLogPval <- negLogPval[target]
term <- term[target]
p <- plot_ly(x=seq_along(negLogPval), y=~negLogPval,
type="bar", color=~negLogPval, text=term,
colors=c("#4b61ba", "gray", "#a87963", "red"))
layout(p, title="MeSH-Enrichment Analysis (I)",
   xaxis=list(title="Term"),
   yaxis=list(title="-Log(P-value)"),
   legend=list(name=""))
```

```
## Warning: textfont.color doesn't (yet) support data arrays

## Warning: textfont.color doesn't (yet) support data arrays
```

## MeSH-Enrichment Analysis (J : Technology and Food and Beverages)

```
negLogPval <- -log10(out.vecLR[['pattern_5_2']]$Enrich$MeSH_J$Pvalue + 1E-10)
term <- out.vecLR[['pattern_5_2']]$Enrich$MeSH_J$Term
target <- seq_len(min(100, length(negLogPval)))
negLogPval <- negLogPval[target]
term <- term[target]
p <- plot_ly(x=seq_along(negLogPval), y=~negLogPval,
type="bar", color=~negLogPval, text=term,
colors=c("#4b61ba", "gray", "#a87963", "red"))
layout(p, title="MeSH-Enrichment Analysis (J)",
   xaxis=list(title="Term"),
   yaxis=list(title="-Log(P-value)"),
   legend=list(name=""))
```

## MeSH-Enrichment Analysis (L : Information Science)

```
negLogPval <- -log10(out.vecLR[['pattern_5_2']]$Enrich$MeSH_L$Pvalue + 1E-10)
term <- out.vecLR[['pattern_5_2']]$Enrich$MeSH_L$Term
target <- seq_len(min(100, length(negLogPval)))
negLogPval <- negLogPval[target]
term <- term[target]
p <- plot_ly(x=seq_along(negLogPval), y=~negLogPval,
type="bar", color=~negLogPval, text=term,
colors=c("#4b61ba", "gray", "#a87963", "red"))
layout(p, title="MeSH-Enrichment Analysis (L)",
   xaxis=list(title="Term"),
   yaxis=list(title="-Log(P-value)"),
   legend=list(name=""))
```

```
## Warning: textfont.color doesn't (yet) support data arrays

## Warning: textfont.color doesn't (yet) support data arrays
```

## MeSH-Enrichment Analysis (M : Persons)

```
negLogPval <- -log10(out.vecLR[['pattern_5_2']]$Enrich$MeSH_M$Pvalue + 1E-10)
term <- out.vecLR[['pattern_5_2']]$Enrich$MeSH_M$Term
target <- seq_len(min(100, length(negLogPval)))
negLogPval <- negLogPval[target]
term <- term[target]
p <- plot_ly(x=seq_along(negLogPval), y=~negLogPval,
type="bar", color=~negLogPval, text=term,
colors=c("#4b61ba", "gray", "#a87963", "red"))
layout(p, title="MeSH-Enrichment Analysis (M)",
   xaxis=list(title="Term"),
   yaxis=list(title="-Log(P-value)"),
   legend=list(name=""))
```

```
## Warning: textfont.color doesn't (yet) support data arrays

## Warning: textfont.color doesn't (yet) support data arrays
```

## MeSH-Enrichment Analysis (N : Health Care)

```
negLogPval <- -log10(out.vecLR[['pattern_5_2']]$Enrich$MeSH_N$Pvalue + 1E-10)
term <- out.vecLR[['pattern_5_2']]$Enrich$MeSH_N$Term
target <- seq_len(min(100, length(negLogPval)))
negLogPval <- negLogPval[target]
term <- term[target]
p <- plot_ly(x=seq_along(negLogPval), y=~negLogPval,
type="bar", color=~negLogPval, text=term,
colors=c("#4b61ba", "gray", "#a87963", "red"))
layout(p, title="MeSH-Enrichment Analysis (N)",
   xaxis=list(title="Term"),
   yaxis=list(title="-Log(P-value)"),
   legend=list(name=""))
```

```
## Warning: textfont.color doesn't (yet) support data arrays

## Warning: textfont.color doesn't (yet) support data arrays
```

## MeSH-Enrichment Analysis (Z : Geographical Locations)

```
negLogPval <- -log10(out.vecLR[['pattern_5_2']]$Enrich$MeSH_Z$Pvalue + 1E-10)
term <- out.vecLR[['pattern_5_2']]$Enrich$MeSH_Z$Term
target <- seq_len(min(100, length(negLogPval)))
negLogPval <- negLogPval[target]
term <- term[target]
p <- plot_ly(x=seq_along(negLogPval), y=~negLogPval,
type="bar", color=~negLogPval, text=term,
colors=c("#4b61ba", "gray", "#a87963", "red"))
layout(p, title="MeSH-Enrichment Analysis (Z)",
   xaxis=list(title="Term"),
   yaxis=list(title="-Log(P-value)"),
   legend=list(name=""))
```

```
## Warning: textfont.color doesn't (yet) support data arrays

## Warning: textfont.color doesn't (yet) support data arrays
```

## Reactome Pathway-Enrichment Analysis

```
negLogPval <- -log10(out.vecLR[['pattern_5_2']]$Enrich$Reactome$Pvalue + 1E-10)
term <- out.vecLR[['pattern_5_2']]$Enrich$Reactome$Term
target <- seq_len(min(100, length(negLogPval)))
negLogPval <- negLogPval[target]
term <- term[target]
p <- plot_ly(x=seq_along(negLogPval), y=~negLogPval,
type="bar", color=~negLogPval, text=term,
colors=c("#4b61ba", "gray", "#a87963", "red"))
layout(p, title="Reactome-Enrichment Analysis",
   xaxis=list(title="Term"),
   yaxis=list(title="-Log(P-value)"),
   legend=list(name=""))
```

```
## Warning: textfont.color doesn't (yet) support data arrays

## Warning: textfont.color doesn't (yet) support data arrays
```

## DO-Enrichment Analysis

```
negLogPval <- -log10(out.vecLR[['pattern_5_2']]$Enrich$DO$Pvalue + 1E-10)
term <- out.vecLR[['pattern_5_2']]$Enrich$DO$Term
target <- seq_len(min(100, length(negLogPval)))
negLogPval <- negLogPval[target]
term <- term[target]
p <- plot_ly(x=seq_along(negLogPval), y=~negLogPval,
type="bar", color=~negLogPval, text=term,
colors=c("#4b61ba", "gray", "#a87963", "red"))
layout(p, title="DO-Enrichment Analysis",
   xaxis=list(title="Term"),
   yaxis=list(title="-Log(P-value)"),
   legend=list(name=""))
```

```
## Warning: textfont.color doesn't (yet) support data arrays

## Warning: textfont.color doesn't (yet) support data arrays
```

## NCG-Enrichment Analysis

```
negLogPval <- -log10(out.vecLR[['pattern_5_2']]$Enrich$NCG$Pvalue + 1E-10)
term <- out.vecLR[['pattern_5_2']]$Enrich$NCG$Term
target <- seq_len(min(100, length(negLogPval)))
negLogPval <- negLogPval[target]
term <- term[target]
p <- plot_ly(x=seq_along(negLogPval), y=~negLogPval,
type="bar", color=~negLogPval, text=term,
colors=c("#4b61ba", "gray", "#a87963", "red"))
layout(p, title="NCG-Enrichment Analysis",
   xaxis=list(title="Term"),
   yaxis=list(title="-Log(P-value)"),
   legend=list(name=""))
```

```
## Warning: textfont.color doesn't (yet) support data arrays

## Warning: textfont.color doesn't (yet) support data arrays
```

## DGN-Enrichment Analysis

```
negLogPval <- -log10(out.vecLR[['pattern_5_2']]$Enrich$DGN$Pvalue + 1E-10)
term <- out.vecLR[['pattern_5_2']]$Enrich$DGN$Term
target <- seq_len(min(100, length(negLogPval)))
negLogPval <- negLogPval[target]
term <- term[target]
p <- plot_ly(x=seq_along(negLogPval), y=~negLogPval,
type="bar", color=~negLogPval, text=term,
colors=c("#4b61ba", "gray", "#a87963", "red"))
layout(p, title="DGN-Enrichment Analysis",
   xaxis=list(title="Term"),
   yaxis=list(title="-Log(P-value)"),
   legend=list(name=""))
```

```
## Warning: textfont.color doesn't (yet) support data arrays

## Warning: textfont.color doesn't (yet) support data arrays
```
