# Supplementary material for: Sctensor detects many-to-many cell–cell interactions from single cell RNA-sequencing data
Source: BMC Bioinformatics. 2023 Nov 7;24:420. doi: 10.1186/s12859-023-05490-y (PMC10631077; doi:10.1186/s12859-023-05490-y)
Supplement: Supplementary file 16 — Additional file 16. HTML report of HeadandNeckCancer. [file 12859_2023_5490_MOESM16_ESM.zip › output/report/Human_HeadandNeckCancer/ligand_all.html]

Details of Ligand Gene-centric Overview (all)


# Details of Ligand Gene-centric Overview (all)

| Ligand Gene | Receptor Gene |
| --- | --- |
| ADAM15 | ITGB3 |
| ADAM2 | ITGA6 ITGB1 |
| ADAM23 | ITGA5 ITGB3 |
| ADIPOQ | ADIPOR1 |
| ADM | CALCR CALCRL MRGPRX2 RAMP1 RAMP2 RAMP3 GPR182 |
| ADM2 | CALCR RAMP1 CALCRL |
| AGRN | MUSK |
| AGT | MAS1 |
| ALOX5AP | ALOX5 |
| AMH | ACVR1 AMHR2 BMPR1A BMPR1B |
| ANGPT1 | TEK |
| ANGPT2 | TEK |
| ANGPT4 | TEK |
| ANGPTL1 | TEK |
| APOB | LDLR |
| APOE | SORL1 LDLR |
| AREG | EGFR |
| ASIP | ATRN MC1R MGRN1 |
| AVP | AVPR1A AVPR1B AVPR2 OXTR |
| BDNF | NTRK2 |
| BMP10 | BMPR1A ACVR1 |
| BMP15 | BMPR1A ACVR1 |
| BMP2 | BMPR1A ACVR1 |
| BMP3 | ACVR1 ACVR2B BMPR1A BMPR1B BMPR2 |
| BMP4 | BMPR1A ACVR1 |
| BMP5 | BMPR1A ACVR1 |
| BMP6 | BMPR1A ACVR1 |
| BMP7 | ACVR1 ACVR2A ACVR2B ACVRL1 BMPR1A BMPR1B BMPR2 |
| BMP8A | BMPR1A ACVR1 |
| BMP8B | BMPR1A ACVR1 |
| BTC | EGFR ERBB4 |
| C10orf99 | GPR15 |
| C3 | C3AR1 ITGAX ITGB2 LRP1 |
| CALCA | CALCR RAMP1 CALCRL |
| CALCB | CALCR RAMP1 CALCRL |
| CALM1 | PDE1A |
| CALM2 | MYLK MYLK2 PDE1A PDE1B PDE1C |
| CALM3 | PDE1A |
| CAMP | FPR2 P2RX7 |
| CCL15 | CCR1 LOC105377067 CCR3 |
| CCL19 | ACKR4 CCR7 CCRL2 CXCR3 |
| CCL20 | CCR6 |
| CCL21 | ACKR4 CCR7 |
| CCL26 | CCR2 LOC105377067 CCR3 |
| CCL3 | CCR1 CCR5 |
| CCL5 | GPR75 |
| CD24 | SELP |
| CD274 | PDCD1 |
| CD34 | SELL |
| CD40LG | CD40 |
| CDH1 | ITGB7 |
| CFH | SELL |
| CGA | LHCGR |
| CGB3 | LHCGR |
| CHAD | ITGA2 ITGB1 |
| CLCF1 | CNTFR |
| CLUL1 | GPR55 |
| CNTF | CNTFR |
| COL18A1 | GPC1 ITGA5 ITGB1 |
| COL1A1 | DDR2 |
| COL2A1 | ITGA10 ITGA2 ITGB1 |
| COL4A1 | ITGB8 |
| COL5A3 | SDC3 |
| CORT | SSTR3 |
| CRH | CRHR2 |
| CRLF1 | CNTFR |
| CSHL1 | PRLR |
| CXCL1 | CXCR2 CXCR1 |
| CXCL13 | CXCR5 |
| CXCL2 | XCR1 CXCR2 |
| CXCL3 | CXCR2 |
| CXCL5 | CXCR2 |
| CXCL6 | CXCR1 CXCR2 |
| CXCL8 | CXCR2 |
| DEFB4A | CCR6 |
| DEFB4B | CCR6 |
| DKK1 | LRP6 |
| DKK2 | LRP6 |
| DLK1 | NOTCH3 |
| DLL1 | NOTCH3 |
| DLL3 | NOTCH3 |
| DLL4 | NOTCH1 NOTCH2 NOTCH3 NOTCH4 |
| DUSP18 | ITGA7 RPSA |
| EBI3 | IL27RA |
| EDA | EDAR EDA2R |
| EDN3 | EDNRB |
| EFNA1 | EPHA3 EPHA6 EPHA2 |
| EFNA2 | EPHA3 EPHA6 EPHA2 |
| EFNA3 | EPHA3 EPHA6 EPHA2 |
| EFNA4 | EPHA3 EPHA6 EPHA2 |
| EFNA5 | EPHA3 EPHA6 EPHB2 EPHA2 |
| EFNB1 | EPHB2 |
| EFNB2 | EPHB2 |
| EFNB3 | EPHB2 |
| EGF | EGFR |
| ENHO | GPR19 |
| EPGN | EGFR |
| EREG | EGFR ERBB4 |
| F8 | LRP1 |
| FGA | ITGAX ITGB2 |
| FGF1 | FGFR2 |
| FGF10 | FGFR1 FGFR2 FGFR3 FGFR4 |
| FGF11 | FGFR2 |
| FGF12 | FGFR2 |
| FGF13 | FGFR2 |
| FGF14 | FGFR1 FGFR2 FGFR3 FGFR4 |
| FGF16 | FGFR2 |
| FGF17 | FGFR2 |
| FGF18 | FGFR2 |
| FGF19 | FGFR4 FGFR1 FGFR2 FGFR3 |
| FGF2 | SDC2 FGFR2 |
| FGF22 | FGFR2 |
| FGF23 | FGFR2 |
| FGF3 | FGFR2 |
| FGF4 | FGFR1 FGFR2 FGFR3 FGFR4 |
| FGF5 | FGFR2 |
| FGF6 | FGFR2 |
| FGF7 | FGFR2 |
| FGF8 | FGFR2 |
| FGF9 | FGFR2 |
| FN1 | SDC2 ITGB8 ITGB3 |
| GAD1 | GRM4 |
| GAL | GALR1 GALR2 GALR3 |
| GALP | GALR3 |
| GAS6 | TYRO3 |
| GDF1 | BMPR1A |
| GDF10 | ACVR1B ACVR1C ACVR2A ACVR2B BMPR1A BMPR1B BMPR2 TGFBR1 |
| GDF15 | GFRAL |
| GDF2 | BMPR1A ACVR1 |
| GDF3 | BMPR1A |
| GDF5 | BMPR1B BMPR2 ROR2 |
| GDF6 | BMPR1A |
| GDF7 | BMPR1A |
| GDF9 | BMPR1A |
| GH1 | GHR |
| GH2 | GHR |
| GHRH | GHRHR |
| GHRL | GHSR GPR39 |
| GPI | AMFR |
| GSTP1 | TRAF2 |
| GUCA1A | GUCY2D GUCY2F |
| GUCA1B | GUCY2D |
| HBEGF | EGFR ERBB4 |
| HDC | HRH1 |
| HEBP1 | FPR3 |
| HLA-A | KIR3DL2 |
| HLA-B | KIR3DL1 |
| HLA-C | KIR2DL1 KIR2DL3 KIR2DS4 |
| HSP90B1 | LRP1 |
| IAPP | CALCR RAMP1 |
| ICAM3 | ITGAL ITGB2 |
| ICAM4 | ITGAM ITGB3 |
| IFNA21 | IFNAR1 IFNAR2 |
| IFNL3 | IFNLR1 IL10RB |
| IGF1 | IGF1R |
| IHH | PTCH1 |
| IL13 | IL2RG IL4R |
| IL15 | IL2RG |
| IL17A | IL17RA IL17RC |
| IL17B | IL17RB |
| IL17C | IL17RE |
| IL19 | IL20RA |
| IL1A | IL1R1 IL1RAP IL1R2 |
| IL1B | IL1R1 |
| IL1R1 | IL1R2 |
| IL1RN | IL1R1 IL1RAP IL2RA IL2RB IL2RG |
| IL2 | IL2RG |
| IL20 | IL20RA |
| IL21 | IL2RG IL21R |
| IL22 | IL10RB IL22RA1 IL10RA IL22RA2 |
| IL24 | IL20RA |
| IL25 | IL17RB |
| IL27 | IL27RA IL6ST |
| IL3 | CSF2RB IL3RA |
| IL31 | IL31RA |
| IL33 | IL1RAP IL1RL1 |
| IL36RN | IL1RAP IL1RL2 |
| IL37 | IL18R1 IL18RAP SIGIRR |
| IL4 | IL2RG IL4R |
| IL7 | IL2RG IL7R |
| IL9 | IL2RG |
| INHA | ACVR1 |
| INHBA | ACVR1 |
| INHBB | ACVR1 |
| INHBC | ACVR1 |
| INSL3 | RXFP1 RXFP2 |
| INSL5 | RXFP3 |
| JAG1 | NOTCH3 |
| JAG2 | NOTCH1 NOTCH2 NOTCH3 NOTCH4 |
| KISS1 | KISS1R |
| KITLG | KIT |
| LAMA1 | ITGB8 ITGA7 RPSA |
| LAMA2 | ITGA7 RPSA |
| LAMB2 | RPSA |
| LAMC2 | ITGA3 ITGA6 ITGB1 ITGB4 |
| LEAP2 | GHSR |
| LHB | LHCGR |
| LIF | IL6ST LIFR |
| LOC101927057 | TNFRSF1B |
| LOC101929777 | LRP6 |
| LOC118142757 | GUCY2D |
| LTA | TNFRSF14 TNFRSF1A TNFRSF1B LTBR |
| LTB | CD40 |
| LTF | LRP11 |
| MADCAM1 | ITGA4 ITGB7 |
| MDK | PTPRB PTPRZ1 ALK |
| MMP13 | LRP1 |
| MST1 | MST1R |
| NCAM1 | ROBO3 |
| NDP | FZD4 LGR4 |
| NMS | NMUR2 |
| NMU | NMUR2 |
| NPFF | NPFFR1 NPFFR2 |
| NPPA | NPR1 NPR3 |
| NPPB | NPR1 |
| NPVF | NPFFR1 |
| NPY | PRLHR |
| NRG1 | ERBB3 ERBB4 ERBB2 |
| NRG2 | ERBB4 |
| NRG3 | ERBB4 |
| NRG4 | ERBB4 |
| NTF3 | NGFR NTRK3 NTRK1 NTRK2 |
| NTF4 | NTRK2 |
| NTN1 | DCC UNC5C |
| NTN4 | UNC5A |
| NTNG1 | LRRC4C |
| NTS | NTSR2 |
| NXPH1 | NRXN1 NRXN2 NRXN3 |
| OMG | TNFRSF1B |
| OSTN | NPR3 |
| OXT | AVPR1A AVPR1B AVPR2 OXTR |
| PDYN | OPRD1 OPRK1 OPRM1 |
| PENK | MRGPRX1 |
| PF4 | LDLR FGFR2 |
| PLG | ITGA9 ITGB1 |
| PODXL2 | SELL |
| PPBP | CXCR2 |
| PRL | PRLR |
| PRLH | PRLHR |
| PRND | RPSA |
| PROK1 | PROKR1 |
| PROK2 | PROKR1 |
| PROS1 | AXL TYRO3 |
| PSAP | GPR37 |
| PSPN | GFRA4 |
| PTGS2 | PTGDR2 |
| PTH | PTH1R PTH2R |
| PTHLH | PRLHR |
| PTN | PTPRB |
| QRFP | QRFPR P2RY14 |
| RLN1 | RXFP1 RXFP2 |
| RLN2 | RXFP3 |
| RLN3 | RXFP1 RXFP2 RXFP3 RXFP4 |
| RSPO1 | LGR5 LRP6 |
| RSPO2 | LGR5 |
| RSPO3 | LGR5 |
| RSPO4 | LGR4 LGR5 LGR6 |
| RTN4 | TNFRSF19 |
| SCT | SCTR VIPR1 VIPR2 |
| SELPLG | SELP |
| SEMA3A | PLXNA1 |
| SEMA3E | PLXND1 |
| SEMA6D | KDR PLXNA1 |
| SERPING1 | LRP1 |
| SHH | PTCH1 |
| SLIT2 | GPC1 ROBO1 ROBO2 ROBO4 SDC1 |
| SOST | LRP6 |
| SPP1 | CD44 ITGAV |
| SPX | GALR3 |
| SST | MRGPRX2 SSTR1 SSTR2 SSTR3 SSTR4 SSTR5 |
| TAC1 | MRGPRX2 TACR1 TACR2 TACR3 |
| TAC3 | TACR2 |
| TF | TFRC |
| TFPI | LRP1 VLDLR |
| TGFA | EGFR |
| TGFB1 | TGFBR2 |
| TGFB2 | TGFBR2 |
| TGFB3 | TGFBR2 |
| THBS1 | CD47 |
| THBS2 | CD47 |
| TNF | TNFRSF1A TNFRSF1B LTBR TNFRSF21 |
| TNFSF10 | TNFRSF10A TNFRSF10B TNFRSF10C TNFRSF10D TNFRSF11B |
| TNFSF12 | TNFRSF12A |
| TNFSF13 | TNFRSF13B |
| TNFSF13B | TNFRSF13B TNFRSF13C TNFRSF17 |
| TNFSF15 | TNFRSF25 |
| TNFSF4 | TNFRSF4 |
| TNFSF8 | TNFRSF8 |
| TNFSF9 | TNFRSF9 |
| TPH1 | HTR4 HTR2B |
| UCN | CRHR2 |
| UCN2 | CRHR2 |
| UCN3 | CRHR2 |
| ULBP2 | KLRC4-KLRK1 |
| UTS2 | UTS2R |
| UTS2B | UTS2R |
| VCAM1 | ITGAD |
| VEGFB | FLT1 NRP1 |
| VTN | ITGB3 |
| WNT1 | FZD3 FZD8 FZD9 RYK |
| WNT11 | FZD7 |
| WNT3 | FZD8 LRP6 RYK |
| WNT3A | FZD6 |
| WNT4 | FZD6 |
| WNT5A | FZD6 |
| XCL1 | XCR1 |
| XCL2 | XCR1 |
