# Supplementary material for: Sctensor detects many-to-many cell–cell interactions from single cell RNA-sequencing data
Source: BMC Bioinformatics. 2023 Nov 7;24:420. doi: 10.1186/s12859-023-05490-y (PMC10631077; doi:10.1186/s12859-023-05490-y)
Supplement: Supplementary file 16 — Additional file 16. HTML report of HeadandNeckCancer. [file 12859_2023_5490_MOESM16_ESM.zip › output/report/Human_HeadandNeckCancer/receptor.html]

Details of Receptor Gene-centric Overview (selected)


# Details of Receptor Gene-centric Overview (selected)

```
library("scTensor")
load("reanalysis.RData")
scTensor:::.myvisNetwork(g)
```

| Rank | No. of related pairs | Receptor Gene | Ligand Genes | Related CCIs |
| --- | --- | --- | --- | --- |
| 1 | 6 | CD47 | THBS2 | See the details of (3,2,)-related L-R patterns See the details of (3,4,)-related L-R patterns See the details of (3,3,)-related L-R patterns See the details of (2,2,)-related L-R patterns See the details of (4,2,)-related L-R patterns See the details of (4,3,)-related L-R patterns |
| 3 | 5 | IL2RG | IL1RN  IL7 | See the details of (4,4,)-related L-R patterns See the details of (1,4,)-related L-R patterns See the details of (5,4,)-related L-R patterns See the details of (4,1,)-related L-R patterns |
| 3 | 5 | RAMP1 | ADM | See the details of (2,1,)-related L-R patterns See the details of (1,1,)-related L-R patterns See the details of (3,1,)-related L-R patterns See the details of (2,2,)-related L-R patterns See the details of (4,1,)-related L-R patterns |
| 3 | 5 | CALCRL | ADM | See the details of (2,3,)-related L-R patterns See the details of (3,3,)-related L-R patterns See the details of (2,2,)-related L-R patterns See the details of (1,3,)-related L-R patterns See the details of (4,3,)-related L-R patterns |
| 7 | 4 | PTPRB | MDK | See the details of (2,3,)-related L-R patterns See the details of (3,3,)-related L-R patterns See the details of (4,3,)-related L-R patterns See the details of (5,3,)-related L-R patterns |
| 7 | 4 | RPSA | PRND | See the details of (5,2,)-related L-R patterns See the details of (5,4,)-related L-R patterns See the details of (5,3,)-related L-R patterns See the details of (5,1,)-related L-R patterns |
| 7 | 4 | EGFR | HBEGF  EREG | See the details of (2,2,)-related L-R patterns See the details of (1,2,)-related L-R patterns See the details of (4,2,)-related L-R patterns |
| 7 | 4 | TNFRSF1B | TNF | See the details of (1,4,)-related L-R patterns See the details of (5,4,)-related L-R patterns See the details of (4,3,)-related L-R patterns See the details of (5,3,)-related L-R patterns |
| 7 | 4 | IL1R1 | IL1RN  IL1A | See the details of (4,1,)-related L-R patterns See the details of (4,2,)-related L-R patterns See the details of (4,3,)-related L-R patterns |
| 10 | 2 | PDE1A | CALM2 | See the details of (4,3,)-related L-R patterns See the details of (5,3,)-related L-R patterns |
| 11 | 1 | PLXNA1 | SEMA6D | See the details of (2,2,)-related L-R patterns |
