# Supplementary material for: Sctensor detects many-to-many cell–cell interactions from single cell RNA-sequencing data
Source: BMC Bioinformatics. 2023 Nov 7;24:420. doi: 10.1186/s12859-023-05490-y (PMC10631077; doi:10.1186/s12859-023-05490-y)
Supplement: Supplementary file 16 — Additional file 16. HTML report of HeadandNeckCancer. [file 12859_2023_5490_MOESM16_ESM.zip › output/report/Human_HeadandNeckCancer/receptor_all.html]

Details of Receptor Gene-centric Overview (all)


# Details of Receptor Gene-centric Overview (all)

| Receptor Gene | Ligand Gene |
| --- | --- |
| ACKR4 | CCL19 CCL21 |
| ACVR1 | BMP3 BMP7 AMH BMP8B BMP10 BMP2 BMP4 GDF2 BMP6 BMP5 BMP8A INHA INHBB INHBC INHBA BMP15 |
| ACVR1B | GDF10 |
| ACVR1C | GDF10 |
| ACVR2A | GDF10 BMP7 |
| ACVR2B | BMP3 GDF10 BMP7 |
| ACVRL1 | BMP7 |
| ADIPOR1 | ADIPOQ |
| ALK | MDK |
| ALOX5 | ALOX5AP |
| AMFR | GPI |
| AMHR2 | AMH |
| ATRN | ASIP |
| AVPR1A | OXT AVP |
| AVPR1B | OXT AVP |
| AVPR2 | OXT AVP |
| AXL | PROS1 |
| BMPR1A | BMP3 GDF10 BMP7 AMH GDF9 BMP5 BMP8B GDF2 GDF3 BMP10 GDF1 BMP4 BMP6 GDF7 BMP2 BMP8A BMP15 GDF6 |
| BMPR1B | BMP3 GDF10 BMP7 AMH GDF5 |
| BMPR2 | BMP3 GDF10 BMP7 GDF5 |
| C3AR1 | C3 |
| CALCR | ADM CALCA ADM2 CALCB IAPP |
| CALCRL | ADM CALCA ADM2 CALCB |
| CCR1 | CCL15 CCL3 |
| CCR2 | CCL26 |
| CCR3 | CCL15 CCL26 |
| CCR5 | CCL3 |
| CCR6 | DEFB4B DEFB4A CCL20 |
| CCR7 | CCL19 CCL21 |
| CCRL2 | CCL19 |
| CD40 | CD40LG LTB |
| CD44 | SPP1 |
| CD47 | THBS2 THBS1 |
| CNTFR | CLCF1 CRLF1 CNTF |
| CRHR2 | UCN UCN2 CRH UCN3 |
| CSF2RB | IL3 |
| CXCR1 | CXCL1 CXCL6 |
| CXCR2 | CXCL1 CXCL6 CXCL8 CXCL3 PPBP CXCL5 CXCL2 |
| CXCR3 | CCL19 |
| CXCR5 | CXCL13 |
| DCC | NTN1 |
| DDR2 | COL1A1 |
| EDA2R | EDA |
| EDAR | EDA |
| EDNRB | EDN3 |
| EGFR | HBEGF EREG BTC TGFA AREG EGF EPGN |
| EPHA2 | EFNA3 EFNA2 EFNA4 EFNA1 EFNA5 |
| EPHA3 | EFNA1 EFNA4 EFNA5 EFNA2 EFNA3 |
| EPHA6 | EFNA1 EFNA4 EFNA2 EFNA5 EFNA3 |
| EPHB2 | EFNB3 EFNB2 EFNB1 EFNA5 |
| ERBB2 | NRG1 |
| ERBB3 | NRG1 |
| ERBB4 | HBEGF EREG NRG1 BTC NRG2 NRG3 NRG4 |
| FGFR1 | FGF10 FGF4 FGF14 FGF19 |
| FGFR2 | FGF10 FGF4 FGF14 FGF19 FGF7 FGF6 FGF13 FGF18 FGF11 FGF12 FGF17 FGF16 FGF3 FGF1 FGF2 FGF8 FGF5 FGF9 FGF22 FGF23 PF4 |
| FGFR3 | FGF10 FGF4 FGF14 FGF19 |
| FGFR4 | FGF10 FGF4 FGF14 FGF19 |
| FLT1 | VEGFB |
| FPR2 | CAMP |
| FPR3 | HEBP1 |
| FZD3 | WNT1 |
| FZD4 | NDP |
| FZD6 | WNT3A WNT5A WNT4 |
| FZD7 | WNT11 |
| FZD8 | WNT3 WNT1 |
| FZD9 | WNT1 |
| GALR1 | GAL |
| GALR2 | GAL |
| GALR3 | GAL GALP SPX |
| GFRA4 | PSPN |
| GFRAL | GDF15 |
| GHR | GH2 GH1 |
| GHRHR | GHRH |
| GHSR | LEAP2 GHRL |
| GPC1 | COL18A1 SLIT2 |
| GPR15 | C10orf99 |
| GPR182 | ADM |
| GPR19 | ENHO |
| GPR37 | PSAP |
| GPR39 | GHRL |
| GPR55 | CLUL1 |
| GPR75 | CCL5 |
| GRM4 | GAD1 |
| GUCY2D | GUCA1A GUCA1B LOC118142757 |
| GUCY2F | GUCA1A |
| HRH1 | HDC |
| HTR2B | TPH1 |
| HTR4 | TPH1 |
| IFNAR1 | IFNA21 |
| IFNAR2 | IFNA21 |
| IFNLR1 | IFNL3 |
| IGF1R | IGF1 |
| IL10RA | IL22 |
| IL10RB | IFNL3 IL22 |
| IL17RA | IL17A |
| IL17RB | IL17B IL25 |
| IL17RC | IL17A |
| IL17RE | IL17C |
| IL18R1 | IL37 |
| IL18RAP | IL37 |
| IL1R1 | IL1A IL1RN IL1B |
| IL1R2 | IL1R1 IL1A |
| IL1RAP | IL36RN IL33 IL1A IL1RN |
| IL1RL1 | IL33 |
| IL1RL2 | IL36RN |
| IL20RA | IL19 IL24 IL20 |
| IL21R | IL21 |
| IL22RA1 | IL22 |
| IL22RA2 | IL22 |
| IL27RA | IL27 EBI3 |
| IL2RA | IL1RN |
| IL2RB | IL1RN |
| IL2RG | IL7 IL1RN IL15 IL9 IL4 IL2 IL21 IL13 |
| IL31RA | IL31 |
| IL3RA | IL3 |
| IL4R | IL4 IL13 |
| IL6ST | IL27 LIF |
| IL7R | IL7 |
| ITGA10 | COL2A1 |
| ITGA2 | CHAD COL2A1 |
| ITGA3 | LAMC2 |
| ITGA4 | MADCAM1 |
| ITGA5 | COL18A1 ADAM23 |
| ITGA6 | ADAM2 LAMC2 |
| ITGA7 | LAMA1 DUSP18 LAMA2 |
| ITGA9 | PLG |
| ITGAD | VCAM1 |
| ITGAL | ICAM3 |
| ITGAM | ICAM4 |
| ITGAV | SPP1 |
| ITGAX | FGA C3 |
| ITGB1 | COL18A1 PLG ADAM2 CHAD LAMC2 COL2A1 |
| ITGB2 | ICAM3 FGA C3 |
| ITGB3 | ADAM23 ICAM4 FN1 VTN ADAM15 |
| ITGB4 | LAMC2 |
| ITGB7 | MADCAM1 CDH1 |
| ITGB8 | LAMA1 COL4A1 FN1 |
| KDR | SEMA6D |
| KIR2DL1 | HLA-C |
| KIR2DL3 | HLA-C |
| KIR2DS4 | HLA-C |
| KIR3DL1 | HLA-B |
| KIR3DL2 | HLA-A |
| KISS1R | KISS1 |
| KIT | KITLG |
| KLRC4-KLRK1 | ULBP2 |
| LDLR | PF4 APOE APOB |
| LGR4 | NDP RSPO4 |
| LGR5 | RSPO4 RSPO3 RSPO1 RSPO2 |
| LGR6 | RSPO4 |
| LHCGR | LHB CGA CGB3 |
| LIFR | LIF |
| LOC105377067 | CCL15 CCL26 |
| LRP1 | MMP13 F8 C3 SERPING1 TFPI HSP90B1 |
| LRP11 | LTF |
| LRP6 | DKK2 WNT3 RSPO1 DKK1 LOC101929777 SOST |
| LRRC4C | NTNG1 |
| LTBR | TNF LTA |
| MAS1 | AGT |
| MC1R | ASIP |
| MGRN1 | ASIP |
| MRGPRX1 | PENK |
| MRGPRX2 | TAC1 SST ADM |
| MST1R | MST1 |
| MUSK | AGRN |
| MYLK | CALM2 |
| MYLK2 | CALM2 |
| NGFR | NTF3 |
| NMUR2 | NMS NMU |
| NOTCH1 | DLL4 JAG2 |
| NOTCH2 | DLL4 JAG2 |
| NOTCH3 | DLL4 JAG2 DLK1 DLL1 JAG1 DLL3 |
| NOTCH4 | DLL4 JAG2 |
| NPFFR1 | NPFF NPVF |
| NPFFR2 | NPFF |
| NPR1 | NPPA NPPB |
| NPR3 | OSTN NPPA |
| NRP1 | VEGFB |
| NRXN1 | NXPH1 |
| NRXN2 | NXPH1 |
| NRXN3 | NXPH1 |
| NTRK1 | NTF3 |
| NTRK2 | NTF3 BDNF NTF4 |
| NTRK3 | NTF3 |
| NTSR2 | NTS |
| OPRD1 | PDYN |
| OPRK1 | PDYN |
| OPRM1 | PDYN |
| OXTR | OXT AVP |
| P2RX7 | CAMP |
| P2RY14 | QRFP |
| PDCD1 | CD274 |
| PDE1A | CALM2 CALM1 CALM3 |
| PDE1B | CALM2 |
| PDE1C | CALM2 |
| PLXNA1 | SEMA6D SEMA3A |
| PLXND1 | SEMA3E |
| PRLHR | PRLH PTHLH NPY |
| PRLR | CSHL1 PRL |
| PROKR1 | PROK2 PROK1 |
| PTCH1 | SHH IHH |
| PTGDR2 | PTGS2 |
| PTH1R | PTH |
| PTH2R | PTH |
| PTPRB | MDK PTN |
| PTPRZ1 | MDK |
| QRFPR | QRFP |
| RAMP1 | ADM CALCB CALCA ADM2 IAPP |
| RAMP2 | ADM |
| RAMP3 | ADM |
| ROBO1 | SLIT2 |
| ROBO2 | SLIT2 |
| ROBO3 | NCAM1 |
| ROBO4 | SLIT2 |
| ROR2 | GDF5 |
| RPSA | PRND LAMA1 LAMB2 LAMA2 DUSP18 |
| RXFP1 | INSL3 RLN1 RLN3 |
| RXFP2 | INSL3 RLN1 RLN3 |
| RXFP3 | RLN3 RLN2 INSL5 |
| RXFP4 | RLN3 |
| RYK | WNT3 WNT1 |
| SCTR | SCT |
| SDC1 | SLIT2 |
| SDC2 | FGF2 FN1 |
| SDC3 | COL5A3 |
| SELL | CD34 CFH PODXL2 |
| SELP | SELPLG CD24 |
| SIGIRR | IL37 |
| SORL1 | APOE |
| SSTR1 | SST |
| SSTR2 | SST |
| SSTR3 | SST CORT |
| SSTR4 | SST |
| SSTR5 | SST |
| TACR1 | TAC1 |
| TACR2 | TAC1 TAC3 |
| TACR3 | TAC1 |
| TEK | ANGPT4 ANGPT1 ANGPTL1 ANGPT2 |
| TFRC | TF |
| TGFBR1 | GDF10 |
| TGFBR2 | TGFB2 TGFB1 TGFB3 |
| TNFRSF10A | TNFSF10 |
| TNFRSF10B | TNFSF10 |
| TNFRSF10C | TNFSF10 |
| TNFRSF10D | TNFSF10 |
| TNFRSF11B | TNFSF10 |
| TNFRSF12A | TNFSF12 |
| TNFRSF13B | TNFSF13B TNFSF13 |
| TNFRSF13C | TNFSF13B |
| TNFRSF14 | LTA |
| TNFRSF17 | TNFSF13B |
| TNFRSF19 | RTN4 |
| TNFRSF1A | TNF LTA |
| TNFRSF1B | TNF LTA LOC101927057 OMG |
| TNFRSF21 | TNF |
| TNFRSF25 | TNFSF15 |
| TNFRSF4 | TNFSF4 |
| TNFRSF8 | TNFSF8 |
| TNFRSF9 | TNFSF9 |
| TRAF2 | GSTP1 |
| TYRO3 | PROS1 GAS6 |
| UNC5A | NTN4 |
| UNC5C | NTN1 |
| UTS2R | UTS2 UTS2B |
| VIPR1 | SCT |
| VIPR2 | SCT |
| VLDLR | TFPI |
| XCR1 | XCL1 XCL2 CXCL2 |
