# Supplementary material for: Sctensor detects many-to-many cell–cell interactions from single cell RNA-sequencing data
Source: BMC Bioinformatics. 2023 Nov 7;24:420. doi: 10.1186/s12859-023-05490-y (PMC10631077; doi:10.1186/s12859-023-05490-y)
Supplement: Supplementary file 16 — Additional file 16. HTML report of HeadandNeckCancer. [file 12859_2023_5490_MOESM16_ESM.zip › output/report/Human_HeadandNeckCancer/pattern_4_4.html]

Details of (4,4,) -related L-R pairs


# Details of (4,4,) -related L-R pairs

# (4,4,:) Pattern

# (4,4,) -related L-R pairs (Top1 pairs)

```
library("scTensor")
load("reanalysis.RData")
col <- .setColor("many")[2]
scTensor:::.myvisNetwork(g, col)
```

| Rank | Ligand Gene | Receptor Gene | Ligand Expression (Log10(exp + 1)) | Receptor Expression (Log10(exp + 1)) | LR-pair factor value (Percentage) | P-value (Grubbs test) | Q-value (BH method) | L-R Evidence cf. rikenbit/lrbase-workflow | PubMed |
| --- | --- | --- | --- | --- | --- | --- | --- | --- | --- |
| 1 | IL1RN Description: IL1RNinterleukin 1 receptor antagonist GO: 1-20 Reactome: 1-6 UniProtKB: 1-2 RefEx: IL1RN Expression Atlas: IL1RN Single Cell Expression Atlas: IL1RN scRNASeqDB: IL1RN PanglaoDB: IL1RN CMap: IL1RN MeSH: 1-98 99-196 197-294 295-392 393-490 491-588 589-686 687-784 785-882 883-980 981-1078 1079-1176 1177-1274 1275-1372 1373-1470 1471-1568 1569-1666 1667-1764 1765-1862 1863-1960 1961-2058 2059-2156 2157-2254 2255-2352 2353-2450 2451-2548 2549-2646 2647-2744 2745-2842 2843-2940 2941-3038 3039-3136 3137-3234 3235-3332 3333-3430 3431-3502 | IL2RG Description: IL1RNinterleukin 2 receptor subunit gamma GO: 1-24 Reactome: 1-19 UniProtKB: 1 RefEx: IL1RN Expression Atlas: IL2RG Single Cell Expression Atlas: IL2RG scRNASeqDB: IL2RG PanglaoDB: IL2RG CMap: IL2RG MeSH: 1-99 100-198 199-297 298-396 397-495 496-594 595-693 694-788 |  |  | 1.602 (86.596%) | 0 | 0 | DLRP | 1 |

# (4,4,) -related Enrichment Analysis

```
load("reanalysis.RData")
library("plotly")
```

## MeSH-Enrichment Analysis (A : Anatomy)

```
negLogPval <- -log10(out.vecLR[['pattern_4_4']]$Enrich$MeSH_A$Pvalue + 1E-10)
term <- out.vecLR[['pattern_4_4']]$Enrich$MeSH_A$Term
target <- seq_len(min(100, length(negLogPval)))
negLogPval <- negLogPval[target]
term <- term[target]
p <- plot_ly(x=seq_along(negLogPval), y=~negLogPval,
type="bar", color=~negLogPval, text=term,
colors=c("#4b61ba", "gray", "#a87963", "red"))
layout(p, title="MeSH-Enrichment Analysis (A)",
   xaxis=list(title="Term"),
   yaxis=list(title="-Log(P-value)"),
   legend=list(name=""))
```

```
## Warning: textfont.color doesn't (yet) support data arrays

## Warning: textfont.color doesn't (yet) support data arrays
```

## MeSH-Enrichment Analysis (B : Organisms)

```
negLogPval <- -log10(out.vecLR[['pattern_4_4']]$Enrich$MeSH_B$Pvalue + 1E-10)
term <- out.vecLR[['pattern_4_4']]$Enrich$MeSH_B$Term
target <- seq_len(min(100, length(negLogPval)))
negLogPval <- negLogPval[target]
term <- term[target]
p <- plot_ly(x=seq_along(negLogPval), y=~negLogPval,
type="bar", color=~negLogPval, text=term,
colors=c("#4b61ba", "gray", "#a87963", "red"))
layout(p, title="MeSH-Enrichment Analysis (B)",
   xaxis=list(title="Term"),
   yaxis=list(title="-Log(P-value)"),
   legend=list(name=""))
```

```
## Warning: textfont.color doesn't (yet) support data arrays

## Warning: textfont.color doesn't (yet) support data arrays
```

## MeSH-Enrichment Analysis (C : Diseases)

```
negLogPval <- -log10(out.vecLR[['pattern_4_4']]$Enrich$MeSH_C$Pvalue + 1E-10)
term <- out.vecLR[['pattern_4_4']]$Enrich$MeSH_C$Term
target <- seq_len(min(100, length(negLogPval)))
negLogPval <- negLogPval[target]
term <- term[target]
p <- plot_ly(x=seq_along(negLogPval), y=~negLogPval,
type="bar", color=~negLogPval, text=term,
colors=c("#4b61ba", "gray", "#a87963", "red"))
layout(p, title="MeSH-Enrichment Analysis (C)",
   xaxis=list(title="Term"),
   yaxis=list(title="-Log(P-value)"),
   legend=list(name=""))
```

```
## Warning: textfont.color doesn't (yet) support data arrays

## Warning: textfont.color doesn't (yet) support data arrays
```

## MeSH-Enrichment Analysis (D : Drugs)

```
negLogPval <- -log10(out.vecLR[['pattern_4_4']]$Enrich$MeSH_D$Pvalue + 1E-10)
term <- out.vecLR[['pattern_4_4']]$Enrich$MeSH_D$Term
target <- seq_len(min(100, length(negLogPval)))
negLogPval <- negLogPval[target]
term <- term[target]
p <- plot_ly(x=seq_along(negLogPval), y=~negLogPval,
type="bar", color=~negLogPval, text=term,
colors=c("#4b61ba", "gray", "#a87963", "red"))
layout(p, title="MeSH-Enrichment Analysis (D)",
   xaxis=list(title="Term"),
   yaxis=list(title="-Log(P-value)"),
   legend=list(name=""))
```

```
## Warning: textfont.color doesn't (yet) support data arrays

## Warning: textfont.color doesn't (yet) support data arrays
```

## MeSH-Enrichment Analysis (E : Analytical, Diagnostic and Therapeutic Techniques and Equipment)

```
negLogPval <- -log10(out.vecLR[['pattern_4_4']]$Enrich$MeSH_E$Pvalue + 1E-10)
term <- out.vecLR[['pattern_4_4']]$Enrich$MeSH_E$Term
target <- seq_len(min(100, length(negLogPval)))
negLogPval <- negLogPval[target]
term <- term[target]
p <- plot_ly(x=seq_along(negLogPval), y=~negLogPval,
type="bar", color=~negLogPval, text=term,
colors=c("#4b61ba", "gray", "#a87963", "red"))
layout(p, title="MeSH-Enrichment Analysis (E)",
   xaxis=list(title="Term"),
   yaxis=list(title="-Log(P-value)"),
   legend=list(name=""))
```

```
## Warning: textfont.color doesn't (yet) support data arrays

## Warning: textfont.color doesn't (yet) support data arrays
```

## MeSH-Enrichment Analysis (F : Psychiatry and Psychology)

```
negLogPval <- -log10(out.vecLR[['pattern_4_4']]$Enrich$MeSH_F$Pvalue + 1E-10)
term <- out.vecLR[['pattern_4_4']]$Enrich$MeSH_F$Term
target <- seq_len(min(100, length(negLogPval)))
negLogPval <- negLogPval[target]
term <- term[target]
p <- plot_ly(x=seq_along(negLogPval), y=~negLogPval,
type="bar", color=~negLogPval, text=term,
colors=c("#4b61ba", "gray", "#a87963", "red"))
layout(p, title="MeSH-Enrichment Analysis (F)",
   xaxis=list(title="Term"),
   yaxis=list(title="-Log(P-value)"),
   legend=list(name=""))
```

```
## Warning: textfont.color doesn't (yet) support data arrays

## Warning: textfont.color doesn't (yet) support data arrays
```

## MeSH-Enrichment Analysis (G : Phenomena and Processes)

```
negLogPval <- -log10(out.vecLR[['pattern_4_4']]$Enrich$MeSH_G$Pvalue + 1E-10)
term <- out.vecLR[['pattern_4_4']]$Enrich$MeSH_G$Term
target <- seq_len(min(100, length(negLogPval)))
negLogPval <- negLogPval[target]
term <- term[target]
p <- plot_ly(x=seq_along(negLogPval), y=~negLogPval,
type="bar", color=~negLogPval, text=term,
colors=c("#4b61ba", "gray", "#a87963", "red"))
layout(p, title="MeSH-Enrichment Analysis (G)",
   xaxis=list(title="Term"),
   yaxis=list(title="-Log(P-value)"),
   legend=list(name=""))
```

```
## Warning: textfont.color doesn't (yet) support data arrays

## Warning: textfont.color doesn't (yet) support data arrays
```

## MeSH-Enrichment Analysis (I : Anthropology, Education, Sociology and Social Phenomena)

```
negLogPval <- -log10(out.vecLR[['pattern_4_4']]$Enrich$MeSH_I$Pvalue + 1E-10)
term <- out.vecLR[['pattern_4_4']]$Enrich$MeSH_I$Term
target <- seq_len(min(100, length(negLogPval)))
negLogPval <- negLogPval[target]
term <- term[target]
p <- plot_ly(x=seq_along(negLogPval), y=~negLogPval,
type="bar", color=~negLogPval, text=term,
colors=c("#4b61ba", "gray", "#a87963", "red"))
layout(p, title="MeSH-Enrichment Analysis (I)",
   xaxis=list(title="Term"),
   yaxis=list(title="-Log(P-value)"),
   legend=list(name=""))
```

```
## Warning: textfont.color doesn't (yet) support data arrays

## Warning: textfont.color doesn't (yet) support data arrays
```

## MeSH-Enrichment Analysis (J : Technology and Food and Beverages)

```
negLogPval <- -log10(out.vecLR[['pattern_4_4']]$Enrich$MeSH_J$Pvalue + 1E-10)
term <- out.vecLR[['pattern_4_4']]$Enrich$MeSH_J$Term
target <- seq_len(min(100, length(negLogPval)))
negLogPval <- negLogPval[target]
term <- term[target]
p <- plot_ly(x=seq_along(negLogPval), y=~negLogPval,
type="bar", color=~negLogPval, text=term,
colors=c("#4b61ba", "gray", "#a87963", "red"))
layout(p, title="MeSH-Enrichment Analysis (J)",
   xaxis=list(title="Term"),
   yaxis=list(title="-Log(P-value)"),
   legend=list(name=""))
```

```
## Warning: textfont.color doesn't (yet) support data arrays

## Warning: textfont.color doesn't (yet) support data arrays
```

## MeSH-Enrichment Analysis (K : Humanities)

```
negLogPval <- -log10(out.vecLR[['pattern_4_4']]$Enrich$MeSH_K$Pvalue + 1E-10)
term <- out.vecLR[['pattern_4_4']]$Enrich$MeSH_K$Term
target <- seq_len(min(100, length(negLogPval)))
negLogPval <- negLogPval[target]
term <- term[target]
p <- plot_ly(x=seq_along(negLogPval), y=~negLogPval,
type="bar", color=~negLogPval, text=term,
colors=c("#4b61ba", "gray", "#a87963", "red"))
layout(p, title="MeSH-Enrichment Analysis (K)",
   xaxis=list(title="Term"),
   yaxis=list(title="-Log(P-value)"),
   legend=list(name=""))
```

## MeSH-Enrichment Analysis (L : Information Science)

```
negLogPval <- -log10(out.vecLR[['pattern_4_4']]$Enrich$MeSH_L$Pvalue + 1E-10)
term <- out.vecLR[['pattern_4_4']]$Enrich$MeSH_L$Term
target <- seq_len(min(100, length(negLogPval)))
negLogPval <- negLogPval[target]
term <- term[target]
p <- plot_ly(x=seq_along(negLogPval), y=~negLogPval,
type="bar", color=~negLogPval, text=term,
colors=c("#4b61ba", "gray", "#a87963", "red"))
layout(p, title="MeSH-Enrichment Analysis (L)",
   xaxis=list(title="Term"),
   yaxis=list(title="-Log(P-value)"),
   legend=list(name=""))
```

```
## Warning: textfont.color doesn't (yet) support data arrays

## Warning: textfont.color doesn't (yet) support data arrays
```

## MeSH-Enrichment Analysis (M : Persons)

```
negLogPval <- -log10(out.vecLR[['pattern_4_4']]$Enrich$MeSH_M$Pvalue + 1E-10)
term <- out.vecLR[['pattern_4_4']]$Enrich$MeSH_M$Term
target <- seq_len(min(100, length(negLogPval)))
negLogPval <- negLogPval[target]
term <- term[target]
p <- plot_ly(x=seq_along(negLogPval), y=~negLogPval,
type="bar", color=~negLogPval, text=term,
colors=c("#4b61ba", "gray", "#a87963", "red"))
layout(p, title="MeSH-Enrichment Analysis (M)",
   xaxis=list(title="Term"),
   yaxis=list(title="-Log(P-value)"),
   legend=list(name=""))
```

```
## Warning: textfont.color doesn't (yet) support data arrays

## Warning: textfont.color doesn't (yet) support data arrays
```

## MeSH-Enrichment Analysis (N : Health Care)

```
negLogPval <- -log10(out.vecLR[['pattern_4_4']]$Enrich$MeSH_N$Pvalue + 1E-10)
term <- out.vecLR[['pattern_4_4']]$Enrich$MeSH_N$Term
target <- seq_len(min(100, length(negLogPval)))
negLogPval <- negLogPval[target]
term <- term[target]
p <- plot_ly(x=seq_along(negLogPval), y=~negLogPval,
type="bar", color=~negLogPval, text=term,
colors=c("#4b61ba", "gray", "#a87963", "red"))
layout(p, title="MeSH-Enrichment Analysis (N)",
   xaxis=list(title="Term"),
   yaxis=list(title="-Log(P-value)"),
   legend=list(name=""))
```

```
## Warning: textfont.color doesn't (yet) support data arrays

## Warning: textfont.color doesn't (yet) support data arrays
```

## MeSH-Enrichment Analysis (Z : Geographical Locations)

```
negLogPval <- -log10(out.vecLR[['pattern_4_4']]$Enrich$MeSH_Z$Pvalue + 1E-10)
term <- out.vecLR[['pattern_4_4']]$Enrich$MeSH_Z$Term
target <- seq_len(min(100, length(negLogPval)))
negLogPval <- negLogPval[target]
term <- term[target]
p <- plot_ly(x=seq_along(negLogPval), y=~negLogPval,
type="bar", color=~negLogPval, text=term,
colors=c("#4b61ba", "gray", "#a87963", "red"))
layout(p, title="MeSH-Enrichment Analysis (Z)",
   xaxis=list(title="Term"),
   yaxis=list(title="-Log(P-value)"),
   legend=list(name=""))
```

```
## Warning: textfont.color doesn't (yet) support data arrays

## Warning: textfont.color doesn't (yet) support data arrays
```

## Reactome Pathway-Enrichment Analysis

```
negLogPval <- -log10(out.vecLR[['pattern_4_4']]$Enrich$Reactome$Pvalue + 1E-10)
term <- out.vecLR[['pattern_4_4']]$Enrich$Reactome$Term
target <- seq_len(min(100, length(negLogPval)))
negLogPval <- negLogPval[target]
term <- term[target]
p <- plot_ly(x=seq_along(negLogPval), y=~negLogPval,
type="bar", color=~negLogPval, text=term,
colors=c("#4b61ba", "gray", "#a87963", "red"))
layout(p, title="Reactome-Enrichment Analysis",
   xaxis=list(title="Term"),
   yaxis=list(title="-Log(P-value)"),
   legend=list(name=""))
```

```
## Warning: textfont.color doesn't (yet) support data arrays

## Warning: textfont.color doesn't (yet) support data arrays
```

## DO-Enrichment Analysis

```
negLogPval <- -log10(out.vecLR[['pattern_4_4']]$Enrich$DO$Pvalue + 1E-10)
term <- out.vecLR[['pattern_4_4']]$Enrich$DO$Term
target <- seq_len(min(100, length(negLogPval)))
negLogPval <- negLogPval[target]
term <- term[target]
p <- plot_ly(x=seq_along(negLogPval), y=~negLogPval,
type="bar", color=~negLogPval, text=term,
colors=c("#4b61ba", "gray", "#a87963", "red"))
layout(p, title="DO-Enrichment Analysis",
   xaxis=list(title="Term"),
   yaxis=list(title="-Log(P-value)"),
   legend=list(name=""))
```

```
## Warning: textfont.color doesn't (yet) support data arrays

## Warning: textfont.color doesn't (yet) support data arrays
```

## NCG-Enrichment Analysis

```
negLogPval <- -log10(out.vecLR[['pattern_4_4']]$Enrich$NCG$Pvalue + 1E-10)
term <- out.vecLR[['pattern_4_4']]$Enrich$NCG$Term
target <- seq_len(min(100, length(negLogPval)))
negLogPval <- negLogPval[target]
term <- term[target]
p <- plot_ly(x=seq_along(negLogPval), y=~negLogPval,
type="bar", color=~negLogPval, text=term,
colors=c("#4b61ba", "gray", "#a87963", "red"))
layout(p, title="NCG-Enrichment Analysis",
   xaxis=list(title="Term"),
   yaxis=list(title="-Log(P-value)"),
   legend=list(name=""))
```

## DGN-Enrichment Analysis

```
negLogPval <- -log10(out.vecLR[['pattern_4_4']]$Enrich$DGN$Pvalue + 1E-10)
term <- out.vecLR[['pattern_4_4']]$Enrich$DGN$Term
target <- seq_len(min(100, length(negLogPval)))
negLogPval <- negLogPval[target]
term <- term[target]
p <- plot_ly(x=seq_along(negLogPval), y=~negLogPval,
type="bar", color=~negLogPval, text=term,
colors=c("#4b61ba", "gray", "#a87963", "red"))
layout(p, title="DGN-Enrichment Analysis",
   xaxis=list(title="Term"),
   yaxis=list(title="-Log(P-value)"),
   legend=list(name=""))
```

```
## Warning: textfont.color doesn't (yet) support data arrays

## Warning: textfont.color doesn't (yet) support data arrays
```
