# Supplementary material for: Sctensor detects many-to-many cell–cell interactions from single cell RNA-sequencing data
Source: BMC Bioinformatics. 2023 Nov 7;24:420. doi: 10.1186/s12859-023-05490-y (PMC10631077; doi:10.1186/s12859-023-05490-y)
Supplement: Supplementary file 16 — Additional file 16. HTML report of HeadandNeckCancer. [file 12859_2023_5490_MOESM16_ESM.zip › output/report/Human_HeadandNeckCancer/pattern_2_1.html]

Details of (2,1,) -related L-R pairs


# Details of (2,1,) -related L-R pairs

# (2,1,:) Pattern

# (2,1,) -related L-R pairs (Top1 pairs)

```
library("scTensor")
load("reanalysis.RData")
col <- .setColor("many")[3]
scTensor:::.myvisNetwork(g, col)
```

| Rank | Ligand Gene | Receptor Gene | Ligand Expression (Log10(exp + 1)) | Receptor Expression (Log10(exp + 1)) | LR-pair factor value (Percentage) | P-value (Grubbs test) | Q-value (BH method) | L-R Evidence cf. rikenbit/lrbase-workflow | PubMed |
| --- | --- | --- | --- | --- | --- | --- | --- | --- | --- |
| 1 | ADM Description: ADMadrenomedullin GO: 1-47 Reactome: 1-13 UniProtKB: 1 RefEx: ADM Expression Atlas: ADM Single Cell Expression Atlas: ADM scRNASeqDB: ADM PanglaoDB: ADM CMap: ADM MeSH: 1-99 100-198 199-297 298-396 397-495 496-594 595-693 694-792 793-891 892-990 991-1089 1090-1188 1189-1287 1288-1386 1387-1485 1486-1584 1585-1682 | RAMP1 Description: ADMreceptor activity modifying protein 1 GO: 1-26 Reactome: 1-13 UniProtKB: 1-2 RefEx: ADM Expression Atlas: RAMP1 Single Cell Expression Atlas: RAMP1 scRNASeqDB: RAMP1 PanglaoDB: RAMP1 CMap: RAMP1 MeSH: 1-100 101-200 201-300 301-399 |  |  | 1.095 (66.155%) | 0.003 | 0.005 | DLRP | 1 |

# (2,1,) -related Enrichment Analysis

```
load("reanalysis.RData")
library("plotly")
```

## MeSH-Enrichment Analysis (A : Anatomy)

```
negLogPval <- -log10(out.vecLR[['pattern_2_1']]$Enrich$MeSH_A$Pvalue + 1E-10)
term <- out.vecLR[['pattern_2_1']]$Enrich$MeSH_A$Term
target <- seq_len(min(100, length(negLogPval)))
negLogPval <- negLogPval[target]
term <- term[target]
p <- plot_ly(x=seq_along(negLogPval), y=~negLogPval,
type="bar", color=~negLogPval, text=term,
colors=c("#4b61ba", "gray", "#a87963", "red"))
layout(p, title="MeSH-Enrichment Analysis (A)",
   xaxis=list(title="Term"),
   yaxis=list(title="-Log(P-value)"),
   legend=list(name=""))
```

```
## Warning: textfont.color doesn't (yet) support data arrays

## Warning: textfont.color doesn't (yet) support data arrays
```

## MeSH-Enrichment Analysis (B : Organisms)

```
negLogPval <- -log10(out.vecLR[['pattern_2_1']]$Enrich$MeSH_B$Pvalue + 1E-10)
term <- out.vecLR[['pattern_2_1']]$Enrich$MeSH_B$Term
target <- seq_len(min(100, length(negLogPval)))
negLogPval <- negLogPval[target]
term <- term[target]
p <- plot_ly(x=seq_along(negLogPval), y=~negLogPval,
type="bar", color=~negLogPval, text=term,
colors=c("#4b61ba", "gray", "#a87963", "red"))
layout(p, title="MeSH-Enrichment Analysis (B)",
   xaxis=list(title="Term"),
   yaxis=list(title="-Log(P-value)"),
   legend=list(name=""))
```

```
## Warning: textfont.color doesn't (yet) support data arrays

## Warning: textfont.color doesn't (yet) support data arrays
```

## MeSH-Enrichment Analysis (C : Diseases)

```
negLogPval <- -log10(out.vecLR[['pattern_2_1']]$Enrich$MeSH_C$Pvalue + 1E-10)
term <- out.vecLR[['pattern_2_1']]$Enrich$MeSH_C$Term
target <- seq_len(min(100, length(negLogPval)))
negLogPval <- negLogPval[target]
term <- term[target]
p <- plot_ly(x=seq_along(negLogPval), y=~negLogPval,
type="bar", color=~negLogPval, text=term,
colors=c("#4b61ba", "gray", "#a87963", "red"))
layout(p, title="MeSH-Enrichment Analysis (C)",
   xaxis=list(title="Term"),
   yaxis=list(title="-Log(P-value)"),
   legend=list(name=""))
```

```
## Warning: textfont.color doesn't (yet) support data arrays

## Warning: textfont.color doesn't (yet) support data arrays
```

## MeSH-Enrichment Analysis (D : Drugs)

```
negLogPval <- -log10(out.vecLR[['pattern_2_1']]$Enrich$MeSH_D$Pvalue + 1E-10)
term <- out.vecLR[['pattern_2_1']]$Enrich$MeSH_D$Term
target <- seq_len(min(100, length(negLogPval)))
negLogPval <- negLogPval[target]
term <- term[target]
p <- plot_ly(x=seq_along(negLogPval), y=~negLogPval,
type="bar", color=~negLogPval, text=term,
colors=c("#4b61ba", "gray", "#a87963", "red"))
layout(p, title="MeSH-Enrichment Analysis (D)",
   xaxis=list(title="Term"),
   yaxis=list(title="-Log(P-value)"),
   legend=list(name=""))
```

```
## Warning: textfont.color doesn't (yet) support data arrays

## Warning: textfont.color doesn't (yet) support data arrays
```

## MeSH-Enrichment Analysis (E : Analytical, Diagnostic and Therapeutic Techniques and Equipment)

```
negLogPval <- -log10(out.vecLR[['pattern_2_1']]$Enrich$MeSH_E$Pvalue + 1E-10)
term <- out.vecLR[['pattern_2_1']]$Enrich$MeSH_E$Term
target <- seq_len(min(100, length(negLogPval)))
negLogPval <- negLogPval[target]
term <- term[target]
p <- plot_ly(x=seq_along(negLogPval), y=~negLogPval,
type="bar", color=~negLogPval, text=term,
colors=c("#4b61ba", "gray", "#a87963", "red"))
layout(p, title="MeSH-Enrichment Analysis (E)",
   xaxis=list(title="Term"),
   yaxis=list(title="-Log(P-value)"),
   legend=list(name=""))
```

```
## Warning: textfont.color doesn't (yet) support data arrays

## Warning: textfont.color doesn't (yet) support data arrays
```

## MeSH-Enrichment Analysis (F : Psychiatry and Psychology)

```
negLogPval <- -log10(out.vecLR[['pattern_2_1']]$Enrich$MeSH_F$Pvalue + 1E-10)
term <- out.vecLR[['pattern_2_1']]$Enrich$MeSH_F$Term
target <- seq_len(min(100, length(negLogPval)))
negLogPval <- negLogPval[target]
term <- term[target]
p <- plot_ly(x=seq_along(negLogPval), y=~negLogPval,
type="bar", color=~negLogPval, text=term,
colors=c("#4b61ba", "gray", "#a87963", "red"))
layout(p, title="MeSH-Enrichment Analysis (F)",
   xaxis=list(title="Term"),
   yaxis=list(title="-Log(P-value)"),
   legend=list(name=""))
```

## MeSH-Enrichment Analysis (G : Phenomena and Processes)

```
negLogPval <- -log10(out.vecLR[['pattern_2_1']]$Enrich$MeSH_G$Pvalue + 1E-10)
term <- out.vecLR[['pattern_2_1']]$Enrich$MeSH_G$Term
target <- seq_len(min(100, length(negLogPval)))
negLogPval <- negLogPval[target]
term <- term[target]
p <- plot_ly(x=seq_along(negLogPval), y=~negLogPval,
type="bar", color=~negLogPval, text=term,
colors=c("#4b61ba", "gray", "#a87963", "red"))
layout(p, title="MeSH-Enrichment Analysis (G)",
   xaxis=list(title="Term"),
   yaxis=list(title="-Log(P-value)"),
   legend=list(name=""))
```

```
## Warning: textfont.color doesn't (yet) support data arrays

## Warning: textfont.color doesn't (yet) support data arrays
```

## MeSH-Enrichment Analysis (H : Disciplines and Occupations)

```
negLogPval <- -log10(out.vecLR[['pattern_2_1']]$Enrich$MeSH_H$Pvalue + 1E-10)
term <- out.vecLR[['pattern_2_1']]$Enrich$MeSH_H$Term
target <- seq_len(min(100, length(negLogPval)))
negLogPval <- negLogPval[target]
term <- term[target]
p <- plot_ly(x=seq_along(negLogPval), y=~negLogPval,
type="bar", color=~negLogPval, text=term,
colors=c("#4b61ba", "gray", "#a87963", "red"))
layout(p, title="MeSH-Enrichment Analysis (H)",
   xaxis=list(title="Term"),
   yaxis=list(title="-Log(P-value)"),
   legend=list(name=""))
```

## MeSH-Enrichment Analysis (J : Technology and Food and Beverages)

```
negLogPval <- -log10(out.vecLR[['pattern_2_1']]$Enrich$MeSH_J$Pvalue + 1E-10)
term <- out.vecLR[['pattern_2_1']]$Enrich$MeSH_J$Term
target <- seq_len(min(100, length(negLogPval)))
negLogPval <- negLogPval[target]
term <- term[target]
p <- plot_ly(x=seq_along(negLogPval), y=~negLogPval,
type="bar", color=~negLogPval, text=term,
colors=c("#4b61ba", "gray", "#a87963", "red"))
layout(p, title="MeSH-Enrichment Analysis (J)",
   xaxis=list(title="Term"),
   yaxis=list(title="-Log(P-value)"),
   legend=list(name=""))
```

```
## Warning: textfont.color doesn't (yet) support data arrays

## Warning: textfont.color doesn't (yet) support data arrays
```

## MeSH-Enrichment Analysis (L : Information Science)

```
negLogPval <- -log10(out.vecLR[['pattern_2_1']]$Enrich$MeSH_L$Pvalue + 1E-10)
term <- out.vecLR[['pattern_2_1']]$Enrich$MeSH_L$Term
target <- seq_len(min(100, length(negLogPval)))
negLogPval <- negLogPval[target]
term <- term[target]
p <- plot_ly(x=seq_along(negLogPval), y=~negLogPval,
type="bar", color=~negLogPval, text=term,
colors=c("#4b61ba", "gray", "#a87963", "red"))
layout(p, title="MeSH-Enrichment Analysis (L)",
   xaxis=list(title="Term"),
   yaxis=list(title="-Log(P-value)"),
   legend=list(name=""))
```

## MeSH-Enrichment Analysis (N : Health Care)

```
negLogPval <- -log10(out.vecLR[['pattern_2_1']]$Enrich$MeSH_N$Pvalue + 1E-10)
term <- out.vecLR[['pattern_2_1']]$Enrich$MeSH_N$Term
target <- seq_len(min(100, length(negLogPval)))
negLogPval <- negLogPval[target]
term <- term[target]
p <- plot_ly(x=seq_along(negLogPval), y=~negLogPval,
type="bar", color=~negLogPval, text=term,
colors=c("#4b61ba", "gray", "#a87963", "red"))
layout(p, title="MeSH-Enrichment Analysis (N)",
   xaxis=list(title="Term"),
   yaxis=list(title="-Log(P-value)"),
   legend=list(name=""))
```

```
## Warning: textfont.color doesn't (yet) support data arrays

## Warning: textfont.color doesn't (yet) support data arrays
```

## Reactome Pathway-Enrichment Analysis

```
negLogPval <- -log10(out.vecLR[['pattern_2_1']]$Enrich$Reactome$Pvalue + 1E-10)
term <- out.vecLR[['pattern_2_1']]$Enrich$Reactome$Term
target <- seq_len(min(100, length(negLogPval)))
negLogPval <- negLogPval[target]
term <- term[target]
p <- plot_ly(x=seq_along(negLogPval), y=~negLogPval,
type="bar", color=~negLogPval, text=term,
colors=c("#4b61ba", "gray", "#a87963", "red"))
layout(p, title="Reactome-Enrichment Analysis",
   xaxis=list(title="Term"),
   yaxis=list(title="-Log(P-value)"),
   legend=list(name=""))
```

```
## Warning: textfont.color doesn't (yet) support data arrays

## Warning: textfont.color doesn't (yet) support data arrays
```

## DO-Enrichment Analysis

```
negLogPval <- -log10(out.vecLR[['pattern_2_1']]$Enrich$DO$Pvalue + 1E-10)
term <- out.vecLR[['pattern_2_1']]$Enrich$DO$Term
target <- seq_len(min(100, length(negLogPval)))
negLogPval <- negLogPval[target]
term <- term[target]
p <- plot_ly(x=seq_along(negLogPval), y=~negLogPval,
type="bar", color=~negLogPval, text=term,
colors=c("#4b61ba", "gray", "#a87963", "red"))
layout(p, title="DO-Enrichment Analysis",
   xaxis=list(title="Term"),
   yaxis=list(title="-Log(P-value)"),
   legend=list(name=""))
```

```
## Warning: textfont.color doesn't (yet) support data arrays

## Warning: textfont.color doesn't (yet) support data arrays
```

## DGN-Enrichment Analysis

```
negLogPval <- -log10(out.vecLR[['pattern_2_1']]$Enrich$DGN$Pvalue + 1E-10)
term <- out.vecLR[['pattern_2_1']]$Enrich$DGN$Term
target <- seq_len(min(100, length(negLogPval)))
negLogPval <- negLogPval[target]
term <- term[target]
p <- plot_ly(x=seq_along(negLogPval), y=~negLogPval,
type="bar", color=~negLogPval, text=term,
colors=c("#4b61ba", "gray", "#a87963", "red"))
layout(p, title="DGN-Enrichment Analysis",
   xaxis=list(title="Term"),
   yaxis=list(title="-Log(P-value)"),
   legend=list(name=""))
```

```
## Warning: textfont.color doesn't (yet) support data arrays

## Warning: textfont.color doesn't (yet) support data arrays
```
