# Supplementary material for: Sctensor detects many-to-many cell–cell interactions from single cell RNA-sequencing data
Source: BMC Bioinformatics. 2023 Nov 7;24:420. doi: 10.1186/s12859-023-05490-y (PMC10631077; doi:10.1186/s12859-023-05490-y)
Supplement: Supplementary file 16 — Additional file 16. HTML report of HeadandNeckCancer. [file 12859_2023_5490_MOESM16_ESM.zip › output/report/Human_HeadandNeckCancer/pattern_1_4.html]

Details of (1,4,) -related L-R pairs


# Details of (1,4,) -related L-R pairs

# (1,4,:) Pattern

# (1,4,) -related L-R pairs (Top3 pairs)

```
library("scTensor")
load("reanalysis.RData")
col <- .setColor("many")[11]
scTensor:::.myvisNetwork(g, col)
```

| Rank | Ligand Gene | Receptor Gene | Ligand Expression (Log10(exp + 1)) | Receptor Expression (Log10(exp + 1)) | LR-pair factor value (Percentage) | P-value (Grubbs test) | Q-value (BH method) | L-R Evidence cf. rikenbit/lrbase-workflow | PubMed |
| --- | --- | --- | --- | --- | --- | --- | --- | --- | --- |
| 1 | IL1RN Description: IL1RNinterleukin 1 receptor antagonist GO: 1-20 Reactome: 1-6 UniProtKB: 1-2 RefEx: IL1RN Expression Atlas: IL1RN Single Cell Expression Atlas: IL1RN scRNASeqDB: IL1RN PanglaoDB: IL1RN CMap: IL1RN MeSH: 1-98 99-196 197-294 295-392 393-490 491-588 589-686 687-784 785-882 883-980 981-1078 1079-1176 1177-1274 1275-1372 1373-1470 1471-1568 1569-1666 1667-1764 1765-1862 1863-1960 1961-2058 2059-2156 2157-2254 2255-2352 2353-2450 2451-2548 2549-2646 2647-2744 2745-2842 2843-2940 2941-3038 3039-3136 3137-3234 3235-3332 3333-3430 3431-3502 | IL2RG Description: IL1RNinterleukin 2 receptor subunit gamma GO: 1-24 Reactome: 1-19 UniProtKB: 1 RefEx: IL1RN Expression Atlas: IL2RG Single Cell Expression Atlas: IL2RG scRNASeqDB: IL2RG PanglaoDB: IL2RG CMap: IL2RG MeSH: 1-99 100-198 199-297 298-396 397-495 496-594 595-693 694-788 |  |  | 0.408 (38.51%) | 0 | 0 | DLRP | 1 |
| 2 | IL7 Description: IL7interleukin 7 GO: 1-25 Reactome: 1-4 UniProtKB: 1-4 RefEx: IL7 Expression Atlas: IL7 Single Cell Expression Atlas: IL7 scRNASeqDB: IL7 PanglaoDB: IL7 CMap: IL7 MeSH: 1-92 93-184 185-276 277-368 369-460 461-552 553-644 645-736 737-828 829-920 921-1012 1013-1104 | IL2RG Description: IL7interleukin 2 receptor subunit gamma GO: 1-24 Reactome: 1-19 UniProtKB: 1 RefEx: IL7 Expression Atlas: IL2RG Single Cell Expression Atlas: IL2RG scRNASeqDB: IL2RG PanglaoDB: IL2RG CMap: IL2RG MeSH: 1-99 100-198 199-297 298-396 397-495 496-594 595-693 694-788 |  |  | 0.313 (29.535%) | 0 | 0 | DLRP | 1 |
| 3 | TNF Description: TNFtumor necrosis factor GO: 1-80 81-160 Reactome: 1-15 UniProtKB: 1-2 STRING: 1 2 3 4 5 6 7 8 RefEx: TNF Expression Atlas: TNF Single Cell Expression Atlas: TNF scRNASeqDB: TNF PanglaoDB: TNF CMap: TNF MeSH: 1-100 101-200 201-300 301-400 401-500 501-600 601-700 701-800 801-900 901-1000 1001-1100 1101-1200 1201-1300 1301-1400 1401-1500 1501-1600 1601-1700 1701-1800 1801-1900 1901-2000 2001-2100 2101-2200 2201-2300 2301-2400 2401-2500 2501-2600 2601-2700 2701-2800 2801-2900 2901-3000 3001-3100 3101-3200 3201-3300 3301-3400 3401-3500 3501-3600 3601-3700 3701-3800 3801-3900 3901-4000 4001-4100 4101-4200 4201-4300 4301-4400 4401-4500 4501-4600 4601-4700 4701-4800 4801-4900 4901-5000 5001-5100 5101-5200 5201-5300 5301-5400 5401-5500 5501-5600 5601-5700 5701-5800 5801-5900 5901-6000 6001-6100 6101-6200 6201-6300 6301-6400 6401-6500 6501-6600 6601-6700 6701-6800 6801-6900 6901-7000 7001-7100 7101-7200 7201-7300 7301-7400 7401-7500 7501-7600 7601-7700 7701-7800 7801-7900 7901-8000 8001-8100 8101-8200 8201-8300 8301-8400 8401-8500 8501-8600 8601-8700 8701-8729 | TNFRSF1B Description: TNFTNF receptor superfamily member 1B GO: 1-42 Reactome: 1-9 UniProtKB: 1 RefEx: TNF Expression Atlas: TNFRSF1B Single Cell Expression Atlas: TNFRSF1B scRNASeqDB: TNFRSF1B PanglaoDB: TNFRSF1B CMap: TNFRSF1B MeSH: 1-96 97-192 193-288 289-384 385-480 481-576 577-672 673-768 769-864 865-960 961-1056 1057-1152 1153-1248 1249-1344 1345-1440 1441-1534 |  |  | 0.216 (20.433%) | 0 | 0 | DLRP | 1 |

# (1,4,) -related Enrichment Analysis

```
load("reanalysis.RData")
library("plotly")
```

## MeSH-Enrichment Analysis (A : Anatomy)

```
negLogPval <- -log10(out.vecLR[['pattern_1_4']]$Enrich$MeSH_A$Pvalue + 1E-10)
term <- out.vecLR[['pattern_1_4']]$Enrich$MeSH_A$Term
target <- seq_len(min(100, length(negLogPval)))
negLogPval <- negLogPval[target]
term <- term[target]
p <- plot_ly(x=seq_along(negLogPval), y=~negLogPval,
type="bar", color=~negLogPval, text=term,
colors=c("#4b61ba", "gray", "#a87963", "red"))
layout(p, title="MeSH-Enrichment Analysis (A)",
   xaxis=list(title="Term"),
   yaxis=list(title="-Log(P-value)"),
   legend=list(name=""))
```

```
## Warning: textfont.color doesn't (yet) support data arrays

## Warning: textfont.color doesn't (yet) support data arrays
```

## MeSH-Enrichment Analysis (B : Organisms)

```
negLogPval <- -log10(out.vecLR[['pattern_1_4']]$Enrich$MeSH_B$Pvalue + 1E-10)
term <- out.vecLR[['pattern_1_4']]$Enrich$MeSH_B$Term
target <- seq_len(min(100, length(negLogPval)))
negLogPval <- negLogPval[target]
term <- term[target]
p <- plot_ly(x=seq_along(negLogPval), y=~negLogPval,
type="bar", color=~negLogPval, text=term,
colors=c("#4b61ba", "gray", "#a87963", "red"))
layout(p, title="MeSH-Enrichment Analysis (B)",
   xaxis=list(title="Term"),
   yaxis=list(title="-Log(P-value)"),
   legend=list(name=""))
```

```
## Warning: textfont.color doesn't (yet) support data arrays

## Warning: textfont.color doesn't (yet) support data arrays
```

## MeSH-Enrichment Analysis (C : Diseases)

```
negLogPval <- -log10(out.vecLR[['pattern_1_4']]$Enrich$MeSH_C$Pvalue + 1E-10)
term <- out.vecLR[['pattern_1_4']]$Enrich$MeSH_C$Term
target <- seq_len(min(100, length(negLogPval)))
negLogPval <- negLogPval[target]
term <- term[target]
p <- plot_ly(x=seq_along(negLogPval), y=~negLogPval,
type="bar", color=~negLogPval, text=term,
colors=c("#4b61ba", "gray", "#a87963", "red"))
layout(p, title="MeSH-Enrichment Analysis (C)",
   xaxis=list(title="Term"),
   yaxis=list(title="-Log(P-value)"),
   legend=list(name=""))
```

```
## Warning: textfont.color doesn't (yet) support data arrays

## Warning: textfont.color doesn't (yet) support data arrays
```

## MeSH-Enrichment Analysis (D : Drugs)

```
negLogPval <- -log10(out.vecLR[['pattern_1_4']]$Enrich$MeSH_D$Pvalue + 1E-10)
term <- out.vecLR[['pattern_1_4']]$Enrich$MeSH_D$Term
target <- seq_len(min(100, length(negLogPval)))
negLogPval <- negLogPval[target]
term <- term[target]
p <- plot_ly(x=seq_along(negLogPval), y=~negLogPval,
type="bar", color=~negLogPval, text=term,
colors=c("#4b61ba", "gray", "#a87963", "red"))
layout(p, title="MeSH-Enrichment Analysis (D)",
   xaxis=list(title="Term"),
   yaxis=list(title="-Log(P-value)"),
   legend=list(name=""))
```

```
## Warning: textfont.color doesn't (yet) support data arrays

## Warning: textfont.color doesn't (yet) support data arrays
```

## MeSH-Enrichment Analysis (E : Analytical, Diagnostic and Therapeutic Techniques and Equipment)

```
negLogPval <- -log10(out.vecLR[['pattern_1_4']]$Enrich$MeSH_E$Pvalue + 1E-10)
term <- out.vecLR[['pattern_1_4']]$Enrich$MeSH_E$Term
target <- seq_len(min(100, length(negLogPval)))
negLogPval <- negLogPval[target]
term <- term[target]
p <- plot_ly(x=seq_along(negLogPval), y=~negLogPval,
type="bar", color=~negLogPval, text=term,
colors=c("#4b61ba", "gray", "#a87963", "red"))
layout(p, title="MeSH-Enrichment Analysis (E)",
   xaxis=list(title="Term"),
   yaxis=list(title="-Log(P-value)"),
   legend=list(name=""))
```

```
## Warning: textfont.color doesn't (yet) support data arrays

## Warning: textfont.color doesn't (yet) support data arrays
```

## MeSH-Enrichment Analysis (F : Psychiatry and Psychology)

```
negLogPval <- -log10(out.vecLR[['pattern_1_4']]$Enrich$MeSH_F$Pvalue + 1E-10)
term <- out.vecLR[['pattern_1_4']]$Enrich$MeSH_F$Term
target <- seq_len(min(100, length(negLogPval)))
negLogPval <- negLogPval[target]
term <- term[target]
p <- plot_ly(x=seq_along(negLogPval), y=~negLogPval,
type="bar", color=~negLogPval, text=term,
colors=c("#4b61ba", "gray", "#a87963", "red"))
layout(p, title="MeSH-Enrichment Analysis (F)",
   xaxis=list(title="Term"),
   yaxis=list(title="-Log(P-value)"),
   legend=list(name=""))
```

```
## Warning: textfont.color doesn't (yet) support data arrays

## Warning: textfont.color doesn't (yet) support data arrays
```

## MeSH-Enrichment Analysis (G : Phenomena and Processes)

```
negLogPval <- -log10(out.vecLR[['pattern_1_4']]$Enrich$MeSH_G$Pvalue + 1E-10)
term <- out.vecLR[['pattern_1_4']]$Enrich$MeSH_G$Term
target <- seq_len(min(100, length(negLogPval)))
negLogPval <- negLogPval[target]
term <- term[target]
p <- plot_ly(x=seq_along(negLogPval), y=~negLogPval,
type="bar", color=~negLogPval, text=term,
colors=c("#4b61ba", "gray", "#a87963", "red"))
layout(p, title="MeSH-Enrichment Analysis (G)",
   xaxis=list(title="Term"),
   yaxis=list(title="-Log(P-value)"),
   legend=list(name=""))
```

```
## Warning: textfont.color doesn't (yet) support data arrays

## Warning: textfont.color doesn't (yet) support data arrays
```

## MeSH-Enrichment Analysis (H : Disciplines and Occupations)

```
negLogPval <- -log10(out.vecLR[['pattern_1_4']]$Enrich$MeSH_H$Pvalue + 1E-10)
term <- out.vecLR[['pattern_1_4']]$Enrich$MeSH_H$Term
target <- seq_len(min(100, length(negLogPval)))
negLogPval <- negLogPval[target]
term <- term[target]
p <- plot_ly(x=seq_along(negLogPval), y=~negLogPval,
type="bar", color=~negLogPval, text=term,
colors=c("#4b61ba", "gray", "#a87963", "red"))
layout(p, title="MeSH-Enrichment Analysis (H)",
   xaxis=list(title="Term"),
   yaxis=list(title="-Log(P-value)"),
   legend=list(name=""))
```

```
## Warning: textfont.color doesn't (yet) support data arrays

## Warning: textfont.color doesn't (yet) support data arrays
```

## MeSH-Enrichment Analysis (I : Anthropology, Education, Sociology and Social Phenomena)

```
negLogPval <- -log10(out.vecLR[['pattern_1_4']]$Enrich$MeSH_I$Pvalue + 1E-10)
term <- out.vecLR[['pattern_1_4']]$Enrich$MeSH_I$Term
target <- seq_len(min(100, length(negLogPval)))
negLogPval <- negLogPval[target]
term <- term[target]
p <- plot_ly(x=seq_along(negLogPval), y=~negLogPval,
type="bar", color=~negLogPval, text=term,
colors=c("#4b61ba", "gray", "#a87963", "red"))
layout(p, title="MeSH-Enrichment Analysis (I)",
   xaxis=list(title="Term"),
   yaxis=list(title="-Log(P-value)"),
   legend=list(name=""))
```

```
## Warning: textfont.color doesn't (yet) support data arrays

## Warning: textfont.color doesn't (yet) support data arrays
```

## MeSH-Enrichment Analysis (J : Technology and Food and Beverages)

```
negLogPval <- -log10(out.vecLR[['pattern_1_4']]$Enrich$MeSH_J$Pvalue + 1E-10)
term <- out.vecLR[['pattern_1_4']]$Enrich$MeSH_J$Term
target <- seq_len(min(100, length(negLogPval)))
negLogPval <- negLogPval[target]
term <- term[target]
p <- plot_ly(x=seq_along(negLogPval), y=~negLogPval,
type="bar", color=~negLogPval, text=term,
colors=c("#4b61ba", "gray", "#a87963", "red"))
layout(p, title="MeSH-Enrichment Analysis (J)",
   xaxis=list(title="Term"),
   yaxis=list(title="-Log(P-value)"),
   legend=list(name=""))
```

```
## Warning: textfont.color doesn't (yet) support data arrays

## Warning: textfont.color doesn't (yet) support data arrays
```

## MeSH-Enrichment Analysis (L : Information Science)

```
negLogPval <- -log10(out.vecLR[['pattern_1_4']]$Enrich$MeSH_L$Pvalue + 1E-10)
term <- out.vecLR[['pattern_1_4']]$Enrich$MeSH_L$Term
target <- seq_len(min(100, length(negLogPval)))
negLogPval <- negLogPval[target]
term <- term[target]
p <- plot_ly(x=seq_along(negLogPval), y=~negLogPval,
type="bar", color=~negLogPval, text=term,
colors=c("#4b61ba", "gray", "#a87963", "red"))
layout(p, title="MeSH-Enrichment Analysis (L)",
   xaxis=list(title="Term"),
   yaxis=list(title="-Log(P-value)"),
   legend=list(name=""))
```

```
## Warning: textfont.color doesn't (yet) support data arrays

## Warning: textfont.color doesn't (yet) support data arrays
```

## MeSH-Enrichment Analysis (M : Persons)

```
negLogPval <- -log10(out.vecLR[['pattern_1_4']]$Enrich$MeSH_M$Pvalue + 1E-10)
term <- out.vecLR[['pattern_1_4']]$Enrich$MeSH_M$Term
target <- seq_len(min(100, length(negLogPval)))
negLogPval <- negLogPval[target]
term <- term[target]
p <- plot_ly(x=seq_along(negLogPval), y=~negLogPval,
type="bar", color=~negLogPval, text=term,
colors=c("#4b61ba", "gray", "#a87963", "red"))
layout(p, title="MeSH-Enrichment Analysis (M)",
   xaxis=list(title="Term"),
   yaxis=list(title="-Log(P-value)"),
   legend=list(name=""))
```

```
## Warning: textfont.color doesn't (yet) support data arrays

## Warning: textfont.color doesn't (yet) support data arrays
```

## MeSH-Enrichment Analysis (N : Health Care)

```
negLogPval <- -log10(out.vecLR[['pattern_1_4']]$Enrich$MeSH_N$Pvalue + 1E-10)
term <- out.vecLR[['pattern_1_4']]$Enrich$MeSH_N$Term
target <- seq_len(min(100, length(negLogPval)))
negLogPval <- negLogPval[target]
term <- term[target]
p <- plot_ly(x=seq_along(negLogPval), y=~negLogPval,
type="bar", color=~negLogPval, text=term,
colors=c("#4b61ba", "gray", "#a87963", "red"))
layout(p, title="MeSH-Enrichment Analysis (N)",
   xaxis=list(title="Term"),
   yaxis=list(title="-Log(P-value)"),
   legend=list(name=""))
```

```
## Warning: textfont.color doesn't (yet) support data arrays

## Warning: textfont.color doesn't (yet) support data arrays
```

## MeSH-Enrichment Analysis (Z : Geographical Locations)

```
negLogPval <- -log10(out.vecLR[['pattern_1_4']]$Enrich$MeSH_Z$Pvalue + 1E-10)
term <- out.vecLR[['pattern_1_4']]$Enrich$MeSH_Z$Term
target <- seq_len(min(100, length(negLogPval)))
negLogPval <- negLogPval[target]
term <- term[target]
p <- plot_ly(x=seq_along(negLogPval), y=~negLogPval,
type="bar", color=~negLogPval, text=term,
colors=c("#4b61ba", "gray", "#a87963", "red"))
layout(p, title="MeSH-Enrichment Analysis (Z)",
   xaxis=list(title="Term"),
   yaxis=list(title="-Log(P-value)"),
   legend=list(name=""))
```

```
## Warning: textfont.color doesn't (yet) support data arrays

## Warning: textfont.color doesn't (yet) support data arrays
```

## Reactome Pathway-Enrichment Analysis

```
negLogPval <- -log10(out.vecLR[['pattern_1_4']]$Enrich$Reactome$Pvalue + 1E-10)
term <- out.vecLR[['pattern_1_4']]$Enrich$Reactome$Term
target <- seq_len(min(100, length(negLogPval)))
negLogPval <- negLogPval[target]
term <- term[target]
p <- plot_ly(x=seq_along(negLogPval), y=~negLogPval,
type="bar", color=~negLogPval, text=term,
colors=c("#4b61ba", "gray", "#a87963", "red"))
layout(p, title="Reactome-Enrichment Analysis",
   xaxis=list(title="Term"),
   yaxis=list(title="-Log(P-value)"),
   legend=list(name=""))
```

```
## Warning: textfont.color doesn't (yet) support data arrays

## Warning: textfont.color doesn't (yet) support data arrays
```

## DO-Enrichment Analysis

```
negLogPval <- -log10(out.vecLR[['pattern_1_4']]$Enrich$DO$Pvalue + 1E-10)
term <- out.vecLR[['pattern_1_4']]$Enrich$DO$Term
target <- seq_len(min(100, length(negLogPval)))
negLogPval <- negLogPval[target]
term <- term[target]
p <- plot_ly(x=seq_along(negLogPval), y=~negLogPval,
type="bar", color=~negLogPval, text=term,
colors=c("#4b61ba", "gray", "#a87963", "red"))
layout(p, title="DO-Enrichment Analysis",
   xaxis=list(title="Term"),
   yaxis=list(title="-Log(P-value)"),
   legend=list(name=""))
```

```
## Warning: textfont.color doesn't (yet) support data arrays

## Warning: textfont.color doesn't (yet) support data arrays
```

## NCG-Enrichment Analysis

```
negLogPval <- -log10(out.vecLR[['pattern_1_4']]$Enrich$NCG$Pvalue + 1E-10)
term <- out.vecLR[['pattern_1_4']]$Enrich$NCG$Term
target <- seq_len(min(100, length(negLogPval)))
negLogPval <- negLogPval[target]
term <- term[target]
p <- plot_ly(x=seq_along(negLogPval), y=~negLogPval,
type="bar", color=~negLogPval, text=term,
colors=c("#4b61ba", "gray", "#a87963", "red"))
layout(p, title="NCG-Enrichment Analysis",
   xaxis=list(title="Term"),
   yaxis=list(title="-Log(P-value)"),
   legend=list(name=""))
```

```
## Warning: textfont.color doesn't (yet) support data arrays

## Warning: textfont.color doesn't (yet) support data arrays
```

## DGN-Enrichment Analysis

```
negLogPval <- -log10(out.vecLR[['pattern_1_4']]$Enrich$DGN$Pvalue + 1E-10)
term <- out.vecLR[['pattern_1_4']]$Enrich$DGN$Term
target <- seq_len(min(100, length(negLogPval)))
negLogPval <- negLogPval[target]
term <- term[target]
p <- plot_ly(x=seq_along(negLogPval), y=~negLogPval,
type="bar", color=~negLogPval, text=term,
colors=c("#4b61ba", "gray", "#a87963", "red"))
layout(p, title="DGN-Enrichment Analysis",
   xaxis=list(title="Term"),
   yaxis=list(title="-Log(P-value)"),
   legend=list(name=""))
```

```
## Warning: textfont.color doesn't (yet) support data arrays

## Warning: textfont.color doesn't (yet) support data arrays
```
