# Supplementary material for: Sctensor detects many-to-many cell–cell interactions from single cell RNA-sequencing data
Source: BMC Bioinformatics. 2023 Nov 7;24:420. doi: 10.1186/s12859-023-05490-y (PMC10631077; doi:10.1186/s12859-023-05490-y)
Supplement: Supplementary file 17 — Additional file 17. HTML report of Uterus. [file 12859_2023_5490_MOESM17_ESM.zip › output/report/Mouse_Uterus/ligand.html]

Details of Ligand Gene-centric Overview (selected)


# Details of Ligand Gene-centric Overview (selected)

```
library("scTensor")
load("reanalysis.RData")
scTensor:::.myvisNetwork(g)
```

| Rank | No. of related pairs | Ligand Gene | Receptor Genes | Related CCIs |
| --- | --- | --- | --- | --- |
| 1 | 19 | Fn1 | Itga4  Itga8 | See the details of (6,4,)-related L-R patterns See the details of (7,3,)-related L-R patterns See the details of (7,6,)-related L-R patterns See the details of (6,1,)-related L-R patterns See the details of (1,6,)-related L-R patterns See the details of (7,1,)-related L-R patterns See the details of (4,3,)-related L-R patterns See the details of (2,3,)-related L-R patterns See the details of (1,4,)-related L-R patterns See the details of (7,4,)-related L-R patterns See the details of (6,3,)-related L-R patterns See the details of (1,1,)-related L-R patterns See the details of (6,6,)-related L-R patterns See the details of (4,4,)-related L-R patterns See the details of (2,6,)-related L-R patterns See the details of (4,1,)-related L-R patterns See the details of (2,4,)-related L-R patterns |
| 3 | 14 | Ptn | Ptprb  Ptprz1 | See the details of (2,5,)-related L-R patterns See the details of (4,5,)-related L-R patterns See the details of (7,5,)-related L-R patterns See the details of (5,5,)-related L-R patterns See the details of (3,5,)-related L-R patterns See the details of (1,5,)-related L-R patterns See the details of (5,2,)-related L-R patterns See the details of (4,2,)-related L-R patterns See the details of (5,4,)-related L-R patterns See the details of (4,4,)-related L-R patterns See the details of (4,1,)-related L-R patterns See the details of (6,5,)-related L-R patterns See the details of (2,4,)-related L-R patterns |
| 3 | 14 | Vegfa | Kdr  Flt1  Nrp1 | See the details of (2,5,)-related L-R patterns See the details of (7,5,)-related L-R patterns See the details of (7,2,)-related L-R patterns See the details of (7,1,)-related L-R patterns See the details of (2,3,)-related L-R patterns See the details of (2,1,)-related L-R patterns See the details of (2,2,)-related L-R patterns See the details of (2,6,)-related L-R patterns See the details of (4,1,)-related L-R patterns See the details of (2,4,)-related L-R patterns |
| 3 | 14 | Igf1 | Igf1r | See the details of (3,5,)-related L-R patterns See the details of (3,2,)-related L-R patterns See the details of (7,6,)-related L-R patterns See the details of (5,2,)-related L-R patterns See the details of (1,6,)-related L-R patterns See the details of (3,6,)-related L-R patterns See the details of (1,4,)-related L-R patterns See the details of (4,2,)-related L-R patterns See the details of (3,4,)-related L-R patterns See the details of (6,6,)-related L-R patterns See the details of (3,3,)-related L-R patterns See the details of (5,6,)-related L-R patterns See the details of (5,4,)-related L-R patterns See the details of (4,4,)-related L-R patterns |
| 5 | 10 | Col4a1 | Itga1 | See the details of (7,6,)-related L-R patterns See the details of (5,3,)-related L-R patterns See the details of (1,3,)-related L-R patterns See the details of (1,5,)-related L-R patterns See the details of (1,6,)-related L-R patterns See the details of (4,3,)-related L-R patterns See the details of (1,4,)-related L-R patterns See the details of (5,6,)-related L-R patterns See the details of (5,4,)-related L-R patterns See the details of (4,4,)-related L-R patterns |
| 6 | 9 | Pdgfa | Pdgfra | See the details of (3,2,)-related L-R patterns See the details of (7,6,)-related L-R patterns See the details of (1,6,)-related L-R patterns See the details of (7,1,)-related L-R patterns See the details of (3,6,)-related L-R patterns See the details of (3,1,)-related L-R patterns See the details of (1,1,)-related L-R patterns See the details of (1,2,)-related L-R patterns See the details of (2,6,)-related L-R patterns |
| 10 | 7 | Nid1 | Ptprf | See the details of (7,2,)-related L-R patterns See the details of (3,2,)-related L-R patterns See the details of (7,6,)-related L-R patterns See the details of (5,3,)-related L-R patterns See the details of (5,2,)-related L-R patterns See the details of (1,2,)-related L-R patterns See the details of (5,6,)-related L-R patterns |
| 10 | 7 | Pdgfc | Kdr  Pdgfra  Flt4 | See the details of (3,5,)-related L-R patterns See the details of (3,2,)-related L-R patterns See the details of (3,6,)-related L-R patterns See the details of (3,1,)-related L-R patterns See the details of (4,1,)-related L-R patterns |
| 10 | 7 | Pgf | Flt1  Nrp1 | See the details of (3,5,)-related L-R patterns See the details of (1,5,)-related L-R patterns See the details of (3,1,)-related L-R patterns See the details of (1,1,)-related L-R patterns See the details of (3,3,)-related L-R patterns |
| 10 | 7 | Npnt | Itga8 | See the details of (7,6,)-related L-R patterns See the details of (4,6,)-related L-R patterns See the details of (3,6,)-related L-R patterns See the details of (4,3,)-related L-R patterns See the details of (6,6,)-related L-R patterns See the details of (2,6,)-related L-R patterns See the details of (4,1,)-related L-R patterns |
| 10 | 7 | Cxcl12 | Ackr3  Cxcr3 | See the details of (5,3,)-related L-R patterns See the details of (4,3,)-related L-R patterns See the details of (1,4,)-related L-R patterns See the details of (5,4,)-related L-R patterns See the details of (4,4,)-related L-R patterns See the details of (4,1,)-related L-R patterns |
| 10 | 7 | Icam2 | Itgb2  Itgal | See the details of (1,4,)-related L-R patterns See the details of (1,1,)-related L-R patterns See the details of (5,4,)-related L-R patterns See the details of (4,4,)-related L-R patterns See the details of (4,1,)-related L-R patterns |
| 10 | 7 | Cd34 | Sell | See the details of (1,4,)-related L-R patterns See the details of (1,1,)-related L-R patterns See the details of (3,4,)-related L-R patterns See the details of (5,4,)-related L-R patterns See the details of (4,4,)-related L-R patterns See the details of (5,1,)-related L-R patterns See the details of (4,1,)-related L-R patterns |
| 15 | 5 | Wnt5a | Fzd6 | See the details of (7,5,)-related L-R patterns See the details of (7,2,)-related L-R patterns See the details of (3,5,)-related L-R patterns See the details of (3,2,)-related L-R patterns See the details of (5,2,)-related L-R patterns |
| 15 | 5 | Edn3 | Ednrb | See the details of (5,3,)-related L-R patterns See the details of (5,2,)-related L-R patterns See the details of (7,1,)-related L-R patterns See the details of (5,4,)-related L-R patterns See the details of (5,1,)-related L-R patterns |
| 15 | 5 | Cd24a | Selp | See the details of (7,1,)-related L-R patterns See the details of (2,1,)-related L-R patterns See the details of (3,1,)-related L-R patterns See the details of (1,1,)-related L-R patterns See the details of (2,4,)-related L-R patterns |
| 17.5 | 4 | Igf2 | Igf1r | See the details of (7,2,)-related L-R patterns See the details of (7,6,)-related L-R patterns See the details of (1,6,)-related L-R patterns See the details of (1,4,)-related L-R patterns |
| 17.5 | 4 | Inhba | Bambi | See the details of (5,2,)-related L-R patterns See the details of (2,2,)-related L-R patterns See the details of (2,6,)-related L-R patterns See the details of (2,4,)-related L-R patterns |
| 21.5 | 3 | Adam12 | Sdc4 | See the details of (7,2,)-related L-R patterns See the details of (7,6,)-related L-R patterns See the details of (5,2,)-related L-R patterns |
| 21.5 | 3 | Rspo3 | Lgr5 | See the details of (3,2,)-related L-R patterns See the details of (5,2,)-related L-R patterns See the details of (4,2,)-related L-R patterns |
| 21.5 | 3 | Kitl | Kit | See the details of (1,5,)-related L-R patterns See the details of (1,4,)-related L-R patterns See the details of (5,4,)-related L-R patterns |
| 21.5 | 3 | Thbs1 | Cd36 | See the details of (7,1,)-related L-R patterns See the details of (2,1,)-related L-R patterns See the details of (2,4,)-related L-R patterns |
| 21.5 | 3 | C3 | C3ar1  Itgb2 | See the details of (3,1,)-related L-R patterns See the details of (3,4,)-related L-R patterns |
| 21.5 | 3 | Cdh1 | Itgb7 | See the details of (1,4,)-related L-R patterns See the details of (3,4,)-related L-R patterns See the details of (2,4,)-related L-R patterns |
| 28.5 | 2 | Ccl7 | Ccr2  Ccr1 | See the details of (6,4,)-related L-R patterns See the details of (6,1,)-related L-R patterns |
| 28.5 | 2 | Ccl8 | Ccr2  Ccr1 | See the details of (6,4,)-related L-R patterns See the details of (6,1,)-related L-R patterns |
| 28.5 | 2 | Ccl2 | Ccr2  Ccr1 | See the details of (6,4,)-related L-R patterns See the details of (6,1,)-related L-R patterns |
| 28.5 | 2 | Fgf1 | Fgfr2 | See the details of (3,2,)-related L-R patterns See the details of (2,2,)-related L-R patterns |
| 28.5 | 2 | Trf | Tfrc | See the details of (6,1,)-related L-R patterns See the details of (6,2,)-related L-R patterns |
| 28.5 | 2 | Col5a3 | Sdc3 | See the details of (5,2,)-related L-R patterns See the details of (5,1,)-related L-R patterns |
| 28.5 | 2 | Ccl11 | Ccr2  Cxcr3 | See the details of (1,4,)-related L-R patterns |
| 28.5 | 2 | Il18 | Il18r1  Il18rap | See the details of (3,4,)-related L-R patterns |
| 40 | 1 | Ccl12 | Ccr2 | See the details of (6,4,)-related L-R patterns |
| 40 | 1 | Ccl24 | Ccr2 | See the details of (6,4,)-related L-R patterns |
| 40 | 1 | Sema3e | Plxnd1 | See the details of (3,5,)-related L-R patterns |
| 40 | 1 | Fgf9 | Fgfr2 | See the details of (3,2,)-related L-R patterns |
| 40 | 1 | Fgf18 | Fgfr2 | See the details of (3,2,)-related L-R patterns |
| 40 | 1 | Efna5 | Epha4 | See the details of (3,2,)-related L-R patterns |
| 40 | 1 | Dlk1 | Notch3 | See the details of (5,3,)-related L-R patterns |
| 40 | 1 | Angpt2 | Tek | See the details of (1,5,)-related L-R patterns |
| 40 | 1 | Ccl6 | Ccr1 | See the details of (6,1,)-related L-R patterns |
| 40 | 1 | Angpt1 | Tie1 | See the details of (5,2,)-related L-R patterns |
| 40 | 1 | Jag1 | Notch3 | See the details of (2,3,)-related L-R patterns |
| 40 | 1 | Ccl21b | Ccr7 | See the details of (1,4,)-related L-R patterns |
| 40 | 1 | Fgf7 | Fgfr2 | See the details of (4,2,)-related L-R patterns |
| 40 | 1 | Il34 | Csf1r | See the details of (1,1,)-related L-R patterns |
| 40 | 1 | Calcb | Ramp1 | See the details of (4,4,)-related L-R patterns |
