# Supplementary material for: Sctensor detects many-to-many cell–cell interactions from single cell RNA-sequencing data
Source: BMC Bioinformatics. 2023 Nov 7;24:420. doi: 10.1186/s12859-023-05490-y (PMC10631077; doi:10.1186/s12859-023-05490-y)
Supplement: Supplementary file 17 — Additional file 17. HTML report of Uterus. [file 12859_2023_5490_MOESM17_ESM.zip › output/report/Mouse_Uterus/ligand_all.html]

Details of Ligand Gene-centric Overview (all)


# Details of Ligand Gene-centric Overview (all)

| Ligand Gene | Receptor Gene |
| --- | --- |
| 100039053 | Ccr7 Cxcr3 |
| A2m | Lrp1 |
| Adam12 | Itga9 Itgb1 Sdc4 |
| Adam2 | Itga6 |
| Adm | Calcrl Ramp1 Ramp2 Ramp3 |
| Adm2 | Calcrl Ramp1 Ramp2 Ramp3 |
| Agrp | Mc4r |
| Agt | Agtr1b Agtr2 Lrp2 |
| Alox5ap | Alox5 |
| Angpt1 | Tek Tie1 |
| Angpt2 | Tek |
| Angpt4 | Tek |
| Angptl1 | Tek |
| Anxa1 | Fpr1 Fpr2 Fpr-rs7 |
| Apln | Aplnr |
| Apoe | Ldlr Lrp5 Lrp8 Sorl1 Vldlr |
| Avp | Avpr1a |
| Bdnf | Ngfr Ntrk2 |
| Bmp15 | Acvr1 Acvr2b Bmpr1a Bmpr1b Bmpr2 |
| Bmp2 | Acvr1 Acvr2b Bmpr1a Bmpr1b Bmpr2 Acvr2a Acvrl1 |
| Bmp4 | Acvr1 Acvr2b Bmpr1a Bmpr1b Bmpr2 Acvr2a Acvrl1 |
| Bmp6 | Acvr1 Acvr2b Bmpr1a Bmpr1b Bmpr2 Acvr2a Acvrl1 |
| Bmp7 | Acvr1 Acvr2b Bmpr1a Bmpr1b Bmpr2 Acvr2a Acvrl1 |
| C3 | C3ar1 Itgax Itgb2 Lrp1 |
| Calca | Calcrl Ramp1 Ramp2 Ramp3 |
| Calcb | Calcr Calcrl Ramp1 Ramp2 Ramp3 |
| Calm1 | Mylk Pde1c |
| Calm2 | Mylk Pde1c |
| Calm3 | Mylk Pde1c |
| Cck | Cckar Cckbr |
| Ccl11 | Ccr2 Ccr3 Ccr5 Cxcr3 |
| Ccl12 | Ccr2 |
| Ccl19 | Ccr7 Cxcr3 |
| Ccl2 | Ccr2 Ccr3 Ccr5 Ccr1 Ccr4 Cxcr3 |
| Ccl20 | Cxcr3 |
| Ccl21b | Ccr7 |
| Ccl21d | Ccr7 |
| Ccl24 | Ccr2 Ccr3 |
| Ccl3 | Ccr1 Ccr3 Ccr5 |
| Ccl4 | Ccr1 Ccr5 |
| Ccl5 | Cxcr3 Gpr75 |
| Ccl6 | Ccr1 Ccr3 |
| Ccl7 | Ccr1 Ccr2 Ccr3 Ccr5 Cxcr3 |
| Ccl8 | Ccr1 Ccr2 Ccr3 Ccr5 |
| Cd24a | Selp |
| Cd274 | Pdcd1 |
| Cd34 | Sell |
| Cdh1 | Itgae Itgb7 |
| Col18a1 | Gpc1 Itgb1 |
| Col1a1 | Ddr1 Ddr2 Itga11 Itga2 Itgb1 |
| Col4a1 | Itga1 Itgb1 Itgb8 |
| Col5a3 | Sdc3 |
| Cort | Sstr3 |
| Csf1 | Csf1r |
| Csf2 | Csf1r |
| Csf3 | Csf1r |
| Cx3cl1 | Cx3cr1 |
| Cxcl10 | Ccr3 Cxcr3 |
| Cxcl12 | Ackr3 Cxcr3 Cxcr4 |
| Cxcl13 | Cxcr5 |
| Cxcl16 | Cxcr6 |
| Cxcl17 | Gpr35 |
| Cxcl9 | Ccr3 Cxcr3 |
| Dhh | Hhip Ptch2 |
| Dkk2 | Lrp6 |
| Dlk1 | Notch1 Notch2 Notch3 Notch4 |
| Dll1 | Notch3 |
| Dll3 | Notch3 |
| Dll4 | Notch3 |
| Dusp18 | Itga1 Itga6 Itgb4 |
| Eda | Edar |
| Edn1 | Ednra |
| Edn3 | Ednrb |
| Efna1 | Epha1 Epha2 Epha3 Epha4 Epha5 Epha7 Epha8 Epha6 |
| Efna2 | Epha4 Epha7 |
| Efna3 | Epha4 Epha7 Ephb1 |
| Efna4 | Epha4 Epha7 |
| Efna5 | Epha1 Epha2 Epha3 Epha4 Epha5 Epha7 Epha8 Epha6 Ephb2 |
| Efnb1 | Epha4 Ephb1 |
| Efnb2 | Epha4 Ephb1 Ephb2 Ephb3 Ephb4 Ephb6 |
| Efnb3 | Epha4 Ephb1 |
| Enho | Gpr19 |
| F2 | F2rl1 |
| F7 | F3 |
| F8 | Lrp1 |
| Fasl | Fas |
| Fgf1 | Fgfr1 Fgfr2 Fgfr3 Fgfr4 |
| Fgf10 | Fgfr2 Fgfr4 |
| Fgf11 | Fgfr2 Fgfr4 |
| Fgf12 | Fgfr2 Fgfr4 |
| Fgf13 | Fgfr2 Fgfr4 |
| Fgf14 | Fgfr2 Fgfr4 |
| Fgf15 | Fgfr2 Fgfr4 |
| Fgf16 | Fgfr2 Fgfr4 |
| Fgf17 | Fgfr2 Fgfr4 |
| Fgf18 | Fgfr1 Fgfr2 Fgfr3 Fgfr4 |
| Fgf2 | Fgfr2 Fgfr4 Sdc4 |
| Fgf22 | Fgfr2 |
| Fgf23 | Fgfr2 Fgfr4 |
| Fgf3 | Fgfr2 |
| Fgf4 | Fgfr2 Fgfr4 |
| Fgf5 | Fgfr2 Fgfr4 |
| Fgf6 | Fgfr2 Fgfr4 |
| Fgf7 | Fgfr2 |
| Fgf8 | Fgfr2 Fgfr4 |
| Fgf9 | Fgfr1 Fgfr2 Fgfr3 Fgfr4 |
| Fn1 | Itga2 Itga2b Itga4 Itga8 Itgb1 Itgb3 Itgb6 Itgb8 Sdc2 |
| Gas6 | Axl Mertk Tyro3 |
| Gdf10 | Acvr1b Acvr1c Acvr2a Acvr2b Bmpr1a Bmpr1b Bmpr2 Tgfbr1 |
| Gdf15 | Gfral |
| Gdnf | Gfra1 Gfra2 Ret |
| Gh | Prlr |
| Gm11127 | Klrc2 Klrd1 |
| Gm7030 | Klrc2 Klrd1 |
| Grp | Grpr |
| Guca1a | Gucy2e |
| Guca1b | Gucy2e |
| Has2 | Cd44 Hmmr |
| Hbegf | Egfr Erbb4 |
| Hc | C5ar1 |
| Hdc | Hrh1 Hrh2 Hrh3 Hrh4 |
| Iapp | Ramp1 Ramp2 Ramp3 |
| Icam1 | Itgal Itgax Itgb2 |
| Icam2 | Itgal Itgb2 |
| Icam4 | Itgal Itgb2 |
| Icam5 | Itgal Itgb2 |
| Igf1 | Igf1r |
| Igf2 | Igf2r Igf1r Insr |
| Ihh | Hhip |
| Il12a | Il12rb1 |
| Il12b | Il12rb1 |
| Il13 | Il13ra2 Il2rg |
| Il15 | Il15ra Il2rb Il2rg |
| Il16 | Cd4 |
| Il17b | Il17rb |
| Il17c | Il17re |
| Il18 | Il18r1 Il18rap Il1rapl1 |
| Il1a | Il1r2 |
| Il1b | Il1r2 |
| Il1r1 | Il1r2 |
| Il1rn | Il2ra Il2rb Il2rg |
| Il2 | Il2ra Il2rb Il2rg |
| Il21 | Il21r Il2rg |
| Il23a | Il12rb1 |
| Il25 | Il17rb |
| Il33 | Il1rl1 |
| Il34 | Csf1r |
| Il4 | Il2rg |
| Il5 | Csf2rb2 Il5ra Csf2rb |
| Il7 | Il2rg Il7r |
| Il9 | Il2rg Il9r |
| Inhba | Acvr1 Acvr1b Acvr2b Acvr1c Acvr2a Bambi |
| Insl3 | Rxfp1 |
| Jag1 | Notch1 Notch2 Notch3 Notch4 |
| Jag2 | Notch3 |
| Kitl | Kit |
| Lama2 | Itgb1 |
| Lamb1 | Itga6 |
| Lamc2 | Itga3 Itga6 Itgb1 Itgb4 |
| Leap2 | Ghsr |
| Lep | Lepr |
| Lif | Il6st Lifr |
| Liph | Lpar1 Lpar4 |
| Ltb | Ltbr Cd40 |
| Ltf | Lrp1 Lrp11 |
| Madcam1 | Itga4 Itgb7 |
| Mdk | Ptprb Ptprz1 |
| Ncam1 | Robo1 Robo3 |
| Ndp | Fzd4 Lgr4 |
| Ngf | Ngfr |
| Nid1 | Ptprf |
| Nms | Nmur1 |
| Nmu | Nmur1 |
| Npnt | Itga8 Itgb1 |
| Nppa | Npr3 |
| Nppb | Npr3 |
| Nppc | Npr2 |
| Npy | Npy2r Npy4r Prlhr Npy1r |
| Ntf3 | Ngfr Ntrk2 |
| Ntf5 | Ngfr Ntrk2 |
| Nts | Ntsr1 Ntsr2 |
| Osm | Il6st Lifr Osmr |
| Ostn | Npr3 |
| Oxt | Avpr1a |
| Pdgfa | Pdgfra |
| Pdgfb | Pdgfra Pdgfrb |
| Pdgfc | Flt4 Kdr Pdgfra |
| Pdgfd | Pdgfrb |
| Penk | Mrgpra2b Oprd1 Oprm1 Ogfr |
| Pgf | Flt1 Nrp1 |
| Plat | Lrp1 |
| Plau | Plaur |
| Podxl2 | Sell |
| Pomc | Mc4r |
| Prl | Prlr |
| Prl4a1 | Prlr |
| Ptdss1 | Scarb1 |
| Pthlh | Prlhr Pth1r Pth2r |
| Ptn | Ptprb Ptprz1 |
| Pyy | Npy1r |
| Rarres2 | Cmklr1 |
| Reln | Itga3 Itgb1 Lrp8 Vldlr |
| Ren1 | Atp6ap2 |
| Rln1 | Rxfp1 |
| Rln3 | Rxfp1 |
| Rps19 | C5ar1 |
| Rspo1 | Lgr5 |
| Rspo2 | Lgr5 |
| Rspo3 | Lgr4 Lgr5 Lgr6 |
| Rspo4 | Lgr5 |
| Selplg | Selp Sele |
| Sema3a | Nrp1 |
| Sema3b | Nrp1 |
| Sema3e | Plxnd1 |
| Sema5a | Met Plxnb3 |
| Sema6d | Kdr |
| Sema7a | Plxnc1 |
| Serpine2 | Lrp1 |
| Serping1 | Lrp1 |
| Shh | Hhip |
| Slit2 | Robo2 Robo4 |
| Sost | Lrp5 Lrp6 |
| Spp1 | Cd44 Itgav |
| Sst | Sstr3 |
| Tac1 | Mrgpra4 Tacr1 Tacr2 Tacr3 |
| Tgfb1 | Tgfbr1 Tgfbr2 Tgfbr3 Acvrl1 |
| Tgfb2 | Tgfbr1 Tgfbr2 Tgfbr3 |
| Tgfb3 | Tgfbr1 Tgfbr2 Tgfbr3 Acvrl1 |
| Thbs1 | Cd36 Cd47 Sdc1 |
| Tnfsf10 | Tnfrsf26 Tnfrsf22 Tnfrsf11b |
| Tnfsf12 | Tnfrsf12a |
| Tnfsf13b | Tnfrsf13c |
| Tnfsf14 | Ltbr Tnfrsf14 |
| Tnfsf18 | Tnfrsf18 |
| Tnfsf9 | Tnfrsf9 |
| Tph1 | Htr1a Htr1b Htr1f Htr2a Htr2b Htr2c Htr4 Htr5a Htr6 Htr7 |
| Trf | Tfrc |
| Tslp | Il7r |
| Vcam1 | Itga4 Itgad Itgb1 Itgb2 |
| Vegfa | Flt1 Kdr Nrp1 |
| Vegfb | Flt1 Nrp1 |
| Vegfc | Flt4 Kdr |
| Vegfd | Flt4 |
| Vtn | Itgb3 |
| Wnt11 | Fzd7 |
| Wnt3a | Fzd6 |
| Wnt4 | Fzd6 |
| Wnt5a | Fzd6 Fzd2 Fzd4 Fzd7 Ryk |
| Wnt7a | Fzd5 |
| Wnt7b | Fzd10 |
| Xcl1 | Xcr1 |
