# Supplementary material for: Sctensor detects many-to-many cell–cell interactions from single cell RNA-sequencing data
Source: BMC Bioinformatics. 2023 Nov 7;24:420. doi: 10.1186/s12859-023-05490-y (PMC10631077; doi:10.1186/s12859-023-05490-y)
Supplement: Supplementary file 17 — Additional file 17. HTML report of Uterus. [file 12859_2023_5490_MOESM17_ESM.zip › output/report/Mouse_Uterus/receptor.html]

Details of Receptor Gene-centric Overview (selected)


# Details of Receptor Gene-centric Overview (selected)

```
library("scTensor")
load("reanalysis.RData")
scTensor:::.myvisNetwork(g)
```

| Rank | No. of related pairs | Receptor Gene | Ligand Genes | Related CCIs |
| --- | --- | --- | --- | --- |
| 1 | 18 | Igf1r | Igf2  Igf1 | See the details of (7,2,)-related L-R patterns See the details of (3,5,)-related L-R patterns See the details of (3,2,)-related L-R patterns See the details of (7,6,)-related L-R patterns See the details of (5,2,)-related L-R patterns See the details of (1,6,)-related L-R patterns See the details of (3,6,)-related L-R patterns See the details of (1,4,)-related L-R patterns See the details of (4,2,)-related L-R patterns See the details of (3,4,)-related L-R patterns See the details of (6,6,)-related L-R patterns See the details of (3,3,)-related L-R patterns See the details of (5,6,)-related L-R patterns See the details of (5,4,)-related L-R patterns See the details of (4,4,)-related L-R patterns |
| 3.5 | 13 | Ptprb | Ptn | See the details of (2,5,)-related L-R patterns See the details of (4,5,)-related L-R patterns See the details of (7,5,)-related L-R patterns See the details of (5,5,)-related L-R patterns See the details of (3,5,)-related L-R patterns See the details of (1,5,)-related L-R patterns See the details of (5,2,)-related L-R patterns See the details of (4,2,)-related L-R patterns See the details of (5,4,)-related L-R patterns See the details of (4,4,)-related L-R patterns See the details of (4,1,)-related L-R patterns See the details of (6,5,)-related L-R patterns See the details of (2,4,)-related L-R patterns |
| 3.5 | 13 | Itga4 | Fn1 | See the details of (6,4,)-related L-R patterns See the details of (7,3,)-related L-R patterns See the details of (6,1,)-related L-R patterns See the details of (7,1,)-related L-R patterns See the details of (4,3,)-related L-R patterns See the details of (2,3,)-related L-R patterns See the details of (1,4,)-related L-R patterns See the details of (7,4,)-related L-R patterns See the details of (6,3,)-related L-R patterns See the details of (1,1,)-related L-R patterns See the details of (4,4,)-related L-R patterns See the details of (4,1,)-related L-R patterns See the details of (2,4,)-related L-R patterns |
| 3.5 | 13 | Pdgfra | Pdgfa  Pdgfc | See the details of (3,2,)-related L-R patterns See the details of (7,6,)-related L-R patterns See the details of (1,6,)-related L-R patterns See the details of (7,1,)-related L-R patterns See the details of (3,6,)-related L-R patterns See the details of (3,1,)-related L-R patterns See the details of (1,1,)-related L-R patterns See the details of (1,2,)-related L-R patterns See the details of (2,6,)-related L-R patterns See the details of (4,1,)-related L-R patterns |
| 3.5 | 13 | Itga8 | Fn1  Npnt | See the details of (7,6,)-related L-R patterns See the details of (4,6,)-related L-R patterns See the details of (1,6,)-related L-R patterns See the details of (7,1,)-related L-R patterns See the details of (3,6,)-related L-R patterns See the details of (4,3,)-related L-R patterns See the details of (6,6,)-related L-R patterns See the details of (2,6,)-related L-R patterns See the details of (4,1,)-related L-R patterns |
| 6 | 12 | Nrp1 | Vegfa  Pgf | See the details of (2,5,)-related L-R patterns See the details of (7,5,)-related L-R patterns See the details of (3,5,)-related L-R patterns See the details of (1,5,)-related L-R patterns See the details of (7,1,)-related L-R patterns See the details of (2,3,)-related L-R patterns See the details of (2,1,)-related L-R patterns See the details of (3,1,)-related L-R patterns See the details of (1,1,)-related L-R patterns See the details of (3,3,)-related L-R patterns See the details of (2,6,)-related L-R patterns See the details of (4,1,)-related L-R patterns |
| 7 | 10 | Itga1 | Col4a1 | See the details of (7,6,)-related L-R patterns See the details of (5,3,)-related L-R patterns See the details of (1,3,)-related L-R patterns See the details of (1,5,)-related L-R patterns See the details of (1,6,)-related L-R patterns See the details of (4,3,)-related L-R patterns See the details of (1,4,)-related L-R patterns See the details of (5,6,)-related L-R patterns See the details of (5,4,)-related L-R patterns See the details of (4,4,)-related L-R patterns |
| 9.5 | 7 | Kdr | Vegfa  Pdgfc | See the details of (2,5,)-related L-R patterns See the details of (7,5,)-related L-R patterns See the details of (7,2,)-related L-R patterns See the details of (3,5,)-related L-R patterns See the details of (3,2,)-related L-R patterns See the details of (2,2,)-related L-R patterns See the details of (2,4,)-related L-R patterns |
| 9.5 | 7 | Ptprf | Nid1 | See the details of (7,2,)-related L-R patterns See the details of (3,2,)-related L-R patterns See the details of (7,6,)-related L-R patterns See the details of (5,3,)-related L-R patterns See the details of (5,2,)-related L-R patterns See the details of (1,2,)-related L-R patterns See the details of (5,6,)-related L-R patterns |
| 9.5 | 7 | Itgb2 | C3  Icam2 | See the details of (3,1,)-related L-R patterns See the details of (1,4,)-related L-R patterns See the details of (1,1,)-related L-R patterns See the details of (3,4,)-related L-R patterns See the details of (5,4,)-related L-R patterns See the details of (4,4,)-related L-R patterns See the details of (4,1,)-related L-R patterns |
| 9.5 | 7 | Sell | Cd34 | See the details of (1,4,)-related L-R patterns See the details of (1,1,)-related L-R patterns See the details of (3,4,)-related L-R patterns See the details of (5,4,)-related L-R patterns See the details of (4,4,)-related L-R patterns See the details of (5,1,)-related L-R patterns See the details of (4,1,)-related L-R patterns |
| 12 | 6 | Ccr2 | Ccl12  Ccl24  Ccl7  Ccl8  Ccl2  Ccl11 | See the details of (6,4,)-related L-R patterns See the details of (1,4,)-related L-R patterns |
| 15 | 5 | Fzd6 | Wnt5a | See the details of (7,5,)-related L-R patterns See the details of (7,2,)-related L-R patterns See the details of (3,5,)-related L-R patterns See the details of (3,2,)-related L-R patterns See the details of (5,2,)-related L-R patterns |
| 15 | 5 | Fgfr2 | Fgf9  Fgf1  Fgf18  Fgf7 | See the details of (3,2,)-related L-R patterns See the details of (4,2,)-related L-R patterns See the details of (2,2,)-related L-R patterns |
| 15 | 5 | Ednrb | Edn3 | See the details of (5,3,)-related L-R patterns See the details of (5,2,)-related L-R patterns See the details of (7,1,)-related L-R patterns See the details of (5,4,)-related L-R patterns See the details of (5,1,)-related L-R patterns |
| 15 | 5 | Selp | Cd24a | See the details of (7,1,)-related L-R patterns See the details of (2,1,)-related L-R patterns See the details of (3,1,)-related L-R patterns See the details of (1,1,)-related L-R patterns See the details of (2,4,)-related L-R patterns |
| 15 | 5 | Cxcr3 | Cxcl12  Ccl11 | See the details of (1,4,)-related L-R patterns See the details of (5,4,)-related L-R patterns See the details of (4,4,)-related L-R patterns See the details of (4,1,)-related L-R patterns |
| 19 | 4 | Flt1 | Vegfa  Pgf | See the details of (2,5,)-related L-R patterns See the details of (7,5,)-related L-R patterns See the details of (3,5,)-related L-R patterns See the details of (1,5,)-related L-R patterns |
| 19 | 4 | Ccr1 | Ccl6  Ccl7  Ccl8  Ccl2 | See the details of (6,1,)-related L-R patterns |
| 19 | 4 | Bambi | Inhba | See the details of (5,2,)-related L-R patterns See the details of (2,2,)-related L-R patterns See the details of (2,6,)-related L-R patterns See the details of (2,4,)-related L-R patterns |
| 23.5 | 3 | Sdc4 | Adam12 | See the details of (7,2,)-related L-R patterns See the details of (7,6,)-related L-R patterns See the details of (5,2,)-related L-R patterns |
| 23.5 | 3 | Lgr5 | Rspo3 | See the details of (3,2,)-related L-R patterns See the details of (5,2,)-related L-R patterns See the details of (4,2,)-related L-R patterns |
| 23.5 | 3 | Ackr3 | Cxcl12 | See the details of (5,3,)-related L-R patterns See the details of (4,3,)-related L-R patterns See the details of (4,1,)-related L-R patterns |
| 23.5 | 3 | Kit | Kitl | See the details of (1,5,)-related L-R patterns See the details of (1,4,)-related L-R patterns See the details of (5,4,)-related L-R patterns |
| 23.5 | 3 | Cd36 | Thbs1 | See the details of (7,1,)-related L-R patterns See the details of (2,1,)-related L-R patterns See the details of (2,4,)-related L-R patterns |
| 23.5 | 3 | Itgb7 | Cdh1 | See the details of (1,4,)-related L-R patterns See the details of (3,4,)-related L-R patterns See the details of (2,4,)-related L-R patterns |
| 28.5 | 2 | Notch3 | Dlk1  Jag1 | See the details of (5,3,)-related L-R patterns See the details of (2,3,)-related L-R patterns |
| 28.5 | 2 | Tfrc | Trf | See the details of (6,1,)-related L-R patterns See the details of (6,2,)-related L-R patterns |
| 28.5 | 2 | Sdc3 | Col5a3 | See the details of (5,2,)-related L-R patterns See the details of (5,1,)-related L-R patterns |
| 28.5 | 2 | Itgal | Icam2 | See the details of (1,4,)-related L-R patterns See the details of (5,4,)-related L-R patterns |
| 36.5 | 1 | Plxnd1 | Sema3e | See the details of (3,5,)-related L-R patterns |
| 36.5 | 1 | Flt4 | Pdgfc | See the details of (3,2,)-related L-R patterns |
| 36.5 | 1 | Epha4 | Efna5 | See the details of (3,2,)-related L-R patterns |
| 36.5 | 1 | Tek | Angpt2 | See the details of (1,5,)-related L-R patterns |
| 36.5 | 1 | Tie1 | Angpt1 | See the details of (5,2,)-related L-R patterns |
| 36.5 | 1 | C3ar1 | C3 | See the details of (3,1,)-related L-R patterns |
| 36.5 | 1 | Ccr7 | Ccl21b | See the details of (1,4,)-related L-R patterns |
| 36.5 | 1 | Csf1r | Il34 | See the details of (1,1,)-related L-R patterns |
| 36.5 | 1 | Il18r1 | Il18 | See the details of (3,4,)-related L-R patterns |
| 36.5 | 1 | Il18rap | Il18 | See the details of (3,4,)-related L-R patterns |
| 36.5 | 1 | Ramp1 | Calcb | See the details of (4,4,)-related L-R patterns |
| 36.5 | 1 | Ptprz1 | Ptn | See the details of (4,1,)-related L-R patterns |
