# Supplementary material for: Sctensor detects many-to-many cell–cell interactions from single cell RNA-sequencing data
Source: BMC Bioinformatics. 2023 Nov 7;24:420. doi: 10.1186/s12859-023-05490-y (PMC10631077; doi:10.1186/s12859-023-05490-y)
Supplement: Supplementary file 17 — Additional file 17. HTML report of Uterus. [file 12859_2023_5490_MOESM17_ESM.zip › output/report/Mouse_Uterus/receptor_all.html]

Details of Receptor Gene-centric Overview (all)


# Details of Receptor Gene-centric Overview (all)

| Receptor Gene | Ligand Gene |
| --- | --- |
| Ackr3 | Cxcl12 |
| Acvr1 | Bmp15 Bmp2 Bmp4 Bmp6 Bmp7 Inhba |
| Acvr1b | Inhba Gdf10 |
| Acvr1c | Inhba Gdf10 |
| Acvr2a | Bmp2 Bmp4 Bmp6 Bmp7 Inhba Gdf10 |
| Acvr2b | Bmp15 Bmp2 Bmp4 Bmp6 Bmp7 Inhba Gdf10 |
| Acvrl1 | Bmp2 Bmp4 Bmp6 Bmp7 Tgfb1 Tgfb3 |
| Agtr1b | Agt |
| Agtr2 | Agt |
| Alox5 | Alox5ap |
| Aplnr | Apln |
| Atp6ap2 | Ren1 |
| Avpr1a | Avp Oxt |
| Axl | Gas6 |
| Bambi | Inhba |
| Bmpr1a | Bmp15 Bmp2 Bmp4 Bmp6 Bmp7 Gdf10 |
| Bmpr1b | Bmp15 Bmp2 Bmp4 Bmp6 Bmp7 Gdf10 |
| Bmpr2 | Bmp15 Bmp2 Bmp4 Bmp6 Bmp7 Gdf10 |
| C3ar1 | C3 |
| C5ar1 | Hc Rps19 |
| Calcr | Calcb |
| Calcrl | Calcb Calca Adm2 Adm |
| Cckar | Cck |
| Cckbr | Cck |
| Ccr1 | Ccl2 Ccl3 Ccl4 Ccl6 Ccl7 Ccl8 |
| Ccr2 | Ccl11 Ccl12 Ccl2 Ccl24 Ccl7 Ccl8 |
| Ccr3 | Ccl11 Ccl2 Ccl24 Ccl3 Ccl6 Ccl7 Ccl8 Cxcl10 Cxcl9 |
| Ccr4 | Ccl2 |
| Ccr5 | Ccl11 Ccl2 Ccl3 Ccl4 Ccl7 Ccl8 |
| Ccr7 | Ccl21b Ccl21d Ccl19 100039053 |
| Cd36 | Thbs1 |
| Cd4 | Il16 |
| Cd40 | Ltb |
| Cd44 | Has2 Spp1 |
| Cd47 | Thbs1 |
| Cmklr1 | Rarres2 |
| Csf1r | Il34 Csf1 Csf2 Csf3 |
| Csf2rb | Il5 |
| Csf2rb2 | Il5 |
| Cx3cr1 | Cx3cl1 |
| Cxcr3 | Ccl11 Ccl2 Ccl5 Ccl7 Cxcl10 Cxcl12 Cxcl9 Ccl19 Ccl20 100039053 |
| Cxcr4 | Cxcl12 |
| Cxcr5 | Cxcl13 |
| Cxcr6 | Cxcl16 |
| Ddr1 | Col1a1 |
| Ddr2 | Col1a1 |
| Edar | Eda |
| Ednra | Edn1 |
| Ednrb | Edn3 |
| Egfr | Hbegf |
| Epha1 | Efna1 Efna5 |
| Epha2 | Efna1 Efna5 |
| Epha3 | Efna1 Efna5 |
| Epha4 | Efna1 Efna5 Efnb2 Efna4 Efnb3 Efnb1 Efna2 Efna3 |
| Epha5 | Efna1 Efna5 |
| Epha6 | Efna1 Efna5 |
| Epha7 | Efna1 Efna5 Efna2 Efna4 Efna3 |
| Epha8 | Efna1 Efna5 |
| Ephb1 | Efnb2 Efnb3 Efnb1 Efna3 |
| Ephb2 | Efna5 Efnb2 |
| Ephb3 | Efnb2 |
| Ephb4 | Efnb2 |
| Ephb6 | Efnb2 |
| Erbb4 | Hbegf |
| F2rl1 | F2 |
| F3 | F7 |
| Fas | Fasl |
| Fgfr1 | Fgf1 Fgf18 Fgf9 |
| Fgfr2 | Fgf1 Fgf18 Fgf7 Fgf9 Fgf6 Fgf13 Fgf11 Fgf15 Fgf12 Fgf10 Fgf17 Fgf16 Fgf3 Fgf2 Fgf8 Fgf4 Fgf14 Fgf5 Fgf22 Fgf23 |
| Fgfr3 | Fgf1 Fgf18 Fgf9 |
| Fgfr4 | Fgf1 Fgf18 Fgf9 Fgf6 Fgf8 Fgf2 Fgf10 Fgf16 Fgf4 Fgf11 Fgf13 Fgf15 Fgf17 Fgf14 Fgf12 Fgf23 Fgf5 |
| Flt1 | Pgf Vegfa Vegfb |
| Flt4 | Pdgfc Vegfc Vegfd |
| Fpr-rs7 | Anxa1 |
| Fpr1 | Anxa1 |
| Fpr2 | Anxa1 |
| Fzd10 | Wnt7b |
| Fzd2 | Wnt5a |
| Fzd4 | Ndp Wnt5a |
| Fzd5 | Wnt7a |
| Fzd6 | Wnt5a Wnt3a Wnt4 |
| Fzd7 | Wnt11 Wnt5a |
| Gfra1 | Gdnf |
| Gfra2 | Gdnf |
| Gfral | Gdf15 |
| Ghsr | Leap2 |
| Gpc1 | Col18a1 |
| Gpr19 | Enho |
| Gpr35 | Cxcl17 |
| Gpr75 | Ccl5 |
| Grpr | Grp |
| Gucy2e | Guca1a Guca1b |
| Hhip | Dhh Ihh Shh |
| Hmmr | Has2 |
| Hrh1 | Hdc |
| Hrh2 | Hdc |
| Hrh3 | Hdc |
| Hrh4 | Hdc |
| Htr1a | Tph1 |
| Htr1b | Tph1 |
| Htr1f | Tph1 |
| Htr2a | Tph1 |
| Htr2b | Tph1 |
| Htr2c | Tph1 |
| Htr4 | Tph1 |
| Htr5a | Tph1 |
| Htr6 | Tph1 |
| Htr7 | Tph1 |
| Igf1r | Igf1 Igf2 |
| Igf2r | Igf2 |
| Il12rb1 | Il12a Il12b Il23a |
| Il13ra2 | Il13 |
| Il15ra | Il15 |
| Il17rb | Il17b Il25 |
| Il17re | Il17c |
| Il18r1 | Il18 |
| Il18rap | Il18 |
| Il1r2 | Il1r1 Il1b Il1a |
| Il1rapl1 | Il18 |
| Il1rl1 | Il33 |
| Il21r | Il21 |
| Il2ra | Il2 Il1rn |
| Il2rb | Il2 Il15 Il1rn |
| Il2rg | Il15 Il13 Il7 Il9 Il2 Il4 Il21 Il1rn |
| Il5ra | Il5 |
| Il6st | Lif Osm |
| Il7r | Il7 Tslp |
| Il9r | Il9 |
| Insr | Igf2 |
| Itga1 | Col4a1 Dusp18 |
| Itga11 | Col1a1 |
| Itga2 | Col1a1 Fn1 |
| Itga2b | Fn1 |
| Itga3 | Lamc2 Reln |
| Itga4 | Fn1 Vcam1 Madcam1 |
| Itga6 | Lamc2 Lamb1 Adam2 Dusp18 |
| Itga8 | Fn1 Npnt |
| Itga9 | Adam12 |
| Itgad | Vcam1 |
| Itgae | Cdh1 |
| Itgal | Icam2 Icam5 Icam1 Icam4 |
| Itgav | Spp1 |
| Itgax | C3 Icam1 |
| Itgb1 | Adam12 Col18a1 Col1a1 Col4a1 Fn1 Lama2 Lamc2 Npnt Reln Vcam1 |
| Itgb2 | C3 Icam2 Vcam1 Icam5 Icam4 Icam1 |
| Itgb3 | Fn1 Vtn |
| Itgb4 | Lamc2 Dusp18 |
| Itgb6 | Fn1 |
| Itgb7 | Cdh1 Madcam1 |
| Itgb8 | Col4a1 Fn1 |
| Kdr | Pdgfc Vegfa Vegfc Sema6d |
| Kit | Kitl |
| Klrc2 | Gm11127 Gm7030 |
| Klrd1 | Gm11127 Gm7030 |
| Ldlr | Apoe |
| Lepr | Lep |
| Lgr4 | Ndp Rspo3 |
| Lgr5 | Rspo3 Rspo4 Rspo1 Rspo2 |
| Lgr6 | Rspo3 |
| Lifr | Lif Osm |
| Lpar1 | Liph |
| Lpar4 | Liph |
| Lrp1 | A2m C3 F8 Ltf Plat Serpine2 Serping1 |
| Lrp11 | Ltf |
| Lrp2 | Agt |
| Lrp5 | Apoe Sost |
| Lrp6 | Dkk2 Sost |
| Lrp8 | Apoe Reln |
| Ltbr | Ltb Tnfsf14 |
| Mc4r | Agrp Pomc |
| Mertk | Gas6 |
| Met | Sema5a |
| Mrgpra2b | Penk |
| Mrgpra4 | Tac1 |
| Mylk | Calm1 Calm3 Calm2 |
| Ngfr | Bdnf Ntf3 Ngf Ntf5 |
| Nmur1 | Nmu Nms |
| Notch1 | Dlk1 Jag1 |
| Notch2 | Dlk1 Jag1 |
| Notch3 | Dlk1 Jag1 Jag2 Dll4 Dll1 Dll3 |
| Notch4 | Dlk1 Jag1 |
| Npr2 | Nppc |
| Npr3 | Ostn Nppb Nppa |
| Npy1r | Npy Pyy |
| Npy2r | Npy |
| Npy4r | Npy |
| Nrp1 | Pgf Vegfa Vegfb Sema3a Sema3b |
| Ntrk2 | Bdnf Ntf3 Ntf5 |
| Ntsr1 | Nts |
| Ntsr2 | Nts |
| Ogfr | Penk |
| Oprd1 | Penk |
| Oprm1 | Penk |
| Osmr | Osm |
| Pdcd1 | Cd274 |
| Pde1c | Calm3 Calm1 Calm2 |
| Pdgfra | Pdgfa Pdgfc Pdgfb |
| Pdgfrb | Pdgfb Pdgfd |
| Plaur | Plau |
| Plxnb3 | Sema5a |
| Plxnc1 | Sema7a |
| Plxnd1 | Sema3e |
| Prlhr | Npy Pthlh |
| Prlr | Gh Prl4a1 Prl |
| Ptch2 | Dhh |
| Pth1r | Pthlh |
| Pth2r | Pthlh |
| Ptprb | Ptn Mdk |
| Ptprf | Nid1 |
| Ptprz1 | Ptn Mdk |
| Ramp1 | Calcb Calca Adm Adm2 Iapp |
| Ramp2 | Calcb Iapp Adm Calca Adm2 |
| Ramp3 | Calcb Adm Adm2 Calca Iapp |
| Ret | Gdnf |
| Robo1 | Ncam1 |
| Robo2 | Slit2 |
| Robo3 | Ncam1 |
| Robo4 | Slit2 |
| Rxfp1 | Insl3 Rln1 Rln3 |
| Ryk | Wnt5a |
| Scarb1 | Ptdss1 |
| Sdc1 | Thbs1 |
| Sdc2 | Fn1 |
| Sdc3 | Col5a3 |
| Sdc4 | Adam12 Fgf2 |
| Sele | Selplg |
| Sell | Cd34 Podxl2 |
| Selp | Cd24a Selplg |
| Sorl1 | Apoe |
| Sstr3 | Sst Cort |
| Tacr1 | Tac1 |
| Tacr2 | Tac1 |
| Tacr3 | Tac1 |
| Tek | Angpt1 Angpt2 Angptl1 Angpt4 |
| Tfrc | Trf |
| Tgfbr1 | Tgfb1 Tgfb2 Gdf10 Tgfb3 |
| Tgfbr2 | Tgfb1 Tgfb2 Tgfb3 |
| Tgfbr3 | Tgfb1 Tgfb2 Tgfb3 |
| Tie1 | Angpt1 |
| Tnfrsf11b | Tnfsf10 |
| Tnfrsf12a | Tnfsf12 |
| Tnfrsf13c | Tnfsf13b |
| Tnfrsf14 | Tnfsf14 |
| Tnfrsf18 | Tnfsf18 |
| Tnfrsf22 | Tnfsf10 |
| Tnfrsf26 | Tnfsf10 |
| Tnfrsf9 | Tnfsf9 |
| Tyro3 | Gas6 |
| Vldlr | Apoe Reln |
| Xcr1 | Xcl1 |
