# Supplementary material for: Sctensor detects many-to-many cell–cell interactions from single cell RNA-sequencing data
Source: BMC Bioinformatics. 2023 Nov 7;24:420. doi: 10.1186/s12859-023-05490-y (PMC10631077; doi:10.1186/s12859-023-05490-y)
Supplement: Supplementary file 17 — Additional file 17. HTML report of Uterus. [file 12859_2023_5490_MOESM17_ESM.zip › output/report/Mouse_Uterus/index.html]

The result of scTensor


# The result of scTensor

Koki Tsuyuzaki

#### 2022-09-19 23:34:44

# 1 About scTensor Algorithm

scTensor is the R/Bioconductor package for visualization of cell-cell interaction within single-cell RNA-Seq data. The calculation consists of several steps.

Firstly, LRBase.XXX.eg.db-type package is loaded for retriving ligand-receptor gene relationship (XXX is the abbreviation of some organisms like “Hsa” as Homo sapiens). scTensor searches the corresponding pair of genes in the rownames of input data matrix and extracted as vector. In this step, the gene identifier is limited as NCBI Gene ID for now.

Next, the all elements of extracted two vectors are summed up with all possible combination (Kronecker sum) and summarized as a matrix. Here, the multiple matrices can be represented as a three-order “tensor” (Ligand-Cell \* Receptor-Cell \* LR-Pair). scTensor decomposes the tensor into a small tensor (core tensor) and three factor matrices. Tensor decomposition is very similar to the matrix decomposition like PCA (principal component analysis). The core tensor is similar to eigenvalue of PCA and means how much the pattern is outstanding. Likewise, three matrices is similar to the PC scores and loadings of PCA and represents which ligand-cell/receptor-cell/LR-pair are informative. When the matrices have negative values, distinguishing that which direction (+/-) is important and which is not, is difficult to interpret and laboring task. That’s why, scTensor performs non-negative Tucker decomposition (NTD), which is non-negative version of Tucker decomposition (c.f. nnTensor).

Finaly, the result of NTD is summarized as this report. Most of plots belows are visualized by plotly package and interactively search the presise information of the plot. The three factor matrices can be interactively viewed and which celltypes are responds to the cell-cell interaction.The mode-3 (LR-pair) direction of sum of the core tensor is calculated and visualized as Ligand-Receptor Patterns. Detail of (Ligand-Cell, Receptor-Cell, LR-pair) Patterns are also visualized as below.

For more detail, visit the vignette of scTensor

# 2 Global statistics and plots

The result of scTensor is saved as a R binary file (reanalysis.RData).

```
load("reanalysis.RData")

# SingleCellExperiment object
sce
```

```
## class: SingleCellExperiment 
## dim: 2332 6443 
## metadata(12): lrbase ahid ... relchange hexbin
## assays(1): counts
## rownames(2332): 78687 68400 ... 16001 22339
## rowData names(0):
## colnames(6443): CATCAGTGTCTT AAAAACGCGTGG ... TTTTTTGAAGAN TTTTTTTGCAGG
## colData names(0):
## reducedDimNames(1): UMAP
## mainExpName: NULL
## altExpNames(0):
```

```
# Data size
metadata(sce)$datasize
```

```
## [1]  12  12 103
```

```
# Reduced data size
metadata(sce)$ranks
```

```
## [1] 7 6
```

```
# Reconstruction Error of NTD
head(metadata(sce)$recerror)
```

```
##      offset           1           2           3           4           5 
## 0.000000001 0.117067461 0.113087195 0.110321037 0.108417251 0.107162300
```

```
tail(metadata(sce)$recerror)
```

```
##        25        26        27        28        29        30 
## 0.1018284 0.1017413 0.1016592 0.1015813 0.1015070 0.1014356
```

```
# Relative Change of NTD
head(metadata(sce)$relchange)
```

```
##      offset           1           2           3           4           5 
## 0.000000001 0.928286966 0.035196435 0.025073709 0.017559809 0.011710748
```

```
tail(metadata(sce)$relchange)
```

```
##           25           26           27           28           29           30 
## 0.0009117788 0.0008565131 0.0008079868 0.0007666394 0.0007321785 0.0007036208
```

```
# Gene expression matrix
is(input)
```

```
## [1] "matrix"                              "array"                              
## [3] "mMatrix"                             "AnyMatrix"                          
## [5] "structure"                           "matrix_OR_array_OR_table_OR_numeric"
## [7] "vector"                              "vector_OR_Vector"                   
## [9] "vector_OR_factor"
```

```
dim(input)
```

```
## [1] 2332 6443
```

```
input[seq_len(2), seq_len(2)]
```

```
##       CATCAGTGTCTT AAAAACGCGTGG
## 78687            0            0
## 68400            0            0
```

```
# The result of 2D dimensional reduction (e.g. t-SNE)
is(twoD)
```

```
## [1] "matrix"                              "array"                              
## [3] "mMatrix"                             "AnyMatrix"                          
## [5] "structure"                           "matrix_OR_array_OR_table_OR_numeric"
## [7] "vector"                              "vector_OR_Vector"                   
## [9] "vector_OR_factor"
```

```
dim(twoD)
```

```
## [1] 6443    2
```

```
head(twoD)
```

```
##                   [,1]       [,2]
## CATCAGTGTCTT  5.313425 -4.6583234
## AAAAACGCGTGG -1.325132  5.0405720
## AAAAAGCGGCCT -5.197450 -0.9902320
## AAAAAGGTGGGC -6.186637  1.2303593
## AAAACAAACTCC -6.639764  0.7649436
## AAAACACAGTTA  4.730367 -4.4140116
```

```
# Ligand-Receptor corresponding table extracted from LRBase.XXX.eg.db
is(LR)
```

```
##  [1] "data.frame"       "list"             "oldClass"         "data.frameOrNULL"
##  [5] "vector"           "list_OR_List"     "AssayData"        "OptionalList"    
##  [9] "vector_OR_Vector" "vector_OR_factor"
```

```
dim(LR)
```

```
## [1] 535   4
```

```
head(LR, 2)
```

```
##   GENEID_L GENEID_R SOURCEID SOURCEDB
## 1   232345    16971     <NA>     DLRP
## 2    11489   104099     <NA>     DLRP
```

```
# Celltype label and color scheme
is(celltypes)
```

```
##  [1] "character"               "vector"                 
##  [3] "data.frameRowLabels"     "SuperClassMethod"       
##  [5] "character_OR_connection" "character_OR_NULL"      
##  [7] "atomic"                  "EnumerationValue"       
##  [9] "characterORMIAME"        "index"                  
## [11] "atomicVector"            "OptionalCharacter"      
## [13] "vector_OR_Vector"        "vector_OR_factor"
```

```
length(celltypes)
```

```
## [1] 6443
```

```
head(celltypes)
```

```
##    Stromal Epithelial   StromalP    Stromal Epithelial   Myocytes 
##  "#E7298A"  "#67001F"  "#980043"  "#E7298A"  "#67001F"  "#2166AC"
```

```
# Core tensor values
is(index)
```

```
## [1] "matrix"                              "array"                              
## [3] "mMatrix"                             "AnyMatrix"                          
## [5] "structure"                           "matrix_OR_array_OR_table_OR_numeric"
## [7] "vector"                              "vector_OR_Vector"                   
## [9] "vector_OR_factor"
```

```
dim(index)
```

```
## [1] 42  4
```

```
head(index)
```

```
##      Mode1 Mode2     Value Rank
## [1,]     2     5 0.1623942    1
## [2,]     4     5 0.1613479    2
## [3,]     6     4 0.1410419    3
## [4,]     7     5 0.1377598    4
## [5,]     5     5 0.1290692    5
## [6,]     7     3 0.1234811    6
```

```
is(corevalue)
```

```
##  [1] "numeric"                             "vector"                             
##  [3] "matrix_OR_array_OR_table_OR_numeric" "atomic"                             
##  [5] "EnumerationValue"                    "index"                              
##  [7] "replValue"                           "numLike"                            
##  [9] "number"                              "atomicVector"                       
## [11] "vector_OR_Vector"                    "vector_OR_factor"                   
## [13] "replValueSp"
```

```
length(corevalue)
```

```
## [1] 42
```

```
head(corevalue)
```

```
## selected selected selected selected selected selected 
## 6.129561 6.090072 5.323621 5.199739 4.871712 4.660790
```

```
# Selected corevalue position with thr threshold "thr"
is(selected)
```

```
##  [1] "integer"                             "double"                             
##  [3] "numeric"                             "vector"                             
##  [5] "data.frameRowLabels"                 "integer_OR_LLint"                   
##  [7] "atomic"                              "EnumerationValue"                   
##  [9] "atomicVector"                        "integer_OR_NULL"                    
## [11] "integer or NULL"                     "matrix_OR_array_OR_table_OR_numeric"
## [13] "vector_OR_Vector"                    "vector_OR_factor"                   
## [15] "index"                               "replValue"                          
## [17] "numLike"                             "number"                             
## [19] "replValueSp"
```

```
length(selected)
```

```
## [1] 42
```

```
head(selected)
```

```
## [1] 1 2 3 4 5 6
```

```
# The result of 2-class clustering
# The result of analysis in each L vector
is(ClusterL)
```

```
## [1] "matrix"                              "array"                              
## [3] "mMatrix"                             "AnyMatrix"                          
## [5] "structure"                           "matrix_OR_array_OR_table_OR_numeric"
## [7] "vector"                              "vector_OR_Vector"                   
## [9] "vector_OR_factor"
```

```
dim(ClusterL)
```

```
## [1]  7 12
```

```
# The result of analysis in each R vector
is(ClusterR)
```

```
## [1] "matrix"                              "array"                              
## [3] "mMatrix"                             "AnyMatrix"                          
## [5] "structure"                           "matrix_OR_array_OR_table_OR_numeric"
## [7] "vector"                              "vector_OR_Vector"                   
## [9] "vector_OR_factor"
```

```
dim(ClusterR)
```

```
## [1]  6 12
```

```
# The result of analysis in each LR vector
is(out.vecLR)
```

```
## [1] "list"             "vector"           "list_OR_List"     "AssayData"       
## [5] "OptionalList"     "vector_OR_Vector" "vector_OR_factor"
```

## 2.1 Number of cells in each celltype

```
color <- names(celltypes)
colors <- celltypes[vapply(unique(color), function(x){which(color == x)[1]}, 0L)]
numcells <- vapply(names(colors), function(x){length(which(color == x))}, 0L)
numcells <- data.frame(numcells=numcells, celltypes=names(numcells))

suppressPackageStartupMessages(library(plotly))
plot_ly(numcells, x=~celltypes, y=~numcells, type="bar", marker = list(color = colors))
```

## 2.2 Number of expressed genes in each celltype (Non-zero genes)

```
expgenes <- apply(input, 2, function(x){length(which(x != 0))})
expgenes <- data.frame(expgenes=expgenes, celltypes=names(celltypes))
plot_ly(expgenes, y=~expgenes, color=color, colors=colors, type="box")
```

## 2.3 Two dimensional plot of all cells

```
plot_ly(x=twoD[,1], y=twoD[,2], color = color, colors = colors, type = "scatter", text = rownames(twoD), mode = "markers")
```

## 2.4 Distribution of core tensor values

```
corenames <- vapply(seq_len(nrow(index)), function(x){paste(index[x,seq_len(3)], collapse=",")}, "")
plot_ly(x=seq_along(corevalue), y=corevalue, type="bar", color=names(corevalue), text=corenames, colors = c("#999999", "#E41A1C"))
```

# 3 Ligand-Cell Patterns

```
suppressPackageStartupMessages(library("heatmaply"))
l <- metadata(sce)$sctensor$ligand
if(nrow(l) >= 2){
rownames(l) <- paste0("(",seq_len(nrow(l)), ",*,*)")
heatmaply(l,xlab="Celltype",ylab="Pattern",fontsize_col=20,fontsize_row=20,subplot_widths=c(0.7, 0.1),subplot_heights=c(0.2, 0.7),labRow = rownames(l),labCol = colnames(l))
}
```

## 3.1 (1,*,*)-Pattern = Endothelial & Pericytes

## 3.2 (2,*,*)-Pattern = Epithelial & Myocytes 2 & 11

## 3.3 (3,*,*)-Pattern = Mesothelial

## 3.4 (4,*,*)-Pattern = Myocytes 2

## 3.5 (5,*,*)-Pattern = Stromal & StromalP & Endothelial

## 3.6 (6,*,*)-Pattern = Myeloid

## 3.7 (7,*,*)-Pattern = Stromal & StromalP & LEC

# 4 Receptor-Cell Patterns

```
r <- metadata(sce)$sctensor$receptor
if(nrow(r) >= 2){
rownames(r) <- paste0("(*,",seq_len(nrow(r)), ",*)")
heatmaply(r,xlab="Celltype",ylab="Pattern",fontsize_col=20,fontsize_row=20,subplot_widths=c(0.7, 0.1),subplot_heights=c(0.2, 0.7),labRow = rownames(r),labCol = colnames(r))
}
```

## 4.1 (*,1,*)-Pattern = Stromal & StromalP & Myeloid

## 4.2 (*,2,*)-Pattern = Epithelial & 11 & Mesothelial & LEC

## 4.3 (*,3,*)-Pattern = Pericytes

## 4.4 (*,4,*)-Pattern = Lymphocytes

## 4.5 (*,5,*)-Pattern = Endothelial

## 4.6 (*,6,*)-Pattern = StromalP & Myocytes 2

# 5 CCI-wise Hypergraph

# 6 Gene-wise Hypergraph

```
library("scTensor")
load("reanalysis.RData")
scTensor:::.myvisNetwork(g)
```

Details of Ligand Gene-centric Overview (selected)

Details of Ligand Gene-centric Overview (all)

Details of Receptor Gene-centric Overview (selected)

Details of Receptor Gene-centric Overview (all)

# 7 (Ligand-Cell, Receptor-Cell, ) -related L-R Pairs

## 7.1 (2,5,) -related L-R Pairs : (6.13%)

Details of (2,5,) -related L-R Pairs

## 7.2 (4,5,) -related L-R Pairs : (6.09%)

Details of (4,5,) -related L-R Pairs

## 7.3 (6,4,) -related L-R Pairs : (5.32%)

Details of (6,4,) -related L-R Pairs

## 7.4 (7,5,) -related L-R Pairs : (5.2%)

Details of (7,5,) -related L-R Pairs

## 7.5 (5,5,) -related L-R Pairs : (4.87%)

Details of (5,5,) -related L-R Pairs

## 7.6 (7,3,) -related L-R Pairs : (4.66%)

Details of (7,3,) -related L-R Pairs

## 7.7 (7,2,) -related L-R Pairs : (4.65%)

Details of (7,2,) -related L-R Pairs

## 7.8 (3,5,) -related L-R Pairs : (4.42%)

Details of (3,5,) -related L-R Pairs

## 7.9 (3,2,) -related L-R Pairs : (3.94%)

Details of (3,2,) -related L-R Pairs

## 7.10 (7,6,) -related L-R Pairs : (3.38%)

Details of (7,6,) -related L-R Pairs

## 7.11 (5,3,) -related L-R Pairs : (3.37%)

Details of (5,3,) -related L-R Pairs

## 7.12 (1,3,) -related L-R Pairs : (3.19%)

Details of (1,3,) -related L-R Pairs

## 7.13 (1,5,) -related L-R Pairs : (2.71%)

Details of (1,5,) -related L-R Pairs

## 7.14 (4,6,) -related L-R Pairs : (2.6%)

Details of (4,6,) -related L-R Pairs

## 7.15 (6,1,) -related L-R Pairs : (2.47%)

Details of (6,1,) -related L-R Pairs

## 7.16 (5,2,) -related L-R Pairs : (2.4%)

Details of (5,2,) -related L-R Pairs

## 7.17 (1,6,) -related L-R Pairs : (2.24%)

Details of (1,6,) -related L-R Pairs

## 7.18 (7,1,) -related L-R Pairs : (2.17%)

Details of (7,1,) -related L-R Pairs

## 7.19 (3,6,) -related L-R Pairs : (2.09%)

Details of (3,6,) -related L-R Pairs

## 7.20 (4,3,) -related L-R Pairs : (1.98%)

Details of (4,3,) -related L-R Pairs

## 7.21 (2,3,) -related L-R Pairs : (1.94%)

Details of (2,3,) -related L-R Pairs

## 7.22 (2,1,) -related L-R Pairs : (1.83%)

Details of (2,1,) -related L-R Pairs

## 7.23 (3,1,) -related L-R Pairs : (1.82%)

Details of (3,1,) -related L-R Pairs

## 7.24 (1,4,) -related L-R Pairs : (1.54%)

Details of (1,4,) -related L-R Pairs

## 7.25 (7,4,) -related L-R Pairs : (1.52%)

Details of (7,4,) -related L-R Pairs

## 7.26 (6,3,) -related L-R Pairs : (1.48%)

Details of (6,3,) -related L-R Pairs

## 7.27 (4,2,) -related L-R Pairs : (1.47%)

Details of (4,2,) -related L-R Pairs

## 7.28 (2,2,) -related L-R Pairs : (1.44%)

Details of (2,2,) -related L-R Pairs

## 7.29 (1,1,) -related L-R Pairs : (1.42%)

Details of (1,1,) -related L-R Pairs

## 7.30 (1,2,) -related L-R Pairs : (1.28%)

Details of (1,2,) -related L-R Pairs

## 7.31 (3,4,) -related L-R Pairs : (1.06%)

Details of (3,4,) -related L-R Pairs

## 7.32 (6,6,) -related L-R Pairs : (1.06%)

Details of (6,6,) -related L-R Pairs

## 7.33 (3,3,) -related L-R Pairs : (1.06%)

Details of (3,3,) -related L-R Pairs

## 7.34 (5,6,) -related L-R Pairs : (1.05%)

Details of (5,6,) -related L-R Pairs

## 7.35 (5,4,) -related L-R Pairs : (0.98%)

Details of (5,4,) -related L-R Pairs

## 7.36 (4,4,) -related L-R Pairs : (0.89%)

Details of (4,4,) -related L-R Pairs

## 7.37 (5,1,) -related L-R Pairs : (0.86%)

Details of (5,1,) -related L-R Pairs

## 7.38 (2,6,) -related L-R Pairs : (0.85%)

Details of (2,6,) -related L-R Pairs

## 7.39 (4,1,) -related L-R Pairs : (0.76%)

Details of (4,1,) -related L-R Pairs

## 7.40 (6,2,) -related L-R Pairs : (0.76%)

Details of (6,2,) -related L-R Pairs

## 7.41 (6,5,) -related L-R Pairs : (0.59%)

Details of (6,5,) -related L-R Pairs

## 7.42 (2,4,) -related L-R Pairs : (0.48%)

Details of (2,4,) -related L-R Pairs

# 8 Session Information

```
sessionInfo()
```

```
## R version 4.1.2 (2021-11-01)
## Platform: x86_64-pc-linux-gnu (64-bit)
## Running under: Ubuntu 20.04.3 LTS
## 
## Matrix products: default
## BLAS/LAPACK: /usr/lib/x86_64-linux-gnu/openblas-pthread/libopenblasp-r0.3.8.so
## 
## locale:
##  [1] LC_CTYPE=en_US.UTF-8       LC_NUMERIC=C              
##  [3] LC_TIME=en_US.UTF-8        LC_COLLATE=en_US.UTF-8    
##  [5] LC_MONETARY=en_US.UTF-8    LC_MESSAGES=C             
##  [7] LC_PAPER=en_US.UTF-8       LC_NAME=C                 
##  [9] LC_ADDRESS=C               LC_TELEPHONE=C            
## [11] LC_MEASUREMENT=en_US.UTF-8 LC_IDENTIFICATION=C       
## 
## attached base packages:
## [1] stats4    stats     graphics  grDevices utils     datasets  methods  
## [8] base     
## 
## other attached packages:
##  [1] heatmaply_1.3.0                          
##  [2] BiocStyle_2.22.0                         
##  [3] plotly_4.10.0                            
##  [4] RSQLite_2.2.9                            
##  [5] SingleCellExperiment_1.16.0              
##  [6] fields_13.3                              
##  [7] spam_2.8-0                               
##  [8] irlba_2.3.5                              
##  [9] Rtsne_0.15                               
## [10] uwot_0.1.11                              
## [11] mclust_5.4.9                             
## [12] dbscan_1.1-10                            
## [13] viridis_0.6.2                            
## [14] viridisLite_0.4.0                        
## [15] ggplot2_3.3.5                            
## [16] AnnotationHub_3.2.1                      
## [17] BiocFileCache_2.2.1                      
## [18] dbplyr_2.1.1                             
## [19] LRBaseDbi_2.4.0                          
## [20] statmod_1.4.36                           
## [21] DESeq2_1.34.0                            
## [22] SummarizedExperiment_1.24.0              
## [23] MatrixGenerics_1.6.0                     
## [24] matrixStats_0.61.0                       
## [25] Mus.musculus_1.3.1                       
## [26] TxDb.Mmusculus.UCSC.mm10.knownGene_3.10.0
## [27] org.Mm.eg.db_3.14.0                      
## [28] Homo.sapiens_1.3.1                       
## [29] TxDb.Hsapiens.UCSC.hg19.knownGene_3.2.2  
## [30] org.Hs.eg.db_3.14.0                      
## [31] GO.db_3.14.0                             
## [32] OrganismDbi_1.36.0                       
## [33] GenomicFeatures_1.46.4                   
## [34] GenomicRanges_1.46.1                     
## [35] GenomeInfoDb_1.30.1                      
## [36] AnnotationDbi_1.56.2                     
## [37] IRanges_2.28.0                           
## [38] S4Vectors_0.32.3                         
## [39] Biobase_2.54.0                           
## [40] BiocGenerics_0.40.0                      
## [41] Matrix_1.4-0                             
## [42] rTensor_1.4.8                            
## [43] nnTensor_1.1.5                           
## [44] ROCR_1.0-11                              
## [45] RColorBrewer_1.1-2                       
## [46] scales_1.1.1                             
## [47] scTGIF_1.8.0                             
## [48] scTensor_2.4.1                           
## [49] igraph_1.2.11                            
## 
## loaded via a namespace (and not attached):
##   [1] ica_1.0-2                     Rsamtools_2.10.0             
##   [3] foreach_1.5.2                 lmtest_0.9-39                
##   [5] crayon_1.4.2                  spatstat.core_2.3-2          
##   [7] MASS_7.3-55                   nlme_3.1-155                 
##   [9] backports_1.4.1               GOSemSim_2.20.0              
##  [11] MeSHDbi_1.30.0                rlang_1.0.1                  
##  [13] XVector_0.34.0                filelock_1.0.2               
##  [15] GOstats_2.60.0                BiocParallel_1.28.3          
##  [17] rjson_0.2.21                  tagcloud_0.6                 
##  [19] bit64_4.0.5                   glue_1.6.1                   
##  [21] sctransform_0.3.3             parallel_4.1.2               
##  [23] spatstat.sparse_2.1-0         dotCall64_1.0-1              
##  [25] tcltk_4.1.2                   DOSE_3.20.1                  
##  [27] spatstat.geom_2.3-1           tidyselect_1.1.1             
##  [29] SeuratObject_4.0.4            fitdistrplus_1.1-6           
##  [31] XML_3.99-0.8                  tidyr_1.2.0                  
##  [33] zoo_1.8-9                     GenomicAlignments_1.30.0     
##  [35] xtable_1.8-4                  magrittr_2.0.2               
##  [37] evaluate_0.14                 cli_3.1.1                    
##  [39] zlibbioc_1.40.0               miniUI_0.1.1.1               
##  [41] bslib_0.3.1                   rpart_4.1.16                 
##  [43] fastmatch_1.1-3               treeio_1.18.1                
##  [45] maps_3.4.0                    shiny_1.7.1                  
##  [47] xfun_0.29                     cluster_2.1.2                
##  [49] tidygraph_1.2.0               TSP_1.1-11                   
##  [51] KEGGREST_1.34.0               tibble_3.1.6                 
##  [53] interactiveDisplayBase_1.32.0 ggrepel_0.9.1                
##  [55] ape_5.6-1                     listenv_0.8.0                
##  [57] dendextend_1.15.2             Biostrings_2.62.0            
##  [59] png_0.1-7                     future_1.23.0                
##  [61] withr_2.4.3                   bitops_1.0-7                 
##  [63] ggforce_0.3.3                 RBGL_1.70.0                  
##  [65] plyr_1.8.6                    GSEABase_1.56.0              
##  [67] pillar_1.7.0                  cachem_1.0.6                 
##  [69] graphite_1.40.0               vctrs_0.3.8                  
##  [71] ellipsis_0.3.2                generics_0.1.2               
##  [73] plot3D_1.4                    outliers_0.14                
##  [75] tools_4.1.2                   entropy_1.3.1                
##  [77] munsell_0.5.0                 tweenr_1.0.2                 
##  [79] fgsea_1.20.0                  DelayedArray_0.20.0          
##  [81] fastmap_1.1.0                 compiler_4.1.2               
##  [83] abind_1.4-5                   httpuv_1.6.5                 
##  [85] rtracklayer_1.54.0            GenomeInfoDbData_1.2.7       
##  [87] gridExtra_2.3                 lattice_0.20-45              
##  [89] deldir_1.0-6                  visNetwork_2.1.0             
##  [91] AnnotationForge_1.36.0        utf8_1.2.2                   
##  [93] later_1.3.0                   dplyr_1.0.7                  
##  [95] jsonlite_1.7.3                ccTensor_1.0.2               
##  [97] concaveman_1.1.0              graph_1.72.0                 
##  [99] tidytree_0.3.7                pbapply_1.5-0                
## [101] genefilter_1.76.0             lazyeval_0.2.2               
## [103] promises_1.2.0.1              goftest_1.2-3                
## [105] spatstat.utils_2.3-0          reticulate_1.24              
## [107] checkmate_2.0.0               rmarkdown_2.11               
## [109] cowplot_1.1.1                 schex_1.8.0                  
## [111] webshot_0.5.2                 survival_3.2-13              
## [113] yaml_2.2.2                    plotrix_3.8-2                
## [115] htmltools_0.5.2               memoise_2.0.1                
## [117] BiocIO_1.4.0                  Seurat_4.1.0                 
## [119] locfit_1.5-9.4                seriation_1.3.1              
## [121] graphlayouts_0.8.0            digest_0.6.29                
## [123] assertthat_0.2.1              ReactomePA_1.38.0            
## [125] mime_0.12                     rappdirs_0.3.3               
## [127] registry_0.5-1                yulab.utils_0.0.4            
## [129] future.apply_1.8.1            misc3d_0.9-1                 
## [131] data.table_1.14.2             blob_1.2.2                   
## [133] labeling_0.4.2                splines_4.1.2                
## [135] RCurl_1.98-1.5                hms_1.1.1                    
## [137] colorspace_2.0-2              BiocManager_1.30.16          
## [139] aplot_0.1.2                   sass_0.4.0                   
## [141] bookdown_0.24                 Rcpp_1.0.8                   
## [143] RANN_2.6.1                    enrichplot_1.14.1            
## [145] fansi_1.0.2                   parallelly_1.30.0            
## [147] R6_2.5.1                      grid_4.1.2                   
## [149] ggridges_0.5.3                lifecycle_1.0.1              
## [151] curl_4.3.2                    jquerylib_0.1.4              
## [153] leiden_0.3.9                  meshr_2.0.2                  
## [155] DO.db_2.9                     qvalue_2.26.0                
## [157] RcppAnnoy_0.0.19              iterators_1.0.14             
## [159] stringr_1.4.0                 htmlwidgets_1.5.4            
## [161] polyclip_1.10-0               biomaRt_2.50.3               
## [163] purrr_0.3.4                   crosstalk_1.2.0              
## [165] shadowtext_0.1.1              gridGraphics_0.5-1           
## [167] reactome.db_1.77.0            mgcv_1.8-38                  
## [169] globals_0.14.0                patchwork_1.1.1              
## [171] codetools_0.2-18              prettyunits_1.1.1            
## [173] RSpectra_0.16-0               gtable_0.3.0                 
## [175] DBI_1.1.2                     ggfun_0.0.5                  
## [177] tensor_1.5                    httr_1.4.2                   
## [179] KernSmooth_2.23-20            stringi_1.7.6                
## [181] progress_1.2.2                reshape2_1.4.4               
## [183] msigdbr_7.4.1                 farver_2.1.0                 
## [185] annotate_1.72.0               hexbin_1.28.2                
## [187] fdrtool_1.2.17                Rgraphviz_2.38.0             
## [189] ggtree_3.2.1                  xml2_1.3.3                   
## [191] restfulr_0.0.13               geneplotter_1.72.0           
## [193] ggplotify_0.1.0               Category_2.60.0              
## [195] scattermore_0.7               BiocVersion_3.14.0           
## [197] bit_4.0.4                     scatterpie_0.1.7             
## [199] spatstat.data_2.1-2           ggraph_2.0.5                 
## [201] pkgconfig_2.0.3               babelgene_21.4               
## [203] knitr_1.37
```

# 9 License

Copyright (c) 2018 Koki Tsuyuzaki and Laboratory for Bioinformatics Research, RIKEN Center for Biosystems Dynamics Reseach Released under the Artistic License 2.0
