# Supplementary material for: Sctensor detects many-to-many cell–cell interactions from single cell RNA-sequencing data
Source: BMC Bioinformatics. 2023 Nov 7;24:420. doi: 10.1186/s12859-023-05490-y (PMC10631077; doi:10.1186/s12859-023-05490-y)
Supplement: Supplementary file 17 — Additional file 17. HTML report of Uterus. [file 12859_2023_5490_MOESM17_ESM.zip › output/report/Mouse_Uterus/pattern_5_2.html]

Details of (5,2,) -related L-R pairs


# Details of (5,2,) -related L-R pairs

# (5,2,:) Pattern

# (5,2,) -related L-R pairs (Top10 pairs)

```
library("scTensor")
load("reanalysis.RData")
col <- .setColor("many")[16]
scTensor:::.myvisNetwork(g, col)
```

| Rank | Ligand Gene | Receptor Gene | Ligand Expression (Log10(exp + 1)) | Receptor Expression (Log10(exp + 1)) | LR-pair factor value (Percentage) | P-value (Grubbs test) | Q-value (BH method) | L-R Evidence cf. rikenbit/lrbase-workflow | PubMed |
| --- | --- | --- | --- | --- | --- | --- | --- | --- | --- |
| 1 | Nid1 Description: Nid1nidogen 1 GO: 1-19 Reactome: 1-3 UniProtKB: 1 STRING: 1 RefEx: Nid1 Expression Atlas: Nid1 Single Cell Expression Atlas: Nid1 PanglaoDB: Nid1 MeSH: 1-95 96-190 191-285 286-380 381-475 476-570 571-665 666-760 761-855 856-950 951-1045 1046-1140 1141-1235 1236-1327 | Ptprf Description: Nid1protein tyrosine phosphatase, receptor type, F GO: 1-44 Reactome: 1-4 UniProtKB: 1-2 RefEx: Nid1 Expression Atlas: Ptprf Single Cell Expression Atlas: Ptprf PanglaoDB: Ptprf MeSH: 1-96 97-192 193-288 289-384 385-478 |  |  | 0.027 (42.014%) | 0 | 0 | DLRP | 1 |
| 2 | Igf1 Description: Igf1insulin-like growth factor 1 GO: 1-70 71-139 Reactome: 1-14 UniProtKB: 1-6 RefEx: Igf1 Expression Atlas: Igf1 Single Cell Expression Atlas: Igf1 PanglaoDB: Igf1 MeSH: 1-99 100-198 199-297 298-396 397-495 496-594 595-693 694-792 793-891 892-990 991-1089 1090-1188 1189-1287 1288-1386 1387-1485 1486-1584 1585-1683 1684-1782 1783-1881 1882-1980 1981-2079 2080-2178 2179-2277 2278-2376 2377-2475 2476-2566 | Igf1r Description: Igf1insulin-like growth factor I receptor GO: 1-97 Reactome: 1-9 UniProtKB: 1-3 RefEx: Igf1 Expression Atlas: Igf1r Single Cell Expression Atlas: Igf1r PanglaoDB: Igf1r MeSH: 1-100 101-200 201-300 301-400 401-500 501-600 601-700 701-800 801-900 901-1000 1001-1100 1101-1200 1201-1300 1301-1400 1401-1500 1501-1600 1601-1688 |  |  | 0.006 (10.059%) | 0 | 0 | DLRP | 1 |
| 3 | Rspo3 Description: Rspo3R-spondin 3 GO: 1-13 Reactome: 1-4 UniProtKB: 1 RefEx: Rspo3 Expression Atlas: Rspo3 Single Cell Expression Atlas: Rspo3 PanglaoDB: Rspo3 MeSH: 1-83 84-166 167-249 250-332 | Lgr5 Description: Rspo3leucine rich repeat containing G protein coupled receptor 5 GO: 1-22 Reactome: 1-4 UniProtKB: 1 RefEx: Rspo3 Expression Atlas: Lgr5 Single Cell Expression Atlas: Lgr5 PanglaoDB: Lgr5 MeSH: 1-100 101-200 201-300 301-400 401-500 501-600 601-700 701-800 801-900 901-1000 1001-1100 1101-1197 |  |  | 0.005 (8.297%) | 0 | 0 | DLRP | 1 |
| 4 | Edn3 Description: Edn3endothelin 3 GO: 1-34 Reactome: 1-7 UniProtKB: 1-2 RefEx: Edn3 Expression Atlas: Edn3 Single Cell Expression Atlas: Edn3 PanglaoDB: Edn3 MeSH: 1-95 96-190 191-285 286-380 381-474 | Ednrb Description: Edn3endothelin receptor type B GO: 1-66 Reactome: 1-7 UniProtKB: 1 RefEx: Edn3 Expression Atlas: Ednrb Single Cell Expression Atlas: Ednrb PanglaoDB: Ednrb MeSH: 1-100 101-200 201-300 301-400 401-500 501-600 601-700 701-800 801-900 901-996 |  |  | 0.005 (8.179%) | 0 | 0 | DLRP | 1 |
| 5 | Col5a3 Description: Col5a3collagen, type V, alpha 3 GO: 1-11 Reactome: 1-21 UniProtKB: 1 STRING: 1 RefEx: Col5a3 Expression Atlas: Col5a3 Single Cell Expression Atlas: Col5a3 PanglaoDB: Col5a3 MeSH: 1-91 92-182 | Sdc3 Description: Col5a3syndecan 3 GO: 1-14 Reactome: 1-18 UniProtKB: 1 RefEx: Col5a3 Expression Atlas: Sdc3 Single Cell Expression Atlas: Sdc3 PanglaoDB: Sdc3 MeSH: 1-95 96-190 191-285 286-380 381-473 |  |  | 0.004 (6.917%) | 0 | 0 | DLRP | 1 |
| 6 | Wnt5a Description: Wnt5awingless-type MMTV integration site family, member 5A GO: 1-92 93-183 Reactome: 1-14 UniProtKB: 1 RefEx: Wnt5a Expression Atlas: Wnt5a Single Cell Expression Atlas: Wnt5a PanglaoDB: Wnt5a MeSH: 1-96 97-192 193-288 289-384 385-480 481-576 577-672 673-768 769-864 865-960 961-1056 1057-1152 1153-1248 1249-1344 1345-1440 1441-1531 | Fzd6 Description: Wnt5afrizzled class receptor 6 GO: 1-35 Reactome: 1-7 UniProtKB: 1-3 RefEx: Wnt5a Expression Atlas: Fzd6 Single Cell Expression Atlas: Fzd6 PanglaoDB: Fzd6 MeSH: 1-94 95-188 189-282 283-376 377-470 471-563 |  |  | 0.004 (6.175%) | 0 | 0 | DLRP | 1 |
| 7 | Ptn Description: Ptnpleiotrophin GO: 1-68 Reactome: 1-3 UniProtKB: 1 RefEx: Ptn Expression Atlas: Ptn Single Cell Expression Atlas: Ptn PanglaoDB: Ptn MeSH: 1-100 101-200 201-300 301-400 401-500 501-600 601-700 701-800 801-900 901-1000 1001-1100 1101-1200 1201-1300 1301-1393 | Ptprb Description: Ptnprotein tyrosine phosphatase, receptor type, B GO: 1-13 Reactome: 1-3 UniProtKB: 1-2 RefEx: Ptn Expression Atlas: Ptprb Single Cell Expression Atlas: Ptprb PanglaoDB: Ptprb MeSH: 1-76 77-152 153-228 229-304 |  |  | 0.002 (3.911%) | 0 | 0 | DLRP | 1 |
| 8 | Adam12 Description: Adam12a disintegrin and metallopeptidase domain 12 (meltrin alpha) GO: 1-14 Reactome: 1-2 UniProtKB: 1 RefEx: Adam12 Expression Atlas: Adam12 Single Cell Expression Atlas: Adam12 PanglaoDB: Adam12 MeSH: 1-93 94-186 187-279 280-372 373-463 | Sdc4 Description: Adam12syndecan 4 GO: 1-24 Reactome: 1-18 UniProtKB: 1-2 STRING: 1 RefEx: Adam12 Expression Atlas: Sdc4 Single Cell Expression Atlas: Sdc4 PanglaoDB: Sdc4 MeSH: 1-97 98-194 195-291 292-388 389-485 486-582 583-679 680-776 777-867 |  |  | 0.002 (3.852%) | 0 | 0 | DLRP | 1 |
| 9 | Angpt1 Description: Angpt1angiopoietin 1 GO: 1-59 Reactome: 1-7 UniProtKB: 1 RefEx: Angpt1 Expression Atlas: Angpt1 Single Cell Expression Atlas: Angpt1 PanglaoDB: Angpt1 MeSH: 1-100 101-200 201-300 301-400 401-500 501-600 601-700 701-800 801-900 901-998 | Tie1 Description: Angpt1tyrosine kinase with immunoglobulin-like and EGF-like domains 1 GO: 1-32 UniProtKB: 1 RefEx: Angpt1 Expression Atlas: Tie1 Single Cell Expression Atlas: Tie1 PanglaoDB: Tie1 MeSH: 1-89 90-178 179-267 268-356 357-445 446-534 535-617 |  |  | 0.002 (2.965%) | 0 | 0 | DLRP | 1 |
| 10 | Inhba Description: Inhbainhibin beta-A GO: 1-50 Reactome: 1-8 UniProtKB: 1-2 RefEx: Inhba Expression Atlas: Inhba Single Cell Expression Atlas: Inhba PanglaoDB: Inhba MeSH: 1-100 101-200 201-300 301-400 401-500 501-600 601-700 701-800 801-900 901-1000 1001-1099 | Bambi Description: InhbaBMP and activin membrane-bound inhibitor GO: 1-16 Reactome: 1-5 UniProtKB: 1 STRING: 1 RefEx: Inhba Expression Atlas: Bambi Single Cell Expression Atlas: Bambi PanglaoDB: Bambi MeSH: 1-88 89-176 177-264 265-352 |  |  | 0.001 (1.948%) | 1 | 1 | DLRP | 1 |

# (5,2,) -related Enrichment Analysis

```
load("reanalysis.RData")
library("plotly")
```

## MeSH-Enrichment Analysis (A : Anatomy)

```
negLogPval <- -log10(out.vecLR[['pattern_5_2']]$Enrich$MeSH_A$Pvalue + 1E-10)
term <- out.vecLR[['pattern_5_2']]$Enrich$MeSH_A$Term
target <- seq_len(min(100, length(negLogPval)))
negLogPval <- negLogPval[target]
term <- term[target]
p <- plot_ly(x=seq_along(negLogPval), y=~negLogPval,
type="bar", color=~negLogPval, text=term,
colors=c("#4b61ba", "gray", "#a87963", "red"))
layout(p, title="MeSH-Enrichment Analysis (A)",
   xaxis=list(title="Term"),
   yaxis=list(title="-Log(P-value)"),
   legend=list(name=""))
```

```
## Warning: textfont.color doesn't (yet) support data arrays

## Warning: textfont.color doesn't (yet) support data arrays
```

## MeSH-Enrichment Analysis (B : Organisms)

```
negLogPval <- -log10(out.vecLR[['pattern_5_2']]$Enrich$MeSH_B$Pvalue + 1E-10)
term <- out.vecLR[['pattern_5_2']]$Enrich$MeSH_B$Term
target <- seq_len(min(100, length(negLogPval)))
negLogPval <- negLogPval[target]
term <- term[target]
p <- plot_ly(x=seq_along(negLogPval), y=~negLogPval,
type="bar", color=~negLogPval, text=term,
colors=c("#4b61ba", "gray", "#a87963", "red"))
layout(p, title="MeSH-Enrichment Analysis (B)",
   xaxis=list(title="Term"),
   yaxis=list(title="-Log(P-value)"),
   legend=list(name=""))
```

```
## Warning: textfont.color doesn't (yet) support data arrays

## Warning: textfont.color doesn't (yet) support data arrays
```

## MeSH-Enrichment Analysis (C : Diseases)

```
negLogPval <- -log10(out.vecLR[['pattern_5_2']]$Enrich$MeSH_C$Pvalue + 1E-10)
term <- out.vecLR[['pattern_5_2']]$Enrich$MeSH_C$Term
target <- seq_len(min(100, length(negLogPval)))
negLogPval <- negLogPval[target]
term <- term[target]
p <- plot_ly(x=seq_along(negLogPval), y=~negLogPval,
type="bar", color=~negLogPval, text=term,
colors=c("#4b61ba", "gray", "#a87963", "red"))
layout(p, title="MeSH-Enrichment Analysis (C)",
   xaxis=list(title="Term"),
   yaxis=list(title="-Log(P-value)"),
   legend=list(name=""))
```

```
## Warning: textfont.color doesn't (yet) support data arrays

## Warning: textfont.color doesn't (yet) support data arrays
```

## MeSH-Enrichment Analysis (D : Drugs)

```
negLogPval <- -log10(out.vecLR[['pattern_5_2']]$Enrich$MeSH_D$Pvalue + 1E-10)
term <- out.vecLR[['pattern_5_2']]$Enrich$MeSH_D$Term
target <- seq_len(min(100, length(negLogPval)))
negLogPval <- negLogPval[target]
term <- term[target]
p <- plot_ly(x=seq_along(negLogPval), y=~negLogPval,
type="bar", color=~negLogPval, text=term,
colors=c("#4b61ba", "gray", "#a87963", "red"))
layout(p, title="MeSH-Enrichment Analysis (D)",
   xaxis=list(title="Term"),
   yaxis=list(title="-Log(P-value)"),
   legend=list(name=""))
```

```
## Warning: textfont.color doesn't (yet) support data arrays

## Warning: textfont.color doesn't (yet) support data arrays
```

## MeSH-Enrichment Analysis (E : Analytical, Diagnostic and Therapeutic Techniques and Equipment)

```
negLogPval <- -log10(out.vecLR[['pattern_5_2']]$Enrich$MeSH_E$Pvalue + 1E-10)
term <- out.vecLR[['pattern_5_2']]$Enrich$MeSH_E$Term
target <- seq_len(min(100, length(negLogPval)))
negLogPval <- negLogPval[target]
term <- term[target]
p <- plot_ly(x=seq_along(negLogPval), y=~negLogPval,
type="bar", color=~negLogPval, text=term,
colors=c("#4b61ba", "gray", "#a87963", "red"))
layout(p, title="MeSH-Enrichment Analysis (E)",
   xaxis=list(title="Term"),
   yaxis=list(title="-Log(P-value)"),
   legend=list(name=""))
```

```
## Warning: textfont.color doesn't (yet) support data arrays

## Warning: textfont.color doesn't (yet) support data arrays
```

## MeSH-Enrichment Analysis (G : Phenomena and Processes)

```
negLogPval <- -log10(out.vecLR[['pattern_5_2']]$Enrich$MeSH_G$Pvalue + 1E-10)
term <- out.vecLR[['pattern_5_2']]$Enrich$MeSH_G$Term
target <- seq_len(min(100, length(negLogPval)))
negLogPval <- negLogPval[target]
term <- term[target]
p <- plot_ly(x=seq_along(negLogPval), y=~negLogPval,
type="bar", color=~negLogPval, text=term,
colors=c("#4b61ba", "gray", "#a87963", "red"))
layout(p, title="MeSH-Enrichment Analysis (G)",
   xaxis=list(title="Term"),
   yaxis=list(title="-Log(P-value)"),
   legend=list(name=""))
```

```
## Warning: textfont.color doesn't (yet) support data arrays

## Warning: textfont.color doesn't (yet) support data arrays
```

## MeSH-Enrichment Analysis (H : Disciplines and Occupations)

```
negLogPval <- -log10(out.vecLR[['pattern_5_2']]$Enrich$MeSH_H$Pvalue + 1E-10)
term <- out.vecLR[['pattern_5_2']]$Enrich$MeSH_H$Term
target <- seq_len(min(100, length(negLogPval)))
negLogPval <- negLogPval[target]
term <- term[target]
p <- plot_ly(x=seq_along(negLogPval), y=~negLogPval,
type="bar", color=~negLogPval, text=term,
colors=c("#4b61ba", "gray", "#a87963", "red"))
layout(p, title="MeSH-Enrichment Analysis (H)",
   xaxis=list(title="Term"),
   yaxis=list(title="-Log(P-value)"),
   legend=list(name=""))
```

```
## Warning: textfont.color doesn't (yet) support data arrays

## Warning: textfont.color doesn't (yet) support data arrays
```

## MeSH-Enrichment Analysis (J : Technology and Food and Beverages)

```
negLogPval <- -log10(out.vecLR[['pattern_5_2']]$Enrich$MeSH_J$Pvalue + 1E-10)
term <- out.vecLR[['pattern_5_2']]$Enrich$MeSH_J$Term
target <- seq_len(min(100, length(negLogPval)))
negLogPval <- negLogPval[target]
term <- term[target]
p <- plot_ly(x=seq_along(negLogPval), y=~negLogPval,
type="bar", color=~negLogPval, text=term,
colors=c("#4b61ba", "gray", "#a87963", "red"))
layout(p, title="MeSH-Enrichment Analysis (J)",
   xaxis=list(title="Term"),
   yaxis=list(title="-Log(P-value)"),
   legend=list(name=""))
```

## MeSH-Enrichment Analysis (L : Information Science)

```
negLogPval <- -log10(out.vecLR[['pattern_5_2']]$Enrich$MeSH_L$Pvalue + 1E-10)
term <- out.vecLR[['pattern_5_2']]$Enrich$MeSH_L$Term
target <- seq_len(min(100, length(negLogPval)))
negLogPval <- negLogPval[target]
term <- term[target]
p <- plot_ly(x=seq_along(negLogPval), y=~negLogPval,
type="bar", color=~negLogPval, text=term,
colors=c("#4b61ba", "gray", "#a87963", "red"))
layout(p, title="MeSH-Enrichment Analysis (L)",
   xaxis=list(title="Term"),
   yaxis=list(title="-Log(P-value)"),
   legend=list(name=""))
```

## MeSH-Enrichment Analysis (N : Health Care)

```
negLogPval <- -log10(out.vecLR[['pattern_5_2']]$Enrich$MeSH_N$Pvalue + 1E-10)
term <- out.vecLR[['pattern_5_2']]$Enrich$MeSH_N$Term
target <- seq_len(min(100, length(negLogPval)))
negLogPval <- negLogPval[target]
term <- term[target]
p <- plot_ly(x=seq_along(negLogPval), y=~negLogPval,
type="bar", color=~negLogPval, text=term,
colors=c("#4b61ba", "gray", "#a87963", "red"))
layout(p, title="MeSH-Enrichment Analysis (N)",
   xaxis=list(title="Term"),
   yaxis=list(title="-Log(P-value)"),
   legend=list(name=""))
```

```
## Warning: textfont.color doesn't (yet) support data arrays

## Warning: textfont.color doesn't (yet) support data arrays
```

## MeSH-Enrichment Analysis (Z : Geographical Locations)

```
negLogPval <- -log10(out.vecLR[['pattern_5_2']]$Enrich$MeSH_Z$Pvalue + 1E-10)
term <- out.vecLR[['pattern_5_2']]$Enrich$MeSH_Z$Term
target <- seq_len(min(100, length(negLogPval)))
negLogPval <- negLogPval[target]
term <- term[target]
p <- plot_ly(x=seq_along(negLogPval), y=~negLogPval,
type="bar", color=~negLogPval, text=term,
colors=c("#4b61ba", "gray", "#a87963", "red"))
layout(p, title="MeSH-Enrichment Analysis (Z)",
   xaxis=list(title="Term"),
   yaxis=list(title="-Log(P-value)"),
   legend=list(name=""))
```

## Reactome Pathway-Enrichment Analysis

```
negLogPval <- -log10(out.vecLR[['pattern_5_2']]$Enrich$Reactome$Pvalue + 1E-10)
term <- out.vecLR[['pattern_5_2']]$Enrich$Reactome$Term
target <- seq_len(min(100, length(negLogPval)))
negLogPval <- negLogPval[target]
term <- term[target]
p <- plot_ly(x=seq_along(negLogPval), y=~negLogPval,
type="bar", color=~negLogPval, text=term,
colors=c("#4b61ba", "gray", "#a87963", "red"))
layout(p, title="Reactome-Enrichment Analysis",
   xaxis=list(title="Term"),
   yaxis=list(title="-Log(P-value)"),
   legend=list(name=""))
```

```
## Warning: textfont.color doesn't (yet) support data arrays

## Warning: textfont.color doesn't (yet) support data arrays
```
