# Supplementary material for: Sctensor detects many-to-many cell–cell interactions from single cell RNA-sequencing data
Source: BMC Bioinformatics. 2023 Nov 7;24:420. doi: 10.1186/s12859-023-05490-y (PMC10631077; doi:10.1186/s12859-023-05490-y)
Supplement: Supplementary file 17 — Additional file 17. HTML report of Uterus. [file 12859_2023_5490_MOESM17_ESM.zip › output/report/Mouse_Uterus/pattern_7_1.html]

Details of (7,1,) -related L-R pairs


# Details of (7,1,) -related L-R pairs

# (7,1,:) Pattern

# (7,1,) -related L-R pairs (Top7 pairs)

```
library("scTensor")
load("reanalysis.RData")
col <- .setColor("many")[18]
scTensor:::.myvisNetwork(g, col)
```

| Rank | Ligand Gene | Receptor Gene | Ligand Expression (Log10(exp + 1)) | Receptor Expression (Log10(exp + 1)) | LR-pair factor value (Percentage) | P-value (Grubbs test) | Q-value (BH method) | L-R Evidence cf. rikenbit/lrbase-workflow | PubMed |
| --- | --- | --- | --- | --- | --- | --- | --- | --- | --- |
| 1 | Vegfa Description: Vegfavascular endothelial growth factor A GO: 1-87 88-173 Reactome: 1-11 UniProtKB: 1-3 RefEx: Vegfa Expression Atlas: Vegfa Single Cell Expression Atlas: Vegfa PanglaoDB: Vegfa MeSH: 1-99 100-198 199-297 298-396 397-495 496-594 595-693 694-792 793-891 892-990 991-1089 1090-1188 1189-1287 1288-1386 1387-1485 1486-1584 1585-1683 1684-1782 1783-1881 1882-1980 1981-2079 2080-2178 2179-2277 2278-2376 2377-2475 2476-2574 2575-2673 2674-2772 2773-2871 2872-2970 2971-3069 3070-3168 3169-3255 | Nrp1 Description: Vegfaneuropilin 1 GO: 1-59 60-118 Reactome: 1-13 UniProtKB: 1 RefEx: Vegfa Expression Atlas: Nrp1 Single Cell Expression Atlas: Nrp1 PanglaoDB: Nrp1 MeSH: 1-95 96-190 191-285 286-380 381-475 476-570 571-665 666-760 761-855 856-950 951-1045 1046-1140 1141-1235 1236-1322 |  |  | 0.017 (28.94%) | 0 | 0 | DLRP | 1 |
| 2 | Fn1 Description: Fn1fibronectin 1 GO: 1-61 Reactome: 1-31 UniProtKB: 1-12 RefEx: Fn1 Expression Atlas: Fn1 Single Cell Expression Atlas: Fn1 PanglaoDB: Fn1 MeSH: 1-99 100-198 199-297 298-396 397-495 496-594 595-693 694-792 793-891 892-990 991-1089 1090-1188 1189-1287 1288-1386 1387-1485 1486-1584 1585-1683 1684-1782 | Itga4 Description: Fn1integrin alpha 4 GO: 1-51 Reactome: 1-7 UniProtKB: 1-3 RefEx: Fn1 Expression Atlas: Itga4 Single Cell Expression Atlas: Itga4 PanglaoDB: Itga4 MeSH: 1-92 93-184 185-276 277-368 369-460 461-552 553-644 645-736 737-828 829-920 921-1005 |  |  | 0.012 (21.081%) | 0.001 | 0.001 | DLRP | 1 |
| 3 | Thbs1 Description: Thbs1thrombospondin 1 GO: 1-84 UniProtKB: 1-2 STRING: 1 RefEx: Thbs1 Expression Atlas: Thbs1 Single Cell Expression Atlas: Thbs1 PanglaoDB: Thbs1 MeSH: 1-100 101-200 201-300 301-400 401-500 501-600 601-700 701-800 801-900 901-1000 1001-1100 1101-1200 1201-1294 | Cd36 Description: Thbs1CD36 molecule GO: 1-63 64-125 Reactome: 1-21 UniProtKB: 1-5 RefEx: Thbs1 Expression Atlas: Cd36 Single Cell Expression Atlas: Cd36 PanglaoDB: Cd36 MeSH: 1-99 100-198 199-297 298-396 397-495 496-594 595-693 694-792 793-891 892-990 991-1089 1090-1188 1189-1287 1288-1386 1387-1485 1486-1581 |  |  | 0.008 (14.508%) | 0.035 | 0.04 | DLRP | 1 |
| 4 | Fn1 Description: Fn1fibronectin 1 GO: 1-61 Reactome: 1-31 UniProtKB: 1-12 RefEx: Fn1 Expression Atlas: Fn1 Single Cell Expression Atlas: Fn1 PanglaoDB: Fn1 MeSH: 1-99 100-198 199-297 298-396 397-495 496-594 595-693 694-792 793-891 892-990 991-1089 1090-1188 1189-1287 1288-1386 1387-1485 1486-1584 1585-1683 1684-1782 | Itga8 Description: Fn1integrin alpha 8 GO: 1-28 Reactome: 1-5 UniProtKB: 1 RefEx: Fn1 Expression Atlas: Itga8 Single Cell Expression Atlas: Itga8 PanglaoDB: Itga8 MeSH: 1-86 87-172 173-258 259-344 345-428 |  |  | 0.004 (7.757%) | 0.026 | 0.03 | DLRP | 1 |
| 5 | Pdgfa Description: Pdgfaplatelet derived growth factor, alpha GO: 1-44 Reactome: 1-17 UniProtKB: 1-3 RefEx: Pdgfa Expression Atlas: Pdgfa Single Cell Expression Atlas: Pdgfa PanglaoDB: Pdgfa MeSH: 1-96 97-192 193-288 289-384 385-480 481-576 577-672 673-768 769-864 865-960 961-1056 1057-1152 1153-1248 1249-1344 1345-1440 1441-1536 1537-1632 1633-1728 1729-1824 1825-1920 1921-2003 | Pdgfra Description: Pdgfaplatelet derived growth factor receptor, alpha polypeptide GO: 1-88 Reactome: 1-11 UniProtKB: 1 RefEx: Pdgfa Expression Atlas: Pdgfra Single Cell Expression Atlas: Pdgfra PanglaoDB: Pdgfra MeSH: 1-98 99-196 197-294 295-392 393-490 491-588 589-686 687-784 785-882 883-980 981-1078 1079-1176 1177-1274 1275-1372 1373-1470 1471-1568 1569-1666 |  |  | 0.004 (7.752%) | 0.015 | 0.019 | DLRP | 1 |
| 6 | Cd24a Description: Cd24aCD24a antigen GO: 1-77 UniProtKB: 1-3 RefEx: Cd24a Expression Atlas: Cd24a Single Cell Expression Atlas: Cd24a PanglaoDB: Cd24a MeSH: 1-97 98-194 195-291 292-388 389-485 486-582 583-679 680-776 777-873 874-970 971-1058 | Selp Description: Cd24aselectin, platelet GO: 1-35 Reactome: 1-5 UniProtKB: 1 RefEx: Cd24a Expression Atlas: Selp Single Cell Expression Atlas: Selp PanglaoDB: Selp MeSH: 1-94 95-188 189-282 283-376 377-470 471-564 565-658 659-752 753-846 847-940 941-1034 1035-1128 1129-1222 1223-1308 |  |  | 0.003 (5.041%) | 0 | 0 | DLRP | 1 |
| 7 | Edn3 Description: Edn3endothelin 3 GO: 1-34 Reactome: 1-7 UniProtKB: 1-2 RefEx: Edn3 Expression Atlas: Edn3 Single Cell Expression Atlas: Edn3 PanglaoDB: Edn3 MeSH: 1-95 96-190 191-285 286-380 381-474 | Ednrb Description: Edn3endothelin receptor type B GO: 1-66 Reactome: 1-7 UniProtKB: 1 RefEx: Edn3 Expression Atlas: Ednrb Single Cell Expression Atlas: Ednrb PanglaoDB: Ednrb MeSH: 1-100 101-200 201-300 301-400 401-500 501-600 601-700 701-800 801-900 901-996 |  |  | 0.003 (4.852%) | 0.008 | 0.011 | DLRP | 1 |

# (7,1,) -related Enrichment Analysis

```
load("reanalysis.RData")
library("plotly")
```

## MeSH-Enrichment Analysis (A : Anatomy)

```
negLogPval <- -log10(out.vecLR[['pattern_7_1']]$Enrich$MeSH_A$Pvalue + 1E-10)
term <- out.vecLR[['pattern_7_1']]$Enrich$MeSH_A$Term
target <- seq_len(min(100, length(negLogPval)))
negLogPval <- negLogPval[target]
term <- term[target]
p <- plot_ly(x=seq_along(negLogPval), y=~negLogPval,
type="bar", color=~negLogPval, text=term,
colors=c("#4b61ba", "gray", "#a87963", "red"))
layout(p, title="MeSH-Enrichment Analysis (A)",
   xaxis=list(title="Term"),
   yaxis=list(title="-Log(P-value)"),
   legend=list(name=""))
```

```
## Warning: textfont.color doesn't (yet) support data arrays

## Warning: textfont.color doesn't (yet) support data arrays
```

## MeSH-Enrichment Analysis (B : Organisms)

```
negLogPval <- -log10(out.vecLR[['pattern_7_1']]$Enrich$MeSH_B$Pvalue + 1E-10)
term <- out.vecLR[['pattern_7_1']]$Enrich$MeSH_B$Term
target <- seq_len(min(100, length(negLogPval)))
negLogPval <- negLogPval[target]
term <- term[target]
p <- plot_ly(x=seq_along(negLogPval), y=~negLogPval,
type="bar", color=~negLogPval, text=term,
colors=c("#4b61ba", "gray", "#a87963", "red"))
layout(p, title="MeSH-Enrichment Analysis (B)",
   xaxis=list(title="Term"),
   yaxis=list(title="-Log(P-value)"),
   legend=list(name=""))
```

```
## Warning: textfont.color doesn't (yet) support data arrays

## Warning: textfont.color doesn't (yet) support data arrays
```

## MeSH-Enrichment Analysis (C : Diseases)

```
negLogPval <- -log10(out.vecLR[['pattern_7_1']]$Enrich$MeSH_C$Pvalue + 1E-10)
term <- out.vecLR[['pattern_7_1']]$Enrich$MeSH_C$Term
target <- seq_len(min(100, length(negLogPval)))
negLogPval <- negLogPval[target]
term <- term[target]
p <- plot_ly(x=seq_along(negLogPval), y=~negLogPval,
type="bar", color=~negLogPval, text=term,
colors=c("#4b61ba", "gray", "#a87963", "red"))
layout(p, title="MeSH-Enrichment Analysis (C)",
   xaxis=list(title="Term"),
   yaxis=list(title="-Log(P-value)"),
   legend=list(name=""))
```

```
## Warning: textfont.color doesn't (yet) support data arrays

## Warning: textfont.color doesn't (yet) support data arrays
```

## MeSH-Enrichment Analysis (D : Drugs)

```
negLogPval <- -log10(out.vecLR[['pattern_7_1']]$Enrich$MeSH_D$Pvalue + 1E-10)
term <- out.vecLR[['pattern_7_1']]$Enrich$MeSH_D$Term
target <- seq_len(min(100, length(negLogPval)))
negLogPval <- negLogPval[target]
term <- term[target]
p <- plot_ly(x=seq_along(negLogPval), y=~negLogPval,
type="bar", color=~negLogPval, text=term,
colors=c("#4b61ba", "gray", "#a87963", "red"))
layout(p, title="MeSH-Enrichment Analysis (D)",
   xaxis=list(title="Term"),
   yaxis=list(title="-Log(P-value)"),
   legend=list(name=""))
```

```
## Warning: textfont.color doesn't (yet) support data arrays

## Warning: textfont.color doesn't (yet) support data arrays
```

## MeSH-Enrichment Analysis (E : Analytical, Diagnostic and Therapeutic Techniques and Equipment)

```
negLogPval <- -log10(out.vecLR[['pattern_7_1']]$Enrich$MeSH_E$Pvalue + 1E-10)
term <- out.vecLR[['pattern_7_1']]$Enrich$MeSH_E$Term
target <- seq_len(min(100, length(negLogPval)))
negLogPval <- negLogPval[target]
term <- term[target]
p <- plot_ly(x=seq_along(negLogPval), y=~negLogPval,
type="bar", color=~negLogPval, text=term,
colors=c("#4b61ba", "gray", "#a87963", "red"))
layout(p, title="MeSH-Enrichment Analysis (E)",
   xaxis=list(title="Term"),
   yaxis=list(title="-Log(P-value)"),
   legend=list(name=""))
```

```
## Warning: textfont.color doesn't (yet) support data arrays

## Warning: textfont.color doesn't (yet) support data arrays
```

## MeSH-Enrichment Analysis (F : Psychiatry and Psychology)

```
negLogPval <- -log10(out.vecLR[['pattern_7_1']]$Enrich$MeSH_F$Pvalue + 1E-10)
term <- out.vecLR[['pattern_7_1']]$Enrich$MeSH_F$Term
target <- seq_len(min(100, length(negLogPval)))
negLogPval <- negLogPval[target]
term <- term[target]
p <- plot_ly(x=seq_along(negLogPval), y=~negLogPval,
type="bar", color=~negLogPval, text=term,
colors=c("#4b61ba", "gray", "#a87963", "red"))
layout(p, title="MeSH-Enrichment Analysis (F)",
   xaxis=list(title="Term"),
   yaxis=list(title="-Log(P-value)"),
   legend=list(name=""))
```

```
## Warning: textfont.color doesn't (yet) support data arrays

## Warning: textfont.color doesn't (yet) support data arrays
```

## MeSH-Enrichment Analysis (G : Phenomena and Processes)

```
negLogPval <- -log10(out.vecLR[['pattern_7_1']]$Enrich$MeSH_G$Pvalue + 1E-10)
term <- out.vecLR[['pattern_7_1']]$Enrich$MeSH_G$Term
target <- seq_len(min(100, length(negLogPval)))
negLogPval <- negLogPval[target]
term <- term[target]
p <- plot_ly(x=seq_along(negLogPval), y=~negLogPval,
type="bar", color=~negLogPval, text=term,
colors=c("#4b61ba", "gray", "#a87963", "red"))
layout(p, title="MeSH-Enrichment Analysis (G)",
   xaxis=list(title="Term"),
   yaxis=list(title="-Log(P-value)"),
   legend=list(name=""))
```

```
## Warning: textfont.color doesn't (yet) support data arrays

## Warning: textfont.color doesn't (yet) support data arrays
```

## MeSH-Enrichment Analysis (H : Disciplines and Occupations)

```
negLogPval <- -log10(out.vecLR[['pattern_7_1']]$Enrich$MeSH_H$Pvalue + 1E-10)
term <- out.vecLR[['pattern_7_1']]$Enrich$MeSH_H$Term
target <- seq_len(min(100, length(negLogPval)))
negLogPval <- negLogPval[target]
term <- term[target]
p <- plot_ly(x=seq_along(negLogPval), y=~negLogPval,
type="bar", color=~negLogPval, text=term,
colors=c("#4b61ba", "gray", "#a87963", "red"))
layout(p, title="MeSH-Enrichment Analysis (H)",
   xaxis=list(title="Term"),
   yaxis=list(title="-Log(P-value)"),
   legend=list(name=""))
```

## MeSH-Enrichment Analysis (I : Anthropology, Education, Sociology and Social Phenomena)

```
negLogPval <- -log10(out.vecLR[['pattern_7_1']]$Enrich$MeSH_I$Pvalue + 1E-10)
term <- out.vecLR[['pattern_7_1']]$Enrich$MeSH_I$Term
target <- seq_len(min(100, length(negLogPval)))
negLogPval <- negLogPval[target]
term <- term[target]
p <- plot_ly(x=seq_along(negLogPval), y=~negLogPval,
type="bar", color=~negLogPval, text=term,
colors=c("#4b61ba", "gray", "#a87963", "red"))
layout(p, title="MeSH-Enrichment Analysis (I)",
   xaxis=list(title="Term"),
   yaxis=list(title="-Log(P-value)"),
   legend=list(name=""))
```

## MeSH-Enrichment Analysis (J : Technology and Food and Beverages)

```
negLogPval <- -log10(out.vecLR[['pattern_7_1']]$Enrich$MeSH_J$Pvalue + 1E-10)
term <- out.vecLR[['pattern_7_1']]$Enrich$MeSH_J$Term
target <- seq_len(min(100, length(negLogPval)))
negLogPval <- negLogPval[target]
term <- term[target]
p <- plot_ly(x=seq_along(negLogPval), y=~negLogPval,
type="bar", color=~negLogPval, text=term,
colors=c("#4b61ba", "gray", "#a87963", "red"))
layout(p, title="MeSH-Enrichment Analysis (J)",
   xaxis=list(title="Term"),
   yaxis=list(title="-Log(P-value)"),
   legend=list(name=""))
```

## MeSH-Enrichment Analysis (L : Information Science)

```
negLogPval <- -log10(out.vecLR[['pattern_7_1']]$Enrich$MeSH_L$Pvalue + 1E-10)
term <- out.vecLR[['pattern_7_1']]$Enrich$MeSH_L$Term
target <- seq_len(min(100, length(negLogPval)))
negLogPval <- negLogPval[target]
term <- term[target]
p <- plot_ly(x=seq_along(negLogPval), y=~negLogPval,
type="bar", color=~negLogPval, text=term,
colors=c("#4b61ba", "gray", "#a87963", "red"))
layout(p, title="MeSH-Enrichment Analysis (L)",
   xaxis=list(title="Term"),
   yaxis=list(title="-Log(P-value)"),
   legend=list(name=""))
```

```
## Warning: textfont.color doesn't (yet) support data arrays

## Warning: textfont.color doesn't (yet) support data arrays
```

## MeSH-Enrichment Analysis (M : Persons)

```
negLogPval <- -log10(out.vecLR[['pattern_7_1']]$Enrich$MeSH_M$Pvalue + 1E-10)
term <- out.vecLR[['pattern_7_1']]$Enrich$MeSH_M$Term
target <- seq_len(min(100, length(negLogPval)))
negLogPval <- negLogPval[target]
term <- term[target]
p <- plot_ly(x=seq_along(negLogPval), y=~negLogPval,
type="bar", color=~negLogPval, text=term,
colors=c("#4b61ba", "gray", "#a87963", "red"))
layout(p, title="MeSH-Enrichment Analysis (M)",
   xaxis=list(title="Term"),
   yaxis=list(title="-Log(P-value)"),
   legend=list(name=""))
```

```
## Warning: textfont.color doesn't (yet) support data arrays

## Warning: textfont.color doesn't (yet) support data arrays
```

## MeSH-Enrichment Analysis (N : Health Care)

```
negLogPval <- -log10(out.vecLR[['pattern_7_1']]$Enrich$MeSH_N$Pvalue + 1E-10)
term <- out.vecLR[['pattern_7_1']]$Enrich$MeSH_N$Term
target <- seq_len(min(100, length(negLogPval)))
negLogPval <- negLogPval[target]
term <- term[target]
p <- plot_ly(x=seq_along(negLogPval), y=~negLogPval,
type="bar", color=~negLogPval, text=term,
colors=c("#4b61ba", "gray", "#a87963", "red"))
layout(p, title="MeSH-Enrichment Analysis (N)",
   xaxis=list(title="Term"),
   yaxis=list(title="-Log(P-value)"),
   legend=list(name=""))
```

```
## Warning: textfont.color doesn't (yet) support data arrays

## Warning: textfont.color doesn't (yet) support data arrays
```

## Reactome Pathway-Enrichment Analysis

```
negLogPval <- -log10(out.vecLR[['pattern_7_1']]$Enrich$Reactome$Pvalue + 1E-10)
term <- out.vecLR[['pattern_7_1']]$Enrich$Reactome$Term
target <- seq_len(min(100, length(negLogPval)))
negLogPval <- negLogPval[target]
term <- term[target]
p <- plot_ly(x=seq_along(negLogPval), y=~negLogPval,
type="bar", color=~negLogPval, text=term,
colors=c("#4b61ba", "gray", "#a87963", "red"))
layout(p, title="Reactome-Enrichment Analysis",
   xaxis=list(title="Term"),
   yaxis=list(title="-Log(P-value)"),
   legend=list(name=""))
```

```
## Warning: textfont.color doesn't (yet) support data arrays

## Warning: textfont.color doesn't (yet) support data arrays
```
