# Supplementary material for: Sctensor detects many-to-many cell–cell interactions from single cell RNA-sequencing data
Source: BMC Bioinformatics. 2023 Nov 7;24:420. doi: 10.1186/s12859-023-05490-y (PMC10631077; doi:10.1186/s12859-023-05490-y)
Supplement: Supplementary file 17 — Additional file 17. HTML report of Uterus. [file 12859_2023_5490_MOESM17_ESM.zip › output/report/Mouse_Uterus/pattern_4_3.html]

Details of (4,3,) -related L-R pairs


# Details of (4,3,) -related L-R pairs

# (4,3,:) Pattern

# (4,3,) -related L-R pairs (Top4 pairs)

```
library("scTensor")
load("reanalysis.RData")
col <- .setColor("many")[20]
scTensor:::.myvisNetwork(g, col)
```

| Rank | Ligand Gene | Receptor Gene | Ligand Expression (Log10(exp + 1)) | Receptor Expression (Log10(exp + 1)) | LR-pair factor value (Percentage) | P-value (Grubbs test) | Q-value (BH method) | L-R Evidence cf. rikenbit/lrbase-workflow | PubMed |
| --- | --- | --- | --- | --- | --- | --- | --- | --- | --- |
| 1 | Fn1 Description: Fn1fibronectin 1 GO: 1-61 Reactome: 1-31 UniProtKB: 1-12 RefEx: Fn1 Expression Atlas: Fn1 Single Cell Expression Atlas: Fn1 PanglaoDB: Fn1 MeSH: 1-99 100-198 199-297 298-396 397-495 496-594 595-693 694-792 793-891 892-990 991-1089 1090-1188 1189-1287 1288-1386 1387-1485 1486-1584 1585-1683 1684-1782 | Itga4 Description: Fn1integrin alpha 4 GO: 1-51 Reactome: 1-7 UniProtKB: 1-3 RefEx: Fn1 Expression Atlas: Itga4 Single Cell Expression Atlas: Itga4 PanglaoDB: Itga4 MeSH: 1-92 93-184 185-276 277-368 369-460 461-552 553-644 645-736 737-828 829-920 921-1005 |  |  | 0.013 (25.375%) | 0 | 0 | DLRP | 1 |
| 2 | Col4a1 Description: Col4a1collagen, type IV, alpha 1 GO: 1-21 Reactome: 1-20 UniProtKB: 1 RefEx: Col4a1 Expression Atlas: Col4a1 Single Cell Expression Atlas: Col4a1 PanglaoDB: Col4a1 MeSH: 1-94 95-188 189-282 283-376 377-470 471-564 565-658 659-752 753-845 | Itga1 Description: Col4a1integrin alpha 1 GO: 1-32 Reactome: 1-4 UniProtKB: 1-2 RefEx: Col4a1 Expression Atlas: Itga1 Single Cell Expression Atlas: Itga1 PanglaoDB: Itga1 MeSH: 1-94 95-188 189-282 283-376 377-470 471-564 565-658 |  |  | 0.013 (24.231%) | 0 | 0 | DLRP | 1 |
| 3 | Npnt Description: Npntnephronectin GO: 1-27 UniProtKB: 1-3 RefEx: Npnt Expression Atlas: Npnt Single Cell Expression Atlas: Npnt PanglaoDB: Npnt MeSH: 1-77 78-154 155-231 232-308 | Itga8 Description: Npntintegrin alpha 8 GO: 1-28 Reactome: 1-5 UniProtKB: 1 RefEx: Npnt Expression Atlas: Itga8 Single Cell Expression Atlas: Itga8 PanglaoDB: Itga8 MeSH: 1-86 87-172 173-258 259-344 345-428 |  |  | 0.009 (16.78%) | 0 | 0 | DLRP | 1 |
| 4 | Cxcl12 Description: Cxcl12chemokine (C-X-C motif) ligand 12 GO: 1-60 Reactome: 1-12 UniProtKB: 1-2 RefEx: Cxcl12 Expression Atlas: Cxcl12 Single Cell Expression Atlas: Cxcl12 PanglaoDB: Cxcl12 MeSH: 1-100 101-200 201-300 301-400 401-500 501-600 601-700 701-800 801-900 901-1000 1001-1100 1101-1200 1201-1300 1301-1400 1401-1500 1501-1600 1601-1700 1701-1800 1801-1900 1901-2000 2001-2100 2101-2200 2201-2295 | Ackr3 Description: Cxcl12atypical chemokine receptor 3 GO: 1-35 Reactome: 1-8 UniProtKB: 1 STRING: 1 2 RefEx: Cxcl12 Expression Atlas: Ackr3 Single Cell Expression Atlas: Ackr3 PanglaoDB: Ackr3 MeSH: 1-100 101-200 201-300 301-400 401-500 501-597 |  |  | 0.005 (9.797%) | 0 | 0 | DLRP | 1 |

# (4,3,) -related Enrichment Analysis

```
load("reanalysis.RData")
library("plotly")
```

## MeSH-Enrichment Analysis (A : Anatomy)

```
negLogPval <- -log10(out.vecLR[['pattern_4_3']]$Enrich$MeSH_A$Pvalue + 1E-10)
term <- out.vecLR[['pattern_4_3']]$Enrich$MeSH_A$Term
target <- seq_len(min(100, length(negLogPval)))
negLogPval <- negLogPval[target]
term <- term[target]
p <- plot_ly(x=seq_along(negLogPval), y=~negLogPval,
type="bar", color=~negLogPval, text=term,
colors=c("#4b61ba", "gray", "#a87963", "red"))
layout(p, title="MeSH-Enrichment Analysis (A)",
   xaxis=list(title="Term"),
   yaxis=list(title="-Log(P-value)"),
   legend=list(name=""))
```

```
## Warning: textfont.color doesn't (yet) support data arrays

## Warning: textfont.color doesn't (yet) support data arrays
```

## MeSH-Enrichment Analysis (B : Organisms)

```
negLogPval <- -log10(out.vecLR[['pattern_4_3']]$Enrich$MeSH_B$Pvalue + 1E-10)
term <- out.vecLR[['pattern_4_3']]$Enrich$MeSH_B$Term
target <- seq_len(min(100, length(negLogPval)))
negLogPval <- negLogPval[target]
term <- term[target]
p <- plot_ly(x=seq_along(negLogPval), y=~negLogPval,
type="bar", color=~negLogPval, text=term,
colors=c("#4b61ba", "gray", "#a87963", "red"))
layout(p, title="MeSH-Enrichment Analysis (B)",
   xaxis=list(title="Term"),
   yaxis=list(title="-Log(P-value)"),
   legend=list(name=""))
```

```
## Warning: textfont.color doesn't (yet) support data arrays

## Warning: textfont.color doesn't (yet) support data arrays
```

## MeSH-Enrichment Analysis (C : Diseases)

```
negLogPval <- -log10(out.vecLR[['pattern_4_3']]$Enrich$MeSH_C$Pvalue + 1E-10)
term <- out.vecLR[['pattern_4_3']]$Enrich$MeSH_C$Term
target <- seq_len(min(100, length(negLogPval)))
negLogPval <- negLogPval[target]
term <- term[target]
p <- plot_ly(x=seq_along(negLogPval), y=~negLogPval,
type="bar", color=~negLogPval, text=term,
colors=c("#4b61ba", "gray", "#a87963", "red"))
layout(p, title="MeSH-Enrichment Analysis (C)",
   xaxis=list(title="Term"),
   yaxis=list(title="-Log(P-value)"),
   legend=list(name=""))
```

```
## Warning: textfont.color doesn't (yet) support data arrays

## Warning: textfont.color doesn't (yet) support data arrays
```

## MeSH-Enrichment Analysis (D : Drugs)

```
negLogPval <- -log10(out.vecLR[['pattern_4_3']]$Enrich$MeSH_D$Pvalue + 1E-10)
term <- out.vecLR[['pattern_4_3']]$Enrich$MeSH_D$Term
target <- seq_len(min(100, length(negLogPval)))
negLogPval <- negLogPval[target]
term <- term[target]
p <- plot_ly(x=seq_along(negLogPval), y=~negLogPval,
type="bar", color=~negLogPval, text=term,
colors=c("#4b61ba", "gray", "#a87963", "red"))
layout(p, title="MeSH-Enrichment Analysis (D)",
   xaxis=list(title="Term"),
   yaxis=list(title="-Log(P-value)"),
   legend=list(name=""))
```

```
## Warning: textfont.color doesn't (yet) support data arrays

## Warning: textfont.color doesn't (yet) support data arrays
```

## MeSH-Enrichment Analysis (E : Analytical, Diagnostic and Therapeutic Techniques and Equipment)

```
negLogPval <- -log10(out.vecLR[['pattern_4_3']]$Enrich$MeSH_E$Pvalue + 1E-10)
term <- out.vecLR[['pattern_4_3']]$Enrich$MeSH_E$Term
target <- seq_len(min(100, length(negLogPval)))
negLogPval <- negLogPval[target]
term <- term[target]
p <- plot_ly(x=seq_along(negLogPval), y=~negLogPval,
type="bar", color=~negLogPval, text=term,
colors=c("#4b61ba", "gray", "#a87963", "red"))
layout(p, title="MeSH-Enrichment Analysis (E)",
   xaxis=list(title="Term"),
   yaxis=list(title="-Log(P-value)"),
   legend=list(name=""))
```

```
## Warning: textfont.color doesn't (yet) support data arrays

## Warning: textfont.color doesn't (yet) support data arrays
```

## MeSH-Enrichment Analysis (G : Phenomena and Processes)

```
negLogPval <- -log10(out.vecLR[['pattern_4_3']]$Enrich$MeSH_G$Pvalue + 1E-10)
term <- out.vecLR[['pattern_4_3']]$Enrich$MeSH_G$Term
target <- seq_len(min(100, length(negLogPval)))
negLogPval <- negLogPval[target]
term <- term[target]
p <- plot_ly(x=seq_along(negLogPval), y=~negLogPval,
type="bar", color=~negLogPval, text=term,
colors=c("#4b61ba", "gray", "#a87963", "red"))
layout(p, title="MeSH-Enrichment Analysis (G)",
   xaxis=list(title="Term"),
   yaxis=list(title="-Log(P-value)"),
   legend=list(name=""))
```

```
## Warning: textfont.color doesn't (yet) support data arrays

## Warning: textfont.color doesn't (yet) support data arrays
```

## MeSH-Enrichment Analysis (L : Information Science)

```
negLogPval <- -log10(out.vecLR[['pattern_4_3']]$Enrich$MeSH_L$Pvalue + 1E-10)
term <- out.vecLR[['pattern_4_3']]$Enrich$MeSH_L$Term
target <- seq_len(min(100, length(negLogPval)))
negLogPval <- negLogPval[target]
term <- term[target]
p <- plot_ly(x=seq_along(negLogPval), y=~negLogPval,
type="bar", color=~negLogPval, text=term,
colors=c("#4b61ba", "gray", "#a87963", "red"))
layout(p, title="MeSH-Enrichment Analysis (L)",
   xaxis=list(title="Term"),
   yaxis=list(title="-Log(P-value)"),
   legend=list(name=""))
```

## MeSH-Enrichment Analysis (N : Health Care)

```
negLogPval <- -log10(out.vecLR[['pattern_4_3']]$Enrich$MeSH_N$Pvalue + 1E-10)
term <- out.vecLR[['pattern_4_3']]$Enrich$MeSH_N$Term
target <- seq_len(min(100, length(negLogPval)))
negLogPval <- negLogPval[target]
term <- term[target]
p <- plot_ly(x=seq_along(negLogPval), y=~negLogPval,
type="bar", color=~negLogPval, text=term,
colors=c("#4b61ba", "gray", "#a87963", "red"))
layout(p, title="MeSH-Enrichment Analysis (N)",
   xaxis=list(title="Term"),
   yaxis=list(title="-Log(P-value)"),
   legend=list(name=""))
```

## Reactome Pathway-Enrichment Analysis

```
negLogPval <- -log10(out.vecLR[['pattern_4_3']]$Enrich$Reactome$Pvalue + 1E-10)
term <- out.vecLR[['pattern_4_3']]$Enrich$Reactome$Term
target <- seq_len(min(100, length(negLogPval)))
negLogPval <- negLogPval[target]
term <- term[target]
p <- plot_ly(x=seq_along(negLogPval), y=~negLogPval,
type="bar", color=~negLogPval, text=term,
colors=c("#4b61ba", "gray", "#a87963", "red"))
layout(p, title="Reactome-Enrichment Analysis",
   xaxis=list(title="Term"),
   yaxis=list(title="-Log(P-value)"),
   legend=list(name=""))
```

```
## Warning: textfont.color doesn't (yet) support data arrays

## Warning: textfont.color doesn't (yet) support data arrays
```
