# Supplementary material for: Sctensor detects many-to-many cell–cell interactions from single cell RNA-sequencing data
Source: BMC Bioinformatics. 2023 Nov 7;24:420. doi: 10.1186/s12859-023-05490-y (PMC10631077; doi:10.1186/s12859-023-05490-y)
Supplement: Supplementary file 17 — Additional file 17. HTML report of Uterus. [file 12859_2023_5490_MOESM17_ESM.zip › output/report/Mouse_Uterus/pattern_1_4.html]

Details of (1,4,) -related L-R pairs


# Details of (1,4,) -related L-R pairs

# (1,4,:) Pattern

# (1,4,) -related L-R pairs (Top13 pairs)

```
library("scTensor")
load("reanalysis.RData")
col <- .setColor("many")[24]
scTensor:::.myvisNetwork(g, col)
```

| Rank | Ligand Gene | Receptor Gene | Ligand Expression (Log10(exp + 1)) | Receptor Expression (Log10(exp + 1)) | LR-pair factor value (Percentage) | P-value (Grubbs test) | Q-value (BH method) | L-R Evidence cf. rikenbit/lrbase-workflow | PubMed |
| --- | --- | --- | --- | --- | --- | --- | --- | --- | --- |
| 1 | Col4a1 Description: Col4a1collagen, type IV, alpha 1 GO: 1-21 Reactome: 1-20 UniProtKB: 1 RefEx: Col4a1 Expression Atlas: Col4a1 Single Cell Expression Atlas: Col4a1 PanglaoDB: Col4a1 MeSH: 1-94 95-188 189-282 283-376 377-470 471-564 565-658 659-752 753-845 | Itga1 Description: Col4a1integrin alpha 1 GO: 1-32 Reactome: 1-4 UniProtKB: 1-2 RefEx: Col4a1 Expression Atlas: Itga1 Single Cell Expression Atlas: Itga1 PanglaoDB: Itga1 MeSH: 1-94 95-188 189-282 283-376 377-470 471-564 565-658 |  |  | 0.013 (32.559%) | 0 | 0 | DLRP | 1 |
| 2 | Kitl Description: Kitlkit ligand GO: 1-32 Reactome: 1-11 UniProtKB: 1 RefEx: Kitl Expression Atlas: Kitl Single Cell Expression Atlas: Kitl PanglaoDB: Kitl MeSH: 1-97 98-194 195-291 292-388 389-485 486-582 583-679 680-776 777-873 874-970 971-1067 1068-1164 1165-1261 1262-1358 1359-1455 1456-1544 | Kit Description: KitlKIT proto-oncogene receptor tyrosine kinase GO: 1-51 52-101 Reactome: 1-11 UniProtKB: 1 RefEx: Kitl Expression Atlas: Kit Single Cell Expression Atlas: Kit PanglaoDB: Kit MeSH: 1-99 100-198 199-297 298-396 397-495 496-594 595-693 694-792 793-891 892-990 991-1089 1090-1188 1189-1287 1288-1386 1387-1485 1486-1584 1585-1683 1684-1782 1783-1881 1882-1980 1981-2079 2080-2178 2179-2277 2278-2376 2377-2475 2476-2574 2575-2673 2674-2772 2773-2871 2872-2970 2971-3053 |  |  | 0.004 (9.011%) | 0 | 0 | DLRP | 1 |
| 3 | Cxcl12 Description: Cxcl12chemokine (C-X-C motif) ligand 12 GO: 1-60 Reactome: 1-12 UniProtKB: 1-2 RefEx: Cxcl12 Expression Atlas: Cxcl12 Single Cell Expression Atlas: Cxcl12 PanglaoDB: Cxcl12 MeSH: 1-100 101-200 201-300 301-400 401-500 501-600 601-700 701-800 801-900 901-1000 1001-1100 1101-1200 1201-1300 1301-1400 1401-1500 1501-1600 1601-1700 1701-1800 1801-1900 1901-2000 2001-2100 2101-2200 2201-2295 | Cxcr3 Description: Cxcl12chemokine (C-X-C motif) receptor 3 GO: 1-36 Reactome: 1-8 UniProtKB: 1 STRING: 1 RefEx: Cxcl12 Expression Atlas: Cxcr3 Single Cell Expression Atlas: Cxcr3 PanglaoDB: Cxcr3 MeSH: 1-96 97-192 193-288 289-384 385-480 481-576 577-672 673-768 769-864 865-960 961-1056 1057-1152 1153-1248 1249-1342 |  |  | 0.003 (8.441%) | 0 | 0 | DLRP | 1 |
| 4 | Ccl11 Description: Ccl11chemokine (C-C motif) ligand 11 GO: 1-31 UniProtKB: 1 STRING: 1 RefEx: Ccl11 Expression Atlas: Ccl11 Single Cell Expression Atlas: Ccl11 PanglaoDB: Ccl11 MeSH: 1-94 95-188 189-282 283-376 377-470 471-564 565-658 659-752 753-846 847-940 941-1034 1035-1125 | Ccr2 Description: Ccl11chemokine (C-C motif) receptor 2 GO: 1-69 UniProtKB: 1-3 RefEx: Ccl11 Expression Atlas: Ccr2 Single Cell Expression Atlas: Ccr2 PanglaoDB: Ccr2 MeSH: 1-98 99-196 197-294 295-392 393-490 491-588 589-686 687-784 785-882 883-980 981-1078 1079-1176 1177-1274 1275-1372 1373-1470 1471-1568 1569-1666 1667-1764 1765-1862 1863-1960 1961-2058 2059-2156 2157-2254 2255-2337 |  |  | 0.003 (6.719%) | 0 | 0 | DLRP | 1 |
| 5 | Icam2 Description: Icam2intercellular adhesion molecule 2 GO: 1-13 Reactome: 1-8 UniProtKB: 1 RefEx: Icam2 Expression Atlas: Icam2 Single Cell Expression Atlas: Icam2 PanglaoDB: Icam2 MeSH: 1-86 87-172 173-258 259-344 345-430 | Itgb2 Description: Icam2integrin beta 2 GO: 1-54 Reactome: 1-11 UniProtKB: 1-2 RefEx: Icam2 Expression Atlas: Itgb2 Single Cell Expression Atlas: Itgb2 PanglaoDB: Itgb2 MeSH: 1-98 99-196 197-294 295-392 393-490 491-588 589-686 687-784 785-882 883-980 981-1078 1079-1176 1177-1274 1275-1359 |  |  | 0.003 (6.411%) | 0 | 0 | DLRP | 1 |
| 6 | Cd34 Description: Cd34CD34 antigen GO: 1-43 Reactome: 1-3 UniProtKB: 1-3 RefEx: Cd34 Expression Atlas: Cd34 Single Cell Expression Atlas: Cd34 PanglaoDB: Cd34 MeSH: 1-98 99-196 197-294 295-392 393-490 491-588 589-686 687-784 785-882 883-974 | Sell Description: Cd34selectin, lymphocyte GO: 1-21 Reactome: 1-7 UniProtKB: 1-4 RefEx: Cd34 Expression Atlas: Sell Single Cell Expression Atlas: Sell PanglaoDB: Sell MeSH: 1-94 95-188 189-282 283-376 377-470 471-564 565-658 659-752 753-846 847-939 |  |  | 0.002 (5.963%) | 0 | 0 | DLRP | 1 |
| 7 | Fn1 Description: Fn1fibronectin 1 GO: 1-61 Reactome: 1-31 UniProtKB: 1-12 RefEx: Fn1 Expression Atlas: Fn1 Single Cell Expression Atlas: Fn1 PanglaoDB: Fn1 MeSH: 1-99 100-198 199-297 298-396 397-495 496-594 595-693 694-792 793-891 892-990 991-1089 1090-1188 1189-1287 1288-1386 1387-1485 1486-1584 1585-1683 1684-1782 | Itga4 Description: Fn1integrin alpha 4 GO: 1-51 Reactome: 1-7 UniProtKB: 1-3 RefEx: Fn1 Expression Atlas: Itga4 Single Cell Expression Atlas: Itga4 PanglaoDB: Itga4 MeSH: 1-92 93-184 185-276 277-368 369-460 461-552 553-644 645-736 737-828 829-920 921-1005 |  |  | 0.002 (5.462%) | 0.003 | 0.003 | DLRP | 1 |
| 8 | Cdh1 Description: Cdh1cadherin 1 GO: 1-84 Reactome: 1-17 UniProtKB: 1-2 RefEx: Cdh1 Expression Atlas: Cdh1 Single Cell Expression Atlas: Cdh1 PanglaoDB: Cdh1 MeSH: 1-99 100-198 199-297 298-396 397-495 496-594 595-693 694-792 793-891 892-990 991-1089 1090-1188 1189-1287 1288-1386 1387-1485 1486-1584 1585-1683 1684-1782 1783-1881 1882-1980 1981-2079 2080-2178 2179-2277 2278-2376 2377-2461 | Itgb7 Description: Cdh1integrin beta 7 GO: 1-22 Reactome: 1-5 UniProtKB: 1 RefEx: Cdh1 Expression Atlas: Itgb7 Single Cell Expression Atlas: Itgb7 PanglaoDB: Itgb7 MeSH: 1-98 99-196 197-294 295-392 393-490 491-587 |  |  | 0.002 (4.135%) | 0 | 0 | DLRP | 1 |
| 9 | Ccl21b Description: Ccl21bchemokine (C-C motif) ligand 21B (leucine) GO: 1-44 Reactome: 1-8 UniProtKB: 1-2 STRING: 1 RefEx: Ccl21b Expression Atlas: Ccl21b Single Cell Expression Atlas: Ccl21b PanglaoDB: Ccl21b MeSH: 1-88 89-176 177-264 265-350 | Ccr7 Description: Ccl21bchemokine (C-C motif) receptor 7 GO: 1-65 Reactome: 1-8 UniProtKB: 1 STRING: 1 RefEx: Ccl21b Expression Atlas: Ccr7 Single Cell Expression Atlas: Ccr7 PanglaoDB: Ccr7 MeSH: 1-96 97-192 193-288 289-384 385-480 481-576 577-672 673-768 769-864 865-960 961-1056 1057-1152 1153-1248 1249-1337 |  |  | 0.002 (4.096%) | 0 | 0 | DLRP | 1 |
| 10 | Igf2 Description: Igf2insulin-like growth factor 2 GO: 1-52 Reactome: 1-12 UniProtKB: 1 RefEx: Igf2 Expression Atlas: Igf2 Single Cell Expression Atlas: Igf2 PanglaoDB: Igf2 MeSH: 1-98 99-196 197-294 295-392 393-490 491-588 589-686 687-784 785-882 883-980 981-1078 1079-1176 1177-1274 1275-1372 1373-1470 1471-1553 | Igf1r Description: Igf2insulin-like growth factor I receptor GO: 1-97 Reactome: 1-9 UniProtKB: 1-3 RefEx: Igf2 Expression Atlas: Igf1r Single Cell Expression Atlas: Igf1r PanglaoDB: Igf1r MeSH: 1-100 101-200 201-300 301-400 401-500 501-600 601-700 701-800 801-900 901-1000 1001-1100 1101-1200 1201-1300 1301-1400 1401-1500 1501-1600 1601-1688 |  |  | 0.001 (3.211%) | 0 | 0 | DLRP | 1 |
| 11 | Icam2 Description: Icam2intercellular adhesion molecule 2 GO: 1-13 Reactome: 1-8 UniProtKB: 1 RefEx: Icam2 Expression Atlas: Icam2 Single Cell Expression Atlas: Icam2 PanglaoDB: Icam2 MeSH: 1-86 87-172 173-258 259-344 345-430 | Itgal Description: Icam2integrin alpha L GO: 1-29 UniProtKB: 1-4 RefEx: Icam2 Expression Atlas: Itgal Single Cell Expression Atlas: Itgal PanglaoDB: Itgal MeSH: 1-92 93-184 185-276 277-368 369-460 461-552 553-644 645-736 737-828 829-916 |  |  | 0.001 (2.812%) | 0 | 0 | DLRP | 1 |
| 12 | Igf1 Description: Igf1insulin-like growth factor 1 GO: 1-70 71-139 Reactome: 1-14 UniProtKB: 1-6 RefEx: Igf1 Expression Atlas: Igf1 Single Cell Expression Atlas: Igf1 PanglaoDB: Igf1 MeSH: 1-99 100-198 199-297 298-396 397-495 496-594 595-693 694-792 793-891 892-990 991-1089 1090-1188 1189-1287 1288-1386 1387-1485 1486-1584 1585-1683 1684-1782 1783-1881 1882-1980 1981-2079 2080-2178 2179-2277 2278-2376 2377-2475 2476-2566 | Igf1r Description: Igf1insulin-like growth factor I receptor GO: 1-97 Reactome: 1-9 UniProtKB: 1-3 RefEx: Igf1 Expression Atlas: Igf1r Single Cell Expression Atlas: Igf1r PanglaoDB: Igf1r MeSH: 1-100 101-200 201-300 301-400 401-500 501-600 601-700 701-800 801-900 901-1000 1001-1100 1101-1200 1201-1300 1301-1400 1401-1500 1501-1600 1601-1688 |  |  | 0.001 (2.25%) | 0 | 0 | DLRP | 1 |
| 13 | Ccl11 Description: Ccl11chemokine (C-C motif) ligand 11 GO: 1-31 UniProtKB: 1 STRING: 1 RefEx: Ccl11 Expression Atlas: Ccl11 Single Cell Expression Atlas: Ccl11 PanglaoDB: Ccl11 MeSH: 1-94 95-188 189-282 283-376 377-470 471-564 565-658 659-752 753-846 847-940 941-1034 1035-1125 | Cxcr3 Description: Ccl11chemokine (C-X-C motif) receptor 3 GO: 1-36 Reactome: 1-8 UniProtKB: 1 STRING: 1 RefEx: Ccl11 Expression Atlas: Cxcr3 Single Cell Expression Atlas: Cxcr3 PanglaoDB: Cxcr3 MeSH: 1-96 97-192 193-288 289-384 385-480 481-576 577-672 673-768 769-864 865-960 961-1056 1057-1152 1153-1248 1249-1342 |  |  | 0.001 (1.895%) | 0 | 0 | DLRP | 1 |

# (1,4,) -related Enrichment Analysis

```
load("reanalysis.RData")
library("plotly")
```

## MeSH-Enrichment Analysis (A : Anatomy)

```
negLogPval <- -log10(out.vecLR[['pattern_1_4']]$Enrich$MeSH_A$Pvalue + 1E-10)
term <- out.vecLR[['pattern_1_4']]$Enrich$MeSH_A$Term
target <- seq_len(min(100, length(negLogPval)))
negLogPval <- negLogPval[target]
term <- term[target]
p <- plot_ly(x=seq_along(negLogPval), y=~negLogPval,
type="bar", color=~negLogPval, text=term,
colors=c("#4b61ba", "gray", "#a87963", "red"))
layout(p, title="MeSH-Enrichment Analysis (A)",
   xaxis=list(title="Term"),
   yaxis=list(title="-Log(P-value)"),
   legend=list(name=""))
```

```
## Warning: textfont.color doesn't (yet) support data arrays

## Warning: textfont.color doesn't (yet) support data arrays
```

## MeSH-Enrichment Analysis (B : Organisms)

```
negLogPval <- -log10(out.vecLR[['pattern_1_4']]$Enrich$MeSH_B$Pvalue + 1E-10)
term <- out.vecLR[['pattern_1_4']]$Enrich$MeSH_B$Term
target <- seq_len(min(100, length(negLogPval)))
negLogPval <- negLogPval[target]
term <- term[target]
p <- plot_ly(x=seq_along(negLogPval), y=~negLogPval,
type="bar", color=~negLogPval, text=term,
colors=c("#4b61ba", "gray", "#a87963", "red"))
layout(p, title="MeSH-Enrichment Analysis (B)",
   xaxis=list(title="Term"),
   yaxis=list(title="-Log(P-value)"),
   legend=list(name=""))
```

```
## Warning: textfont.color doesn't (yet) support data arrays

## Warning: textfont.color doesn't (yet) support data arrays
```

## MeSH-Enrichment Analysis (C : Diseases)

```
negLogPval <- -log10(out.vecLR[['pattern_1_4']]$Enrich$MeSH_C$Pvalue + 1E-10)
term <- out.vecLR[['pattern_1_4']]$Enrich$MeSH_C$Term
target <- seq_len(min(100, length(negLogPval)))
negLogPval <- negLogPval[target]
term <- term[target]
p <- plot_ly(x=seq_along(negLogPval), y=~negLogPval,
type="bar", color=~negLogPval, text=term,
colors=c("#4b61ba", "gray", "#a87963", "red"))
layout(p, title="MeSH-Enrichment Analysis (C)",
   xaxis=list(title="Term"),
   yaxis=list(title="-Log(P-value)"),
   legend=list(name=""))
```

```
## Warning: textfont.color doesn't (yet) support data arrays

## Warning: textfont.color doesn't (yet) support data arrays
```

## MeSH-Enrichment Analysis (D : Drugs)

```
negLogPval <- -log10(out.vecLR[['pattern_1_4']]$Enrich$MeSH_D$Pvalue + 1E-10)
term <- out.vecLR[['pattern_1_4']]$Enrich$MeSH_D$Term
target <- seq_len(min(100, length(negLogPval)))
negLogPval <- negLogPval[target]
term <- term[target]
p <- plot_ly(x=seq_along(negLogPval), y=~negLogPval,
type="bar", color=~negLogPval, text=term,
colors=c("#4b61ba", "gray", "#a87963", "red"))
layout(p, title="MeSH-Enrichment Analysis (D)",
   xaxis=list(title="Term"),
   yaxis=list(title="-Log(P-value)"),
   legend=list(name=""))
```

```
## Warning: textfont.color doesn't (yet) support data arrays

## Warning: textfont.color doesn't (yet) support data arrays
```

## MeSH-Enrichment Analysis (E : Analytical, Diagnostic and Therapeutic Techniques and Equipment)

```
negLogPval <- -log10(out.vecLR[['pattern_1_4']]$Enrich$MeSH_E$Pvalue + 1E-10)
term <- out.vecLR[['pattern_1_4']]$Enrich$MeSH_E$Term
target <- seq_len(min(100, length(negLogPval)))
negLogPval <- negLogPval[target]
term <- term[target]
p <- plot_ly(x=seq_along(negLogPval), y=~negLogPval,
type="bar", color=~negLogPval, text=term,
colors=c("#4b61ba", "gray", "#a87963", "red"))
layout(p, title="MeSH-Enrichment Analysis (E)",
   xaxis=list(title="Term"),
   yaxis=list(title="-Log(P-value)"),
   legend=list(name=""))
```

```
## Warning: textfont.color doesn't (yet) support data arrays

## Warning: textfont.color doesn't (yet) support data arrays
```

## MeSH-Enrichment Analysis (F : Psychiatry and Psychology)

```
negLogPval <- -log10(out.vecLR[['pattern_1_4']]$Enrich$MeSH_F$Pvalue + 1E-10)
term <- out.vecLR[['pattern_1_4']]$Enrich$MeSH_F$Term
target <- seq_len(min(100, length(negLogPval)))
negLogPval <- negLogPval[target]
term <- term[target]
p <- plot_ly(x=seq_along(negLogPval), y=~negLogPval,
type="bar", color=~negLogPval, text=term,
colors=c("#4b61ba", "gray", "#a87963", "red"))
layout(p, title="MeSH-Enrichment Analysis (F)",
   xaxis=list(title="Term"),
   yaxis=list(title="-Log(P-value)"),
   legend=list(name=""))
```

```
## Warning: textfont.color doesn't (yet) support data arrays

## Warning: textfont.color doesn't (yet) support data arrays
```

## MeSH-Enrichment Analysis (G : Phenomena and Processes)

```
negLogPval <- -log10(out.vecLR[['pattern_1_4']]$Enrich$MeSH_G$Pvalue + 1E-10)
term <- out.vecLR[['pattern_1_4']]$Enrich$MeSH_G$Term
target <- seq_len(min(100, length(negLogPval)))
negLogPval <- negLogPval[target]
term <- term[target]
p <- plot_ly(x=seq_along(negLogPval), y=~negLogPval,
type="bar", color=~negLogPval, text=term,
colors=c("#4b61ba", "gray", "#a87963", "red"))
layout(p, title="MeSH-Enrichment Analysis (G)",
   xaxis=list(title="Term"),
   yaxis=list(title="-Log(P-value)"),
   legend=list(name=""))
```

```
## Warning: textfont.color doesn't (yet) support data arrays

## Warning: textfont.color doesn't (yet) support data arrays
```

## MeSH-Enrichment Analysis (H : Disciplines and Occupations)

```
negLogPval <- -log10(out.vecLR[['pattern_1_4']]$Enrich$MeSH_H$Pvalue + 1E-10)
term <- out.vecLR[['pattern_1_4']]$Enrich$MeSH_H$Term
target <- seq_len(min(100, length(negLogPval)))
negLogPval <- negLogPval[target]
term <- term[target]
p <- plot_ly(x=seq_along(negLogPval), y=~negLogPval,
type="bar", color=~negLogPval, text=term,
colors=c("#4b61ba", "gray", "#a87963", "red"))
layout(p, title="MeSH-Enrichment Analysis (H)",
   xaxis=list(title="Term"),
   yaxis=list(title="-Log(P-value)"),
   legend=list(name=""))
```

```
## Warning: textfont.color doesn't (yet) support data arrays

## Warning: textfont.color doesn't (yet) support data arrays
```

## MeSH-Enrichment Analysis (J : Technology and Food and Beverages)

```
negLogPval <- -log10(out.vecLR[['pattern_1_4']]$Enrich$MeSH_J$Pvalue + 1E-10)
term <- out.vecLR[['pattern_1_4']]$Enrich$MeSH_J$Term
target <- seq_len(min(100, length(negLogPval)))
negLogPval <- negLogPval[target]
term <- term[target]
p <- plot_ly(x=seq_along(negLogPval), y=~negLogPval,
type="bar", color=~negLogPval, text=term,
colors=c("#4b61ba", "gray", "#a87963", "red"))
layout(p, title="MeSH-Enrichment Analysis (J)",
   xaxis=list(title="Term"),
   yaxis=list(title="-Log(P-value)"),
   legend=list(name=""))
```

```
## Warning: textfont.color doesn't (yet) support data arrays

## Warning: textfont.color doesn't (yet) support data arrays
```

## MeSH-Enrichment Analysis (L : Information Science)

```
negLogPval <- -log10(out.vecLR[['pattern_1_4']]$Enrich$MeSH_L$Pvalue + 1E-10)
term <- out.vecLR[['pattern_1_4']]$Enrich$MeSH_L$Term
target <- seq_len(min(100, length(negLogPval)))
negLogPval <- negLogPval[target]
term <- term[target]
p <- plot_ly(x=seq_along(negLogPval), y=~negLogPval,
type="bar", color=~negLogPval, text=term,
colors=c("#4b61ba", "gray", "#a87963", "red"))
layout(p, title="MeSH-Enrichment Analysis (L)",
   xaxis=list(title="Term"),
   yaxis=list(title="-Log(P-value)"),
   legend=list(name=""))
```

```
## Warning: textfont.color doesn't (yet) support data arrays

## Warning: textfont.color doesn't (yet) support data arrays
```

## MeSH-Enrichment Analysis (M : Persons)

```
negLogPval <- -log10(out.vecLR[['pattern_1_4']]$Enrich$MeSH_M$Pvalue + 1E-10)
term <- out.vecLR[['pattern_1_4']]$Enrich$MeSH_M$Term
target <- seq_len(min(100, length(negLogPval)))
negLogPval <- negLogPval[target]
term <- term[target]
p <- plot_ly(x=seq_along(negLogPval), y=~negLogPval,
type="bar", color=~negLogPval, text=term,
colors=c("#4b61ba", "gray", "#a87963", "red"))
layout(p, title="MeSH-Enrichment Analysis (M)",
   xaxis=list(title="Term"),
   yaxis=list(title="-Log(P-value)"),
   legend=list(name=""))
```

```
## Warning: textfont.color doesn't (yet) support data arrays

## Warning: textfont.color doesn't (yet) support data arrays
```

## MeSH-Enrichment Analysis (N : Health Care)

```
negLogPval <- -log10(out.vecLR[['pattern_1_4']]$Enrich$MeSH_N$Pvalue + 1E-10)
term <- out.vecLR[['pattern_1_4']]$Enrich$MeSH_N$Term
target <- seq_len(min(100, length(negLogPval)))
negLogPval <- negLogPval[target]
term <- term[target]
p <- plot_ly(x=seq_along(negLogPval), y=~negLogPval,
type="bar", color=~negLogPval, text=term,
colors=c("#4b61ba", "gray", "#a87963", "red"))
layout(p, title="MeSH-Enrichment Analysis (N)",
   xaxis=list(title="Term"),
   yaxis=list(title="-Log(P-value)"),
   legend=list(name=""))
```

```
## Warning: textfont.color doesn't (yet) support data arrays

## Warning: textfont.color doesn't (yet) support data arrays
```

## Reactome Pathway-Enrichment Analysis

```
negLogPval <- -log10(out.vecLR[['pattern_1_4']]$Enrich$Reactome$Pvalue + 1E-10)
term <- out.vecLR[['pattern_1_4']]$Enrich$Reactome$Term
target <- seq_len(min(100, length(negLogPval)))
negLogPval <- negLogPval[target]
term <- term[target]
p <- plot_ly(x=seq_along(negLogPval), y=~negLogPval,
type="bar", color=~negLogPval, text=term,
colors=c("#4b61ba", "gray", "#a87963", "red"))
layout(p, title="Reactome-Enrichment Analysis",
   xaxis=list(title="Term"),
   yaxis=list(title="-Log(P-value)"),
   legend=list(name=""))
```

```
## Warning: textfont.color doesn't (yet) support data arrays

## Warning: textfont.color doesn't (yet) support data arrays
```
