# Supplementary material for: Sctensor detects many-to-many cell–cell interactions from single cell RNA-sequencing data
Source: BMC Bioinformatics. 2023 Nov 7;24:420. doi: 10.1186/s12859-023-05490-y (PMC10631077; doi:10.1186/s12859-023-05490-y)
Supplement: Supplementary file 18 — Additional file 18. HTML report of VisualCortex. [file 12859_2023_5490_MOESM18_ESM.zip › output/report/Mouse_VisualCortex/ligand.html]

Details of Ligand Gene-centric Overview (selected)


# Details of Ligand Gene-centric Overview (selected)

```
library("scTensor")
load("reanalysis.RData")
scTensor:::.myvisNetwork(g)
```

| Rank | No. of related pairs | Ligand Gene | Receptor Genes | Related CCIs |
| --- | --- | --- | --- | --- |
| 1 | 17 | Efnb3 | Epha4  Ephb1  Ephb2  Ephb6 | See the details of (3,4,)-related L-R patterns See the details of (5,4,)-related L-R patterns See the details of (3,5,)-related L-R patterns See the details of (3,1,)-related L-R patterns See the details of (3,3,)-related L-R patterns See the details of (5,3,)-related L-R patterns See the details of (4,4,)-related L-R patterns See the details of (4,3,)-related L-R patterns See the details of (3,7,)-related L-R patterns See the details of (3,2,)-related L-R patterns |
| 3.5 | 10 | Vegfa | Flt1  Kdr | See the details of (6,1,)-related L-R patterns See the details of (4,1,)-related L-R patterns See the details of (5,1,)-related L-R patterns See the details of (2,1,)-related L-R patterns See the details of (4,6,)-related L-R patterns See the details of (5,6,)-related L-R patterns |
| 3.5 | 10 | Apoe | Ldlr | See the details of (6,1,)-related L-R patterns See the details of (1,1,)-related L-R patterns See the details of (3,5,)-related L-R patterns See the details of (6,6,)-related L-R patterns See the details of (6,4,)-related L-R patterns See the details of (6,3,)-related L-R patterns See the details of (6,2,)-related L-R patterns See the details of (1,4,)-related L-R patterns See the details of (3,6,)-related L-R patterns See the details of (3,2,)-related L-R patterns |
| 3.5 | 10 | Efna3 | Epha4  Epha5  Ephb1  Epha2 | See the details of (5,4,)-related L-R patterns See the details of (5,3,)-related L-R patterns See the details of (4,4,)-related L-R patterns See the details of (4,3,)-related L-R patterns See the details of (2,4,)-related L-R patterns See the details of (2,7,)-related L-R patterns See the details of (5,6,)-related L-R patterns |
| 3.5 | 10 | Tgfb2 | Tgfbr1  Tgfbr2  Tgfbr3 | See the details of (1,2,)-related L-R patterns See the details of (1,1,)-related L-R patterns See the details of (2,1,)-related L-R patterns See the details of (6,2,)-related L-R patterns See the details of (4,2,)-related L-R patterns |
| 6 | 8 | Efna5 | Epha4  Epha5  Epha2 | See the details of (5,4,)-related L-R patterns See the details of (4,4,)-related L-R patterns See the details of (2,4,)-related L-R patterns See the details of (2,7,)-related L-R patterns See the details of (5,6,)-related L-R patterns |
| 7 | 7 | Nlgn3 | Nrxn3 | See the details of (5,4,)-related L-R patterns See the details of (6,7,)-related L-R patterns See the details of (4,4,)-related L-R patterns See the details of (6,4,)-related L-R patterns See the details of (4,7,)-related L-R patterns See the details of (2,4,)-related L-R patterns See the details of (2,7,)-related L-R patterns |
| 8 | 6 | Efnb2 | Epha4  Ephb1  Ephb6 | See the details of (4,4,)-related L-R patterns See the details of (6,4,)-related L-R patterns See the details of (4,3,)-related L-R patterns See the details of (2,4,)-related L-R patterns See the details of (1,4,)-related L-R patterns |
| 12.5 | 5 | Pdgfb | Pdgfra  Pdgfrb | See the details of (1,5,)-related L-R patterns See the details of (1,6,)-related L-R patterns See the details of (2,5,)-related L-R patterns See the details of (2,6,)-related L-R patterns See the details of (4,6,)-related L-R patterns |
| 12.5 | 5 | Fn1 | Itgb8  Itga4  Itga8  Itgb3 | See the details of (1,5,)-related L-R patterns See the details of (1,3,)-related L-R patterns See the details of (1,4,)-related L-R patterns |
| 12.5 | 5 | Nxph1 | Nrxn3 | See the details of (5,7,)-related L-R patterns See the details of (5,4,)-related L-R patterns See the details of (6,7,)-related L-R patterns See the details of (4,4,)-related L-R patterns See the details of (6,4,)-related L-R patterns |
| 12.5 | 5 | Sst | Sstr2  Sstr3  Sstr1 | See the details of (5,4,)-related L-R patterns See the details of (3,5,)-related L-R patterns See the details of (5,5,)-related L-R patterns |
| 12.5 | 5 | Il34 | Csf1r | See the details of (1,2,)-related L-R patterns See the details of (2,2,)-related L-R patterns See the details of (2,4,)-related L-R patterns See the details of (5,2,)-related L-R patterns See the details of (5,6,)-related L-R patterns |
| 12.5 | 5 | Jag1 | Notch3  Notch4 | See the details of (1,1,)-related L-R patterns See the details of (2,1,)-related L-R patterns See the details of (2,6,)-related L-R patterns See the details of (4,6,)-related L-R patterns |
| 12.5 | 5 | Fgf12 | Fgfr2  Fgfr3 | See the details of (5,3,)-related L-R patterns See the details of (4,3,)-related L-R patterns See the details of (5,6,)-related L-R patterns |
| 12.5 | 5 | Fgf13 | Fgfr2  Fgfr3 | See the details of (5,3,)-related L-R patterns See the details of (4,3,)-related L-R patterns See the details of (5,6,)-related L-R patterns |
| 20.5 | 4 | Mdk | Ptprb  Ptprz1 | See the details of (6,1,)-related L-R patterns See the details of (6,5,)-related L-R patterns See the details of (6,4,)-related L-R patterns See the details of (6,2,)-related L-R patterns |
| 20.5 | 4 | Sema6d | Kdr | See the details of (6,1,)-related L-R patterns See the details of (4,1,)-related L-R patterns See the details of (3,1,)-related L-R patterns See the details of (5,1,)-related L-R patterns |
| 20.5 | 4 | Pdgfa | Pdgfra | See the details of (4,5,)-related L-R patterns See the details of (3,5,)-related L-R patterns See the details of (5,5,)-related L-R patterns See the details of (2,5,)-related L-R patterns |
| 20.5 | 4 | Fgf9 | Fgfr2  Fgfr3 | See the details of (5,3,)-related L-R patterns See the details of (4,3,)-related L-R patterns |
| 20.5 | 4 | Cck | Cckbr | See the details of (4,4,)-related L-R patterns See the details of (6,4,)-related L-R patterns See the details of (2,4,)-related L-R patterns See the details of (2,7,)-related L-R patterns |
| 20.5 | 4 | Efna2 | Epha4  Epha5 | See the details of (4,4,)-related L-R patterns See the details of (6,4,)-related L-R patterns |
| 20.5 | 4 | Col4a1 | Itgb8  Itga1 | See the details of (2,5,)-related L-R patterns See the details of (2,1,)-related L-R patterns See the details of (2,6,)-related L-R patterns See the details of (2,3,)-related L-R patterns |
| 20.5 | 4 | Vcam1 | Itga4  Itgb2 | See the details of (6,4,)-related L-R patterns See the details of (6,2,)-related L-R patterns See the details of (1,4,)-related L-R patterns See the details of (3,2,)-related L-R patterns |
| 27 | 3 | Fgf11 | Fgfr2  Fgfr3 | See the details of (5,3,)-related L-R patterns See the details of (4,3,)-related L-R patterns |
| 27 | 3 | Sema5a | Plxnb3 | See the details of (5,3,)-related L-R patterns See the details of (4,4,)-related L-R patterns See the details of (4,3,)-related L-R patterns |
| 27 | 3 | Dusp18 | Itga6 | See the details of (2,1,)-related L-R patterns See the details of (6,2,)-related L-R patterns See the details of (3,2,)-related L-R patterns |
| 27 | 3 | Icam5 | Itgb2 | See the details of (6,2,)-related L-R patterns See the details of (4,2,)-related L-R patterns See the details of (3,2,)-related L-R patterns |
| 27 | 3 | Nodal | Acvr1b  Acvr2a | See the details of (2,4,)-related L-R patterns See the details of (2,7,)-related L-R patterns |
| 36.5 | 2 | Cxcl12 | Ackr3 | See the details of (1,6,)-related L-R patterns See the details of (1,1,)-related L-R patterns |
| 36.5 | 2 | Icam2 | Itgb2 | See the details of (1,2,)-related L-R patterns See the details of (3,2,)-related L-R patterns |
| 36.5 | 2 | Vegfc | Kdr | See the details of (1,1,)-related L-R patterns See the details of (2,1,)-related L-R patterns |
| 36.5 | 2 | Kitl | Kit | See the details of (1,1,)-related L-R patterns See the details of (1,7,)-related L-R patterns |
| 36.5 | 2 | Angpt2 | Tek | See the details of (1,1,)-related L-R patterns See the details of (2,1,)-related L-R patterns |
| 36.5 | 2 | Edn3 | Ednrb | See the details of (2,5,)-related L-R patterns See the details of (4,3,)-related L-R patterns |
| 36.5 | 2 | Bmp5 | Acvrl1  Acvr2a | See the details of (2,1,)-related L-R patterns See the details of (2,4,)-related L-R patterns |
| 36.5 | 2 | Wnt4 | Fzd6 | See the details of (2,1,)-related L-R patterns See the details of (5,6,)-related L-R patterns |
| 36.5 | 2 | Gdf10 | Tgfbr1 | See the details of (6,2,)-related L-R patterns See the details of (3,2,)-related L-R patterns |
| 36.5 | 2 | Sema4d | Plxnb1 | See the details of (4,3,)-related L-R patterns See the details of (4,6,)-related L-R patterns |
| 36.5 | 2 | Col18a1 | Gpc1 | See the details of (4,3,)-related L-R patterns See the details of (1,4,)-related L-R patterns |
| 36.5 | 2 | Hbegf | Erbb4 | See the details of (4,3,)-related L-R patterns See the details of (2,7,)-related L-R patterns |
| 36.5 | 2 | Wnt5a | Fzd2  Fzd6 | See the details of (4,3,)-related L-R patterns See the details of (4,6,)-related L-R patterns |
| 36.5 | 2 | Pthlh | Pth1r | See the details of (4,6,)-related L-R patterns See the details of (5,6,)-related L-R patterns |
| 56 | 1 | Il33 | Il1rap | See the details of (3,5,)-related L-R patterns |
| 56 | 1 | Pdgfc | Pdgfra | See the details of (3,5,)-related L-R patterns |
| 56 | 1 | Vip | Vipr1 | See the details of (4,4,)-related L-R patterns |
| 56 | 1 | Sema3e | Plxnd1 | See the details of (4,4,)-related L-R patterns |
| 56 | 1 | Bmp7 | Acvr2a | See the details of (6,4,)-related L-R patterns |
| 56 | 1 | Inhbb | Acvr1b | See the details of (6,4,)-related L-R patterns |
| 56 | 1 | Pgf | Flt1 | See the details of (2,1,)-related L-R patterns |
| 56 | 1 | Lamc2 | Itga6 | See the details of (2,1,)-related L-R patterns |
| 56 | 1 | Fgf7 | Fgfr2 | See the details of (4,3,)-related L-R patterns |
| 56 | 1 | Fgf1 | Fgfr2 | See the details of (4,3,)-related L-R patterns |
| 56 | 1 | Slit2 | Gpc1 | See the details of (4,3,)-related L-R patterns |
| 56 | 1 | Fgf14 | Fgfr2 | See the details of (4,3,)-related L-R patterns |
| 56 | 1 | Col1a1 | Ddr1 | See the details of (4,3,)-related L-R patterns |
| 56 | 1 | Vtn | Itgb3 | See the details of (2,4,)-related L-R patterns |
| 56 | 1 | Dll4 | Notch3 | See the details of (4,6,)-related L-R patterns |
| 56 | 1 | Pdgfd | Pdgfrb | See the details of (4,6,)-related L-R patterns |
| 56 | 1 | Edn1 | Ednra | See the details of (4,6,)-related L-R patterns |
| 56 | 1 | Sema3c | Nrp2 | See the details of (1,4,)-related L-R patterns |
| 56 | 1 | Nxph3 | Nrxn3 | See the details of (2,7,)-related L-R patterns |
| 56 | 1 | Nrg3 | Erbb4 | See the details of (2,7,)-related L-R patterns |
| 56 | 1 | Dll3 | Notch3 | See the details of (5,6,)-related L-R patterns |
| 56 | 1 | Dll1 | Notch3 | See the details of (5,6,)-related L-R patterns |
| 56 | 1 | Dlk1 | Notch3 | See the details of (5,6,)-related L-R patterns |
| 56 | 1 | Tgfb3 | Tgfbr2 | See the details of (5,6,)-related L-R patterns |
| 56 | 1 | Sema3a | Plxna2 | See the details of (5,6,)-related L-R patterns |
