# Supplementary material for: Sctensor detects many-to-many cell–cell interactions from single cell RNA-sequencing data
Source: BMC Bioinformatics. 2023 Nov 7;24:420. doi: 10.1186/s12859-023-05490-y (PMC10631077; doi:10.1186/s12859-023-05490-y)
Supplement: Supplementary file 18 — Additional file 18. HTML report of VisualCortex. [file 12859_2023_5490_MOESM18_ESM.zip › output/report/Mouse_VisualCortex/ligand_all.html]

Details of Ligand Gene-centric Overview (all)


# Details of Ligand Gene-centric Overview (all)

| Ligand Gene | Receptor Gene |
| --- | --- |
| 100039053 | Ccrl2 |
| A2m | Lrp1 |
| Adam12 | Sdc4 |
| Adam15 | Itgb3 |
| Adam2 | Itga6 |
| Adam23 | Itgb3 |
| Adcyap1 | Adcyap1r1 Vipr1 Vipr2 |
| Adm | Calcrl Gpr182 Ramp2 Ramp3 |
| Adm2 | Calcrl Ramp2 Ramp3 |
| Agrp | Mc4r Mc5r |
| Agt | Agtr1b Agtr2 Lrp2 Mas1 Agtr1a |
| Alox5ap | Alox5 |
| Angpt1 | Tek |
| Angpt2 | Tek |
| Angpt4 | Tek |
| Angptl1 | Tek |
| Anxa1 | Fpr1 Fpr2 Fpr-rs7 |
| Apln | Aplnr |
| Apob | Ldlr |
| Apoe | Ldlr Lrp5 Lrp8 Sorl1 Vldlr |
| App | Fpr2 |
| Artn | Ret |
| Avp | Oxtr |
| Bdnf | Ngfr Ntrk2 |
| Bmp10 | Acvr2a Acvrl1 |
| Bmp15 | Acvr1 Acvr2b Bmpr1a Bmpr1b Bmpr2 |
| Bmp2 | Acvr1 Acvr2b Bmpr1a Bmpr1b Bmpr2 Acvr2a Acvrl1 |
| Bmp3 | Acvr1 Acvr2b Bmpr1a Bmpr1b Bmpr2 |
| Bmp4 | Acvr1 Acvr2b Bmpr1a Bmpr1b Bmpr2 Acvr2a Acvrl1 |
| Bmp5 | Acvr1 Acvr2b Bmpr1a Bmpr1b Bmpr2 Acvr2a Acvrl1 |
| Bmp6 | Acvr2a Acvrl1 |
| Bmp7 | Acvr1 Acvr2b Bmpr1a Bmpr1b Bmpr2 Acvr2a Acvrl1 |
| Btc | Erbb4 |
| C3 | C3ar1 Itgax Itgb2 |
| Calca | Calcr Calcrl Ramp1 Ramp2 Ramp3 |
| Calcb | Calcrl Ramp2 Ramp3 |
| Calm1 | Mylk Pde1a Pde1c |
| Calm2 | Mylk Pde1a Pde1c |
| Calm3 | Mylk Pde1a Pde1c |
| Calr | Scarf1 |
| Camp | Fpr2 P2rx7 |
| Cck | Cckar Cckbr |
| Ccl11 | Ccr2 Ccr3 Ccr5 |
| Ccl12 | Ccr2 |
| Ccl19 | Ccrl2 |
| Ccl2 | Ccr2 Ccr3 Ccr5 Ccr1 Ccr4 Cxcr3 |
| Ccl20 | Ccr6 Cxcr3 |
| Ccl22 | Ccr4 |
| Ccl24 | Ccr2 Ccr3 |
| Ccl28 | Ccr3 |
| Ccl3 | Ccr1 Ccr3 Ccr5 |
| Ccl4 | Ccr1 Ccr5 |
| Ccl5 | Cxcr3 Gpr75 |
| Ccl6 | Ccr1 Ccr3 |
| Ccl7 | Ccr1 Ccr2 Ccr3 Ccr5 Cxcr3 |
| Ccl8 | Ccr1 Ccr2 Ccr3 Ccr5 |
| Cd24a | Selp |
| Cd34 | Sell |
| Cd40lg | Cd40 |
| Cdh1 | Itgae |
| Chad | Itga2 Itgb1 |
| Clcf1 | Cntfr |
| Cntf | Cntfr Il6st Lifr |
| Col18a1 | Gpc1 Itgb1 |
| Col1a1 | Ddr1 Ddr2 Itga11 Itga2 Itgb1 |
| Col2a1 | Itga10 Itga2 Itgb1 |
| Col4a1 | Itga1 Itgb1 Itgb8 |
| Col5a3 | Sdc3 |
| Cort | Sstr1 Sstr2 Sstr3 Sstr4 Sstr5 Ghsr Mrgpra4 |
| Crh | Crhr1 Crhr2 Mc2r |
| Crlf1 | Cntfr |
| Csf1 | Csf1r |
| Csf2 | Csf1r Csf2rb Csf2rb2 |
| Csf3 | Csf1r Csf2rb Csf2rb2 |
| Ctf1 | Il6st Lifr |
| Cx3cl1 | Cx3cr1 |
| Cxcl10 | Ccr3 Cxcr3 |
| Cxcl12 | Ackr3 Cxcr3 Cxcr4 |
| Cxcl16 | Cxcr6 |
| Cxcl5 | Cxcr1 Cxcr2 |
| Cxcl9 | Ccr3 |
| Dhh | Hhip Ptch2 |
| Dlk1 | Notch1 Notch2 Notch3 Notch4 |
| Dll1 | Notch1 Notch2 Notch3 Notch4 |
| Dll3 | Notch1 Notch2 Notch3 Notch4 |
| Dll4 | Notch1 Notch2 Notch3 Notch4 |
| Dusp18 | Cd151 Itga1 Itga2 Itga3 Itga6 Itgb1 Itgb4 |
| Eda | Eda2r Edar |
| Edn1 | Ednra |
| Edn2 | Ednra |
| Edn3 | Ednrb |
| Efna1 | Epha2 Epha3 Epha4 Epha5 Epha7 |
| Efna2 | Epha1 Epha2 Epha3 Epha4 Epha5 Epha7 Epha8 Epha6 |
| Efna3 | Epha1 Epha2 Epha3 Epha4 Epha5 Epha7 Epha8 Epha6 Ephb1 |
| Efna4 | Epha2 Epha3 Epha4 Epha5 Epha7 |
| Efna5 | Epha1 Epha2 Epha3 Epha4 Epha5 Epha7 Epha8 Epha6 Ephb2 |
| Efnb1 | Epha4 Ephb1 Ephb2 Ephb6 |
| Efnb2 | Epha4 Ephb1 Ephb2 Ephb3 Ephb4 Ephb6 |
| Efnb3 | Epha4 Ephb1 Ephb2 Ephb3 Ephb4 Ephb6 |
| Epgn | Egfr |
| Ereg | Erbb4 |
| F2 | F2r F2rl1 |
| F7 | F3 |
| F9 | Lrp1 |
| Fasl | Fas |
| Fgf1 | Fgfr1 Fgfr2 Fgfr3 Fgfr4 |
| Fgf10 | Fgfr1 Fgfr2 Fgfr3 Fgfr4 |
| Fgf11 | Fgfr1 Fgfr2 Fgfr3 Fgfr4 |
| Fgf12 | Fgfr1 Fgfr2 Fgfr3 Fgfr4 |
| Fgf13 | Fgfr1 Fgfr2 Fgfr3 Fgfr4 |
| Fgf14 | Fgfr1 Fgfr2 Fgfr3 Fgfr4 |
| Fgf15 | Fgfr2 Fgfr3 |
| Fgf16 | Fgfr1 Fgfr2 Fgfr3 Fgfr4 |
| Fgf17 | Fgfr1 Fgfr2 Fgfr3 Fgfr4 |
| Fgf18 | Fgfr2 Fgfr3 |
| Fgf2 | Fgfr1 Fgfr2 Fgfr3 Fgfr4 Sdc2 Sdc4 |
| Fgf22 | Fgfr2 |
| Fgf23 | Fgfr2 |
| Fgf3 | Fgfr2 |
| Fgf4 | Fgfr2 Fgfr3 |
| Fgf5 | Fgfr2 Fgfr3 |
| Fgf6 | Fgfr2 |
| Fgf7 | Fgfr2 |
| Fgf8 | Fgfr2 Fgfr3 |
| Fgf9 | Fgfr1 Fgfr2 Fgfr3 Fgfr4 |
| Fn1 | Itga2 Itga2b Itga4 Itga8 Itgb1 Itgb3 Itgb6 Itgb8 Sdc2 |
| Gad1 | Grm4 |
| Gal | Galr1 |
| Galp | Galr1 |
| Gas6 | Axl |
| Gast | Cckbr |
| Gcg | Glp2r |
| Gdf1 | Acvr1b Acvr1c Acvr2a Tgfbr1 |
| Gdf10 | Acvr1b Acvr1c Acvr2a Acvr2b Bmpr1a Bmpr1b Bmpr2 Tgfbr1 |
| Gdf2 | Acvr1 Acvr2a Acvr2b Acvrl1 Bmpr1a Bmpr1b Bmpr2 |
| Gdf3 | Acvr1b Acvr1c Acvr2a Tgfbr1 |
| Gdf5 | Ror2 |
| Gdf6 | Bmpr1a Bmpr2 |
| Gdf7 | Acvr1b Acvr1c Acvr2a Tgfbr1 |
| Gdf9 | Acvr1b Acvr1c Acvr2a Tgfbr1 |
| Gdnf | Gfra1 Gfra2 Ret |
| Gh | Ghr |
| Ghrh | Vipr1 |
| Ghrl | Ghsr Gpr39 |
| Gm11127 | Klrd1 |
| Gm7030 | Klrd1 |
| Grp | Grpr |
| Guca1a | Gucy2f |
| Guca1b | Gucy2f |
| Has2 | Cd44 |
| Hbegf | Egfr Erbb4 |
| Hc | C5ar1 C5ar2 |
| Hcrt | Hcrtr1 Hcrtr2 |
| Hdc | Hrh1 Hrh2 Hrh3 Hrh4 |
| Hgf | Met |
| Iapp | Ramp2 Ramp3 |
| Icam1 | Itgal Itgax Itgb2 |
| Icam2 | Itgal Itgb2 |
| Icam4 | Itgal Itgam Itgb2 Itgb3 |
| Icam5 | Itgal Itgb2 |
| Ifnab | Ifnar1 Ifnar2 |
| Ifnl2 | Il10rb |
| Ifnl3 | Il10rb |
| Igf1 | Igf1r |
| Igf2 | Igf2r Igf1r Insr |
| Ihh | Hhip Ptch1 |
| Il10 | Il10ra Il10rb |
| Il12a | Il12rb1 Il12rb2 |
| Il12b | Il12rb1 Il12rb2 |
| Il13 | Il13ra1 Il2rg Il4ra |
| Il15 | Il2rg |
| Il16 | Cd4 |
| Il17b | Il17ra Il17rb |
| Il18 | Il1rapl1 |
| Il19 | Il20ra Il20rb |
| Il1a | Il1r1 Il1r2 Il1rap |
| Il1b | Il1r1 Il1r2 Il1rap |
| Il1r1 | Il1r2 |
| Il1rn | Il1r1 Il1rap Il2ra Il2rb Il2rg |
| Il2 | Il2ra Il2rg |
| Il20 | Il20ra Il20rb |
| Il21 | Il2rg |
| Il22 | Il10rb Il22ra1 Il10ra Il22ra2 |
| Il23a | Il12rb1 |
| Il24 | Il20ra Il20rb |
| Il25 | Il17rb |
| Il27 | Il27ra Il6st |
| Il3 | Csf2rb2 |
| Il31 | Osmr |
| Il33 | Il1rap Il1rl1 |
| Il34 | Csf1r |
| Il36a | Il1rap |
| Il36b | Il1rap |
| Il36g | Il1rap |
| Il36rn | Il1rap |
| Il4 | Il13ra1 Il2rg Il4ra |
| Il5 | Csf2rb Csf2rb2 |
| Il7 | Il2rg Il7r |
| Il9 | Il2rg |
| Inha | Acvr1b Acvr1c Acvr2a Tgfbr3 |
| Inhba | Acvr1 Acvr1b Acvr2b Acvr1c Acvr2a Bambi |
| Inhbb | Acvr1 Acvr1b Acvr2b Acvr1c Acvr2a |
| Inhbc | Acvr1b |
| Insl3 | Rxfp1 |
| Insl5 | Rxfp4 |
| Jag1 | Notch1 Notch2 Notch3 Notch4 |
| Jag2 | Notch3 Notch4 |
| Kitl | Kit |
| Lama1 | Itgb1 Itgb8 |
| Lama2 | Itgb1 |
| Lamb1 | Itga6 |
| Lamc2 | Itga3 Itga6 Itgb1 Itgb4 |
| Lefty2 | Acvr2b |
| Liph | Lpar1 Lpar4 |
| Lta | Ltbr Tnfrsf14 Tnfrsf1a Tnfrsf1b |
| Ltb | Cd40 |
| Madcam1 | Itga4 |
| Mdk | Ptprb Ptprz1 Alk |
| Mmp13 | Lrp1 |
| Mmp9 | Lrp1 |
| Ncam1 | Robo3 |
| Ndp | Fzd4 Lgr4 |
| Nid1 | Ptprf |
| Nlgn1 | Nrxn3 |
| Nlgn2 | Nrxn3 |
| Nlgn3 | Nrxn1 Nrxn2 Nrxn3 |
| Nmb | Grpr Nmbr |
| Nmu | Nmur1 Nmur2 |
| Nodal | Acvr1b Acvr1c Acvr2a Acvr2b |
| Npb | Npbwr1 |
| Npnt | Itga8 Itgb1 |
| Nppa | Npr3 |
| Nppb | Npr2 Npr3 |
| Nppc | Npr2 |
| Nps | Npsr1 |
| Npy | Npy2r Npy4r Prlhr Npy1r |
| Nrg1 | Erbb4 |
| Nrg2 | Erbb4 |
| Nrg3 | Erbb4 |
| Nrg4 | Erbb4 |
| Nrtn | Gfra1 Gfra2 Ret |
| Ntf3 | Ngfr Ntrk1 Ntrk2 E430016F16Rik |
| Ntn1 | Unc5c |
| Ntn3 | Cdon Neo1 |
| Ntn4 | Dcc Unc5a |
| Ntng1 | Lrrc4c |
| Nts | Ntsr1 Ntsr2 |
| Nxph1 | Nrxn1 Nrxn2 Nrxn3 |
| Nxph3 | Nrxn1 Nrxn2 Nrxn3 |
| Omg | Lingo1 Tnfrsf1b |
| Osm | Il6st Lifr Osmr |
| Ostn | Npr3 |
| Oxt | Oxtr |
| Pdgfa | Pdgfra |
| Pdgfb | Pdgfra Pdgfrb |
| Pdgfc | Flt4 Kdr Pdgfra |
| Pdgfd | Pdgfrb |
| Pdyn | Oprd1 Oprk1 Oprm1 |
| Penk | Mrgpra2b Oprd1 Oprm1 Ogfr |
| Pgf | Flt1 Nrp1 |
| Plat | Lrp1 |
| Plau | Plaur |
| Pmch | Mchr1 |
| Pnoc | Oprl1 |
| Podxl2 | Sell |
| Pomc | Mc4r Mc5r Oprk1 |
| Ppbp | Cxcr2 Cxcr1 |
| Pros1 | Axl |
| Psap | Gpr37 Gpr37l1 |
| Psen1 | Ncstn |
| Pspn | Ret |
| Ptdss1 | Scarb1 |
| Ptgs2 | Ptgdr2 |
| Pth | Pth1r Pth2r |
| Pth2 | Pth1r Pth2r |
| Pthlh | Prlhr Pth1r Pth2r |
| Ptn | Ptprb Ptprz1 |
| Pyy | Npy2r |
| Rarres2 | Cmklr1 |
| Reln | Itga3 |
| Ren1 | Atp6ap2 |
| Rgma | Neo1 |
| Rln1 | Rxfp1 Rxfp2 |
| Rln3 | Rxfp1 Rxfp3 |
| Rps19 | C5ar1 |
| Rspo1 | Lgr4 Lgr5 Lgr6 Lrp6 |
| Rspo2 | Lgr4 Lgr5 Lgr6 |
| Rspo3 | Lgr4 Lgr5 Lgr6 |
| Rspo4 | Lgr4 Lgr5 Lgr6 |
| Rtn4 | Lingo1 |
| Sct | Vipr1 |
| Selplg | Selp Sele |
| Sema3a | Nrp1 Plxna1 Plxna2 Plxna4 |
| Sema3c | Nrp2 |
| Sema3e | Plxnd1 |
| Sema3f | Nrp2 Plxna3 |
| Sema4d | Met Plxnb1 Plxnb2 |
| Sema5a | Met Plxnb3 |
| Sema6d | Kdr Plxna1 |
| Sema7a | Plxnc1 |
| Serpine1 | Lrp1 |
| Serping1 | Lrp1 |
| Shh | Boc Cdon Hhip Ptch1 Smo |
| Slit1 | Gpc1 |
| Slit2 | Gpc1 Robo1 Robo2 Robo4 Sdc1 |
| Spp1 | Cd44 Itgav |
| Sst | Mrgpra4 Sstr1 Sstr2 Sstr3 Sstr4 Sstr5 |
| Tac1 | Mrgpra4 Tacr1 Tacr2 Tacr3 |
| Tac2 | Tacr1 Tacr2 Tacr3 |
| Tac4 | Tacr1 Tacr3 |
| Tdgf1 | Acvr2a |
| Tgfb1 | Acvrl1 Tgfbr1 Tgfbr2 Tgfbr3 |
| Tgfb2 | Tgfbr1 Tgfbr2 Tgfbr3 |
| Tgfb3 | Tgfbr1 Tgfbr2 Tgfbr3 Acvrl1 |
| Thbs1 | Cd36 Cd47 Sdc1 |
| Thbs2 | Cd47 |
| Thpo | Mpl |
| Tnf | Tnfrsf1b Tnfrsf21 |
| Tnfsf10 | Tnfrsf26 Tnfrsf22 Tnfrsf11b |
| Tnfsf12 | Tnfrsf12a |
| Tnfsf13 | Tnfrsf13b |
| Tnfsf13b | Tnfrsf13b |
| Tnfsf18 | Tnfrsf18 |
| Tnfsf4 | Tnfrsf4 |
| Tnfsf8 | Tnfrsf8 |
| Tph1 | Htr1a Htr1f Htr2c Htr5a Htr7 |
| Trf | Tfrc |
| Trh | Trhr |
| Tslp | Crlf2 Il7r |
| Ucn | Crhr2 |
| Ucn2 | Crhr2 |
| Ucn3 | Crhr2 |
| Vcam1 | Itga4 Itgad Itgb1 Itgb2 |
| Vegfa | Flt1 Kdr Nrp1 |
| Vegfb | Flt1 |
| Vegfc | Flt4 Kdr Nrp2 Vipr2 |
| Vegfd | Flt4 |
| Vip | Adcyap1r1 Sctr Vipr1 Vipr2 |
| Vtn | Itgb3 |
| Wnt1 | Fzd3 Fzd8 Fzd9 Ryk |
| Wnt11 | Fzd7 |
| Wnt3 | Fzd8 Lrp6 Ryk |
| Wnt3a | Fzd6 |
| Wnt4 | Fzd6 Fzd2 |
| Wnt5a | Fzd6 Fzd2 Fzd4 Fzd7 Ryk |
| Wnt7a | Fzd5 |
| Wnt7b | Fzd1 |
| Zp3 | Mertk |
