# Supplementary material for: Sctensor detects many-to-many cell–cell interactions from single cell RNA-sequencing data
Source: BMC Bioinformatics. 2023 Nov 7;24:420. doi: 10.1186/s12859-023-05490-y (PMC10631077; doi:10.1186/s12859-023-05490-y)
Supplement: Supplementary file 18 — Additional file 18. HTML report of VisualCortex. [file 12859_2023_5490_MOESM18_ESM.zip › output/report/Mouse_VisualCortex/receptor.html]

Details of Receptor Gene-centric Overview (selected)


# Details of Receptor Gene-centric Overview (selected)

```
library("scTensor")
load("reanalysis.RData")
scTensor:::.myvisNetwork(g)
```

| Rank | No. of related pairs | Receptor Gene | Ligand Genes | Related CCIs |
| --- | --- | --- | --- | --- |
| 1 | 17 | Epha4 | Efnb3  Efna5  Efna3  Efna2  Efnb2 | See the details of (3,4,)-related L-R patterns See the details of (5,4,)-related L-R patterns See the details of (3,5,)-related L-R patterns See the details of (3,1,)-related L-R patterns See the details of (4,4,)-related L-R patterns See the details of (6,4,)-related L-R patterns See the details of (2,4,)-related L-R patterns See the details of (1,4,)-related L-R patterns |
| 2 | 13 | Nrxn3 | Nxph1  Nlgn3  Nxph3 | See the details of (5,7,)-related L-R patterns See the details of (5,4,)-related L-R patterns See the details of (6,7,)-related L-R patterns See the details of (4,4,)-related L-R patterns See the details of (6,4,)-related L-R patterns See the details of (4,7,)-related L-R patterns See the details of (2,4,)-related L-R patterns See the details of (2,7,)-related L-R patterns |
| 3 | 11 | Fgfr2 | Fgf12  Fgf13  Fgf9  Fgf11  Fgf7  Fgf1  Fgf14 | See the details of (5,3,)-related L-R patterns See the details of (4,3,)-related L-R patterns |
| 5 | 10 | Kdr | Vegfa  Sema6d  Vegfc | See the details of (6,1,)-related L-R patterns See the details of (1,1,)-related L-R patterns See the details of (4,1,)-related L-R patterns See the details of (3,1,)-related L-R patterns See the details of (5,1,)-related L-R patterns See the details of (2,1,)-related L-R patterns |
| 5 | 10 | Ldlr | Apoe | See the details of (6,1,)-related L-R patterns See the details of (1,1,)-related L-R patterns See the details of (3,5,)-related L-R patterns See the details of (6,6,)-related L-R patterns See the details of (6,4,)-related L-R patterns See the details of (6,3,)-related L-R patterns See the details of (6,2,)-related L-R patterns See the details of (1,4,)-related L-R patterns See the details of (3,6,)-related L-R patterns See the details of (3,2,)-related L-R patterns |
| 5 | 10 | Epha5 | Efna5  Efna3  Efna2 | See the details of (5,4,)-related L-R patterns See the details of (4,4,)-related L-R patterns See the details of (6,4,)-related L-R patterns See the details of (2,4,)-related L-R patterns See the details of (2,7,)-related L-R patterns |
| 7 | 9 | Fgfr3 | Fgf12  Fgf13  Fgf9  Fgf11 | See the details of (5,3,)-related L-R patterns See the details of (4,3,)-related L-R patterns See the details of (5,6,)-related L-R patterns |
| 8.5 | 8 | Notch3 | Jag1  Dll4  Dll3  Dll1  Dlk1 | See the details of (1,1,)-related L-R patterns See the details of (2,1,)-related L-R patterns See the details of (2,6,)-related L-R patterns See the details of (4,6,)-related L-R patterns See the details of (5,6,)-related L-R patterns |
| 8.5 | 8 | Ephb1 | Efnb3  Efna3  Efnb2 | See the details of (3,5,)-related L-R patterns See the details of (3,1,)-related L-R patterns See the details of (3,3,)-related L-R patterns See the details of (5,3,)-related L-R patterns See the details of (4,3,)-related L-R patterns |
| 11 | 7 | Pdgfra | Pdgfb  Pdgfa  Pdgfc | See the details of (1,5,)-related L-R patterns See the details of (4,5,)-related L-R patterns See the details of (3,5,)-related L-R patterns See the details of (5,5,)-related L-R patterns See the details of (2,5,)-related L-R patterns |
| 11 | 7 | Flt1 | Vegfa  Pgf | See the details of (6,1,)-related L-R patterns See the details of (4,1,)-related L-R patterns See the details of (5,1,)-related L-R patterns See the details of (2,1,)-related L-R patterns See the details of (4,6,)-related L-R patterns See the details of (5,6,)-related L-R patterns |
| 11 | 7 | Itgb2 | Icam2  Vcam1  Icam5 | See the details of (1,2,)-related L-R patterns See the details of (6,2,)-related L-R patterns See the details of (4,2,)-related L-R patterns See the details of (3,2,)-related L-R patterns |
| 13 | 6 | Tgfbr2 | Tgfb2  Tgfb3 | See the details of (1,2,)-related L-R patterns See the details of (1,1,)-related L-R patterns See the details of (2,1,)-related L-R patterns See the details of (6,2,)-related L-R patterns See the details of (4,2,)-related L-R patterns See the details of (5,6,)-related L-R patterns |
| 15 | 5 | Tgfbr1 | Tgfb2  Gdf10 | See the details of (1,2,)-related L-R patterns See the details of (6,2,)-related L-R patterns See the details of (4,2,)-related L-R patterns See the details of (3,2,)-related L-R patterns |
| 15 | 5 | Csf1r | Il34 | See the details of (1,2,)-related L-R patterns See the details of (2,2,)-related L-R patterns See the details of (2,4,)-related L-R patterns See the details of (5,2,)-related L-R patterns See the details of (5,6,)-related L-R patterns |
| 15 | 5 | Ephb6 | Efnb3  Efnb2 | See the details of (3,5,)-related L-R patterns See the details of (4,4,)-related L-R patterns See the details of (3,7,)-related L-R patterns See the details of (1,4,)-related L-R patterns See the details of (3,2,)-related L-R patterns |
| 18.5 | 4 | Itgb8 | Fn1  Col4a1 | See the details of (1,5,)-related L-R patterns See the details of (1,3,)-related L-R patterns See the details of (2,5,)-related L-R patterns See the details of (2,3,)-related L-R patterns |
| 18.5 | 4 | Pdgfrb | Pdgfb  Pdgfd | See the details of (1,6,)-related L-R patterns See the details of (2,6,)-related L-R patterns See the details of (4,6,)-related L-R patterns |
| 18.5 | 4 | Cckbr | Cck | See the details of (4,4,)-related L-R patterns See the details of (6,4,)-related L-R patterns See the details of (2,4,)-related L-R patterns See the details of (2,7,)-related L-R patterns |
| 18.5 | 4 | Itga6 | Dusp18  Lamc2 | See the details of (2,1,)-related L-R patterns See the details of (6,2,)-related L-R patterns See the details of (3,2,)-related L-R patterns |
| 25.5 | 3 | Sstr1 | Sst | See the details of (5,4,)-related L-R patterns See the details of (3,5,)-related L-R patterns See the details of (5,5,)-related L-R patterns |
| 25.5 | 3 | Ptprz1 | Mdk | See the details of (6,5,)-related L-R patterns See the details of (6,4,)-related L-R patterns See the details of (6,2,)-related L-R patterns |
| 25.5 | 3 | Ephb2 | Efnb3 | See the details of (3,5,)-related L-R patterns See the details of (3,7,)-related L-R patterns See the details of (3,2,)-related L-R patterns |
| 25.5 | 3 | Plxnb3 | Sema5a | See the details of (5,3,)-related L-R patterns See the details of (4,4,)-related L-R patterns See the details of (4,3,)-related L-R patterns |
| 25.5 | 3 | Acvr2a | Bmp7  Nodal  Bmp5 | See the details of (6,4,)-related L-R patterns See the details of (2,4,)-related L-R patterns |
| 25.5 | 3 | Itga4 | Vcam1  Fn1 | See the details of (6,4,)-related L-R patterns See the details of (1,4,)-related L-R patterns |
| 25.5 | 3 | Acvr1b | Inhbb  Nodal | See the details of (6,4,)-related L-R patterns See the details of (2,4,)-related L-R patterns See the details of (2,7,)-related L-R patterns |
| 25.5 | 3 | Fzd6 | Wnt4  Wnt5a | See the details of (2,1,)-related L-R patterns See the details of (4,6,)-related L-R patterns See the details of (5,6,)-related L-R patterns |
| 25.5 | 3 | Gpc1 | Col18a1  Slit2 | See the details of (4,3,)-related L-R patterns See the details of (1,4,)-related L-R patterns |
| 25.5 | 3 | Erbb4 | Hbegf  Nrg3 | See the details of (4,3,)-related L-R patterns See the details of (2,7,)-related L-R patterns |
| 35.5 | 2 | Ackr3 | Cxcl12 | See the details of (1,6,)-related L-R patterns See the details of (1,1,)-related L-R patterns |
| 35.5 | 2 | Kit | Kitl | See the details of (1,1,)-related L-R patterns See the details of (1,7,)-related L-R patterns |
| 35.5 | 2 | Tek | Angpt2 | See the details of (1,1,)-related L-R patterns See the details of (2,1,)-related L-R patterns |
| 35.5 | 2 | Tgfbr3 | Tgfb2 | See the details of (1,1,)-related L-R patterns See the details of (2,1,)-related L-R patterns |
| 35.5 | 2 | Ednrb | Edn3 | See the details of (2,5,)-related L-R patterns See the details of (4,3,)-related L-R patterns |
| 35.5 | 2 | Itga1 | Col4a1 | See the details of (2,1,)-related L-R patterns See the details of (2,6,)-related L-R patterns |
| 35.5 | 2 | Plxnb1 | Sema4d | See the details of (4,3,)-related L-R patterns See the details of (4,6,)-related L-R patterns |
| 35.5 | 2 | Itgb3 | Vtn  Fn1 | See the details of (2,4,)-related L-R patterns See the details of (1,4,)-related L-R patterns |
| 35.5 | 2 | Pth1r | Pthlh | See the details of (4,6,)-related L-R patterns See the details of (5,6,)-related L-R patterns |
| 35.5 | 2 | Epha2 | Efna5  Efna3 | See the details of (5,6,)-related L-R patterns |
| 47.5 | 1 | Ptprb | Mdk | See the details of (6,1,)-related L-R patterns |
| 47.5 | 1 | Sstr2 | Sst | See the details of (5,4,)-related L-R patterns |
| 47.5 | 1 | Sstr3 | Sst | See the details of (5,4,)-related L-R patterns |
| 47.5 | 1 | Il1rap | Il33 | See the details of (3,5,)-related L-R patterns |
| 47.5 | 1 | Vipr1 | Vip | See the details of (4,4,)-related L-R patterns |
| 47.5 | 1 | Plxnd1 | Sema3e | See the details of (4,4,)-related L-R patterns |
| 47.5 | 1 | Acvrl1 | Bmp5 | See the details of (2,1,)-related L-R patterns |
| 47.5 | 1 | Notch4 | Jag1 | See the details of (2,1,)-related L-R patterns |
| 47.5 | 1 | Fzd2 | Wnt5a | See the details of (4,3,)-related L-R patterns |
| 47.5 | 1 | Ddr1 | Col1a1 | See the details of (4,3,)-related L-R patterns |
| 47.5 | 1 | Ednra | Edn1 | See the details of (4,6,)-related L-R patterns |
| 47.5 | 1 | Itga8 | Fn1 | See the details of (1,4,)-related L-R patterns |
| 47.5 | 1 | Nrp2 | Sema3c | See the details of (1,4,)-related L-R patterns |
| 47.5 | 1 | Plxna2 | Sema3a | See the details of (5,6,)-related L-R patterns |
