# Supplementary material for: Sctensor detects many-to-many cell–cell interactions from single cell RNA-sequencing data
Source: BMC Bioinformatics. 2023 Nov 7;24:420. doi: 10.1186/s12859-023-05490-y (PMC10631077; doi:10.1186/s12859-023-05490-y)
Supplement: Supplementary file 18 — Additional file 18. HTML report of VisualCortex. [file 12859_2023_5490_MOESM18_ESM.zip › output/report/Mouse_VisualCortex/receptor_all.html]

Details of Receptor Gene-centric Overview (all)


# Details of Receptor Gene-centric Overview (all)

| Receptor Gene | Ligand Gene |
| --- | --- |
| Ackr3 | Cxcl12 |
| Acvr1 | Bmp15 Bmp2 Bmp3 Bmp4 Bmp5 Bmp7 Gdf2 Inhba Inhbb |
| Acvr1b | Gdf10 Inhba Inhbb Nodal Inhbc Inha Gdf9 Gdf1 Gdf3 Gdf7 |
| Acvr1c | Gdf10 Inhba Inhbb Nodal Gdf3 Gdf7 Gdf1 Inha Gdf9 |
| Acvr2a | Bmp2 Bmp4 Bmp5 Bmp7 Gdf10 Gdf2 Inhba Inhbb Nodal Gdf7 Gdf9 Gdf3 Bmp6 Bmp10 Inha Gdf1 Tdgf1 |
| Acvr2b | Bmp15 Bmp2 Bmp3 Bmp4 Bmp5 Bmp7 Gdf10 Gdf2 Inhba Inhbb Lefty2 Nodal |
| Acvrl1 | Bmp2 Bmp4 Bmp5 Bmp7 Gdf2 Tgfb3 Bmp6 Bmp10 Tgfb1 |
| Adcyap1r1 | Adcyap1 Vip |
| Agtr1a | Agt |
| Agtr1b | Agt |
| Agtr2 | Agt |
| Alk | Mdk |
| Alox5 | Alox5ap |
| Aplnr | Apln |
| Atp6ap2 | Ren1 |
| Axl | Pros1 Gas6 |
| Bambi | Inhba |
| Bmpr1a | Bmp15 Bmp2 Bmp3 Bmp4 Bmp5 Bmp7 Gdf10 Gdf2 Gdf6 |
| Bmpr1b | Bmp15 Bmp2 Bmp3 Bmp4 Bmp5 Bmp7 Gdf10 Gdf2 |
| Bmpr2 | Bmp15 Bmp2 Bmp3 Bmp4 Bmp5 Bmp7 Gdf10 Gdf2 Gdf6 |
| Boc | Shh |
| C3ar1 | C3 |
| C5ar1 | Hc Rps19 |
| C5ar2 | Hc |
| Calcr | Calca |
| Calcrl | Calca Adm2 Calcb Adm |
| Cckar | Cck |
| Cckbr | Cck Gast |
| Ccr1 | Ccl2 Ccl3 Ccl4 Ccl6 Ccl7 Ccl8 |
| Ccr2 | Ccl12 Ccl2 Ccl24 Ccl7 Ccl8 Ccl11 |
| Ccr3 | Ccl2 Ccl24 Ccl3 Ccl6 Ccl7 Ccl8 Cxcl10 Ccl11 Ccl28 Cxcl9 |
| Ccr4 | Ccl2 Ccl22 |
| Ccr5 | Ccl2 Ccl3 Ccl4 Ccl7 Ccl8 Ccl11 |
| Ccr6 | Ccl20 |
| Ccrl2 | Ccl19 100039053 |
| Cd151 | Dusp18 |
| Cd36 | Thbs1 |
| Cd4 | Il16 |
| Cd40 | Cd40lg Ltb |
| Cd44 | Spp1 Has2 |
| Cd47 | Thbs1 Thbs2 |
| Cdon | Shh Ntn3 |
| Cmklr1 | Rarres2 |
| Cntfr | Cntf Clcf1 Crlf1 |
| Crhr1 | Crh |
| Crhr2 | Crh Ucn Ucn2 Ucn3 |
| Crlf2 | Tslp |
| Csf1r | Il34 Csf1 Csf2 Csf3 |
| Csf2rb | Csf2 Il5 Csf3 |
| Csf2rb2 | Csf2 Il5 Il3 Csf3 |
| Cx3cr1 | Cx3cl1 |
| Cxcr1 | Cxcl5 Ppbp |
| Cxcr2 | Cxcl5 Ppbp |
| Cxcr3 | Ccl2 Ccl20 Ccl5 Ccl7 Cxcl10 Cxcl12 |
| Cxcr4 | Cxcl12 |
| Cxcr6 | Cxcl16 |
| Dcc | Ntn4 |
| Ddr1 | Col1a1 |
| Ddr2 | Col1a1 |
| E430016F16Rik | Ntf3 |
| Eda2r | Eda |
| Edar | Eda |
| Ednra | Edn1 Edn2 |
| Ednrb | Edn3 |
| Egfr | Epgn Hbegf |
| Epha1 | Efna2 Efna3 Efna5 |
| Epha2 | Efna2 Efna3 Efna5 Efna4 Efna1 |
| Epha3 | Efna2 Efna3 Efna5 Efna1 Efna4 |
| Epha4 | Efna2 Efna3 Efna5 Efnb2 Efnb3 Efna4 Efna1 Efnb1 |
| Epha5 | Efna2 Efna3 Efna5 Efna1 Efna4 |
| Epha6 | Efna2 Efna3 Efna5 |
| Epha7 | Efna2 Efna3 Efna5 Efna1 Efna4 |
| Epha8 | Efna2 Efna3 Efna5 |
| Ephb1 | Efna3 Efnb2 Efnb3 Efnb1 |
| Ephb2 | Efna5 Efnb2 Efnb3 Efnb1 |
| Ephb3 | Efnb2 Efnb3 |
| Ephb4 | Efnb2 Efnb3 |
| Ephb6 | Efnb2 Efnb3 Efnb1 |
| Erbb4 | Hbegf Nrg3 Ereg Btc Nrg1 Nrg2 Nrg4 |
| F2r | F2 |
| F2rl1 | F2 |
| F3 | F7 |
| Fas | Fasl |
| Fgfr1 | Fgf1 Fgf10 Fgf11 Fgf12 Fgf13 Fgf14 Fgf16 Fgf17 Fgf2 Fgf9 |
| Fgfr2 | Fgf1 Fgf10 Fgf11 Fgf12 Fgf13 Fgf14 Fgf16 Fgf17 Fgf2 Fgf7 Fgf9 Fgf6 Fgf18 Fgf15 Fgf3 Fgf8 Fgf4 Fgf5 Fgf22 Fgf23 |
| Fgfr3 | Fgf1 Fgf10 Fgf11 Fgf12 Fgf13 Fgf14 Fgf16 Fgf17 Fgf2 Fgf9 Fgf18 Fgf15 Fgf4 Fgf8 Fgf5 |
| Fgfr4 | Fgf1 Fgf10 Fgf11 Fgf12 Fgf13 Fgf14 Fgf16 Fgf17 Fgf2 Fgf9 |
| Flt1 | Pgf Vegfa Vegfb |
| Flt4 | Pdgfc Vegfc Vegfd |
| Fpr-rs7 | Anxa1 |
| Fpr1 | Anxa1 |
| Fpr2 | Anxa1 Camp App |
| Fzd1 | Wnt7b |
| Fzd2 | Wnt4 Wnt5a |
| Fzd3 | Wnt1 |
| Fzd4 | Ndp Wnt5a |
| Fzd5 | Wnt7a |
| Fzd6 | Wnt4 Wnt5a Wnt3a |
| Fzd7 | Wnt5a Wnt11 |
| Fzd8 | Wnt1 Wnt3 |
| Fzd9 | Wnt1 |
| Galr1 | Gal Galp |
| Gfra1 | Nrtn Gdnf |
| Gfra2 | Nrtn Gdnf |
| Ghr | Gh |
| Ghsr | Cort Ghrl |
| Glp2r | Gcg |
| Gpc1 | Col18a1 Slit2 Slit1 |
| Gpr182 | Adm |
| Gpr37 | Psap |
| Gpr37l1 | Psap |
| Gpr39 | Ghrl |
| Gpr75 | Ccl5 |
| Grm4 | Gad1 |
| Grpr | Grp Nmb |
| Gucy2f | Guca1b Guca1a |
| Hcrtr1 | Hcrt |
| Hcrtr2 | Hcrt |
| Hhip | Dhh Ihh Shh |
| Hrh1 | Hdc |
| Hrh2 | Hdc |
| Hrh3 | Hdc |
| Hrh4 | Hdc |
| Htr1a | Tph1 |
| Htr1f | Tph1 |
| Htr2c | Tph1 |
| Htr5a | Tph1 |
| Htr7 | Tph1 |
| Ifnar1 | Ifnab |
| Ifnar2 | Ifnab |
| Igf1r | Igf1 Igf2 |
| Igf2r | Igf2 |
| Il10ra | Il22 Il10 |
| Il10rb | Il22 Il10 Ifnl2 Ifnl3 |
| Il12rb1 | Il12a Il23a Il12b |
| Il12rb2 | Il12a Il12b |
| Il13ra1 | Il13 Il4 |
| Il17ra | Il17b |
| Il17rb | Il17b Il25 |
| Il1r1 | Il1a Il1b Il1rn |
| Il1r2 | Il1a Il1b Il1r1 |
| Il1rap | Il1a Il1b Il1rn Il33 Il36a Il36g Il36b Il36rn |
| Il1rapl1 | Il18 |
| Il1rl1 | Il33 |
| Il20ra | Il19 Il24 Il20 |
| Il20rb | Il24 Il20 Il19 |
| Il22ra1 | Il22 |
| Il22ra2 | Il22 |
| Il27ra | Il27 |
| Il2ra | Il1rn Il2 |
| Il2rb | Il1rn |
| Il2rg | Il1rn Il15 Il13 Il7 Il9 Il2 Il4 Il21 |
| Il4ra | Il4 Il13 |
| Il6st | Cntf Ctf1 Il27 Osm |
| Il7r | Tslp Il7 |
| Insr | Igf2 |
| Itga1 | Col4a1 Dusp18 |
| Itga10 | Col2a1 |
| Itga11 | Col1a1 |
| Itga2 | Chad Col1a1 Col2a1 Dusp18 Fn1 |
| Itga2b | Fn1 |
| Itga3 | Dusp18 Lamc2 Reln |
| Itga4 | Fn1 Vcam1 Madcam1 |
| Itga6 | Dusp18 Lamc2 Lamb1 Adam2 |
| Itga8 | Fn1 Npnt |
| Itgad | Vcam1 |
| Itgae | Cdh1 |
| Itgal | Icam1 Icam2 Icam5 Icam4 |
| Itgam | Icam4 |
| Itgav | Spp1 |
| Itgax | Icam1 C3 |
| Itgb1 | Chad Col18a1 Col1a1 Col2a1 Col4a1 Dusp18 Fn1 Lama1 Lama2 Lamc2 Npnt Vcam1 |
| Itgb2 | Icam1 Icam2 Icam5 Vcam1 C3 Icam4 |
| Itgb3 | Fn1 Vtn Icam4 Adam23 Adam15 |
| Itgb4 | Dusp18 Lamc2 |
| Itgb6 | Fn1 |
| Itgb8 | Col4a1 Fn1 Lama1 |
| Kdr | Pdgfc Sema6d Vegfa Vegfc |
| Kit | Kitl |
| Klrd1 | Gm11127 Gm7030 |
| Ldlr | Apoe Apob |
| Lgr4 | Ndp Rspo1 Rspo2 Rspo3 Rspo4 |
| Lgr5 | Rspo1 Rspo2 Rspo3 Rspo4 |
| Lgr6 | Rspo1 Rspo2 Rspo3 Rspo4 |
| Lifr | Cntf Ctf1 Osm |
| Lingo1 | Rtn4 Omg |
| Lpar1 | Liph |
| Lpar4 | Liph |
| Lrp1 | A2m F9 Mmp13 Mmp9 Plat Serpine1 Serping1 |
| Lrp2 | Agt |
| Lrp5 | Apoe |
| Lrp6 | Rspo1 Wnt3 |
| Lrp8 | Apoe |
| Lrrc4c | Ntng1 |
| Ltbr | Lta |
| Mas1 | Agt |
| Mc2r | Crh |
| Mc4r | Agrp Pomc |
| Mc5r | Pomc Agrp |
| Mchr1 | Pmch |
| Mertk | Zp3 |
| Met | Hgf Sema4d Sema5a |
| Mpl | Thpo |
| Mrgpra2b | Penk |
| Mrgpra4 | Cort Sst Tac1 |
| Mylk | Calm1 Calm3 Calm2 |
| Ncstn | Psen1 |
| Neo1 | Rgma Ntn3 |
| Ngfr | Bdnf Ntf3 |
| Nmbr | Nmb |
| Nmur1 | Nmu |
| Nmur2 | Nmu |
| Notch1 | Dlk1 Dll1 Dll3 Dll4 Jag1 |
| Notch2 | Dlk1 Dll1 Dll3 Dll4 Jag1 |
| Notch3 | Dlk1 Dll1 Dll3 Dll4 Jag1 Jag2 |
| Notch4 | Dlk1 Dll1 Dll3 Dll4 Jag1 Jag2 |
| Npbwr1 | Npb |
| Npr2 | Nppc Nppb |
| Npr3 | Ostn Nppb Nppa |
| Npsr1 | Nps |
| Npy1r | Npy |
| Npy2r | Npy Pyy |
| Npy4r | Npy |
| Nrp1 | Pgf Sema3a Vegfa |
| Nrp2 | Sema3c Vegfc Sema3f |
| Nrxn1 | Nlgn3 Nxph1 Nxph3 |
| Nrxn2 | Nlgn3 Nxph1 Nxph3 |
| Nrxn3 | Nlgn3 Nxph1 Nxph3 Nlgn1 Nlgn2 |
| Ntrk1 | Ntf3 |
| Ntrk2 | Bdnf Ntf3 |
| Ntsr1 | Nts |
| Ntsr2 | Nts |
| Ogfr | Penk |
| Oprd1 | Pdyn Penk |
| Oprk1 | Pdyn Pomc |
| Oprl1 | Pnoc |
| Oprm1 | Pdyn Penk |
| Osmr | Osm Il31 |
| Oxtr | Avp Oxt |
| P2rx7 | Camp |
| Pde1a | Calm1 Calm3 Calm2 |
| Pde1c | Calm3 Calm1 Calm2 |
| Pdgfra | Pdgfa Pdgfb Pdgfc |
| Pdgfrb | Pdgfb Pdgfd |
| Plaur | Plau |
| Plxna1 | Sema3a Sema6d |
| Plxna2 | Sema3a |
| Plxna3 | Sema3f |
| Plxna4 | Sema3a |
| Plxnb1 | Sema4d |
| Plxnb2 | Sema4d |
| Plxnb3 | Sema5a |
| Plxnc1 | Sema7a |
| Plxnd1 | Sema3e |
| Prlhr | Npy Pthlh |
| Ptch1 | Ihh Shh |
| Ptch2 | Dhh |
| Ptgdr2 | Ptgs2 |
| Pth1r | Pthlh Pth2 Pth |
| Pth2r | Pthlh Pth2 Pth |
| Ptprb | Mdk Ptn |
| Ptprf | Nid1 |
| Ptprz1 | Mdk Ptn |
| Ramp1 | Calca |
| Ramp2 | Calca Iapp Adm Adm2 Calcb |
| Ramp3 | Calca Adm Adm2 Calcb Iapp |
| Ret | Nrtn Gdnf Artn Pspn |
| Robo1 | Slit2 |
| Robo2 | Slit2 |
| Robo3 | Ncam1 |
| Robo4 | Slit2 |
| Ror2 | Gdf5 |
| Rxfp1 | Rln1 Insl3 Rln3 |
| Rxfp2 | Rln1 |
| Rxfp3 | Rln3 |
| Rxfp4 | Insl5 |
| Ryk | Wnt1 Wnt3 Wnt5a |
| Scarb1 | Ptdss1 |
| Scarf1 | Calr |
| Sctr | Vip |
| Sdc1 | Slit2 Thbs1 |
| Sdc2 | Fgf2 Fn1 |
| Sdc3 | Col5a3 |
| Sdc4 | Fgf2 Adam12 |
| Sele | Selplg |
| Sell | Cd34 Podxl2 |
| Selp | Cd24a Selplg |
| Smo | Shh |
| Sorl1 | Apoe |
| Sstr1 | Cort Sst |
| Sstr2 | Cort Sst |
| Sstr3 | Cort Sst |
| Sstr4 | Cort Sst |
| Sstr5 | Cort Sst |
| Tacr1 | Tac1 Tac2 Tac4 |
| Tacr2 | Tac1 Tac2 |
| Tacr3 | Tac1 Tac2 Tac4 |
| Tek | Angpt2 Angpt4 Angptl1 Angpt1 |
| Tfrc | Trf |
| Tgfbr1 | Gdf10 Tgfb2 Tgfb3 Tgfb1 Gdf3 Gdf7 Gdf1 Gdf9 |
| Tgfbr2 | Tgfb2 Tgfb3 Tgfb1 |
| Tgfbr3 | Tgfb2 Tgfb3 Tgfb1 Inha |
| Tnfrsf11b | Tnfsf10 |
| Tnfrsf12a | Tnfsf12 |
| Tnfrsf13b | Tnfsf13b Tnfsf13 |
| Tnfrsf14 | Lta |
| Tnfrsf18 | Tnfsf18 |
| Tnfrsf1a | Lta |
| Tnfrsf1b | Lta Tnf Omg |
| Tnfrsf21 | Tnf |
| Tnfrsf22 | Tnfsf10 |
| Tnfrsf26 | Tnfsf10 |
| Tnfrsf4 | Tnfsf4 |
| Tnfrsf8 | Tnfsf8 |
| Trhr | Trh |
| Unc5a | Ntn4 |
| Unc5c | Ntn1 |
| Vipr1 | Adcyap1 Vip Sct Ghrh |
| Vipr2 | Adcyap1 Vegfc Vip |
| Vldlr | Apoe |
