# Supplementary material for: Sctensor detects many-to-many cell–cell interactions from single cell RNA-sequencing data
Source: BMC Bioinformatics. 2023 Nov 7;24:420. doi: 10.1186/s12859-023-05490-y (PMC10631077; doi:10.1186/s12859-023-05490-y)
Supplement: Supplementary file 18 — Additional file 18. HTML report of VisualCortex. [file 12859_2023_5490_MOESM18_ESM.zip › output/report/Mouse_VisualCortex/pattern_5_4.html]

Details of (5,4,) -related L-R pairs


# Details of (5,4,) -related L-R pairs

# (5,4,:) Pattern

# (5,4,) -related L-R pairs (Top10 pairs)

```
library("scTensor")
load("reanalysis.RData")
col <- .setColor("many")[7]
scTensor:::.myvisNetwork(g, col)
```

| Rank | Ligand Gene | Receptor Gene | Ligand Expression (Log10(exp + 1)) | Receptor Expression (Log10(exp + 1)) | LR-pair factor value (Percentage) | P-value (Grubbs test) | Q-value (BH method) | L-R Evidence cf. rikenbit/lrbase-workflow | PubMed |
| --- | --- | --- | --- | --- | --- | --- | --- | --- | --- |
| 1 | Nxph1 Description: Nxph1neurexophilin 1 GO: 1-2 UniProtKB: 1 STRING: 1 2 RefEx: Nxph1 Expression Atlas: Nxph1 Single Cell Expression Atlas: Nxph1 PanglaoDB: Nxph1 MeSH: 1-91 92-181 | Nrxn3 Description: Nxph1neurexin III GO: 1-18 Reactome: 1-3 UniProtKB: 1-2 RefEx: Nxph1 Expression Atlas: Nrxn3 Single Cell Expression Atlas: Nrxn3 PanglaoDB: Nrxn3 MeSH: 1-78 79-156 157-234 235-311 |  |  | 0.181 (19.506%) | 0 | 0 | DLRP | 1 |
| 2 | Sst Description: Sstsomatostatin GO: 1-6 Reactome: 1-7 UniProtKB: 1-2 STRING: 1 RefEx: Sst Expression Atlas: Sst Single Cell Expression Atlas: Sst PanglaoDB: Sst MeSH: 1-97 98-194 195-291 292-388 389-485 486-582 583-679 680-776 777-873 874-970 971-1067 1068-1164 1165-1261 1262-1358 1359-1455 1456-1552 1553-1649 1650-1746 1747-1843 1844-1940 1941-2037 2038-2127 | Sstr2 Description: Sstsomatostatin receptor 2 GO: 1-20 Reactome: 1-7 UniProtKB: 1 STRING: 1 2 3 RefEx: Sst Expression Atlas: Sstr2 Single Cell Expression Atlas: Sstr2 PanglaoDB: Sstr2 MeSH: 1-96 97-192 193-288 289-384 385-480 481-576 577-668 |  |  | 0.13 (13.952%) | 0 | 0 | DLRP | 1 |
| 3 | Efna5 Description: Efna5ephrin A5 GO: 1-34 Reactome: 1-6 UniProtKB: 1 RefEx: Efna5 Expression Atlas: Efna5 Single Cell Expression Atlas: Efna5 PanglaoDB: Efna5 MeSH: 1-90 91-180 181-270 271-360 361-450 451-540 541-630 631-718 | Epha4 Description: Efna5Eph receptor A4 GO: 1-90 Reactome: 1-6 UniProtKB: 1 RefEx: Efna5 Expression Atlas: Epha4 Single Cell Expression Atlas: Epha4 PanglaoDB: Epha4 MeSH: 1-95 96-190 191-285 286-380 381-475 476-570 571-665 666-760 761-855 856-950 951-1045 1046-1131 |  |  | 0.063 (6.744%) | 0 | 0 | DLRP | 1 |
| 4 | Efnb3 Description: Efnb3ephrin B3 GO: 1-18 Reactome: 1-7 UniProtKB: 1-2 STRING: 1 RefEx: Efnb3 Expression Atlas: Efnb3 Single Cell Expression Atlas: Efnb3 PanglaoDB: Efnb3 MeSH: 1-89 90-178 179-267 268-356 357-445 446-534 535-622 | Epha4 Description: Efnb3Eph receptor A4 GO: 1-90 Reactome: 1-6 UniProtKB: 1 RefEx: Efnb3 Expression Atlas: Epha4 Single Cell Expression Atlas: Epha4 PanglaoDB: Epha4 MeSH: 1-95 96-190 191-285 286-380 381-475 476-570 571-665 666-760 761-855 856-950 951-1045 1046-1131 |  |  | 0.06 (6.466%) | 0 | 0 | DLRP | 1 |
| 5 | Efna3 Description: Efna3ephrin A3 GO: 1-8 Reactome: 1-6 UniProtKB: 1 RefEx: Efna3 Expression Atlas: Efna3 Single Cell Expression Atlas: Efna3 PanglaoDB: Efna3 MeSH: 1-84 85-168 169-252 253-336 337-419 | Epha4 Description: Efna3Eph receptor A4 GO: 1-90 Reactome: 1-6 UniProtKB: 1 RefEx: Efna3 Expression Atlas: Epha4 Single Cell Expression Atlas: Epha4 PanglaoDB: Epha4 MeSH: 1-95 96-190 191-285 286-380 381-475 476-570 571-665 666-760 761-855 856-950 951-1045 1046-1131 |  |  | 0.053 (5.75%) | 0 | 0 | DLRP | 1 |
| 6 | Sst Description: Sstsomatostatin GO: 1-6 Reactome: 1-7 UniProtKB: 1-2 STRING: 1 RefEx: Sst Expression Atlas: Sst Single Cell Expression Atlas: Sst PanglaoDB: Sst MeSH: 1-97 98-194 195-291 292-388 389-485 486-582 583-679 680-776 777-873 874-970 971-1067 1068-1164 1165-1261 1262-1358 1359-1455 1456-1552 1553-1649 1650-1746 1747-1843 1844-1940 1941-2037 2038-2127 | Sstr3 Description: Sstsomatostatin receptor 3 GO: 1-17 Reactome: 1-11 UniProtKB: 1 STRING: 1 2 RefEx: Sst Expression Atlas: Sstr3 Single Cell Expression Atlas: Sstr3 PanglaoDB: Sstr3 MeSH: 1-77 78-154 155-231 232-306 |  |  | 0.053 (5.692%) | 0 | 0 | DLRP | 1 |
| 7 | Efna5 Description: Efna5ephrin A5 GO: 1-34 Reactome: 1-6 UniProtKB: 1 RefEx: Efna5 Expression Atlas: Efna5 Single Cell Expression Atlas: Efna5 PanglaoDB: Efna5 MeSH: 1-90 91-180 181-270 271-360 361-450 451-540 541-630 631-718 | Epha5 Description: Efna5Eph receptor A5 GO: 1-45 Reactome: 1-6 UniProtKB: 1-2 RefEx: Efna5 Expression Atlas: Epha5 Single Cell Expression Atlas: Epha5 PanglaoDB: Epha5 MeSH: 1-98 99-196 197-294 295-392 393-489 |  |  | 0.045 (4.835%) | 0 | 0 | DLRP | 1 |
| 8 | Nlgn3 Description: Nlgn3neuroligin 3 GO: 1-59 Reactome: 1-3 UniProtKB: 1 RefEx: Nlgn3 Expression Atlas: Nlgn3 Single Cell Expression Atlas: Nlgn3 PanglaoDB: Nlgn3 MeSH: 1-82 83-164 165-246 247-328 329-408 | Nrxn3 Description: Nlgn3neurexin III GO: 1-18 Reactome: 1-3 UniProtKB: 1-2 RefEx: Nlgn3 Expression Atlas: Nrxn3 Single Cell Expression Atlas: Nrxn3 PanglaoDB: Nrxn3 MeSH: 1-78 79-156 157-234 235-311 |  |  | 0.044 (4.751%) | 0 | 0 | DLRP | 1 |
| 9 | Efna3 Description: Efna3ephrin A3 GO: 1-8 Reactome: 1-6 UniProtKB: 1 RefEx: Efna3 Expression Atlas: Efna3 Single Cell Expression Atlas: Efna3 PanglaoDB: Efna3 MeSH: 1-84 85-168 169-252 253-336 337-419 | Epha5 Description: Efna3Eph receptor A5 GO: 1-45 Reactome: 1-6 UniProtKB: 1-2 RefEx: Efna3 Expression Atlas: Epha5 Single Cell Expression Atlas: Epha5 PanglaoDB: Epha5 MeSH: 1-98 99-196 197-294 295-392 393-489 |  |  | 0.038 (4.122%) | 0 | 0 | DLRP | 1 |
| 10 | Sst Description: Sstsomatostatin GO: 1-6 Reactome: 1-7 UniProtKB: 1-2 STRING: 1 RefEx: Sst Expression Atlas: Sst Single Cell Expression Atlas: Sst PanglaoDB: Sst MeSH: 1-97 98-194 195-291 292-388 389-485 486-582 583-679 680-776 777-873 874-970 971-1067 1068-1164 1165-1261 1262-1358 1359-1455 1456-1552 1553-1649 1650-1746 1747-1843 1844-1940 1941-2037 2038-2127 | Sstr1 Description: Sstsomatostatin receptor 1 GO: 1-17 Reactome: 1-7 UniProtKB: 1-2 STRING: 1 2 RefEx: Sst Expression Atlas: Sstr1 Single Cell Expression Atlas: Sstr1 PanglaoDB: Sstr1 MeSH: 1-90 91-180 181-270 271-358 |  |  | 0.036 (3.832%) | 0 | 0 | DLRP | 1 |

# (5,4,) -related Enrichment Analysis

```
load("reanalysis.RData")
library("plotly")
```

## MeSH-Enrichment Analysis (A : Anatomy)

```
negLogPval <- -log10(out.vecLR[['pattern_5_4']]$Enrich$MeSH_A$Pvalue + 1E-10)
term <- out.vecLR[['pattern_5_4']]$Enrich$MeSH_A$Term
target <- seq_len(min(100, length(negLogPval)))
negLogPval <- negLogPval[target]
term <- term[target]
p <- plot_ly(x=seq_along(negLogPval), y=~negLogPval,
type="bar", color=~negLogPval, text=term,
colors=c("#4b61ba", "gray", "#a87963", "red"))
layout(p, title="MeSH-Enrichment Analysis (A)",
   xaxis=list(title="Term"),
   yaxis=list(title="-Log(P-value)"),
   legend=list(name=""))
```

```
## Warning: textfont.color doesn't (yet) support data arrays

## Warning: textfont.color doesn't (yet) support data arrays
```

## MeSH-Enrichment Analysis (B : Organisms)

```
negLogPval <- -log10(out.vecLR[['pattern_5_4']]$Enrich$MeSH_B$Pvalue + 1E-10)
term <- out.vecLR[['pattern_5_4']]$Enrich$MeSH_B$Term
target <- seq_len(min(100, length(negLogPval)))
negLogPval <- negLogPval[target]
term <- term[target]
p <- plot_ly(x=seq_along(negLogPval), y=~negLogPval,
type="bar", color=~negLogPval, text=term,
colors=c("#4b61ba", "gray", "#a87963", "red"))
layout(p, title="MeSH-Enrichment Analysis (B)",
   xaxis=list(title="Term"),
   yaxis=list(title="-Log(P-value)"),
   legend=list(name=""))
```

```
## Warning: textfont.color doesn't (yet) support data arrays

## Warning: textfont.color doesn't (yet) support data arrays
```

## MeSH-Enrichment Analysis (C : Diseases)

```
negLogPval <- -log10(out.vecLR[['pattern_5_4']]$Enrich$MeSH_C$Pvalue + 1E-10)
term <- out.vecLR[['pattern_5_4']]$Enrich$MeSH_C$Term
target <- seq_len(min(100, length(negLogPval)))
negLogPval <- negLogPval[target]
term <- term[target]
p <- plot_ly(x=seq_along(negLogPval), y=~negLogPval,
type="bar", color=~negLogPval, text=term,
colors=c("#4b61ba", "gray", "#a87963", "red"))
layout(p, title="MeSH-Enrichment Analysis (C)",
   xaxis=list(title="Term"),
   yaxis=list(title="-Log(P-value)"),
   legend=list(name=""))
```

```
## Warning: textfont.color doesn't (yet) support data arrays

## Warning: textfont.color doesn't (yet) support data arrays
```

## MeSH-Enrichment Analysis (D : Drugs)

```
negLogPval <- -log10(out.vecLR[['pattern_5_4']]$Enrich$MeSH_D$Pvalue + 1E-10)
term <- out.vecLR[['pattern_5_4']]$Enrich$MeSH_D$Term
target <- seq_len(min(100, length(negLogPval)))
negLogPval <- negLogPval[target]
term <- term[target]
p <- plot_ly(x=seq_along(negLogPval), y=~negLogPval,
type="bar", color=~negLogPval, text=term,
colors=c("#4b61ba", "gray", "#a87963", "red"))
layout(p, title="MeSH-Enrichment Analysis (D)",
   xaxis=list(title="Term"),
   yaxis=list(title="-Log(P-value)"),
   legend=list(name=""))
```

```
## Warning: textfont.color doesn't (yet) support data arrays

## Warning: textfont.color doesn't (yet) support data arrays
```

## MeSH-Enrichment Analysis (E : Analytical, Diagnostic and Therapeutic Techniques and Equipment)

```
negLogPval <- -log10(out.vecLR[['pattern_5_4']]$Enrich$MeSH_E$Pvalue + 1E-10)
term <- out.vecLR[['pattern_5_4']]$Enrich$MeSH_E$Term
target <- seq_len(min(100, length(negLogPval)))
negLogPval <- negLogPval[target]
term <- term[target]
p <- plot_ly(x=seq_along(negLogPval), y=~negLogPval,
type="bar", color=~negLogPval, text=term,
colors=c("#4b61ba", "gray", "#a87963", "red"))
layout(p, title="MeSH-Enrichment Analysis (E)",
   xaxis=list(title="Term"),
   yaxis=list(title="-Log(P-value)"),
   legend=list(name=""))
```

```
## Warning: textfont.color doesn't (yet) support data arrays

## Warning: textfont.color doesn't (yet) support data arrays
```

## MeSH-Enrichment Analysis (F : Psychiatry and Psychology)

```
negLogPval <- -log10(out.vecLR[['pattern_5_4']]$Enrich$MeSH_F$Pvalue + 1E-10)
term <- out.vecLR[['pattern_5_4']]$Enrich$MeSH_F$Term
target <- seq_len(min(100, length(negLogPval)))
negLogPval <- negLogPval[target]
term <- term[target]
p <- plot_ly(x=seq_along(negLogPval), y=~negLogPval,
type="bar", color=~negLogPval, text=term,
colors=c("#4b61ba", "gray", "#a87963", "red"))
layout(p, title="MeSH-Enrichment Analysis (F)",
   xaxis=list(title="Term"),
   yaxis=list(title="-Log(P-value)"),
   legend=list(name=""))
```

```
## Warning: textfont.color doesn't (yet) support data arrays

## Warning: textfont.color doesn't (yet) support data arrays
```

## MeSH-Enrichment Analysis (G : Phenomena and Processes)

```
negLogPval <- -log10(out.vecLR[['pattern_5_4']]$Enrich$MeSH_G$Pvalue + 1E-10)
term <- out.vecLR[['pattern_5_4']]$Enrich$MeSH_G$Term
target <- seq_len(min(100, length(negLogPval)))
negLogPval <- negLogPval[target]
term <- term[target]
p <- plot_ly(x=seq_along(negLogPval), y=~negLogPval,
type="bar", color=~negLogPval, text=term,
colors=c("#4b61ba", "gray", "#a87963", "red"))
layout(p, title="MeSH-Enrichment Analysis (G)",
   xaxis=list(title="Term"),
   yaxis=list(title="-Log(P-value)"),
   legend=list(name=""))
```

```
## Warning: textfont.color doesn't (yet) support data arrays

## Warning: textfont.color doesn't (yet) support data arrays
```

## MeSH-Enrichment Analysis (H : Disciplines and Occupations)

```
negLogPval <- -log10(out.vecLR[['pattern_5_4']]$Enrich$MeSH_H$Pvalue + 1E-10)
term <- out.vecLR[['pattern_5_4']]$Enrich$MeSH_H$Term
target <- seq_len(min(100, length(negLogPval)))
negLogPval <- negLogPval[target]
term <- term[target]
p <- plot_ly(x=seq_along(negLogPval), y=~negLogPval,
type="bar", color=~negLogPval, text=term,
colors=c("#4b61ba", "gray", "#a87963", "red"))
layout(p, title="MeSH-Enrichment Analysis (H)",
   xaxis=list(title="Term"),
   yaxis=list(title="-Log(P-value)"),
   legend=list(name=""))
```

```
## Warning: textfont.color doesn't (yet) support data arrays

## Warning: textfont.color doesn't (yet) support data arrays
```

## MeSH-Enrichment Analysis (I : Anthropology, Education, Sociology and Social Phenomena)

```
negLogPval <- -log10(out.vecLR[['pattern_5_4']]$Enrich$MeSH_I$Pvalue + 1E-10)
term <- out.vecLR[['pattern_5_4']]$Enrich$MeSH_I$Term
target <- seq_len(min(100, length(negLogPval)))
negLogPval <- negLogPval[target]
term <- term[target]
p <- plot_ly(x=seq_along(negLogPval), y=~negLogPval,
type="bar", color=~negLogPval, text=term,
colors=c("#4b61ba", "gray", "#a87963", "red"))
layout(p, title="MeSH-Enrichment Analysis (I)",
   xaxis=list(title="Term"),
   yaxis=list(title="-Log(P-value)"),
   legend=list(name=""))
```

```
## Warning: textfont.color doesn't (yet) support data arrays

## Warning: textfont.color doesn't (yet) support data arrays
```

## MeSH-Enrichment Analysis (J : Technology and Food and Beverages)

```
negLogPval <- -log10(out.vecLR[['pattern_5_4']]$Enrich$MeSH_J$Pvalue + 1E-10)
term <- out.vecLR[['pattern_5_4']]$Enrich$MeSH_J$Term
target <- seq_len(min(100, length(negLogPval)))
negLogPval <- negLogPval[target]
term <- term[target]
p <- plot_ly(x=seq_along(negLogPval), y=~negLogPval,
type="bar", color=~negLogPval, text=term,
colors=c("#4b61ba", "gray", "#a87963", "red"))
layout(p, title="MeSH-Enrichment Analysis (J)",
   xaxis=list(title="Term"),
   yaxis=list(title="-Log(P-value)"),
   legend=list(name=""))
```

```
## Warning: textfont.color doesn't (yet) support data arrays

## Warning: textfont.color doesn't (yet) support data arrays
```

## MeSH-Enrichment Analysis (K : Humanities)

```
negLogPval <- -log10(out.vecLR[['pattern_5_4']]$Enrich$MeSH_K$Pvalue + 1E-10)
term <- out.vecLR[['pattern_5_4']]$Enrich$MeSH_K$Term
target <- seq_len(min(100, length(negLogPval)))
negLogPval <- negLogPval[target]
term <- term[target]
p <- plot_ly(x=seq_along(negLogPval), y=~negLogPval,
type="bar", color=~negLogPval, text=term,
colors=c("#4b61ba", "gray", "#a87963", "red"))
layout(p, title="MeSH-Enrichment Analysis (K)",
   xaxis=list(title="Term"),
   yaxis=list(title="-Log(P-value)"),
   legend=list(name=""))
```

## MeSH-Enrichment Analysis (L : Information Science)

```
negLogPval <- -log10(out.vecLR[['pattern_5_4']]$Enrich$MeSH_L$Pvalue + 1E-10)
term <- out.vecLR[['pattern_5_4']]$Enrich$MeSH_L$Term
target <- seq_len(min(100, length(negLogPval)))
negLogPval <- negLogPval[target]
term <- term[target]
p <- plot_ly(x=seq_along(negLogPval), y=~negLogPval,
type="bar", color=~negLogPval, text=term,
colors=c("#4b61ba", "gray", "#a87963", "red"))
layout(p, title="MeSH-Enrichment Analysis (L)",
   xaxis=list(title="Term"),
   yaxis=list(title="-Log(P-value)"),
   legend=list(name=""))
```

```
## Warning: textfont.color doesn't (yet) support data arrays

## Warning: textfont.color doesn't (yet) support data arrays
```

## MeSH-Enrichment Analysis (N : Health Care)

```
negLogPval <- -log10(out.vecLR[['pattern_5_4']]$Enrich$MeSH_N$Pvalue + 1E-10)
term <- out.vecLR[['pattern_5_4']]$Enrich$MeSH_N$Term
target <- seq_len(min(100, length(negLogPval)))
negLogPval <- negLogPval[target]
term <- term[target]
p <- plot_ly(x=seq_along(negLogPval), y=~negLogPval,
type="bar", color=~negLogPval, text=term,
colors=c("#4b61ba", "gray", "#a87963", "red"))
layout(p, title="MeSH-Enrichment Analysis (N)",
   xaxis=list(title="Term"),
   yaxis=list(title="-Log(P-value)"),
   legend=list(name=""))
```

```
## Warning: textfont.color doesn't (yet) support data arrays

## Warning: textfont.color doesn't (yet) support data arrays
```

## Reactome Pathway-Enrichment Analysis

```
negLogPval <- -log10(out.vecLR[['pattern_5_4']]$Enrich$Reactome$Pvalue + 1E-10)
term <- out.vecLR[['pattern_5_4']]$Enrich$Reactome$Term
target <- seq_len(min(100, length(negLogPval)))
negLogPval <- negLogPval[target]
term <- term[target]
p <- plot_ly(x=seq_along(negLogPval), y=~negLogPval,
type="bar", color=~negLogPval, text=term,
colors=c("#4b61ba", "gray", "#a87963", "red"))
layout(p, title="Reactome-Enrichment Analysis",
   xaxis=list(title="Term"),
   yaxis=list(title="-Log(P-value)"),
   legend=list(name=""))
```

```
## Warning: textfont.color doesn't (yet) support data arrays

## Warning: textfont.color doesn't (yet) support data arrays
```
