# Supplementary material for: Sctensor detects many-to-many cell–cell interactions from single cell RNA-sequencing data
Source: BMC Bioinformatics. 2023 Nov 7;24:420. doi: 10.1186/s12859-023-05490-y (PMC10631077; doi:10.1186/s12859-023-05490-y)
Supplement: Supplementary file 18 — Additional file 18. HTML report of VisualCortex. [file 12859_2023_5490_MOESM18_ESM.zip › output/report/Mouse_VisualCortex/pattern_5_3.html]

Details of (5,3,) -related L-R pairs


# Details of (5,3,) -related L-R pairs

# (5,3,:) Pattern

# (5,3,) -related L-R pairs (Top10 pairs)

```
library("scTensor")
load("reanalysis.RData")
col <- .setColor("many")[19]
scTensor:::.myvisNetwork(g, col)
```

| Rank | Ligand Gene | Receptor Gene | Ligand Expression (Log10(exp + 1)) | Receptor Expression (Log10(exp + 1)) | LR-pair factor value (Percentage) | P-value (Grubbs test) | Q-value (BH method) | L-R Evidence cf. rikenbit/lrbase-workflow | PubMed |
| --- | --- | --- | --- | --- | --- | --- | --- | --- | --- |
| 1 | Fgf12 Description: Fgf12fibroblast growth factor 12 GO: 1-18 Reactome: 1-3 UniProtKB: 1-2 RefEx: Fgf12 Expression Atlas: Fgf12 Single Cell Expression Atlas: Fgf12 PanglaoDB: Fgf12 MeSH: 1-86 87-172 173-258 259-341 | Fgfr2 Description: Fgf12fibroblast growth factor receptor 2 GO: 1-66 67-131 Reactome: 1-27 UniProtKB: 1-4 RefEx: Fgf12 Expression Atlas: Fgfr2 Single Cell Expression Atlas: Fgfr2 PanglaoDB: Fgfr2 MeSH: 1-100 101-200 201-300 301-400 401-500 501-600 601-700 701-800 801-900 901-1000 1001-1100 1101-1200 1201-1300 1301-1400 1401-1500 1501-1600 1601-1700 1701-1791 |  |  | 0.094 (19.976%) | 0 | 0 | DLRP | 1 |
| 2 | Fgf13 Description: Fgf13fibroblast growth factor 13 GO: 1-36 Reactome: 1-3 UniProtKB: 1 RefEx: Fgf13 Expression Atlas: Fgf13 Single Cell Expression Atlas: Fgf13 PanglaoDB: Fgf13 MeSH: 1-92 93-184 185-276 277-365 | Fgfr2 Description: Fgf13fibroblast growth factor receptor 2 GO: 1-66 67-131 Reactome: 1-27 UniProtKB: 1-4 RefEx: Fgf13 Expression Atlas: Fgfr2 Single Cell Expression Atlas: Fgfr2 PanglaoDB: Fgfr2 MeSH: 1-100 101-200 201-300 301-400 401-500 501-600 601-700 701-800 801-900 901-1000 1001-1100 1101-1200 1201-1300 1301-1400 1401-1500 1501-1600 1601-1700 1701-1791 |  |  | 0.075 (15.974%) | 0 | 0 | DLRP | 1 |
| 3 | Fgf9 Description: Fgf9fibroblast growth factor 9 GO: 1-48 Reactome: 1-53 UniProtKB: 1 RefEx: Fgf9 Expression Atlas: Fgf9 Single Cell Expression Atlas: Fgf9 PanglaoDB: Fgf9 MeSH: 1-96 97-192 193-288 289-384 385-480 481-576 577-672 673-768 769-864 865-958 | Fgfr2 Description: Fgf9fibroblast growth factor receptor 2 GO: 1-66 67-131 Reactome: 1-27 UniProtKB: 1-4 RefEx: Fgf9 Expression Atlas: Fgfr2 Single Cell Expression Atlas: Fgfr2 PanglaoDB: Fgfr2 MeSH: 1-100 101-200 201-300 301-400 401-500 501-600 601-700 701-800 801-900 901-1000 1001-1100 1101-1200 1201-1300 1301-1400 1401-1500 1501-1600 1601-1700 1701-1791 |  |  | 0.052 (11.067%) | 0 | 0 | DLRP | 1 |
| 4 | Fgf12 Description: Fgf12fibroblast growth factor 12 GO: 1-18 Reactome: 1-3 UniProtKB: 1-2 RefEx: Fgf12 Expression Atlas: Fgf12 Single Cell Expression Atlas: Fgf12 PanglaoDB: Fgf12 MeSH: 1-86 87-172 173-258 259-341 | Fgfr3 Description: Fgf12fibroblast growth factor receptor 3 GO: 1-91 UniProtKB: 1-4 RefEx: Fgf12 Expression Atlas: Fgfr3 Single Cell Expression Atlas: Fgfr3 PanglaoDB: Fgfr3 MeSH: 1-97 98-194 195-291 292-388 389-485 486-582 583-679 680-776 777-873 874-970 971-1067 1068-1164 1165-1261 1262-1358 1359-1454 |  |  | 0.036 (7.701%) | 0 | 0 | DLRP | 1 |
| 5 | Fgf13 Description: Fgf13fibroblast growth factor 13 GO: 1-36 Reactome: 1-3 UniProtKB: 1 RefEx: Fgf13 Expression Atlas: Fgf13 Single Cell Expression Atlas: Fgf13 PanglaoDB: Fgf13 MeSH: 1-92 93-184 185-276 277-365 | Fgfr3 Description: Fgf13fibroblast growth factor receptor 3 GO: 1-91 UniProtKB: 1-4 RefEx: Fgf13 Expression Atlas: Fgfr3 Single Cell Expression Atlas: Fgfr3 PanglaoDB: Fgfr3 MeSH: 1-97 98-194 195-291 292-388 389-485 486-582 583-679 680-776 777-873 874-970 971-1067 1068-1164 1165-1261 1262-1358 1359-1454 |  |  | 0.029 (6.158%) | 0 | 0 | DLRP | 1 |
| 6 | Fgf11 Description: Fgf11fibroblast growth factor 11 GO: 1-6 Reactome: 1-3 UniProtKB: 1-2 RefEx: Fgf11 Expression Atlas: Fgf11 Single Cell Expression Atlas: Fgf11 PanglaoDB: Fgf11 MeSH: 1-68 69-136 137-203 | Fgfr2 Description: Fgf11fibroblast growth factor receptor 2 GO: 1-66 67-131 Reactome: 1-27 UniProtKB: 1-4 RefEx: Fgf11 Expression Atlas: Fgfr2 Single Cell Expression Atlas: Fgfr2 PanglaoDB: Fgfr2 MeSH: 1-100 101-200 201-300 301-400 401-500 501-600 601-700 701-800 801-900 901-1000 1001-1100 1101-1200 1201-1300 1301-1400 1401-1500 1501-1600 1601-1700 1701-1791 |  |  | 0.023 (4.961%) | 0 | 0 | DLRP | 1 |
| 7 | Efnb3 Description: Efnb3ephrin B3 GO: 1-18 Reactome: 1-7 UniProtKB: 1-2 STRING: 1 RefEx: Efnb3 Expression Atlas: Efnb3 Single Cell Expression Atlas: Efnb3 PanglaoDB: Efnb3 MeSH: 1-89 90-178 179-267 268-356 357-445 446-534 535-622 | Ephb1 Description: Efnb3Eph receptor B1 GO: 1-62 Reactome: 1-7 UniProtKB: 1-2 RefEx: Efnb3 Expression Atlas: Ephb1 Single Cell Expression Atlas: Ephb1 PanglaoDB: Ephb1 MeSH: 1-100 101-200 201-300 301-400 401-500 501-600 601-695 |  |  | 0.023 (4.873%) | 0 | 0 | DLRP | 1 |
| 8 | Efna3 Description: Efna3ephrin A3 GO: 1-8 Reactome: 1-6 UniProtKB: 1 RefEx: Efna3 Expression Atlas: Efna3 Single Cell Expression Atlas: Efna3 PanglaoDB: Efna3 MeSH: 1-84 85-168 169-252 253-336 337-419 | Ephb1 Description: Efna3Eph receptor B1 GO: 1-62 Reactome: 1-7 UniProtKB: 1-2 RefEx: Efna3 Expression Atlas: Ephb1 Single Cell Expression Atlas: Ephb1 PanglaoDB: Ephb1 MeSH: 1-100 101-200 201-300 301-400 401-500 501-600 601-695 |  |  | 0.02 (4.334%) | 0 | 0 | DLRP | 1 |
| 9 | Sema5a Description: Sema5asema domain, seven thrombospondin repeats (type 1 and type 1-like), transmembrane domain (TM) and short cytoplasmic domain, (semaphorin) 5A GO: 1-38 UniProtKB: 1 RefEx: Sema5a Expression Atlas: Sema5a Single Cell Expression Atlas: Sema5a PanglaoDB: Sema5a MeSH: 1-88 89-176 177-264 265-350 | Plxnb3 Description: Sema5aplexin B3 GO: 1-28 Reactome: 1-5 UniProtKB: 1 RefEx: Sema5a Expression Atlas: Plxnb3 Single Cell Expression Atlas: Plxnb3 PanglaoDB: Plxnb3 MeSH: 1-73 74-146 147-218 |  |  | 0.02 (4.277%) | 0 | 0 | DLRP | 1 |
| 10 | Fgf9 Description: Fgf9fibroblast growth factor 9 GO: 1-48 Reactome: 1-53 UniProtKB: 1 RefEx: Fgf9 Expression Atlas: Fgf9 Single Cell Expression Atlas: Fgf9 PanglaoDB: Fgf9 MeSH: 1-96 97-192 193-288 289-384 385-480 481-576 577-672 673-768 769-864 865-958 | Fgfr3 Description: Fgf9fibroblast growth factor receptor 3 GO: 1-91 UniProtKB: 1-4 RefEx: Fgf9 Expression Atlas: Fgfr3 Single Cell Expression Atlas: Fgfr3 PanglaoDB: Fgfr3 MeSH: 1-97 98-194 195-291 292-388 389-485 486-582 583-679 680-776 777-873 874-970 971-1067 1068-1164 1165-1261 1262-1358 1359-1454 |  |  | 0.02 (4.266%) | 0 | 0 | DLRP | 1 |

# (5,3,) -related Enrichment Analysis

```
load("reanalysis.RData")
library("plotly")
```

## MeSH-Enrichment Analysis (A : Anatomy)

```
negLogPval <- -log10(out.vecLR[['pattern_5_3']]$Enrich$MeSH_A$Pvalue + 1E-10)
term <- out.vecLR[['pattern_5_3']]$Enrich$MeSH_A$Term
target <- seq_len(min(100, length(negLogPval)))
negLogPval <- negLogPval[target]
term <- term[target]
p <- plot_ly(x=seq_along(negLogPval), y=~negLogPval,
type="bar", color=~negLogPval, text=term,
colors=c("#4b61ba", "gray", "#a87963", "red"))
layout(p, title="MeSH-Enrichment Analysis (A)",
   xaxis=list(title="Term"),
   yaxis=list(title="-Log(P-value)"),
   legend=list(name=""))
```

```
## Warning: textfont.color doesn't (yet) support data arrays

## Warning: textfont.color doesn't (yet) support data arrays
```

## MeSH-Enrichment Analysis (B : Organisms)

```
negLogPval <- -log10(out.vecLR[['pattern_5_3']]$Enrich$MeSH_B$Pvalue + 1E-10)
term <- out.vecLR[['pattern_5_3']]$Enrich$MeSH_B$Term
target <- seq_len(min(100, length(negLogPval)))
negLogPval <- negLogPval[target]
term <- term[target]
p <- plot_ly(x=seq_along(negLogPval), y=~negLogPval,
type="bar", color=~negLogPval, text=term,
colors=c("#4b61ba", "gray", "#a87963", "red"))
layout(p, title="MeSH-Enrichment Analysis (B)",
   xaxis=list(title="Term"),
   yaxis=list(title="-Log(P-value)"),
   legend=list(name=""))
```

```
## Warning: textfont.color doesn't (yet) support data arrays

## Warning: textfont.color doesn't (yet) support data arrays
```

## MeSH-Enrichment Analysis (C : Diseases)

```
negLogPval <- -log10(out.vecLR[['pattern_5_3']]$Enrich$MeSH_C$Pvalue + 1E-10)
term <- out.vecLR[['pattern_5_3']]$Enrich$MeSH_C$Term
target <- seq_len(min(100, length(negLogPval)))
negLogPval <- negLogPval[target]
term <- term[target]
p <- plot_ly(x=seq_along(negLogPval), y=~negLogPval,
type="bar", color=~negLogPval, text=term,
colors=c("#4b61ba", "gray", "#a87963", "red"))
layout(p, title="MeSH-Enrichment Analysis (C)",
   xaxis=list(title="Term"),
   yaxis=list(title="-Log(P-value)"),
   legend=list(name=""))
```

```
## Warning: textfont.color doesn't (yet) support data arrays

## Warning: textfont.color doesn't (yet) support data arrays
```

## MeSH-Enrichment Analysis (D : Drugs)

```
negLogPval <- -log10(out.vecLR[['pattern_5_3']]$Enrich$MeSH_D$Pvalue + 1E-10)
term <- out.vecLR[['pattern_5_3']]$Enrich$MeSH_D$Term
target <- seq_len(min(100, length(negLogPval)))
negLogPval <- negLogPval[target]
term <- term[target]
p <- plot_ly(x=seq_along(negLogPval), y=~negLogPval,
type="bar", color=~negLogPval, text=term,
colors=c("#4b61ba", "gray", "#a87963", "red"))
layout(p, title="MeSH-Enrichment Analysis (D)",
   xaxis=list(title="Term"),
   yaxis=list(title="-Log(P-value)"),
   legend=list(name=""))
```

```
## Warning: textfont.color doesn't (yet) support data arrays

## Warning: textfont.color doesn't (yet) support data arrays
```

## MeSH-Enrichment Analysis (E : Analytical, Diagnostic and Therapeutic Techniques and Equipment)

```
negLogPval <- -log10(out.vecLR[['pattern_5_3']]$Enrich$MeSH_E$Pvalue + 1E-10)
term <- out.vecLR[['pattern_5_3']]$Enrich$MeSH_E$Term
target <- seq_len(min(100, length(negLogPval)))
negLogPval <- negLogPval[target]
term <- term[target]
p <- plot_ly(x=seq_along(negLogPval), y=~negLogPval,
type="bar", color=~negLogPval, text=term,
colors=c("#4b61ba", "gray", "#a87963", "red"))
layout(p, title="MeSH-Enrichment Analysis (E)",
   xaxis=list(title="Term"),
   yaxis=list(title="-Log(P-value)"),
   legend=list(name=""))
```

```
## Warning: textfont.color doesn't (yet) support data arrays

## Warning: textfont.color doesn't (yet) support data arrays
```

## MeSH-Enrichment Analysis (F : Psychiatry and Psychology)

```
negLogPval <- -log10(out.vecLR[['pattern_5_3']]$Enrich$MeSH_F$Pvalue + 1E-10)
term <- out.vecLR[['pattern_5_3']]$Enrich$MeSH_F$Term
target <- seq_len(min(100, length(negLogPval)))
negLogPval <- negLogPval[target]
term <- term[target]
p <- plot_ly(x=seq_along(negLogPval), y=~negLogPval,
type="bar", color=~negLogPval, text=term,
colors=c("#4b61ba", "gray", "#a87963", "red"))
layout(p, title="MeSH-Enrichment Analysis (F)",
   xaxis=list(title="Term"),
   yaxis=list(title="-Log(P-value)"),
   legend=list(name=""))
```

```
## Warning: textfont.color doesn't (yet) support data arrays

## Warning: textfont.color doesn't (yet) support data arrays
```

## MeSH-Enrichment Analysis (G : Phenomena and Processes)

```
negLogPval <- -log10(out.vecLR[['pattern_5_3']]$Enrich$MeSH_G$Pvalue + 1E-10)
term <- out.vecLR[['pattern_5_3']]$Enrich$MeSH_G$Term
target <- seq_len(min(100, length(negLogPval)))
negLogPval <- negLogPval[target]
term <- term[target]
p <- plot_ly(x=seq_along(negLogPval), y=~negLogPval,
type="bar", color=~negLogPval, text=term,
colors=c("#4b61ba", "gray", "#a87963", "red"))
layout(p, title="MeSH-Enrichment Analysis (G)",
   xaxis=list(title="Term"),
   yaxis=list(title="-Log(P-value)"),
   legend=list(name=""))
```

```
## Warning: textfont.color doesn't (yet) support data arrays

## Warning: textfont.color doesn't (yet) support data arrays
```

## MeSH-Enrichment Analysis (I : Anthropology, Education, Sociology and Social Phenomena)

```
negLogPval <- -log10(out.vecLR[['pattern_5_3']]$Enrich$MeSH_I$Pvalue + 1E-10)
term <- out.vecLR[['pattern_5_3']]$Enrich$MeSH_I$Term
target <- seq_len(min(100, length(negLogPval)))
negLogPval <- negLogPval[target]
term <- term[target]
p <- plot_ly(x=seq_along(negLogPval), y=~negLogPval,
type="bar", color=~negLogPval, text=term,
colors=c("#4b61ba", "gray", "#a87963", "red"))
layout(p, title="MeSH-Enrichment Analysis (I)",
   xaxis=list(title="Term"),
   yaxis=list(title="-Log(P-value)"),
   legend=list(name=""))
```

```
## Warning: textfont.color doesn't (yet) support data arrays

## Warning: textfont.color doesn't (yet) support data arrays
```

## MeSH-Enrichment Analysis (L : Information Science)

```
negLogPval <- -log10(out.vecLR[['pattern_5_3']]$Enrich$MeSH_L$Pvalue + 1E-10)
term <- out.vecLR[['pattern_5_3']]$Enrich$MeSH_L$Term
target <- seq_len(min(100, length(negLogPval)))
negLogPval <- negLogPval[target]
term <- term[target]
p <- plot_ly(x=seq_along(negLogPval), y=~negLogPval,
type="bar", color=~negLogPval, text=term,
colors=c("#4b61ba", "gray", "#a87963", "red"))
layout(p, title="MeSH-Enrichment Analysis (L)",
   xaxis=list(title="Term"),
   yaxis=list(title="-Log(P-value)"),
   legend=list(name=""))
```

```
## Warning: textfont.color doesn't (yet) support data arrays

## Warning: textfont.color doesn't (yet) support data arrays
```

## Reactome Pathway-Enrichment Analysis

```
negLogPval <- -log10(out.vecLR[['pattern_5_3']]$Enrich$Reactome$Pvalue + 1E-10)
term <- out.vecLR[['pattern_5_3']]$Enrich$Reactome$Term
target <- seq_len(min(100, length(negLogPval)))
negLogPval <- negLogPval[target]
term <- term[target]
p <- plot_ly(x=seq_along(negLogPval), y=~negLogPval,
type="bar", color=~negLogPval, text=term,
colors=c("#4b61ba", "gray", "#a87963", "red"))
layout(p, title="Reactome-Enrichment Analysis",
   xaxis=list(title="Term"),
   yaxis=list(title="-Log(P-value)"),
   legend=list(name=""))
```

```
## Warning: textfont.color doesn't (yet) support data arrays

## Warning: textfont.color doesn't (yet) support data arrays
```
