# Supplementary material for: Sctensor detects many-to-many cell–cell interactions from single cell RNA-sequencing data
Source: BMC Bioinformatics. 2023 Nov 7;24:420. doi: 10.1186/s12859-023-05490-y (PMC10631077; doi:10.1186/s12859-023-05490-y)
Supplement: Supplementary file 18 — Additional file 18. HTML report of VisualCortex. [file 12859_2023_5490_MOESM18_ESM.zip › output/report/Mouse_VisualCortex/pattern_4_4.html]

Details of (4,4,) -related L-R pairs


# Details of (4,4,) -related L-R pairs

# (4,4,:) Pattern

# (4,4,) -related L-R pairs (Top15 pairs)

```
library("scTensor")
load("reanalysis.RData")
col <- .setColor("many")[20]
scTensor:::.myvisNetwork(g, col)
```

| Rank | Ligand Gene | Receptor Gene | Ligand Expression (Log10(exp + 1)) | Receptor Expression (Log10(exp + 1)) | LR-pair factor value (Percentage) | P-value (Grubbs test) | Q-value (BH method) | L-R Evidence cf. rikenbit/lrbase-workflow | PubMed |
| --- | --- | --- | --- | --- | --- | --- | --- | --- | --- |
| 1 | Cck Description: Cckcholecystokinin GO: 1-35 Reactome: 1-7 UniProtKB: 1-2 STRING: 1 2 3 4 5 6 RefEx: Cck Expression Atlas: Cck Single Cell Expression Atlas: Cck PanglaoDB: Cck MeSH: 1-99 100-198 199-297 298-396 397-495 496-594 595-693 694-792 793-891 892-990 991-1089 1090-1188 1189-1287 1288-1386 1387-1485 1486-1584 1585-1683 1684-1782 1783-1881 1882-1980 1981-2079 2080-2178 2179-2277 2278-2376 2377-2475 2476-2574 2575-2673 2674-2772 2773-2871 2872-2970 2971-3069 3070-3168 3169-3267 3268-3340 | Cckbr Description: Cckcholecystokinin B receptor GO: 1-32 Reactome: 1-8 UniProtKB: 1-2 RefEx: Cck Expression Atlas: Cckbr Single Cell Expression Atlas: Cckbr PanglaoDB: Cckbr MeSH: 1-99 100-198 199-297 298-396 397-495 496-589 |  |  | 0.053 (11.43%) | 0 | 0 | DLRP | 1 |
| 2 | Efnb3 Description: Efnb3ephrin B3 GO: 1-18 Reactome: 1-7 UniProtKB: 1-2 STRING: 1 RefEx: Efnb3 Expression Atlas: Efnb3 Single Cell Expression Atlas: Efnb3 PanglaoDB: Efnb3 MeSH: 1-89 90-178 179-267 268-356 357-445 446-534 535-622 | Epha4 Description: Efnb3Eph receptor A4 GO: 1-90 Reactome: 1-6 UniProtKB: 1 RefEx: Efnb3 Expression Atlas: Epha4 Single Cell Expression Atlas: Epha4 PanglaoDB: Epha4 MeSH: 1-95 96-190 191-285 286-380 381-475 476-570 571-665 666-760 761-855 856-950 951-1045 1046-1131 |  |  | 0.043 (9.212%) | 0 | 0 | DLRP | 1 |
| 3 | Nlgn3 Description: Nlgn3neuroligin 3 GO: 1-59 Reactome: 1-3 UniProtKB: 1 RefEx: Nlgn3 Expression Atlas: Nlgn3 Single Cell Expression Atlas: Nlgn3 PanglaoDB: Nlgn3 MeSH: 1-82 83-164 165-246 247-328 329-408 | Nrxn3 Description: Nlgn3neurexin III GO: 1-18 Reactome: 1-3 UniProtKB: 1-2 RefEx: Nlgn3 Expression Atlas: Nrxn3 Single Cell Expression Atlas: Nrxn3 PanglaoDB: Nrxn3 MeSH: 1-78 79-156 157-234 235-311 |  |  | 0.039 (8.305%) | 0 | 0 | DLRP | 1 |
| 4 | Sema5a Description: Sema5asema domain, seven thrombospondin repeats (type 1 and type 1-like), transmembrane domain (TM) and short cytoplasmic domain, (semaphorin) 5A GO: 1-38 UniProtKB: 1 RefEx: Sema5a Expression Atlas: Sema5a Single Cell Expression Atlas: Sema5a PanglaoDB: Sema5a MeSH: 1-88 89-176 177-264 265-350 | Plxnb3 Description: Sema5aplexin B3 GO: 1-28 Reactome: 1-5 UniProtKB: 1 RefEx: Sema5a Expression Atlas: Plxnb3 Single Cell Expression Atlas: Plxnb3 PanglaoDB: Plxnb3 MeSH: 1-73 74-146 147-218 |  |  | 0.028 (6.087%) | 0 | 0 | DLRP | 1 |
| 5 | Vip Description: Vipvasoactive intestinal polypeptide GO: 1-29 Reactome: 1-7 UniProtKB: 1-2 STRING: 1 RefEx: Vip Expression Atlas: Vip Single Cell Expression Atlas: Vip PanglaoDB: Vip MeSH: 1-100 101-200 201-300 301-400 401-500 501-600 601-700 701-800 801-900 901-1000 1001-1100 1101-1200 1201-1300 1301-1400 1401-1500 1501-1600 1601-1700 1701-1800 1801-1900 1901-2000 2001-2100 2101-2200 2201-2300 2301-2400 2401-2500 2501-2600 2601-2700 2701-2800 2801-2900 2901-3000 3001-3100 3101-3200 3201-3274 | Vipr1 Description: Vipvasoactive intestinal peptide receptor 1 GO: 1-14 Reactome: 1-7 UniProtKB: 1 STRING: 1 RefEx: Vip Expression Atlas: Vipr1 Single Cell Expression Atlas: Vipr1 PanglaoDB: Vipr1 MeSH: 1-98 99-196 197-294 295-390 |  |  | 0.027 (5.715%) | 0.003 | 0.003 | DLRP | 1 |
| 6 | Efna2 Description: Efna2ephrin A2 GO: 1-11 Reactome: 1-6 UniProtKB: 1-2 STRING: 1 RefEx: Efna2 Expression Atlas: Efna2 Single Cell Expression Atlas: Efna2 PanglaoDB: Efna2 MeSH: 1-89 90-178 179-267 268-356 357-445 446-531 | Epha4 Description: Efna2Eph receptor A4 GO: 1-90 Reactome: 1-6 UniProtKB: 1 RefEx: Efna2 Expression Atlas: Epha4 Single Cell Expression Atlas: Epha4 PanglaoDB: Epha4 MeSH: 1-95 96-190 191-285 286-380 381-475 476-570 571-665 666-760 761-855 856-950 951-1045 1046-1131 |  |  | 0.026 (5.571%) | 0 | 0 | DLRP | 1 |
| 7 | Efna5 Description: Efna5ephrin A5 GO: 1-34 Reactome: 1-6 UniProtKB: 1 RefEx: Efna5 Expression Atlas: Efna5 Single Cell Expression Atlas: Efna5 PanglaoDB: Efna5 MeSH: 1-90 91-180 181-270 271-360 361-450 451-540 541-630 631-718 | Epha4 Description: Efna5Eph receptor A4 GO: 1-90 Reactome: 1-6 UniProtKB: 1 RefEx: Efna5 Expression Atlas: Epha4 Single Cell Expression Atlas: Epha4 PanglaoDB: Epha4 MeSH: 1-95 96-190 191-285 286-380 381-475 476-570 571-665 666-760 761-855 856-950 951-1045 1046-1131 |  |  | 0.025 (5.407%) | 0 | 0 | DLRP | 1 |
| 8 | Efnb2 Description: Efnb2ephrin B2 GO: 1-35 Reactome: 1-7 UniProtKB: 1-2 RefEx: Efnb2 Expression Atlas: Efnb2 Single Cell Expression Atlas: Efnb2 PanglaoDB: Efnb2 MeSH: 1-94 95-188 189-282 283-376 377-470 471-564 565-658 659-752 753-846 847-940 941-1034 1035-1125 | Epha4 Description: Efnb2Eph receptor A4 GO: 1-90 Reactome: 1-6 UniProtKB: 1 RefEx: Efnb2 Expression Atlas: Epha4 Single Cell Expression Atlas: Epha4 PanglaoDB: Epha4 MeSH: 1-95 96-190 191-285 286-380 381-475 476-570 571-665 666-760 761-855 856-950 951-1045 1046-1131 |  |  | 0.021 (4.418%) | 0 | 0 | DLRP | 1 |
| 9 | Efna3 Description: Efna3ephrin A3 GO: 1-8 Reactome: 1-6 UniProtKB: 1 RefEx: Efna3 Expression Atlas: Efna3 Single Cell Expression Atlas: Efna3 PanglaoDB: Efna3 MeSH: 1-84 85-168 169-252 253-336 337-419 | Epha4 Description: Efna3Eph receptor A4 GO: 1-90 Reactome: 1-6 UniProtKB: 1 RefEx: Efna3 Expression Atlas: Epha4 Single Cell Expression Atlas: Epha4 PanglaoDB: Epha4 MeSH: 1-95 96-190 191-285 286-380 381-475 476-570 571-665 666-760 761-855 856-950 951-1045 1046-1131 |  |  | 0.02 (4.218%) | 0 | 0 | DLRP | 1 |
| 10 | Efna2 Description: Efna2ephrin A2 GO: 1-11 Reactome: 1-6 UniProtKB: 1-2 STRING: 1 RefEx: Efna2 Expression Atlas: Efna2 Single Cell Expression Atlas: Efna2 PanglaoDB: Efna2 MeSH: 1-89 90-178 179-267 268-356 357-445 446-531 | Epha5 Description: Efna2Eph receptor A5 GO: 1-45 Reactome: 1-6 UniProtKB: 1-2 RefEx: Efna2 Expression Atlas: Epha5 Single Cell Expression Atlas: Epha5 PanglaoDB: Epha5 MeSH: 1-98 99-196 197-294 295-392 393-489 |  |  | 0.019 (3.994%) | 0 | 0 | DLRP | 1 |
| 11 | Efna5 Description: Efna5ephrin A5 GO: 1-34 Reactome: 1-6 UniProtKB: 1 RefEx: Efna5 Expression Atlas: Efna5 Single Cell Expression Atlas: Efna5 PanglaoDB: Efna5 MeSH: 1-90 91-180 181-270 271-360 361-450 451-540 541-630 631-718 | Epha5 Description: Efna5Eph receptor A5 GO: 1-45 Reactome: 1-6 UniProtKB: 1-2 RefEx: Efna5 Expression Atlas: Epha5 Single Cell Expression Atlas: Epha5 PanglaoDB: Epha5 MeSH: 1-98 99-196 197-294 295-392 393-489 |  |  | 0.018 (3.876%) | 0 | 0 | DLRP | 1 |
| 12 | Efna3 Description: Efna3ephrin A3 GO: 1-8 Reactome: 1-6 UniProtKB: 1 RefEx: Efna3 Expression Atlas: Efna3 Single Cell Expression Atlas: Efna3 PanglaoDB: Efna3 MeSH: 1-84 85-168 169-252 253-336 337-419 | Epha5 Description: Efna3Eph receptor A5 GO: 1-45 Reactome: 1-6 UniProtKB: 1-2 RefEx: Efna3 Expression Atlas: Epha5 Single Cell Expression Atlas: Epha5 PanglaoDB: Epha5 MeSH: 1-98 99-196 197-294 295-392 393-489 |  |  | 0.014 (3.024%) | 0 | 0 | DLRP | 1 |
| 13 | Sema3e Description: Sema3esema domain, immunoglobulin domain (Ig), short basic domain, secreted, (semaphorin) 3E GO: 1-23 Reactome: 1-5 UniProtKB: 1 STRING: 1 RefEx: Sema3e Expression Atlas: Sema3e Single Cell Expression Atlas: Sema3e PanglaoDB: Sema3e MeSH: 1-92 93-184 185-276 277-368 369-460 461-550 | Plxnd1 Description: Sema3eplexin D1 GO: 1-31 Reactome: 1-10 UniProtKB: 1 RefEx: Sema3e Expression Atlas: Plxnd1 Single Cell Expression Atlas: Plxnd1 PanglaoDB: Plxnd1 MeSH: 1-85 86-170 171-255 256-340 341-425 426-508 |  |  | 0.013 (2.7%) | 0 | 0 | DLRP | 1 |
| 14 | Efnb3 Description: Efnb3ephrin B3 GO: 1-18 Reactome: 1-7 UniProtKB: 1-2 STRING: 1 RefEx: Efnb3 Expression Atlas: Efnb3 Single Cell Expression Atlas: Efnb3 PanglaoDB: Efnb3 MeSH: 1-89 90-178 179-267 268-356 357-445 446-534 535-622 | Ephb6 Description: Efnb3Eph receptor B6 GO: 1-26 Reactome: 1-7 UniProtKB: 1 STRING: 1 2 3 RefEx: Efnb3 Expression Atlas: Ephb6 Single Cell Expression Atlas: Ephb6 PanglaoDB: Ephb6 MeSH: 1-82 83-164 165-246 247-328 329-406 |  |  | 0.013 (2.684%) | 0 | 0 | DLRP | 1 |
| 15 | Nxph1 Description: Nxph1neurexophilin 1 GO: 1-2 UniProtKB: 1 STRING: 1 2 RefEx: Nxph1 Expression Atlas: Nxph1 Single Cell Expression Atlas: Nxph1 PanglaoDB: Nxph1 MeSH: 1-91 92-181 | Nrxn3 Description: Nxph1neurexin III GO: 1-18 Reactome: 1-3 UniProtKB: 1-2 RefEx: Nxph1 Expression Atlas: Nrxn3 Single Cell Expression Atlas: Nrxn3 PanglaoDB: Nrxn3 MeSH: 1-78 79-156 157-234 235-311 |  |  | 0.01 (2.085%) | 0 | 0 | DLRP | 1 |

# (4,4,) -related Enrichment Analysis

```
load("reanalysis.RData")
library("plotly")
```

## MeSH-Enrichment Analysis (A : Anatomy)

```
negLogPval <- -log10(out.vecLR[['pattern_4_4']]$Enrich$MeSH_A$Pvalue + 1E-10)
term <- out.vecLR[['pattern_4_4']]$Enrich$MeSH_A$Term
target <- seq_len(min(100, length(negLogPval)))
negLogPval <- negLogPval[target]
term <- term[target]
p <- plot_ly(x=seq_along(negLogPval), y=~negLogPval,
type="bar", color=~negLogPval, text=term,
colors=c("#4b61ba", "gray", "#a87963", "red"))
layout(p, title="MeSH-Enrichment Analysis (A)",
   xaxis=list(title="Term"),
   yaxis=list(title="-Log(P-value)"),
   legend=list(name=""))
```

```
## Warning: textfont.color doesn't (yet) support data arrays

## Warning: textfont.color doesn't (yet) support data arrays
```

## MeSH-Enrichment Analysis (B : Organisms)

```
negLogPval <- -log10(out.vecLR[['pattern_4_4']]$Enrich$MeSH_B$Pvalue + 1E-10)
term <- out.vecLR[['pattern_4_4']]$Enrich$MeSH_B$Term
target <- seq_len(min(100, length(negLogPval)))
negLogPval <- negLogPval[target]
term <- term[target]
p <- plot_ly(x=seq_along(negLogPval), y=~negLogPval,
type="bar", color=~negLogPval, text=term,
colors=c("#4b61ba", "gray", "#a87963", "red"))
layout(p, title="MeSH-Enrichment Analysis (B)",
   xaxis=list(title="Term"),
   yaxis=list(title="-Log(P-value)"),
   legend=list(name=""))
```

```
## Warning: textfont.color doesn't (yet) support data arrays

## Warning: textfont.color doesn't (yet) support data arrays
```

## MeSH-Enrichment Analysis (C : Diseases)

```
negLogPval <- -log10(out.vecLR[['pattern_4_4']]$Enrich$MeSH_C$Pvalue + 1E-10)
term <- out.vecLR[['pattern_4_4']]$Enrich$MeSH_C$Term
target <- seq_len(min(100, length(negLogPval)))
negLogPval <- negLogPval[target]
term <- term[target]
p <- plot_ly(x=seq_along(negLogPval), y=~negLogPval,
type="bar", color=~negLogPval, text=term,
colors=c("#4b61ba", "gray", "#a87963", "red"))
layout(p, title="MeSH-Enrichment Analysis (C)",
   xaxis=list(title="Term"),
   yaxis=list(title="-Log(P-value)"),
   legend=list(name=""))
```

```
## Warning: textfont.color doesn't (yet) support data arrays

## Warning: textfont.color doesn't (yet) support data arrays
```

## MeSH-Enrichment Analysis (D : Drugs)

```
negLogPval <- -log10(out.vecLR[['pattern_4_4']]$Enrich$MeSH_D$Pvalue + 1E-10)
term <- out.vecLR[['pattern_4_4']]$Enrich$MeSH_D$Term
target <- seq_len(min(100, length(negLogPval)))
negLogPval <- negLogPval[target]
term <- term[target]
p <- plot_ly(x=seq_along(negLogPval), y=~negLogPval,
type="bar", color=~negLogPval, text=term,
colors=c("#4b61ba", "gray", "#a87963", "red"))
layout(p, title="MeSH-Enrichment Analysis (D)",
   xaxis=list(title="Term"),
   yaxis=list(title="-Log(P-value)"),
   legend=list(name=""))
```

```
## Warning: textfont.color doesn't (yet) support data arrays

## Warning: textfont.color doesn't (yet) support data arrays
```

## MeSH-Enrichment Analysis (E : Analytical, Diagnostic and Therapeutic Techniques and Equipment)

```
negLogPval <- -log10(out.vecLR[['pattern_4_4']]$Enrich$MeSH_E$Pvalue + 1E-10)
term <- out.vecLR[['pattern_4_4']]$Enrich$MeSH_E$Term
target <- seq_len(min(100, length(negLogPval)))
negLogPval <- negLogPval[target]
term <- term[target]
p <- plot_ly(x=seq_along(negLogPval), y=~negLogPval,
type="bar", color=~negLogPval, text=term,
colors=c("#4b61ba", "gray", "#a87963", "red"))
layout(p, title="MeSH-Enrichment Analysis (E)",
   xaxis=list(title="Term"),
   yaxis=list(title="-Log(P-value)"),
   legend=list(name=""))
```

```
## Warning: textfont.color doesn't (yet) support data arrays

## Warning: textfont.color doesn't (yet) support data arrays
```

## MeSH-Enrichment Analysis (F : Psychiatry and Psychology)

```
negLogPval <- -log10(out.vecLR[['pattern_4_4']]$Enrich$MeSH_F$Pvalue + 1E-10)
term <- out.vecLR[['pattern_4_4']]$Enrich$MeSH_F$Term
target <- seq_len(min(100, length(negLogPval)))
negLogPval <- negLogPval[target]
term <- term[target]
p <- plot_ly(x=seq_along(negLogPval), y=~negLogPval,
type="bar", color=~negLogPval, text=term,
colors=c("#4b61ba", "gray", "#a87963", "red"))
layout(p, title="MeSH-Enrichment Analysis (F)",
   xaxis=list(title="Term"),
   yaxis=list(title="-Log(P-value)"),
   legend=list(name=""))
```

```
## Warning: textfont.color doesn't (yet) support data arrays

## Warning: textfont.color doesn't (yet) support data arrays
```

## MeSH-Enrichment Analysis (G : Phenomena and Processes)

```
negLogPval <- -log10(out.vecLR[['pattern_4_4']]$Enrich$MeSH_G$Pvalue + 1E-10)
term <- out.vecLR[['pattern_4_4']]$Enrich$MeSH_G$Term
target <- seq_len(min(100, length(negLogPval)))
negLogPval <- negLogPval[target]
term <- term[target]
p <- plot_ly(x=seq_along(negLogPval), y=~negLogPval,
type="bar", color=~negLogPval, text=term,
colors=c("#4b61ba", "gray", "#a87963", "red"))
layout(p, title="MeSH-Enrichment Analysis (G)",
   xaxis=list(title="Term"),
   yaxis=list(title="-Log(P-value)"),
   legend=list(name=""))
```

```
## Warning: textfont.color doesn't (yet) support data arrays

## Warning: textfont.color doesn't (yet) support data arrays
```

## MeSH-Enrichment Analysis (H : Disciplines and Occupations)

```
negLogPval <- -log10(out.vecLR[['pattern_4_4']]$Enrich$MeSH_H$Pvalue + 1E-10)
term <- out.vecLR[['pattern_4_4']]$Enrich$MeSH_H$Term
target <- seq_len(min(100, length(negLogPval)))
negLogPval <- negLogPval[target]
term <- term[target]
p <- plot_ly(x=seq_along(negLogPval), y=~negLogPval,
type="bar", color=~negLogPval, text=term,
colors=c("#4b61ba", "gray", "#a87963", "red"))
layout(p, title="MeSH-Enrichment Analysis (H)",
   xaxis=list(title="Term"),
   yaxis=list(title="-Log(P-value)"),
   legend=list(name=""))
```

```
## Warning: textfont.color doesn't (yet) support data arrays

## Warning: textfont.color doesn't (yet) support data arrays
```

## MeSH-Enrichment Analysis (J : Technology and Food and Beverages)

```
negLogPval <- -log10(out.vecLR[['pattern_4_4']]$Enrich$MeSH_J$Pvalue + 1E-10)
term <- out.vecLR[['pattern_4_4']]$Enrich$MeSH_J$Term
target <- seq_len(min(100, length(negLogPval)))
negLogPval <- negLogPval[target]
term <- term[target]
p <- plot_ly(x=seq_along(negLogPval), y=~negLogPval,
type="bar", color=~negLogPval, text=term,
colors=c("#4b61ba", "gray", "#a87963", "red"))
layout(p, title="MeSH-Enrichment Analysis (J)",
   xaxis=list(title="Term"),
   yaxis=list(title="-Log(P-value)"),
   legend=list(name=""))
```

## MeSH-Enrichment Analysis (L : Information Science)

```
negLogPval <- -log10(out.vecLR[['pattern_4_4']]$Enrich$MeSH_L$Pvalue + 1E-10)
term <- out.vecLR[['pattern_4_4']]$Enrich$MeSH_L$Term
target <- seq_len(min(100, length(negLogPval)))
negLogPval <- negLogPval[target]
term <- term[target]
p <- plot_ly(x=seq_along(negLogPval), y=~negLogPval,
type="bar", color=~negLogPval, text=term,
colors=c("#4b61ba", "gray", "#a87963", "red"))
layout(p, title="MeSH-Enrichment Analysis (L)",
   xaxis=list(title="Term"),
   yaxis=list(title="-Log(P-value)"),
   legend=list(name=""))
```

```
## Warning: textfont.color doesn't (yet) support data arrays

## Warning: textfont.color doesn't (yet) support data arrays
```

## MeSH-Enrichment Analysis (N : Health Care)

```
negLogPval <- -log10(out.vecLR[['pattern_4_4']]$Enrich$MeSH_N$Pvalue + 1E-10)
term <- out.vecLR[['pattern_4_4']]$Enrich$MeSH_N$Term
target <- seq_len(min(100, length(negLogPval)))
negLogPval <- negLogPval[target]
term <- term[target]
p <- plot_ly(x=seq_along(negLogPval), y=~negLogPval,
type="bar", color=~negLogPval, text=term,
colors=c("#4b61ba", "gray", "#a87963", "red"))
layout(p, title="MeSH-Enrichment Analysis (N)",
   xaxis=list(title="Term"),
   yaxis=list(title="-Log(P-value)"),
   legend=list(name=""))
```

```
## Warning: textfont.color doesn't (yet) support data arrays

## Warning: textfont.color doesn't (yet) support data arrays
```

## Reactome Pathway-Enrichment Analysis

```
negLogPval <- -log10(out.vecLR[['pattern_4_4']]$Enrich$Reactome$Pvalue + 1E-10)
term <- out.vecLR[['pattern_4_4']]$Enrich$Reactome$Term
target <- seq_len(min(100, length(negLogPval)))
negLogPval <- negLogPval[target]
term <- term[target]
p <- plot_ly(x=seq_along(negLogPval), y=~negLogPval,
type="bar", color=~negLogPval, text=term,
colors=c("#4b61ba", "gray", "#a87963", "red"))
layout(p, title="Reactome-Enrichment Analysis",
   xaxis=list(title="Term"),
   yaxis=list(title="-Log(P-value)"),
   legend=list(name=""))
```

```
## Warning: textfont.color doesn't (yet) support data arrays

## Warning: textfont.color doesn't (yet) support data arrays
```
