# Supplementary material for: Sctensor detects many-to-many cell–cell interactions from single cell RNA-sequencing data
Source: BMC Bioinformatics. 2023 Nov 7;24:420. doi: 10.1186/s12859-023-05490-y (PMC10631077; doi:10.1186/s12859-023-05490-y)
Supplement: Supplementary file 18 — Additional file 18. HTML report of VisualCortex. [file 12859_2023_5490_MOESM18_ESM.zip › output/report/Mouse_VisualCortex/pattern_6_4.html]

Details of (6,4,) -related L-R pairs


# Details of (6,4,) -related L-R pairs

# (6,4,:) Pattern

# (6,4,) -related L-R pairs (Top11 pairs)

```
library("scTensor")
load("reanalysis.RData")
col <- .setColor("many")[24]
scTensor:::.myvisNetwork(g, col)
```

| Rank | Ligand Gene | Receptor Gene | Ligand Expression (Log10(exp + 1)) | Receptor Expression (Log10(exp + 1)) | LR-pair factor value (Percentage) | P-value (Grubbs test) | Q-value (BH method) | L-R Evidence cf. rikenbit/lrbase-workflow | PubMed |
| --- | --- | --- | --- | --- | --- | --- | --- | --- | --- |
| 1 | Nlgn3 Description: Nlgn3neuroligin 3 GO: 1-59 Reactome: 1-3 UniProtKB: 1 RefEx: Nlgn3 Expression Atlas: Nlgn3 Single Cell Expression Atlas: Nlgn3 PanglaoDB: Nlgn3 MeSH: 1-82 83-164 165-246 247-328 329-408 | Nrxn3 Description: Nlgn3neurexin III GO: 1-18 Reactome: 1-3 UniProtKB: 1-2 RefEx: Nlgn3 Expression Atlas: Nrxn3 Single Cell Expression Atlas: Nrxn3 PanglaoDB: Nrxn3 MeSH: 1-78 79-156 157-234 235-311 |  |  | 0.095 (23.253%) | 0 | 0 | DLRP | 1 |
| 2 | Nxph1 Description: Nxph1neurexophilin 1 GO: 1-2 UniProtKB: 1 STRING: 1 2 RefEx: Nxph1 Expression Atlas: Nxph1 Single Cell Expression Atlas: Nxph1 PanglaoDB: Nxph1 MeSH: 1-91 92-181 | Nrxn3 Description: Nxph1neurexin III GO: 1-18 Reactome: 1-3 UniProtKB: 1-2 RefEx: Nxph1 Expression Atlas: Nrxn3 Single Cell Expression Atlas: Nrxn3 PanglaoDB: Nrxn3 MeSH: 1-78 79-156 157-234 235-311 |  |  | 0.056 (13.732%) | 0 | 0 | DLRP | 1 |
| 3 | Apoe Description: Apoeapolipoprotein E GO: 1-83 84-165 Reactome: 1-22 UniProtKB: 1-2 RefEx: Apoe Expression Atlas: Apoe Single Cell Expression Atlas: Apoe PanglaoDB: Apoe MeSH: 1-100 101-200 201-300 301-400 401-500 501-600 601-700 701-800 801-900 901-1000 1001-1100 1101-1200 1201-1300 1301-1400 1401-1500 1501-1600 1601-1700 1701-1800 1801-1900 1901-2000 2001-2100 2101-2200 2201-2300 2301-2400 2401-2500 2501-2600 2601-2700 2701-2800 2801-2900 2901-3000 3001-3100 3101-3200 3201-3300 3301-3400 3401-3500 3501-3600 3601-3700 3701-3800 3801-3900 3901-4000 4001-4100 4101-4200 4201-4300 4301-4400 4401-4500 4501-4600 4601-4677 | Ldlr Description: Apoelow density lipoprotein receptor GO: 1-68 Reactome: 1-9 UniProtKB: 1-2 RefEx: Apoe Expression Atlas: Ldlr Single Cell Expression Atlas: Ldlr PanglaoDB: Ldlr MeSH: 1-100 101-200 201-300 301-400 401-500 501-600 601-700 701-800 801-900 901-1000 1001-1100 1101-1200 1201-1300 1301-1400 1401-1500 1501-1600 1601-1700 1701-1800 1801-1900 1901-2000 2001-2100 2101-2200 2201-2300 2301-2400 2401-2500 2501-2600 2601-2700 2701-2800 2801-2880 |  |  | 0.044 (10.7%) | 0 | 0 | DLRP | 1 |
| 4 | Cck Description: Cckcholecystokinin GO: 1-35 Reactome: 1-7 UniProtKB: 1-2 STRING: 1 2 3 4 5 6 RefEx: Cck Expression Atlas: Cck Single Cell Expression Atlas: Cck PanglaoDB: Cck MeSH: 1-99 100-198 199-297 298-396 397-495 496-594 595-693 694-792 793-891 892-990 991-1089 1090-1188 1189-1287 1288-1386 1387-1485 1486-1584 1585-1683 1684-1782 1783-1881 1882-1980 1981-2079 2080-2178 2179-2277 2278-2376 2377-2475 2476-2574 2575-2673 2674-2772 2773-2871 2872-2970 2971-3069 3070-3168 3169-3267 3268-3340 | Cckbr Description: Cckcholecystokinin B receptor GO: 1-32 Reactome: 1-8 UniProtKB: 1-2 RefEx: Cck Expression Atlas: Cckbr Single Cell Expression Atlas: Cckbr PanglaoDB: Cckbr MeSH: 1-99 100-198 199-297 298-396 397-495 496-589 |  |  | 0.032 (7.805%) | 0 | 0 | DLRP | 1 |
| 5 | Efna2 Description: Efna2ephrin A2 GO: 1-11 Reactome: 1-6 UniProtKB: 1-2 STRING: 1 RefEx: Efna2 Expression Atlas: Efna2 Single Cell Expression Atlas: Efna2 PanglaoDB: Efna2 MeSH: 1-89 90-178 179-267 268-356 357-445 446-531 | Epha4 Description: Efna2Eph receptor A4 GO: 1-90 Reactome: 1-6 UniProtKB: 1 RefEx: Efna2 Expression Atlas: Epha4 Single Cell Expression Atlas: Epha4 PanglaoDB: Epha4 MeSH: 1-95 96-190 191-285 286-380 381-475 476-570 571-665 666-760 761-855 856-950 951-1045 1046-1131 |  |  | 0.021 (5.089%) | 0 | 0 | DLRP | 1 |
| 6 | Bmp7 Description: Bmp7bone morphogenetic protein 7 GO: 1-88 Reactome: 1-3 UniProtKB: 1 STRING: 1 RefEx: Bmp7 Expression Atlas: Bmp7 Single Cell Expression Atlas: Bmp7 PanglaoDB: Bmp7 MeSH: 1-98 99-196 197-294 295-392 393-490 491-588 589-686 687-784 785-882 883-980 981-1078 1079-1176 1177-1274 1275-1360 | Acvr2a Description: Bmp7activin receptor IIA GO: 1-53 Reactome: 1-4 UniProtKB: 1-3 STRING: 1 RefEx: Bmp7 Expression Atlas: Acvr2a Single Cell Expression Atlas: Acvr2a PanglaoDB: Acvr2a MeSH: 1-88 89-176 177-264 265-352 353-440 441-528 529-610 |  |  | 0.018 (4.5%) | 0 | 0 | DLRP | 1 |
| 7 | Mdk Description: Mdkmidkine GO: 1-68 Reactome: 1-3 UniProtKB: 1-2 RefEx: Mdk Expression Atlas: Mdk Single Cell Expression Atlas: Mdk PanglaoDB: Mdk MeSH: 1-91 92-182 183-273 274-364 365-455 456-546 547-637 638-728 729-819 820-906 | Ptprz1 Description: Mdkprotein tyrosine phosphatase, receptor type Z, polypeptide 1 GO: 1-50 Reactome: 1-7 UniProtKB: 1-3 STRING: 1 2 3 RefEx: Mdk Expression Atlas: Ptprz1 Single Cell Expression Atlas: Ptprz1 PanglaoDB: Ptprz1 MeSH: 1-97 98-194 195-291 292-388 389-485 486-582 |  |  | 0.018 (4.479%) | 0 | 0 | DLRP | 1 |
| 8 | Efna2 Description: Efna2ephrin A2 GO: 1-11 Reactome: 1-6 UniProtKB: 1-2 STRING: 1 RefEx: Efna2 Expression Atlas: Efna2 Single Cell Expression Atlas: Efna2 PanglaoDB: Efna2 MeSH: 1-89 90-178 179-267 268-356 357-445 446-531 | Epha5 Description: Efna2Eph receptor A5 GO: 1-45 Reactome: 1-6 UniProtKB: 1-2 RefEx: Efna2 Expression Atlas: Epha5 Single Cell Expression Atlas: Epha5 PanglaoDB: Epha5 MeSH: 1-98 99-196 197-294 295-392 393-489 |  |  | 0.015 (3.648%) | 0 | 0 | DLRP | 1 |
| 9 | Vcam1 Description: Vcam1vascular cell adhesion molecule 1 GO: 1-43 UniProtKB: 1 RefEx: Vcam1 Expression Atlas: Vcam1 Single Cell Expression Atlas: Vcam1 PanglaoDB: Vcam1 MeSH: 1-96 97-192 193-288 289-384 385-480 481-576 577-672 673-768 769-864 865-960 961-1056 1057-1152 1153-1248 1249-1343 | Itga4 Description: Vcam1integrin alpha 4 GO: 1-51 Reactome: 1-7 UniProtKB: 1-3 RefEx: Vcam1 Expression Atlas: Itga4 Single Cell Expression Atlas: Itga4 PanglaoDB: Itga4 MeSH: 1-92 93-184 185-276 277-368 369-460 461-552 553-644 645-736 737-828 829-920 921-1005 |  |  | 0.013 (3.243%) | 0 | 0 | DLRP | 1 |
| 10 | Efnb2 Description: Efnb2ephrin B2 GO: 1-35 Reactome: 1-7 UniProtKB: 1-2 RefEx: Efnb2 Expression Atlas: Efnb2 Single Cell Expression Atlas: Efnb2 PanglaoDB: Efnb2 MeSH: 1-94 95-188 189-282 283-376 377-470 471-564 565-658 659-752 753-846 847-940 941-1034 1035-1125 | Epha4 Description: Efnb2Eph receptor A4 GO: 1-90 Reactome: 1-6 UniProtKB: 1 RefEx: Efnb2 Expression Atlas: Epha4 Single Cell Expression Atlas: Epha4 PanglaoDB: Epha4 MeSH: 1-95 96-190 191-285 286-380 381-475 476-570 571-665 666-760 761-855 856-950 951-1045 1046-1131 |  |  | 0.012 (3.039%) | 0 | 0 | DLRP | 1 |
| 11 | Inhbb Description: Inhbbinhibin beta-B GO: 1-27 Reactome: 1-8 UniProtKB: 1 STRING: 1 RefEx: Inhbb Expression Atlas: Inhbb Single Cell Expression Atlas: Inhbb PanglaoDB: Inhbb MeSH: 1-97 98-194 195-291 292-388 389-485 486-577 | Acvr1b Description: Inhbbactivin A receptor, type 1B GO: 1-50 Reactome: 1-3 UniProtKB: 1-2 RefEx: Inhbb Expression Atlas: Acvr1b Single Cell Expression Atlas: Acvr1b PanglaoDB: Acvr1b MeSH: 1-96 97-192 193-288 289-384 385-480 |  |  | 0.011 (2.715%) | 0 | 0 | DLRP | 1 |

# (6,4,) -related Enrichment Analysis

```
load("reanalysis.RData")
library("plotly")
```

## MeSH-Enrichment Analysis (A : Anatomy)

```
negLogPval <- -log10(out.vecLR[['pattern_6_4']]$Enrich$MeSH_A$Pvalue + 1E-10)
term <- out.vecLR[['pattern_6_4']]$Enrich$MeSH_A$Term
target <- seq_len(min(100, length(negLogPval)))
negLogPval <- negLogPval[target]
term <- term[target]
p <- plot_ly(x=seq_along(negLogPval), y=~negLogPval,
type="bar", color=~negLogPval, text=term,
colors=c("#4b61ba", "gray", "#a87963", "red"))
layout(p, title="MeSH-Enrichment Analysis (A)",
   xaxis=list(title="Term"),
   yaxis=list(title="-Log(P-value)"),
   legend=list(name=""))
```

```
## Warning: textfont.color doesn't (yet) support data arrays

## Warning: textfont.color doesn't (yet) support data arrays
```

## MeSH-Enrichment Analysis (B : Organisms)

```
negLogPval <- -log10(out.vecLR[['pattern_6_4']]$Enrich$MeSH_B$Pvalue + 1E-10)
term <- out.vecLR[['pattern_6_4']]$Enrich$MeSH_B$Term
target <- seq_len(min(100, length(negLogPval)))
negLogPval <- negLogPval[target]
term <- term[target]
p <- plot_ly(x=seq_along(negLogPval), y=~negLogPval,
type="bar", color=~negLogPval, text=term,
colors=c("#4b61ba", "gray", "#a87963", "red"))
layout(p, title="MeSH-Enrichment Analysis (B)",
   xaxis=list(title="Term"),
   yaxis=list(title="-Log(P-value)"),
   legend=list(name=""))
```

```
## Warning: textfont.color doesn't (yet) support data arrays

## Warning: textfont.color doesn't (yet) support data arrays
```

## MeSH-Enrichment Analysis (C : Diseases)

```
negLogPval <- -log10(out.vecLR[['pattern_6_4']]$Enrich$MeSH_C$Pvalue + 1E-10)
term <- out.vecLR[['pattern_6_4']]$Enrich$MeSH_C$Term
target <- seq_len(min(100, length(negLogPval)))
negLogPval <- negLogPval[target]
term <- term[target]
p <- plot_ly(x=seq_along(negLogPval), y=~negLogPval,
type="bar", color=~negLogPval, text=term,
colors=c("#4b61ba", "gray", "#a87963", "red"))
layout(p, title="MeSH-Enrichment Analysis (C)",
   xaxis=list(title="Term"),
   yaxis=list(title="-Log(P-value)"),
   legend=list(name=""))
```

```
## Warning: textfont.color doesn't (yet) support data arrays

## Warning: textfont.color doesn't (yet) support data arrays
```

## MeSH-Enrichment Analysis (D : Drugs)

```
negLogPval <- -log10(out.vecLR[['pattern_6_4']]$Enrich$MeSH_D$Pvalue + 1E-10)
term <- out.vecLR[['pattern_6_4']]$Enrich$MeSH_D$Term
target <- seq_len(min(100, length(negLogPval)))
negLogPval <- negLogPval[target]
term <- term[target]
p <- plot_ly(x=seq_along(negLogPval), y=~negLogPval,
type="bar", color=~negLogPval, text=term,
colors=c("#4b61ba", "gray", "#a87963", "red"))
layout(p, title="MeSH-Enrichment Analysis (D)",
   xaxis=list(title="Term"),
   yaxis=list(title="-Log(P-value)"),
   legend=list(name=""))
```

```
## Warning: textfont.color doesn't (yet) support data arrays

## Warning: textfont.color doesn't (yet) support data arrays
```

## MeSH-Enrichment Analysis (E : Analytical, Diagnostic and Therapeutic Techniques and Equipment)

```
negLogPval <- -log10(out.vecLR[['pattern_6_4']]$Enrich$MeSH_E$Pvalue + 1E-10)
term <- out.vecLR[['pattern_6_4']]$Enrich$MeSH_E$Term
target <- seq_len(min(100, length(negLogPval)))
negLogPval <- negLogPval[target]
term <- term[target]
p <- plot_ly(x=seq_along(negLogPval), y=~negLogPval,
type="bar", color=~negLogPval, text=term,
colors=c("#4b61ba", "gray", "#a87963", "red"))
layout(p, title="MeSH-Enrichment Analysis (E)",
   xaxis=list(title="Term"),
   yaxis=list(title="-Log(P-value)"),
   legend=list(name=""))
```

```
## Warning: textfont.color doesn't (yet) support data arrays

## Warning: textfont.color doesn't (yet) support data arrays
```

## MeSH-Enrichment Analysis (F : Psychiatry and Psychology)

```
negLogPval <- -log10(out.vecLR[['pattern_6_4']]$Enrich$MeSH_F$Pvalue + 1E-10)
term <- out.vecLR[['pattern_6_4']]$Enrich$MeSH_F$Term
target <- seq_len(min(100, length(negLogPval)))
negLogPval <- negLogPval[target]
term <- term[target]
p <- plot_ly(x=seq_along(negLogPval), y=~negLogPval,
type="bar", color=~negLogPval, text=term,
colors=c("#4b61ba", "gray", "#a87963", "red"))
layout(p, title="MeSH-Enrichment Analysis (F)",
   xaxis=list(title="Term"),
   yaxis=list(title="-Log(P-value)"),
   legend=list(name=""))
```

```
## Warning: textfont.color doesn't (yet) support data arrays

## Warning: textfont.color doesn't (yet) support data arrays
```

## MeSH-Enrichment Analysis (G : Phenomena and Processes)

```
negLogPval <- -log10(out.vecLR[['pattern_6_4']]$Enrich$MeSH_G$Pvalue + 1E-10)
term <- out.vecLR[['pattern_6_4']]$Enrich$MeSH_G$Term
target <- seq_len(min(100, length(negLogPval)))
negLogPval <- negLogPval[target]
term <- term[target]
p <- plot_ly(x=seq_along(negLogPval), y=~negLogPval,
type="bar", color=~negLogPval, text=term,
colors=c("#4b61ba", "gray", "#a87963", "red"))
layout(p, title="MeSH-Enrichment Analysis (G)",
   xaxis=list(title="Term"),
   yaxis=list(title="-Log(P-value)"),
   legend=list(name=""))
```

```
## Warning: textfont.color doesn't (yet) support data arrays

## Warning: textfont.color doesn't (yet) support data arrays
```

## MeSH-Enrichment Analysis (H : Disciplines and Occupations)

```
negLogPval <- -log10(out.vecLR[['pattern_6_4']]$Enrich$MeSH_H$Pvalue + 1E-10)
term <- out.vecLR[['pattern_6_4']]$Enrich$MeSH_H$Term
target <- seq_len(min(100, length(negLogPval)))
negLogPval <- negLogPval[target]
term <- term[target]
p <- plot_ly(x=seq_along(negLogPval), y=~negLogPval,
type="bar", color=~negLogPval, text=term,
colors=c("#4b61ba", "gray", "#a87963", "red"))
layout(p, title="MeSH-Enrichment Analysis (H)",
   xaxis=list(title="Term"),
   yaxis=list(title="-Log(P-value)"),
   legend=list(name=""))
```

```
## Warning: textfont.color doesn't (yet) support data arrays

## Warning: textfont.color doesn't (yet) support data arrays
```

## MeSH-Enrichment Analysis (I : Anthropology, Education, Sociology and Social Phenomena)

```
negLogPval <- -log10(out.vecLR[['pattern_6_4']]$Enrich$MeSH_I$Pvalue + 1E-10)
term <- out.vecLR[['pattern_6_4']]$Enrich$MeSH_I$Term
target <- seq_len(min(100, length(negLogPval)))
negLogPval <- negLogPval[target]
term <- term[target]
p <- plot_ly(x=seq_along(negLogPval), y=~negLogPval,
type="bar", color=~negLogPval, text=term,
colors=c("#4b61ba", "gray", "#a87963", "red"))
layout(p, title="MeSH-Enrichment Analysis (I)",
   xaxis=list(title="Term"),
   yaxis=list(title="-Log(P-value)"),
   legend=list(name=""))
```

```
## Warning: textfont.color doesn't (yet) support data arrays

## Warning: textfont.color doesn't (yet) support data arrays
```

## MeSH-Enrichment Analysis (J : Technology and Food and Beverages)

```
negLogPval <- -log10(out.vecLR[['pattern_6_4']]$Enrich$MeSH_J$Pvalue + 1E-10)
term <- out.vecLR[['pattern_6_4']]$Enrich$MeSH_J$Term
target <- seq_len(min(100, length(negLogPval)))
negLogPval <- negLogPval[target]
term <- term[target]
p <- plot_ly(x=seq_along(negLogPval), y=~negLogPval,
type="bar", color=~negLogPval, text=term,
colors=c("#4b61ba", "gray", "#a87963", "red"))
layout(p, title="MeSH-Enrichment Analysis (J)",
   xaxis=list(title="Term"),
   yaxis=list(title="-Log(P-value)"),
   legend=list(name=""))
```

```
## Warning: textfont.color doesn't (yet) support data arrays

## Warning: textfont.color doesn't (yet) support data arrays
```

## MeSH-Enrichment Analysis (L : Information Science)

```
negLogPval <- -log10(out.vecLR[['pattern_6_4']]$Enrich$MeSH_L$Pvalue + 1E-10)
term <- out.vecLR[['pattern_6_4']]$Enrich$MeSH_L$Term
target <- seq_len(min(100, length(negLogPval)))
negLogPval <- negLogPval[target]
term <- term[target]
p <- plot_ly(x=seq_along(negLogPval), y=~negLogPval,
type="bar", color=~negLogPval, text=term,
colors=c("#4b61ba", "gray", "#a87963", "red"))
layout(p, title="MeSH-Enrichment Analysis (L)",
   xaxis=list(title="Term"),
   yaxis=list(title="-Log(P-value)"),
   legend=list(name=""))
```

## MeSH-Enrichment Analysis (N : Health Care)

```
negLogPval <- -log10(out.vecLR[['pattern_6_4']]$Enrich$MeSH_N$Pvalue + 1E-10)
term <- out.vecLR[['pattern_6_4']]$Enrich$MeSH_N$Term
target <- seq_len(min(100, length(negLogPval)))
negLogPval <- negLogPval[target]
term <- term[target]
p <- plot_ly(x=seq_along(negLogPval), y=~negLogPval,
type="bar", color=~negLogPval, text=term,
colors=c("#4b61ba", "gray", "#a87963", "red"))
layout(p, title="MeSH-Enrichment Analysis (N)",
   xaxis=list(title="Term"),
   yaxis=list(title="-Log(P-value)"),
   legend=list(name=""))
```

```
## Warning: textfont.color doesn't (yet) support data arrays

## Warning: textfont.color doesn't (yet) support data arrays
```

## Reactome Pathway-Enrichment Analysis

```
negLogPval <- -log10(out.vecLR[['pattern_6_4']]$Enrich$Reactome$Pvalue + 1E-10)
term <- out.vecLR[['pattern_6_4']]$Enrich$Reactome$Term
target <- seq_len(min(100, length(negLogPval)))
negLogPval <- negLogPval[target]
term <- term[target]
p <- plot_ly(x=seq_along(negLogPval), y=~negLogPval,
type="bar", color=~negLogPval, text=term,
colors=c("#4b61ba", "gray", "#a87963", "red"))
layout(p, title="Reactome-Enrichment Analysis",
   xaxis=list(title="Term"),
   yaxis=list(title="-Log(P-value)"),
   legend=list(name=""))
```

```
## Warning: textfont.color doesn't (yet) support data arrays

## Warning: textfont.color doesn't (yet) support data arrays
```
