# Supplementary material for: Sctensor detects many-to-many cell–cell interactions from single cell RNA-sequencing data
Source: BMC Bioinformatics. 2023 Nov 7;24:420. doi: 10.1186/s12859-023-05490-y (PMC10631077; doi:10.1186/s12859-023-05490-y)
Supplement: Supplementary file 18 — Additional file 18. HTML report of VisualCortex. [file 12859_2023_5490_MOESM18_ESM.zip › output/report/Mouse_VisualCortex/pattern_4_3.html]

Details of (4,3,) -related L-R pairs


# Details of (4,3,) -related L-R pairs

# (4,3,:) Pattern

# (4,3,) -related L-R pairs (Top22 pairs)

```
library("scTensor")
load("reanalysis.RData")
col <- .setColor("many")[29]
scTensor:::.myvisNetwork(g, col)
```

| Rank | Ligand Gene | Receptor Gene | Ligand Expression (Log10(exp + 1)) | Receptor Expression (Log10(exp + 1)) | LR-pair factor value (Percentage) | P-value (Grubbs test) | Q-value (BH method) | L-R Evidence cf. rikenbit/lrbase-workflow | PubMed |
| --- | --- | --- | --- | --- | --- | --- | --- | --- | --- |
| 1 | Sema5a Description: Sema5asema domain, seven thrombospondin repeats (type 1 and type 1-like), transmembrane domain (TM) and short cytoplasmic domain, (semaphorin) 5A GO: 1-38 UniProtKB: 1 RefEx: Sema5a Expression Atlas: Sema5a Single Cell Expression Atlas: Sema5a PanglaoDB: Sema5a MeSH: 1-88 89-176 177-264 265-350 | Plxnb3 Description: Sema5aplexin B3 GO: 1-28 Reactome: 1-5 UniProtKB: 1 RefEx: Sema5a Expression Atlas: Plxnb3 Single Cell Expression Atlas: Plxnb3 PanglaoDB: Plxnb3 MeSH: 1-73 74-146 147-218 |  |  | 0.078 (25.414%) | 0 | 0 | DLRP | 1 |
| 2 | Fgf12 Description: Fgf12fibroblast growth factor 12 GO: 1-18 Reactome: 1-3 UniProtKB: 1-2 RefEx: Fgf12 Expression Atlas: Fgf12 Single Cell Expression Atlas: Fgf12 PanglaoDB: Fgf12 MeSH: 1-86 87-172 173-258 259-341 | Fgfr2 Description: Fgf12fibroblast growth factor receptor 2 GO: 1-66 67-131 Reactome: 1-27 UniProtKB: 1-4 RefEx: Fgf12 Expression Atlas: Fgfr2 Single Cell Expression Atlas: Fgfr2 PanglaoDB: Fgfr2 MeSH: 1-100 101-200 201-300 301-400 401-500 501-600 601-700 701-800 801-900 901-1000 1001-1100 1101-1200 1201-1300 1301-1400 1401-1500 1501-1600 1601-1700 1701-1791 |  |  | 0.027 (8.803%) | 0 | 0 | DLRP | 1 |
| 3 | Fgf13 Description: Fgf13fibroblast growth factor 13 GO: 1-36 Reactome: 1-3 UniProtKB: 1 RefEx: Fgf13 Expression Atlas: Fgf13 Single Cell Expression Atlas: Fgf13 PanglaoDB: Fgf13 MeSH: 1-92 93-184 185-276 277-365 | Fgfr2 Description: Fgf13fibroblast growth factor receptor 2 GO: 1-66 67-131 Reactome: 1-27 UniProtKB: 1-4 RefEx: Fgf13 Expression Atlas: Fgfr2 Single Cell Expression Atlas: Fgfr2 PanglaoDB: Fgfr2 MeSH: 1-100 101-200 201-300 301-400 401-500 501-600 601-700 701-800 801-900 901-1000 1001-1100 1101-1200 1201-1300 1301-1400 1401-1500 1501-1600 1601-1700 1701-1791 |  |  | 0.025 (8.107%) | 0 | 0 | DLRP | 1 |
| 4 | Sema4d Description: Sema4dsema domain, immunoglobulin domain (Ig), transmembrane domain (TM) and short cytoplasmic domain, (semaphorin) 4D GO: 1-39 Reactome: 1-8 UniProtKB: 1 RefEx: Sema4d Expression Atlas: Sema4d Single Cell Expression Atlas: Sema4d PanglaoDB: Sema4d MeSH: 1-89 90-178 179-267 268-356 357-445 446-531 | Plxnb1 Description: Sema4dplexin B1 GO: 1-24 Reactome: 1-15 UniProtKB: 1-2 RefEx: Sema4d Expression Atlas: Plxnb1 Single Cell Expression Atlas: Plxnb1 PanglaoDB: Plxnb1 MeSH: 1-83 84-166 167-249 250-332 333-415 |  |  | 0.019 (6.076%) | 0 | 0 | DLRP | 1 |
| 5 | Fgf9 Description: Fgf9fibroblast growth factor 9 GO: 1-48 Reactome: 1-53 UniProtKB: 1 RefEx: Fgf9 Expression Atlas: Fgf9 Single Cell Expression Atlas: Fgf9 PanglaoDB: Fgf9 MeSH: 1-96 97-192 193-288 289-384 385-480 481-576 577-672 673-768 769-864 865-958 | Fgfr2 Description: Fgf9fibroblast growth factor receptor 2 GO: 1-66 67-131 Reactome: 1-27 UniProtKB: 1-4 RefEx: Fgf9 Expression Atlas: Fgfr2 Single Cell Expression Atlas: Fgfr2 PanglaoDB: Fgfr2 MeSH: 1-100 101-200 201-300 301-400 401-500 501-600 601-700 701-800 801-900 901-1000 1001-1100 1101-1200 1201-1300 1301-1400 1401-1500 1501-1600 1601-1700 1701-1791 |  |  | 0.018 (5.997%) | 0 | 0 | DLRP | 1 |
| 6 | Efnb3 Description: Efnb3ephrin B3 GO: 1-18 Reactome: 1-7 UniProtKB: 1-2 STRING: 1 RefEx: Efnb3 Expression Atlas: Efnb3 Single Cell Expression Atlas: Efnb3 PanglaoDB: Efnb3 MeSH: 1-89 90-178 179-267 268-356 357-445 446-534 535-622 | Ephb1 Description: Efnb3Eph receptor B1 GO: 1-62 Reactome: 1-7 UniProtKB: 1-2 RefEx: Efnb3 Expression Atlas: Ephb1 Single Cell Expression Atlas: Ephb1 PanglaoDB: Ephb1 MeSH: 1-100 101-200 201-300 301-400 401-500 501-600 601-695 |  |  | 0.016 (5.34%) | 0 | 0 | DLRP | 1 |
| 7 | Fgf11 Description: Fgf11fibroblast growth factor 11 GO: 1-6 Reactome: 1-3 UniProtKB: 1-2 RefEx: Fgf11 Expression Atlas: Fgf11 Single Cell Expression Atlas: Fgf11 PanglaoDB: Fgf11 MeSH: 1-68 69-136 137-203 | Fgfr2 Description: Fgf11fibroblast growth factor receptor 2 GO: 1-66 67-131 Reactome: 1-27 UniProtKB: 1-4 RefEx: Fgf11 Expression Atlas: Fgfr2 Single Cell Expression Atlas: Fgfr2 PanglaoDB: Fgfr2 MeSH: 1-100 101-200 201-300 301-400 401-500 501-600 601-700 701-800 801-900 901-1000 1001-1100 1101-1200 1201-1300 1301-1400 1401-1500 1501-1600 1601-1700 1701-1791 |  |  | 0.011 (3.527%) | 0 | 0 | DLRP | 1 |
| 8 | Fgf12 Description: Fgf12fibroblast growth factor 12 GO: 1-18 Reactome: 1-3 UniProtKB: 1-2 RefEx: Fgf12 Expression Atlas: Fgf12 Single Cell Expression Atlas: Fgf12 PanglaoDB: Fgf12 MeSH: 1-86 87-172 173-258 259-341 | Fgfr3 Description: Fgf12fibroblast growth factor receptor 3 GO: 1-91 UniProtKB: 1-4 RefEx: Fgf12 Expression Atlas: Fgfr3 Single Cell Expression Atlas: Fgfr3 PanglaoDB: Fgfr3 MeSH: 1-97 98-194 195-291 292-388 389-485 486-582 583-679 680-776 777-873 874-970 971-1067 1068-1164 1165-1261 1262-1358 1359-1454 |  |  | 0.01 (3.394%) | 0 | 0 | DLRP | 1 |
| 9 | Fgf13 Description: Fgf13fibroblast growth factor 13 GO: 1-36 Reactome: 1-3 UniProtKB: 1 RefEx: Fgf13 Expression Atlas: Fgf13 Single Cell Expression Atlas: Fgf13 PanglaoDB: Fgf13 MeSH: 1-92 93-184 185-276 277-365 | Fgfr3 Description: Fgf13fibroblast growth factor receptor 3 GO: 1-91 UniProtKB: 1-4 RefEx: Fgf13 Expression Atlas: Fgfr3 Single Cell Expression Atlas: Fgfr3 PanglaoDB: Fgfr3 MeSH: 1-97 98-194 195-291 292-388 389-485 486-582 583-679 680-776 777-873 874-970 971-1067 1068-1164 1165-1261 1262-1358 1359-1454 |  |  | 0.01 (3.125%) | 0 | 0 | DLRP | 1 |
| 10 | Efnb2 Description: Efnb2ephrin B2 GO: 1-35 Reactome: 1-7 UniProtKB: 1-2 RefEx: Efnb2 Expression Atlas: Efnb2 Single Cell Expression Atlas: Efnb2 PanglaoDB: Efnb2 MeSH: 1-94 95-188 189-282 283-376 377-470 471-564 565-658 659-752 753-846 847-940 941-1034 1035-1125 | Ephb1 Description: Efnb2Eph receptor B1 GO: 1-62 Reactome: 1-7 UniProtKB: 1-2 RefEx: Efnb2 Expression Atlas: Ephb1 Single Cell Expression Atlas: Ephb1 PanglaoDB: Ephb1 MeSH: 1-100 101-200 201-300 301-400 401-500 501-600 601-695 |  |  | 0.008 (2.561%) | 0 | 0 | DLRP | 1 |
| 11 | Efna3 Description: Efna3ephrin A3 GO: 1-8 Reactome: 1-6 UniProtKB: 1 RefEx: Efna3 Expression Atlas: Efna3 Single Cell Expression Atlas: Efna3 PanglaoDB: Efna3 MeSH: 1-84 85-168 169-252 253-336 337-419 | Ephb1 Description: Efna3Eph receptor B1 GO: 1-62 Reactome: 1-7 UniProtKB: 1-2 RefEx: Efna3 Expression Atlas: Ephb1 Single Cell Expression Atlas: Ephb1 PanglaoDB: Ephb1 MeSH: 1-100 101-200 201-300 301-400 401-500 501-600 601-695 |  |  | 0.008 (2.445%) | 0 | 0 | DLRP | 1 |
| 12 | Fgf9 Description: Fgf9fibroblast growth factor 9 GO: 1-48 Reactome: 1-53 UniProtKB: 1 RefEx: Fgf9 Expression Atlas: Fgf9 Single Cell Expression Atlas: Fgf9 PanglaoDB: Fgf9 MeSH: 1-96 97-192 193-288 289-384 385-480 481-576 577-672 673-768 769-864 865-958 | Fgfr3 Description: Fgf9fibroblast growth factor receptor 3 GO: 1-91 UniProtKB: 1-4 RefEx: Fgf9 Expression Atlas: Fgfr3 Single Cell Expression Atlas: Fgfr3 PanglaoDB: Fgfr3 MeSH: 1-97 98-194 195-291 292-388 389-485 486-582 583-679 680-776 777-873 874-970 971-1067 1068-1164 1165-1261 1262-1358 1359-1454 |  |  | 0.007 (2.312%) | 0 | 0 | DLRP | 1 |
| 13 | Edn3 Description: Edn3endothelin 3 GO: 1-34 Reactome: 1-7 UniProtKB: 1-2 RefEx: Edn3 Expression Atlas: Edn3 Single Cell Expression Atlas: Edn3 PanglaoDB: Edn3 MeSH: 1-95 96-190 191-285 286-380 381-474 | Ednrb Description: Edn3endothelin receptor type B GO: 1-66 Reactome: 1-7 UniProtKB: 1 RefEx: Edn3 Expression Atlas: Ednrb Single Cell Expression Atlas: Ednrb PanglaoDB: Ednrb MeSH: 1-100 101-200 201-300 301-400 401-500 501-600 601-700 701-800 801-900 901-996 |  |  | 0.006 (2.052%) | 0 | 0 | DLRP | 1 |
| 14 | Fgf7 Description: Fgf7fibroblast growth factor 7 GO: 1-38 Reactome: 1-26 UniProtKB: 1-2 RefEx: Fgf7 Expression Atlas: Fgf7 Single Cell Expression Atlas: Fgf7 PanglaoDB: Fgf7 MeSH: 1-93 94-186 187-279 280-372 373-465 466-558 559-651 652-744 745-837 838-930 931-1023 1024-1116 1117-1202 | Fgfr2 Description: Fgf7fibroblast growth factor receptor 2 GO: 1-66 67-131 Reactome: 1-27 UniProtKB: 1-4 RefEx: Fgf7 Expression Atlas: Fgfr2 Single Cell Expression Atlas: Fgfr2 PanglaoDB: Fgfr2 MeSH: 1-100 101-200 201-300 301-400 401-500 501-600 601-700 701-800 801-900 901-1000 1001-1100 1101-1200 1201-1300 1301-1400 1401-1500 1501-1600 1601-1700 1701-1791 |  |  | 0.006 (2.015%) | 0 | 0 | DLRP | 1 |
| 15 | Col18a1 Description: Col18a1collagen, type XVIII, alpha 1 GO: 1-18 Reactome: 1-10 UniProtKB: 1-2 RefEx: Col18a1 Expression Atlas: Col18a1 Single Cell Expression Atlas: Col18a1 PanglaoDB: Col18a1 MeSH: 1-93 94-186 187-279 280-372 373-465 466-558 559-651 652-744 | Gpc1 Description: Col18a1glypican 1 GO: 1-27 Reactome: 1-19 UniProtKB: 1-2 STRING: 1 RefEx: Col18a1 Expression Atlas: Gpc1 Single Cell Expression Atlas: Gpc1 PanglaoDB: Gpc1 MeSH: 1-91 92-182 183-273 274-364 |  |  | 0.006 (1.925%) | 0 | 0 | DLRP | 1 |
| 16 | Fgf1 Description: Fgf1fibroblast growth factor 1 GO: 1-48 Reactome: 1-55 UniProtKB: 1-2 RefEx: Fgf1 Expression Atlas: Fgf1 Single Cell Expression Atlas: Fgf1 PanglaoDB: Fgf1 MeSH: 1-98 99-196 197-294 295-392 393-490 491-588 589-686 687-784 785-882 883-979 | Fgfr2 Description: Fgf1fibroblast growth factor receptor 2 GO: 1-66 67-131 Reactome: 1-27 UniProtKB: 1-4 RefEx: Fgf1 Expression Atlas: Fgfr2 Single Cell Expression Atlas: Fgfr2 PanglaoDB: Fgfr2 MeSH: 1-100 101-200 201-300 301-400 401-500 501-600 601-700 701-800 801-900 901-1000 1001-1100 1101-1200 1201-1300 1301-1400 1401-1500 1501-1600 1601-1700 1701-1791 |  |  | 0.005 (1.476%) | 0 | 0 | DLRP | 1 |
| 17 | Fgf11 Description: Fgf11fibroblast growth factor 11 GO: 1-6 Reactome: 1-3 UniProtKB: 1-2 RefEx: Fgf11 Expression Atlas: Fgf11 Single Cell Expression Atlas: Fgf11 PanglaoDB: Fgf11 MeSH: 1-68 69-136 137-203 | Fgfr3 Description: Fgf11fibroblast growth factor receptor 3 GO: 1-91 UniProtKB: 1-4 RefEx: Fgf11 Expression Atlas: Fgfr3 Single Cell Expression Atlas: Fgfr3 PanglaoDB: Fgfr3 MeSH: 1-97 98-194 195-291 292-388 389-485 486-582 583-679 680-776 777-873 874-970 971-1067 1068-1164 1165-1261 1262-1358 1359-1454 |  |  | 0.004 (1.36%) | 0 | 0 | DLRP | 1 |
| 18 | Hbegf Description: Hbegfheparin-binding EGF-like growth factor GO: 1-27 Reactome: 1-40 UniProtKB: 1-2 STRING: 1 RefEx: Hbegf Expression Atlas: Hbegf Single Cell Expression Atlas: Hbegf PanglaoDB: Hbegf MeSH: 1-93 94-186 187-279 280-372 373-465 466-558 559-651 652-744 745-834 | Erbb4 Description: Hbegferb-b2 receptor tyrosine kinase 4 GO: 1-80 Reactome: 1-26 UniProtKB: 1 RefEx: Hbegf Expression Atlas: Erbb4 Single Cell Expression Atlas: Erbb4 PanglaoDB: Erbb4 MeSH: 1-97 98-194 195-291 292-388 389-485 486-582 583-679 680-776 777-873 874-970 971-1061 |  |  | 0.004 (1.216%) | 0 | 0 | DLRP | 1 |
| 19 | Slit2 Description: Slit2slit guidance ligand 2 GO: 1-72 Reactome: 1-7 UniProtKB: 1-4 RefEx: Slit2 Expression Atlas: Slit2 Single Cell Expression Atlas: Slit2 PanglaoDB: Slit2 MeSH: 1-94 95-188 189-282 283-376 377-470 471-564 565-658 659-752 753-844 | Gpc1 Description: Slit2glypican 1 GO: 1-27 Reactome: 1-19 UniProtKB: 1-2 STRING: 1 RefEx: Slit2 Expression Atlas: Gpc1 Single Cell Expression Atlas: Gpc1 PanglaoDB: Gpc1 MeSH: 1-91 92-182 183-273 274-364 |  |  | 0.004 (1.204%) | 0 | 0 | DLRP | 1 |
| 20 | Wnt5a Description: Wnt5awingless-type MMTV integration site family, member 5A GO: 1-92 93-183 Reactome: 1-14 UniProtKB: 1 RefEx: Wnt5a Expression Atlas: Wnt5a Single Cell Expression Atlas: Wnt5a PanglaoDB: Wnt5a MeSH: 1-96 97-192 193-288 289-384 385-480 481-576 577-672 673-768 769-864 865-960 961-1056 1057-1152 1153-1248 1249-1344 1345-1440 1441-1531 | Fzd2 Description: Wnt5afrizzled class receptor 2 GO: 1-35 Reactome: 1-9 UniProtKB: 1 STRING: 1 RefEx: Wnt5a Expression Atlas: Fzd2 Single Cell Expression Atlas: Fzd2 PanglaoDB: Fzd2 MeSH: 1-99 100-198 199-297 298-396 397-491 |  |  | 0.003 (1.022%) | 0 | 0 | DLRP | 1 |
| 21 | Fgf14 Description: Fgf14fibroblast growth factor 14 GO: 1-18 Reactome: 1-3 UniProtKB: 1-3 RefEx: Fgf14 Expression Atlas: Fgf14 Single Cell Expression Atlas: Fgf14 PanglaoDB: Fgf14 MeSH: 1-89 90-178 179-267 268-356 357-445 446-534 535-620 | Fgfr2 Description: Fgf14fibroblast growth factor receptor 2 GO: 1-66 67-131 Reactome: 1-27 UniProtKB: 1-4 RefEx: Fgf14 Expression Atlas: Fgfr2 Single Cell Expression Atlas: Fgfr2 PanglaoDB: Fgfr2 MeSH: 1-100 101-200 201-300 301-400 401-500 501-600 601-700 701-800 801-900 901-1000 1001-1100 1101-1200 1201-1300 1301-1400 1401-1500 1501-1600 1601-1700 1701-1791 |  |  | 0.003 (0.907%) | 0 | 0 | DLRP | 1 |
| 22 | Col1a1 Description: Col1a1collagen, type I, alpha 1 GO: 1-48 Reactome: 1-27 UniProtKB: 1 RefEx: Col1a1 Expression Atlas: Col1a1 Single Cell Expression Atlas: Col1a1 PanglaoDB: Col1a1 MeSH: 1-99 100-198 199-297 298-396 397-495 496-594 595-693 694-792 793-891 892-990 991-1089 1090-1188 1189-1287 1288-1386 1387-1485 1486-1584 1585-1683 1684-1782 1783-1881 1882-1980 1981-2079 2080-2178 2179-2276 | Ddr1 Description: Col1a1discoidin domain receptor family, member 1 GO: 1-42 Reactome: 1-2 UniProtKB: 1-3 RefEx: Col1a1 Expression Atlas: Ddr1 Single Cell Expression Atlas: Ddr1 PanglaoDB: Ddr1 MeSH: 1-89 90-178 179-267 268-356 357-445 446-530 |  |  | 0.003 (0.86%) | 0 | 0 | DLRP | 1 |

# (4,3,) -related Enrichment Analysis

```
load("reanalysis.RData")
library("plotly")
```

## MeSH-Enrichment Analysis (A : Anatomy)

```
negLogPval <- -log10(out.vecLR[['pattern_4_3']]$Enrich$MeSH_A$Pvalue + 1E-10)
term <- out.vecLR[['pattern_4_3']]$Enrich$MeSH_A$Term
target <- seq_len(min(100, length(negLogPval)))
negLogPval <- negLogPval[target]
term <- term[target]
p <- plot_ly(x=seq_along(negLogPval), y=~negLogPval,
type="bar", color=~negLogPval, text=term,
colors=c("#4b61ba", "gray", "#a87963", "red"))
layout(p, title="MeSH-Enrichment Analysis (A)",
   xaxis=list(title="Term"),
   yaxis=list(title="-Log(P-value)"),
   legend=list(name=""))
```

```
## Warning: textfont.color doesn't (yet) support data arrays

## Warning: textfont.color doesn't (yet) support data arrays
```

## MeSH-Enrichment Analysis (B : Organisms)

```
negLogPval <- -log10(out.vecLR[['pattern_4_3']]$Enrich$MeSH_B$Pvalue + 1E-10)
term <- out.vecLR[['pattern_4_3']]$Enrich$MeSH_B$Term
target <- seq_len(min(100, length(negLogPval)))
negLogPval <- negLogPval[target]
term <- term[target]
p <- plot_ly(x=seq_along(negLogPval), y=~negLogPval,
type="bar", color=~negLogPval, text=term,
colors=c("#4b61ba", "gray", "#a87963", "red"))
layout(p, title="MeSH-Enrichment Analysis (B)",
   xaxis=list(title="Term"),
   yaxis=list(title="-Log(P-value)"),
   legend=list(name=""))
```

```
## Warning: textfont.color doesn't (yet) support data arrays

## Warning: textfont.color doesn't (yet) support data arrays
```

## MeSH-Enrichment Analysis (C : Diseases)

```
negLogPval <- -log10(out.vecLR[['pattern_4_3']]$Enrich$MeSH_C$Pvalue + 1E-10)
term <- out.vecLR[['pattern_4_3']]$Enrich$MeSH_C$Term
target <- seq_len(min(100, length(negLogPval)))
negLogPval <- negLogPval[target]
term <- term[target]
p <- plot_ly(x=seq_along(negLogPval), y=~negLogPval,
type="bar", color=~negLogPval, text=term,
colors=c("#4b61ba", "gray", "#a87963", "red"))
layout(p, title="MeSH-Enrichment Analysis (C)",
   xaxis=list(title="Term"),
   yaxis=list(title="-Log(P-value)"),
   legend=list(name=""))
```

```
## Warning: textfont.color doesn't (yet) support data arrays

## Warning: textfont.color doesn't (yet) support data arrays
```

## MeSH-Enrichment Analysis (D : Drugs)

```
negLogPval <- -log10(out.vecLR[['pattern_4_3']]$Enrich$MeSH_D$Pvalue + 1E-10)
term <- out.vecLR[['pattern_4_3']]$Enrich$MeSH_D$Term
target <- seq_len(min(100, length(negLogPval)))
negLogPval <- negLogPval[target]
term <- term[target]
p <- plot_ly(x=seq_along(negLogPval), y=~negLogPval,
type="bar", color=~negLogPval, text=term,
colors=c("#4b61ba", "gray", "#a87963", "red"))
layout(p, title="MeSH-Enrichment Analysis (D)",
   xaxis=list(title="Term"),
   yaxis=list(title="-Log(P-value)"),
   legend=list(name=""))
```

```
## Warning: textfont.color doesn't (yet) support data arrays

## Warning: textfont.color doesn't (yet) support data arrays
```

## MeSH-Enrichment Analysis (E : Analytical, Diagnostic and Therapeutic Techniques and Equipment)

```
negLogPval <- -log10(out.vecLR[['pattern_4_3']]$Enrich$MeSH_E$Pvalue + 1E-10)
term <- out.vecLR[['pattern_4_3']]$Enrich$MeSH_E$Term
target <- seq_len(min(100, length(negLogPval)))
negLogPval <- negLogPval[target]
term <- term[target]
p <- plot_ly(x=seq_along(negLogPval), y=~negLogPval,
type="bar", color=~negLogPval, text=term,
colors=c("#4b61ba", "gray", "#a87963", "red"))
layout(p, title="MeSH-Enrichment Analysis (E)",
   xaxis=list(title="Term"),
   yaxis=list(title="-Log(P-value)"),
   legend=list(name=""))
```

```
## Warning: textfont.color doesn't (yet) support data arrays

## Warning: textfont.color doesn't (yet) support data arrays
```

## MeSH-Enrichment Analysis (F : Psychiatry and Psychology)

```
negLogPval <- -log10(out.vecLR[['pattern_4_3']]$Enrich$MeSH_F$Pvalue + 1E-10)
term <- out.vecLR[['pattern_4_3']]$Enrich$MeSH_F$Term
target <- seq_len(min(100, length(negLogPval)))
negLogPval <- negLogPval[target]
term <- term[target]
p <- plot_ly(x=seq_along(negLogPval), y=~negLogPval,
type="bar", color=~negLogPval, text=term,
colors=c("#4b61ba", "gray", "#a87963", "red"))
layout(p, title="MeSH-Enrichment Analysis (F)",
   xaxis=list(title="Term"),
   yaxis=list(title="-Log(P-value)"),
   legend=list(name=""))
```

```
## Warning: textfont.color doesn't (yet) support data arrays

## Warning: textfont.color doesn't (yet) support data arrays
```

## MeSH-Enrichment Analysis (G : Phenomena and Processes)

```
negLogPval <- -log10(out.vecLR[['pattern_4_3']]$Enrich$MeSH_G$Pvalue + 1E-10)
term <- out.vecLR[['pattern_4_3']]$Enrich$MeSH_G$Term
target <- seq_len(min(100, length(negLogPval)))
negLogPval <- negLogPval[target]
term <- term[target]
p <- plot_ly(x=seq_along(negLogPval), y=~negLogPval,
type="bar", color=~negLogPval, text=term,
colors=c("#4b61ba", "gray", "#a87963", "red"))
layout(p, title="MeSH-Enrichment Analysis (G)",
   xaxis=list(title="Term"),
   yaxis=list(title="-Log(P-value)"),
   legend=list(name=""))
```

```
## Warning: textfont.color doesn't (yet) support data arrays

## Warning: textfont.color doesn't (yet) support data arrays
```

## MeSH-Enrichment Analysis (L : Information Science)

```
negLogPval <- -log10(out.vecLR[['pattern_4_3']]$Enrich$MeSH_L$Pvalue + 1E-10)
term <- out.vecLR[['pattern_4_3']]$Enrich$MeSH_L$Term
target <- seq_len(min(100, length(negLogPval)))
negLogPval <- negLogPval[target]
term <- term[target]
p <- plot_ly(x=seq_along(negLogPval), y=~negLogPval,
type="bar", color=~negLogPval, text=term,
colors=c("#4b61ba", "gray", "#a87963", "red"))
layout(p, title="MeSH-Enrichment Analysis (L)",
   xaxis=list(title="Term"),
   yaxis=list(title="-Log(P-value)"),
   legend=list(name=""))
```

```
## Warning: textfont.color doesn't (yet) support data arrays

## Warning: textfont.color doesn't (yet) support data arrays
```

## MeSH-Enrichment Analysis (M : Persons)

```
negLogPval <- -log10(out.vecLR[['pattern_4_3']]$Enrich$MeSH_M$Pvalue + 1E-10)
term <- out.vecLR[['pattern_4_3']]$Enrich$MeSH_M$Term
target <- seq_len(min(100, length(negLogPval)))
negLogPval <- negLogPval[target]
term <- term[target]
p <- plot_ly(x=seq_along(negLogPval), y=~negLogPval,
type="bar", color=~negLogPval, text=term,
colors=c("#4b61ba", "gray", "#a87963", "red"))
layout(p, title="MeSH-Enrichment Analysis (M)",
   xaxis=list(title="Term"),
   yaxis=list(title="-Log(P-value)"),
   legend=list(name=""))
```

## Reactome Pathway-Enrichment Analysis

```
negLogPval <- -log10(out.vecLR[['pattern_4_3']]$Enrich$Reactome$Pvalue + 1E-10)
term <- out.vecLR[['pattern_4_3']]$Enrich$Reactome$Term
target <- seq_len(min(100, length(negLogPval)))
negLogPval <- negLogPval[target]
term <- term[target]
p <- plot_ly(x=seq_along(negLogPval), y=~negLogPval,
type="bar", color=~negLogPval, text=term,
colors=c("#4b61ba", "gray", "#a87963", "red"))
layout(p, title="Reactome-Enrichment Analysis",
   xaxis=list(title="Term"),
   yaxis=list(title="-Log(P-value)"),
   legend=list(name=""))
```

```
## Warning: textfont.color doesn't (yet) support data arrays

## Warning: textfont.color doesn't (yet) support data arrays
```
