# Supplementary material for: Sctensor detects many-to-many cell–cell interactions from single cell RNA-sequencing data
Source: BMC Bioinformatics. 2023 Nov 7;24:420. doi: 10.1186/s12859-023-05490-y (PMC10631077; doi:10.1186/s12859-023-05490-y)
Supplement: Supplementary file 18 — Additional file 18. HTML report of VisualCortex. [file 12859_2023_5490_MOESM18_ESM.zip › output/report/Mouse_VisualCortex/pattern_2_4.html]

Details of (2,4,) -related L-R pairs


# Details of (2,4,) -related L-R pairs

# (2,4,:) Pattern

# (2,4,) -related L-R pairs (Top12 pairs)

```
library("scTensor")
load("reanalysis.RData")
col <- .setColor("many")[33]
scTensor:::.myvisNetwork(g, col)
```

| Rank | Ligand Gene | Receptor Gene | Ligand Expression (Log10(exp + 1)) | Receptor Expression (Log10(exp + 1)) | LR-pair factor value (Percentage) | P-value (Grubbs test) | Q-value (BH method) | L-R Evidence cf. rikenbit/lrbase-workflow | PubMed |
| --- | --- | --- | --- | --- | --- | --- | --- | --- | --- |
| 1 | Cck Description: Cckcholecystokinin GO: 1-35 Reactome: 1-7 UniProtKB: 1-2 STRING: 1 2 3 4 5 6 RefEx: Cck Expression Atlas: Cck Single Cell Expression Atlas: Cck PanglaoDB: Cck MeSH: 1-99 100-198 199-297 298-396 397-495 496-594 595-693 694-792 793-891 892-990 991-1089 1090-1188 1189-1287 1288-1386 1387-1485 1486-1584 1585-1683 1684-1782 1783-1881 1882-1980 1981-2079 2080-2178 2179-2277 2278-2376 2377-2475 2476-2574 2575-2673 2674-2772 2773-2871 2872-2970 2971-3069 3070-3168 3169-3267 3268-3340 | Cckbr Description: Cckcholecystokinin B receptor GO: 1-32 Reactome: 1-8 UniProtKB: 1-2 RefEx: Cck Expression Atlas: Cckbr Single Cell Expression Atlas: Cckbr PanglaoDB: Cckbr MeSH: 1-99 100-198 199-297 298-396 397-495 496-589 |  |  | 0.053 (18.336%) | 0 | 0 | DLRP | 1 |
| 2 | Nodal Description: Nodalnodal GO: 1-78 UniProtKB: 1 STRING: 1 RefEx: Nodal Expression Atlas: Nodal Single Cell Expression Atlas: Nodal PanglaoDB: Nodal MeSH: 1-97 98-194 195-291 292-388 389-485 486-582 583-679 680-776 777-873 874-963 | Acvr1b Description: Nodalactivin A receptor, type 1B GO: 1-50 Reactome: 1-3 UniProtKB: 1-2 RefEx: Nodal Expression Atlas: Acvr1b Single Cell Expression Atlas: Acvr1b PanglaoDB: Acvr1b MeSH: 1-96 97-192 193-288 289-384 385-480 |  |  | 0.032 (10.988%) | 0 | 0 | DLRP | 1 |
| 3 | Efna3 Description: Efna3ephrin A3 GO: 1-8 Reactome: 1-6 UniProtKB: 1 RefEx: Efna3 Expression Atlas: Efna3 Single Cell Expression Atlas: Efna3 PanglaoDB: Efna3 MeSH: 1-84 85-168 169-252 253-336 337-419 | Epha4 Description: Efna3Eph receptor A4 GO: 1-90 Reactome: 1-6 UniProtKB: 1 RefEx: Efna3 Expression Atlas: Epha4 Single Cell Expression Atlas: Epha4 PanglaoDB: Epha4 MeSH: 1-95 96-190 191-285 286-380 381-475 476-570 571-665 666-760 761-855 856-950 951-1045 1046-1131 |  |  | 0.02 (6.904%) | 0 | 0 | DLRP | 1 |
| 4 | Efna5 Description: Efna5ephrin A5 GO: 1-34 Reactome: 1-6 UniProtKB: 1 RefEx: Efna5 Expression Atlas: Efna5 Single Cell Expression Atlas: Efna5 PanglaoDB: Efna5 MeSH: 1-90 91-180 181-270 271-360 361-450 451-540 541-630 631-718 | Epha4 Description: Efna5Eph receptor A4 GO: 1-90 Reactome: 1-6 UniProtKB: 1 RefEx: Efna5 Expression Atlas: Epha4 Single Cell Expression Atlas: Epha4 PanglaoDB: Epha4 MeSH: 1-95 96-190 191-285 286-380 381-475 476-570 571-665 666-760 761-855 856-950 951-1045 1046-1131 |  |  | 0.02 (6.807%) | 0 | 0 | DLRP | 1 |
| 5 | Efnb2 Description: Efnb2ephrin B2 GO: 1-35 Reactome: 1-7 UniProtKB: 1-2 RefEx: Efnb2 Expression Atlas: Efnb2 Single Cell Expression Atlas: Efnb2 PanglaoDB: Efnb2 MeSH: 1-94 95-188 189-282 283-376 377-470 471-564 565-658 659-752 753-846 847-940 941-1034 1035-1125 | Epha4 Description: Efnb2Eph receptor A4 GO: 1-90 Reactome: 1-6 UniProtKB: 1 RefEx: Efnb2 Expression Atlas: Epha4 Single Cell Expression Atlas: Epha4 PanglaoDB: Epha4 MeSH: 1-95 96-190 191-285 286-380 381-475 476-570 571-665 666-760 761-855 856-950 951-1045 1046-1131 |  |  | 0.016 (5.454%) | 0 | 0 | DLRP | 1 |
| 6 | Efna3 Description: Efna3ephrin A3 GO: 1-8 Reactome: 1-6 UniProtKB: 1 RefEx: Efna3 Expression Atlas: Efna3 Single Cell Expression Atlas: Efna3 PanglaoDB: Efna3 MeSH: 1-84 85-168 169-252 253-336 337-419 | Epha5 Description: Efna3Eph receptor A5 GO: 1-45 Reactome: 1-6 UniProtKB: 1-2 RefEx: Efna3 Expression Atlas: Epha5 Single Cell Expression Atlas: Epha5 PanglaoDB: Epha5 MeSH: 1-98 99-196 197-294 295-392 393-489 |  |  | 0.014 (4.95%) | 0 | 0 | DLRP | 1 |
| 7 | Efna5 Description: Efna5ephrin A5 GO: 1-34 Reactome: 1-6 UniProtKB: 1 RefEx: Efna5 Expression Atlas: Efna5 Single Cell Expression Atlas: Efna5 PanglaoDB: Efna5 MeSH: 1-90 91-180 181-270 271-360 361-450 451-540 541-630 631-718 | Epha5 Description: Efna5Eph receptor A5 GO: 1-45 Reactome: 1-6 UniProtKB: 1-2 RefEx: Efna5 Expression Atlas: Epha5 Single Cell Expression Atlas: Epha5 PanglaoDB: Epha5 MeSH: 1-98 99-196 197-294 295-392 393-489 |  |  | 0.014 (4.879%) | 0 | 0 | DLRP | 1 |
| 8 | Nodal Description: Nodalnodal GO: 1-78 UniProtKB: 1 STRING: 1 RefEx: Nodal Expression Atlas: Nodal Single Cell Expression Atlas: Nodal PanglaoDB: Nodal MeSH: 1-97 98-194 195-291 292-388 389-485 486-582 583-679 680-776 777-873 874-963 | Acvr2a Description: Nodalactivin receptor IIA GO: 1-53 Reactome: 1-4 UniProtKB: 1-3 STRING: 1 RefEx: Nodal Expression Atlas: Acvr2a Single Cell Expression Atlas: Acvr2a PanglaoDB: Acvr2a MeSH: 1-88 89-176 177-264 265-352 353-440 441-528 529-610 |  |  | 0.013 (4.555%) | 0 | 0 | DLRP | 1 |
| 9 | Il34 Description: Il34interleukin 34 GO: 1-21 Reactome: 1-4 UniProtKB: 1 RefEx: Il34 Expression Atlas: Il34 Single Cell Expression Atlas: Il34 PanglaoDB: Il34 MeSH: 1-94 95-188 189-281 | Csf1r Description: Il34colony stimulating factor 1 receptor GO: 1-70 Reactome: 1-4 UniProtKB: 1-4 RefEx: Il34 Expression Atlas: Csf1r Single Cell Expression Atlas: Csf1r PanglaoDB: Csf1r MeSH: 1-99 100-198 199-297 298-396 397-495 496-594 595-693 694-792 793-891 892-990 991-1080 |  |  | 0.011 (3.853%) | 0 | 0 | DLRP | 1 |
| 10 | Vtn Description: Vtnvitronectin GO: 1-27 Reactome: 1-11 UniProtKB: 1 STRING: 1 RefEx: Vtn Expression Atlas: Vtn Single Cell Expression Atlas: Vtn PanglaoDB: Vtn MeSH: 1-99 100-198 199-297 298-396 397-495 496-593 | Itgb3 Description: Vtnintegrin beta 3 GO: 1-51 52-102 Reactome: 1-30 UniProtKB: 1 STRING: 1 RefEx: Vtn Expression Atlas: Itgb3 Single Cell Expression Atlas: Itgb3 PanglaoDB: Itgb3 MeSH: 1-95 96-190 191-285 286-380 381-475 476-570 571-665 666-760 761-855 856-950 951-1045 1046-1140 1141-1235 1236-1330 1331-1425 1426-1506 |  |  | 0.01 (3.361%) | 0 | 0 | DLRP | 1 |
| 11 | Bmp5 Description: Bmp5bone morphogenetic protein 5 GO: 1-38 UniProtKB: 1-2 STRING: 1 RefEx: Bmp5 Expression Atlas: Bmp5 Single Cell Expression Atlas: Bmp5 PanglaoDB: Bmp5 MeSH: 1-93 94-186 187-279 280-372 373-465 466-558 559-651 652-741 | Acvr2a Description: Bmp5activin receptor IIA GO: 1-53 Reactome: 1-4 UniProtKB: 1-3 STRING: 1 RefEx: Bmp5 Expression Atlas: Acvr2a Single Cell Expression Atlas: Acvr2a PanglaoDB: Acvr2a MeSH: 1-88 89-176 177-264 265-352 353-440 441-528 529-610 |  |  | 0.009 (3.058%) | 0 | 0 | DLRP | 1 |
| 12 | Nlgn3 Description: Nlgn3neuroligin 3 GO: 1-59 Reactome: 1-3 UniProtKB: 1 RefEx: Nlgn3 Expression Atlas: Nlgn3 Single Cell Expression Atlas: Nlgn3 PanglaoDB: Nlgn3 MeSH: 1-82 83-164 165-246 247-328 329-408 | Nrxn3 Description: Nlgn3neurexin III GO: 1-18 Reactome: 1-3 UniProtKB: 1-2 RefEx: Nlgn3 Expression Atlas: Nrxn3 Single Cell Expression Atlas: Nrxn3 PanglaoDB: Nrxn3 MeSH: 1-78 79-156 157-234 235-311 |  |  | 0.008 (2.724%) | 0 | 0 | DLRP | 1 |

# (2,4,) -related Enrichment Analysis

```
load("reanalysis.RData")
library("plotly")
```

## MeSH-Enrichment Analysis (A : Anatomy)

```
negLogPval <- -log10(out.vecLR[['pattern_2_4']]$Enrich$MeSH_A$Pvalue + 1E-10)
term <- out.vecLR[['pattern_2_4']]$Enrich$MeSH_A$Term
target <- seq_len(min(100, length(negLogPval)))
negLogPval <- negLogPval[target]
term <- term[target]
p <- plot_ly(x=seq_along(negLogPval), y=~negLogPval,
type="bar", color=~negLogPval, text=term,
colors=c("#4b61ba", "gray", "#a87963", "red"))
layout(p, title="MeSH-Enrichment Analysis (A)",
   xaxis=list(title="Term"),
   yaxis=list(title="-Log(P-value)"),
   legend=list(name=""))
```

```
## Warning: textfont.color doesn't (yet) support data arrays

## Warning: textfont.color doesn't (yet) support data arrays
```

## MeSH-Enrichment Analysis (B : Organisms)

```
negLogPval <- -log10(out.vecLR[['pattern_2_4']]$Enrich$MeSH_B$Pvalue + 1E-10)
term <- out.vecLR[['pattern_2_4']]$Enrich$MeSH_B$Term
target <- seq_len(min(100, length(negLogPval)))
negLogPval <- negLogPval[target]
term <- term[target]
p <- plot_ly(x=seq_along(negLogPval), y=~negLogPval,
type="bar", color=~negLogPval, text=term,
colors=c("#4b61ba", "gray", "#a87963", "red"))
layout(p, title="MeSH-Enrichment Analysis (B)",
   xaxis=list(title="Term"),
   yaxis=list(title="-Log(P-value)"),
   legend=list(name=""))
```

```
## Warning: textfont.color doesn't (yet) support data arrays

## Warning: textfont.color doesn't (yet) support data arrays
```

## MeSH-Enrichment Analysis (C : Diseases)

```
negLogPval <- -log10(out.vecLR[['pattern_2_4']]$Enrich$MeSH_C$Pvalue + 1E-10)
term <- out.vecLR[['pattern_2_4']]$Enrich$MeSH_C$Term
target <- seq_len(min(100, length(negLogPval)))
negLogPval <- negLogPval[target]
term <- term[target]
p <- plot_ly(x=seq_along(negLogPval), y=~negLogPval,
type="bar", color=~negLogPval, text=term,
colors=c("#4b61ba", "gray", "#a87963", "red"))
layout(p, title="MeSH-Enrichment Analysis (C)",
   xaxis=list(title="Term"),
   yaxis=list(title="-Log(P-value)"),
   legend=list(name=""))
```

```
## Warning: textfont.color doesn't (yet) support data arrays

## Warning: textfont.color doesn't (yet) support data arrays
```

## MeSH-Enrichment Analysis (D : Drugs)

```
negLogPval <- -log10(out.vecLR[['pattern_2_4']]$Enrich$MeSH_D$Pvalue + 1E-10)
term <- out.vecLR[['pattern_2_4']]$Enrich$MeSH_D$Term
target <- seq_len(min(100, length(negLogPval)))
negLogPval <- negLogPval[target]
term <- term[target]
p <- plot_ly(x=seq_along(negLogPval), y=~negLogPval,
type="bar", color=~negLogPval, text=term,
colors=c("#4b61ba", "gray", "#a87963", "red"))
layout(p, title="MeSH-Enrichment Analysis (D)",
   xaxis=list(title="Term"),
   yaxis=list(title="-Log(P-value)"),
   legend=list(name=""))
```

```
## Warning: textfont.color doesn't (yet) support data arrays

## Warning: textfont.color doesn't (yet) support data arrays
```

## MeSH-Enrichment Analysis (E : Analytical, Diagnostic and Therapeutic Techniques and Equipment)

```
negLogPval <- -log10(out.vecLR[['pattern_2_4']]$Enrich$MeSH_E$Pvalue + 1E-10)
term <- out.vecLR[['pattern_2_4']]$Enrich$MeSH_E$Term
target <- seq_len(min(100, length(negLogPval)))
negLogPval <- negLogPval[target]
term <- term[target]
p <- plot_ly(x=seq_along(negLogPval), y=~negLogPval,
type="bar", color=~negLogPval, text=term,
colors=c("#4b61ba", "gray", "#a87963", "red"))
layout(p, title="MeSH-Enrichment Analysis (E)",
   xaxis=list(title="Term"),
   yaxis=list(title="-Log(P-value)"),
   legend=list(name=""))
```

```
## Warning: textfont.color doesn't (yet) support data arrays

## Warning: textfont.color doesn't (yet) support data arrays
```

## MeSH-Enrichment Analysis (F : Psychiatry and Psychology)

```
negLogPval <- -log10(out.vecLR[['pattern_2_4']]$Enrich$MeSH_F$Pvalue + 1E-10)
term <- out.vecLR[['pattern_2_4']]$Enrich$MeSH_F$Term
target <- seq_len(min(100, length(negLogPval)))
negLogPval <- negLogPval[target]
term <- term[target]
p <- plot_ly(x=seq_along(negLogPval), y=~negLogPval,
type="bar", color=~negLogPval, text=term,
colors=c("#4b61ba", "gray", "#a87963", "red"))
layout(p, title="MeSH-Enrichment Analysis (F)",
   xaxis=list(title="Term"),
   yaxis=list(title="-Log(P-value)"),
   legend=list(name=""))
```

```
## Warning: textfont.color doesn't (yet) support data arrays

## Warning: textfont.color doesn't (yet) support data arrays
```

## MeSH-Enrichment Analysis (G : Phenomena and Processes)

```
negLogPval <- -log10(out.vecLR[['pattern_2_4']]$Enrich$MeSH_G$Pvalue + 1E-10)
term <- out.vecLR[['pattern_2_4']]$Enrich$MeSH_G$Term
target <- seq_len(min(100, length(negLogPval)))
negLogPval <- negLogPval[target]
term <- term[target]
p <- plot_ly(x=seq_along(negLogPval), y=~negLogPval,
type="bar", color=~negLogPval, text=term,
colors=c("#4b61ba", "gray", "#a87963", "red"))
layout(p, title="MeSH-Enrichment Analysis (G)",
   xaxis=list(title="Term"),
   yaxis=list(title="-Log(P-value)"),
   legend=list(name=""))
```

```
## Warning: textfont.color doesn't (yet) support data arrays

## Warning: textfont.color doesn't (yet) support data arrays
```

## MeSH-Enrichment Analysis (H : Disciplines and Occupations)

```
negLogPval <- -log10(out.vecLR[['pattern_2_4']]$Enrich$MeSH_H$Pvalue + 1E-10)
term <- out.vecLR[['pattern_2_4']]$Enrich$MeSH_H$Term
target <- seq_len(min(100, length(negLogPval)))
negLogPval <- negLogPval[target]
term <- term[target]
p <- plot_ly(x=seq_along(negLogPval), y=~negLogPval,
type="bar", color=~negLogPval, text=term,
colors=c("#4b61ba", "gray", "#a87963", "red"))
layout(p, title="MeSH-Enrichment Analysis (H)",
   xaxis=list(title="Term"),
   yaxis=list(title="-Log(P-value)"),
   legend=list(name=""))
```

```
## Warning: textfont.color doesn't (yet) support data arrays

## Warning: textfont.color doesn't (yet) support data arrays
```

## MeSH-Enrichment Analysis (I : Anthropology, Education, Sociology and Social Phenomena)

```
negLogPval <- -log10(out.vecLR[['pattern_2_4']]$Enrich$MeSH_I$Pvalue + 1E-10)
term <- out.vecLR[['pattern_2_4']]$Enrich$MeSH_I$Term
target <- seq_len(min(100, length(negLogPval)))
negLogPval <- negLogPval[target]
term <- term[target]
p <- plot_ly(x=seq_along(negLogPval), y=~negLogPval,
type="bar", color=~negLogPval, text=term,
colors=c("#4b61ba", "gray", "#a87963", "red"))
layout(p, title="MeSH-Enrichment Analysis (I)",
   xaxis=list(title="Term"),
   yaxis=list(title="-Log(P-value)"),
   legend=list(name=""))
```

## MeSH-Enrichment Analysis (J : Technology and Food and Beverages)

```
negLogPval <- -log10(out.vecLR[['pattern_2_4']]$Enrich$MeSH_J$Pvalue + 1E-10)
term <- out.vecLR[['pattern_2_4']]$Enrich$MeSH_J$Term
target <- seq_len(min(100, length(negLogPval)))
negLogPval <- negLogPval[target]
term <- term[target]
p <- plot_ly(x=seq_along(negLogPval), y=~negLogPval,
type="bar", color=~negLogPval, text=term,
colors=c("#4b61ba", "gray", "#a87963", "red"))
layout(p, title="MeSH-Enrichment Analysis (J)",
   xaxis=list(title="Term"),
   yaxis=list(title="-Log(P-value)"),
   legend=list(name=""))
```

## MeSH-Enrichment Analysis (L : Information Science)

```
negLogPval <- -log10(out.vecLR[['pattern_2_4']]$Enrich$MeSH_L$Pvalue + 1E-10)
term <- out.vecLR[['pattern_2_4']]$Enrich$MeSH_L$Term
target <- seq_len(min(100, length(negLogPval)))
negLogPval <- negLogPval[target]
term <- term[target]
p <- plot_ly(x=seq_along(negLogPval), y=~negLogPval,
type="bar", color=~negLogPval, text=term,
colors=c("#4b61ba", "gray", "#a87963", "red"))
layout(p, title="MeSH-Enrichment Analysis (L)",
   xaxis=list(title="Term"),
   yaxis=list(title="-Log(P-value)"),
   legend=list(name=""))
```

```
## Warning: textfont.color doesn't (yet) support data arrays

## Warning: textfont.color doesn't (yet) support data arrays
```

## Reactome Pathway-Enrichment Analysis

```
negLogPval <- -log10(out.vecLR[['pattern_2_4']]$Enrich$Reactome$Pvalue + 1E-10)
term <- out.vecLR[['pattern_2_4']]$Enrich$Reactome$Term
target <- seq_len(min(100, length(negLogPval)))
negLogPval <- negLogPval[target]
term <- term[target]
p <- plot_ly(x=seq_along(negLogPval), y=~negLogPval,
type="bar", color=~negLogPval, text=term,
colors=c("#4b61ba", "gray", "#a87963", "red"))
layout(p, title="Reactome-Enrichment Analysis",
   xaxis=list(title="Term"),
   yaxis=list(title="-Log(P-value)"),
   legend=list(name=""))
```

```
## Warning: textfont.color doesn't (yet) support data arrays

## Warning: textfont.color doesn't (yet) support data arrays
```
