# Supplementary material for: Sctensor detects many-to-many cell–cell interactions from single cell RNA-sequencing data
Source: BMC Bioinformatics. 2023 Nov 7;24:420. doi: 10.1186/s12859-023-05490-y (PMC10631077; doi:10.1186/s12859-023-05490-y)
Supplement: Supplementary file 18 — Additional file 18. HTML report of VisualCortex. [file 12859_2023_5490_MOESM18_ESM.zip › output/report/Mouse_VisualCortex/pattern_4_6.html]

Details of (4,6,) -related L-R pairs


# Details of (4,6,) -related L-R pairs

# (4,6,:) Pattern

# (4,6,) -related L-R pairs (Top9 pairs)

```
library("scTensor")
load("reanalysis.RData")
col <- .setColor("many")[34]
scTensor:::.myvisNetwork(g, col)
```

| Rank | Ligand Gene | Receptor Gene | Ligand Expression (Log10(exp + 1)) | Receptor Expression (Log10(exp + 1)) | LR-pair factor value (Percentage) | P-value (Grubbs test) | Q-value (BH method) | L-R Evidence cf. rikenbit/lrbase-workflow | PubMed |
| --- | --- | --- | --- | --- | --- | --- | --- | --- | --- |
| 1 | Jag1 Description: Jag1jagged 1 GO: 1-50 Reactome: 1-9 UniProtKB: 1-2 STRING: 1 RefEx: Jag1 Expression Atlas: Jag1 Single Cell Expression Atlas: Jag1 PanglaoDB: Jag1 MeSH: 1-100 101-200 201-300 301-400 401-500 501-600 601-700 701-800 801-900 901-1000 1001-1100 1101-1200 1201-1300 1301-1400 1401-1500 1501-1600 1601-1700 1701-1800 1801-1897 | Notch3 Description: Jag1notch 3 GO: 1-30 Reactome: 1-11 UniProtKB: 1 STRING: 1 2 RefEx: Jag1 Expression Atlas: Notch3 Single Cell Expression Atlas: Notch3 PanglaoDB: Notch3 MeSH: 1-94 95-188 189-282 283-376 377-470 471-564 565-658 659-752 753-846 847-940 941-1034 1035-1120 |  |  | 0.059 (21.008%) | 0 | 0 | DLRP | 1 |
| 2 | Pdgfb Description: Pdgfbplatelet derived growth factor, B polypeptide GO: 1-53 54-106 Reactome: 1-17 UniProtKB: 1-2 STRING: 1 2 3 RefEx: Pdgfb Expression Atlas: Pdgfb Single Cell Expression Atlas: Pdgfb PanglaoDB: Pdgfb MeSH: 1-95 96-190 191-285 286-380 381-475 476-570 571-665 666-760 761-855 856-950 951-1045 1046-1140 1141-1224 | Pdgfrb Description: Pdgfbplatelet derived growth factor receptor, beta polypeptide GO: 1-100 Reactome: 1-11 UniProtKB: 1-2 RefEx: Pdgfb Expression Atlas: Pdgfrb Single Cell Expression Atlas: Pdgfrb PanglaoDB: Pdgfrb MeSH: 1-99 100-198 199-297 298-396 397-495 496-594 595-693 694-792 793-891 892-990 991-1089 1090-1188 1189-1287 1288-1386 1387-1483 |  |  | 0.05 (17.835%) | 0 | 0 | DLRP | 1 |
| 3 | Sema4d Description: Sema4dsema domain, immunoglobulin domain (Ig), transmembrane domain (TM) and short cytoplasmic domain, (semaphorin) 4D GO: 1-39 Reactome: 1-8 UniProtKB: 1 RefEx: Sema4d Expression Atlas: Sema4d Single Cell Expression Atlas: Sema4d PanglaoDB: Sema4d MeSH: 1-89 90-178 179-267 268-356 357-445 446-531 | Plxnb1 Description: Sema4dplexin B1 GO: 1-24 Reactome: 1-15 UniProtKB: 1-2 RefEx: Sema4d Expression Atlas: Plxnb1 Single Cell Expression Atlas: Plxnb1 PanglaoDB: Plxnb1 MeSH: 1-83 84-166 167-249 250-332 333-415 |  |  | 0.029 (10.257%) | 0 | 0 | DLRP | 1 |
| 4 | Dll4 Description: Dll4delta like canonical Notch ligand 4 GO: 1-41 Reactome: 1-4 UniProtKB: 1 STRING: 1 RefEx: Dll4 Expression Atlas: Dll4 Single Cell Expression Atlas: Dll4 PanglaoDB: Dll4 MeSH: 1-93 94-186 187-279 280-372 373-465 466-558 559-651 652-744 745-837 838-922 | Notch3 Description: Dll4notch 3 GO: 1-30 Reactome: 1-11 UniProtKB: 1 STRING: 1 2 RefEx: Dll4 Expression Atlas: Notch3 Single Cell Expression Atlas: Notch3 PanglaoDB: Notch3 MeSH: 1-94 95-188 189-282 283-376 377-470 471-564 565-658 659-752 753-846 847-940 941-1034 1035-1120 |  |  | 0.019 (6.646%) | 0 | 0 | DLRP | 1 |
| 5 | Pdgfd Description: Pdgfdplatelet-derived growth factor, D polypeptide GO: 1-24 Reactome: 1-3 UniProtKB: 1 RefEx: Pdgfd Expression Atlas: Pdgfd Single Cell Expression Atlas: Pdgfd PanglaoDB: Pdgfd MeSH: 1-86 87-172 173-258 259-343 | Pdgfrb Description: Pdgfdplatelet derived growth factor receptor, beta polypeptide GO: 1-100 Reactome: 1-11 UniProtKB: 1-2 RefEx: Pdgfd Expression Atlas: Pdgfrb Single Cell Expression Atlas: Pdgfrb PanglaoDB: Pdgfrb MeSH: 1-99 100-198 199-297 298-396 397-495 496-594 595-693 694-792 793-891 892-990 991-1089 1090-1188 1189-1287 1288-1386 1387-1483 |  |  | 0.016 (5.753%) | 0 | 0 | DLRP | 1 |
| 6 | Vegfa Description: Vegfavascular endothelial growth factor A GO: 1-87 88-173 Reactome: 1-11 UniProtKB: 1-3 RefEx: Vegfa Expression Atlas: Vegfa Single Cell Expression Atlas: Vegfa PanglaoDB: Vegfa MeSH: 1-99 100-198 199-297 298-396 397-495 496-594 595-693 694-792 793-891 892-990 991-1089 1090-1188 1189-1287 1288-1386 1387-1485 1486-1584 1585-1683 1684-1782 1783-1881 1882-1980 1981-2079 2080-2178 2179-2277 2278-2376 2377-2475 2476-2574 2575-2673 2674-2772 2773-2871 2872-2970 2971-3069 3070-3168 3169-3255 | Flt1 Description: VegfaFMS-like tyrosine kinase 1 GO: 1-59 Reactome: 1-6 UniProtKB: 1-2 RefEx: Vegfa Expression Atlas: Flt1 Single Cell Expression Atlas: Flt1 PanglaoDB: Flt1 MeSH: 1-96 97-192 193-288 289-384 385-480 481-576 577-672 673-768 769-864 865-960 961-1056 1057-1152 1153-1248 1249-1344 1345-1440 1441-1526 |  |  | 0.015 (5.416%) | 0 | 0 | DLRP | 1 |
| 7 | Edn1 Description: Edn1endothelin 1 GO: 1-61 62-121 Reactome: 1-7 UniProtKB: 1-2 STRING: 1 RefEx: Edn1 Expression Atlas: Edn1 Single Cell Expression Atlas: Edn1 PanglaoDB: Edn1 MeSH: 1-98 99-196 197-294 295-392 393-490 491-588 589-686 687-784 785-882 883-980 981-1078 1079-1176 1177-1274 1275-1369 | Ednra Description: Edn1endothelin receptor type A GO: 1-58 59-116 Reactome: 1-7 UniProtKB: 1 STRING: 1 RefEx: Edn1 Expression Atlas: Ednra Single Cell Expression Atlas: Ednra PanglaoDB: Ednra MeSH: 1-90 91-180 181-270 271-360 361-450 451-540 541-630 631-717 |  |  | 0.012 (4.188%) | 0.001 | 0.001 | DLRP | 1 |
| 8 | Wnt5a Description: Wnt5awingless-type MMTV integration site family, member 5A GO: 1-92 93-183 Reactome: 1-14 UniProtKB: 1 RefEx: Wnt5a Expression Atlas: Wnt5a Single Cell Expression Atlas: Wnt5a PanglaoDB: Wnt5a MeSH: 1-96 97-192 193-288 289-384 385-480 481-576 577-672 673-768 769-864 865-960 961-1056 1057-1152 1153-1248 1249-1344 1345-1440 1441-1531 | Fzd6 Description: Wnt5afrizzled class receptor 6 GO: 1-35 Reactome: 1-7 UniProtKB: 1-3 RefEx: Wnt5a Expression Atlas: Fzd6 Single Cell Expression Atlas: Fzd6 PanglaoDB: Fzd6 MeSH: 1-94 95-188 189-282 283-376 377-470 471-563 |  |  | 0.011 (4.027%) | 0.001 | 0.001 | DLRP | 1 |
| 9 | Pthlh Description: Pthlhparathyroid hormone-like peptide GO: 1-30 UniProtKB: 1-2 STRING: 1 2 RefEx: Pthlh Expression Atlas: Pthlh Single Cell Expression Atlas: Pthlh PanglaoDB: Pthlh MeSH: 1-93 94-186 187-279 280-372 373-465 466-558 559-651 652-744 745-837 838-930 931-1015 | Pth1r Description: Pthlhparathyroid hormone 1 receptor GO: 1-38 Reactome: 1-6 UniProtKB: 1 RefEx: Pthlh Expression Atlas: Pth1r Single Cell Expression Atlas: Pth1r PanglaoDB: Pth1r MeSH: 1-95 96-190 191-285 286-380 381-475 476-570 571-665 666-760 761-851 |  |  | 0.011 (3.928%) | 0 | 0 | DLRP | 1 |

# (4,6,) -related Enrichment Analysis

```
load("reanalysis.RData")
library("plotly")
```

## MeSH-Enrichment Analysis (A : Anatomy)

```
negLogPval <- -log10(out.vecLR[['pattern_4_6']]$Enrich$MeSH_A$Pvalue + 1E-10)
term <- out.vecLR[['pattern_4_6']]$Enrich$MeSH_A$Term
target <- seq_len(min(100, length(negLogPval)))
negLogPval <- negLogPval[target]
term <- term[target]
p <- plot_ly(x=seq_along(negLogPval), y=~negLogPval,
type="bar", color=~negLogPval, text=term,
colors=c("#4b61ba", "gray", "#a87963", "red"))
layout(p, title="MeSH-Enrichment Analysis (A)",
   xaxis=list(title="Term"),
   yaxis=list(title="-Log(P-value)"),
   legend=list(name=""))
```

```
## Warning: textfont.color doesn't (yet) support data arrays

## Warning: textfont.color doesn't (yet) support data arrays
```

## MeSH-Enrichment Analysis (B : Organisms)

```
negLogPval <- -log10(out.vecLR[['pattern_4_6']]$Enrich$MeSH_B$Pvalue + 1E-10)
term <- out.vecLR[['pattern_4_6']]$Enrich$MeSH_B$Term
target <- seq_len(min(100, length(negLogPval)))
negLogPval <- negLogPval[target]
term <- term[target]
p <- plot_ly(x=seq_along(negLogPval), y=~negLogPval,
type="bar", color=~negLogPval, text=term,
colors=c("#4b61ba", "gray", "#a87963", "red"))
layout(p, title="MeSH-Enrichment Analysis (B)",
   xaxis=list(title="Term"),
   yaxis=list(title="-Log(P-value)"),
   legend=list(name=""))
```

```
## Warning: textfont.color doesn't (yet) support data arrays

## Warning: textfont.color doesn't (yet) support data arrays
```

## MeSH-Enrichment Analysis (C : Diseases)

```
negLogPval <- -log10(out.vecLR[['pattern_4_6']]$Enrich$MeSH_C$Pvalue + 1E-10)
term <- out.vecLR[['pattern_4_6']]$Enrich$MeSH_C$Term
target <- seq_len(min(100, length(negLogPval)))
negLogPval <- negLogPval[target]
term <- term[target]
p <- plot_ly(x=seq_along(negLogPval), y=~negLogPval,
type="bar", color=~negLogPval, text=term,
colors=c("#4b61ba", "gray", "#a87963", "red"))
layout(p, title="MeSH-Enrichment Analysis (C)",
   xaxis=list(title="Term"),
   yaxis=list(title="-Log(P-value)"),
   legend=list(name=""))
```

```
## Warning: textfont.color doesn't (yet) support data arrays

## Warning: textfont.color doesn't (yet) support data arrays
```

## MeSH-Enrichment Analysis (D : Drugs)

```
negLogPval <- -log10(out.vecLR[['pattern_4_6']]$Enrich$MeSH_D$Pvalue + 1E-10)
term <- out.vecLR[['pattern_4_6']]$Enrich$MeSH_D$Term
target <- seq_len(min(100, length(negLogPval)))
negLogPval <- negLogPval[target]
term <- term[target]
p <- plot_ly(x=seq_along(negLogPval), y=~negLogPval,
type="bar", color=~negLogPval, text=term,
colors=c("#4b61ba", "gray", "#a87963", "red"))
layout(p, title="MeSH-Enrichment Analysis (D)",
   xaxis=list(title="Term"),
   yaxis=list(title="-Log(P-value)"),
   legend=list(name=""))
```

```
## Warning: textfont.color doesn't (yet) support data arrays

## Warning: textfont.color doesn't (yet) support data arrays
```

## MeSH-Enrichment Analysis (E : Analytical, Diagnostic and Therapeutic Techniques and Equipment)

```
negLogPval <- -log10(out.vecLR[['pattern_4_6']]$Enrich$MeSH_E$Pvalue + 1E-10)
term <- out.vecLR[['pattern_4_6']]$Enrich$MeSH_E$Term
target <- seq_len(min(100, length(negLogPval)))
negLogPval <- negLogPval[target]
term <- term[target]
p <- plot_ly(x=seq_along(negLogPval), y=~negLogPval,
type="bar", color=~negLogPval, text=term,
colors=c("#4b61ba", "gray", "#a87963", "red"))
layout(p, title="MeSH-Enrichment Analysis (E)",
   xaxis=list(title="Term"),
   yaxis=list(title="-Log(P-value)"),
   legend=list(name=""))
```

```
## Warning: textfont.color doesn't (yet) support data arrays

## Warning: textfont.color doesn't (yet) support data arrays
```

## MeSH-Enrichment Analysis (F : Psychiatry and Psychology)

```
negLogPval <- -log10(out.vecLR[['pattern_4_6']]$Enrich$MeSH_F$Pvalue + 1E-10)
term <- out.vecLR[['pattern_4_6']]$Enrich$MeSH_F$Term
target <- seq_len(min(100, length(negLogPval)))
negLogPval <- negLogPval[target]
term <- term[target]
p <- plot_ly(x=seq_along(negLogPval), y=~negLogPval,
type="bar", color=~negLogPval, text=term,
colors=c("#4b61ba", "gray", "#a87963", "red"))
layout(p, title="MeSH-Enrichment Analysis (F)",
   xaxis=list(title="Term"),
   yaxis=list(title="-Log(P-value)"),
   legend=list(name=""))
```

```
## Warning: textfont.color doesn't (yet) support data arrays

## Warning: textfont.color doesn't (yet) support data arrays
```

## MeSH-Enrichment Analysis (G : Phenomena and Processes)

```
negLogPval <- -log10(out.vecLR[['pattern_4_6']]$Enrich$MeSH_G$Pvalue + 1E-10)
term <- out.vecLR[['pattern_4_6']]$Enrich$MeSH_G$Term
target <- seq_len(min(100, length(negLogPval)))
negLogPval <- negLogPval[target]
term <- term[target]
p <- plot_ly(x=seq_along(negLogPval), y=~negLogPval,
type="bar", color=~negLogPval, text=term,
colors=c("#4b61ba", "gray", "#a87963", "red"))
layout(p, title="MeSH-Enrichment Analysis (G)",
   xaxis=list(title="Term"),
   yaxis=list(title="-Log(P-value)"),
   legend=list(name=""))
```

```
## Warning: textfont.color doesn't (yet) support data arrays

## Warning: textfont.color doesn't (yet) support data arrays
```

## MeSH-Enrichment Analysis (H : Disciplines and Occupations)

```
negLogPval <- -log10(out.vecLR[['pattern_4_6']]$Enrich$MeSH_H$Pvalue + 1E-10)
term <- out.vecLR[['pattern_4_6']]$Enrich$MeSH_H$Term
target <- seq_len(min(100, length(negLogPval)))
negLogPval <- negLogPval[target]
term <- term[target]
p <- plot_ly(x=seq_along(negLogPval), y=~negLogPval,
type="bar", color=~negLogPval, text=term,
colors=c("#4b61ba", "gray", "#a87963", "red"))
layout(p, title="MeSH-Enrichment Analysis (H)",
   xaxis=list(title="Term"),
   yaxis=list(title="-Log(P-value)"),
   legend=list(name=""))
```

## MeSH-Enrichment Analysis (J : Technology and Food and Beverages)

```
negLogPval <- -log10(out.vecLR[['pattern_4_6']]$Enrich$MeSH_J$Pvalue + 1E-10)
term <- out.vecLR[['pattern_4_6']]$Enrich$MeSH_J$Term
target <- seq_len(min(100, length(negLogPval)))
negLogPval <- negLogPval[target]
term <- term[target]
p <- plot_ly(x=seq_along(negLogPval), y=~negLogPval,
type="bar", color=~negLogPval, text=term,
colors=c("#4b61ba", "gray", "#a87963", "red"))
layout(p, title="MeSH-Enrichment Analysis (J)",
   xaxis=list(title="Term"),
   yaxis=list(title="-Log(P-value)"),
   legend=list(name=""))
```

## MeSH-Enrichment Analysis (L : Information Science)

```
negLogPval <- -log10(out.vecLR[['pattern_4_6']]$Enrich$MeSH_L$Pvalue + 1E-10)
term <- out.vecLR[['pattern_4_6']]$Enrich$MeSH_L$Term
target <- seq_len(min(100, length(negLogPval)))
negLogPval <- negLogPval[target]
term <- term[target]
p <- plot_ly(x=seq_along(negLogPval), y=~negLogPval,
type="bar", color=~negLogPval, text=term,
colors=c("#4b61ba", "gray", "#a87963", "red"))
layout(p, title="MeSH-Enrichment Analysis (L)",
   xaxis=list(title="Term"),
   yaxis=list(title="-Log(P-value)"),
   legend=list(name=""))
```

```
## Warning: textfont.color doesn't (yet) support data arrays

## Warning: textfont.color doesn't (yet) support data arrays
```

## MeSH-Enrichment Analysis (M : Persons)

```
negLogPval <- -log10(out.vecLR[['pattern_4_6']]$Enrich$MeSH_M$Pvalue + 1E-10)
term <- out.vecLR[['pattern_4_6']]$Enrich$MeSH_M$Term
target <- seq_len(min(100, length(negLogPval)))
negLogPval <- negLogPval[target]
term <- term[target]
p <- plot_ly(x=seq_along(negLogPval), y=~negLogPval,
type="bar", color=~negLogPval, text=term,
colors=c("#4b61ba", "gray", "#a87963", "red"))
layout(p, title="MeSH-Enrichment Analysis (M)",
   xaxis=list(title="Term"),
   yaxis=list(title="-Log(P-value)"),
   legend=list(name=""))
```

## MeSH-Enrichment Analysis (N : Health Care)

```
negLogPval <- -log10(out.vecLR[['pattern_4_6']]$Enrich$MeSH_N$Pvalue + 1E-10)
term <- out.vecLR[['pattern_4_6']]$Enrich$MeSH_N$Term
target <- seq_len(min(100, length(negLogPval)))
negLogPval <- negLogPval[target]
term <- term[target]
p <- plot_ly(x=seq_along(negLogPval), y=~negLogPval,
type="bar", color=~negLogPval, text=term,
colors=c("#4b61ba", "gray", "#a87963", "red"))
layout(p, title="MeSH-Enrichment Analysis (N)",
   xaxis=list(title="Term"),
   yaxis=list(title="-Log(P-value)"),
   legend=list(name=""))
```

```
## Warning: textfont.color doesn't (yet) support data arrays

## Warning: textfont.color doesn't (yet) support data arrays
```

## Reactome Pathway-Enrichment Analysis

```
negLogPval <- -log10(out.vecLR[['pattern_4_6']]$Enrich$Reactome$Pvalue + 1E-10)
term <- out.vecLR[['pattern_4_6']]$Enrich$Reactome$Term
target <- seq_len(min(100, length(negLogPval)))
negLogPval <- negLogPval[target]
term <- term[target]
p <- plot_ly(x=seq_along(negLogPval), y=~negLogPval,
type="bar", color=~negLogPval, text=term,
colors=c("#4b61ba", "gray", "#a87963", "red"))
layout(p, title="Reactome-Enrichment Analysis",
   xaxis=list(title="Term"),
   yaxis=list(title="-Log(P-value)"),
   legend=list(name=""))
```

```
## Warning: textfont.color doesn't (yet) support data arrays

## Warning: textfont.color doesn't (yet) support data arrays
```
